# Supplementary material for: AI-identified CD133-targeting natural compounds demonstrate differential anti-tumor effects and mechanisms in pan-cancer models
Source: EMBO Mol Med. 2025 Oct 2;17(11):2932–65. doi: 10.1038/s44321-025-00308-1 (PMC12603267; doi:10.1038/s44321-025-00308-1)
Supplement: Supplementary file 1 — Appendix [file 44321_2025_308_MOESM1_ESM.pdf]

Appendix

AI-Identified CD133-Targeting Natural Compounds Demonstrate Different Anti-tumor Effects and Mechanisms in Pan-cancer

Yibo Hou[1,†], Zixian Wang[1,†], Wenlin Wang[2,†], Qing Tang[2], Yongde Cai[3], Siyang Yu[1], Jin Wang[3], Xiu Yan[3], Guocai Wang[2], Peter E. Lobie[1], Yubo Zhang[2,*], Xiaoyong Dai[2,*], Shaohua Ma[1,*]

[1] Institute of Biopharmaceutical and Health Engineering, Tsinghua Shenzhen International Graduate School (SIGS), Tsinghua University, Shenzhen, 518055 China;

[2] Department of Physiology, School of Medicine; Institute of Traditional Chinese Medicine & Natural Products, College of Pharmacy, and Guangdong Province Key Laboratory of Pharmacodynamic Constituents of TCM and New Drugs Research, Jinan University, Guangzhou, 510632, China;

[3] Synorg Biotechnology (Shenzhen) Co. Ltd., Shenzhen, 518107, China.

[†] These authors contributed equally to this work.

[*] Correspondence: Yubo Zhang, ybzhang99@jnu.edu.cn; Xiaoyong Dai, daixy18@jnu.edu.cn; Shaohua Ma, ma.shaohua@sz.tsinghua.edu.cn.

Appendix	Description	Page
Appendix Figure S1	MTT results on cytotoxicity of PP10 and PP24	2
Appendix Figure S2	Quantification of relative expression level of EMT pathway protein after PP10 and PP24 treatment in CRC cells	3
Appendix Figure S3	In Vivo safety evaluation of PP10 and PP24 in CDX model	4
Appendix Figure S4	In Vivo safety evaluation of PP10 and PP24 in CRC-PDOX model.	5
Appendix Figure S5	In Vivo safety evaluation of PP10 and PP24 in liver cancer PDOX model	7
Appendix Figure S6	In Vivo safety evaluation of PP10 and PP24 in lung cancer PDOX model	9
Appendix table S1	*P* value in Figure 1-7	10
Appendix table S2	*P* value in Figure EV1-7	11
Appendix table S3	*P* value in AF S1-6	12

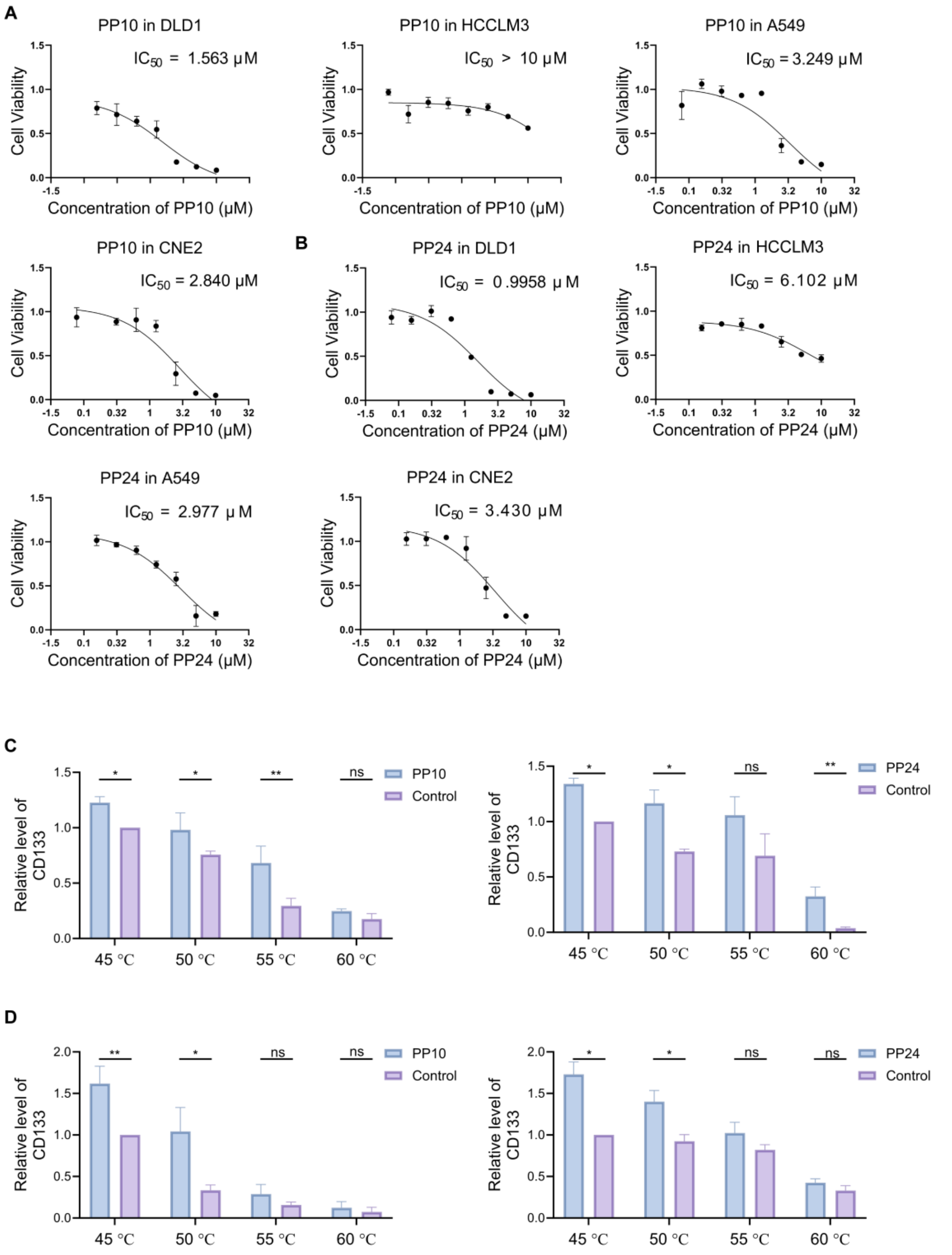

Appendix Figure S1 MTT results on cytotoxicity of PP10 and PP24. (A, B) MTT results of PP10 and PP24 on HCT 116, DLD1, CNE2, HCCLM3, and A549 cells. (C) Quantitative analysis of the level of CD133 in HCT116 cell line for Fig. 2I, J, normalized by Control-45°C. (D) Quantitative analysis of the level of CD133 in DLD1 cell line for Fig. 2I, J. Student's *t* test. Exact p- value are presented in Appendix table S3. Data are presented as mean ± SD. $^{*}P < 0.05$, $^{**}P < 0.01$, and $^{***}P < 0.001$, when compared with Control-45°C.group.

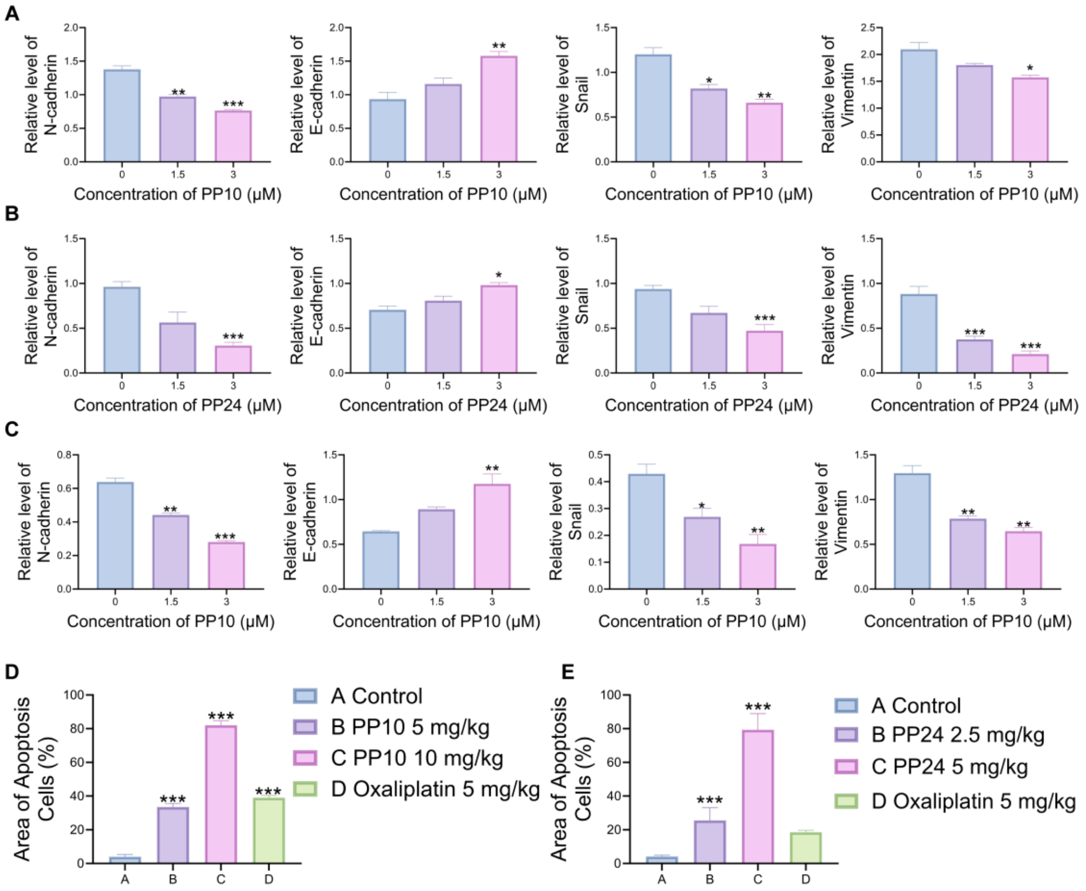

Appendix Figure S2 Quantification of relative expression level of EMT pathway protein after PP10 and PP24 treatment in CRC cells. (A, B) Quantification of relative expression levels to β-actin after PP10 and PP24 treatment in HCT116 cells (N=3). (C) Quantification of relative expression levels to β-actin after PP10 treatment in DLD1 cells (N=3). (D, E) Statistics of TUNEL stained cells after PP10 and PP24 treatment (N=3). one-way ANOVA test. Exact p-value are presented in Appendix table S3. Data are presented as mean ± SD. *$P < 0.05$, **$P < 0.01$, and ***$P < 0.001$, when compared with control group.

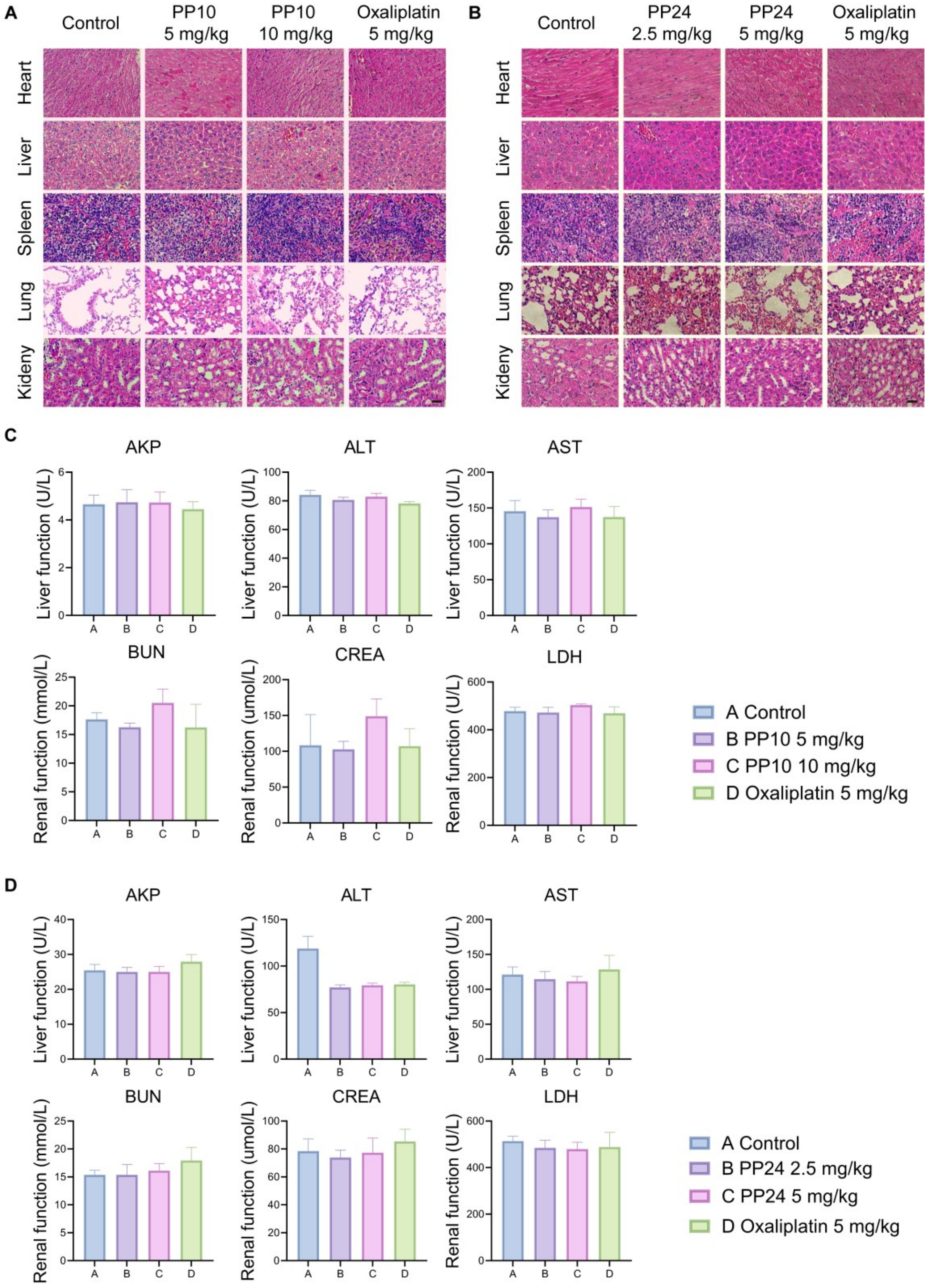

Appendix Figure S3 In *Vivo* safety evaluation of PP10 and PP24 in CDX model. (A, B) HE staining of sections of main organs acquired from CDX mice after PP10 and PP24 treatment. Effect of PP10 (C) and PP24 (D) on liver, kidney function in CDX model (N=3). liver function indicators: AKP, ALT, AST, and kidney function indicators: BUN, CREA, LDH. Scale bar, 50 μm. one-way ANOVA test. Exact p- value are presented in Appendix table S3. Data are presented as mean ± SD. **P* < 0.05, ***P* < 0.01, and ****P* < 0.001, when compared with control group.

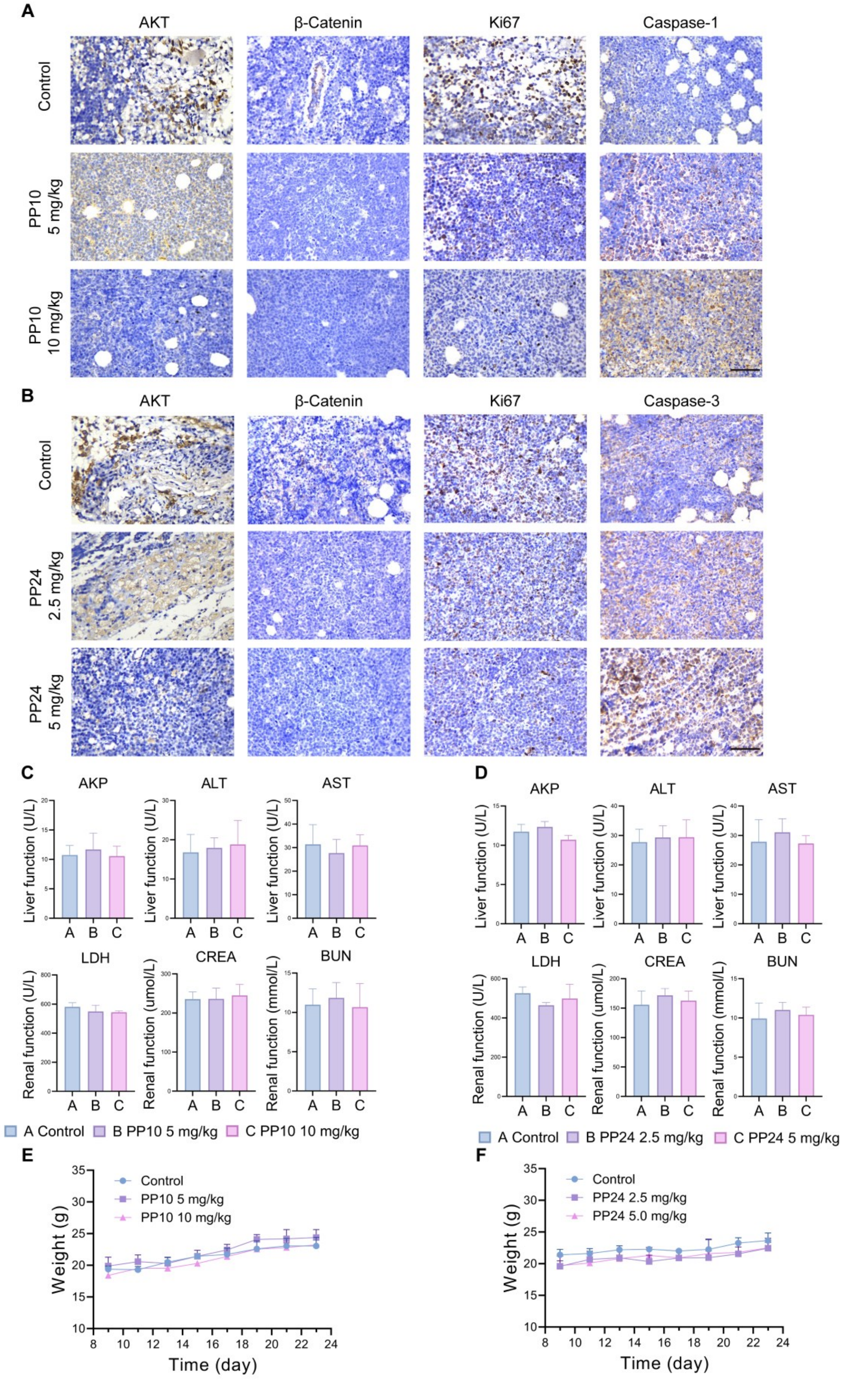

Appendix Figure S4 In *Vivo* safety evaluation of PP10 and PP24 in CRC-PDOX model. (A, B)

IHC staining of tumor tissue sections of CRC-PDOX model on AKT, β-Catenin, Ki67, and Caspase-3 after PP10 and PP24 treatment. (C, D) Effect of PP10 and PP24 on liver, kidney function in CRC-PDOX model (N=3). (E, F) Body weight of PP10 and PP24 treated mice during experiment (N = 4). Scale bar, 50 μm. one-way ANOVA test. Exact p- value are presented in Appendix table S3. Data are presented as mean ± SD. *$P < 0.05$, **$P < 0.01$, and ***$P < 0.001$, when compared with control group.

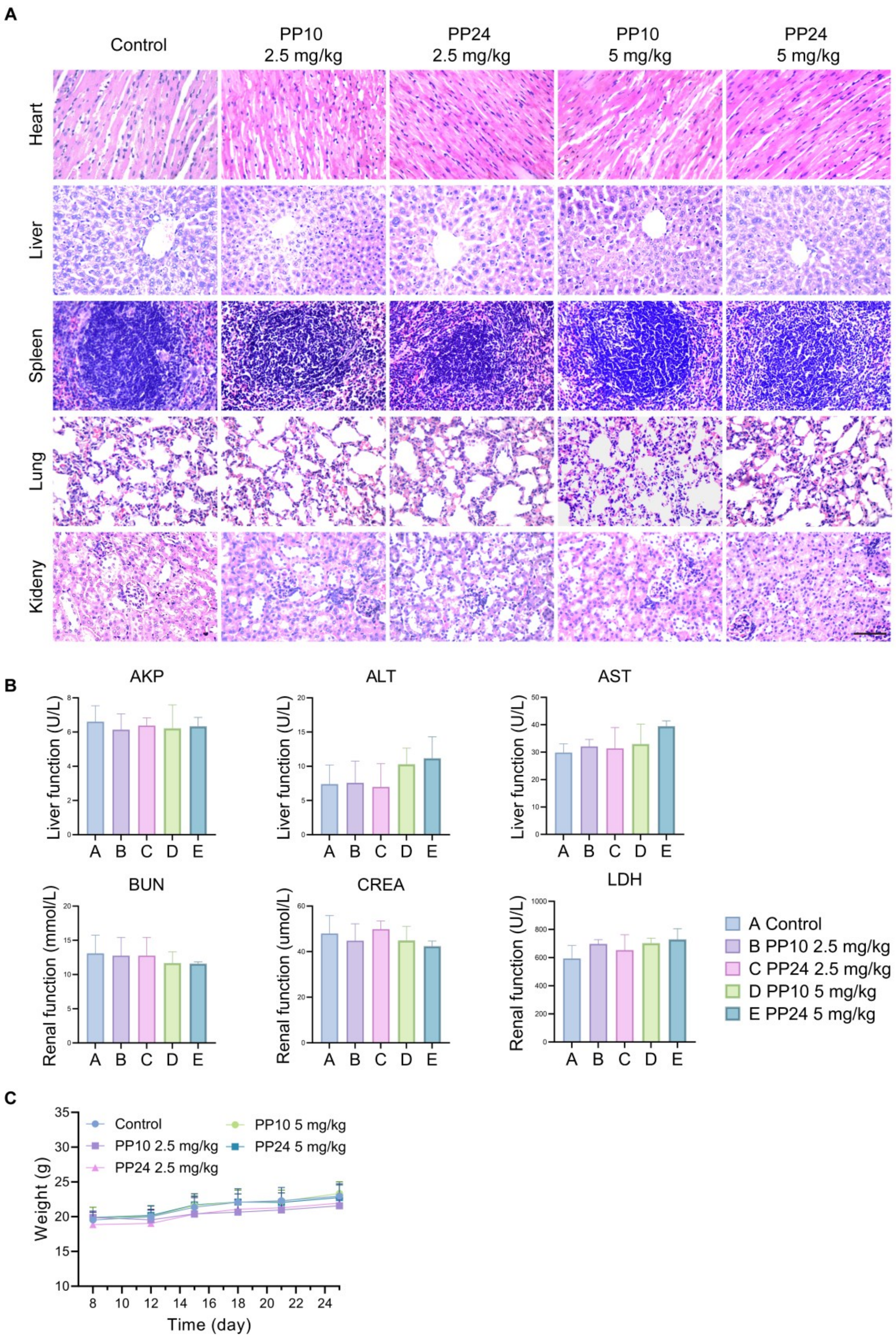

Appendix Figure S5 In *Vivo* safety evaluation of PP10 and PP24 in liver cancer PDOX model. (A) HE staining of sections of main organs acquired from liver cancer PDOX mode after PP10 and PP24 treatment. (B) Effect of PP10 and PP24 on liver, kidney function in CDX model (N=3). Scale bar, 50 μm. (C) Body weight of PP10 and PP24 treated mice during experiment

(N = 5). one-way ANOVA test. Exact p- value are presented in Appendix table S3. Data are presented as mean ± SD. *$P < 0.05$, **$P < 0.01$, and ***$P < 0.001$, when compared with control group.

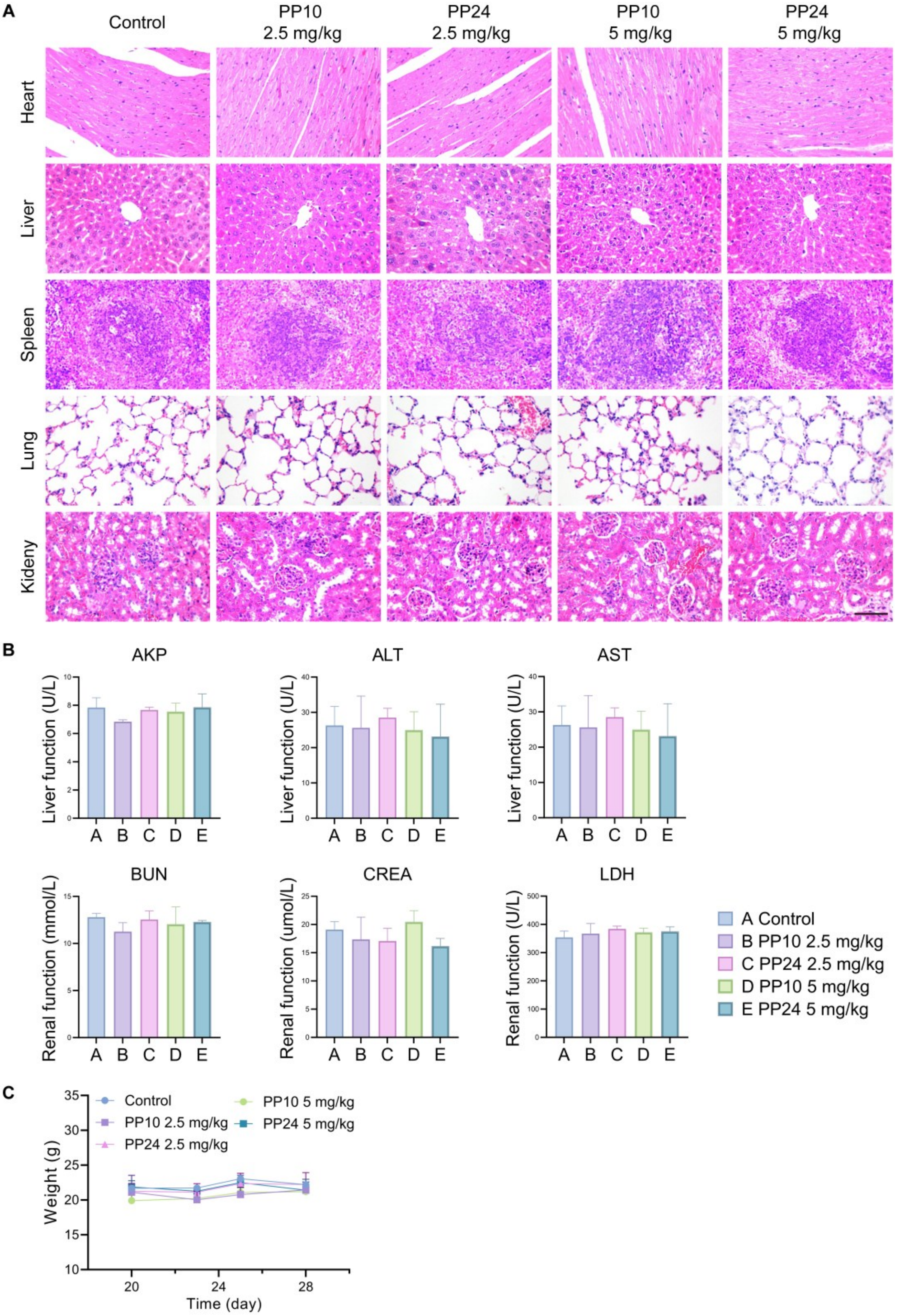

Appendix Figure S6 In *Vivo* safety evaluation of PP10 and PP24 in lung cancer PDOX model. (A) HE staining of sections of main organs acquired from lung cancer PDOX mode after PP10 and PP24 treatment. (B) Effect of PP10 and PP24 on liver, kidney function in CDX model (N=3). Scale bar, 50 μm. (C) Body weight of PP10 and PP24 treated mice during experiment (N = 5). one-way ANOVA test. Exact p- value are presented in Appendix table S3. Data are presented as mean ± SD. *$P < 0.05$, **$P < 0.01$, and ***$P < 0.001$, when compared with control group.

Appendix table S1 *P* value in Figure 1-7.

Figure	Group	p-value	Explaination
Fig. 1B	Tumor vs Normal	0.0006816	
Fig. 1C	Tumor vs Normal	0.00507	
Fig. 1F	Tumor vs Normal	0.0404	liver cancer
	Tumor vs Normal	0.0215	lung cancer
	Tumor vs Normal	0.0107	colorectal cancer
	Tumor vs Normal	0.0021	gastric cancer
Fig. 1H	Tumor vs Normal	0.0000358	
Fig. 3D	Control vs PP10 5 mg/kg	0.00153	
	Control vs PP10 10 mg/kg	0.000247	
	Control vs Oxaliplatin 5 mg/kg	0.000934	
Fig. 3E	Control vs. PP24 2.5 mg/kg	0.000253	
	Control vs. PP24 5 mg/kg	0.000691	
	Control vs. Oxaliplatin 5 mg/kg	0.000447	
Fig. 6G	0 vs. 0.5	0.358	
	0 vs. 1	0.0811	
	0 vs. 2	0.000462	
	0 vs. 4	0.0000655	

Figure	Group	p-value	Explaination
Fig. 7D	Control vs. PP10 5 mg/kg	0.00236	
	Control vs. PP10 10 mg/kg	0.000435	
Fig. 7E	Control vs. PP24 2.5 mg/kg	0.00117	
	Control vs. PP24 5.0 mg/kg	0.000932	
Fig. 7H	Control vs. PP10 2.5 mg/kg	0.0135	
	Control vs. PP24 2.5 mg/kg	0.000368	
	Control vs. PP10 5 mg/kg	0.000261	
	Control vs. PP24 5 mg/kg	0.0000965	
Fig. 7K	Control vs. PP10 2.5 mg/kg	0.0357	
	Control vs. PP24 2.5 mg/kg	0.0464	
	Control vs. PP10 5 mg/kg	0.00844	
	Control vs. PP24 5 mg/kg	0.0000686	

Appendix table S2 *P* value in Figure EV1-7.

Figure	Group	p-value	Explaination
Fig. EV2C	0 vs. 0.25	0.0036	PP10
	0 vs. 0.5	0.000351	
	0 vs. 1	0.000114	
	0 vs. 0.25	0.199	PP24
	0 vs. 0.5	0.0917	
	0 vs. 1	0.00249	
Fig. EV2E	0 vs. 0.75	0.0000582	PP10
	0 vs. 1.5	0.0000698	
	0 vs. 0.75	0.0000969	PP24
	0 vs. 1.5	0.0000573	
Fig. EV2G	0 vs. 0.75	0.0000357	PP10
	0 vs. 1.5	0.0000268	
	0 vs. 0.75	0.0000628	PP24
	0 vs. 1.5	0.0000241	
Fig. EV2I	0 vs. 0.25	0.00168	PP10
	0 vs. 0.5	0.000544	
	0 vs. 1	0.000163	
	0 vs. 0.25	0.0103	PP24
	0 vs. 0.5	0.00115	
	0 vs. 1	0.000436	
Fig. EV3C	0 vs. 0.25	0.00165	PP10
	0 vs. 0.5	0.000912	
	0 vs. 1	0.000538	
	0 vs. 0.25	0.192	PP24
	0 vs. 0.5	0.0441	
	0 vs. 1	0.0302	
Fig. EV3E	0 vs. 0.75	0.000528	PP10
	0 vs. 1.5	0.0000957	
	0 vs. 0.75	0.000935	PP24
	0 vs. 1.5	0.0000852	
Fig. EV3G	0 vs. 0.75	0.000688	PP10
	0 vs. 1.5	0.0000945	
	0 vs. 0.75	0.0201	PP24
	0 vs. 1.5	0.000338	
Fig. EV3I	0 vs. 0.25	0.000665	PP10
	0 vs. 0.5	0.000133	
	0 vs. 1	0.0000681	
	0 vs. 0.25	0.0184	PP24
	0 vs. 0.5	0.00641	
	0 vs. 1	0.00245	
Fig. EV4A	0 vs. 1.5	0.0413	HCT116-mTOR
	0 vs. 3	0.0124	
	0 vs. 1.5	0.0386	HCT116-PI3K
	0 vs. 3	0.00135	
	0 vs. 1.5	0.0537	HCT116-AKT
	0 vs. 3	0.0455	
	0 vs. 1.5	0.0273	DLD1-mTOR
	0 vs. 3	0.000739	
	0 vs. 1.5	0.0599	DLD1-PI3K
	0 vs. 3	0.000966	
	0 vs. 1.5	0.0627	DLD1-AKT
	0 vs. 3	0.0382	
Fig. EV4B	0 vs. 1.5	0.0322	HCT116-P62
	0 vs. 3	0.00164	
	0 vs. CQ	0.0162	
	0 vs. CQ+3	0.00912	
	0 vs. 1.5	0.0000692	HCT116-Bax
	0 vs. 3	0.0000367	
	0 vs. CQ	0.5979	
	0 vs. CQ+3	0.4063	
	0 vs. 1.5	0.00362	HCT116-Bcl2
	0 vs. 3	0.0000923	
	0 vs. CQ	0.209	
	0 vs. CQ+3	0.0337	
	0 vs. 1.5	0.000296	HCT116-LC3B
	0 vs. 3	0.0000568	
	0 vs. CQ	0.0196	
	0 vs. CQ+3	0.0000648	
	0 vs. 1.5	0.00352	DLD1-P62
	0 vs. 3	0.000166	
	0 vs. CQ	0.00423	
	0 vs. CQ+3	0.00119	
	0 vs. 1.5	0.00741	DLD1-Bax
	0 vs. 3	0.00187	
	0 vs. CQ	0.0333	
	0 vs. CQ+3	0.000335	
	0 vs. 1.5	0.0141	DLD1-Bcl2
	0 vs. 3	0.000165	
	0 vs. CQ	0.000625	
	0 vs. CQ+3	0.000443	
	0 vs. 1.5	0.136	DLD1-LC3B
	0 vs. 3	0.000255	
	0 vs. CQ	0.652	
	0 vs. CQ+3	0.000654	

Figure	Group	p-value	Explaination
Fig. EV4D	0 vs. 1.5	0.0688	HCT116-LC3B
	0 vs. 3	0.0189	
	0 vs. Baf-A1	0.00124	
	0 vs. Baf-A1+3	0.000266	
	0 vs. 1.5	0.909	HCT116-P62
	0 vs. 3	0.0955	
	0 vs. Baf-A1	0.00458	
	0 vs. Baf-A1+3	0.00892	
	0 vs. 1.5	0.954	DLD1-LC3B
	0 vs. 3	0.101	
	0 vs. Baf-A1	0.00776	
	0 vs. Baf-A1+3	0.00361	
	0 vs. 1.5	0.9721	DLD1-P62
	0 vs. 3	0.1855	
	0 vs. Baf-A1	0.1158	
	0 vs. Baf-A1+3	0.1049	
Fig. EV4H	0 vs. 1.5	0.0291	HCT116-IL-1β
	0 vs. 3	0.000601	
	0 vs. DSF	0.0909	
	0 vs. DSF+3	0.0252	
	0 vs. 1.5	0.00146	HCT116-GSDMD
	0 vs. 3	0.000112	
	0 vs. DSF	0.103	
	0 vs. DSF+3	0.000612	
	0 vs. 1.5	0.0331	HCT116-Caspase1
	0 vs. 3	0.0032	
	0 vs. DSF	0.754	
	0 vs. DSF+3	0.496	
	0 vs. 1.5	0.8504	DLD1-IL-1β
	0 vs. 3	0.0126	
	0 vs. DSF	0.9989	
	0 vs. DSF+3	0.0065	
	0 vs. 1.5	0.1488	DLD1-GSDMD
	0 vs. 3	0.0136	
	0 vs. DSF	0.9277	
	0 vs. DSF+3	0.0378	
	0 vs. 1.5	0.9491	DLD1-Caspase1
	0 vs. 3	0.0069	
	0 vs. DSF	0.5351	
	0 vs. DSF+3	0.0556	
Fig. EV5A	#1 vs. #2	0.0000254	β-catenin
	#1 vs. #3	0.0000514	
	#1 vs. #4	0.573	
	#1 vs. #5	0.000185	
	#1 vs. #2	0.00257	Dvl2
	#1 vs. #3	0.0000355	
	#1 vs. #4	0.0269	
	#1 vs. #5	0.000782	
	#1 vs. #2	0.0722	Naked1
	#1 vs. #3	0.00856	
	#1 vs. #4	0.0584	
	#1 vs. #5	0.00858	
	#1 vs. #2	0.4243	Akt
	#1 vs. #3	0.0207	
	#1 vs. #4	0.00055	
	#1 vs. #5	0.2373	
	#1 vs. #2	0.00334	GSK3β
	#1 vs. #3	0.00015	
	#1 vs. #4	0.0552	
	#1 vs. #5	0.00695	
	#1 vs. #2	0.000456	LEF1
	#1 vs. #3	0.000468	
	#1 vs. #4	0.0244	
	#1 vs. #5	0.0114	
	#1 vs. #2	0.000254	TCF
	#1 vs. #3	0.000186	
	#1 vs. #4	0.658	
	#1 vs. #5	0.215	
Fig. EV5E	0 vs. 0.5	0.00257	
	0 vs. 1	0.00156	
	0 vs. 2	0.000988	
	0 vs. 4	0.000667	
Fig. EV5F	0 vs. 1.5	0.00145	Bax
	0 vs. 3	0.0000655	
	0 vs. 1.5	0.000369	Bcl2
	0 vs. 3	0.000267	

Appendix table S3 *P* value in AF S1-6.

Figure	Group	p-value	Explaination
AS F1C	PP10 vs Control	0.0196	45 °C
	PP10 vs Control	0.0388	50 °C
	PP10 vs Control	0.00162	55 °C
	PP10 vs Control	0.074855	60 °C
	PP24 vs Control	0.0113	45 °C
	PP24 vs Control	0.0375	50 °C
	PP24 vs Control	0.183	55 °C
	PP24 vs Control	0.00405	60 °C
AS F1D	PP10 vs Control	0.00675	45 °C
	PP10 vs Control	0.0142	50 °C
	PP10 vs Control	0.129	55 °C
	PP10 vs Control	0.407801	60 °C
	PP24 vs Control	0.020551	45 °C
	PP24 vs Control	0.040345	50 °C
	PP24 vs Control	0.180248	55 °C
	PP24 vs Control	0.210649	60 °C
AS F2A	0 vs. 1.5	0.00259	N-cadherin
	0 vs. 3	0.000733	
	0 vs. 1.5	0.1258	E-cadherin
	0 vs. 3	0.0081	
	0 vs. 1.5	0.0105	Snail
	0 vs. 3	0.0038	
	0 vs. 1.5	0.0524	Vimentin
	0 vs. 3	0.0109	
AS F2B	0 vs. 1.5	0.00134	N-cadherin
	0 vs. 3	0.0000254	
	0 vs. 1.5	0.0692	E-cadherin
	0 vs. 3	0.0367	
	0 vs. 1.5	0.0035	Snail
	0 vs. 3	0.000268	
	0 vs. 1.5	0.000378	Vimentin
	0 vs. 3	0.000787	
AS F2C	0 vs. 1.5	0.0019	N-cadherin
	0 vs. 3	0.0003	
	0 vs. 1.5	0.0547	E-cadherin
	0 vs. 3	0.0067	
	0 vs. 1.5	0.0296	Snail
	0 vs. 3	0.0076	
	0 vs. 1.5	0.0051	Vimentin
	0 vs. 3	0.0025	
AS F2D	A vs. B	0.000554	
	A vs. C	0.000315	
	A vs. D	0.000477	
AS F2E	A vs. B	0.000338	
	A vs. C	0.000265	
	A vs. D	0.0588	
AS F3C	A vs. B	0.9887	AKP
	A vs. C	0.9941	
	A vs. D	0.8804	
	A vs. B	0.2014	ALT
	A vs. C	0.8446	
	A vs. D	0.284	
	A vs. B	0.7731	AST
	A vs. C	0.898	
	A vs. D	0.7847	
	A vs. B	0.8336	BUN
	A vs. C	0.3913	
	A vs. D	0.8262	
	A vs. B	0.9881	CREA
	A vs. C	0.2529	
	A vs. D	0.9999	
	A vs. B	0.9577	LDH
	A vs. C	0.3217	
	A vs. D	0.8856	
AS F3D	A vs. B	0.9684	AKP
	A vs. C	0.9684	
	A vs. D	0.2464	
	A vs. B	0.9308	ALT
	A vs. C	0.8589	
	A vs. D	0.5709	
	A vs. B	0.8738	AST
	A vs. C	0.7087	
	A vs. D	0.8309	
	A vs. B	>0.9999	BUN
	A vs. C	0.9026	
	A vs. D	0.2229	
	A vs. B	0.8469	CREA
	A vs. C	0.9965	
	A vs. D	0.6481	
	A vs. B	0.7106	LDH
	A vs. C	0.6044	
	A vs. D	0.7793	

Figure	Group	p-value	Explaination
AS F4C	A vs. B	0.8084	AKP
	A vs. C	0.9889	
	A vs. B	0.9348	ALT
	A vs. C	0.8162	
	A vs. B	0.7102	AST
	A vs. C	0.9947	
	A vs. B	0.8667	BUN
	A vs. C	0.9825	
	A vs. B	0.9988	CREA
	A vs. C	0.8539	
	A vs. B	0.3691	LDH
	A vs. C	0.2835	
AS F4D	A vs. B	0.5334	AKP
	A vs. C	0.2259	
	A vs. B	0.8917	ALT
	A vs. C	0.8796	
	A vs. B	0.692	AST
	A vs. C	0.9873	
	A vs. B	0.5615	BUN
	A vs. C	0.8871	
	A vs. B	0.4733	CREA
	A vs. C	0.8456	
	A vs. B	0.2586	LDH
	A vs. C	0.7097	
AS F5B	A vs. B	0.7079	AKP
	A vs. C	0.9778	
	A vs. D	0.8555	
	A vs. E	0.9418	
	A vs. B	>0.9999	ALT
	A vs. C	0.9989	
	A vs. D	0.5238	
	A vs. E	0.3104	
	A vs. B	0.9481	AST
	A vs. C	0.9817	
	A vs. D	0.7461	
	A vs. E	0.0801	
	A vs. B	0.9933	BUN
	A vs. C	0.9933	
	A vs. D	0.5269	
	A vs. E	0.4831	
	A vs. B	0.7628	CREA
	A vs. C	0.9602	
	A vs. D	0.8147	
	A vs. E	0.3916	
	A vs. B	0.2574	LDH
	A vs. C	0.7159	
	A vs. D	0.2184	
	A vs. E	0.444	
AS F6B	A vs. B	0.0724	AKP
	A vs. C	0.9481	
	A vs. D	0.8218	
	A vs. E	>0.9999	
	A vs. B	0.985	ALT
	A vs. C	0.7416	
	A vs. D	0.966	
	A vs. E	0.852	
	A vs. B	0.9993	AST
	A vs. C	0.8639	
	A vs. D	0.9856	
	A vs. E	0.7823	
	A vs. B	0.848	BUN
	A vs. C	0.9697	
	A vs. D	0.5429	
	A vs. E	0.772	
	A vs. B	0.6982	CREA
	A vs. C	0.5296	
	A vs. D	0.8847	
	A vs. E	0.3905	
	A vs. B	0.5236	LDH
	A vs. C	0.304	
	A vs. D	0.2301	
	A vs. E	0.1477	
