# Supplementary figures and images for: AI-identified CD133-targeting natural compounds demonstrate differential anti-tumor effects and mechanisms in pan-cancer models (part 1 of 4)
Source: EMBO Mol Med. 2025 Oct 2;17(11):2932–65. doi: 10.1038/s44321-025-00308-1 (PMC12603267; doi:10.1038/s44321-025-00308-1)

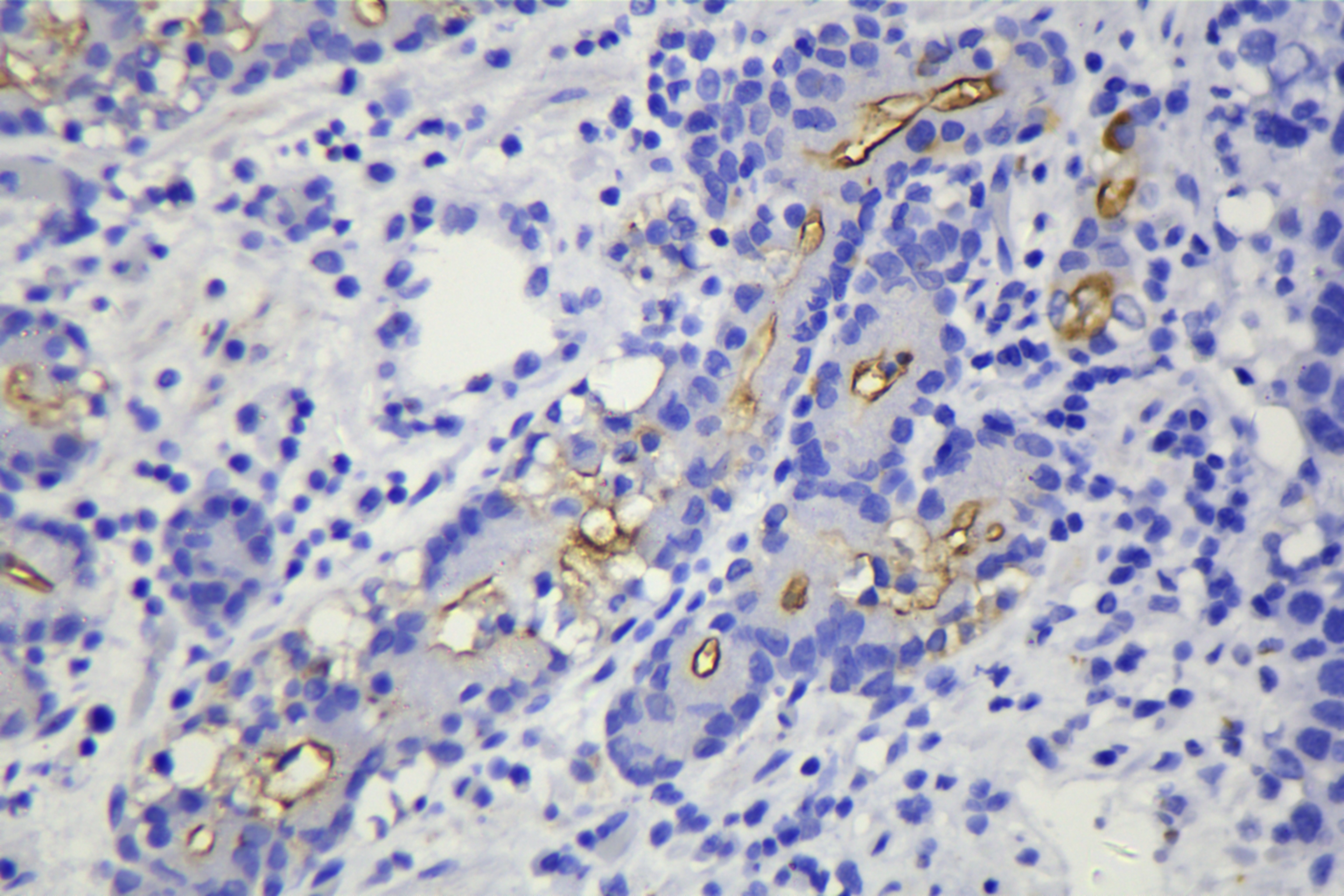

Supplement: Supplementary file 3 — Source data Fig. 1 [file 44321_2025_308_MOESM3_ESM.zip › Figure 1/1f/H240418GC 40X1.jpg]

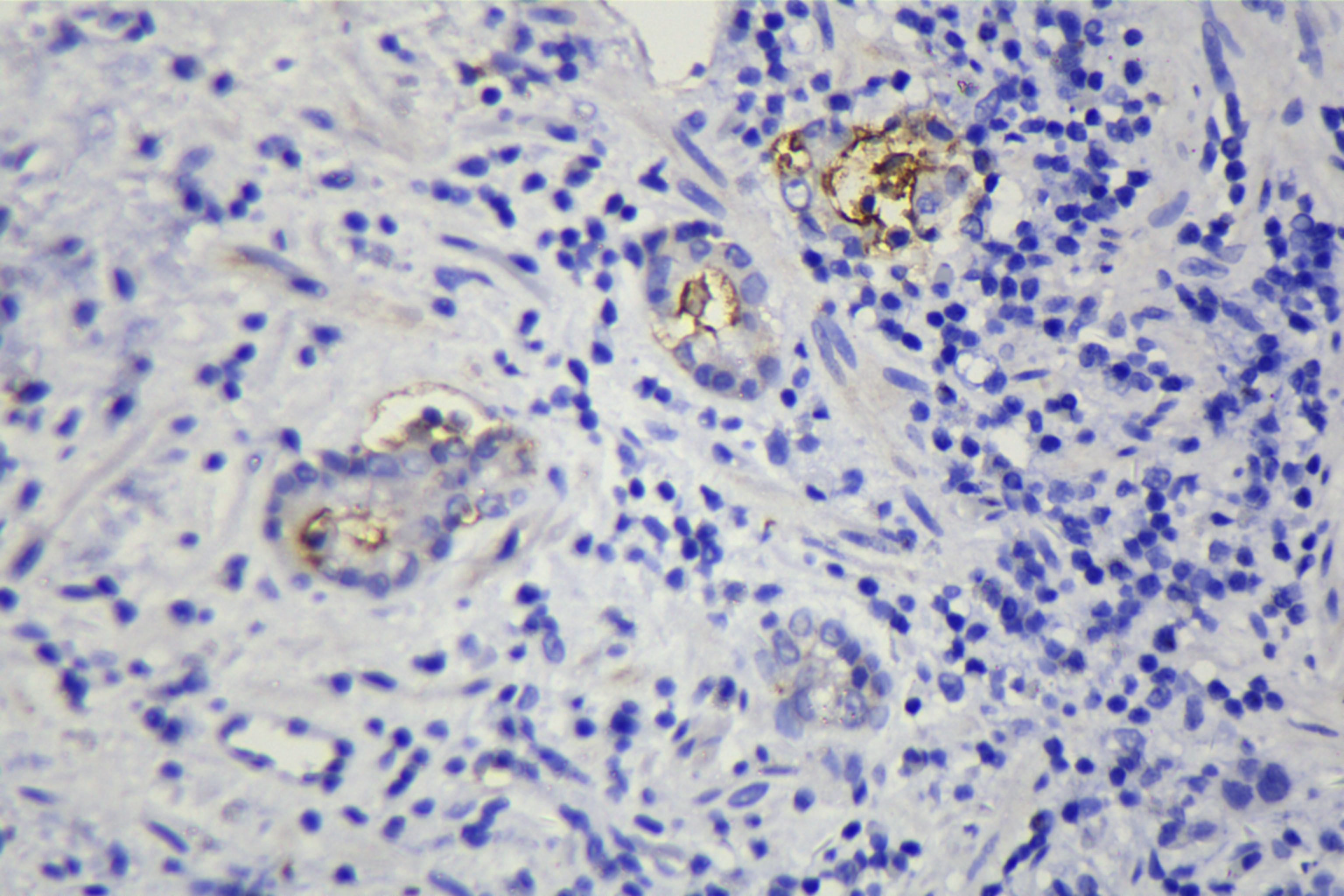

Supplement: Supplementary file 3 — Source data Fig. 1 [file 44321_2025_308_MOESM3_ESM.zip › Figure 1/1f/H240418GC 40X2.jpg]

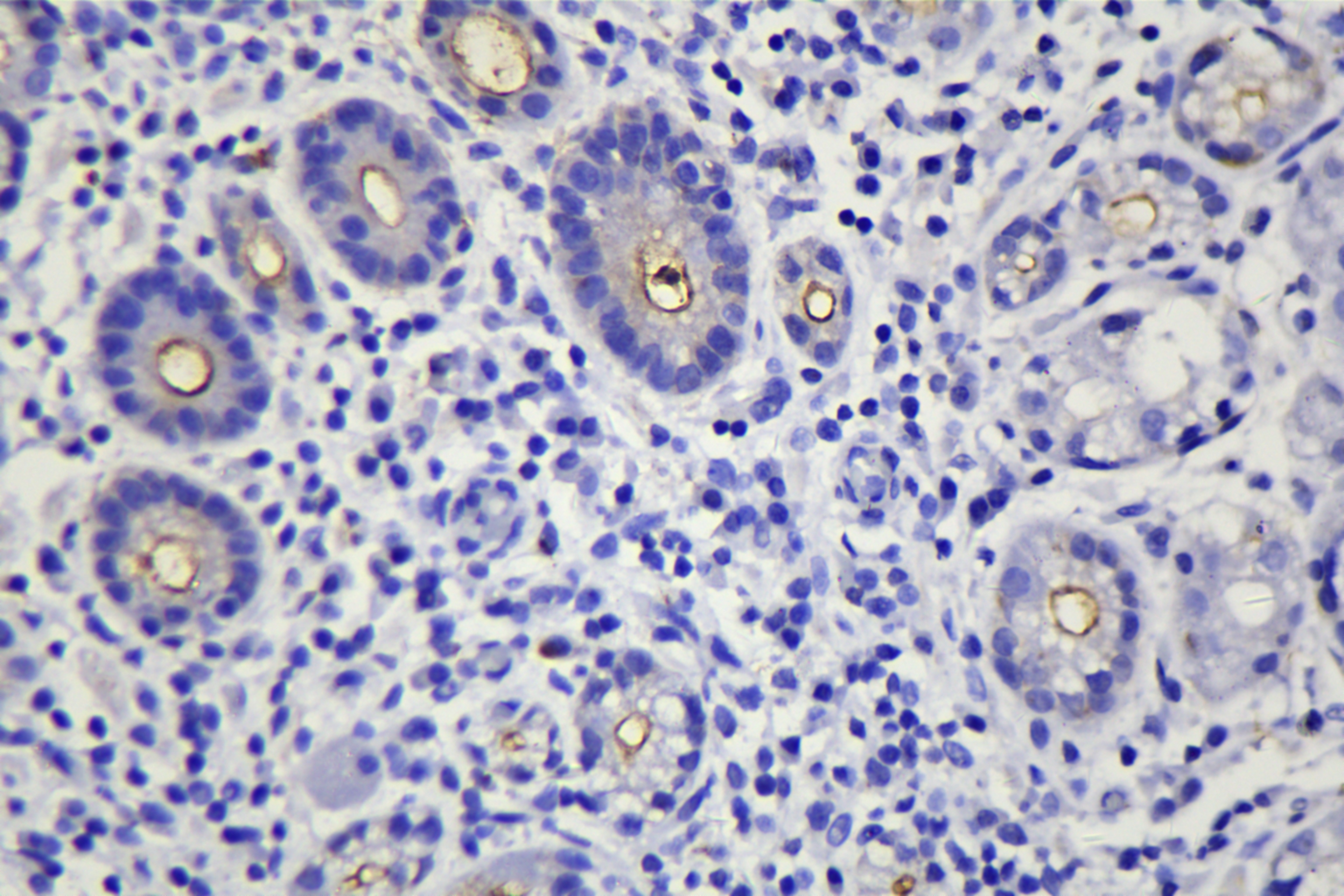

Supplement: Supplementary file 3 — Source data Fig. 1 [file 44321_2025_308_MOESM3_ESM.zip › Figure 1/1f/H240418GC 40X3.jpg]

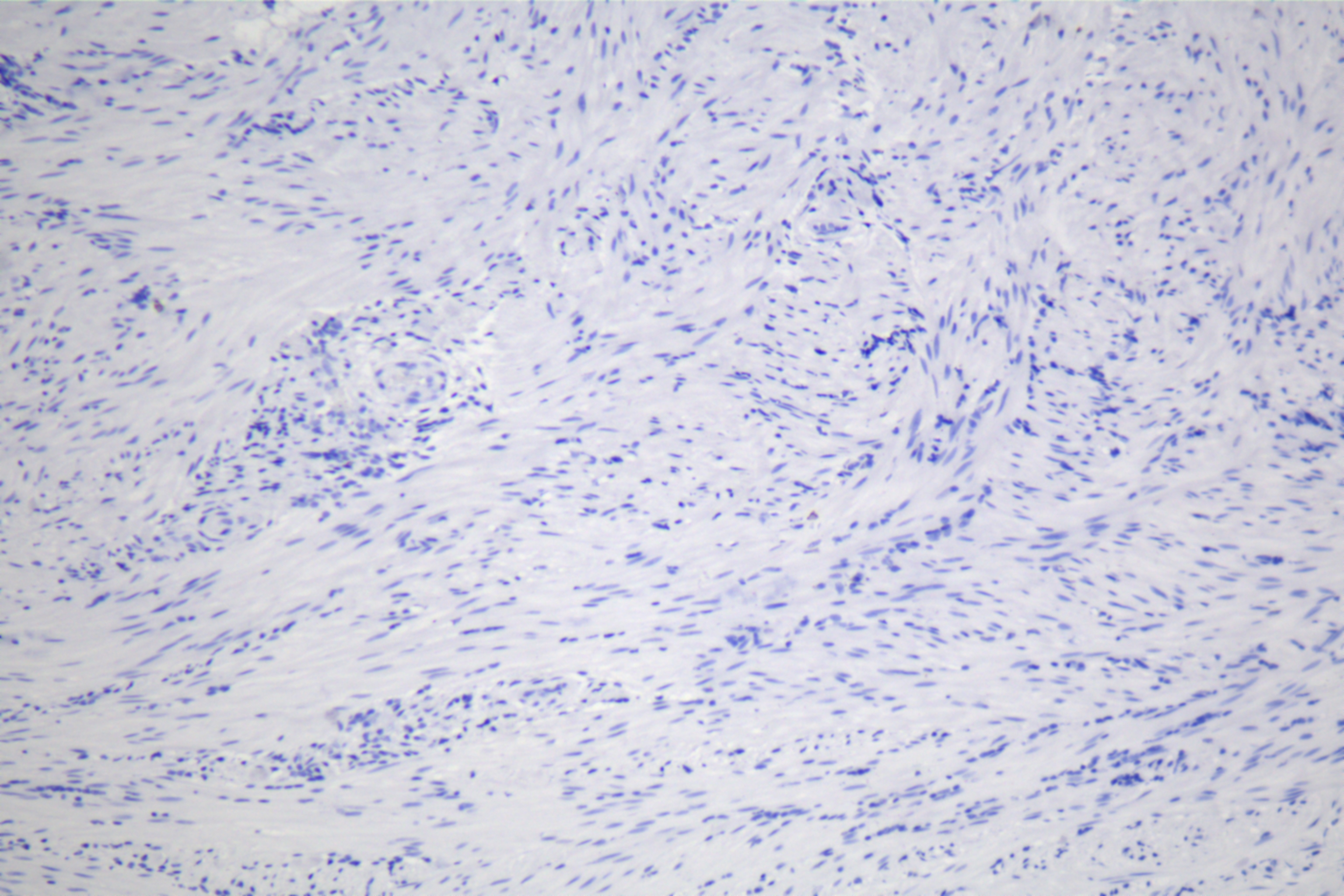

Supplement: Supplementary file 3 — Source data Fig. 1 [file 44321_2025_308_MOESM3_ESM.zip › Figure 1/1f/H240418GN 40X1.jpg]

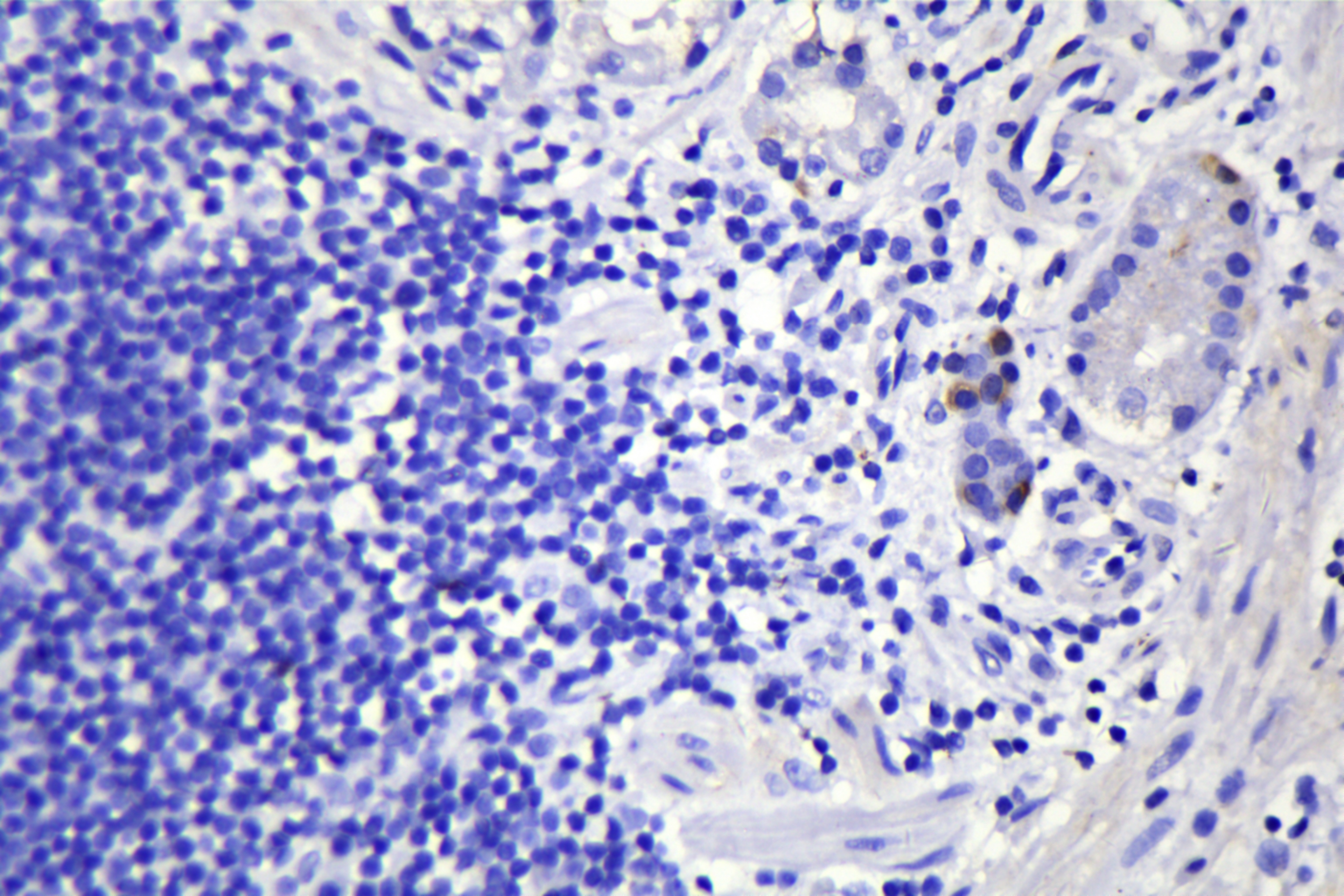

Supplement: Supplementary file 3 — Source data Fig. 1 [file 44321_2025_308_MOESM3_ESM.zip › Figure 1/1f/H240418GN 40X2.jpg]

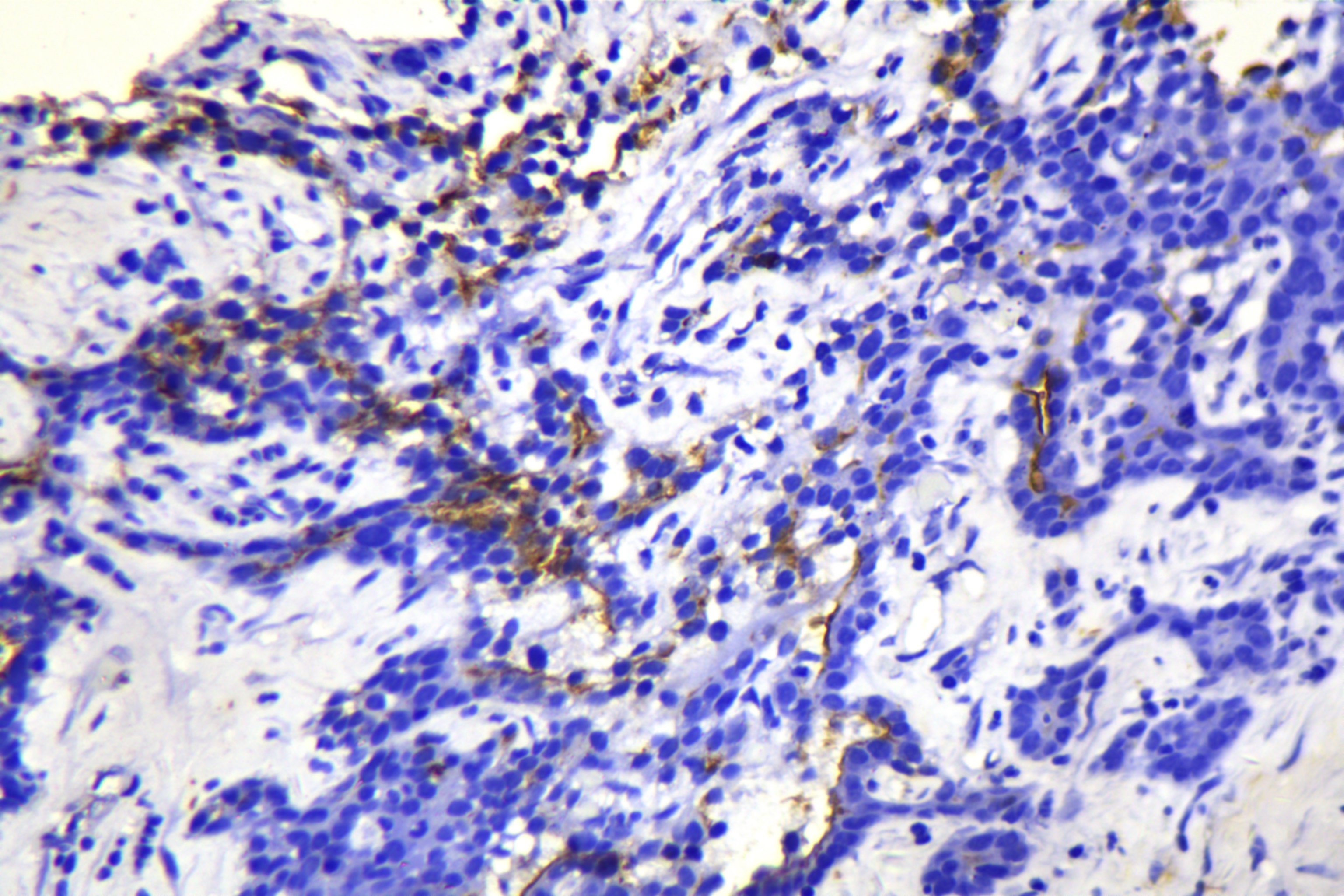

Supplement: Supplementary file 3 — Source data Fig. 1 [file 44321_2025_308_MOESM3_ESM.zip › Figure 1/1f/H240418HC-2 40X1.jpg]

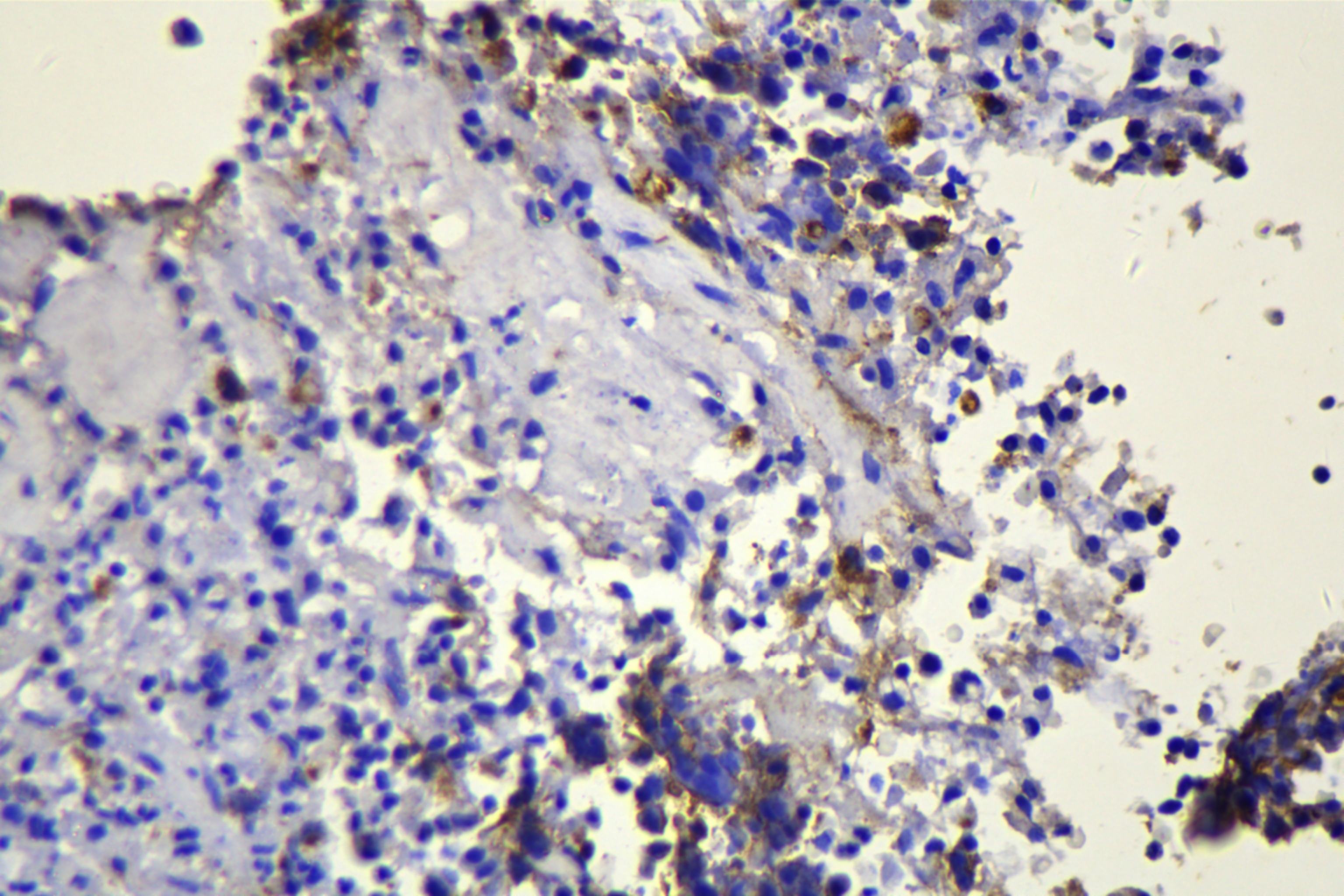

Supplement: Supplementary file 3 — Source data Fig. 1 [file 44321_2025_308_MOESM3_ESM.zip › Figure 1/1f/H240418HC-2 40X2.jpg]

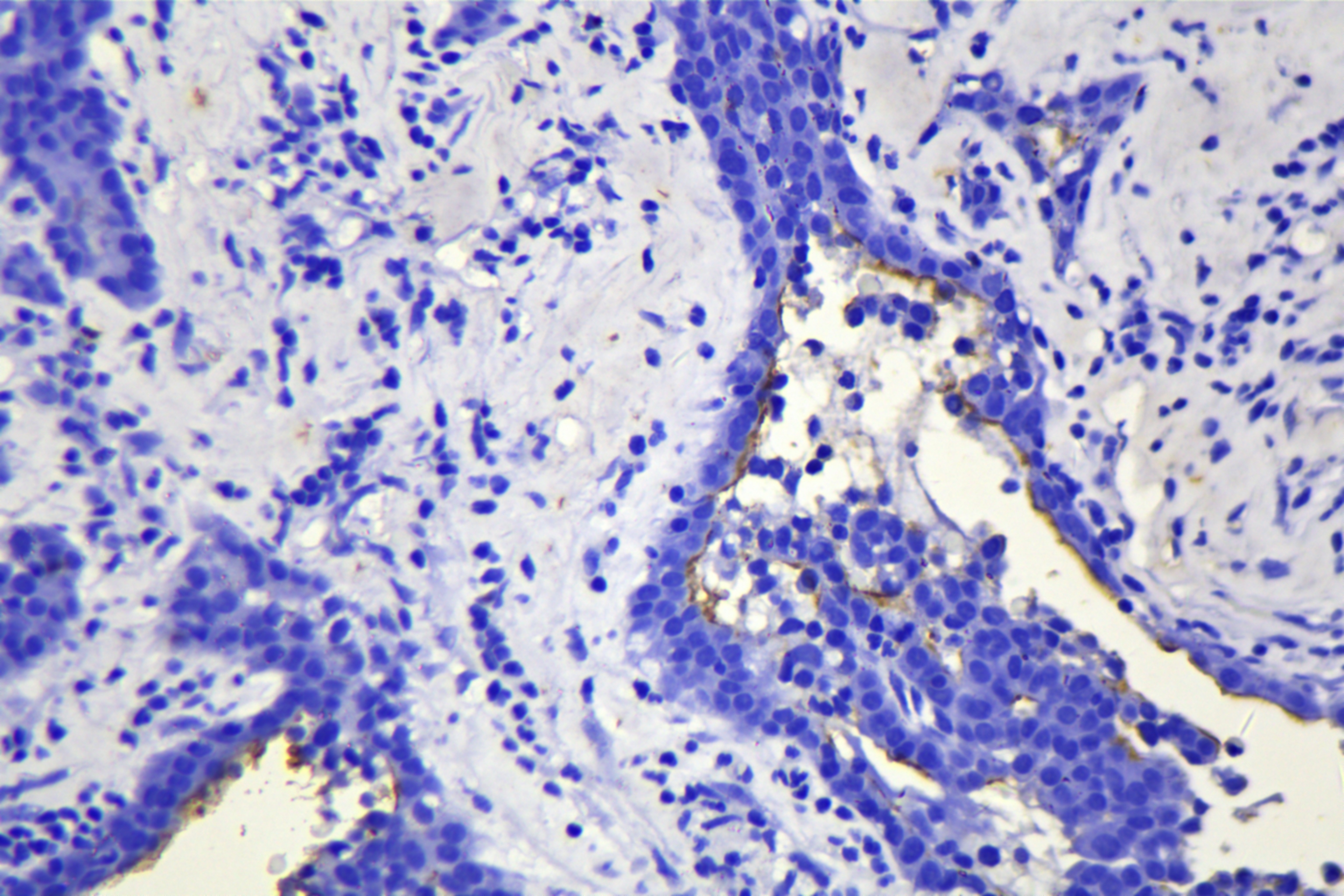

Supplement: Supplementary file 3 — Source data Fig. 1 [file 44321_2025_308_MOESM3_ESM.zip › Figure 1/1f/H240418HC-2 40X3.jpg]

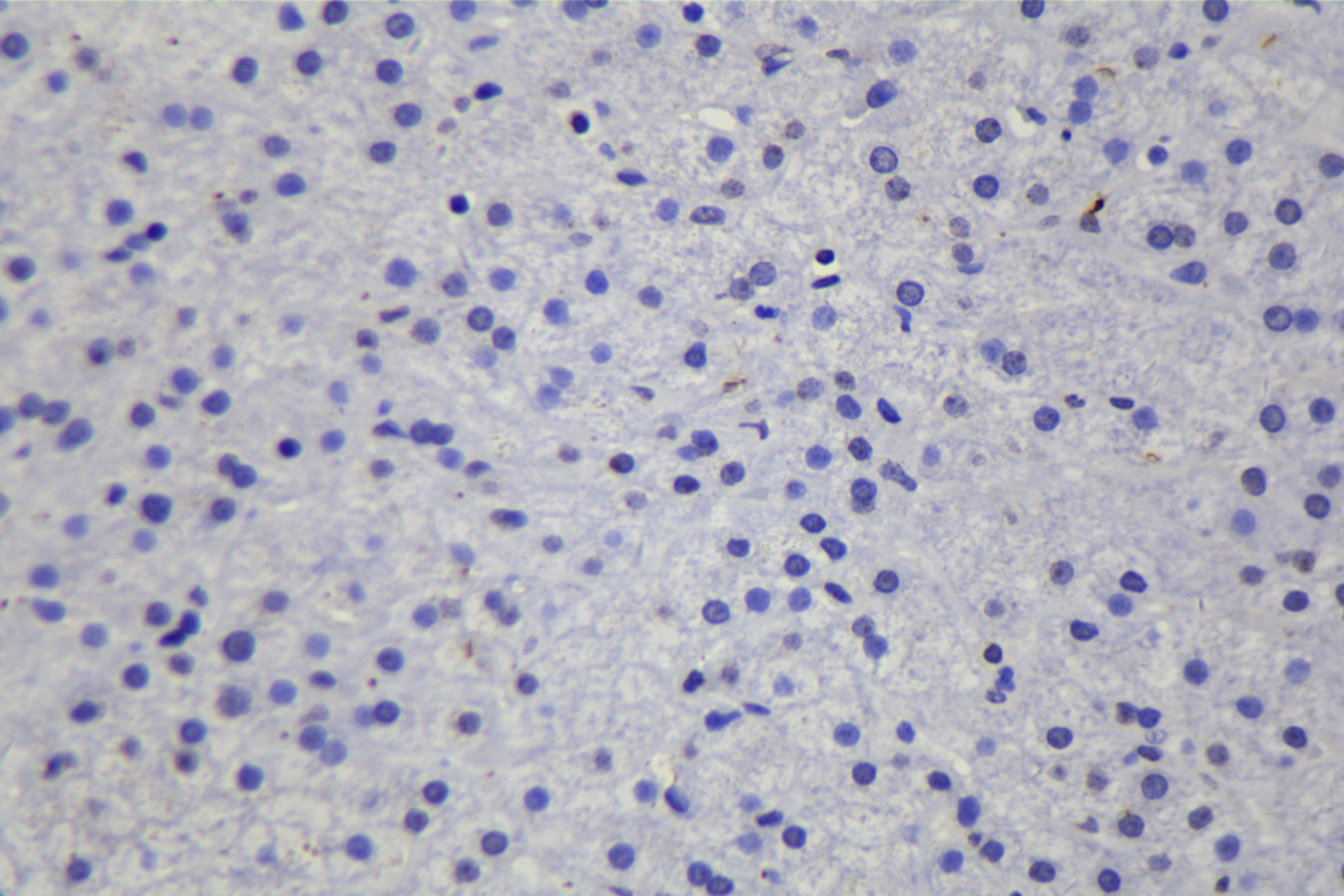

Supplement: Supplementary file 3 — Source data Fig. 1 [file 44321_2025_308_MOESM3_ESM.zip › Figure 1/1f/H240418HN-2 40X1.jpg]

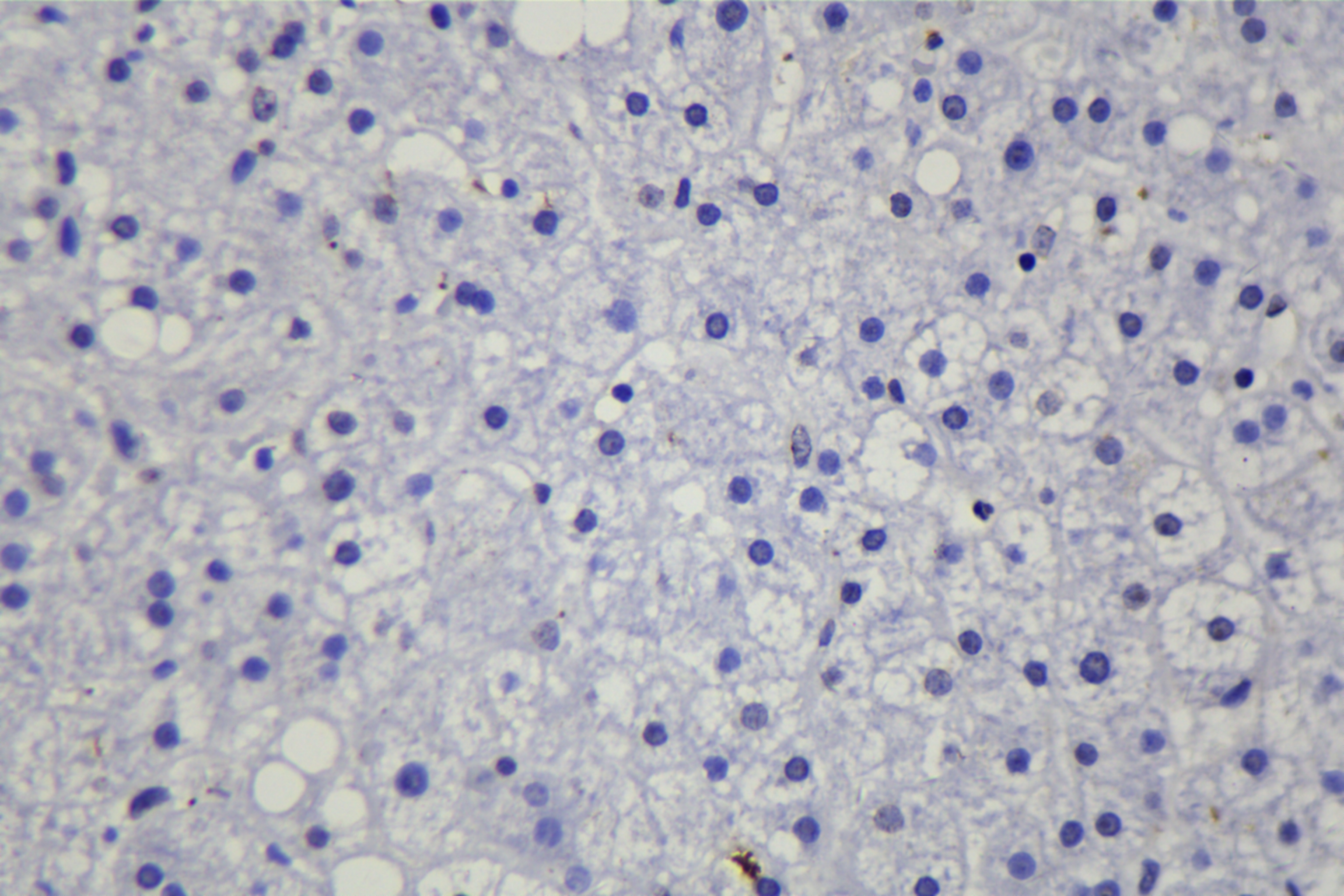

Supplement: Supplementary file 3 — Source data Fig. 1 [file 44321_2025_308_MOESM3_ESM.zip › Figure 1/1f/H240418HN-2 40X2.jpg]

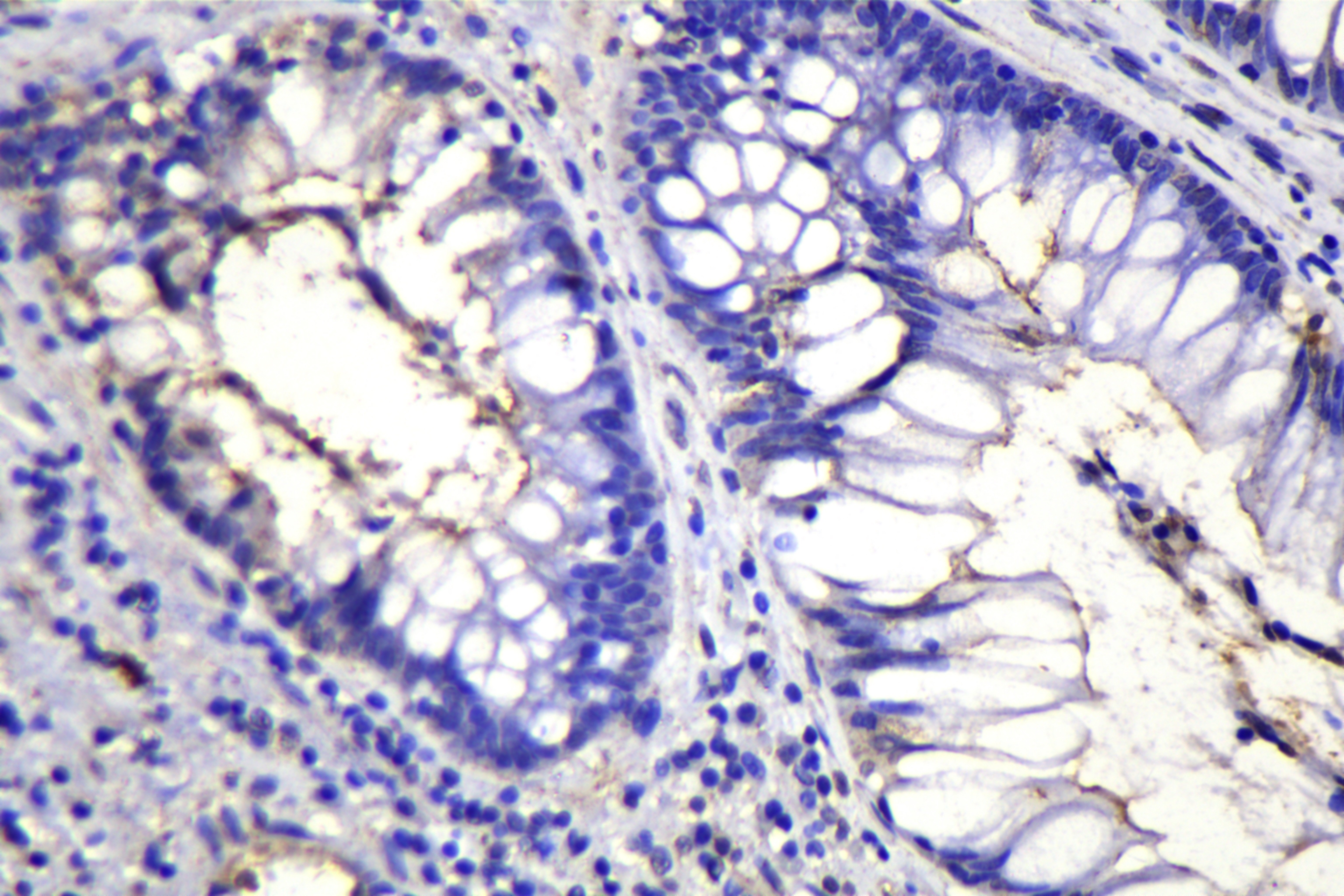

Supplement: Supplementary file 3 — Source data Fig. 1 [file 44321_2025_308_MOESM3_ESM.zip › Figure 1/1f/H240419RC 40X1.jpg]

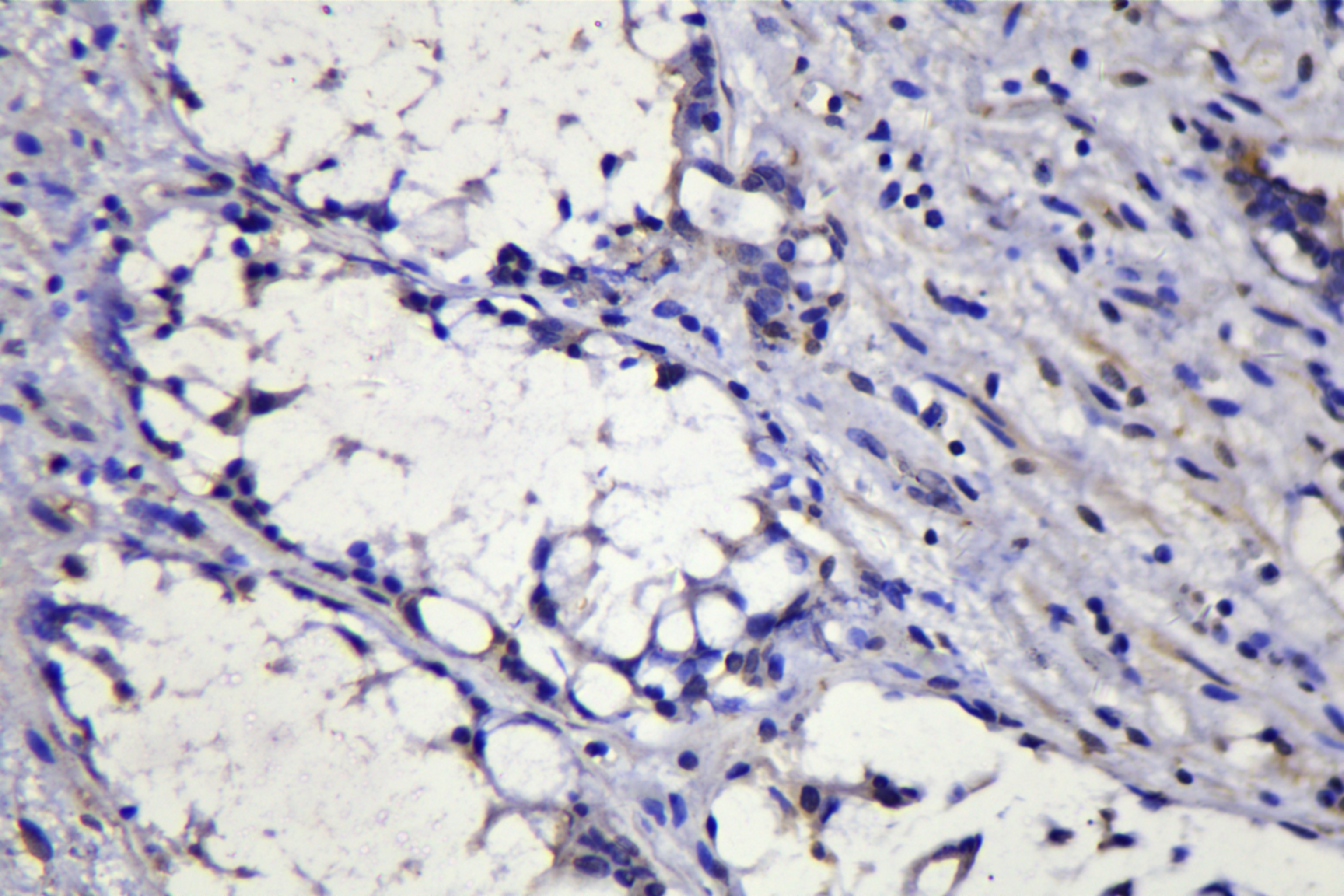

Supplement: Supplementary file 3 — Source data Fig. 1 [file 44321_2025_308_MOESM3_ESM.zip › Figure 1/1f/H240419RC 40X2.jpg]

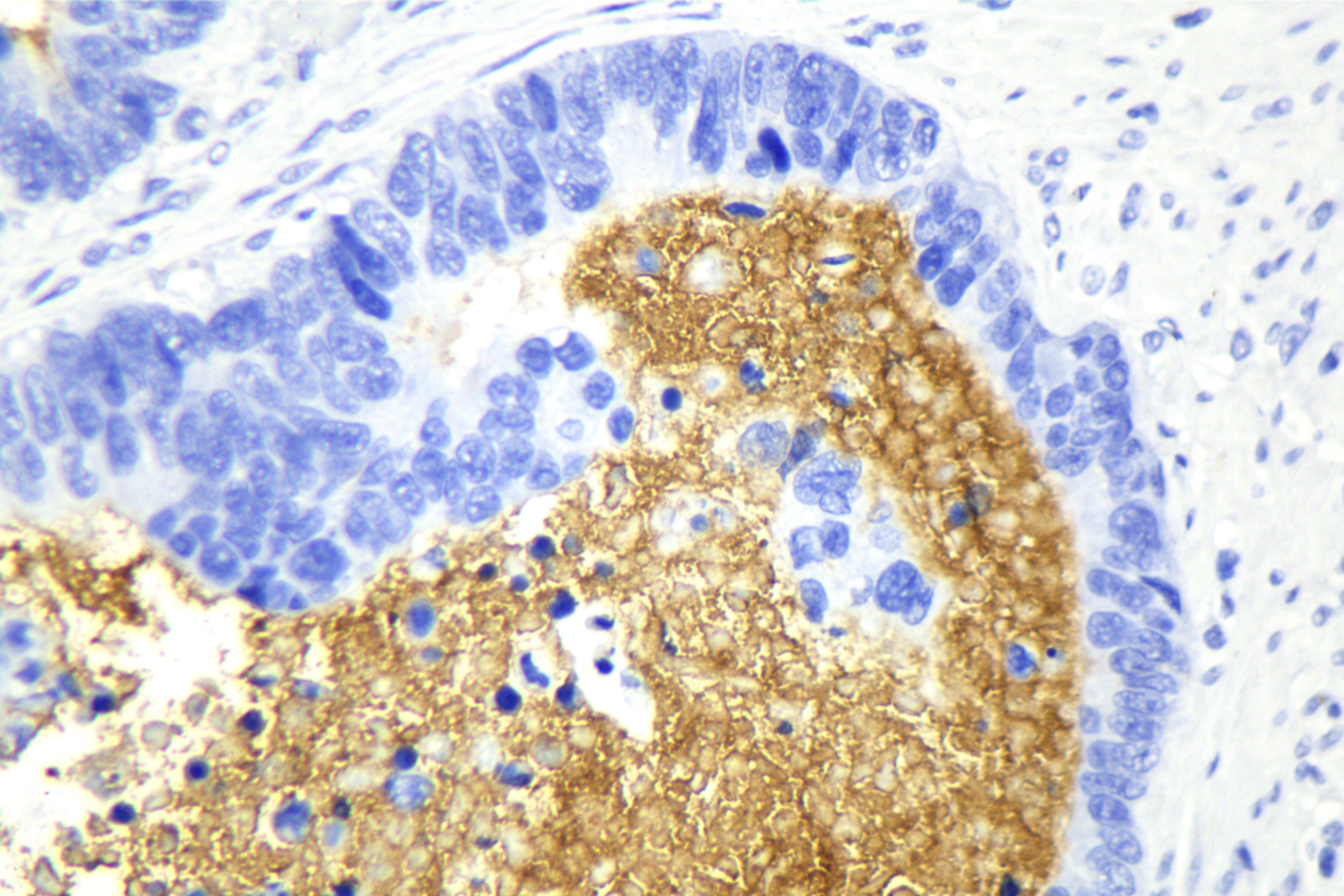

Supplement: Supplementary file 3 — Source data Fig. 1 [file 44321_2025_308_MOESM3_ESM.zip › Figure 1/1f/H240419RC 40X3.jpg]

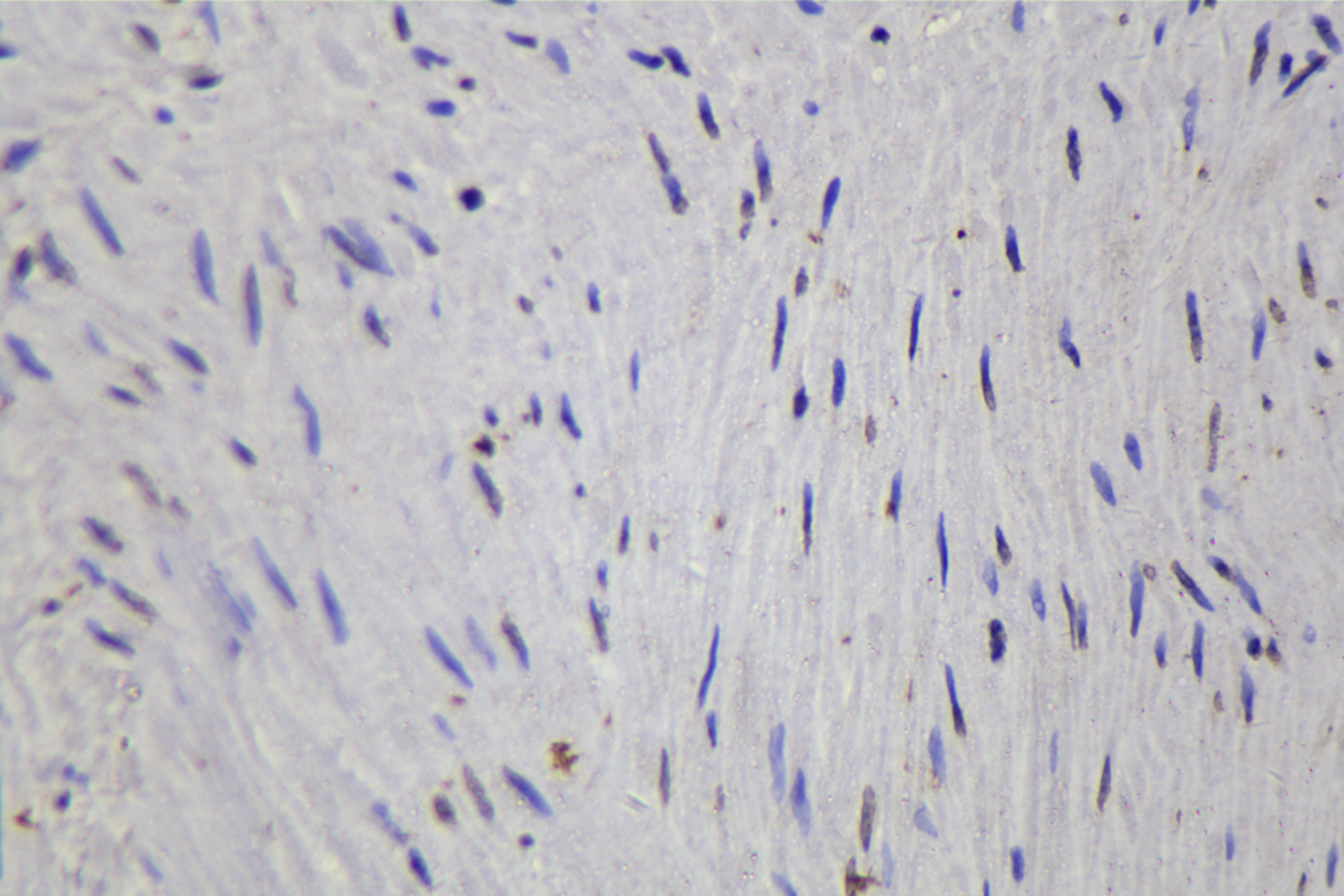

Supplement: Supplementary file 3 — Source data Fig. 1 [file 44321_2025_308_MOESM3_ESM.zip › Figure 1/1f/H240419RN 40X1.jpg]

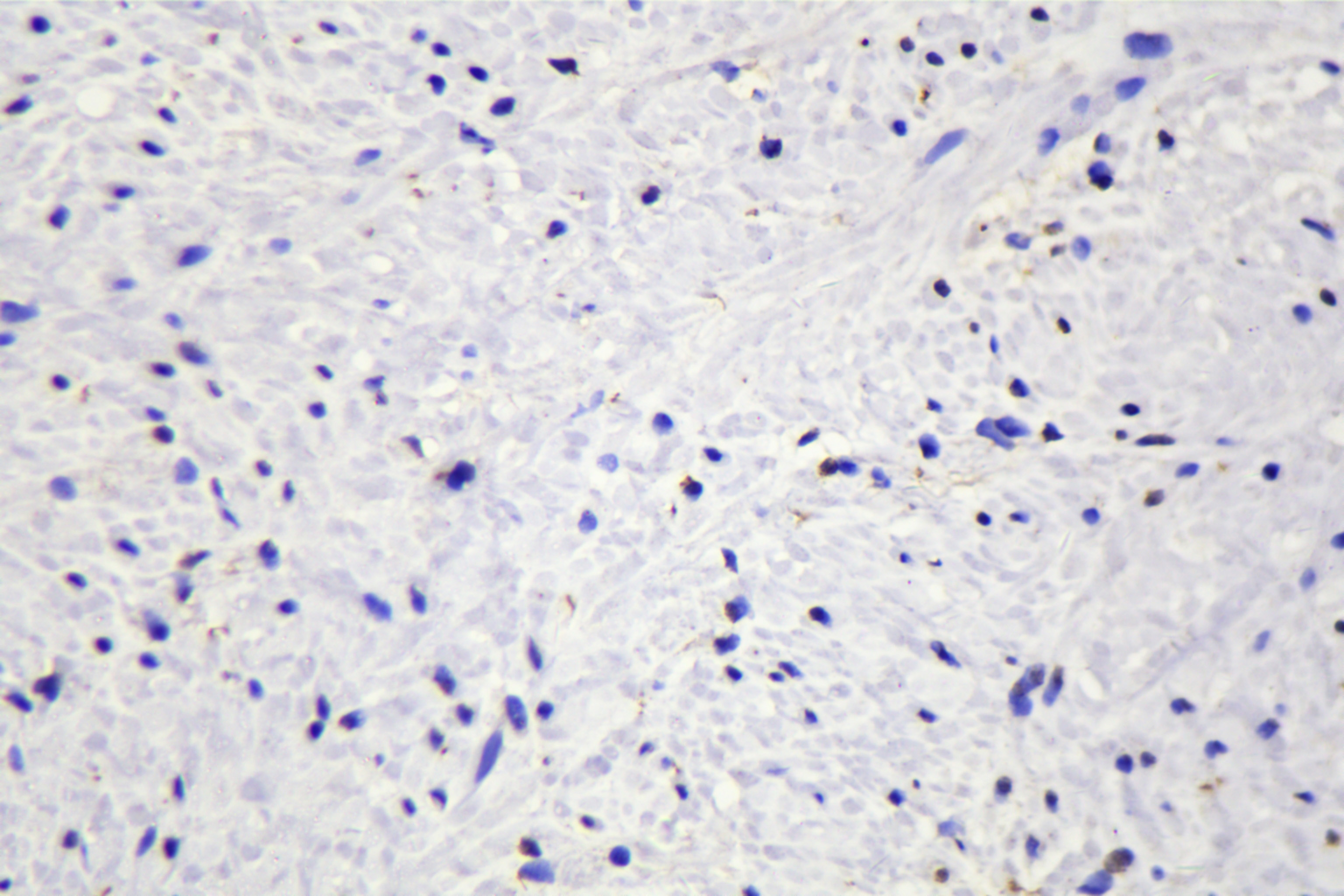

Supplement: Supplementary file 3 — Source data Fig. 1 [file 44321_2025_308_MOESM3_ESM.zip › Figure 1/1f/H240419RN 40X2(2).jpg]

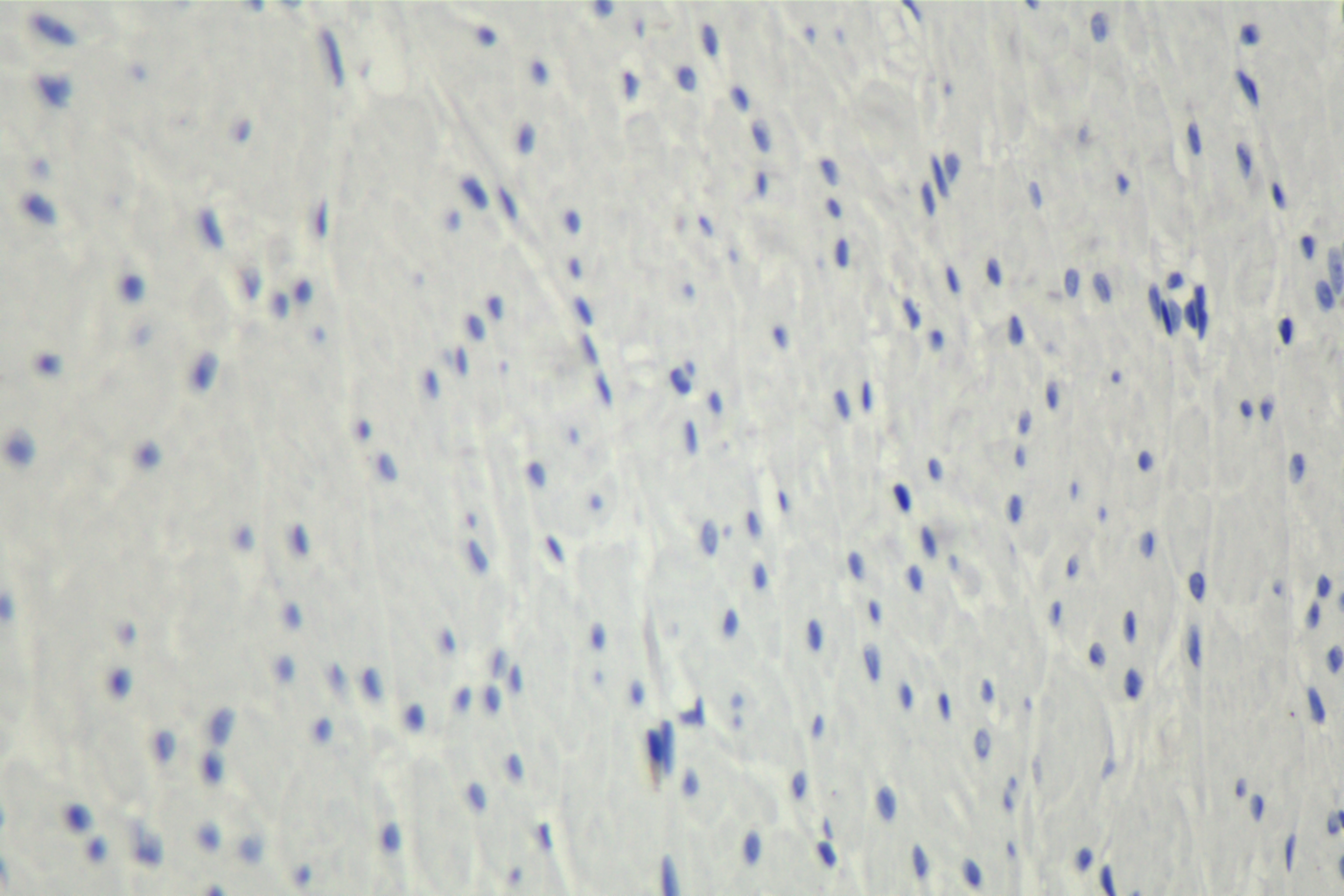

Supplement: Supplementary file 3 — Source data Fig. 1 [file 44321_2025_308_MOESM3_ESM.zip › Figure 1/1f/H240419RN 40X3.jpg]

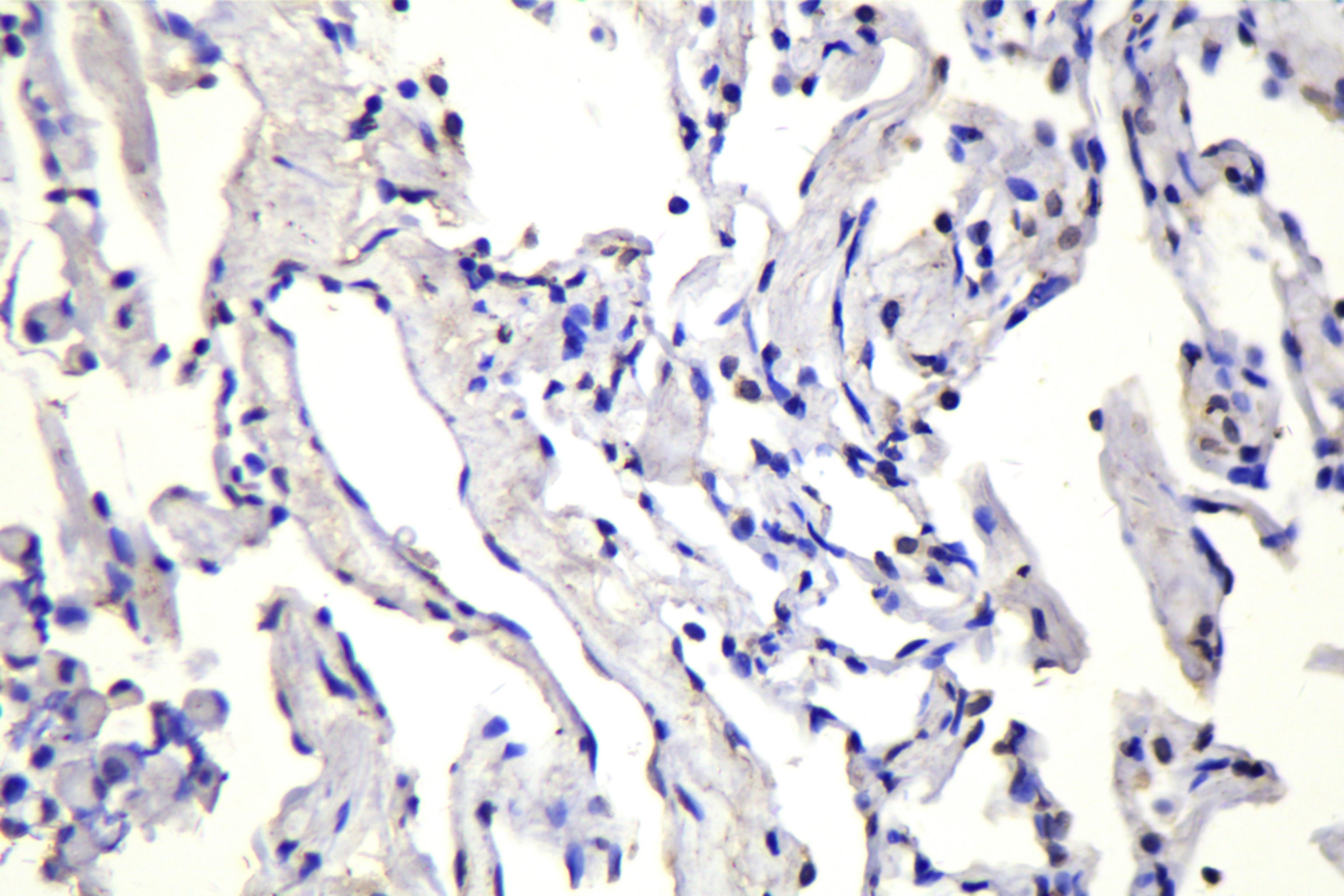

Supplement: Supplementary file 3 — Source data Fig. 1 [file 44321_2025_308_MOESM3_ESM.zip › Figure 1/1f/H240523LC 40X1.jpg]

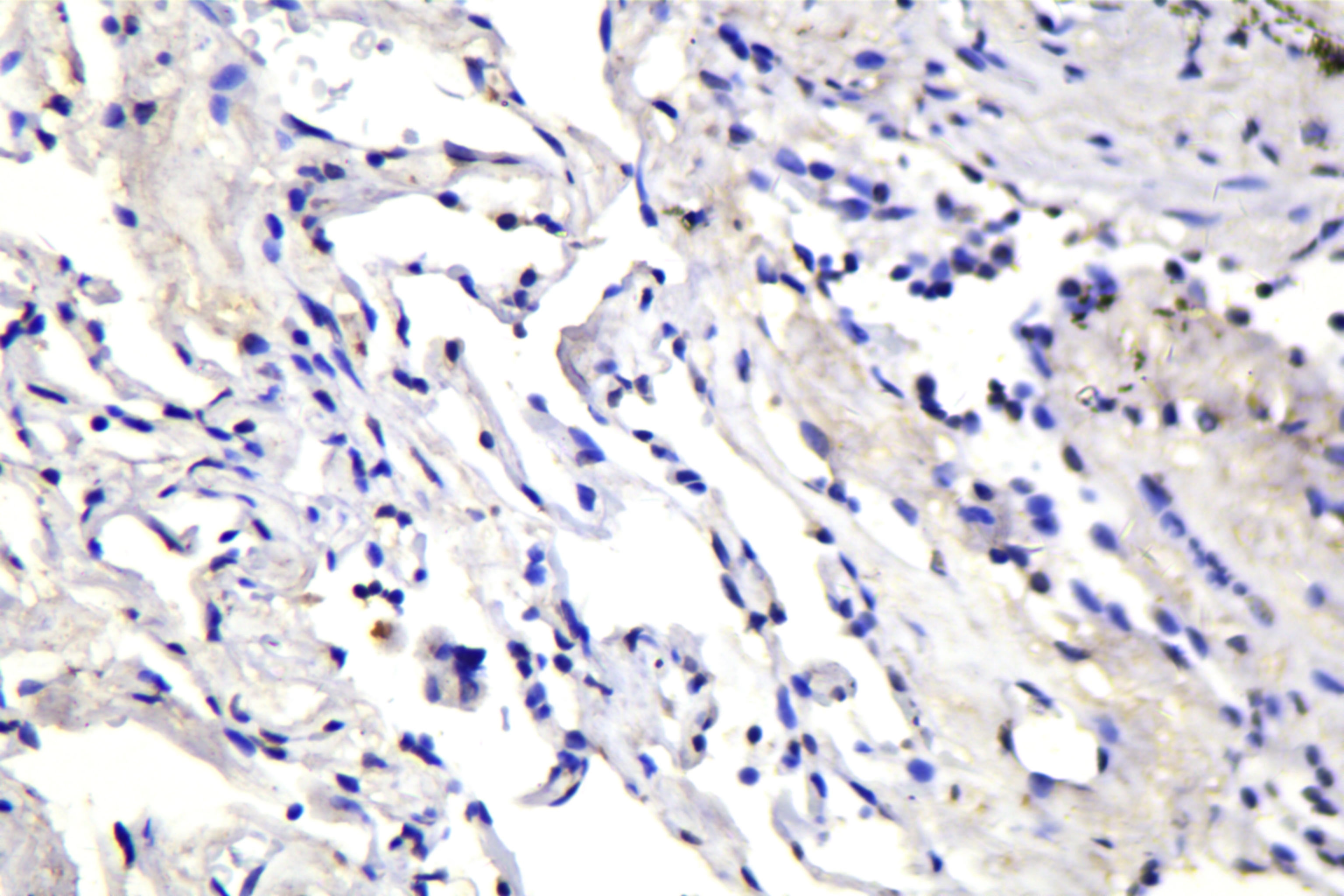

Supplement: Supplementary file 3 — Source data Fig. 1 [file 44321_2025_308_MOESM3_ESM.zip › Figure 1/1f/H240523LC 40X2.jpg]

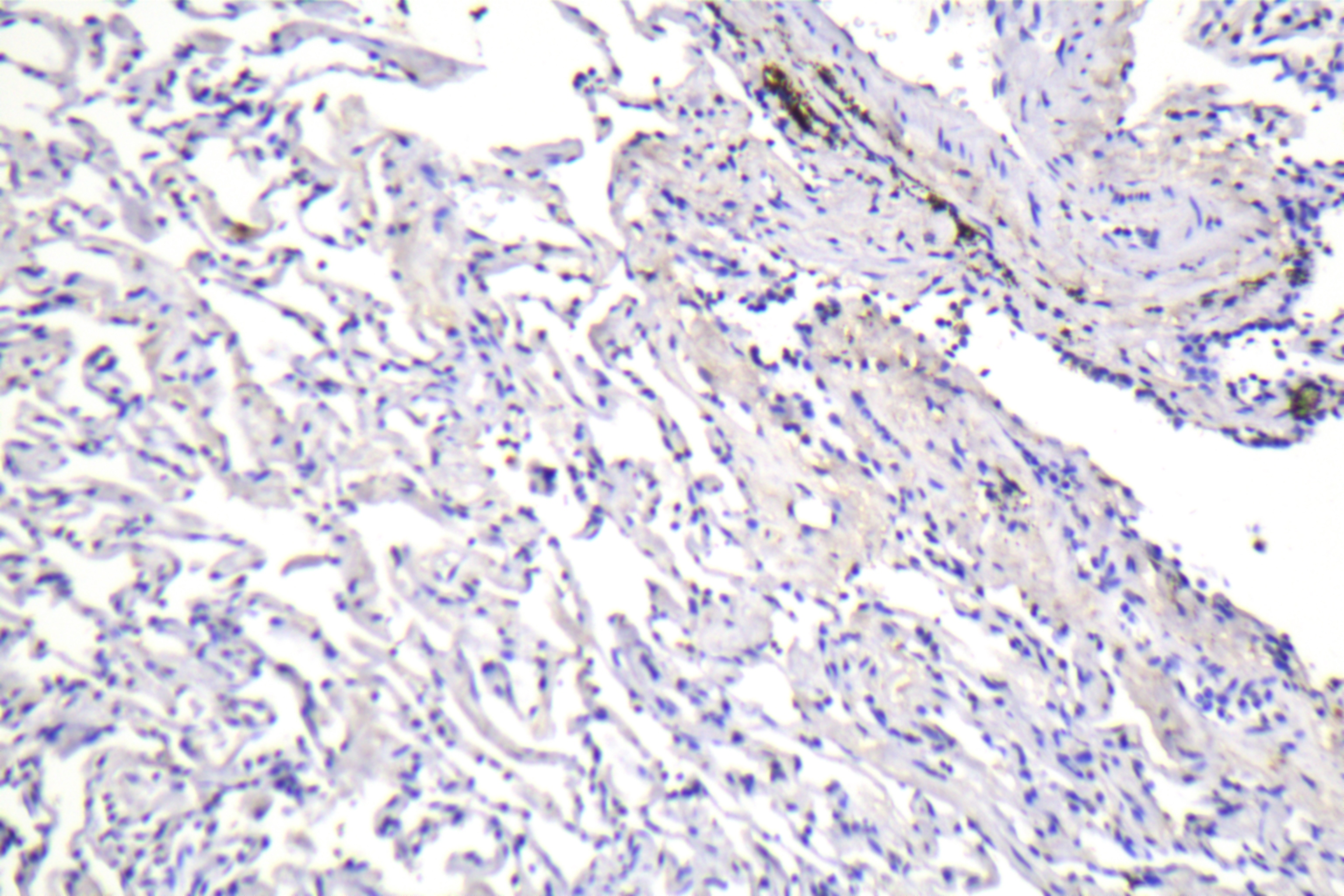

Supplement: Supplementary file 3 — Source data Fig. 1 [file 44321_2025_308_MOESM3_ESM.zip › Figure 1/1f/H240523LC 40X3.jpg]

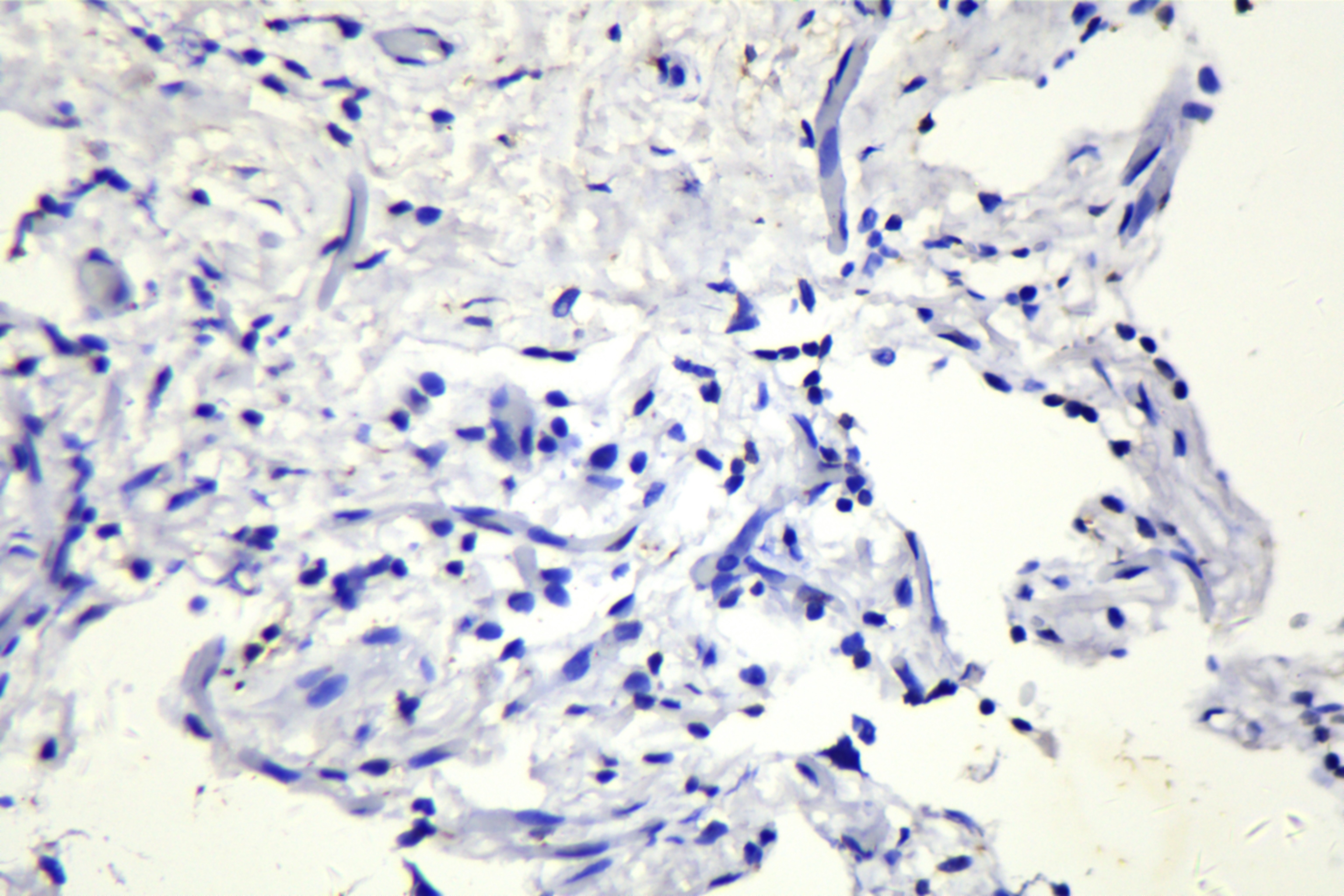

Supplement: Supplementary file 3 — Source data Fig. 1 [file 44321_2025_308_MOESM3_ESM.zip › Figure 1/1f/H240523LN 40X1.jpg]

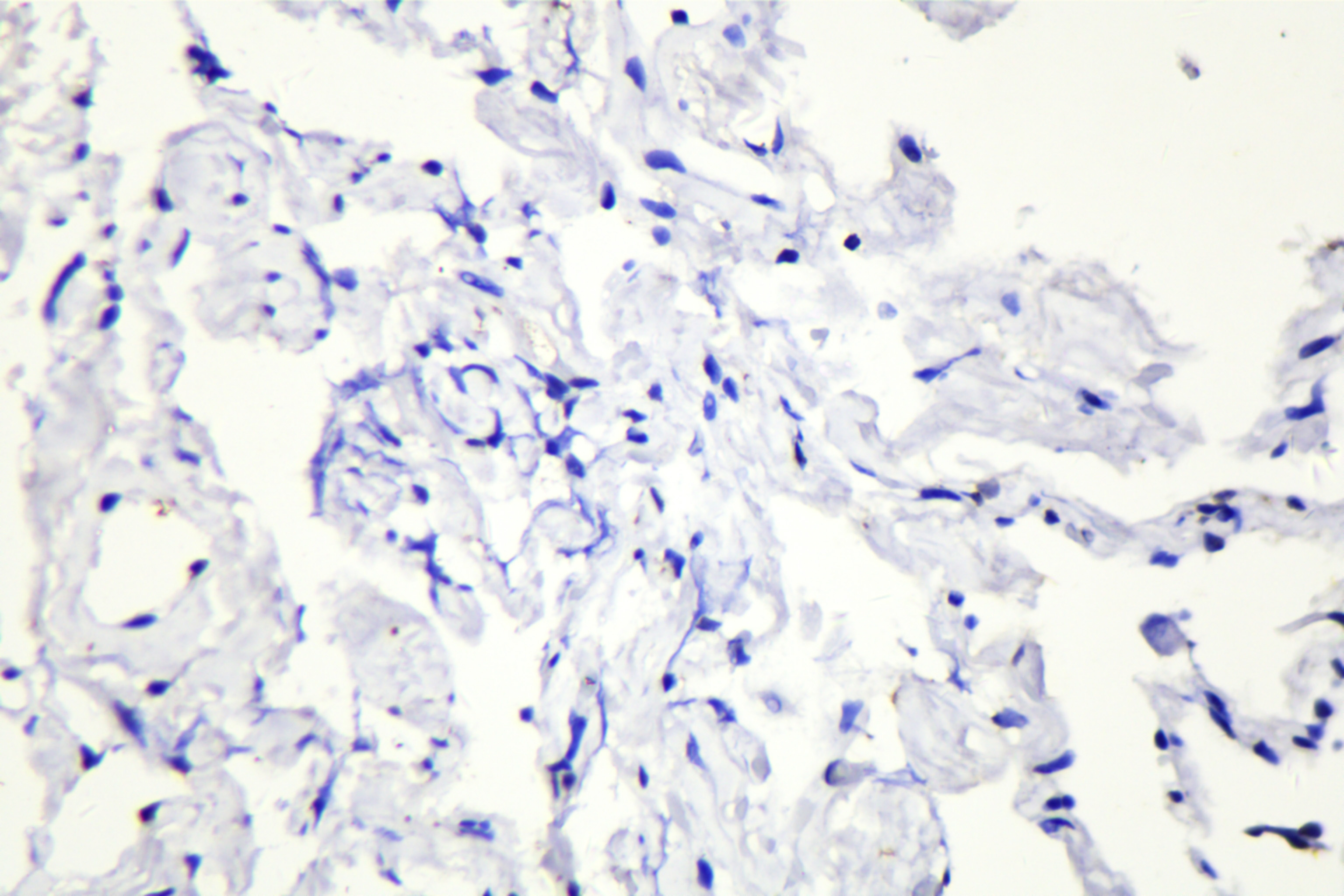

Supplement: Supplementary file 3 — Source data Fig. 1 [file 44321_2025_308_MOESM3_ESM.zip › Figure 1/1f/H240523LN 40X2(2).jpg]

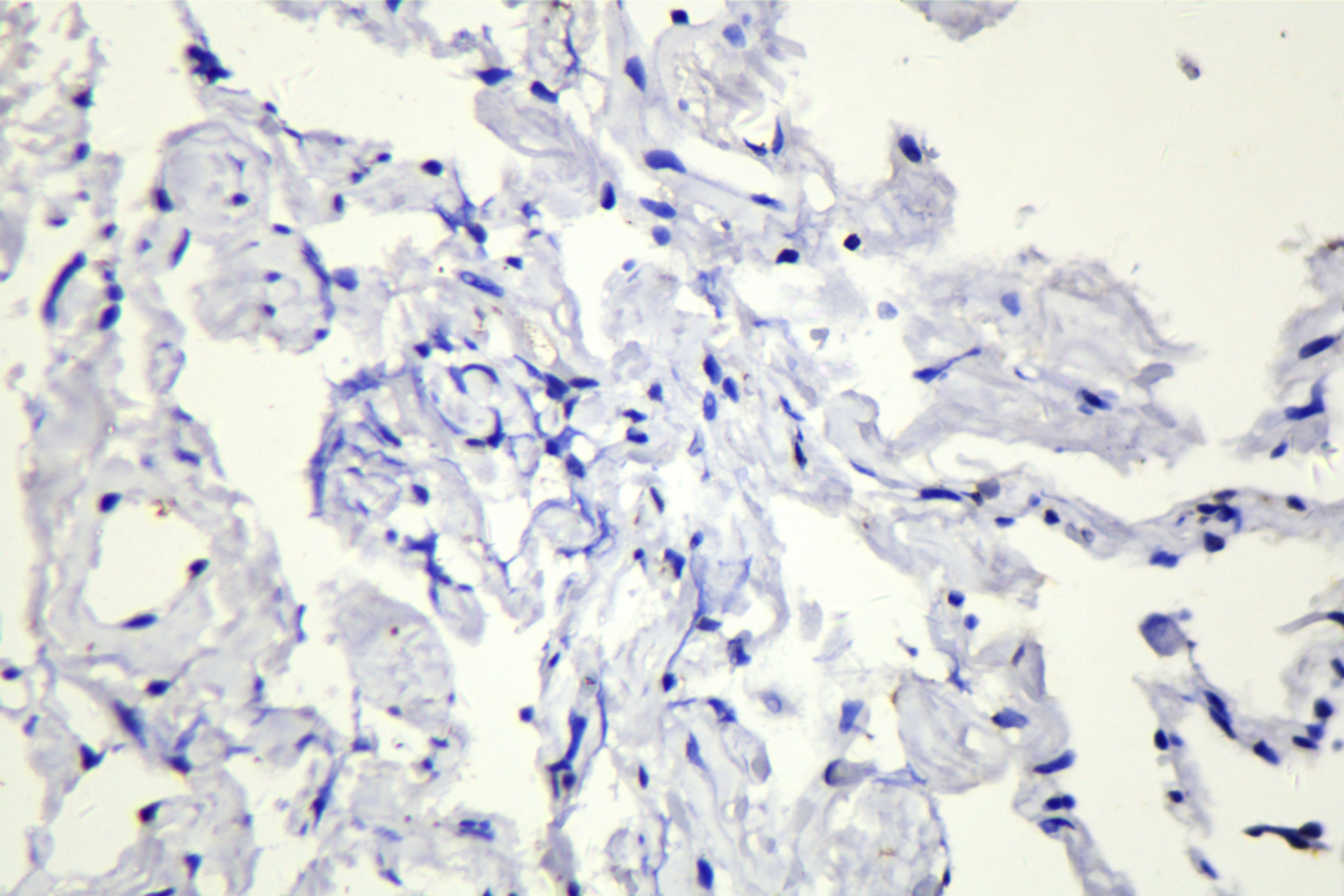

Supplement: Supplementary file 3 — Source data Fig. 1 [file 44321_2025_308_MOESM3_ESM.zip › Figure 1/1f/H240523LN 40X2.jpg]

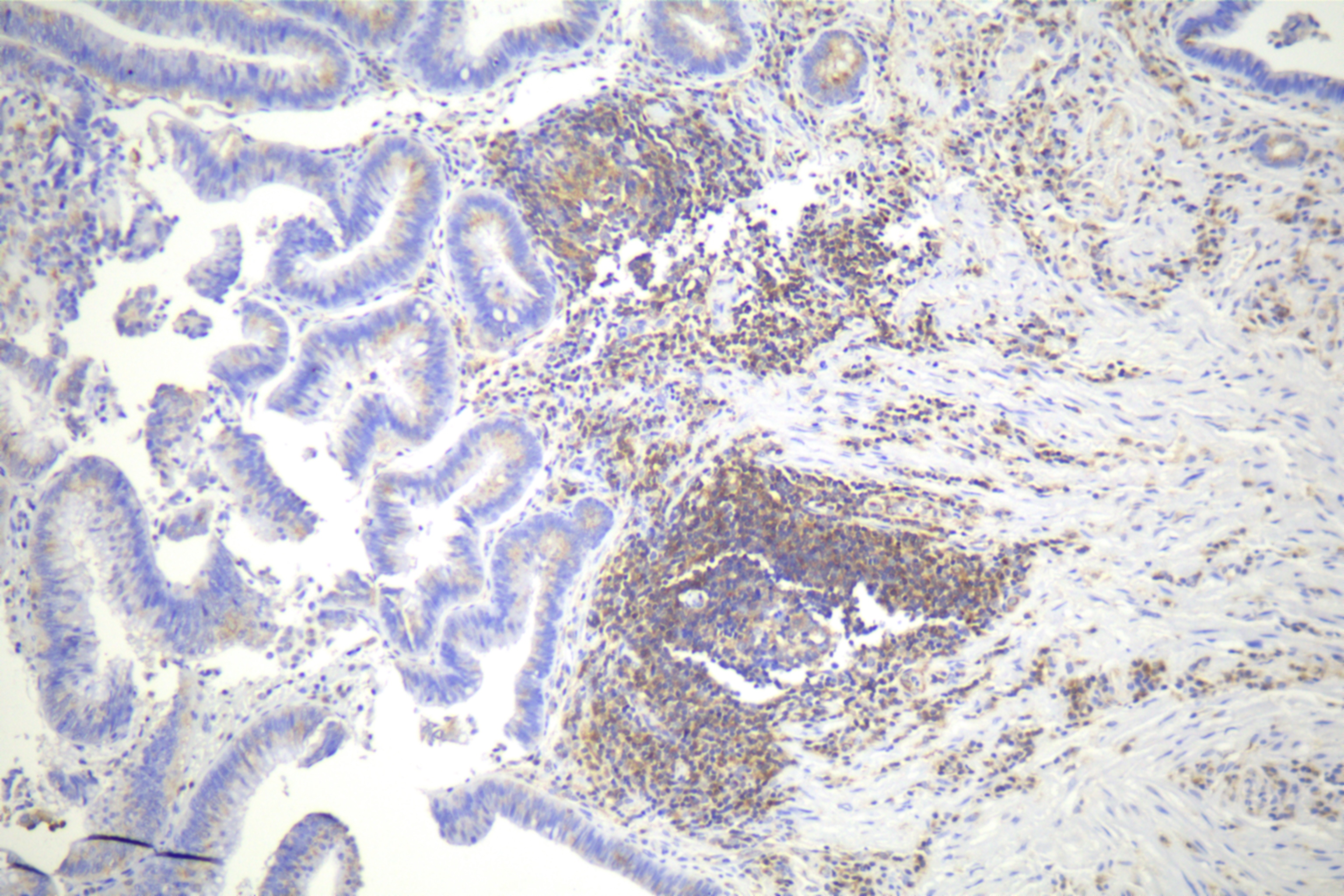

Supplement: Supplementary file 3 — Source data Fig. 1 [file 44321_2025_308_MOESM3_ESM.zip › Figure 1/1g/S24-002143 A2 10X 2.jpg]

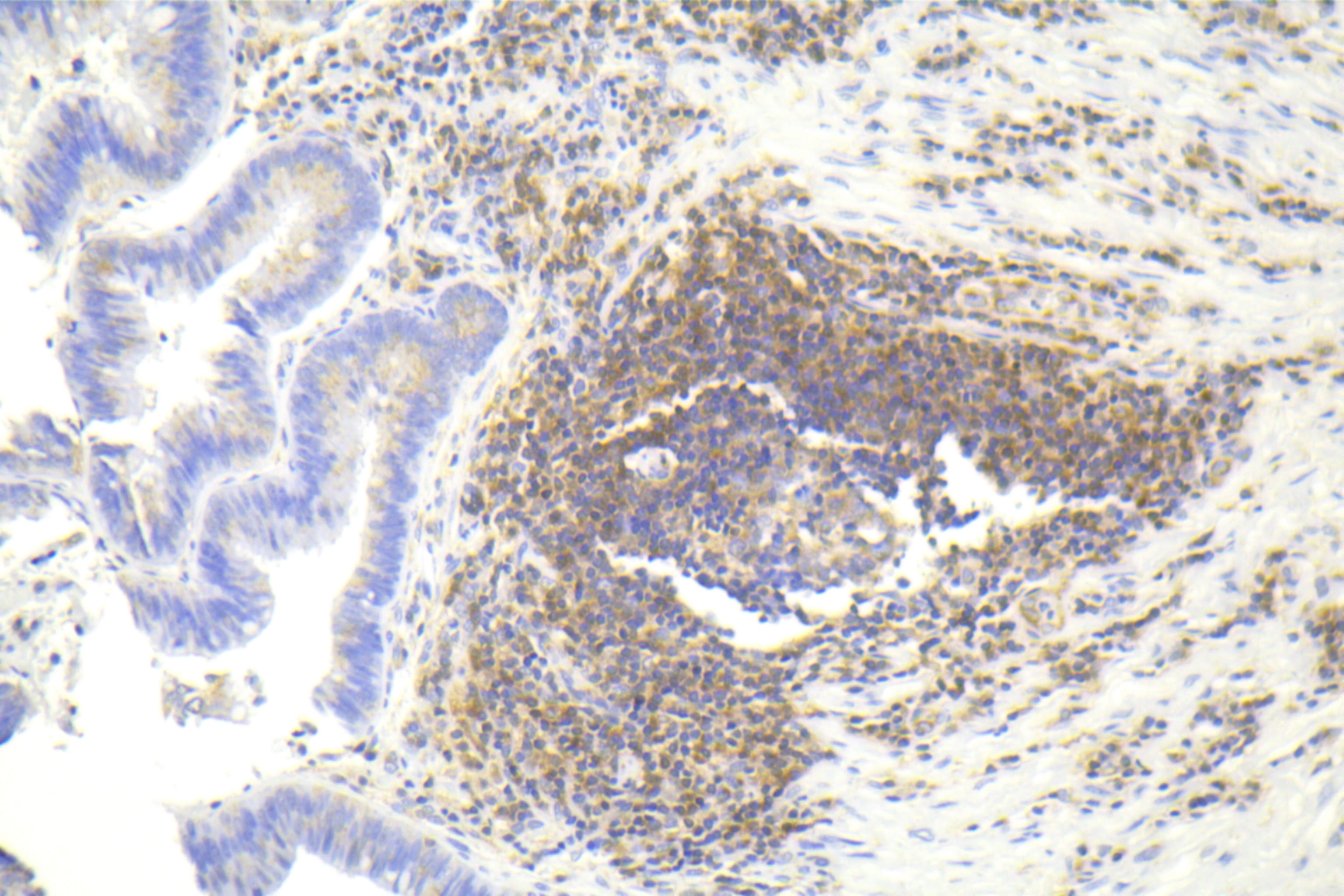

Supplement: Supplementary file 3 — Source data Fig. 1 [file 44321_2025_308_MOESM3_ESM.zip › Figure 1/1g/S24-002143 A2 20X 2.jpg]

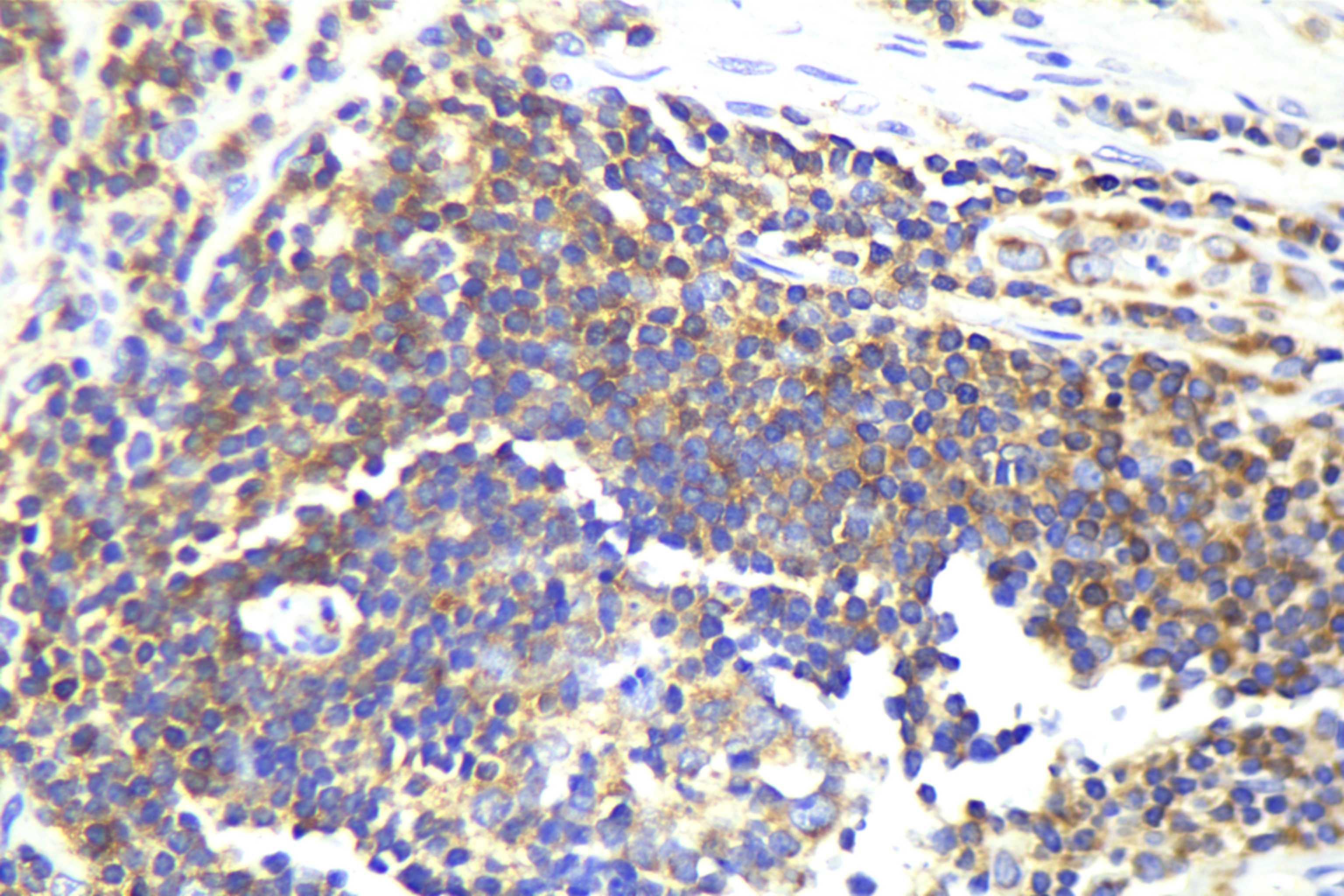

Supplement: Supplementary file 3 — Source data Fig. 1 [file 44321_2025_308_MOESM3_ESM.zip › Figure 1/1g/S24-002143 A2 40X 2.jpg]

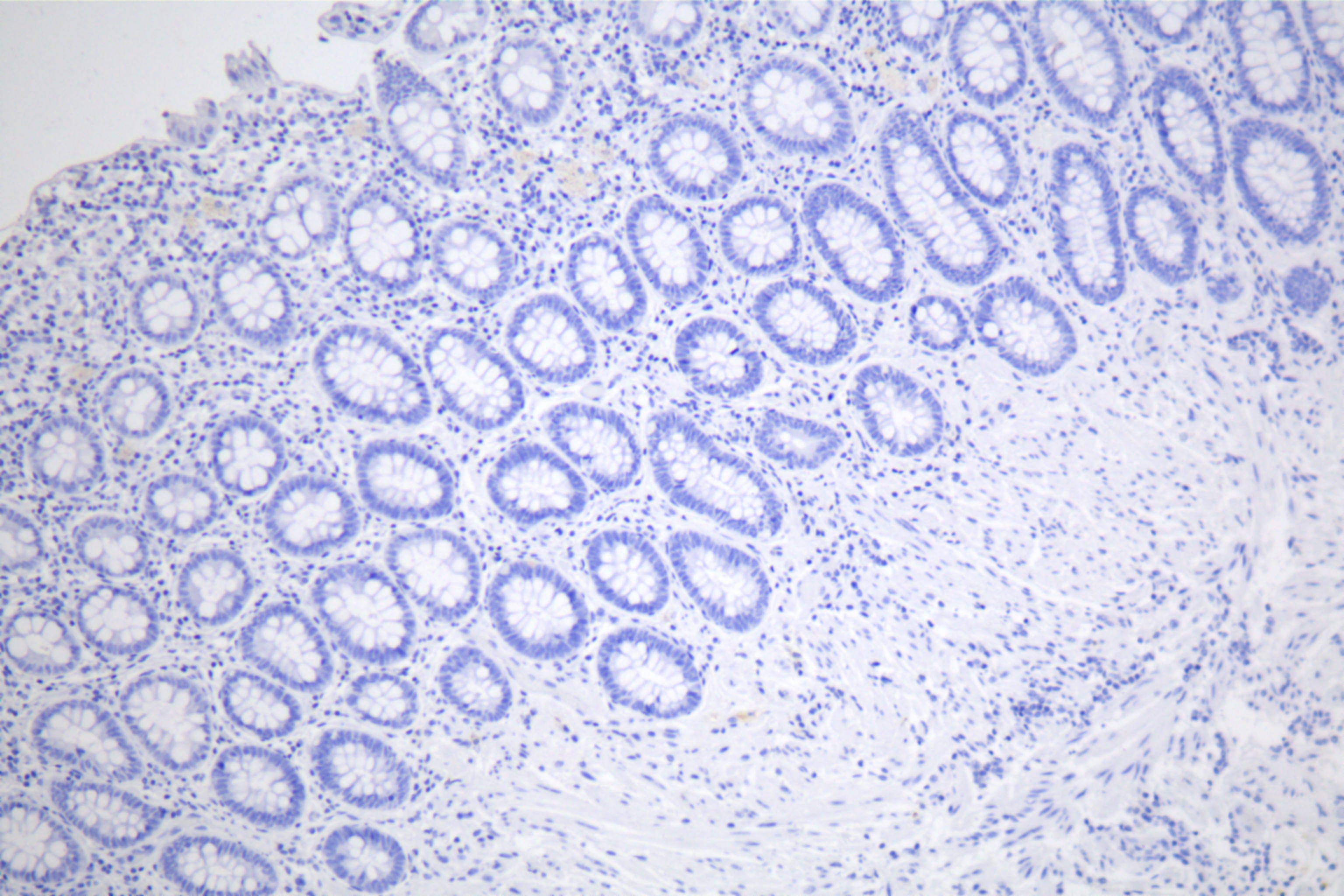

Supplement: Supplementary file 3 — Source data Fig. 1 [file 44321_2025_308_MOESM3_ESM.zip › Figure 1/1g/S24-002143 A8 10X 3.jpg]

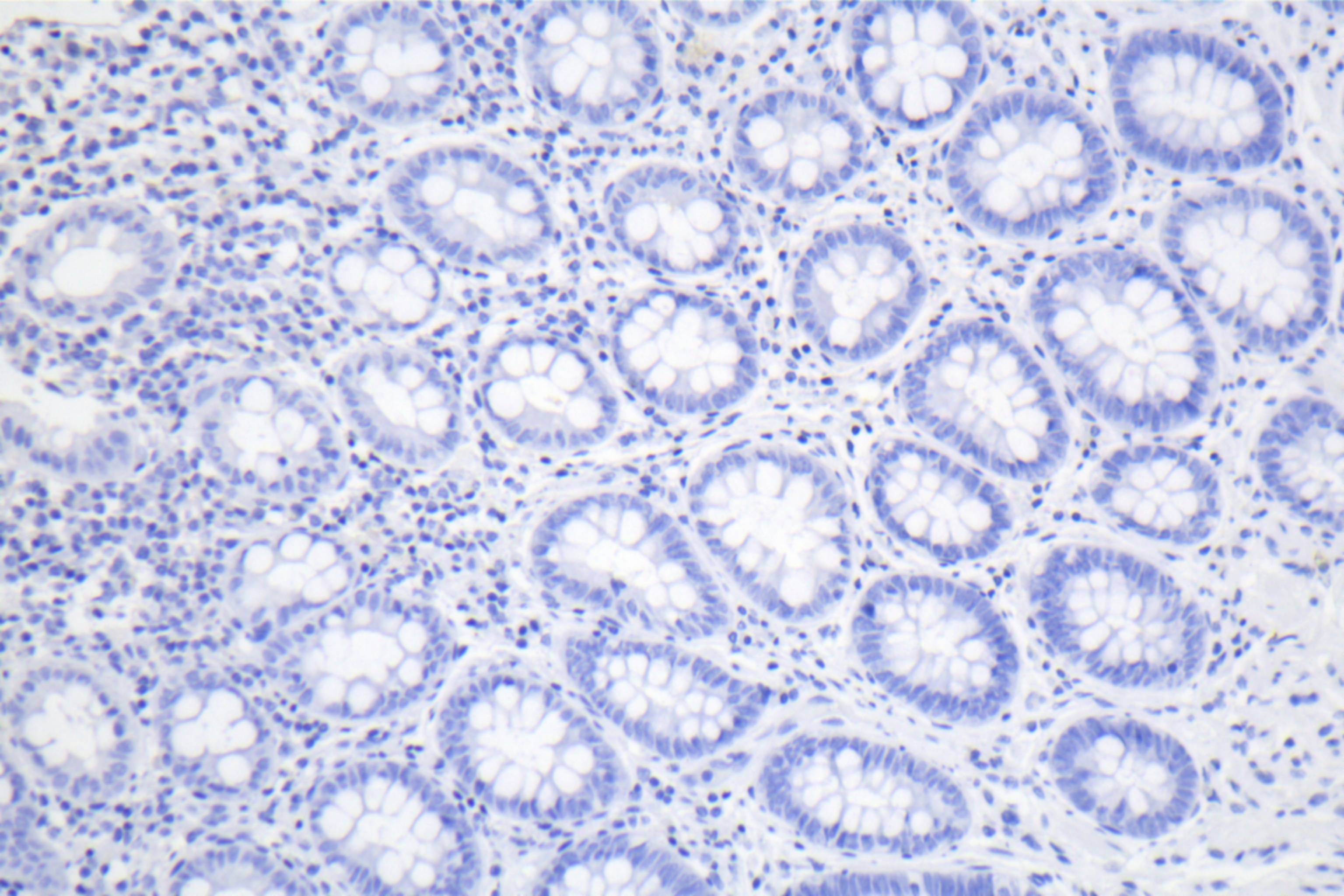

Supplement: Supplementary file 3 — Source data Fig. 1 [file 44321_2025_308_MOESM3_ESM.zip › Figure 1/1g/S24-002143 A8 20X 6.jpg]

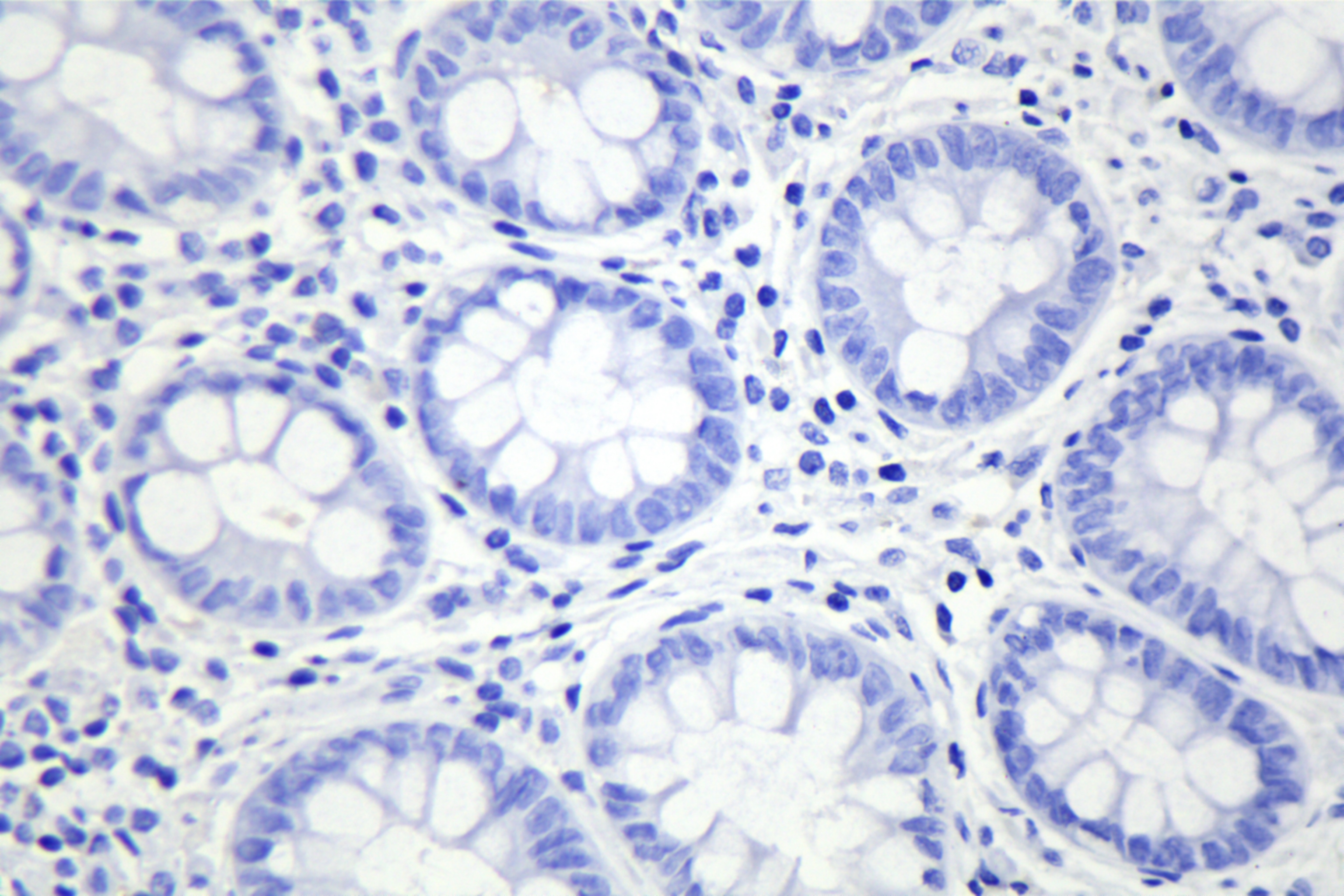

Supplement: Supplementary file 3 — Source data Fig. 1 [file 44321_2025_308_MOESM3_ESM.zip › Figure 1/1g/S24-002143 A8 40X 1.jpg]

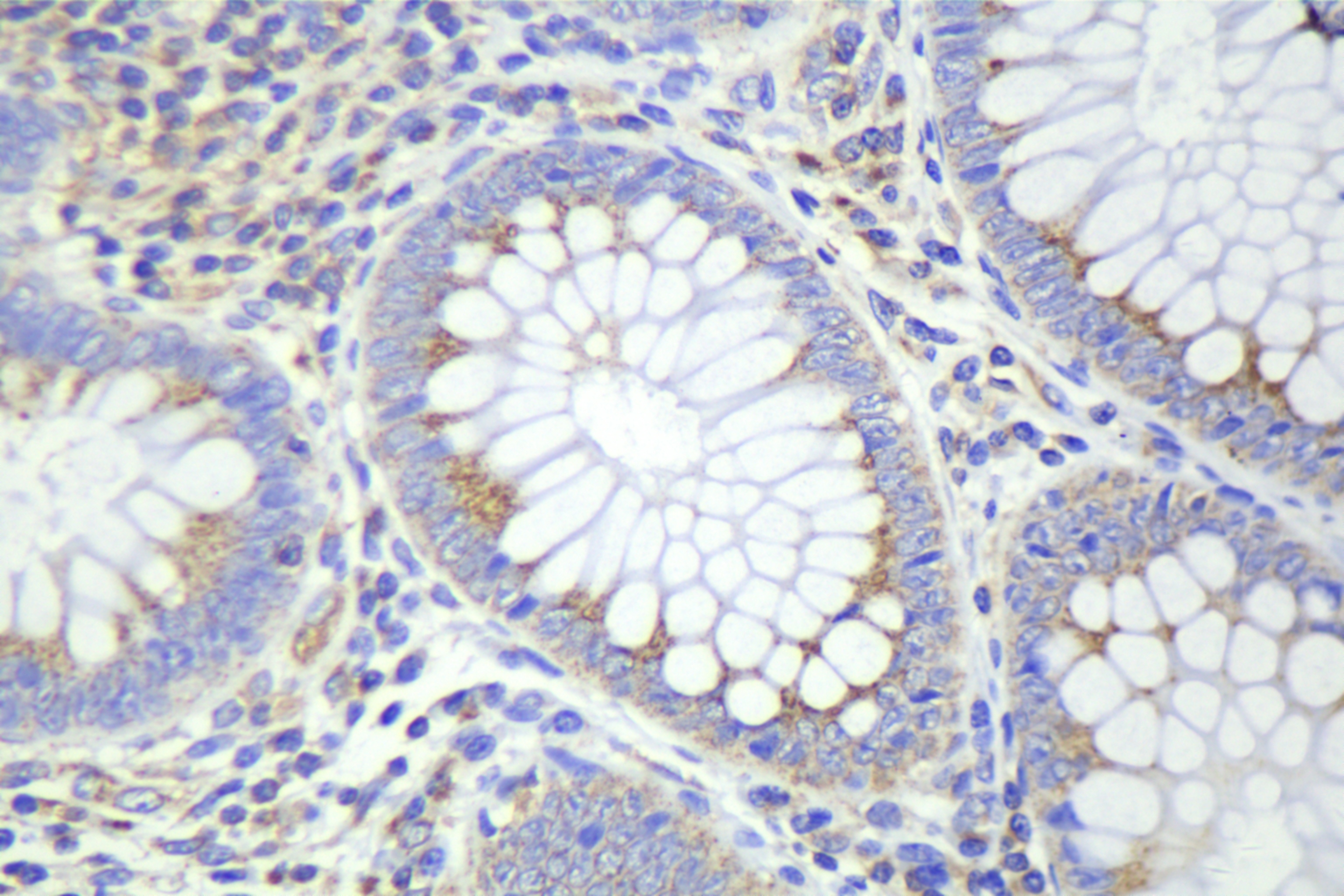

Supplement: Supplementary file 3 — Source data Fig. 1 [file 44321_2025_308_MOESM3_ESM.zip › Figure 1/h/S24-001706 A6 40X 3.jpg]

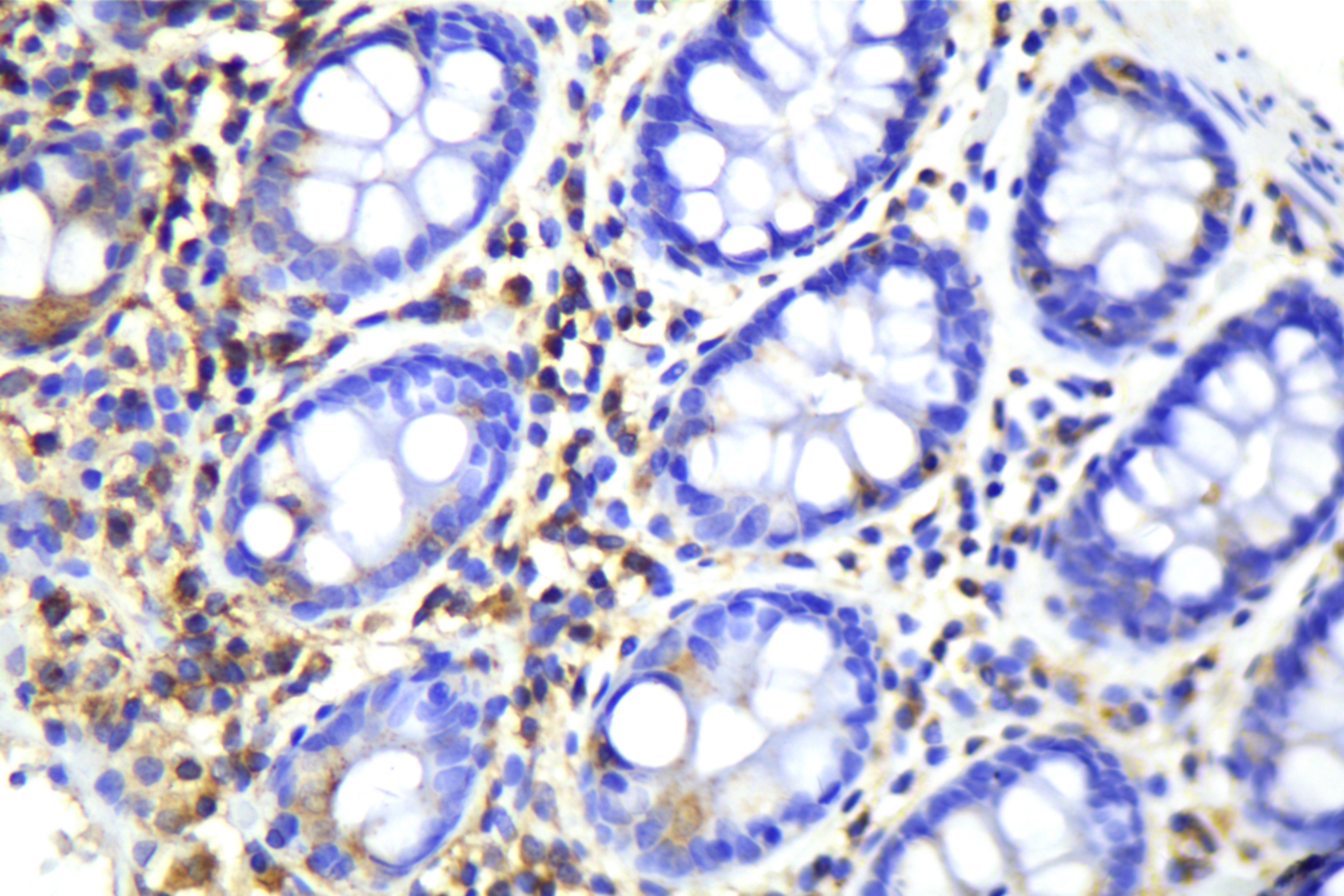

Supplement: Supplementary file 3 — Source data Fig. 1 [file 44321_2025_308_MOESM3_ESM.zip › Figure 1/h/S24-001706 A8 40X 1.jpg]

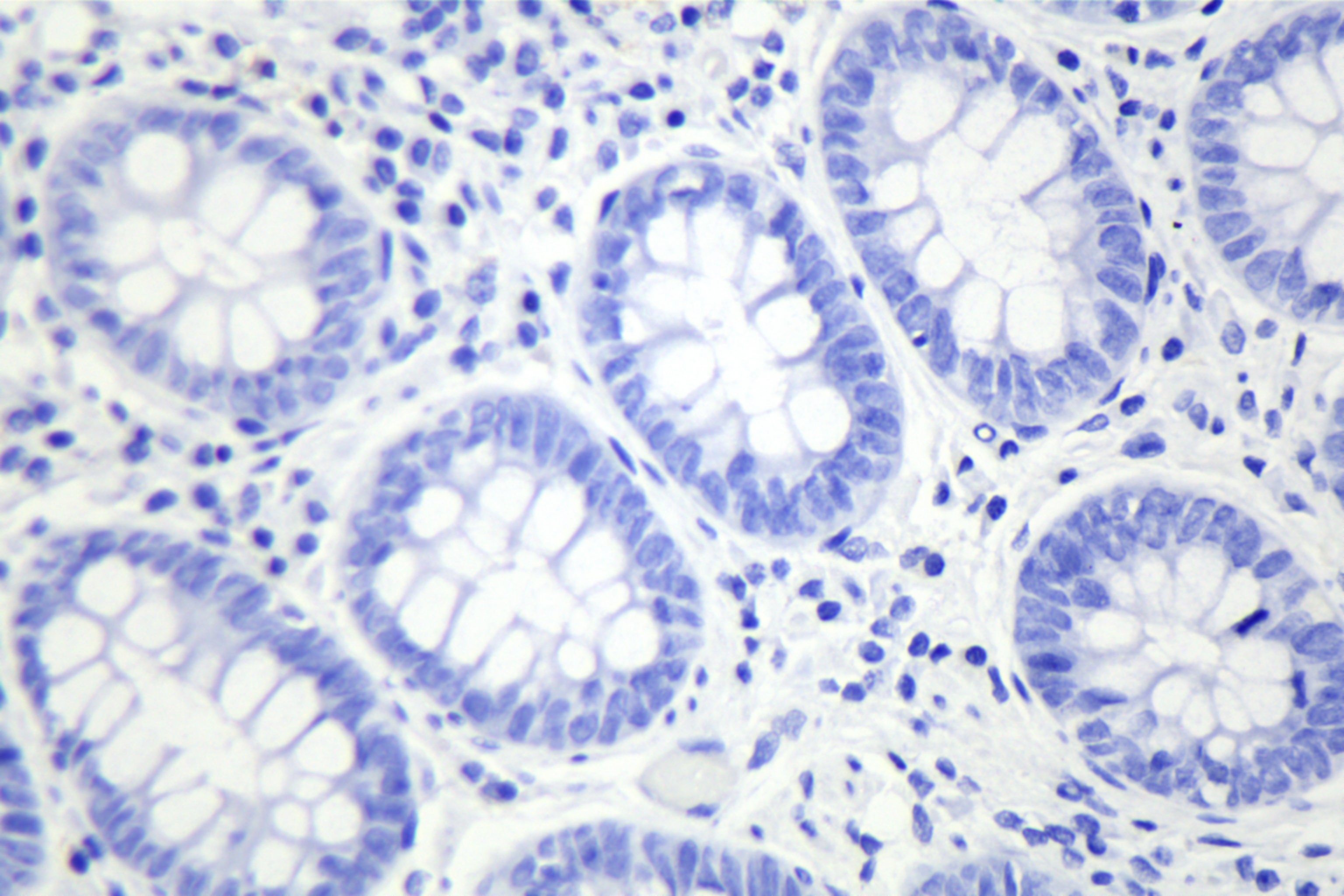

Supplement: Supplementary file 3 — Source data Fig. 1 [file 44321_2025_308_MOESM3_ESM.zip › Figure 1/h/S24-001713 A3 40X.jpg]

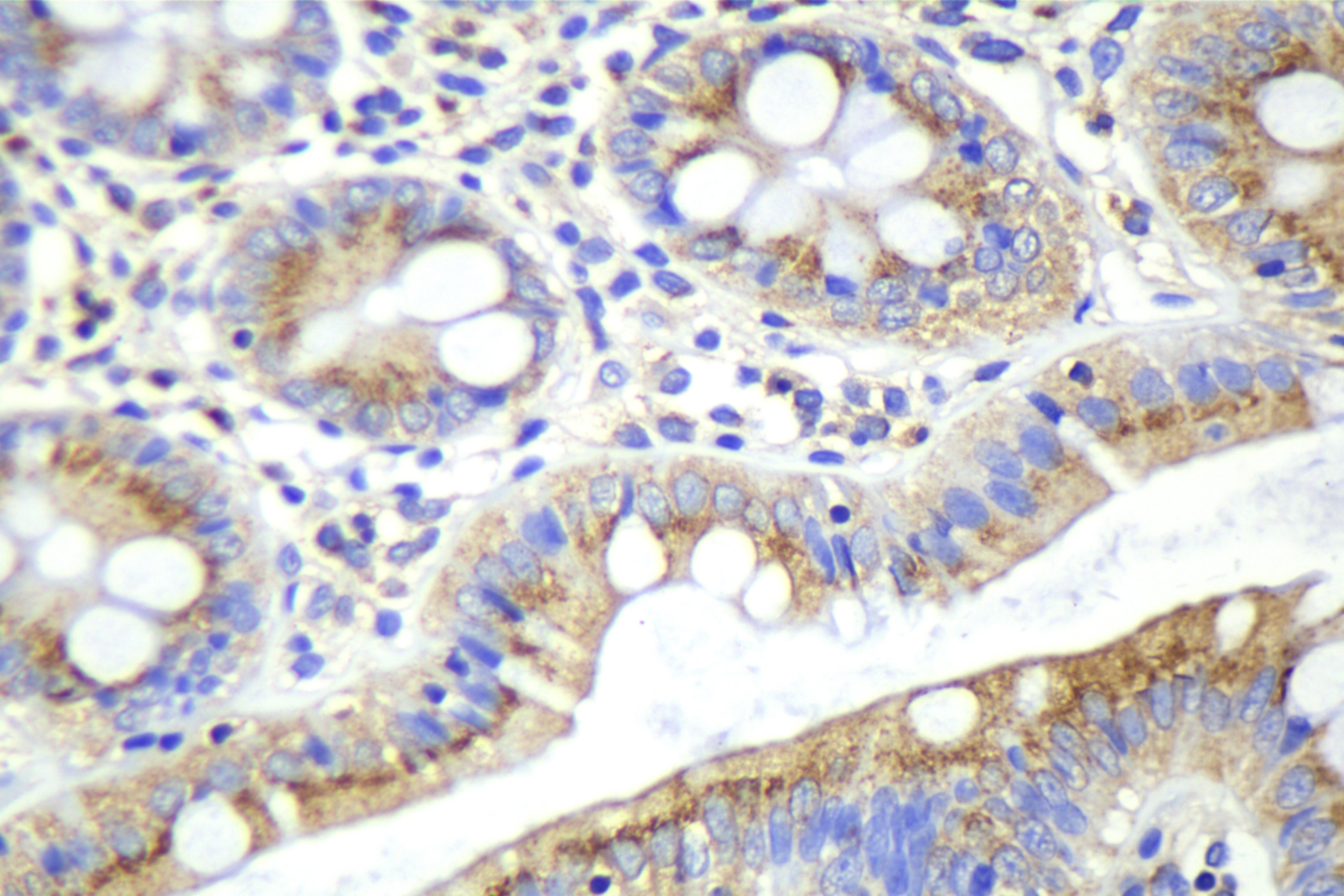

Supplement: Supplementary file 3 — Source data Fig. 1 [file 44321_2025_308_MOESM3_ESM.zip › Figure 1/h/S24-001713 A7 40X.jpg]

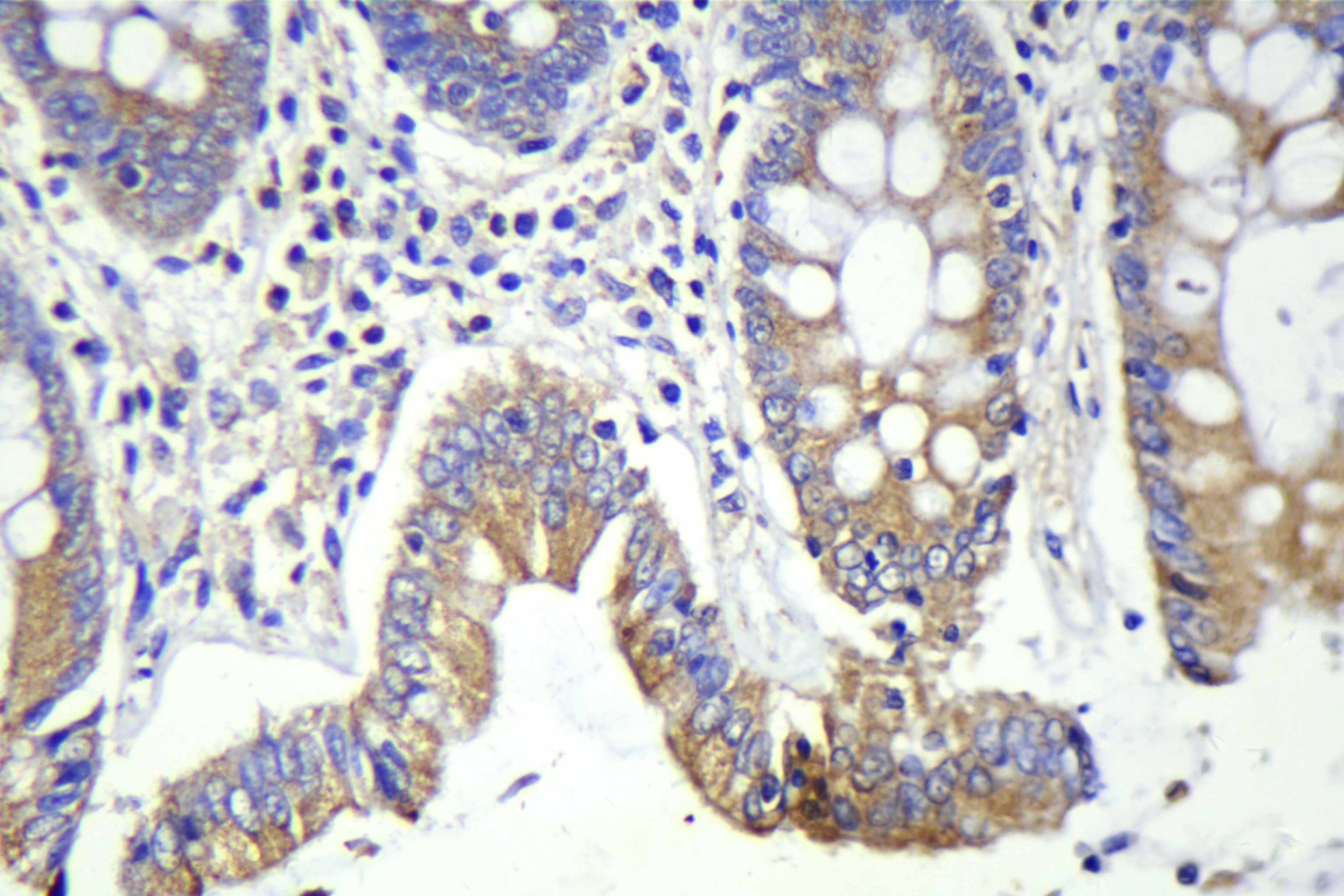

Supplement: Supplementary file 3 — Source data Fig. 1 [file 44321_2025_308_MOESM3_ESM.zip › Figure 1/h/S24-001718 A10 40X.jpg]

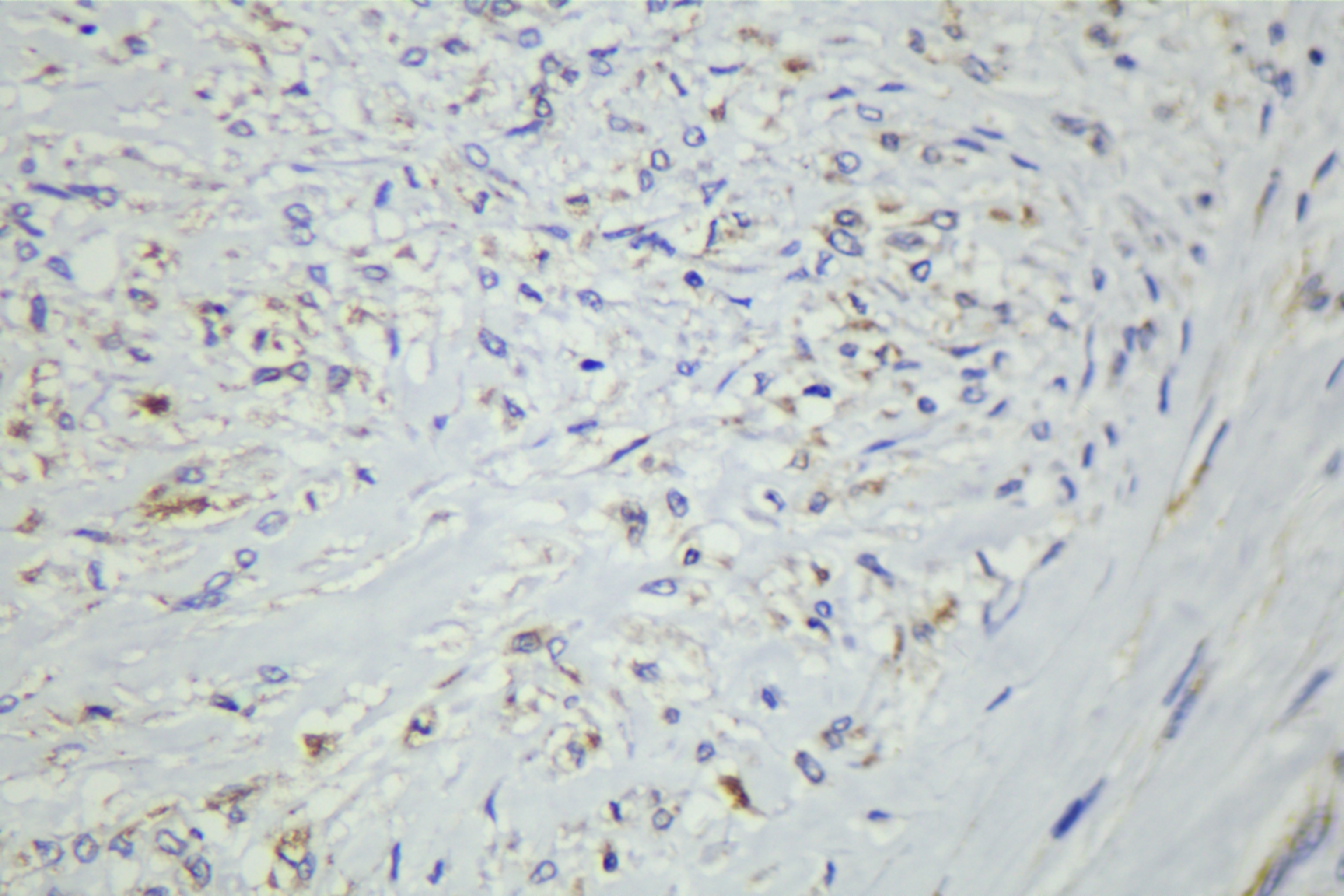

Supplement: Supplementary file 3 — Source data Fig. 1 [file 44321_2025_308_MOESM3_ESM.zip › Figure 1/h/S24-001718 A5 40X 2.jpg]

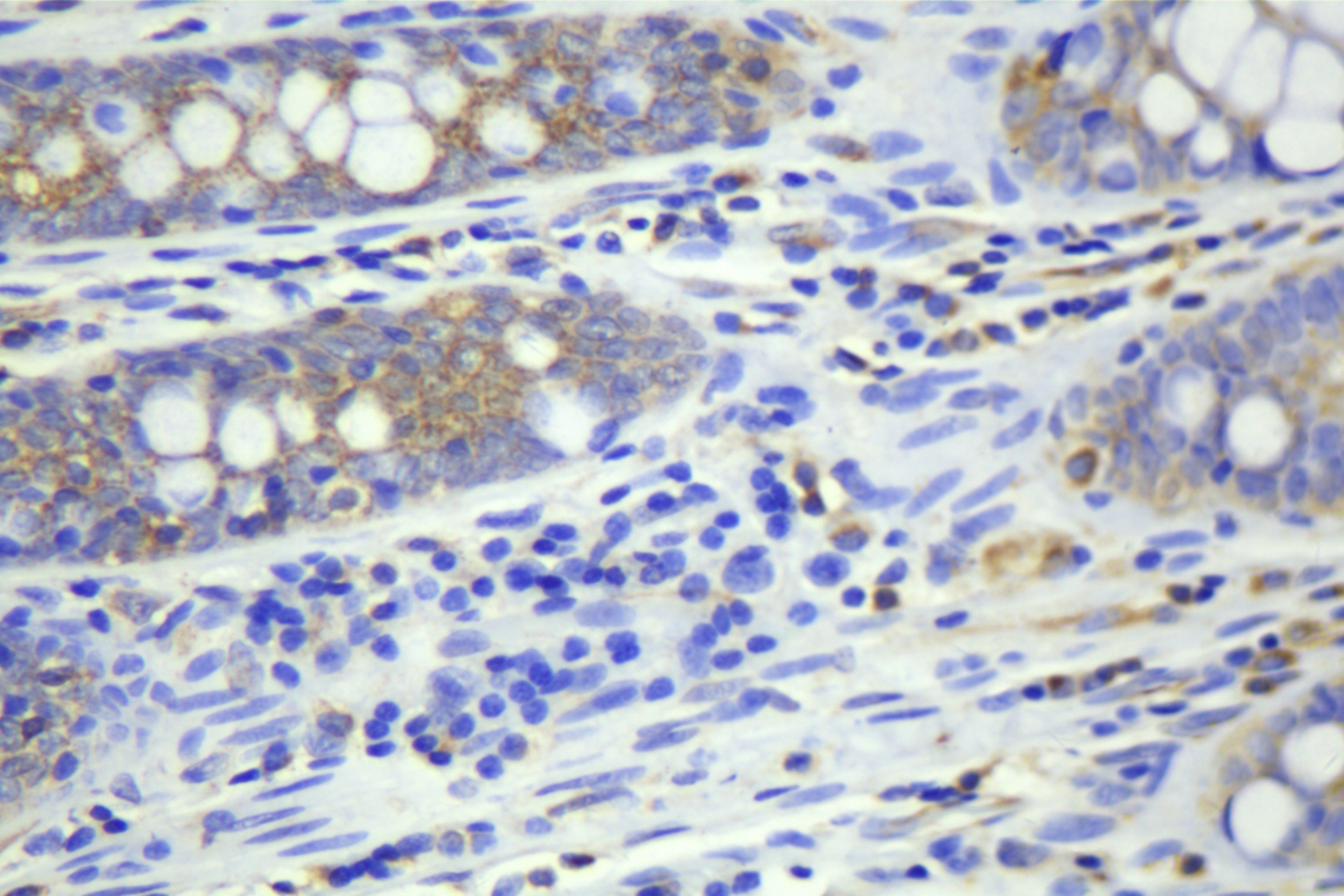

Supplement: Supplementary file 3 — Source data Fig. 1 [file 44321_2025_308_MOESM3_ESM.zip › Figure 1/h/S24-001848 A2 40X 2.jpg]

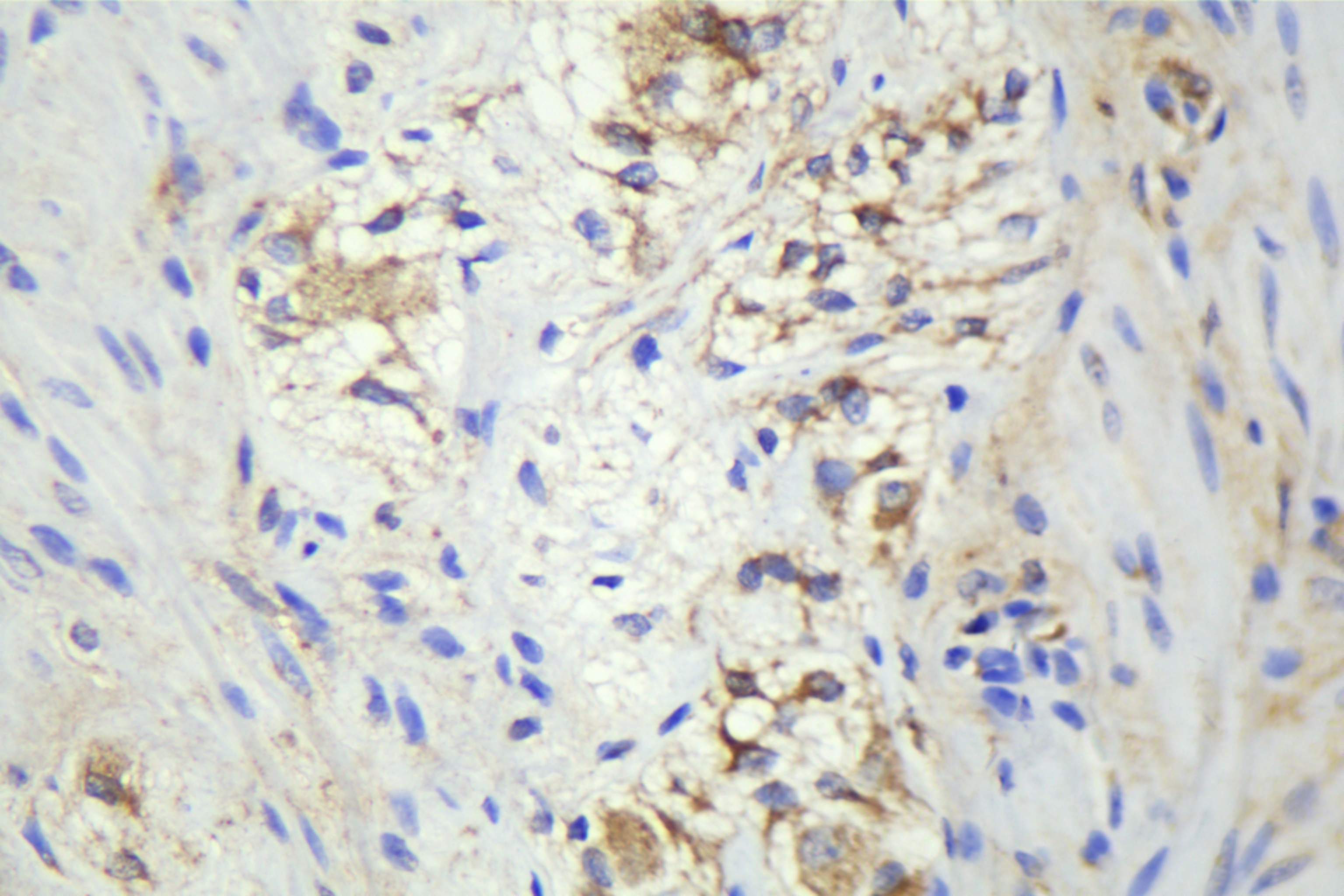

Supplement: Supplementary file 3 — Source data Fig. 1 [file 44321_2025_308_MOESM3_ESM.zip › Figure 1/h/S24-001848 A8 40X.jpg]

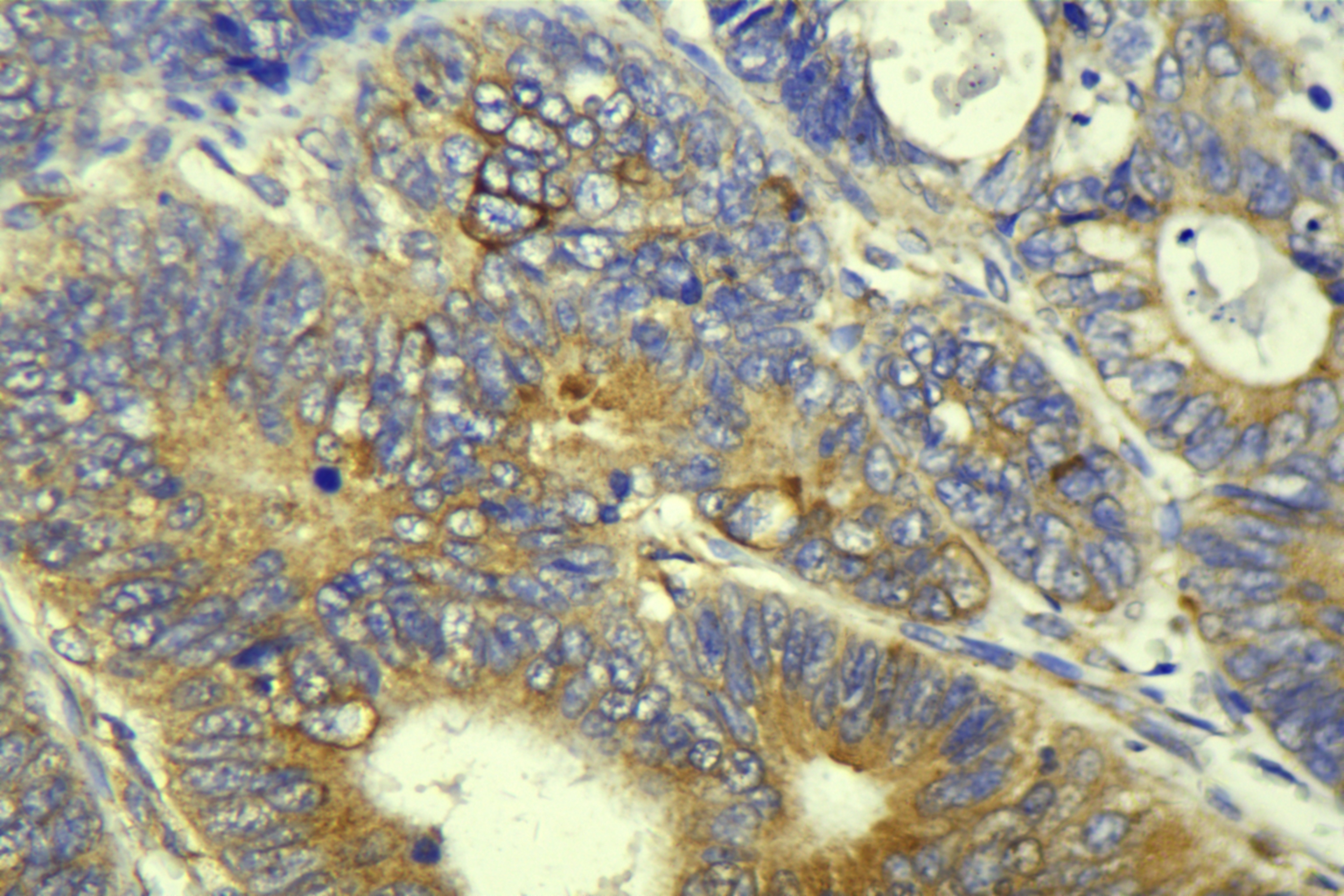

Supplement: Supplementary file 3 — Source data Fig. 1 [file 44321_2025_308_MOESM3_ESM.zip › Figure 1/h/S24-001991 A3 40X 2.jpg]

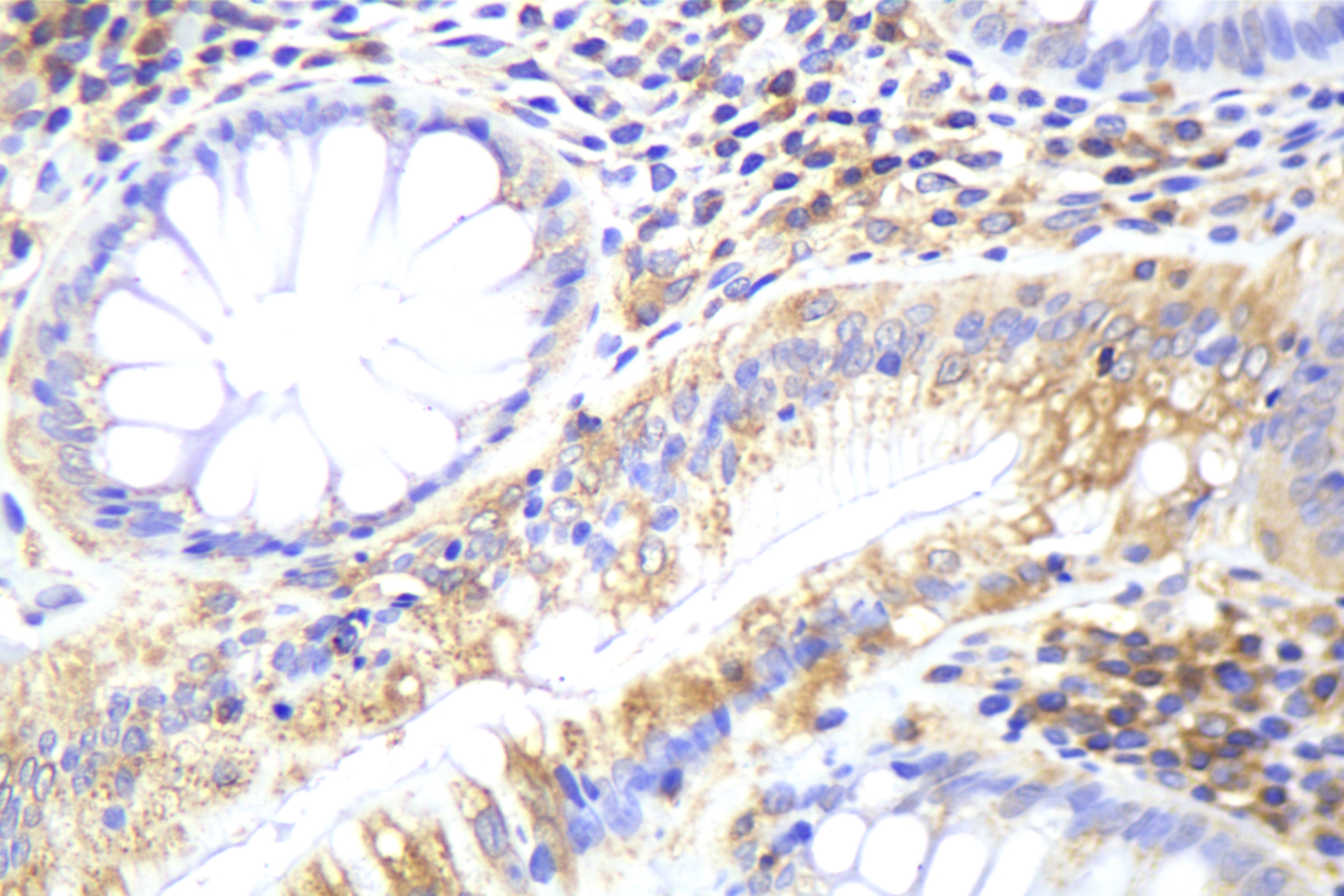

Supplement: Supplementary file 3 — Source data Fig. 1 [file 44321_2025_308_MOESM3_ESM.zip › Figure 1/h/S24-001991 A8 40X.jpg]

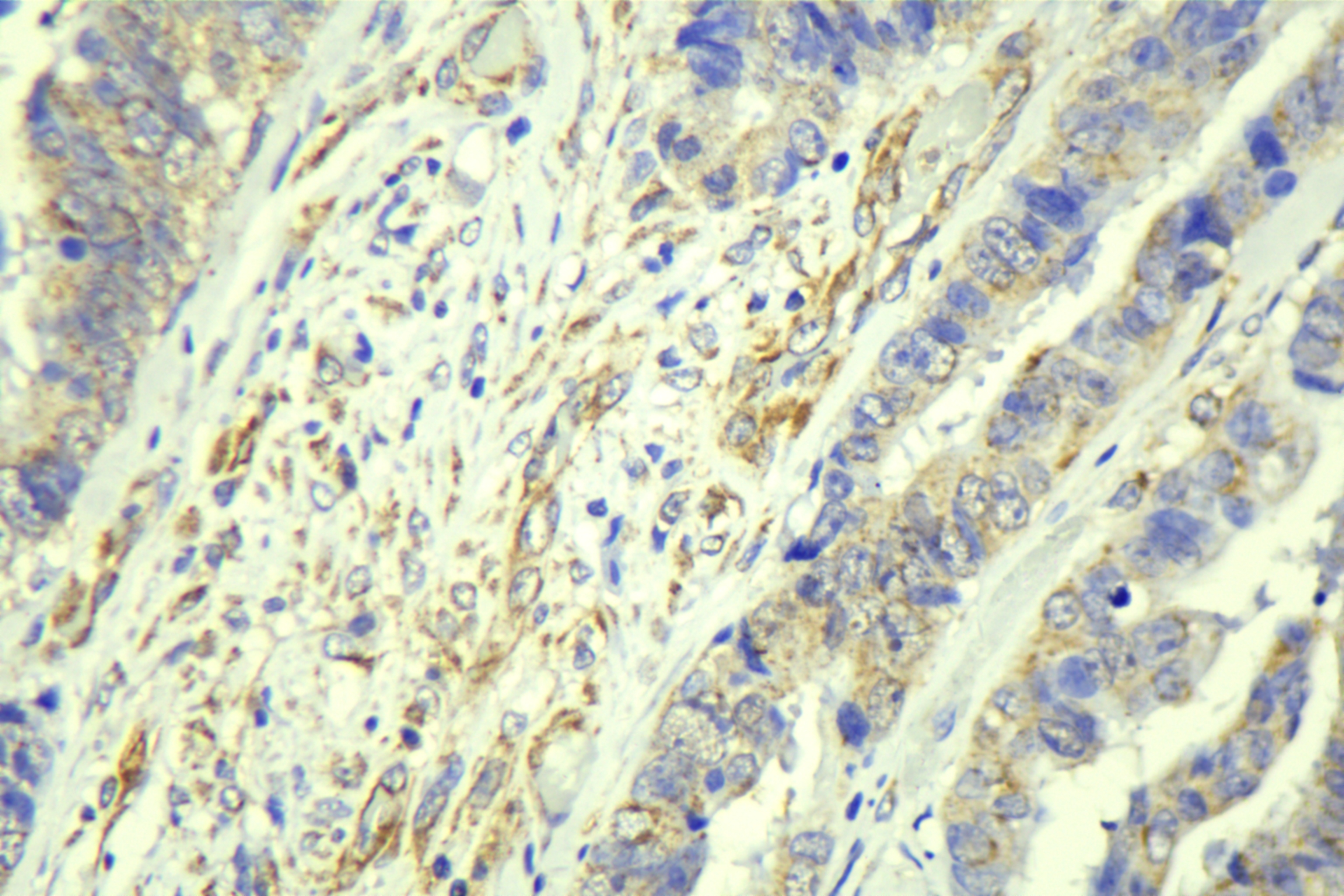

Supplement: Supplementary file 3 — Source data Fig. 1 [file 44321_2025_308_MOESM3_ESM.zip › Figure 1/h/S24-002000 A3 40X 2.jpg]

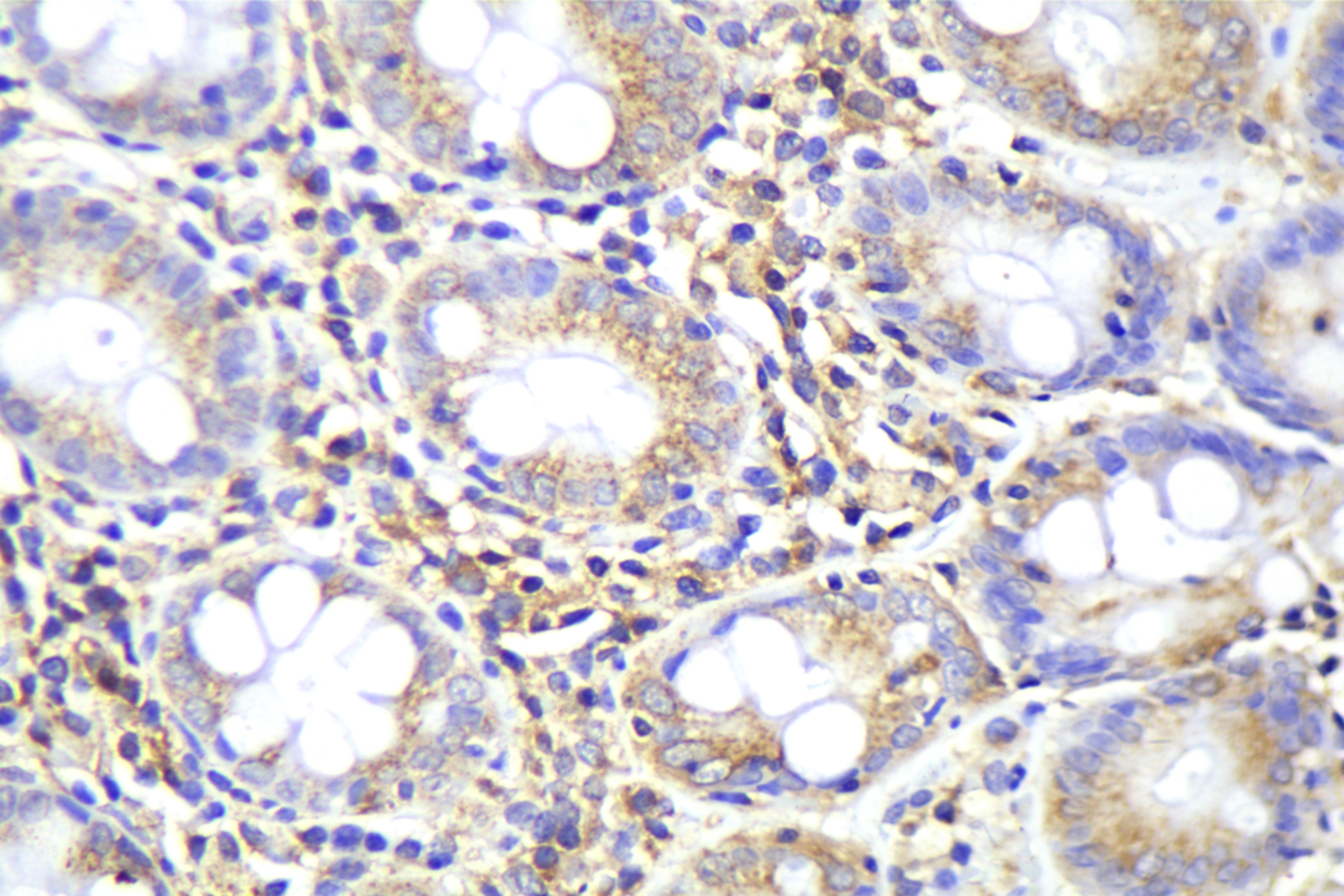

Supplement: Supplementary file 3 — Source data Fig. 1 [file 44321_2025_308_MOESM3_ESM.zip › Figure 1/h/S24-002000 A8 40X 4.jpg]

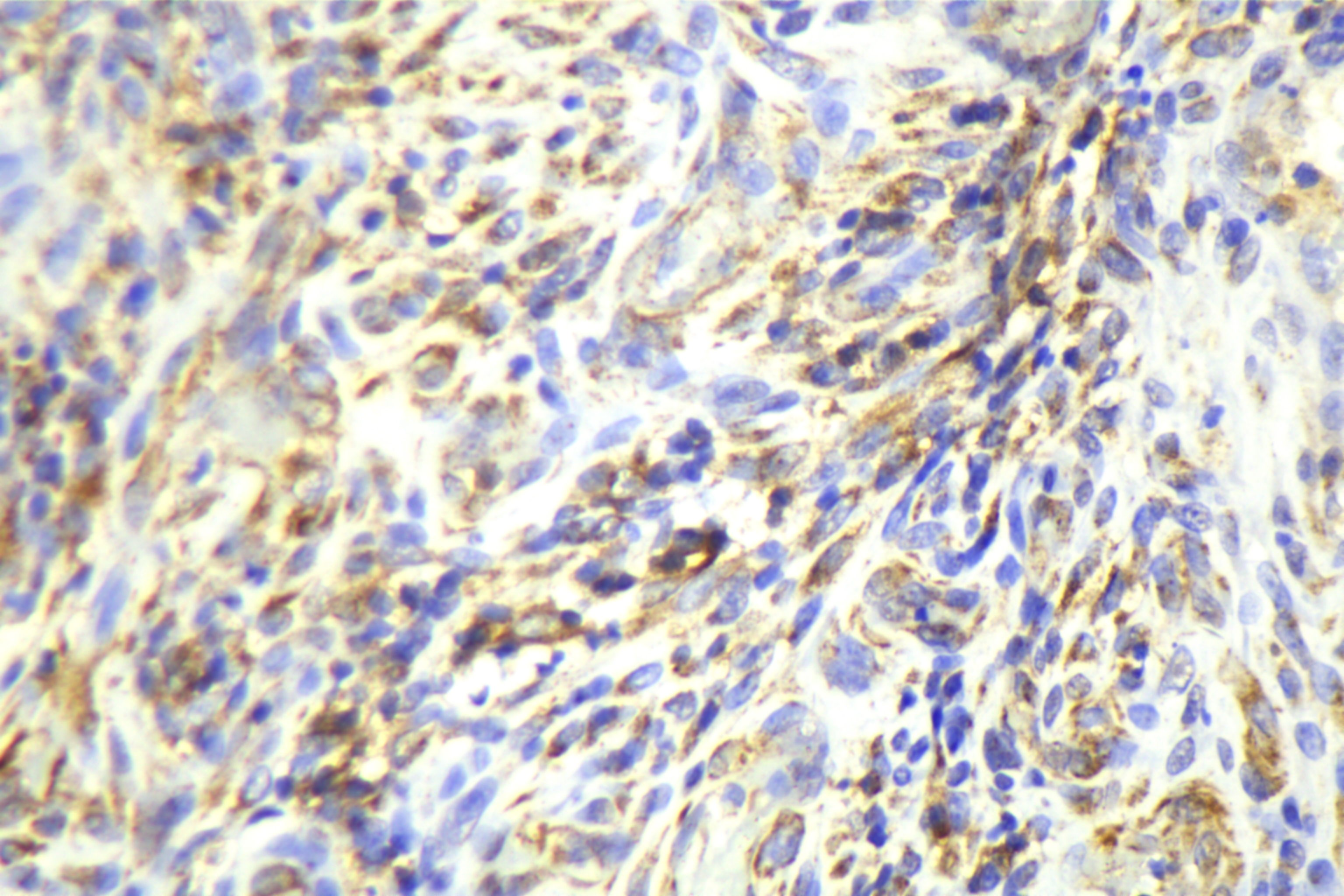

Supplement: Supplementary file 3 — Source data Fig. 1 [file 44321_2025_308_MOESM3_ESM.zip › Figure 1/h/S24-002547 A2 40X3.jpg]

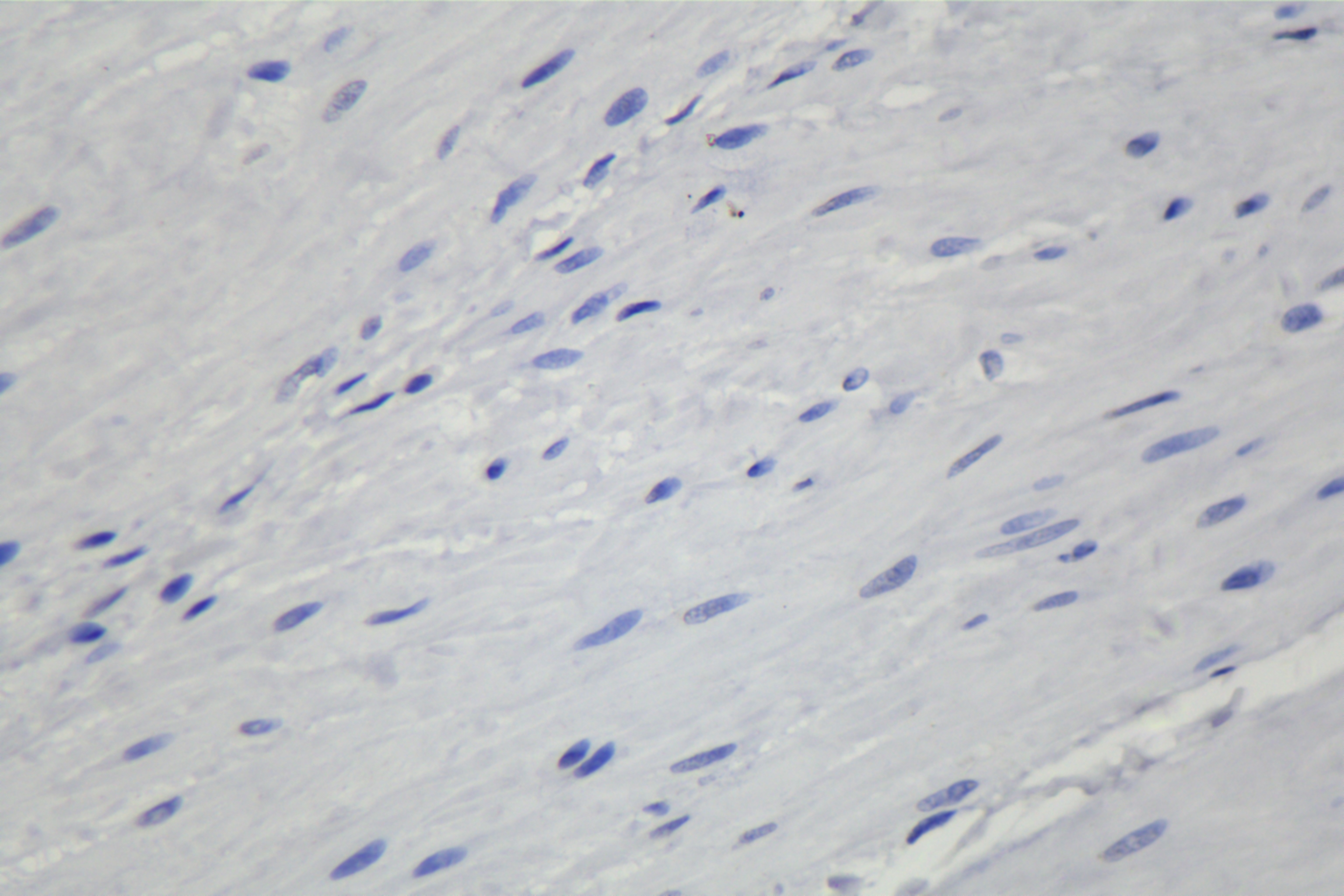

Supplement: Supplementary file 3 — Source data Fig. 1 [file 44321_2025_308_MOESM3_ESM.zip › Figure 1/h/S24-002547 A8 40X3.jpg]

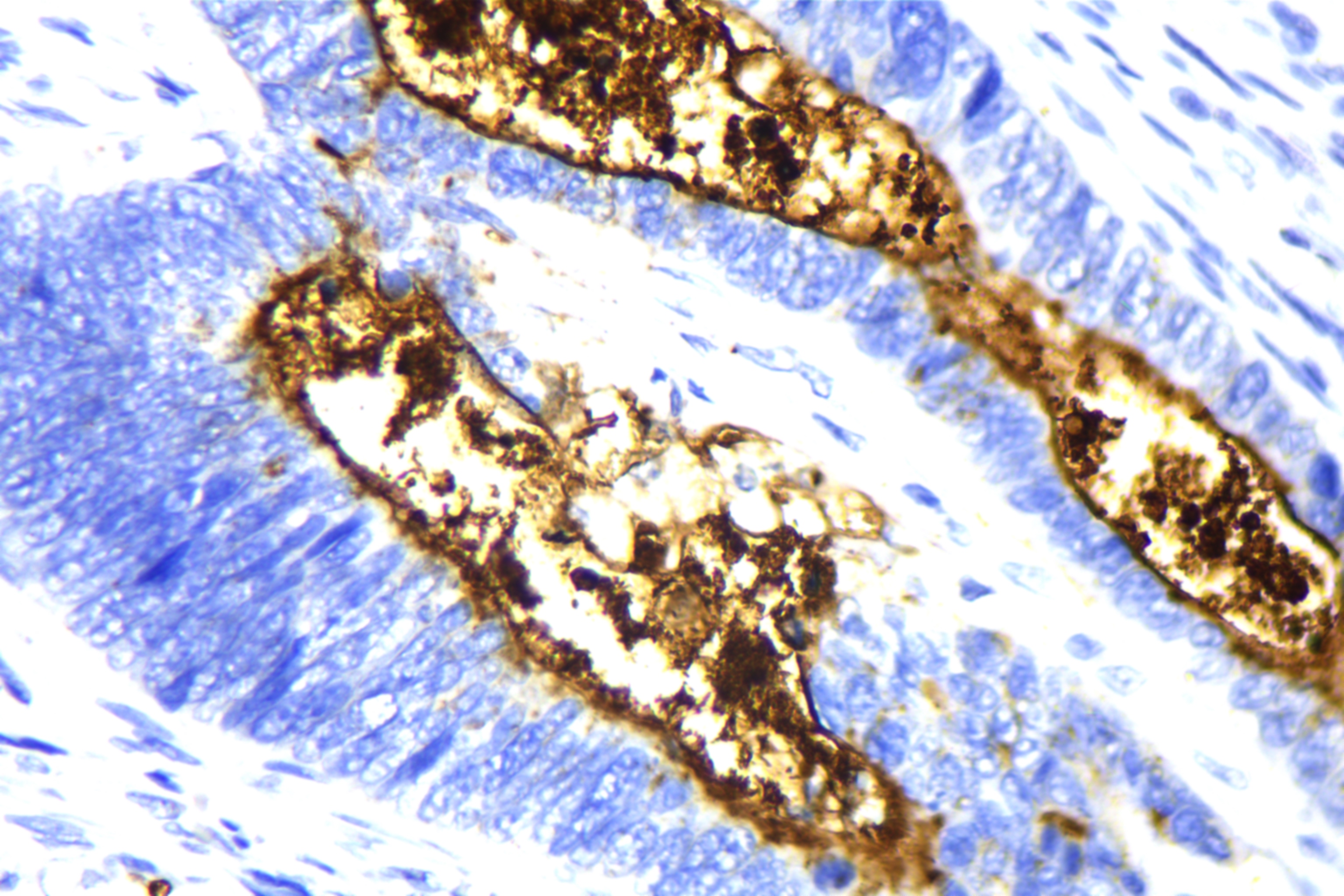

Supplement: Supplementary file 3 — Source data Fig. 1 [file 44321_2025_308_MOESM3_ESM.zip › Figure 1/h/S24-2547A3 40X 4.jpg]

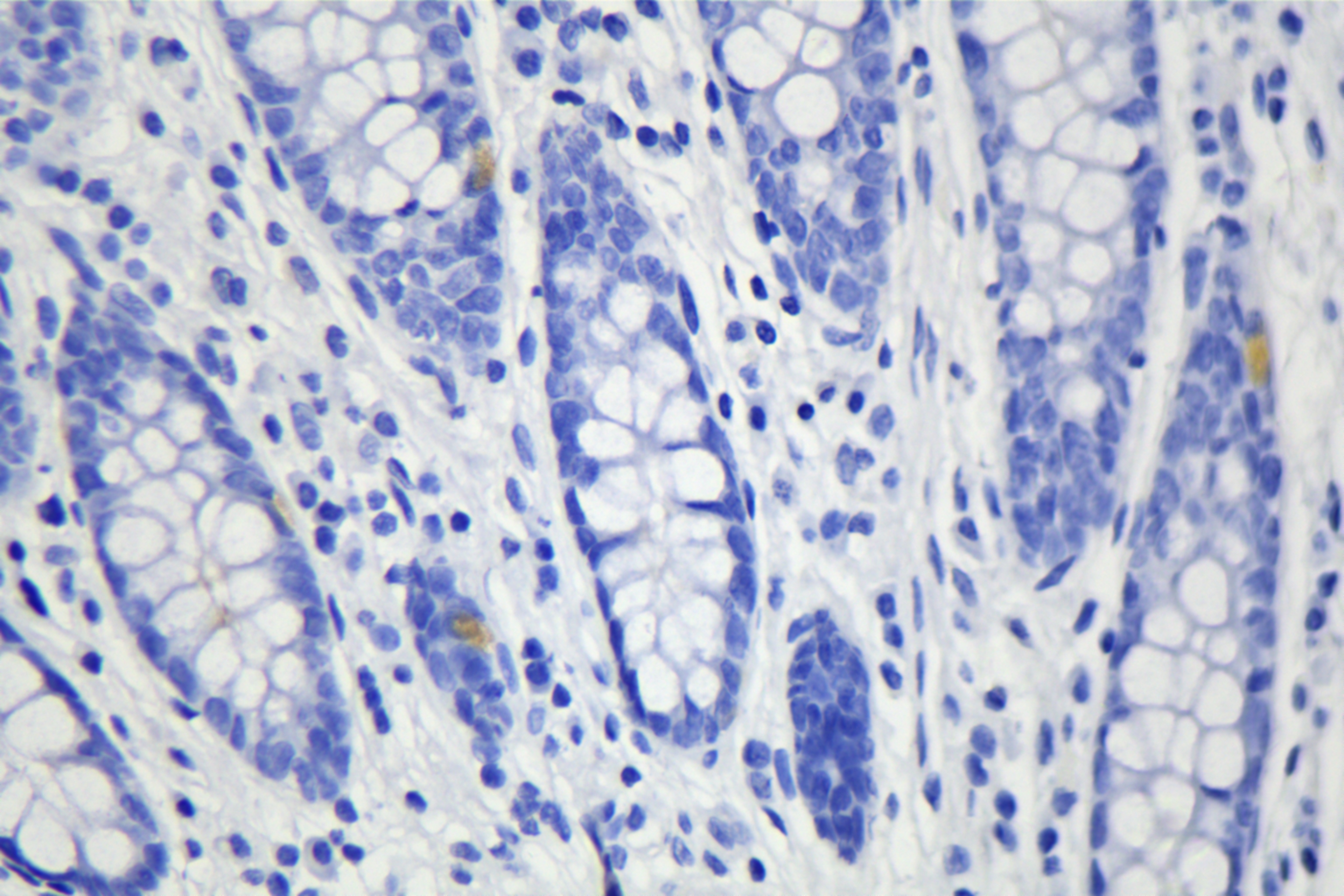

Supplement: Supplementary file 3 — Source data Fig. 1 [file 44321_2025_308_MOESM3_ESM.zip › Figure 1/h/S24-2695 A7 40X2.jpg]

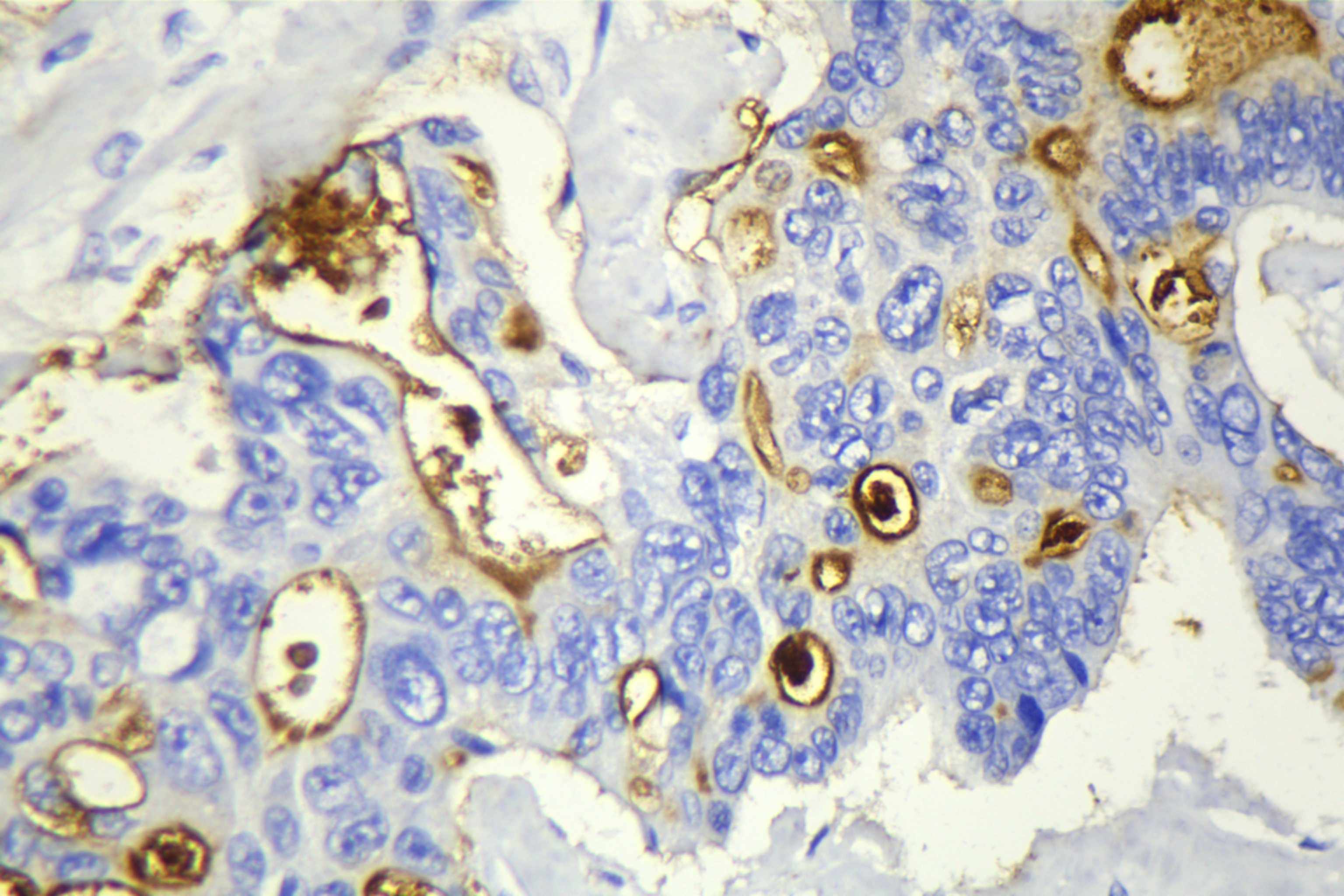

Supplement: Supplementary file 3 — Source data Fig. 1 [file 44321_2025_308_MOESM3_ESM.zip › Figure 1/h/S24-2695A1 40X 2.jpg]

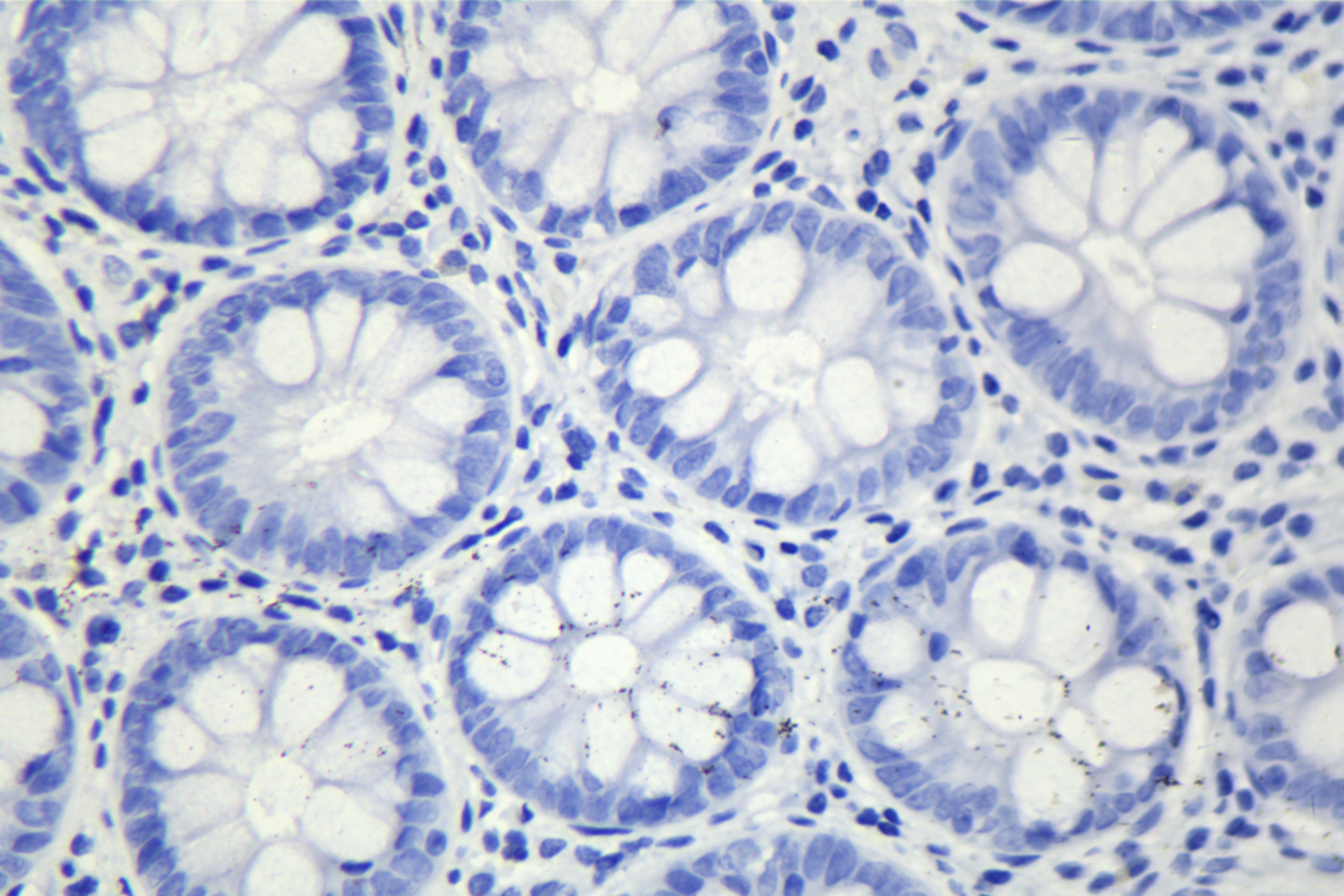

Supplement: Supplementary file 3 — Source data Fig. 1 [file 44321_2025_308_MOESM3_ESM.zip › Figure 1/h/S24-2696A7 40X.jpg]

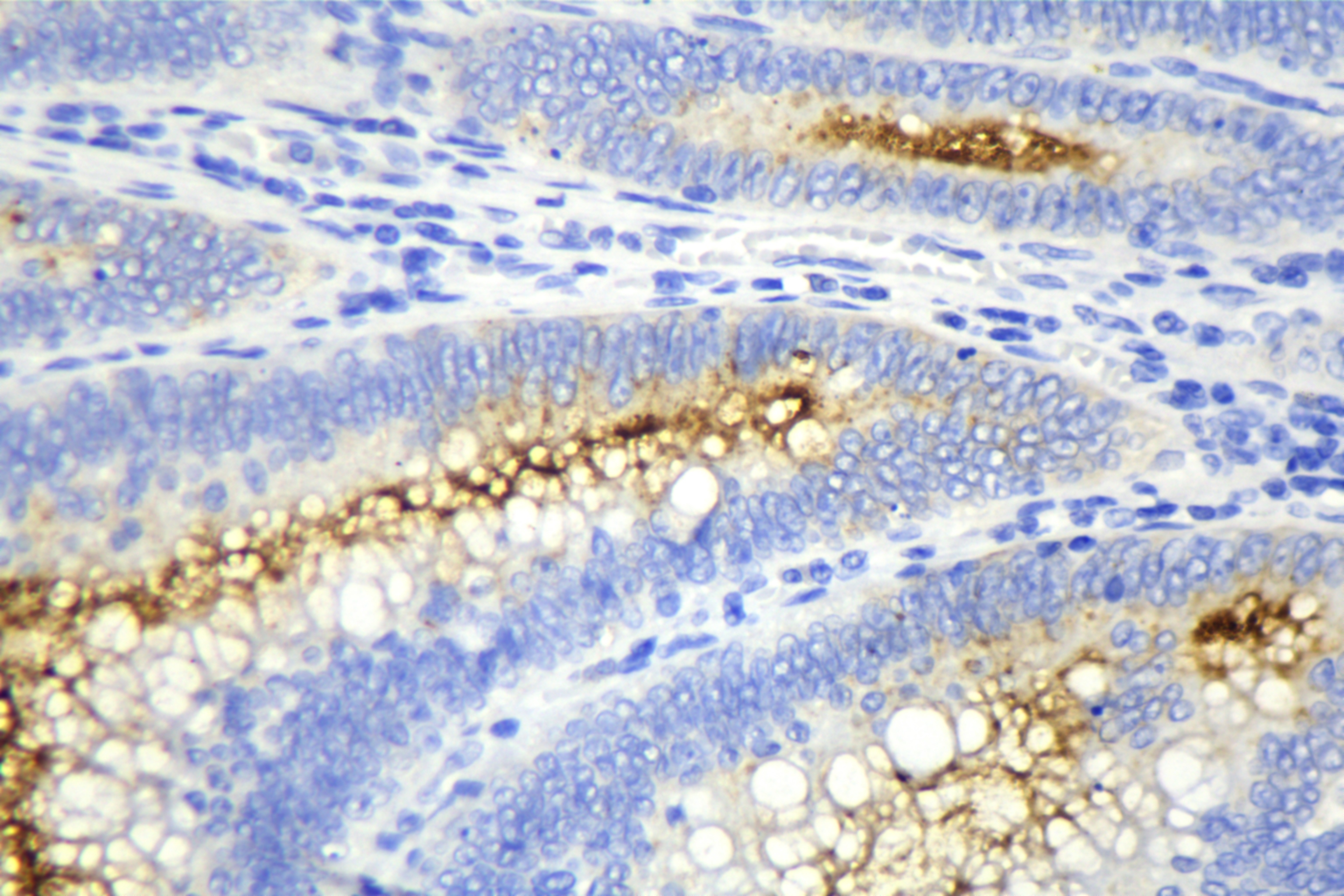

Supplement: Supplementary file 3 — Source data Fig. 1 [file 44321_2025_308_MOESM3_ESM.zip › Figure 1/h/S24-2698A6 40X.jpg]

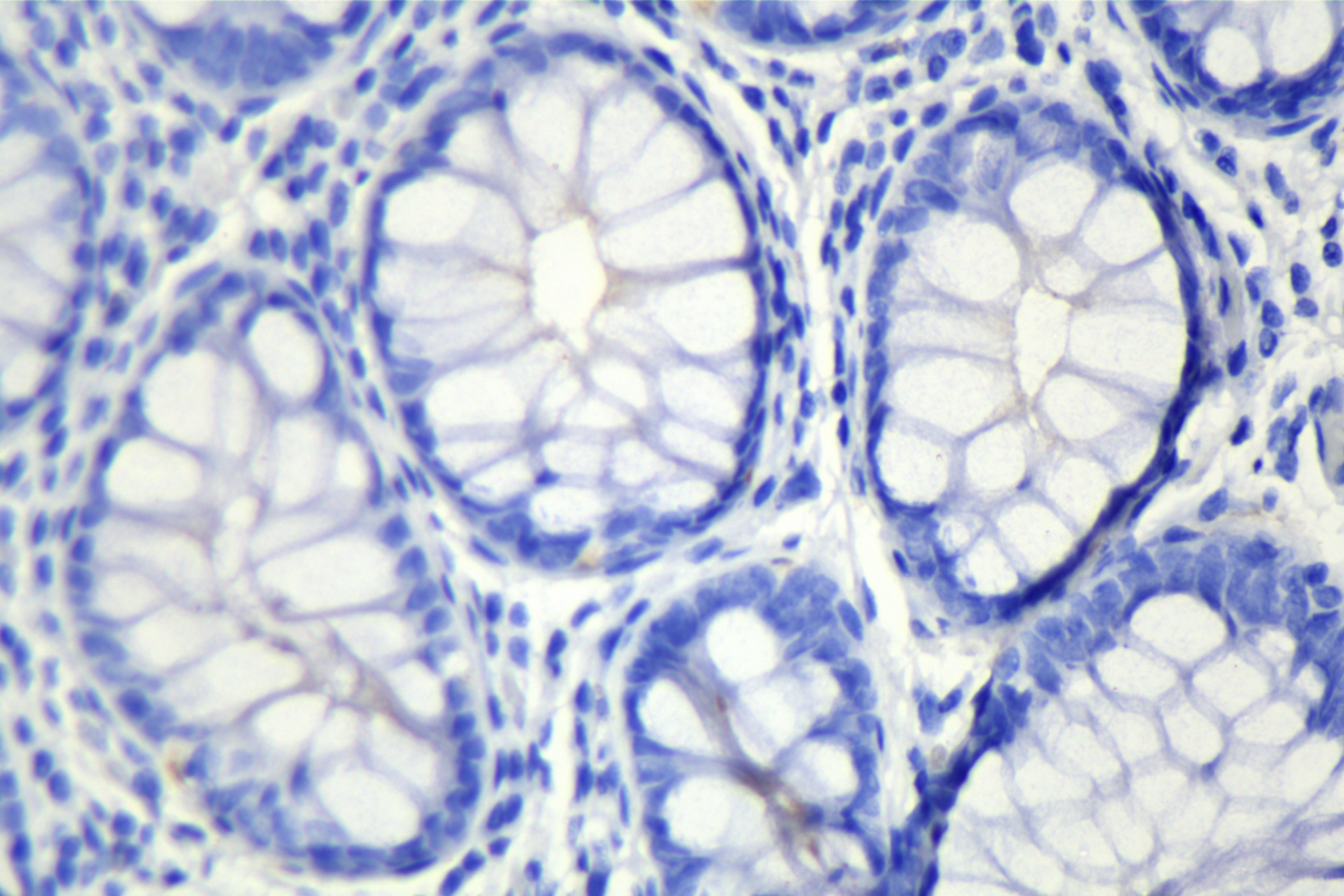

Supplement: Supplementary file 3 — Source data Fig. 1 [file 44321_2025_308_MOESM3_ESM.zip › Figure 1/h/S24-2698A8 40X.jpg]

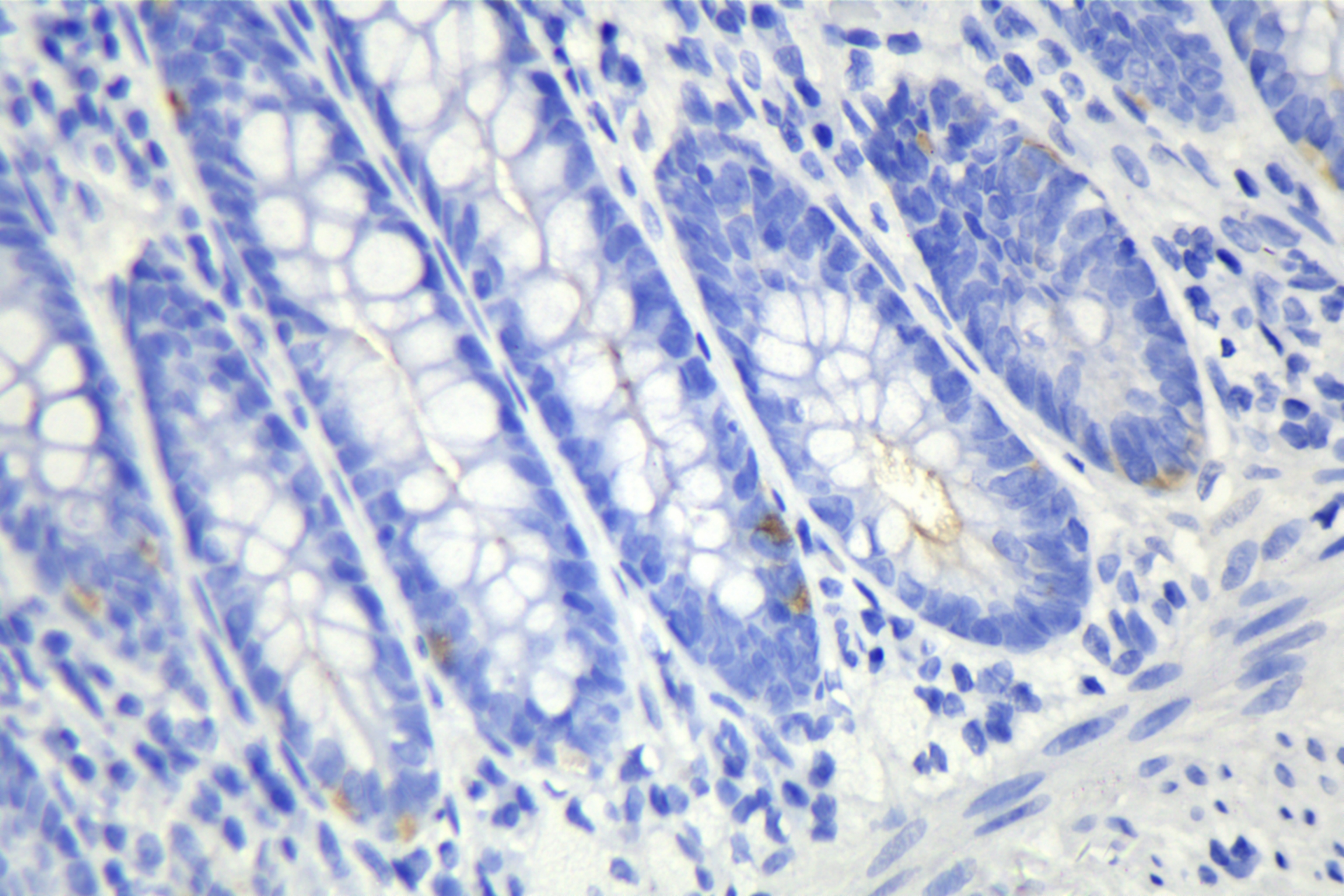

Supplement: Supplementary file 3 — Source data Fig. 1 [file 44321_2025_308_MOESM3_ESM.zip › Figure 1/h/S24-2708A8 40X 1.jpg]

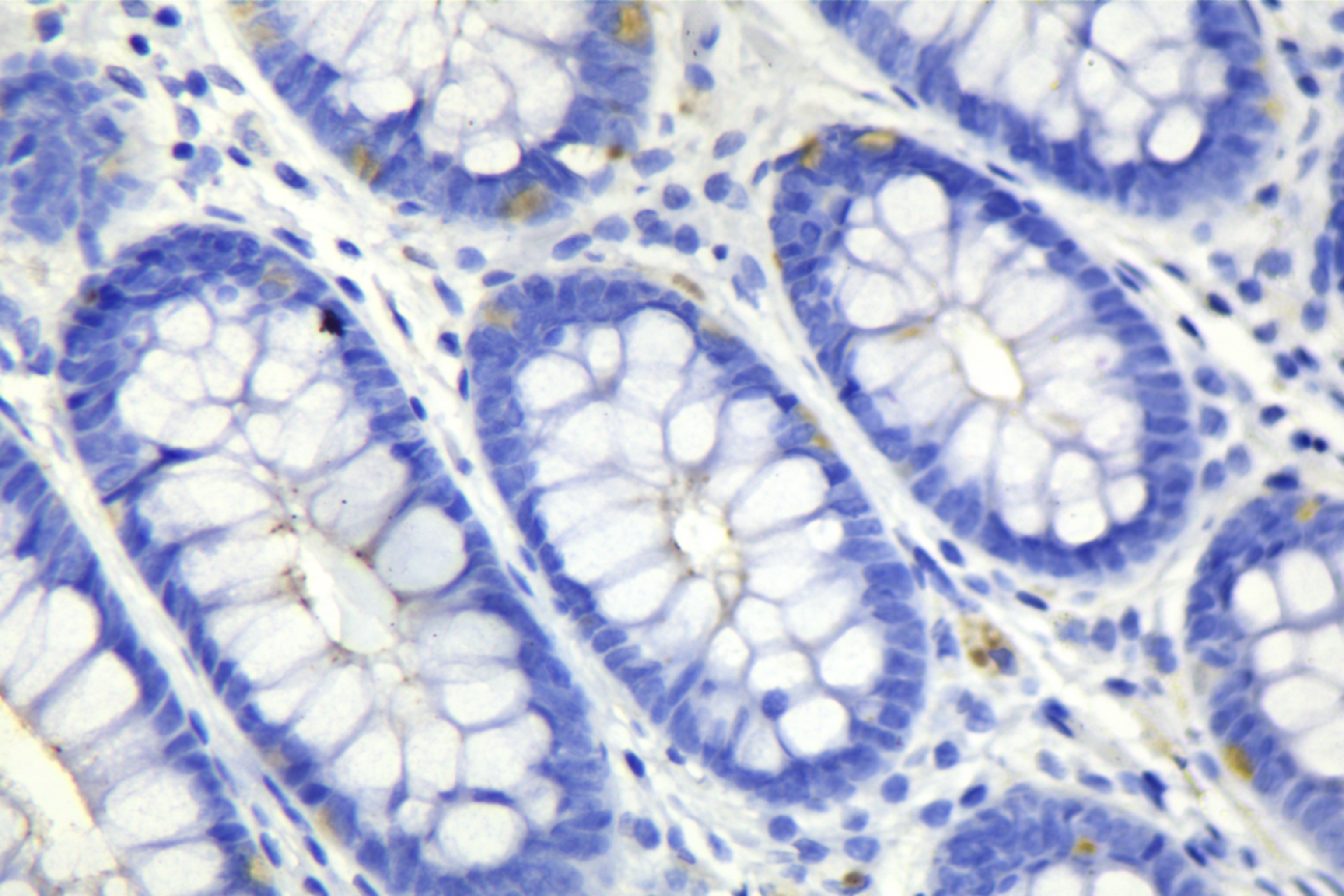

Supplement: Supplementary file 3 — Source data Fig. 1 [file 44321_2025_308_MOESM3_ESM.zip › Figure 1/h/S24-2892A6 40X 3.jpg]

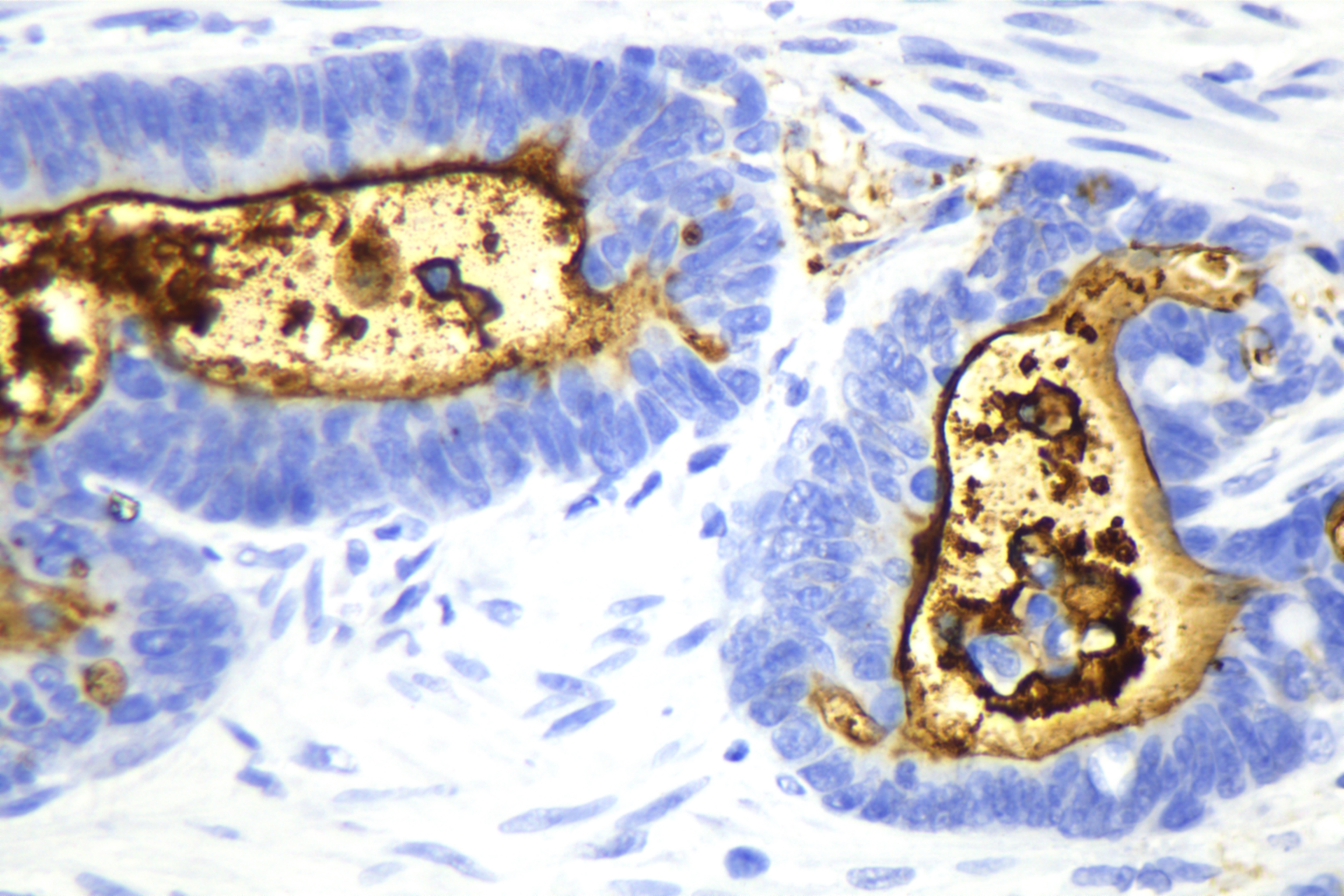

Supplement: Supplementary file 3 — Source data Fig. 1 [file 44321_2025_308_MOESM3_ESM.zip › Figure 1/h/S24-2892A8 40X 1.jpg]

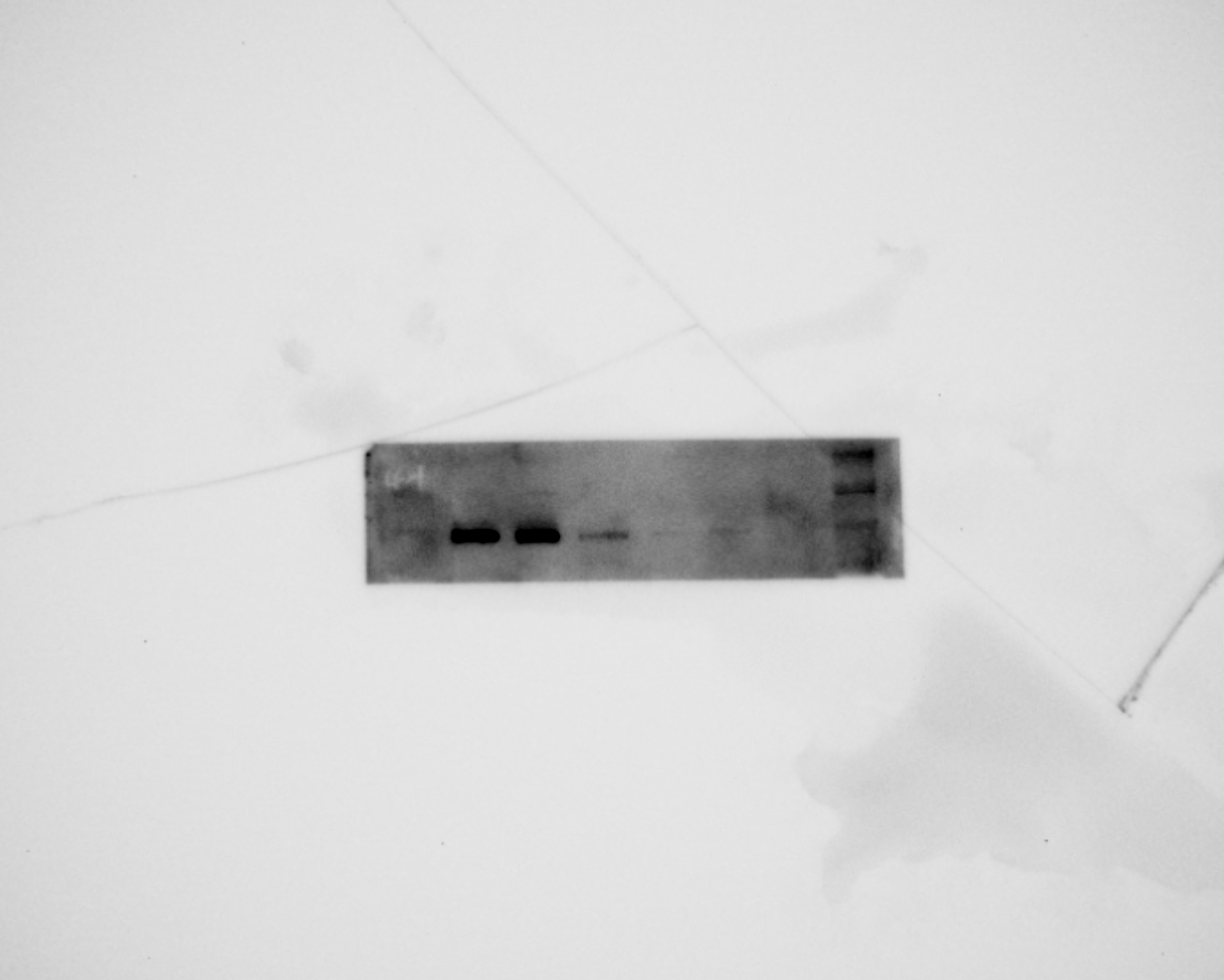

Supplement: Supplementary file 4 — Source data Fig. 2 [file 44321_2025_308_MOESM4_ESM.zip › Figure 2/2i, k/western CD133 DLD1 PP10.tif]

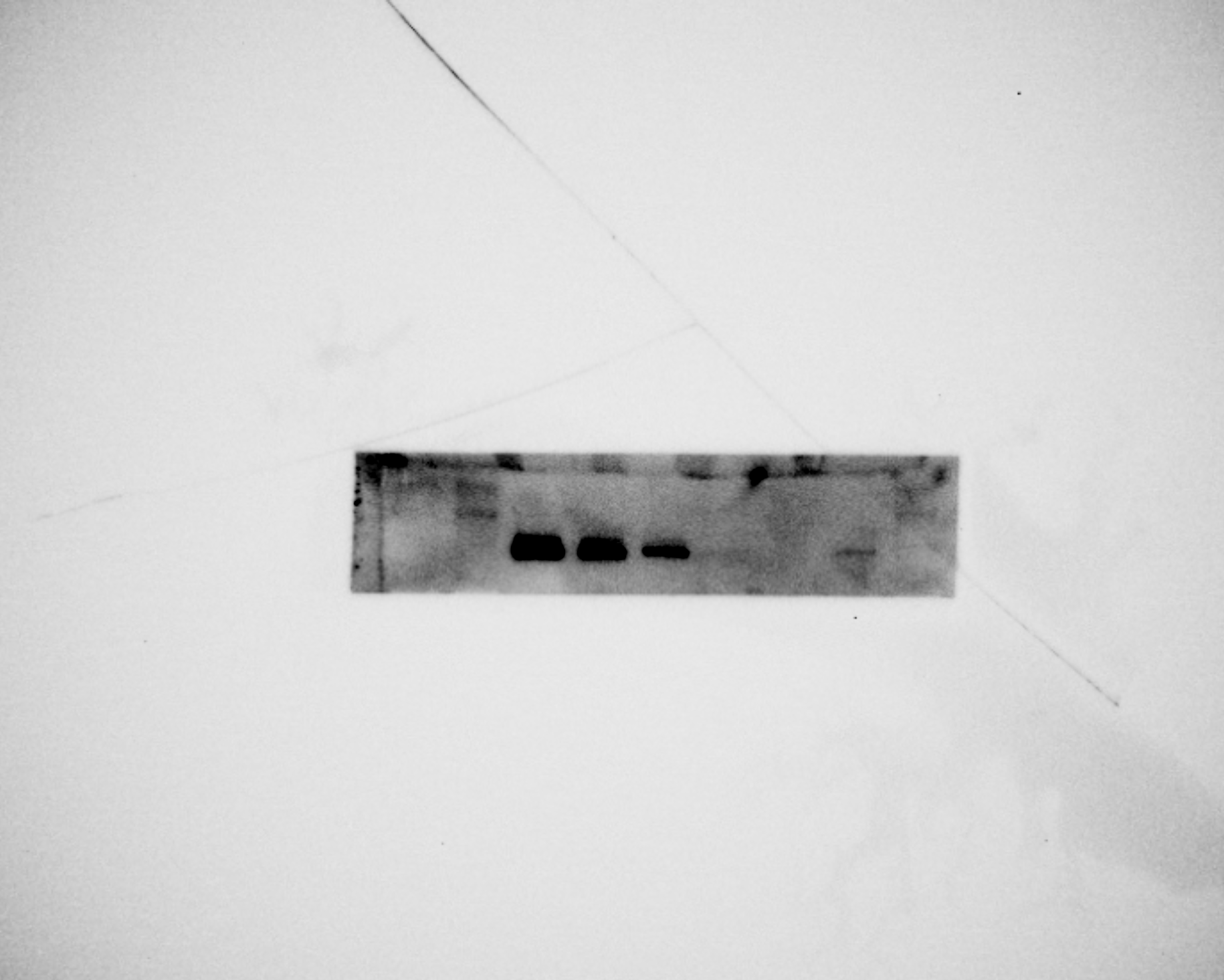

Supplement: Supplementary file 4 — Source data Fig. 2 [file 44321_2025_308_MOESM4_ESM.zip › Figure 2/2i, k/western CD133 HCT116 PP10.tif]

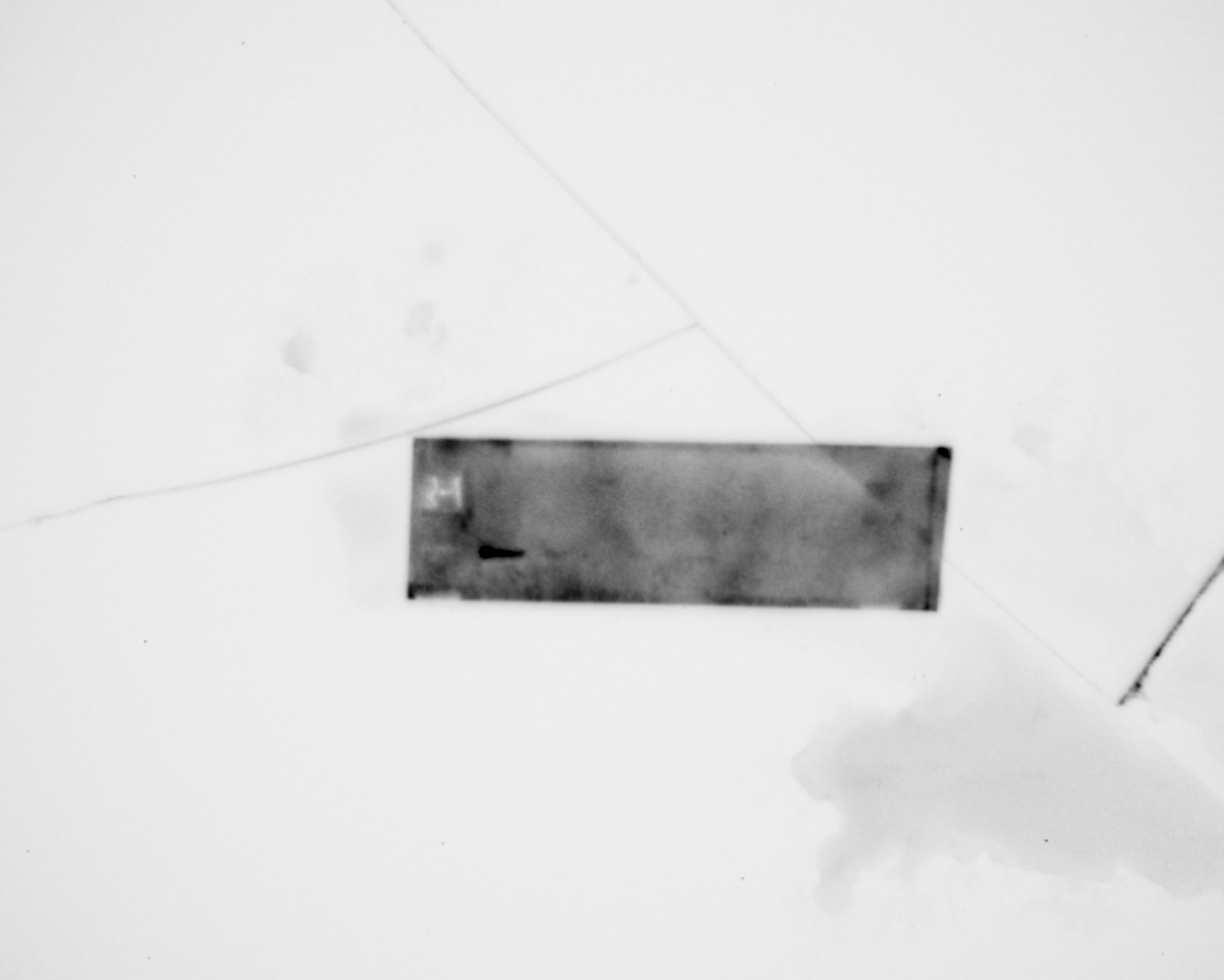

Supplement: Supplementary file 4 — Source data Fig. 2 [file 44321_2025_308_MOESM4_ESM.zip › Figure 2/2i, k/western CD133 DLD1 Con.tif]

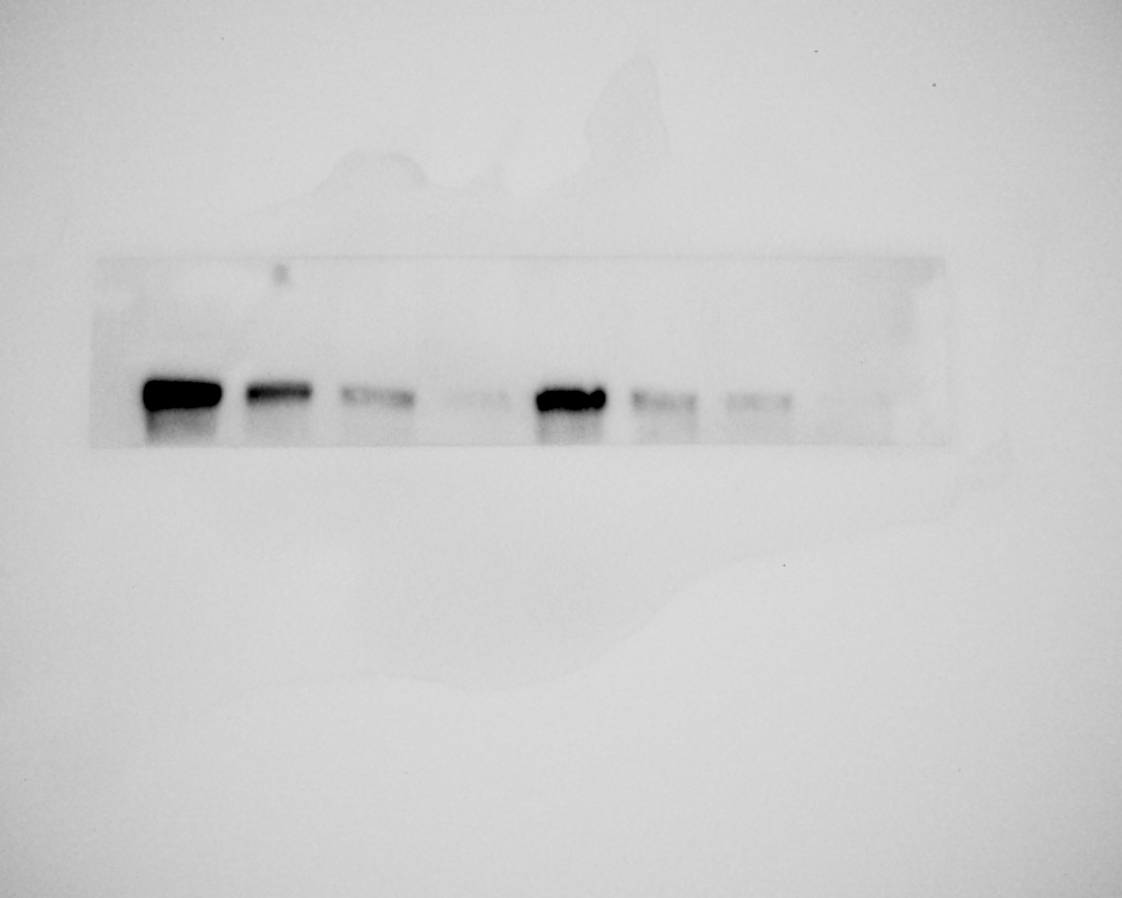

Supplement: Supplementary file 4 — Source data Fig. 2 [file 44321_2025_308_MOESM4_ESM.zip › Figure 2/2i, k/western CD133 DLD1 PP10 2.tif]

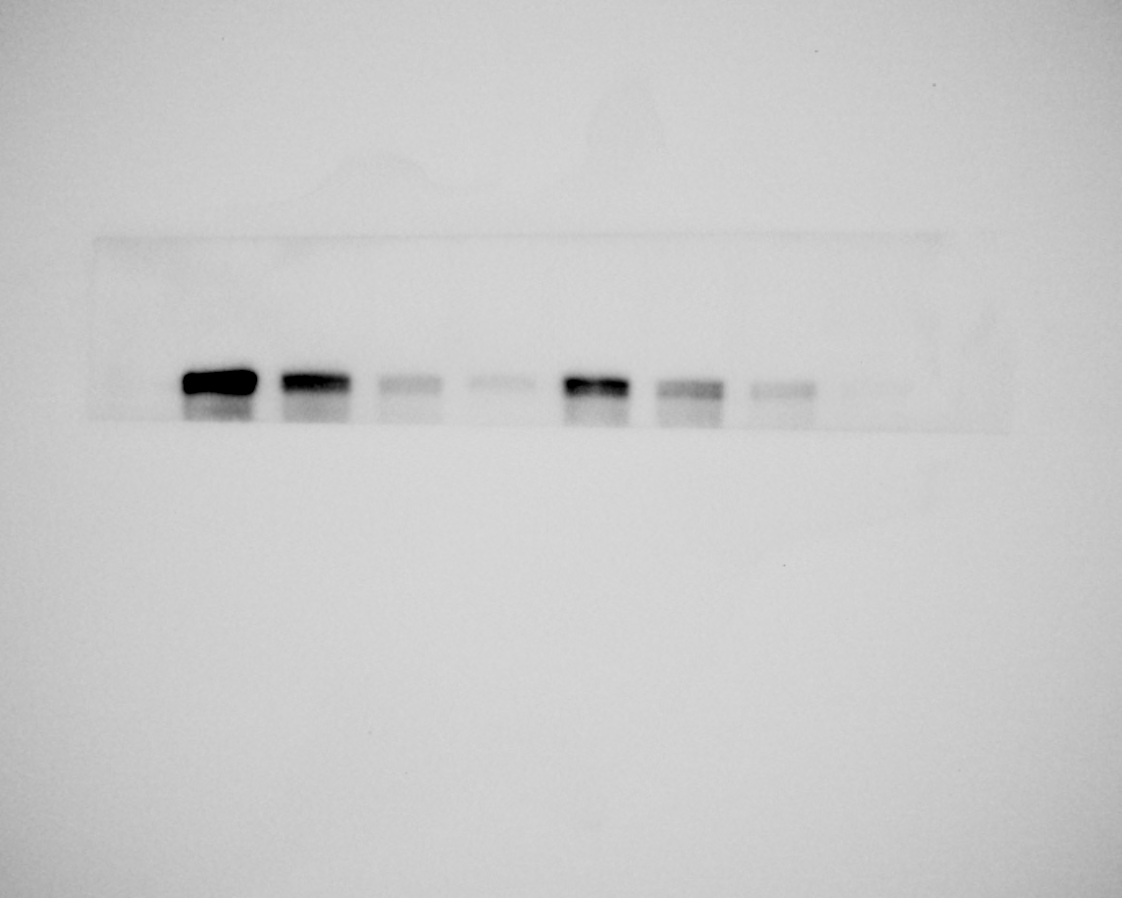

Supplement: Supplementary file 4 — Source data Fig. 2 [file 44321_2025_308_MOESM4_ESM.zip › Figure 2/2i, k/western CD133 DLD1 PP10 3.tif]

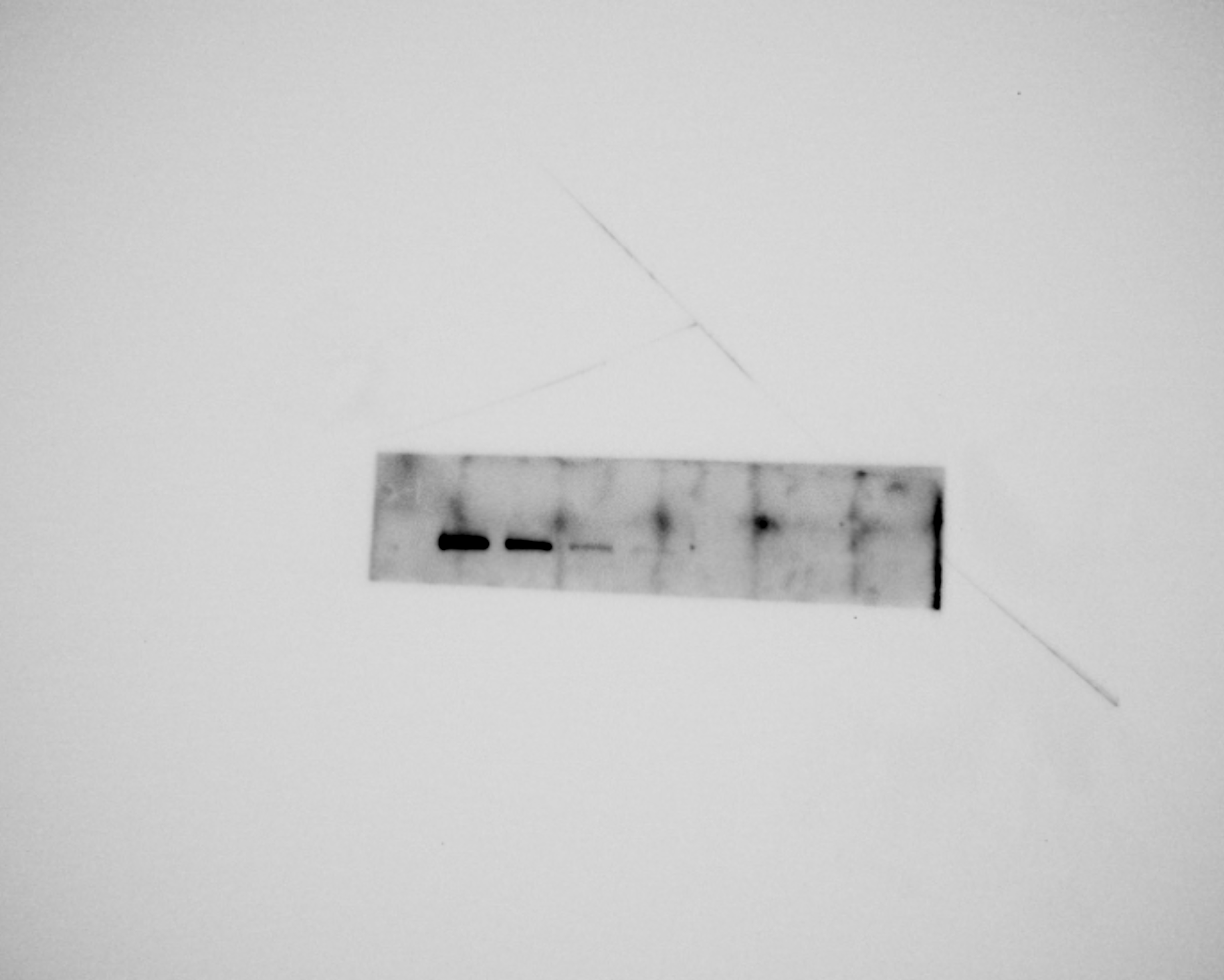

Supplement: Supplementary file 4 — Source data Fig. 2 [file 44321_2025_308_MOESM4_ESM.zip › Figure 2/2i, k/western CD133 HCT116 Con.tif]

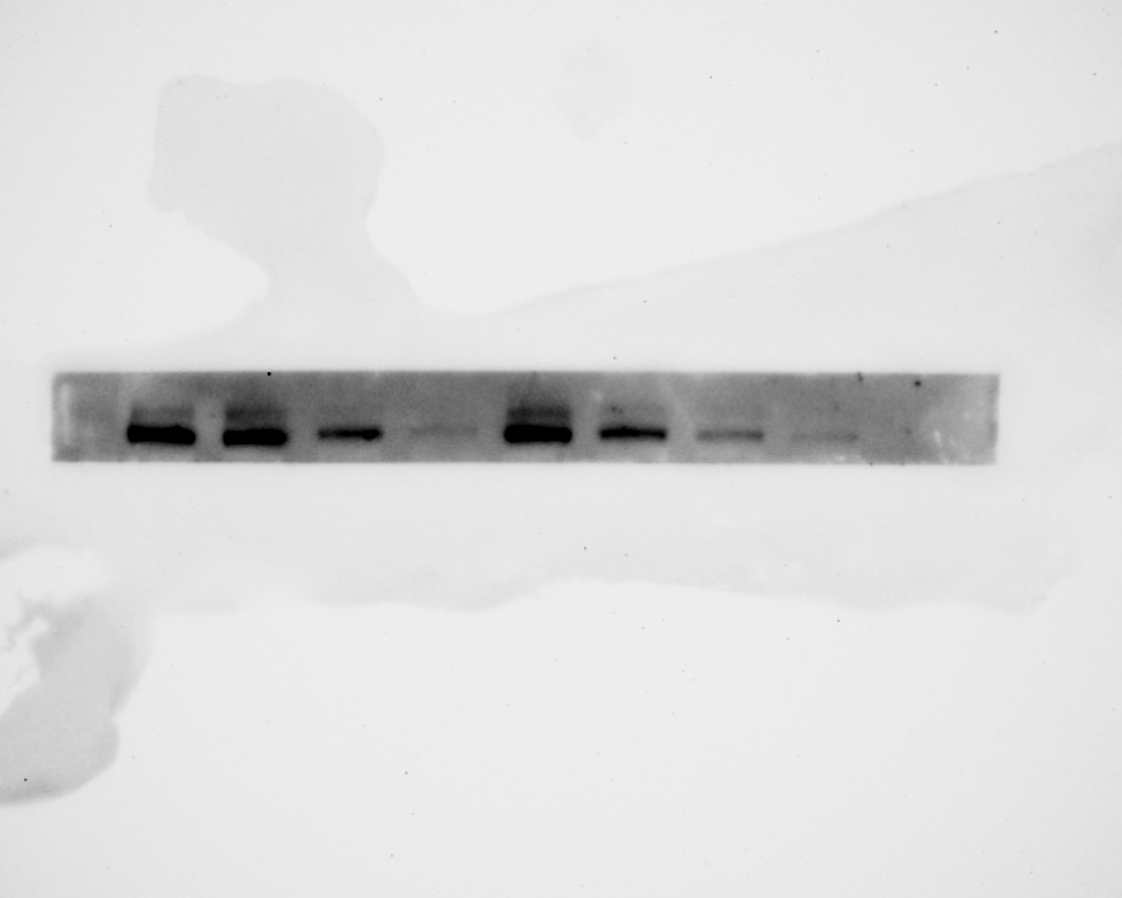

Supplement: Supplementary file 4 — Source data Fig. 2 [file 44321_2025_308_MOESM4_ESM.zip › Figure 2/2i, k/western CD133 HCT116 PP10 2.tif]

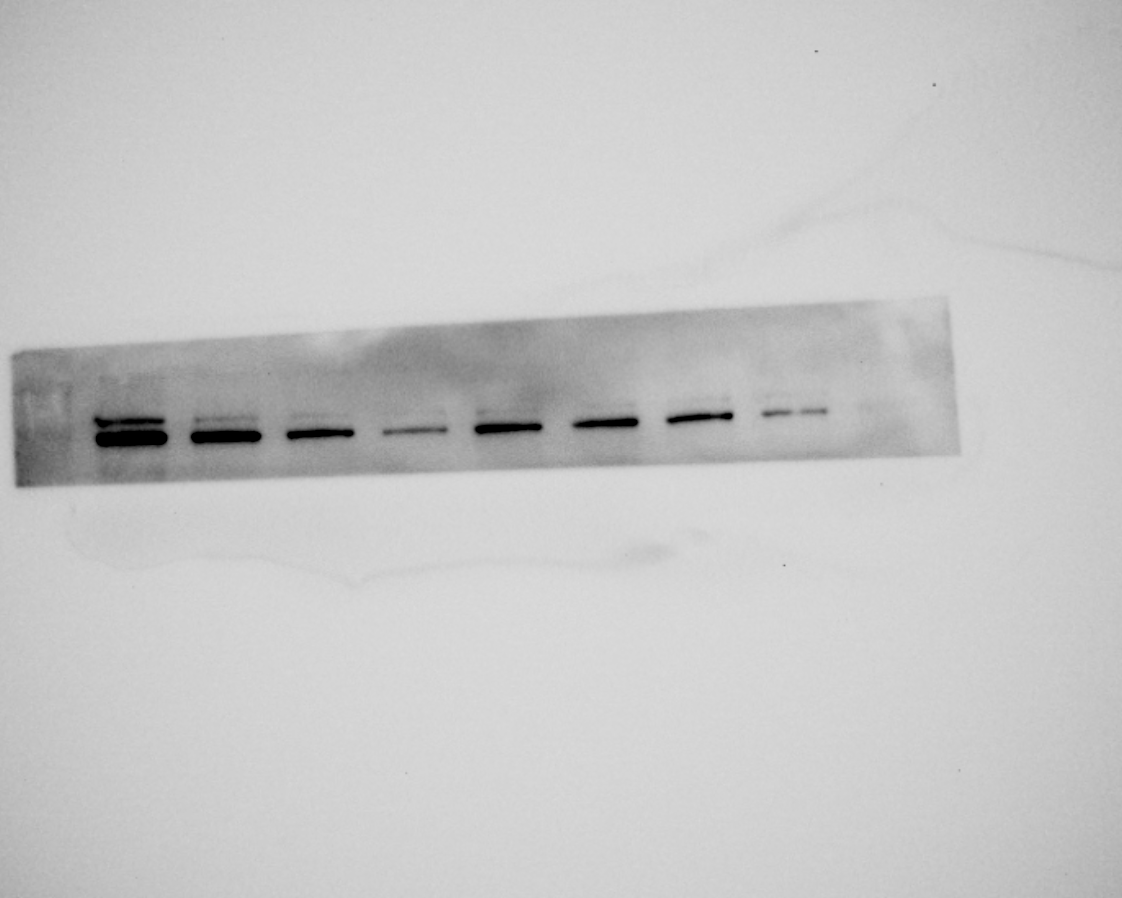

Supplement: Supplementary file 4 — Source data Fig. 2 [file 44321_2025_308_MOESM4_ESM.zip › Figure 2/2j, l/western CD133 DLD1 PP24 1.tif]

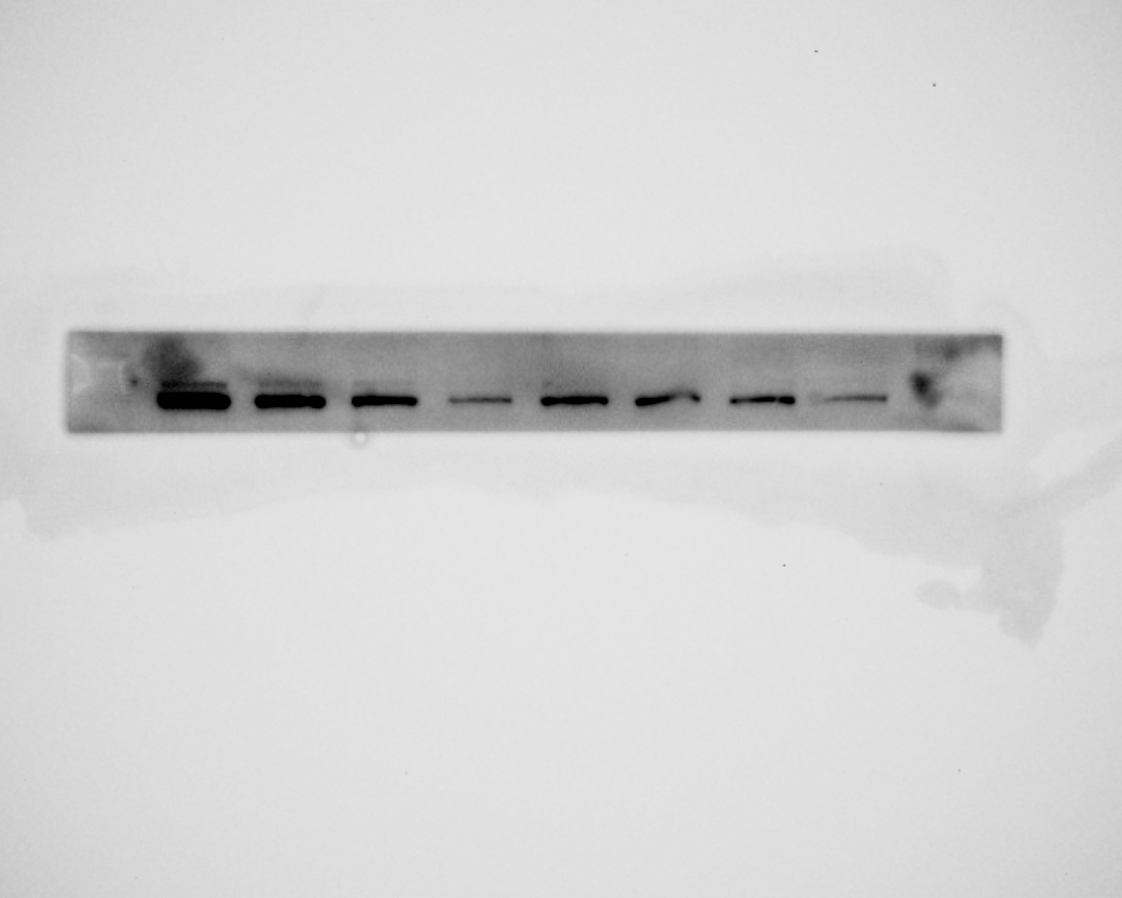

Supplement: Supplementary file 4 — Source data Fig. 2 [file 44321_2025_308_MOESM4_ESM.zip › Figure 2/2j, l/western CD133 DLD1 PP24 2.tif]

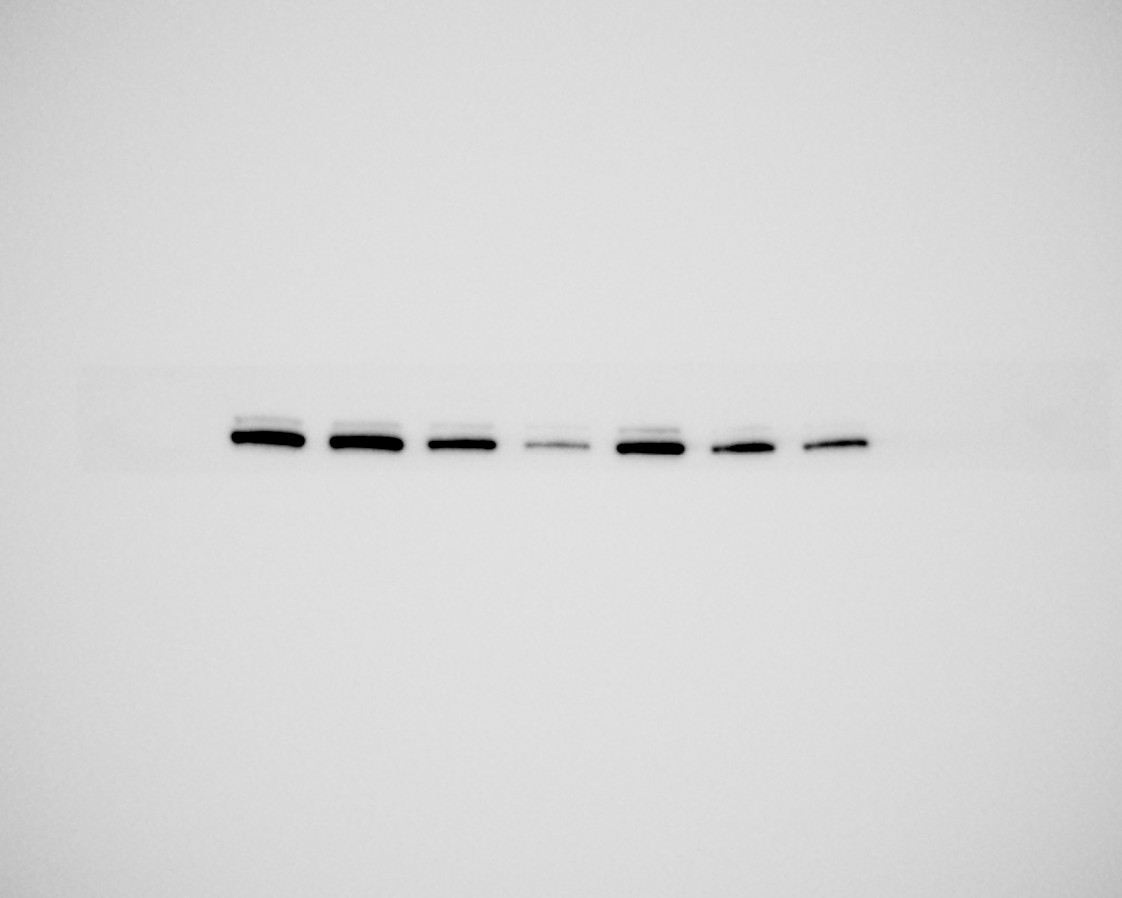

Supplement: Supplementary file 4 — Source data Fig. 2 [file 44321_2025_308_MOESM4_ESM.zip › Figure 2/2j, l/western CD133 HCT116 PP24 1.tif]

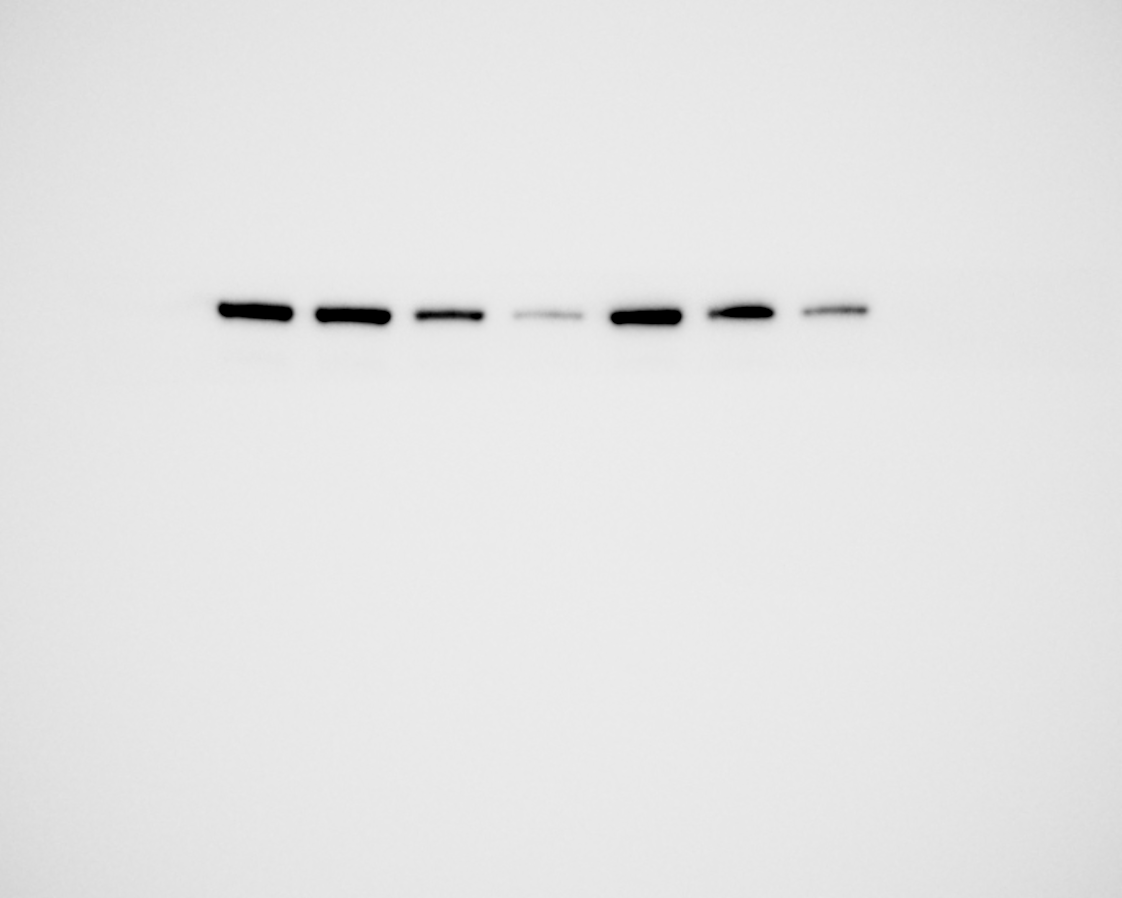

Supplement: Supplementary file 4 — Source data Fig. 2 [file 44321_2025_308_MOESM4_ESM.zip › Figure 2/2j, l/western CD133 HCT116 PP24 2.tif]

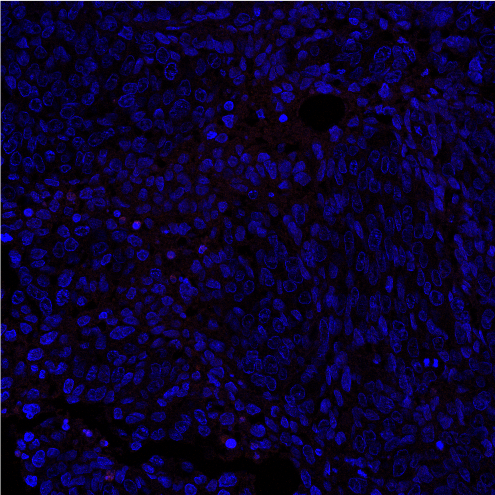

Supplement: Supplementary file 5 — Source data Fig. 3 [file 44321_2025_308_MOESM5_ESM.zip › Figure 3/3f, g/con-11.png]

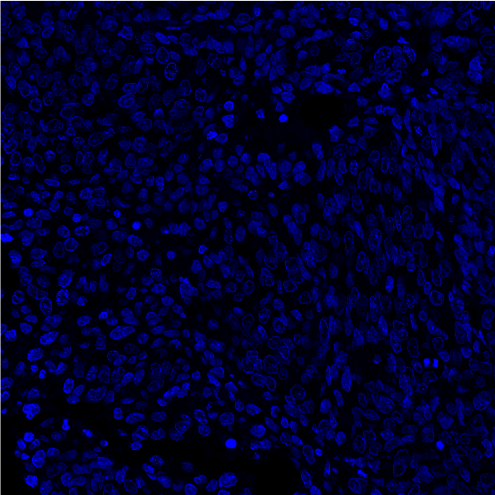

Supplement: Supplementary file 5 — Source data Fig. 3 [file 44321_2025_308_MOESM5_ESM.zip › Figure 3/3f, g/con-12.png]

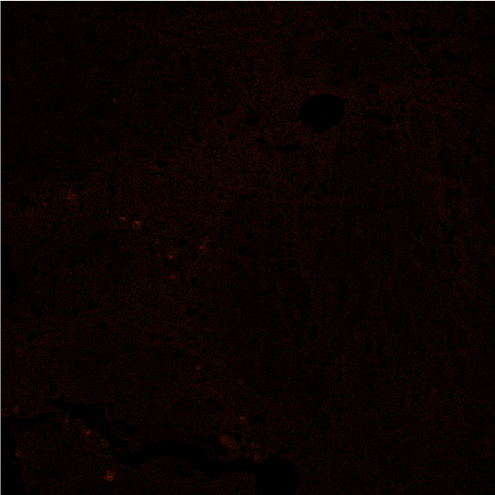

Supplement: Supplementary file 5 — Source data Fig. 3 [file 44321_2025_308_MOESM5_ESM.zip › Figure 3/3f, g/con-13.png]

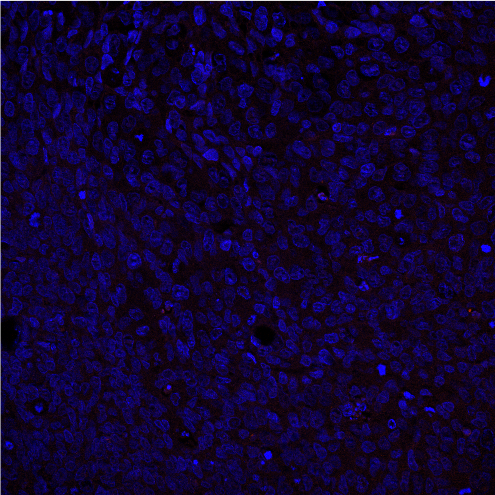

Supplement: Supplementary file 5 — Source data Fig. 3 [file 44321_2025_308_MOESM5_ESM.zip › Figure 3/3f, g/con-21.png]

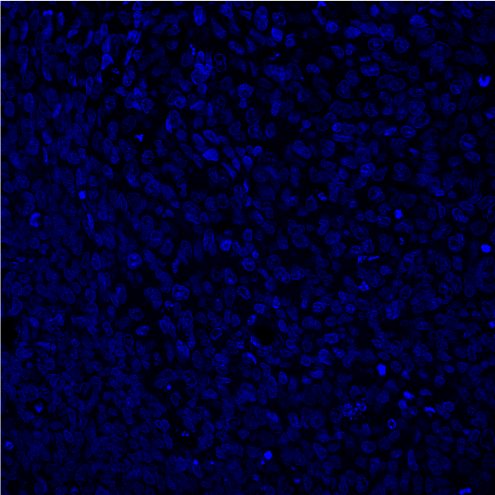

Supplement: Supplementary file 5 — Source data Fig. 3 [file 44321_2025_308_MOESM5_ESM.zip › Figure 3/3f, g/con-22.png]

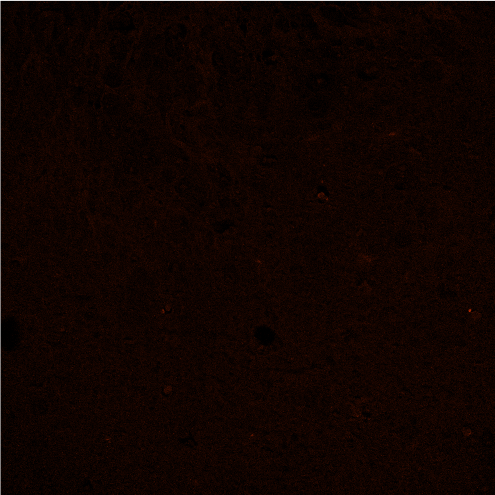

Supplement: Supplementary file 5 — Source data Fig. 3 [file 44321_2025_308_MOESM5_ESM.zip › Figure 3/3f, g/con-23.png]

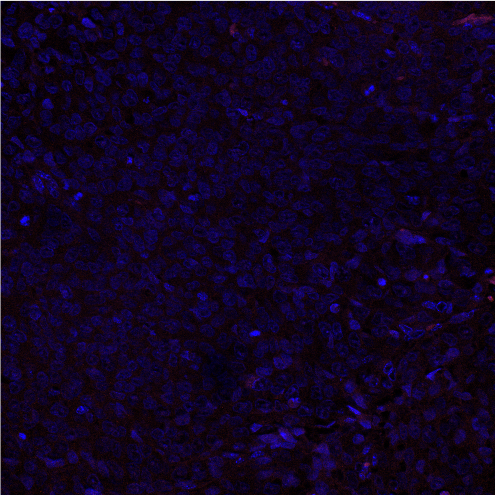

Supplement: Supplementary file 5 — Source data Fig. 3 [file 44321_2025_308_MOESM5_ESM.zip › Figure 3/3f, g/con-31.png]

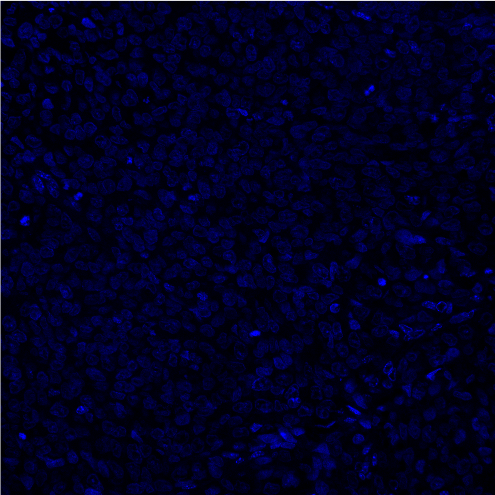

Supplement: Supplementary file 5 — Source data Fig. 3 [file 44321_2025_308_MOESM5_ESM.zip › Figure 3/3f, g/con-32.png]

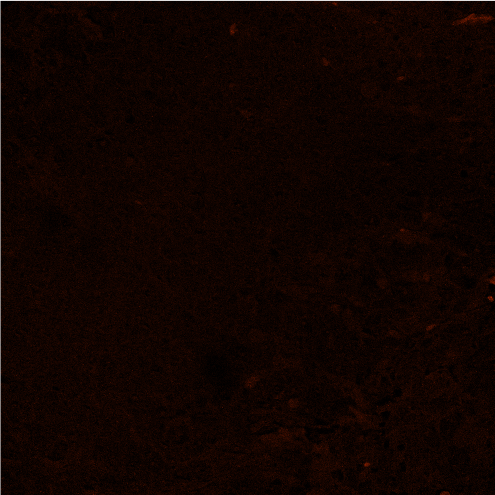

Supplement: Supplementary file 5 — Source data Fig. 3 [file 44321_2025_308_MOESM5_ESM.zip › Figure 3/3f, g/con-33.png]

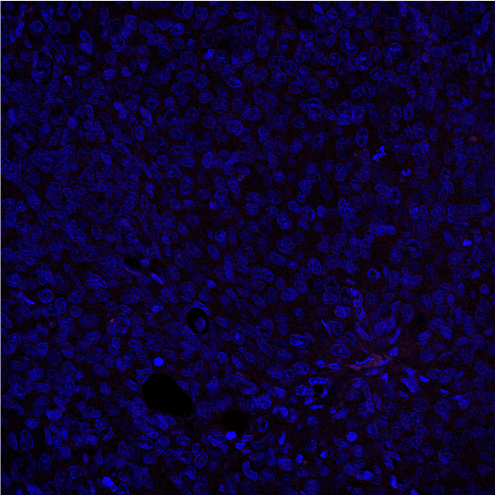

Supplement: Supplementary file 5 — Source data Fig. 3 [file 44321_2025_308_MOESM5_ESM.zip › Figure 3/3f, g/con-41.png]

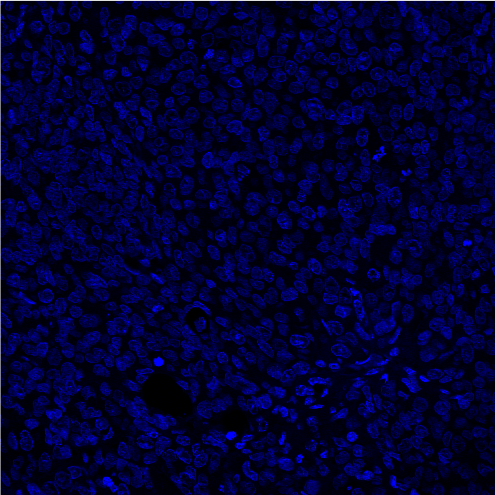

Supplement: Supplementary file 5 — Source data Fig. 3 [file 44321_2025_308_MOESM5_ESM.zip › Figure 3/3f, g/con-42.png]

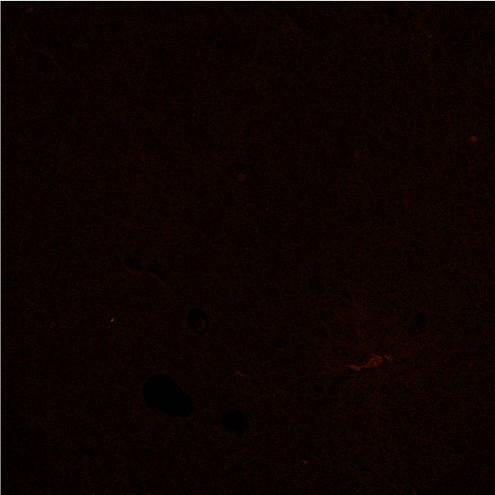

Supplement: Supplementary file 5 — Source data Fig. 3 [file 44321_2025_308_MOESM5_ESM.zip › Figure 3/3f, g/con-43.png]

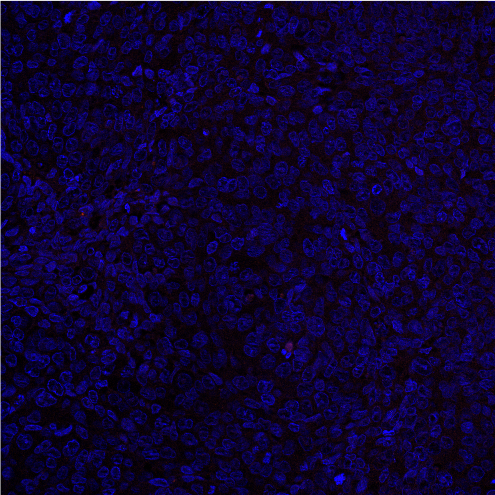

Supplement: Supplementary file 5 — Source data Fig. 3 [file 44321_2025_308_MOESM5_ESM.zip › Figure 3/3f, g/con-51.png]

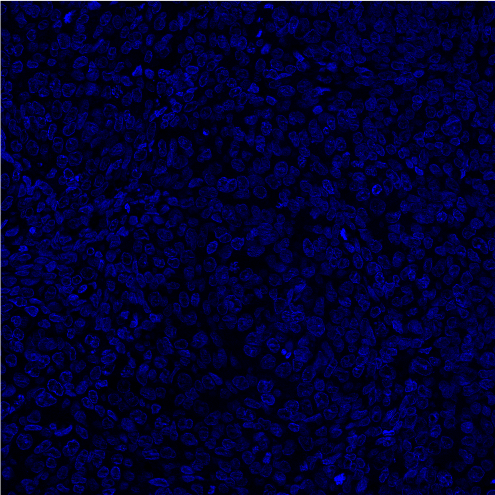

Supplement: Supplementary file 5 — Source data Fig. 3 [file 44321_2025_308_MOESM5_ESM.zip › Figure 3/3f, g/con-52.png]

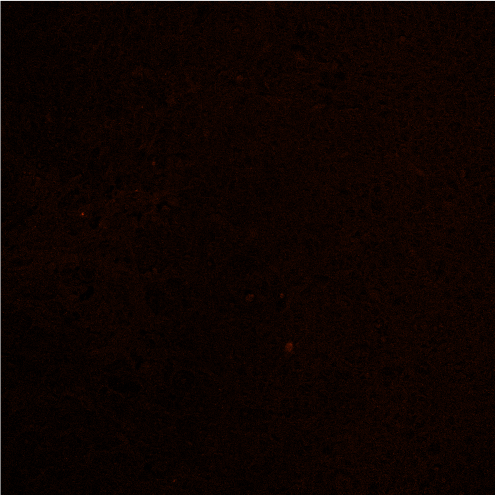

Supplement: Supplementary file 5 — Source data Fig. 3 [file 44321_2025_308_MOESM5_ESM.zip › Figure 3/3f, g/con-53.png]

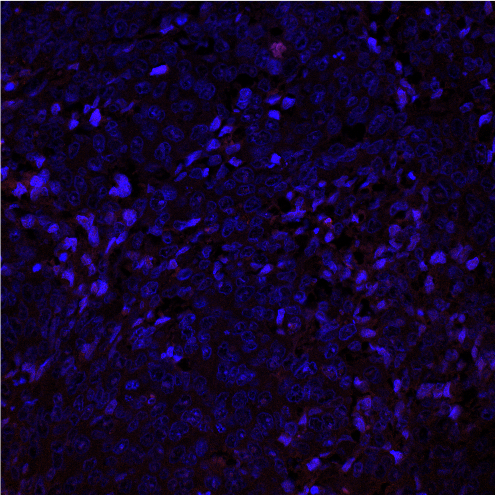

Supplement: Supplementary file 5 — Source data Fig. 3 [file 44321_2025_308_MOESM5_ESM.zip › Figure 3/3f, g/OX-11.png]

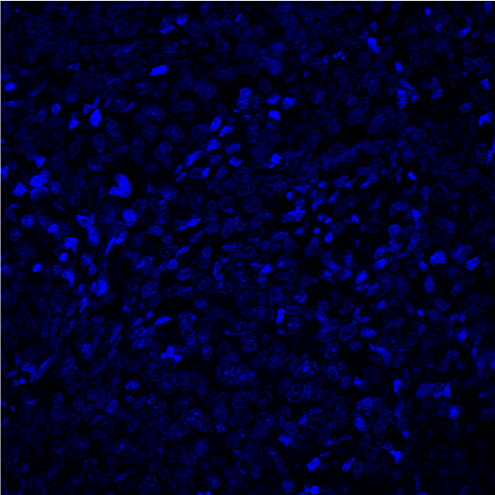

Supplement: Supplementary file 5 — Source data Fig. 3 [file 44321_2025_308_MOESM5_ESM.zip › Figure 3/3f, g/OX-12.png]

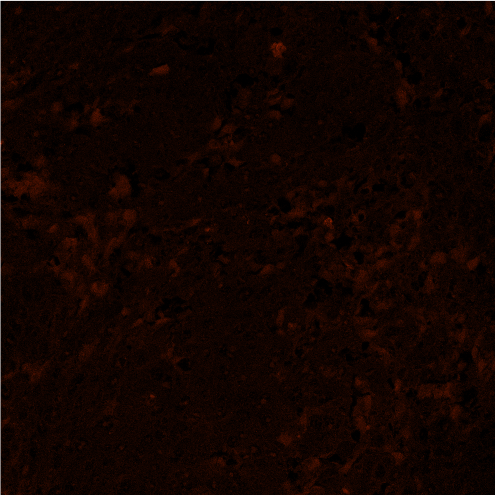

Supplement: Supplementary file 5 — Source data Fig. 3 [file 44321_2025_308_MOESM5_ESM.zip › Figure 3/3f, g/OX-13.png]

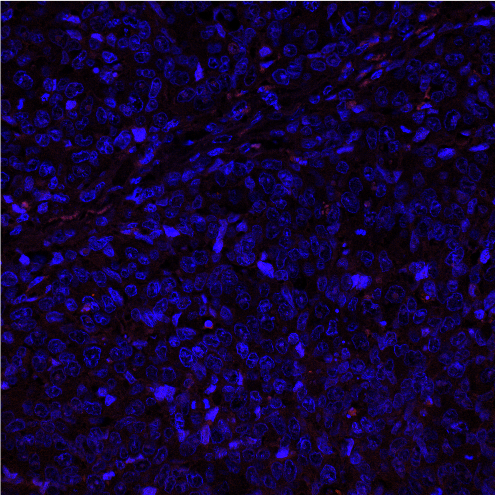

Supplement: Supplementary file 5 — Source data Fig. 3 [file 44321_2025_308_MOESM5_ESM.zip › Figure 3/3f, g/OX-21.png]

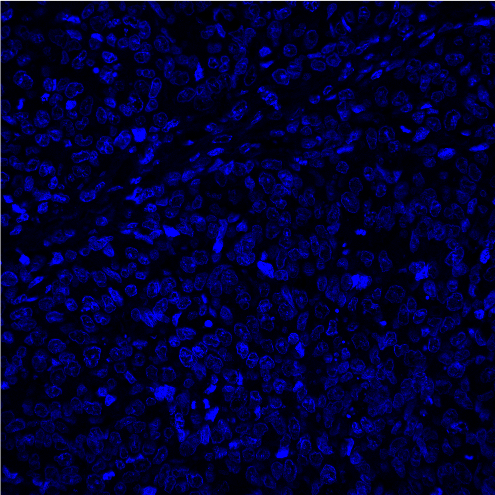

Supplement: Supplementary file 5 — Source data Fig. 3 [file 44321_2025_308_MOESM5_ESM.zip › Figure 3/3f, g/OX-22.png]

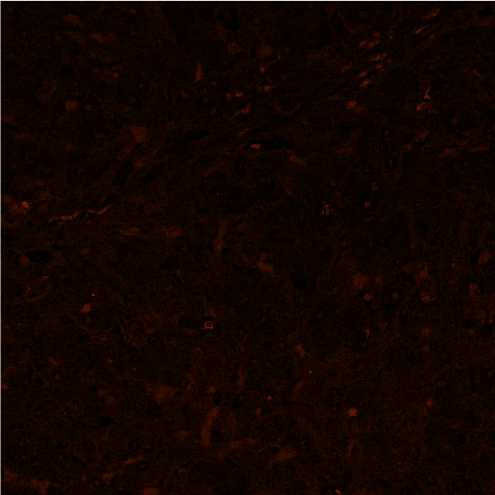

Supplement: Supplementary file 5 — Source data Fig. 3 [file 44321_2025_308_MOESM5_ESM.zip › Figure 3/3f, g/OX-23.png]

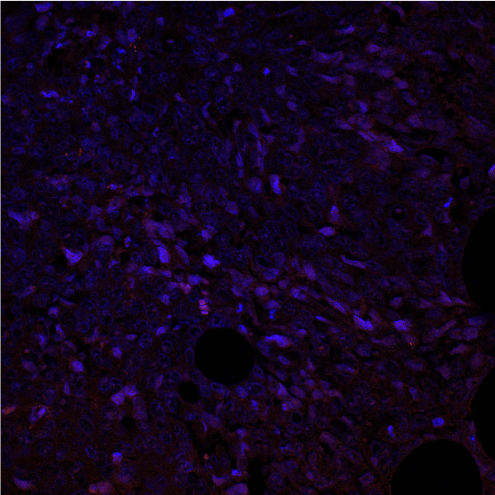

Supplement: Supplementary file 5 — Source data Fig. 3 [file 44321_2025_308_MOESM5_ESM.zip › Figure 3/3f, g/OX-31.png]

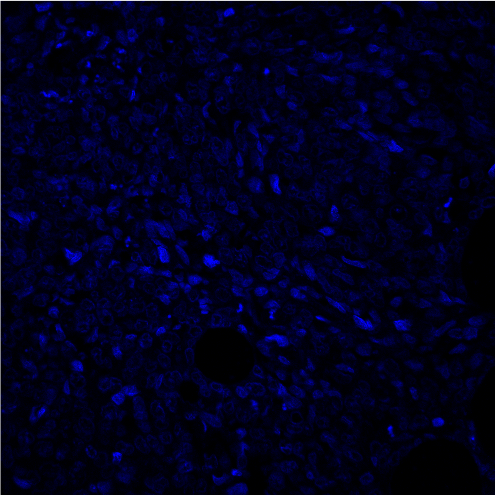

Supplement: Supplementary file 5 — Source data Fig. 3 [file 44321_2025_308_MOESM5_ESM.zip › Figure 3/3f, g/OX-32.png]

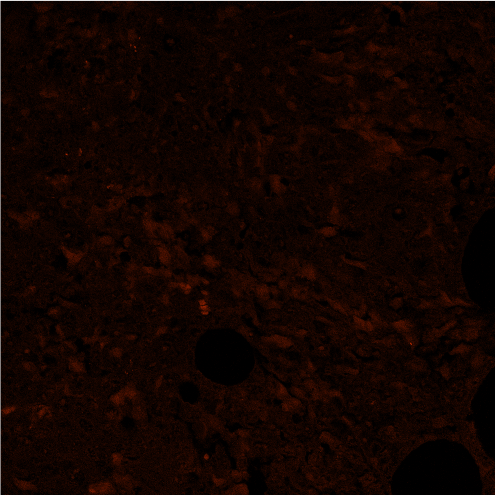

Supplement: Supplementary file 5 — Source data Fig. 3 [file 44321_2025_308_MOESM5_ESM.zip › Figure 3/3f, g/OX-33.png]

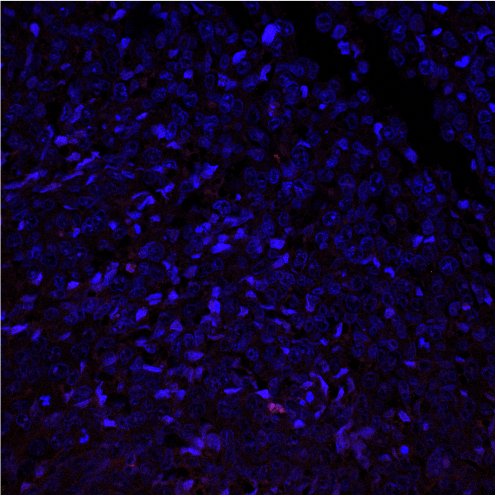

Supplement: Supplementary file 5 — Source data Fig. 3 [file 44321_2025_308_MOESM5_ESM.zip › Figure 3/3f, g/OX-41.png]

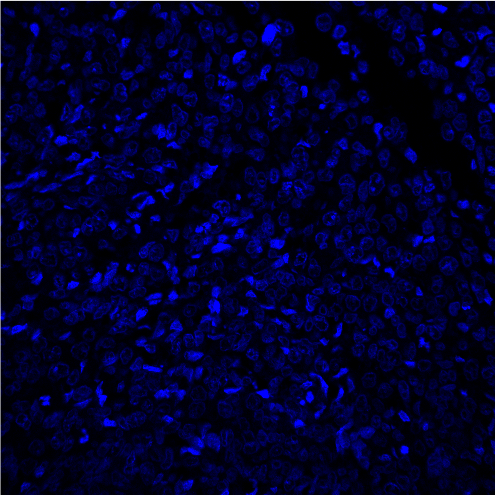

Supplement: Supplementary file 5 — Source data Fig. 3 [file 44321_2025_308_MOESM5_ESM.zip › Figure 3/3f, g/OX-42.png]

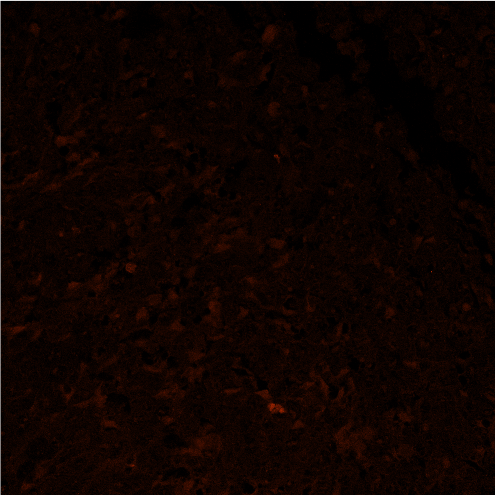

Supplement: Supplementary file 5 — Source data Fig. 3 [file 44321_2025_308_MOESM5_ESM.zip › Figure 3/3f, g/OX-43.png]

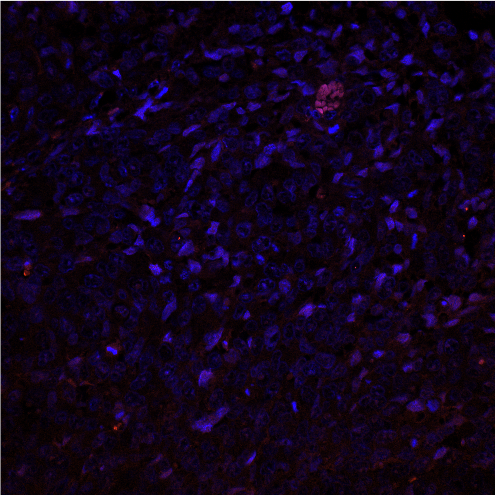

Supplement: Supplementary file 5 — Source data Fig. 3 [file 44321_2025_308_MOESM5_ESM.zip › Figure 3/3f, g/OX-51.png]

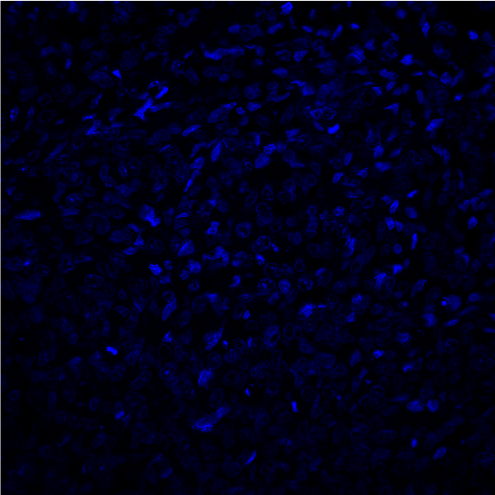

Supplement: Supplementary file 5 — Source data Fig. 3 [file 44321_2025_308_MOESM5_ESM.zip › Figure 3/3f, g/OX-52.png]

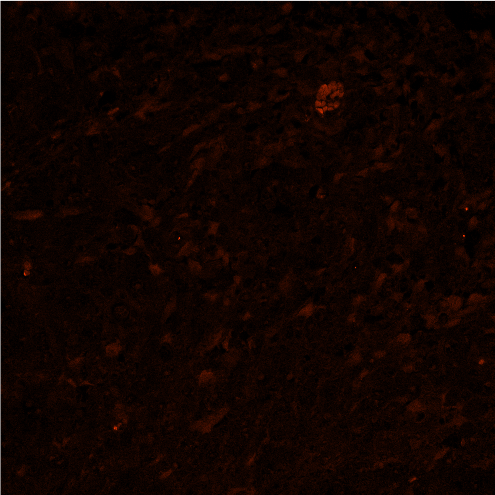

Supplement: Supplementary file 5 — Source data Fig. 3 [file 44321_2025_308_MOESM5_ESM.zip › Figure 3/3f, g/OX-53.png]

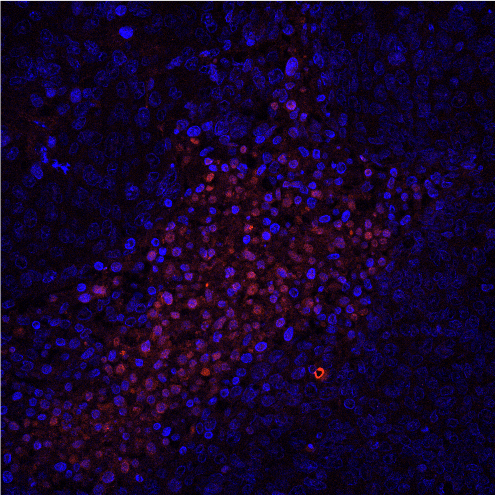

Supplement: Supplementary file 5 — Source data Fig. 3 [file 44321_2025_308_MOESM5_ESM.zip › Figure 3/3f, g/PP10 2.5-11.png]

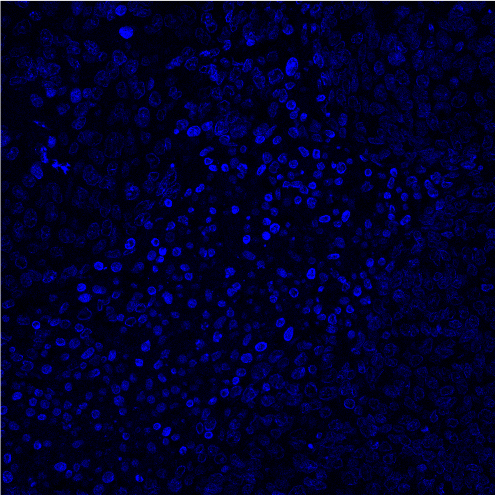

Supplement: Supplementary file 5 — Source data Fig. 3 [file 44321_2025_308_MOESM5_ESM.zip › Figure 3/3f, g/PP10 2.5-12.png]

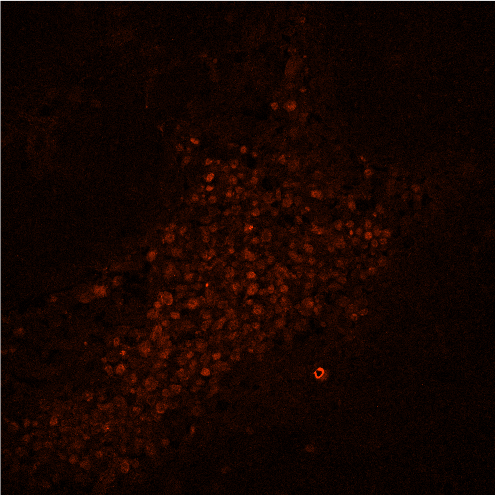

Supplement: Supplementary file 5 — Source data Fig. 3 [file 44321_2025_308_MOESM5_ESM.zip › Figure 3/3f, g/PP10 2.5-13.png]

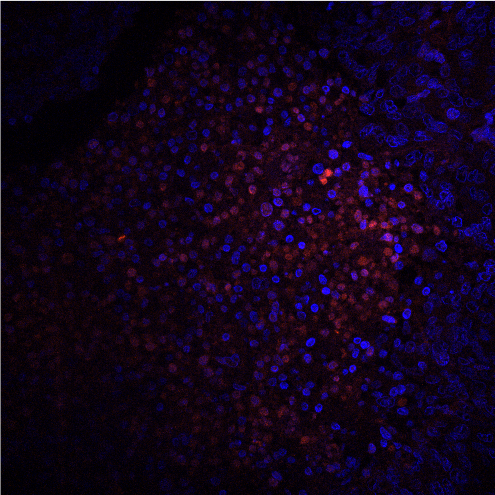

Supplement: Supplementary file 5 — Source data Fig. 3 [file 44321_2025_308_MOESM5_ESM.zip › Figure 3/3f, g/PP10 2.5-21.png]

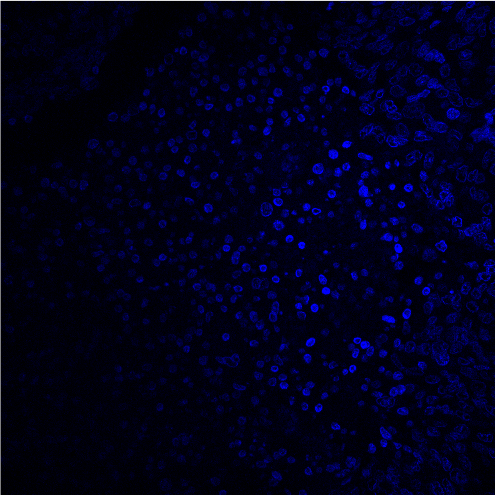

Supplement: Supplementary file 5 — Source data Fig. 3 [file 44321_2025_308_MOESM5_ESM.zip › Figure 3/3f, g/PP10 2.5-22.png]

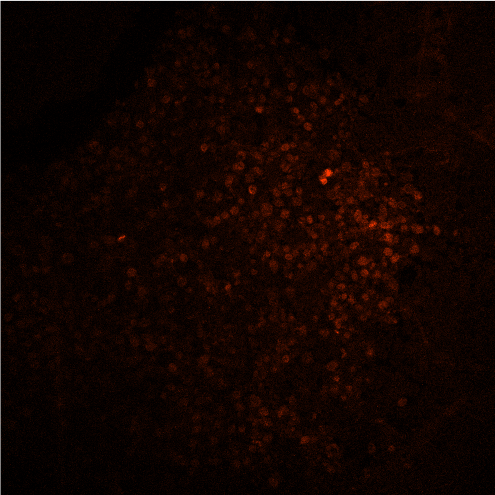

Supplement: Supplementary file 5 — Source data Fig. 3 [file 44321_2025_308_MOESM5_ESM.zip › Figure 3/3f, g/PP10 2.5-23.png]

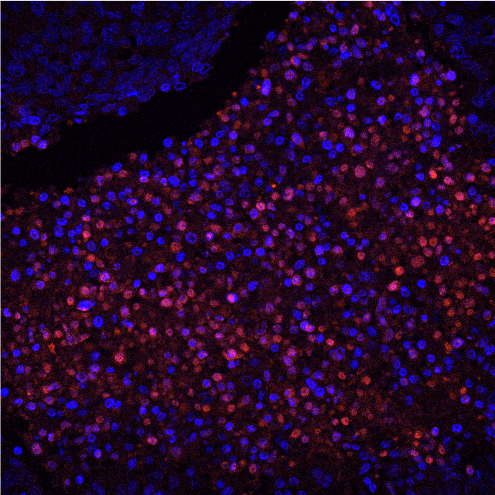

Supplement: Supplementary file 5 — Source data Fig. 3 [file 44321_2025_308_MOESM5_ESM.zip › Figure 3/3f, g/PP10 2.5-31.png]

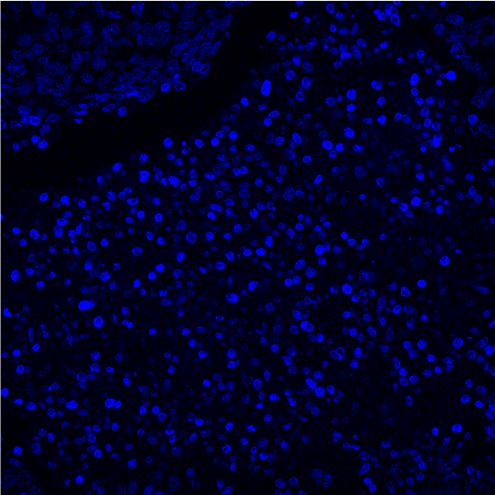

Supplement: Supplementary file 5 — Source data Fig. 3 [file 44321_2025_308_MOESM5_ESM.zip › Figure 3/3f, g/PP10 2.5-32.png]
