# Supplementary figures and images for: AI-identified CD133-targeting natural compounds demonstrate differential anti-tumor effects and mechanisms in pan-cancer models (part 2 of 4)
Source: EMBO Mol Med. 2025 Oct 2;17(11):2932–65. doi: 10.1038/s44321-025-00308-1 (PMC12603267; doi:10.1038/s44321-025-00308-1)

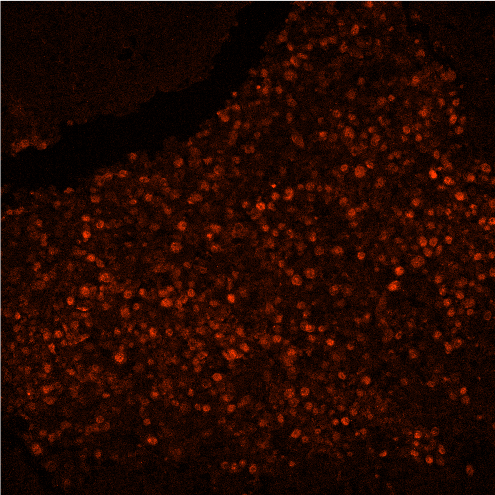

Supplement: Supplementary file 5 — Source data Fig. 3 [file 44321_2025_308_MOESM5_ESM.zip › Figure 3/3f, g/PP10 2.5-33.png]

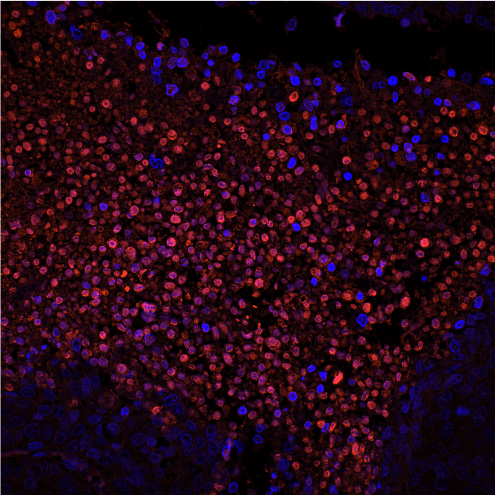

Supplement: Supplementary file 5 — Source data Fig. 3 [file 44321_2025_308_MOESM5_ESM.zip › Figure 3/3f, g/PP10 5-11.png]

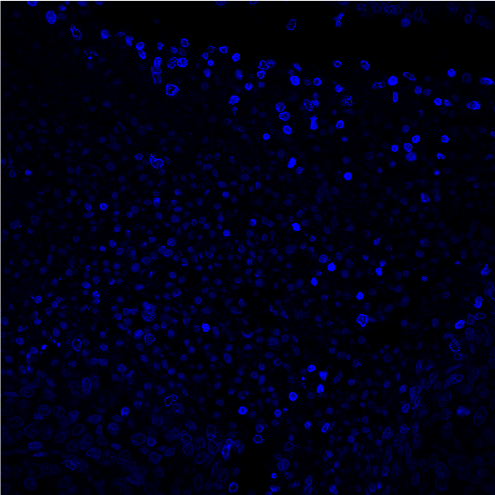

Supplement: Supplementary file 5 — Source data Fig. 3 [file 44321_2025_308_MOESM5_ESM.zip › Figure 3/3f, g/PP10 5-12.png]

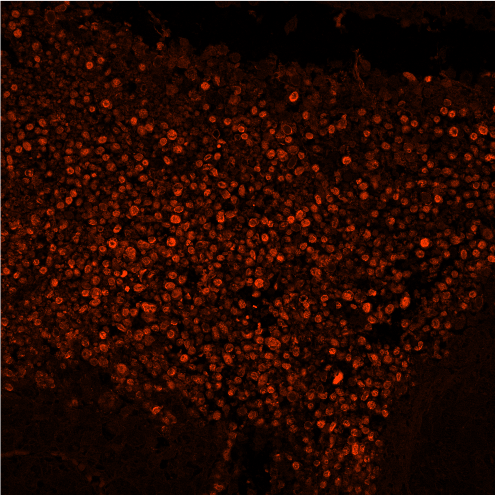

Supplement: Supplementary file 5 — Source data Fig. 3 [file 44321_2025_308_MOESM5_ESM.zip › Figure 3/3f, g/PP10 5-13.png]

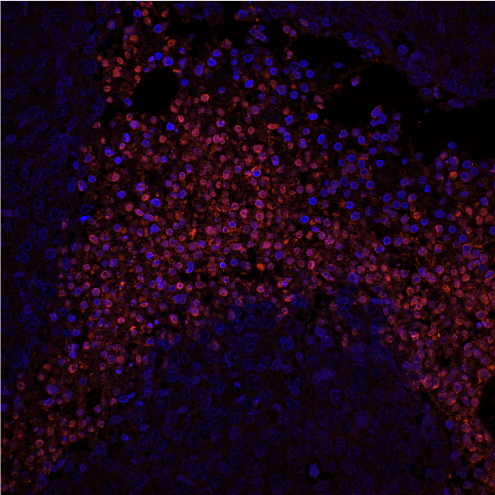

Supplement: Supplementary file 5 — Source data Fig. 3 [file 44321_2025_308_MOESM5_ESM.zip › Figure 3/3f, g/PP10 5-21.png]

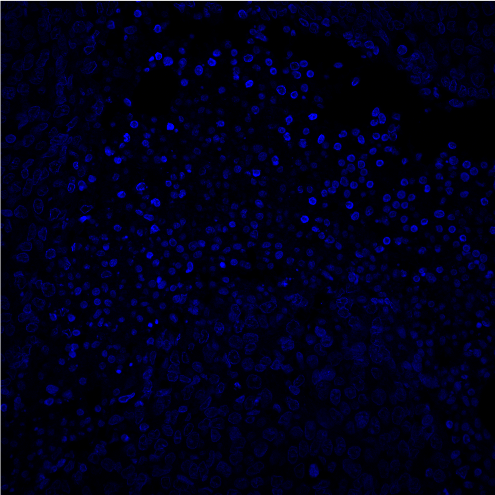

Supplement: Supplementary file 5 — Source data Fig. 3 [file 44321_2025_308_MOESM5_ESM.zip › Figure 3/3f, g/PP10 5-22.png]

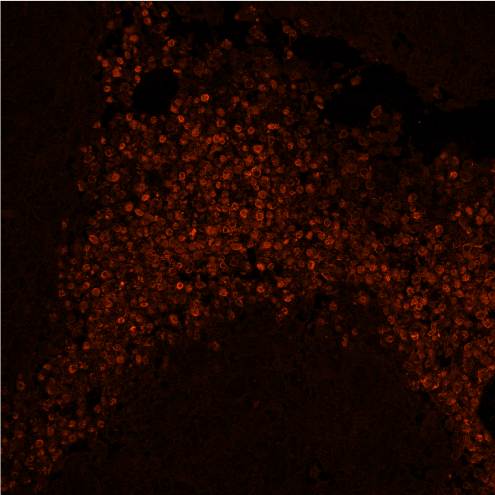

Supplement: Supplementary file 5 — Source data Fig. 3 [file 44321_2025_308_MOESM5_ESM.zip › Figure 3/3f, g/PP10 5-23.png]

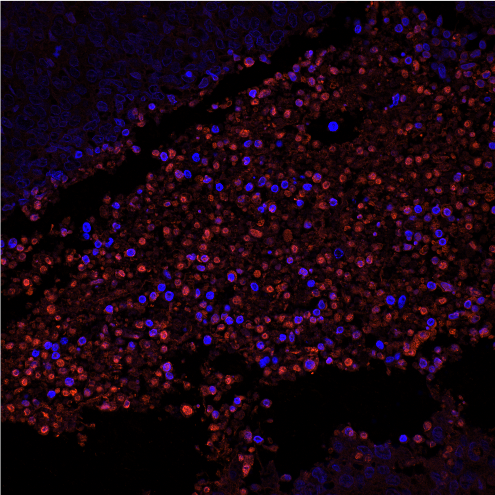

Supplement: Supplementary file 5 — Source data Fig. 3 [file 44321_2025_308_MOESM5_ESM.zip › Figure 3/3f, g/PP10 5-31.png]

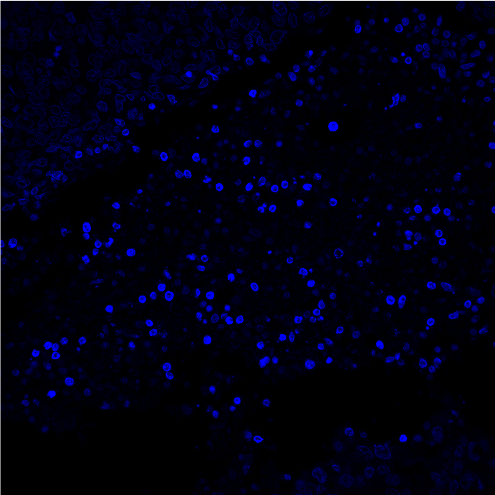

Supplement: Supplementary file 5 — Source data Fig. 3 [file 44321_2025_308_MOESM5_ESM.zip › Figure 3/3f, g/PP10 5-32.png]

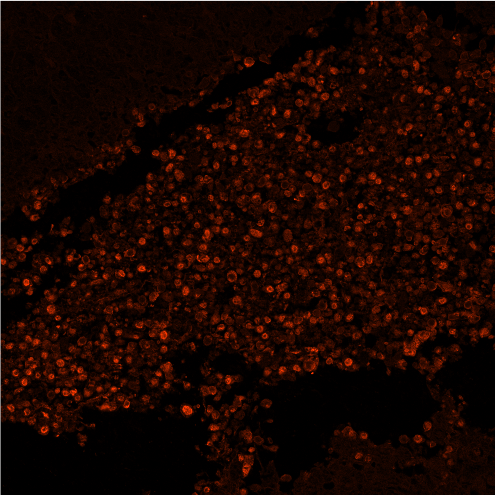

Supplement: Supplementary file 5 — Source data Fig. 3 [file 44321_2025_308_MOESM5_ESM.zip › Figure 3/3f, g/PP10 5-33.png]

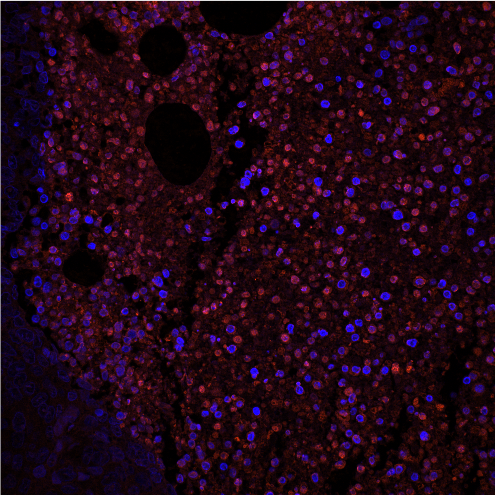

Supplement: Supplementary file 5 — Source data Fig. 3 [file 44321_2025_308_MOESM5_ESM.zip › Figure 3/3f, g/PP10 5-41.png]

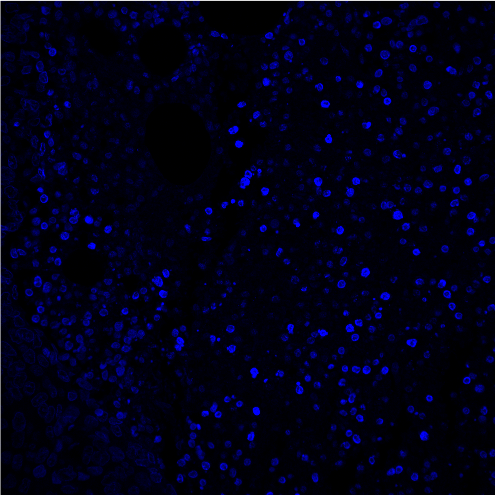

Supplement: Supplementary file 5 — Source data Fig. 3 [file 44321_2025_308_MOESM5_ESM.zip › Figure 3/3f, g/PP10 5-42.png]

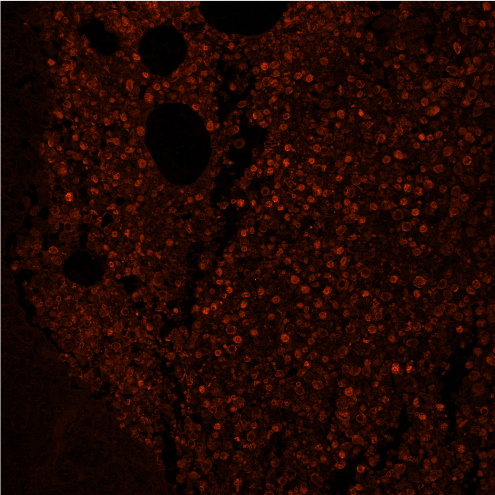

Supplement: Supplementary file 5 — Source data Fig. 3 [file 44321_2025_308_MOESM5_ESM.zip › Figure 3/3f, g/PP10 5-43.png]

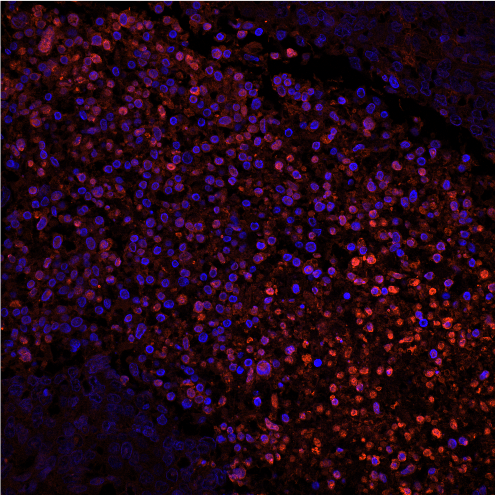

Supplement: Supplementary file 5 — Source data Fig. 3 [file 44321_2025_308_MOESM5_ESM.zip › Figure 3/3f, g/PP10 5-51.png]

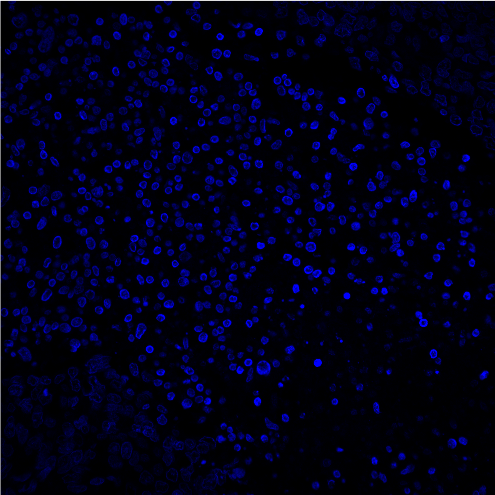

Supplement: Supplementary file 5 — Source data Fig. 3 [file 44321_2025_308_MOESM5_ESM.zip › Figure 3/3f, g/PP10 5-52.png]

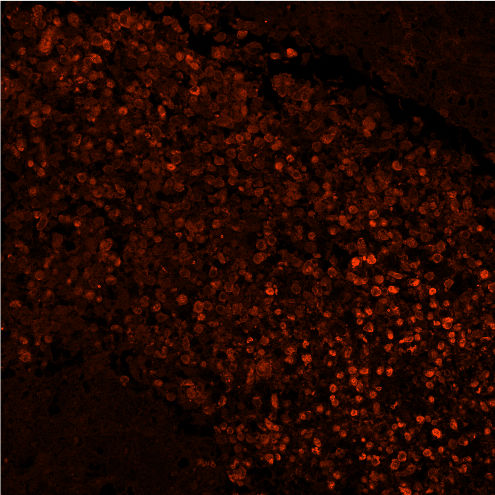

Supplement: Supplementary file 5 — Source data Fig. 3 [file 44321_2025_308_MOESM5_ESM.zip › Figure 3/3f, g/PP10 5-53.png]

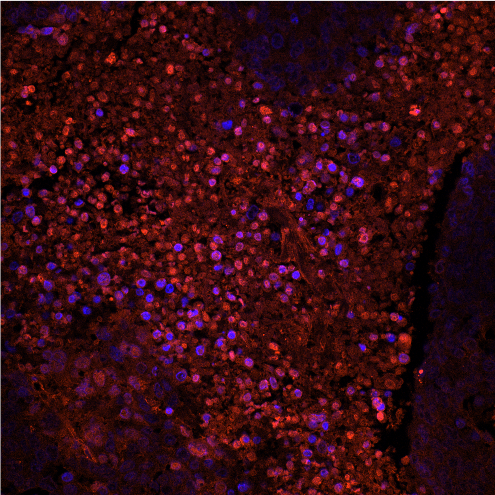

Supplement: Supplementary file 5 — Source data Fig. 3 [file 44321_2025_308_MOESM5_ESM.zip › Figure 3/3f, g/PP24 2. 5-31.png]

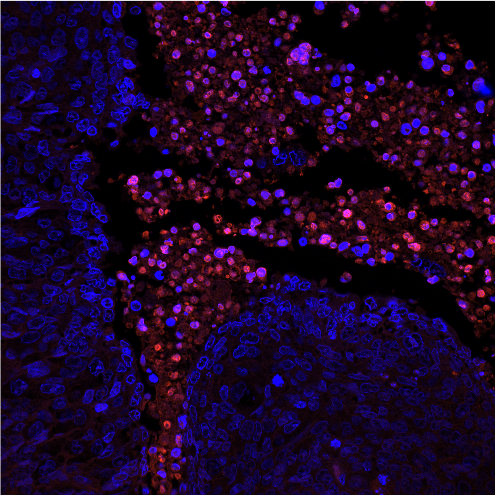

Supplement: Supplementary file 5 — Source data Fig. 3 [file 44321_2025_308_MOESM5_ESM.zip › Figure 3/3f, g/PP24 2.5-11.png]

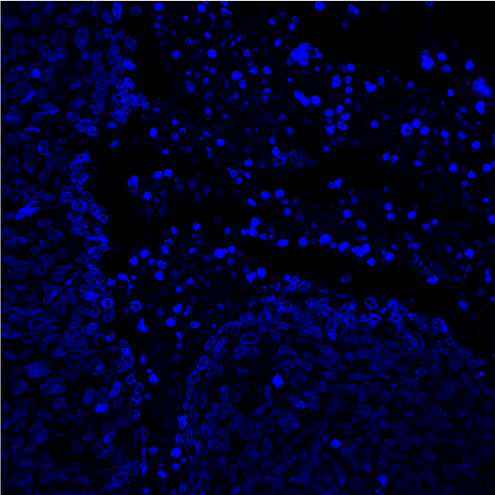

Supplement: Supplementary file 5 — Source data Fig. 3 [file 44321_2025_308_MOESM5_ESM.zip › Figure 3/3f, g/PP24 2.5-12.png]

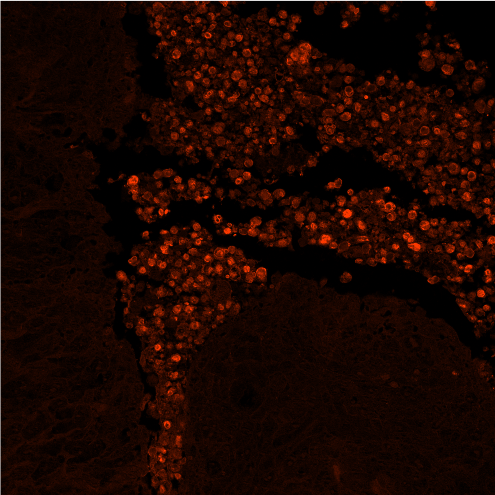

Supplement: Supplementary file 5 — Source data Fig. 3 [file 44321_2025_308_MOESM5_ESM.zip › Figure 3/3f, g/PP24 2.5-13.png]

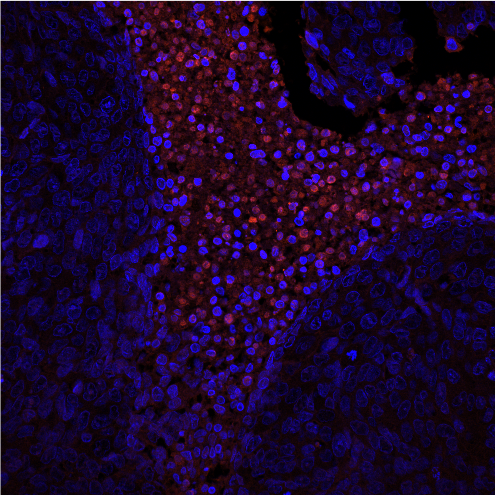

Supplement: Supplementary file 5 — Source data Fig. 3 [file 44321_2025_308_MOESM5_ESM.zip › Figure 3/3f, g/PP24 2.5-21.png]

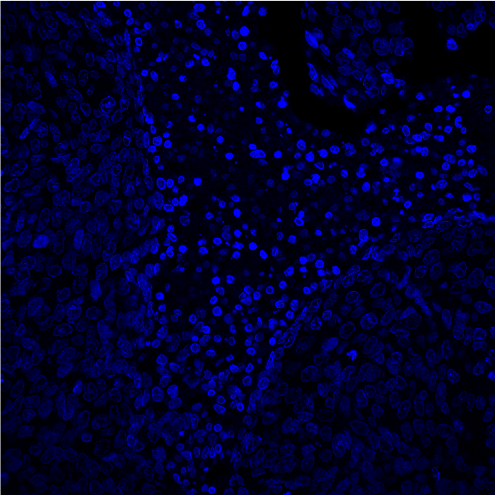

Supplement: Supplementary file 5 — Source data Fig. 3 [file 44321_2025_308_MOESM5_ESM.zip › Figure 3/3f, g/PP24 2.5-22.png]

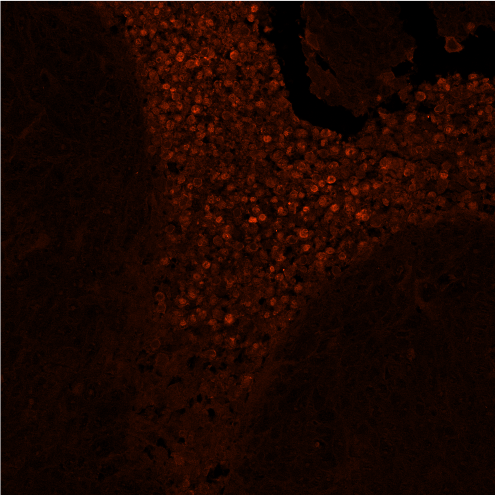

Supplement: Supplementary file 5 — Source data Fig. 3 [file 44321_2025_308_MOESM5_ESM.zip › Figure 3/3f, g/PP24 2.5-23.png]

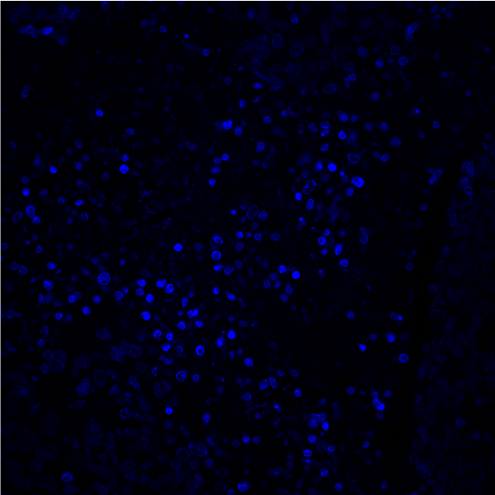

Supplement: Supplementary file 5 — Source data Fig. 3 [file 44321_2025_308_MOESM5_ESM.zip › Figure 3/3f, g/PP24 2.5-32.png]

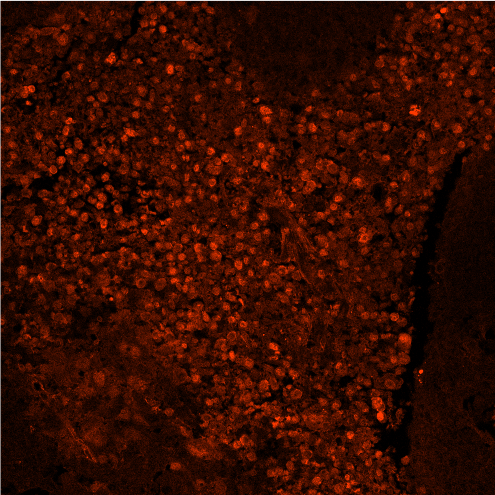

Supplement: Supplementary file 5 — Source data Fig. 3 [file 44321_2025_308_MOESM5_ESM.zip › Figure 3/3f, g/PP24 2.5-33.png]

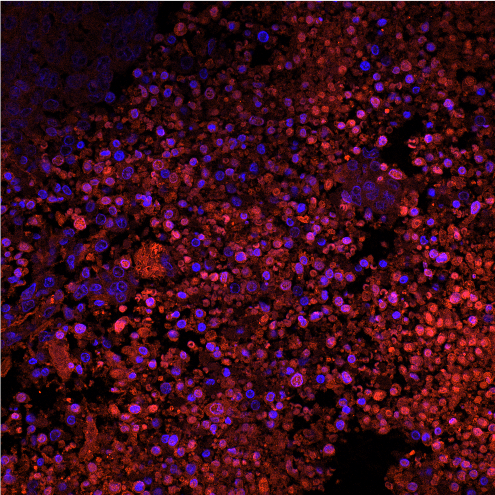

Supplement: Supplementary file 5 — Source data Fig. 3 [file 44321_2025_308_MOESM5_ESM.zip › Figure 3/3f, g/PP24 5-11.png]

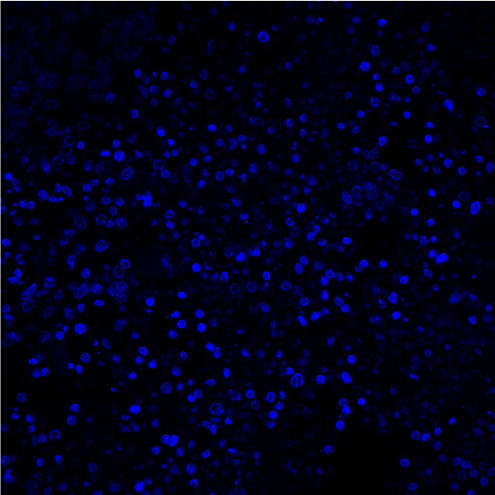

Supplement: Supplementary file 5 — Source data Fig. 3 [file 44321_2025_308_MOESM5_ESM.zip › Figure 3/3f, g/PP24 5-12.png]

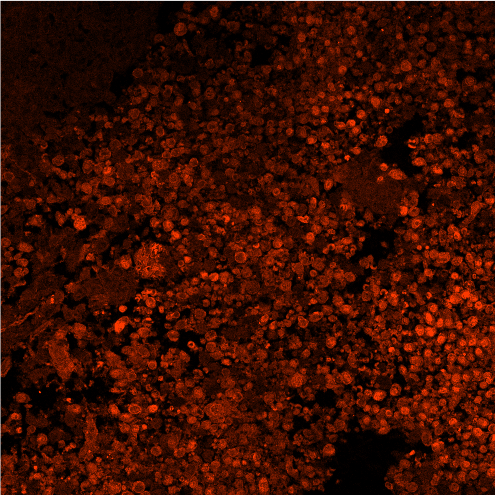

Supplement: Supplementary file 5 — Source data Fig. 3 [file 44321_2025_308_MOESM5_ESM.zip › Figure 3/3f, g/PP24 5-13.png]

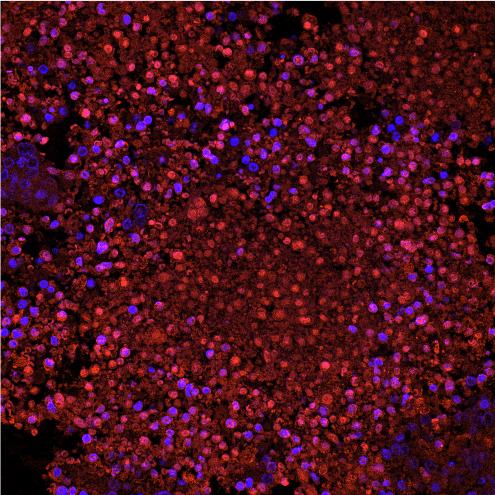

Supplement: Supplementary file 5 — Source data Fig. 3 [file 44321_2025_308_MOESM5_ESM.zip › Figure 3/3f, g/PP24 5-21.png]

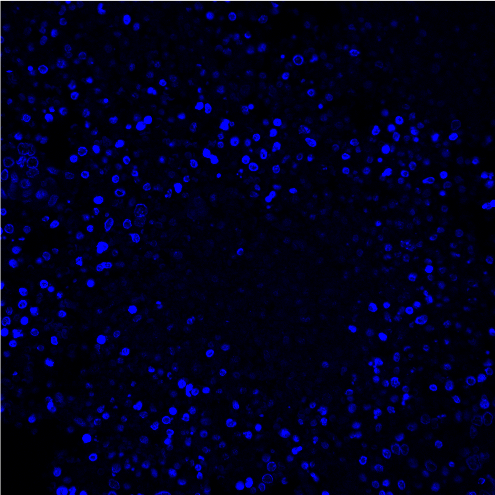

Supplement: Supplementary file 5 — Source data Fig. 3 [file 44321_2025_308_MOESM5_ESM.zip › Figure 3/3f, g/PP24 5-22.png]

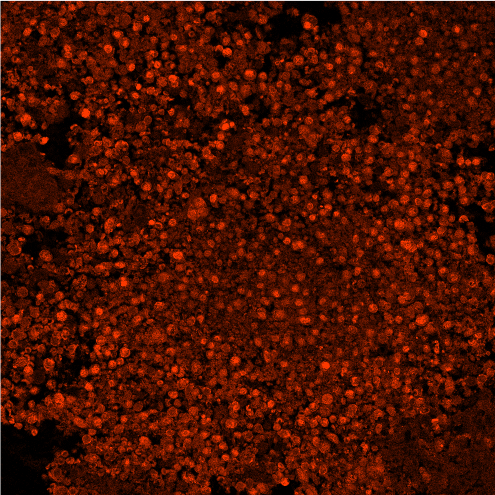

Supplement: Supplementary file 5 — Source data Fig. 3 [file 44321_2025_308_MOESM5_ESM.zip › Figure 3/3f, g/PP24 5-23.png]

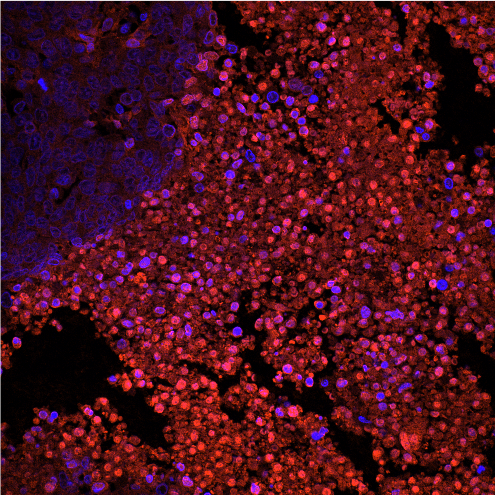

Supplement: Supplementary file 5 — Source data Fig. 3 [file 44321_2025_308_MOESM5_ESM.zip › Figure 3/3f, g/PP24 5-31.png]

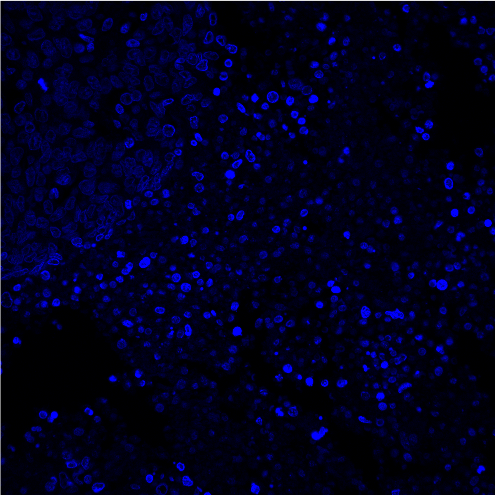

Supplement: Supplementary file 5 — Source data Fig. 3 [file 44321_2025_308_MOESM5_ESM.zip › Figure 3/3f, g/PP24 5-32.png]

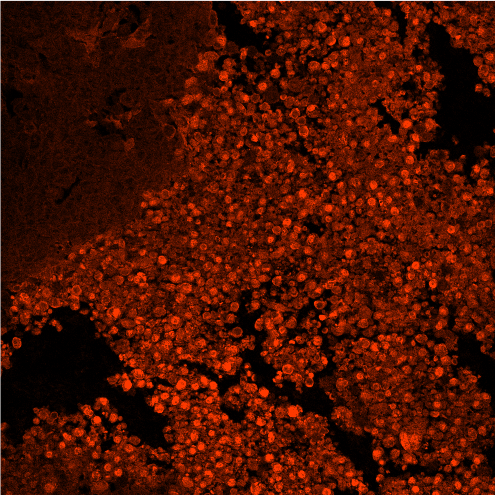

Supplement: Supplementary file 5 — Source data Fig. 3 [file 44321_2025_308_MOESM5_ESM.zip › Figure 3/3f, g/PP24 5-33.png]

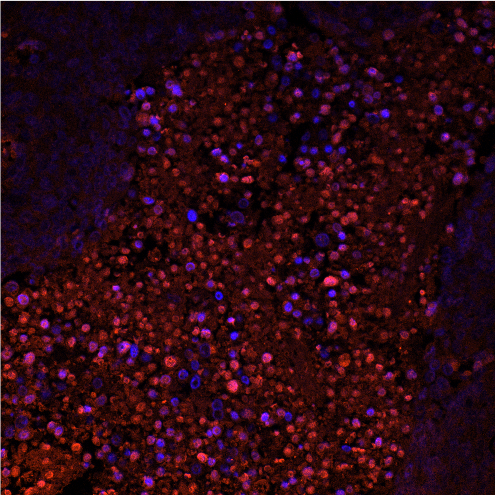

Supplement: Supplementary file 5 — Source data Fig. 3 [file 44321_2025_308_MOESM5_ESM.zip › Figure 3/3f, g/PP24 5-41.png]

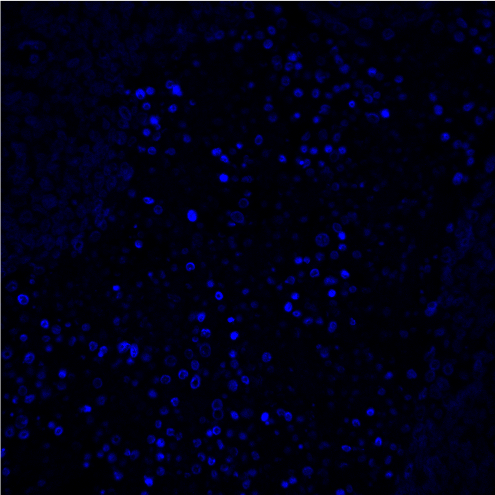

Supplement: Supplementary file 5 — Source data Fig. 3 [file 44321_2025_308_MOESM5_ESM.zip › Figure 3/3f, g/PP24 5-42.png]

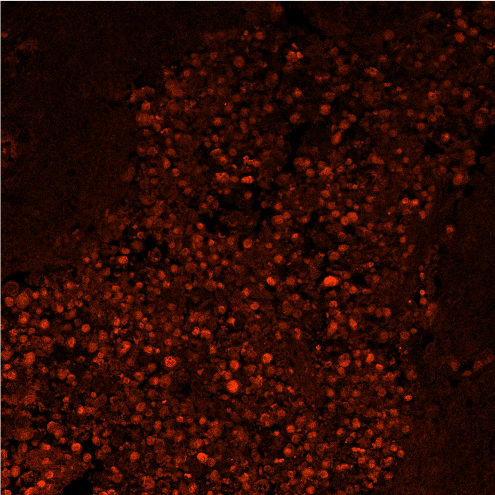

Supplement: Supplementary file 5 — Source data Fig. 3 [file 44321_2025_308_MOESM5_ESM.zip › Figure 3/3f, g/PP24 5-43.png]

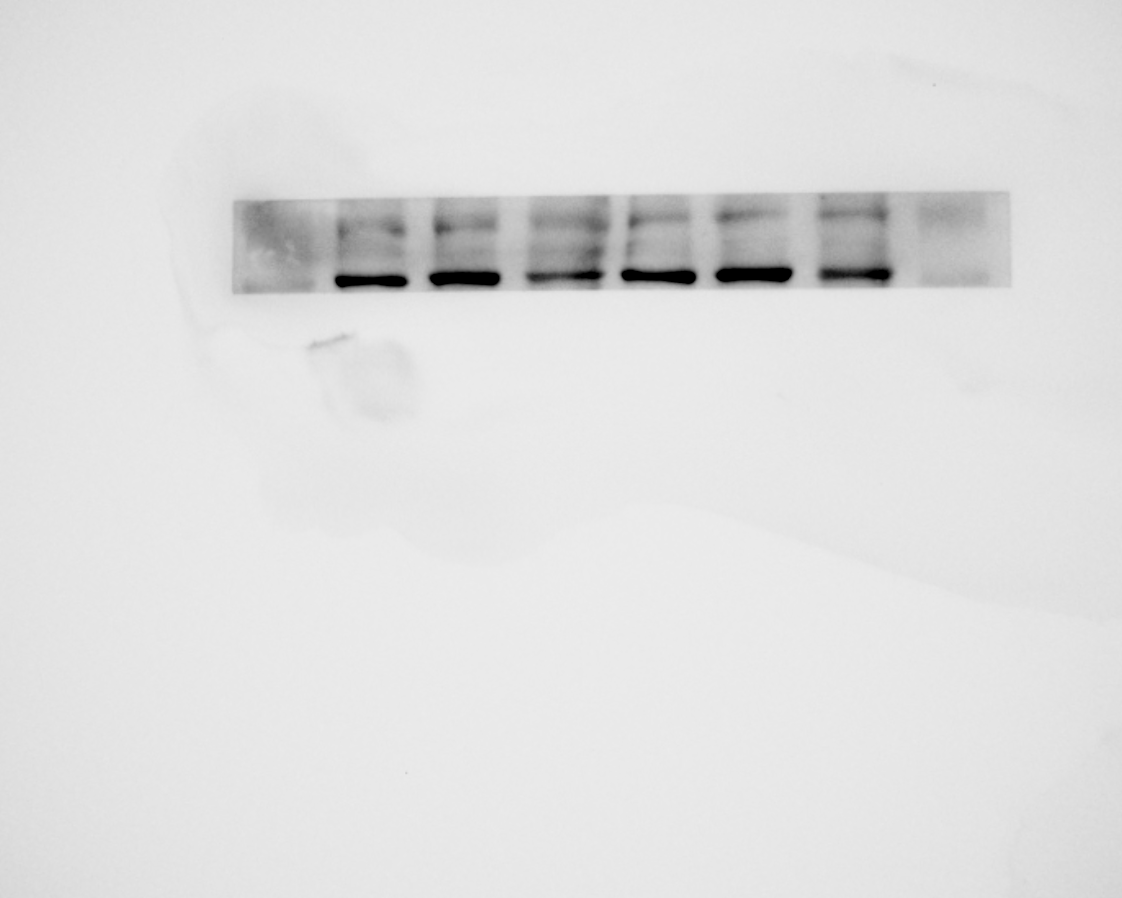

Supplement: Supplementary file 6 — Source data Fig. 4 [file 44321_2025_308_MOESM6_ESM.zip › Figure 4/4h/western akt 1.tif]

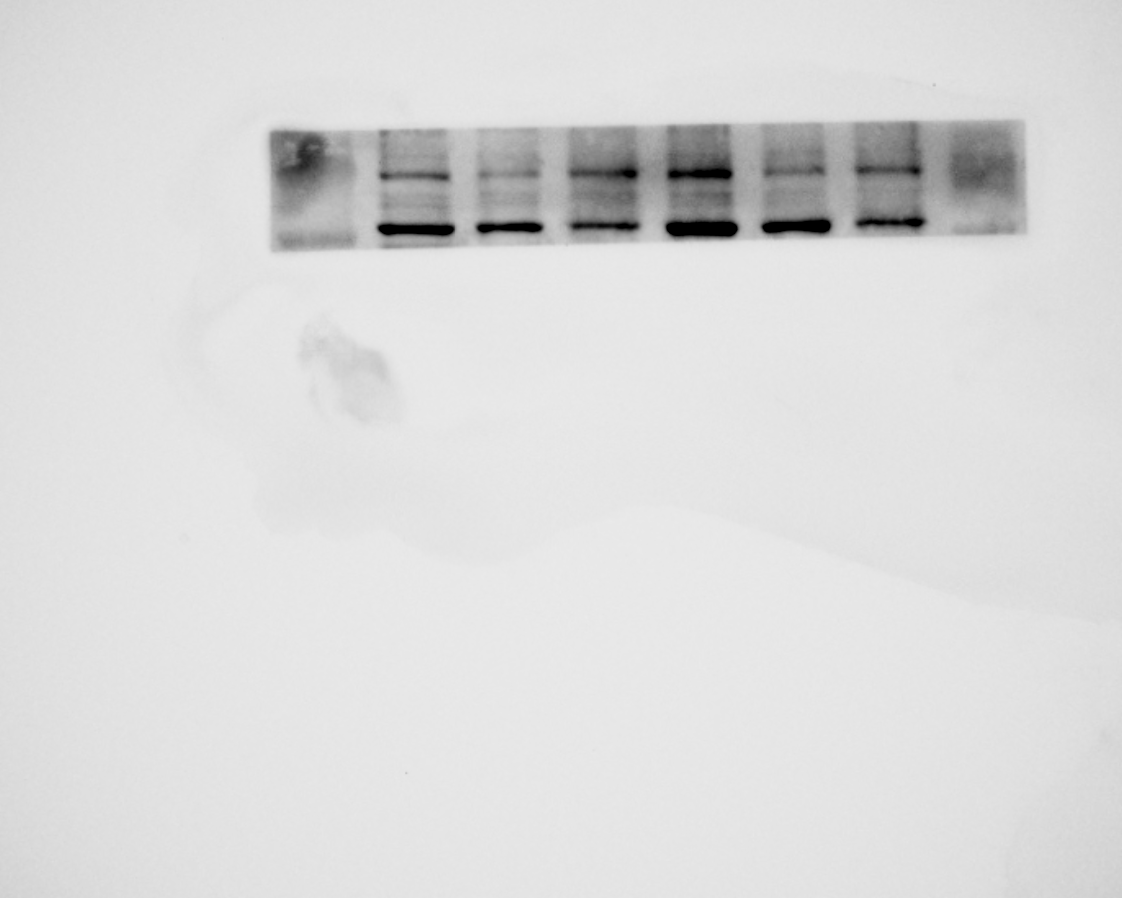

Supplement: Supplementary file 6 — Source data Fig. 4 [file 44321_2025_308_MOESM6_ESM.zip › Figure 4/4h/western akt 2.tif]

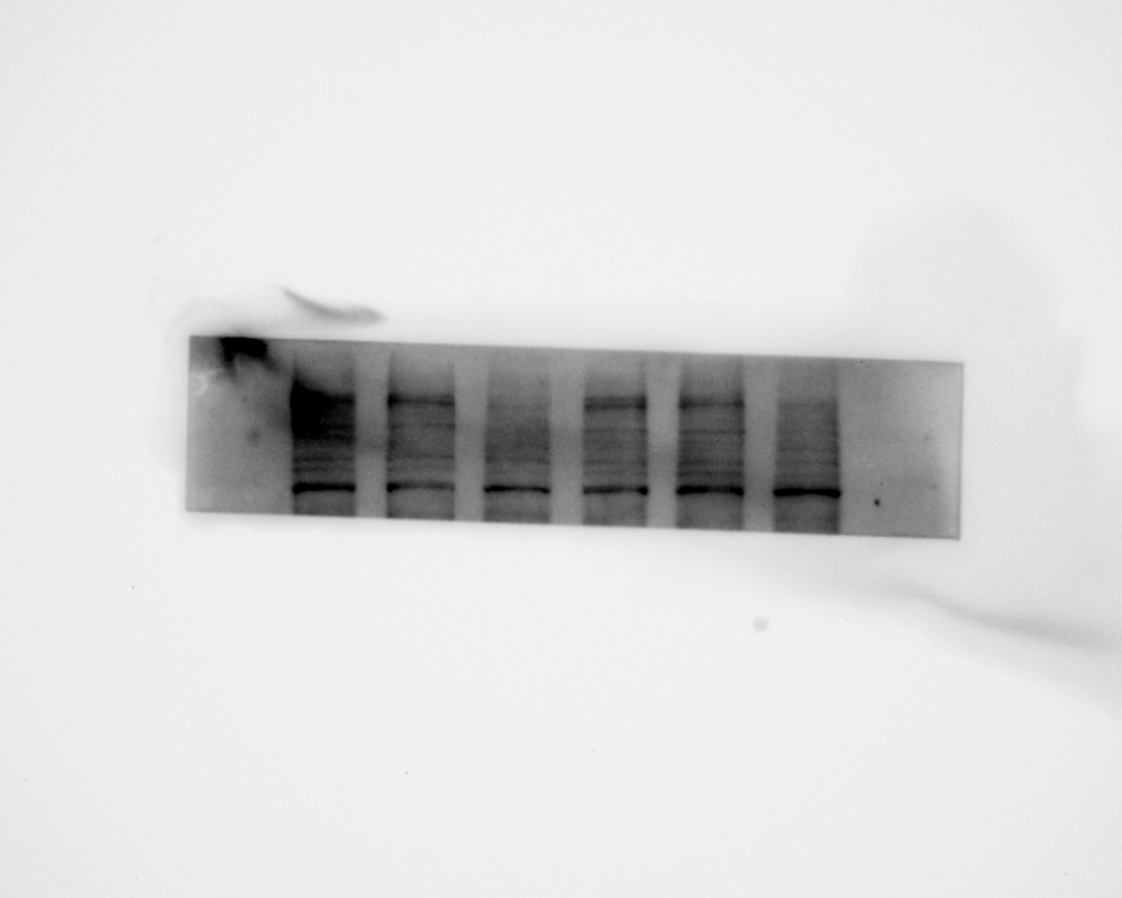

Supplement: Supplementary file 6 — Source data Fig. 4 [file 44321_2025_308_MOESM6_ESM.zip › Figure 4/4h/western mtor 1.tif]

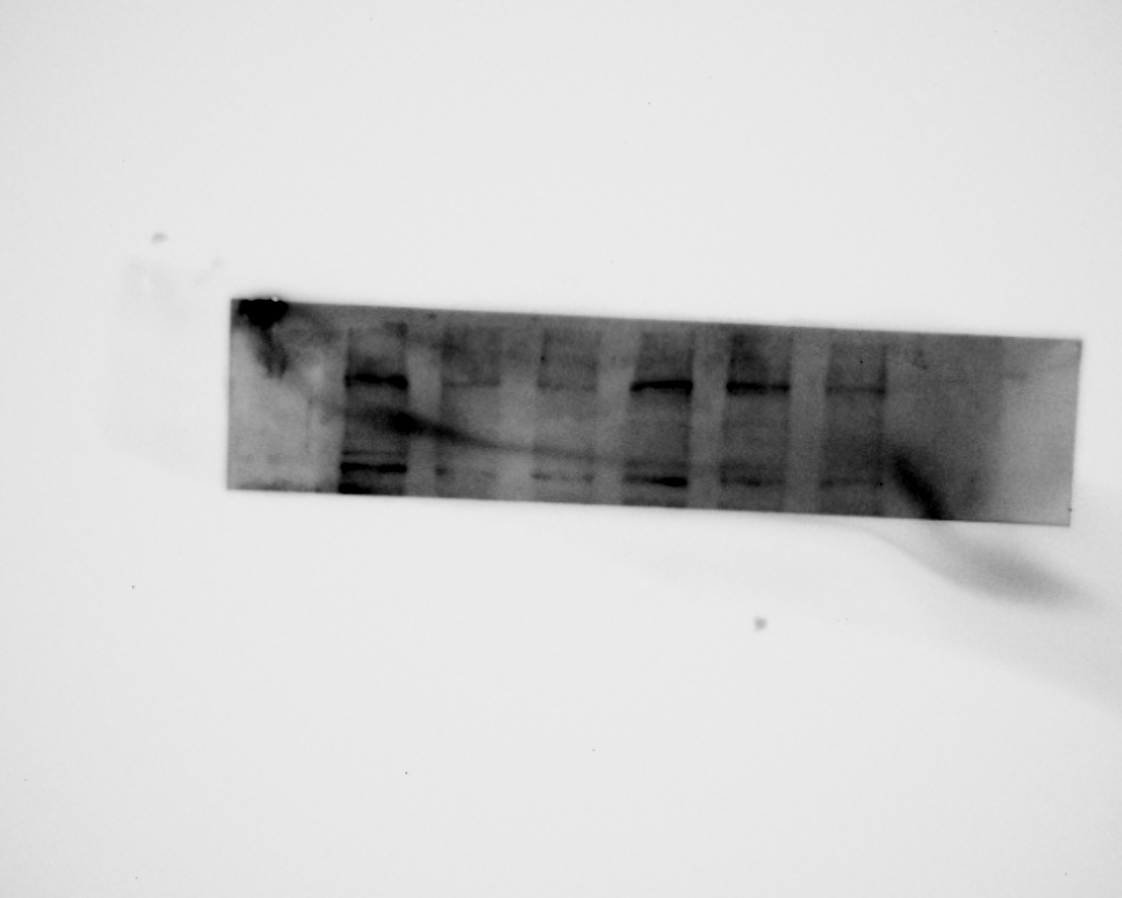

Supplement: Supplementary file 6 — Source data Fig. 4 [file 44321_2025_308_MOESM6_ESM.zip › Figure 4/4h/western mtor 2.tif]

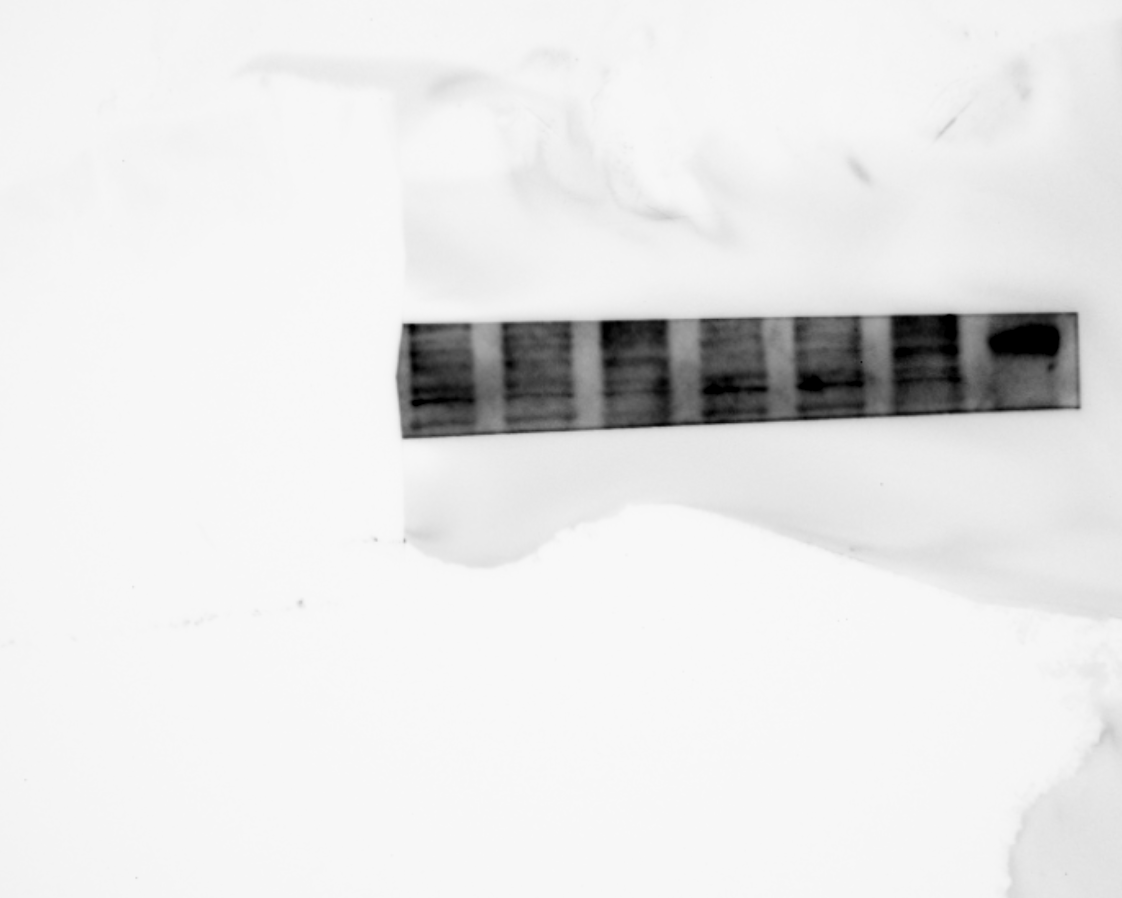

Supplement: Supplementary file 6 — Source data Fig. 4 [file 44321_2025_308_MOESM6_ESM.zip › Figure 4/4h/western pakt 1.tif]

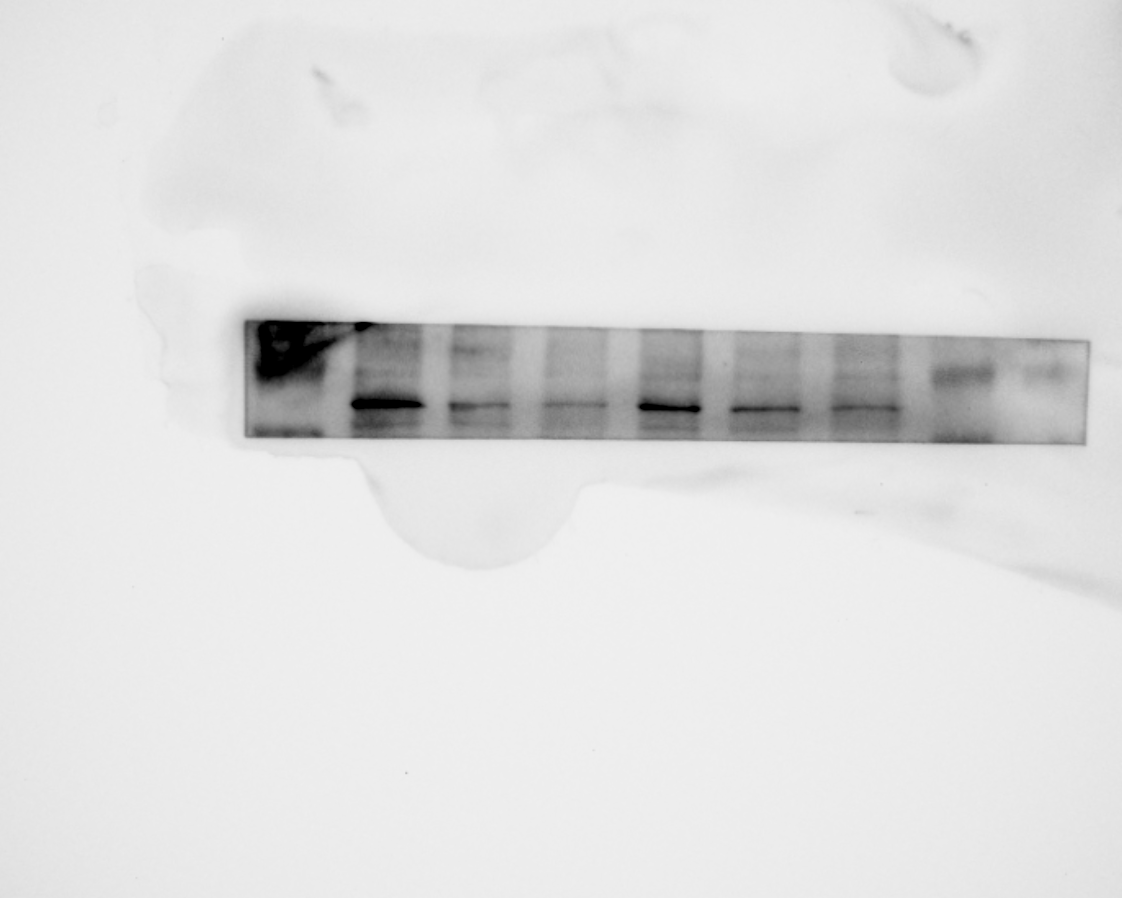

Supplement: Supplementary file 6 — Source data Fig. 4 [file 44321_2025_308_MOESM6_ESM.zip › Figure 4/4h/western pakt 2.tif]

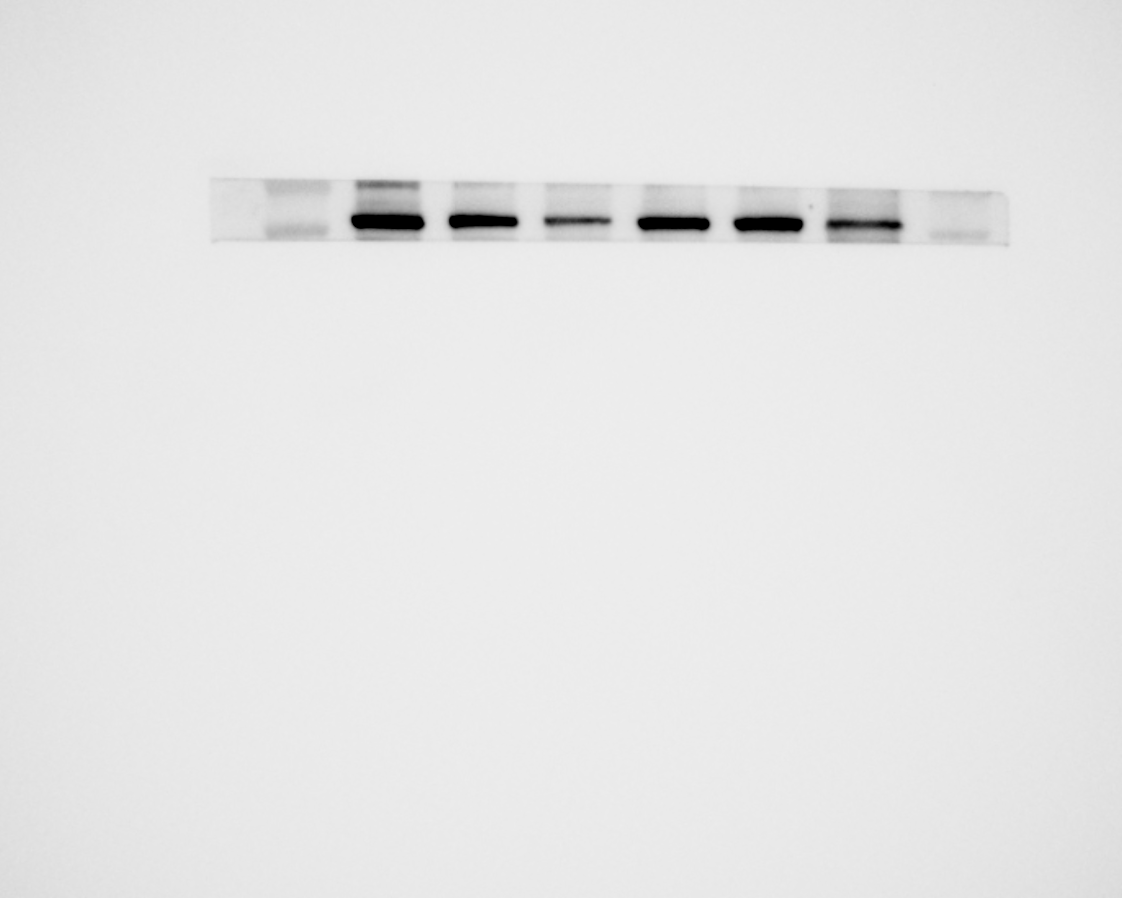

Supplement: Supplementary file 6 — Source data Fig. 4 [file 44321_2025_308_MOESM6_ESM.zip › Figure 4/4h/western pi3k 1.tif]

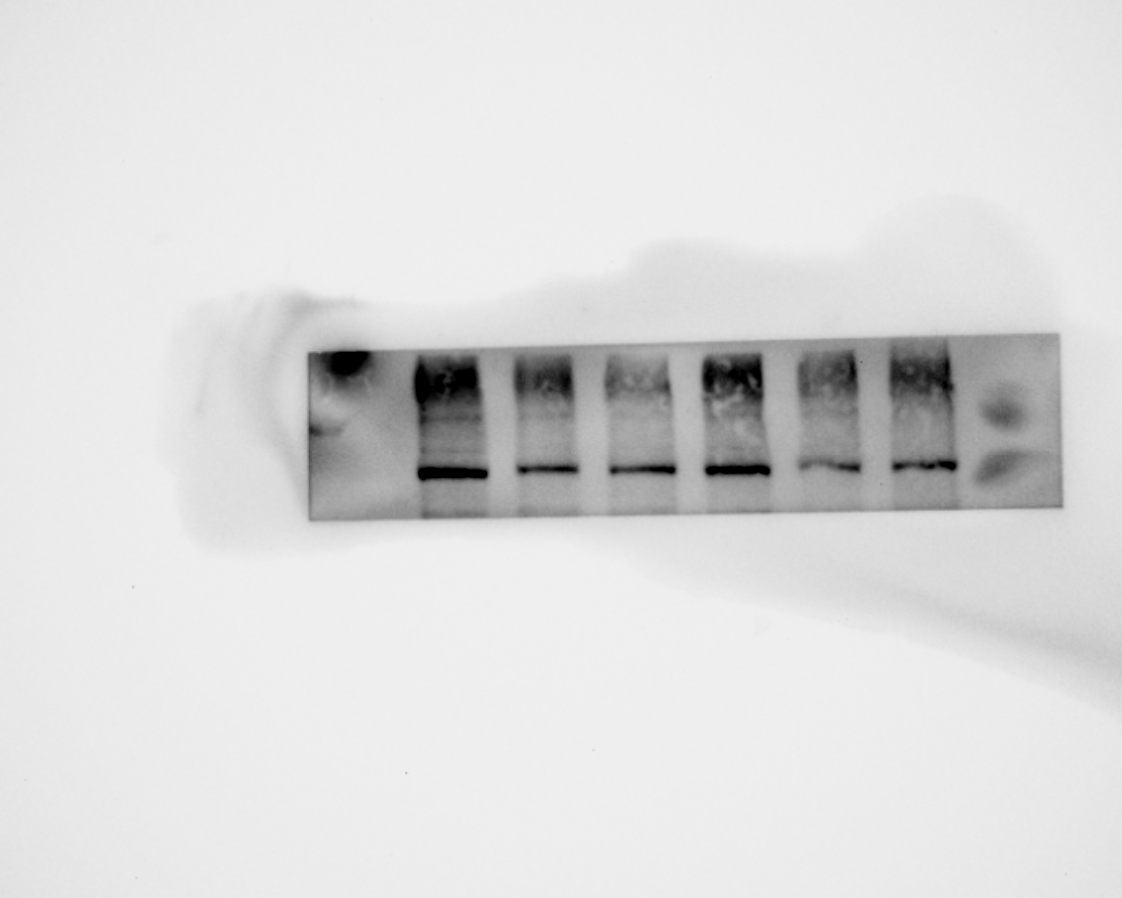

Supplement: Supplementary file 6 — Source data Fig. 4 [file 44321_2025_308_MOESM6_ESM.zip › Figure 4/4h/western pi3k 2.tif]

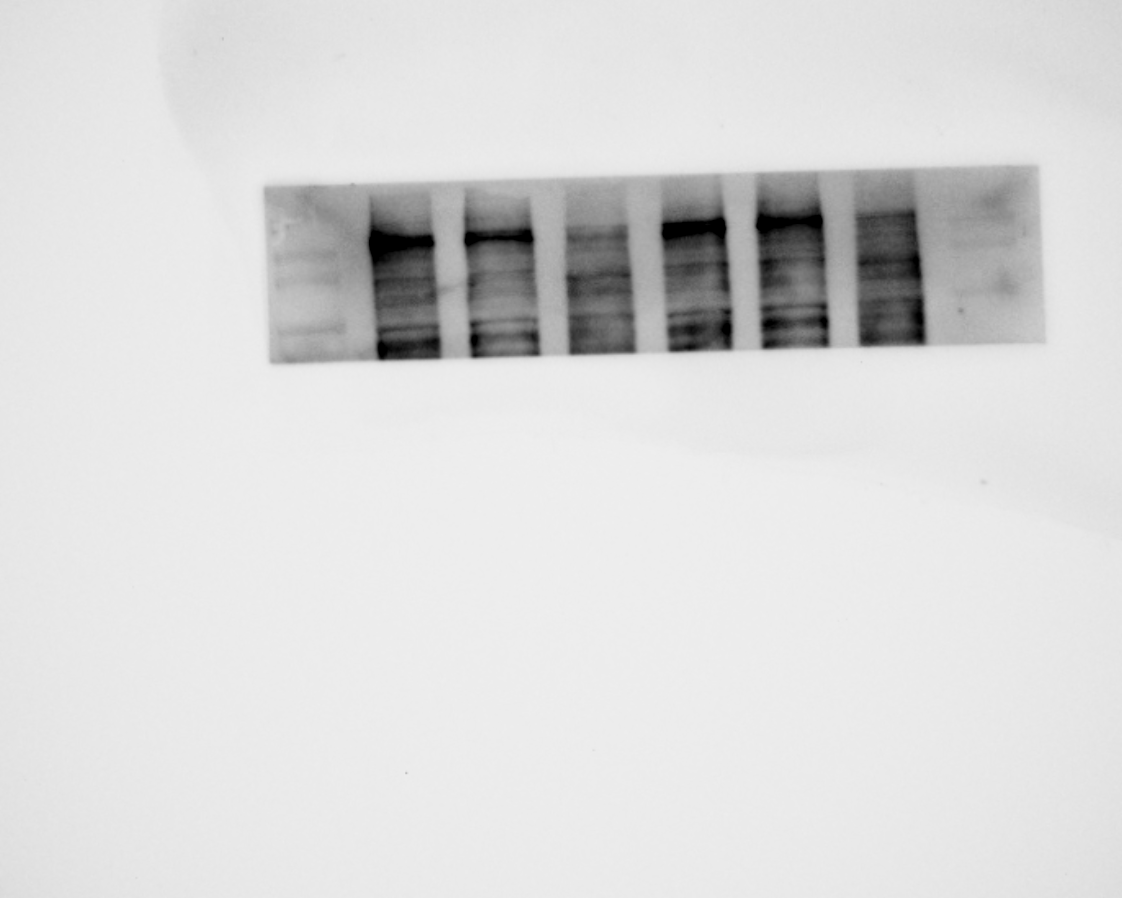

Supplement: Supplementary file 6 — Source data Fig. 4 [file 44321_2025_308_MOESM6_ESM.zip › Figure 4/4h/western pmtor 1.tif]

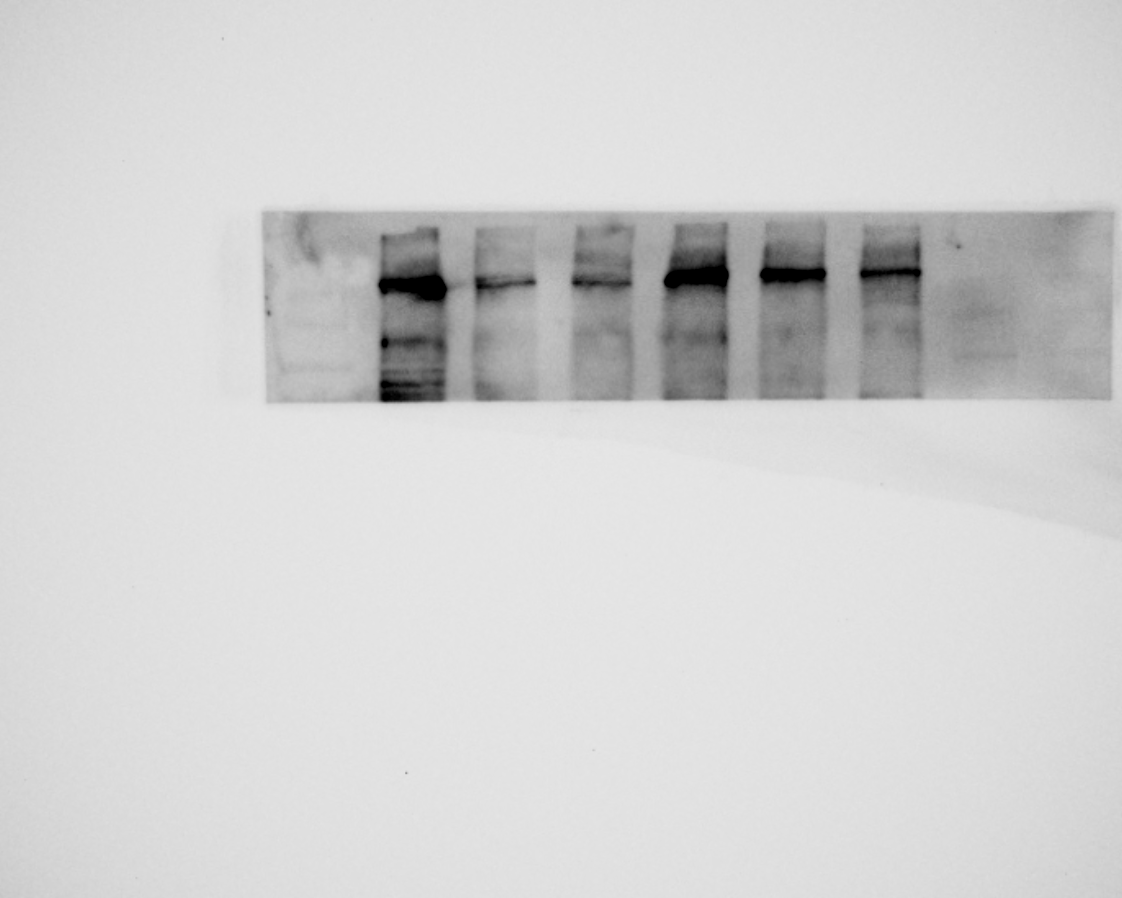

Supplement: Supplementary file 6 — Source data Fig. 4 [file 44321_2025_308_MOESM6_ESM.zip › Figure 4/4h/western pmtor 2.tif]

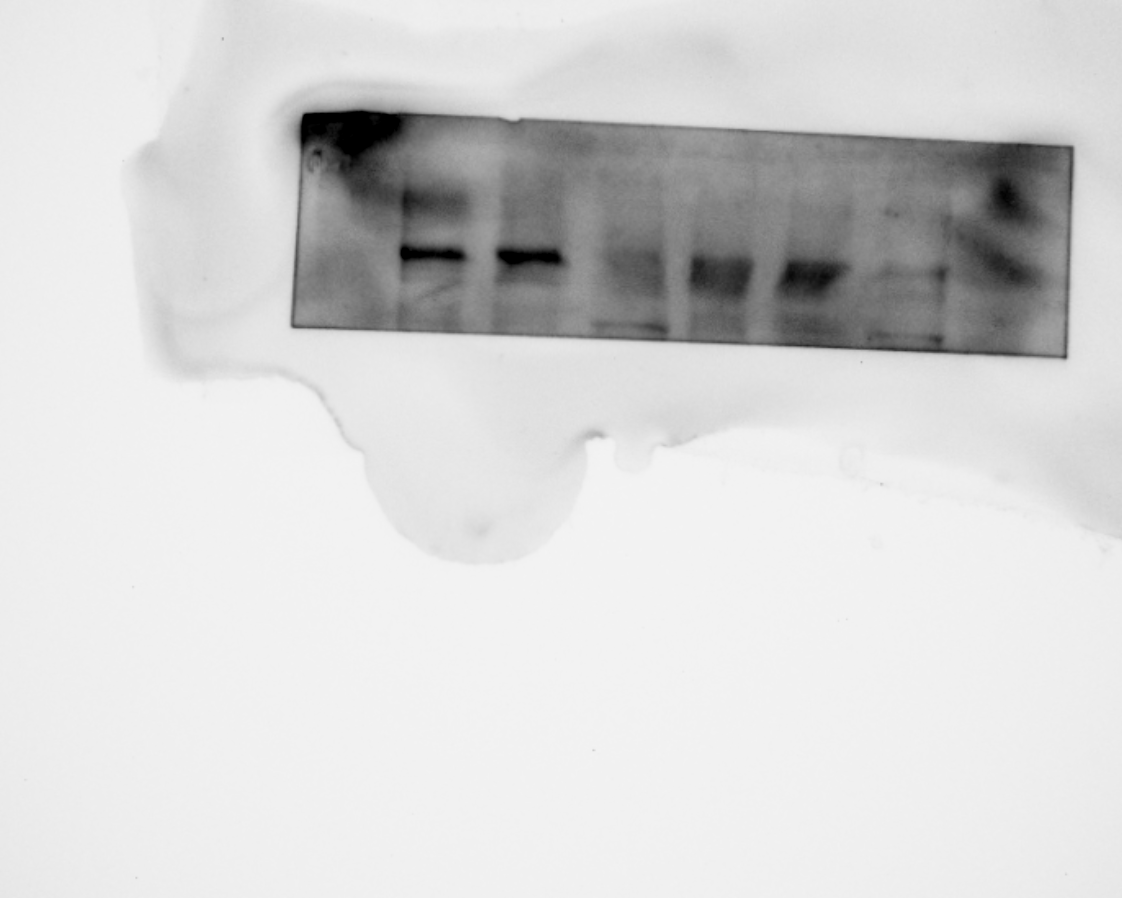

Supplement: Supplementary file 6 — Source data Fig. 4 [file 44321_2025_308_MOESM6_ESM.zip › Figure 4/4h/western ppi3k 1.tif]

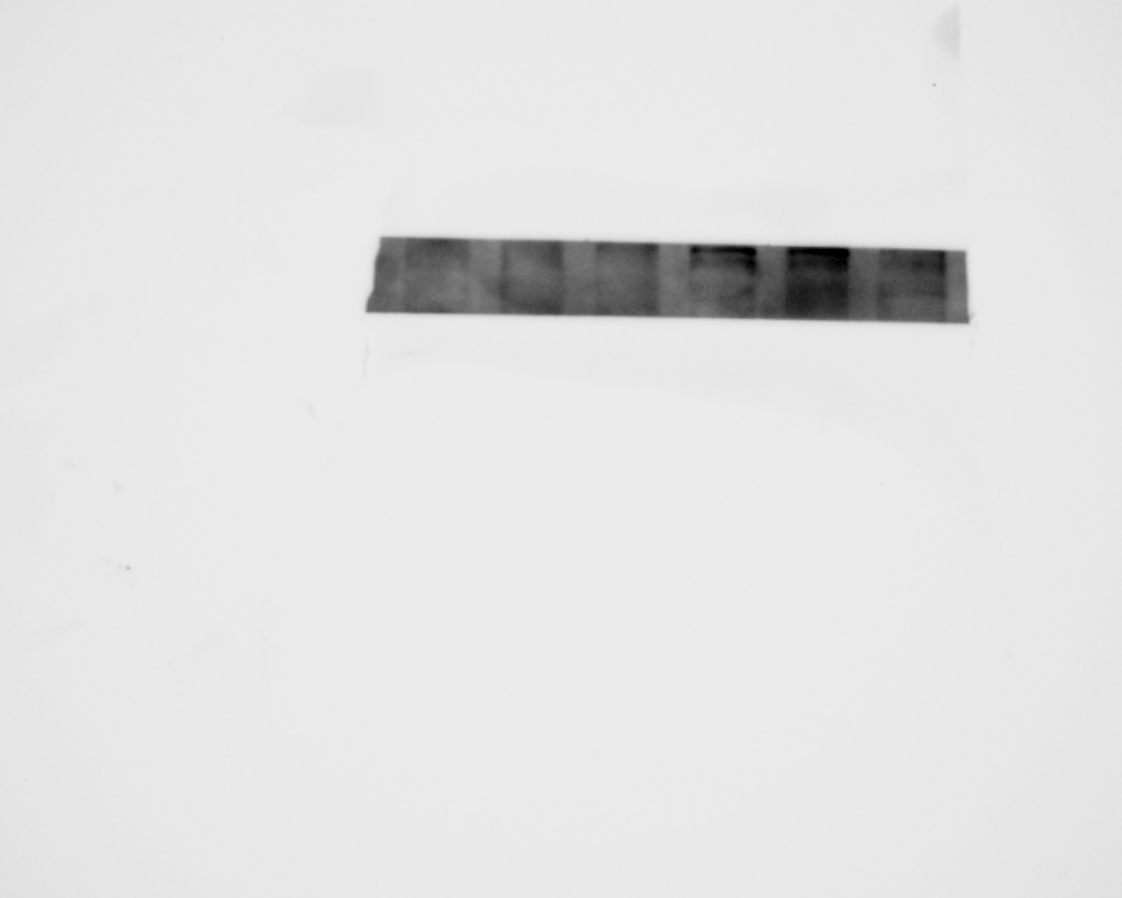

Supplement: Supplementary file 6 — Source data Fig. 4 [file 44321_2025_308_MOESM6_ESM.zip › Figure 4/4h/western ppi3k 2.tif]

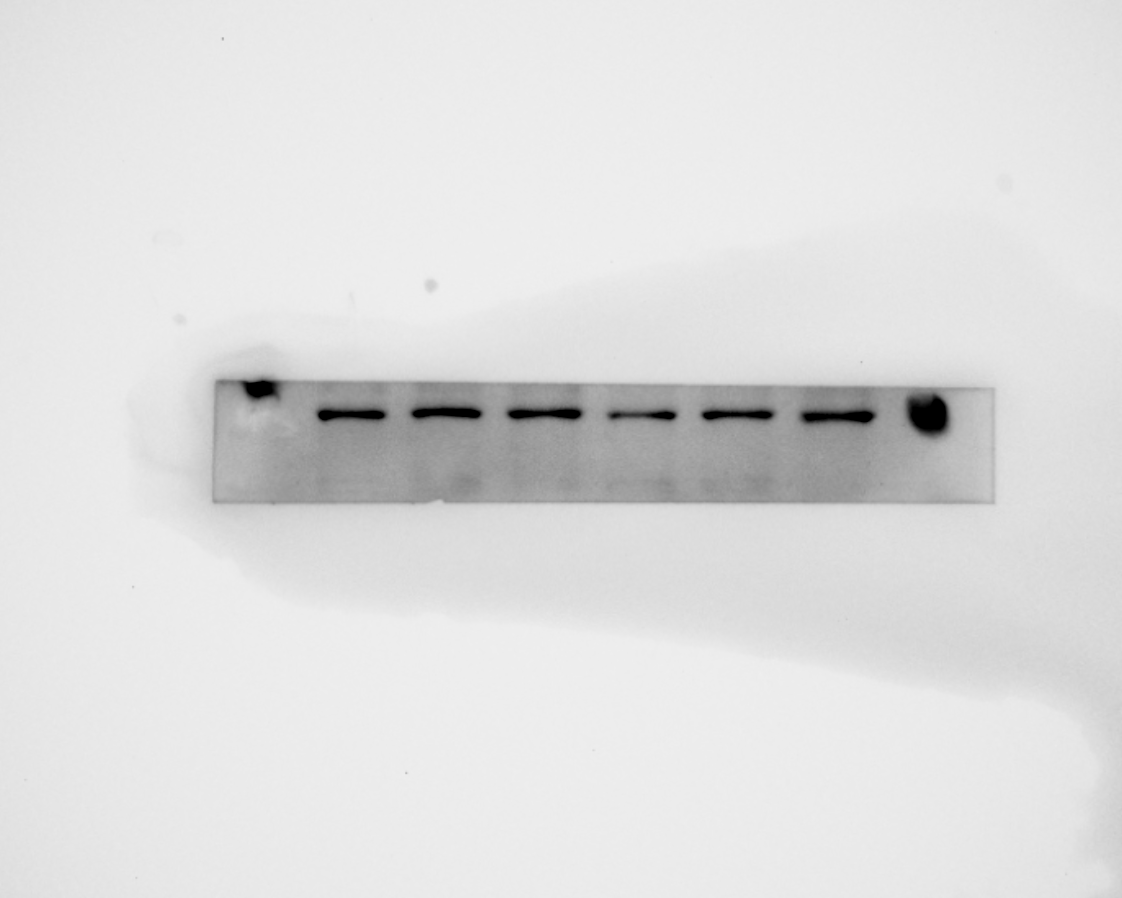

Supplement: Supplementary file 6 — Source data Fig. 4 [file 44321_2025_308_MOESM6_ESM.zip › Figure 4/4h/western-actin 1.tif]

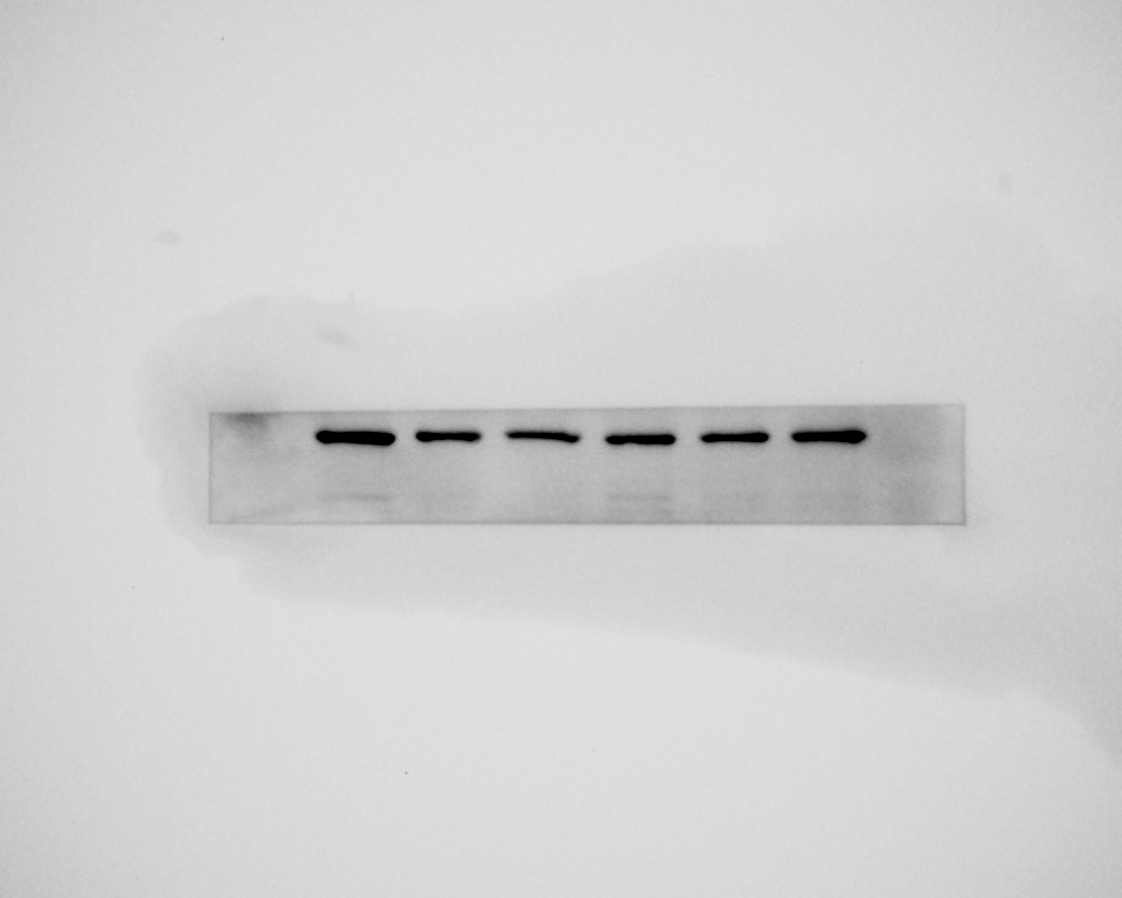

Supplement: Supplementary file 6 — Source data Fig. 4 [file 44321_2025_308_MOESM6_ESM.zip › Figure 4/4h/western-actin 2.tif]

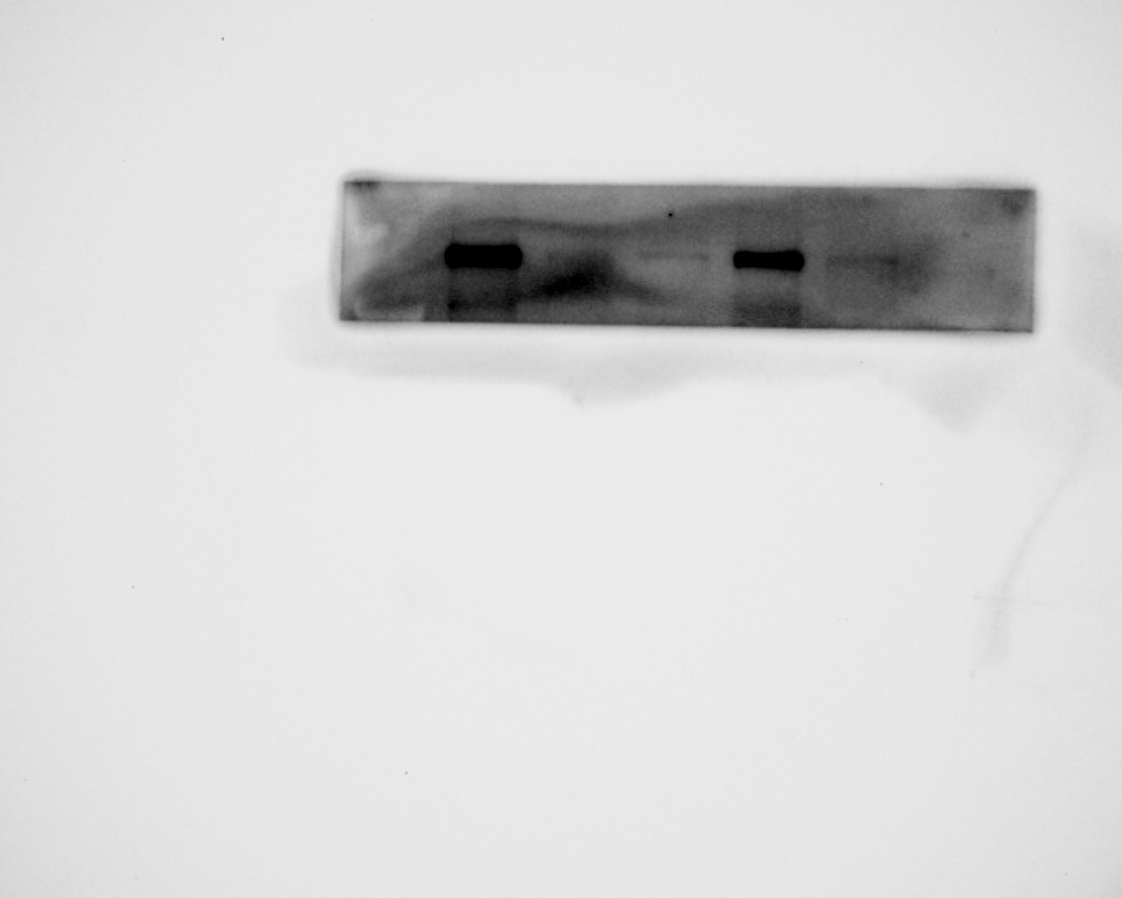

Supplement: Supplementary file 6 — Source data Fig. 4 [file 44321_2025_308_MOESM6_ESM.zip › Figure 4/4h/western-pp65 1.tif]

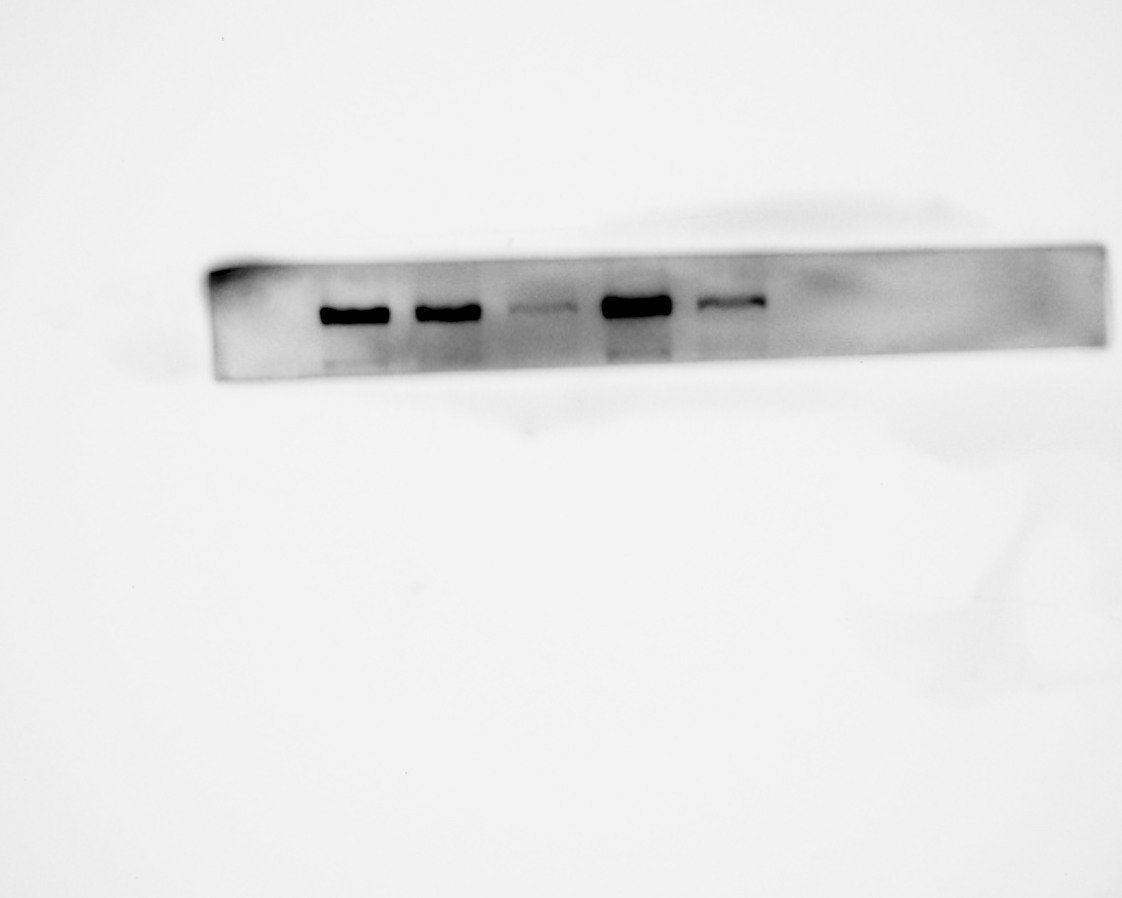

Supplement: Supplementary file 6 — Source data Fig. 4 [file 44321_2025_308_MOESM6_ESM.zip › Figure 4/4h/western-pp65 2.tif]

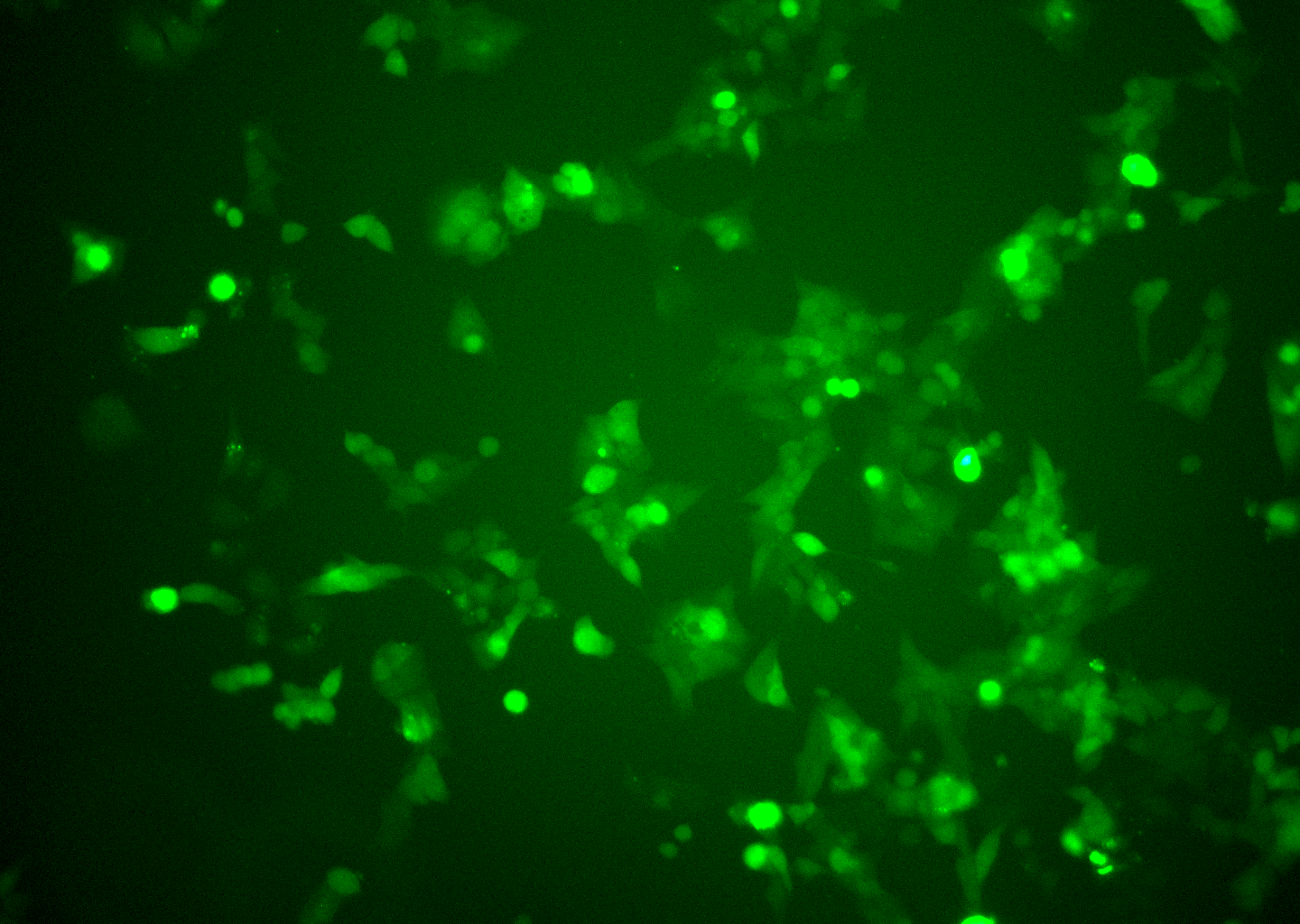

Supplement: Supplementary file 7 — Source data Fig. 5 [file 44321_2025_308_MOESM7_ESM.zip › Figure 5/5a/116 1.5.png]

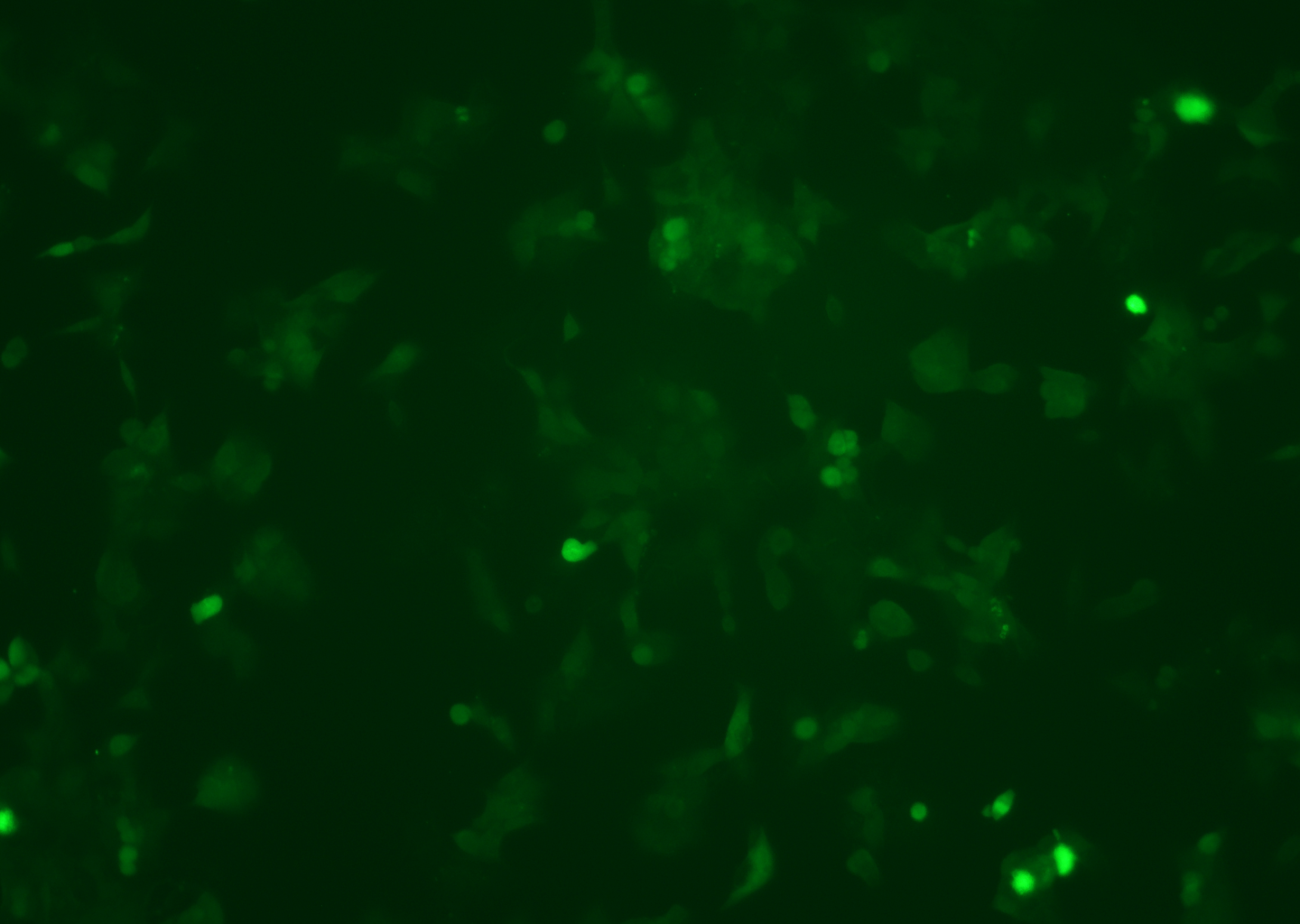

Supplement: Supplementary file 7 — Source data Fig. 5 [file 44321_2025_308_MOESM7_ESM.zip › Figure 5/5a/116 CON.png]

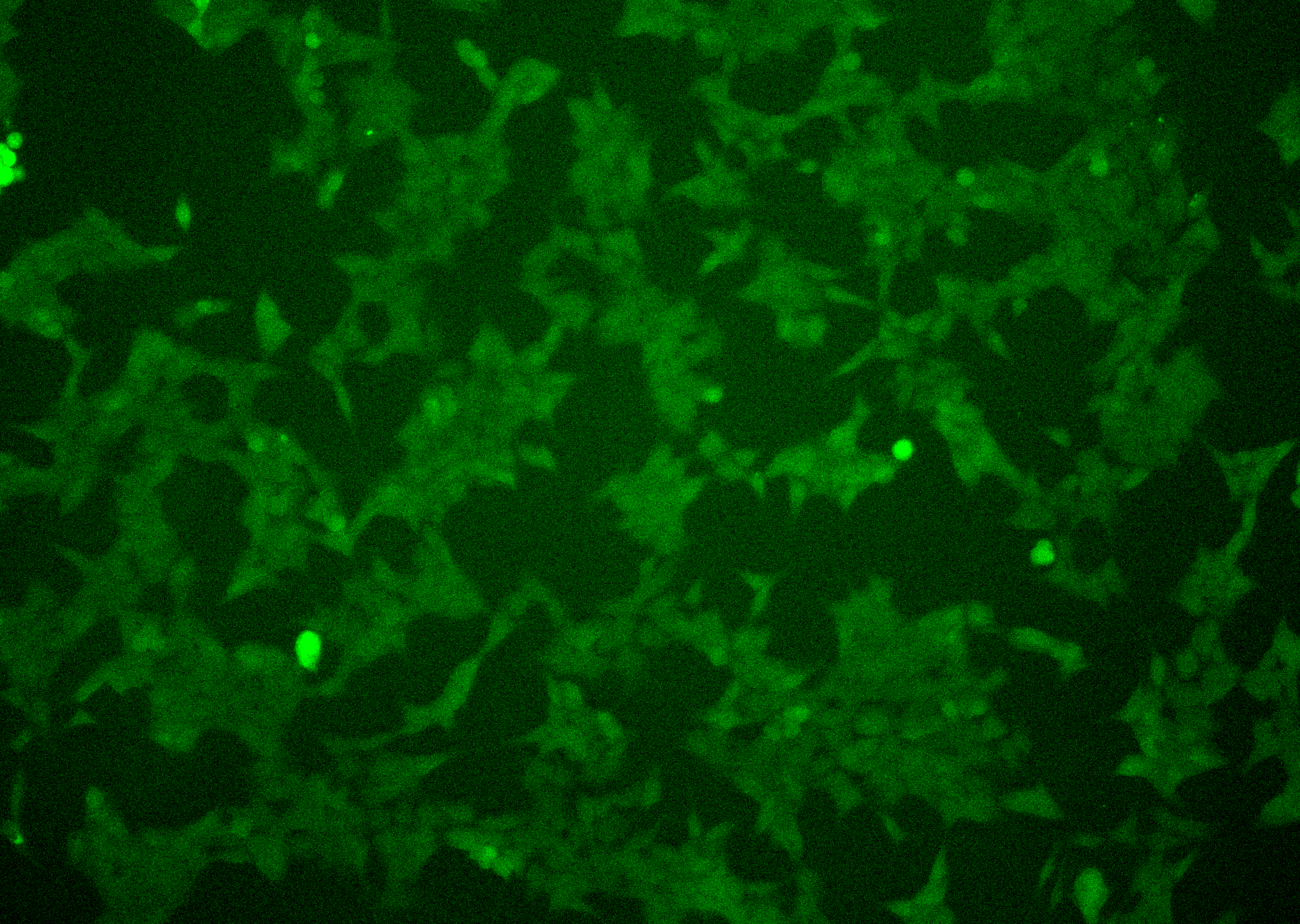

Supplement: Supplementary file 7 — Source data Fig. 5 [file 44321_2025_308_MOESM7_ESM.zip › Figure 5/5a/116 NAC+3.png]

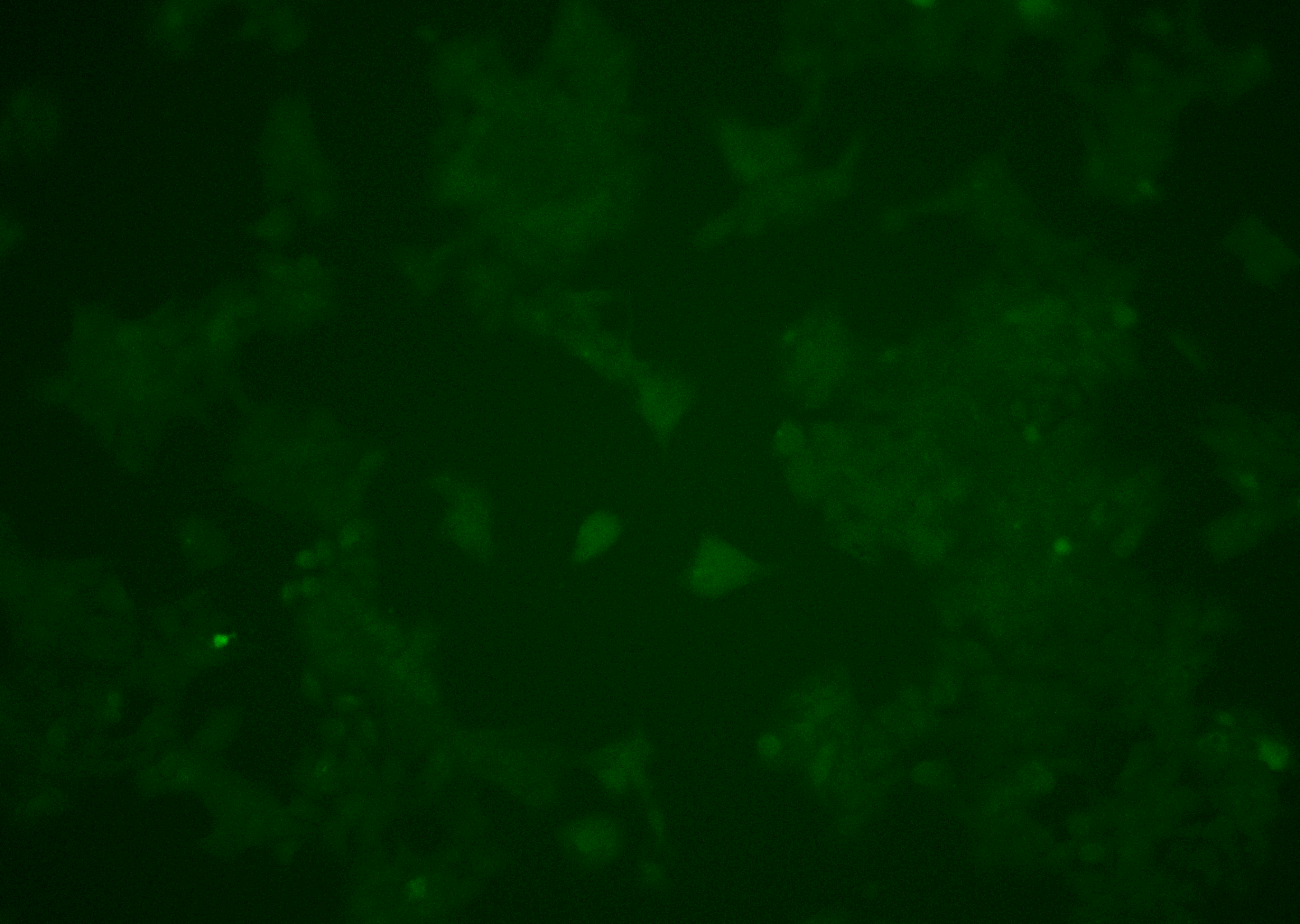

Supplement: Supplementary file 7 — Source data Fig. 5 [file 44321_2025_308_MOESM7_ESM.zip › Figure 5/5a/116 NAC.png]

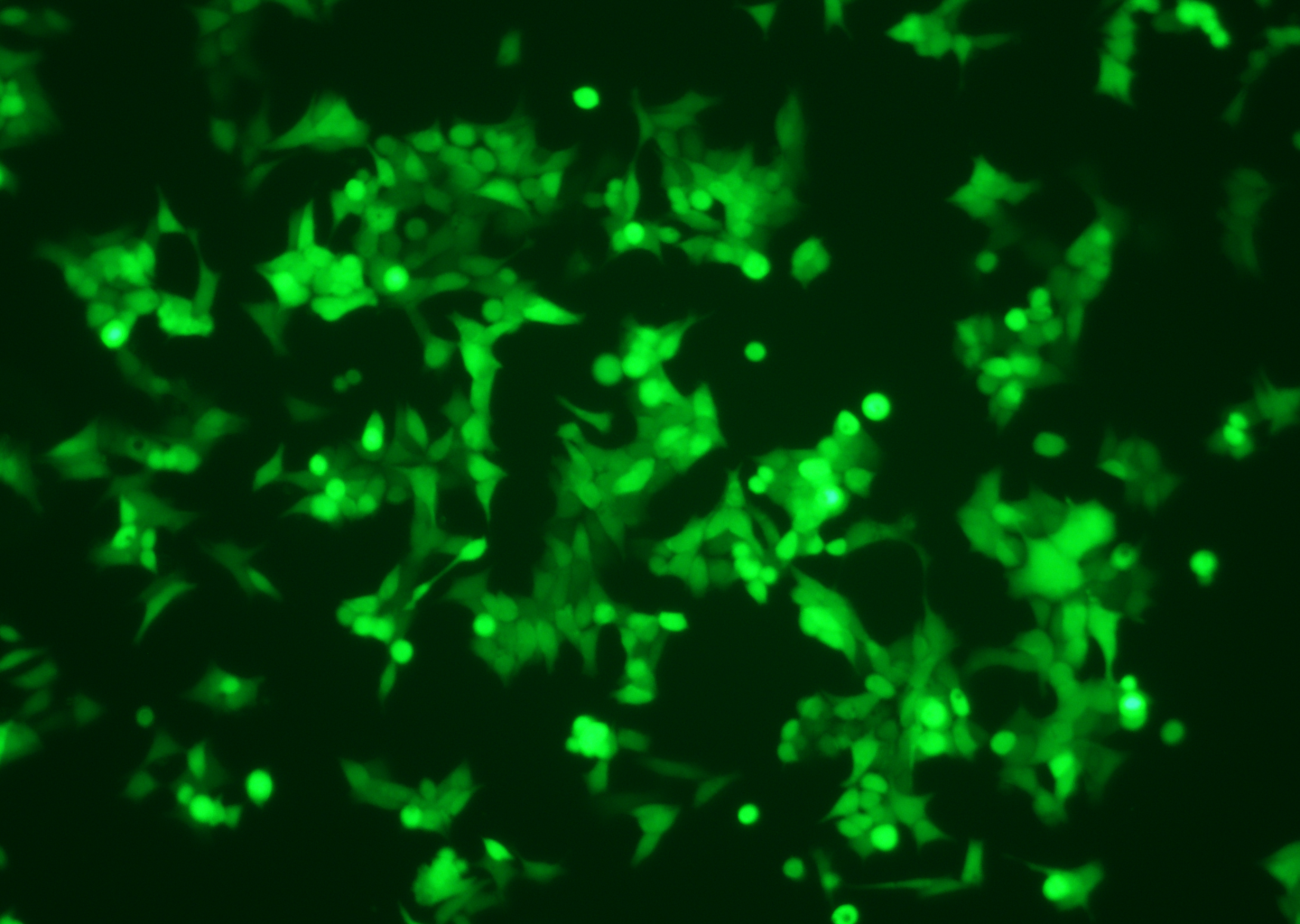

Supplement: Supplementary file 7 — Source data Fig. 5 [file 44321_2025_308_MOESM7_ESM.zip › Figure 5/5a/166 3.png]

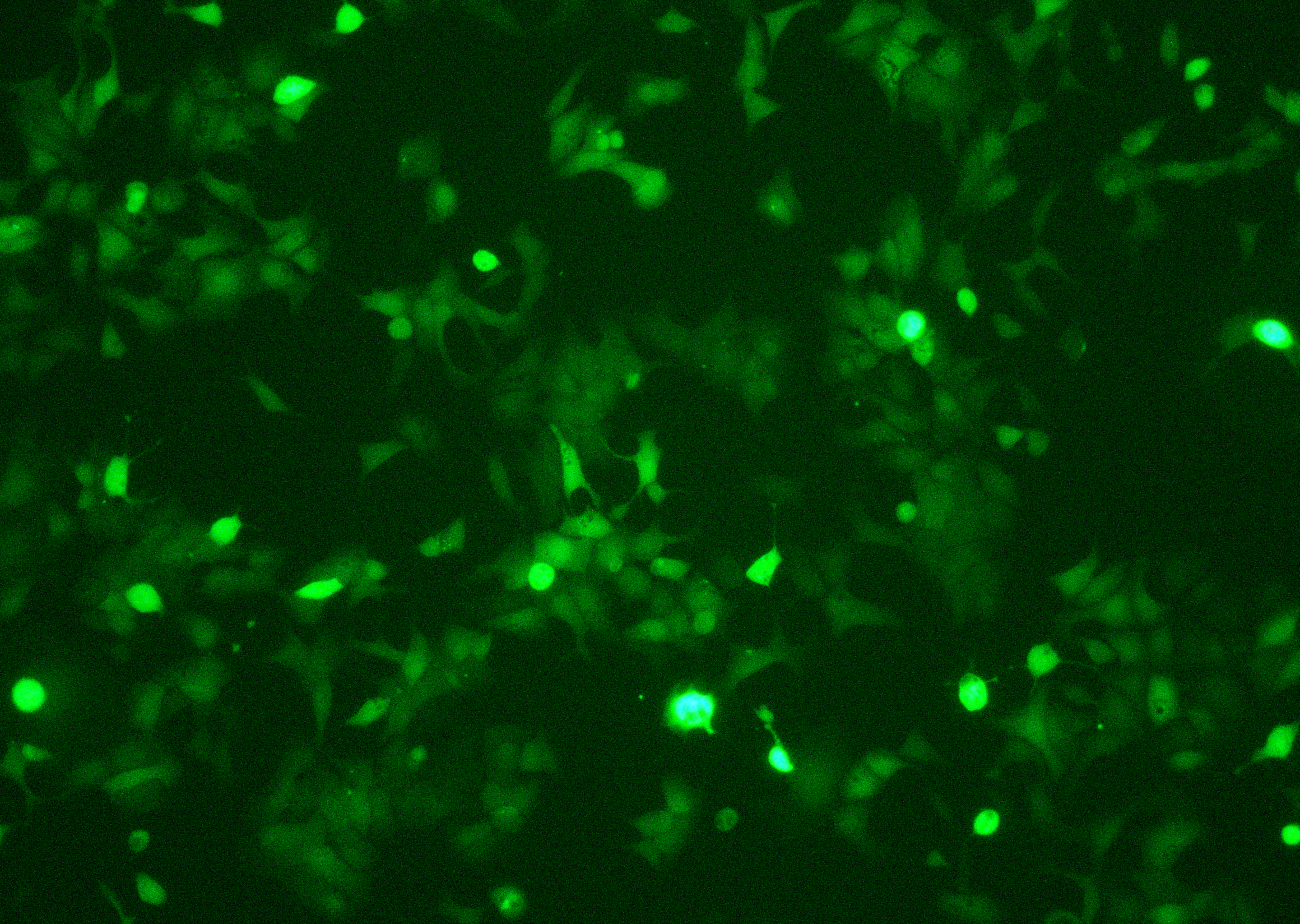

Supplement: Supplementary file 7 — Source data Fig. 5 [file 44321_2025_308_MOESM7_ESM.zip › Figure 5/5a/DLD1 1.5.png]

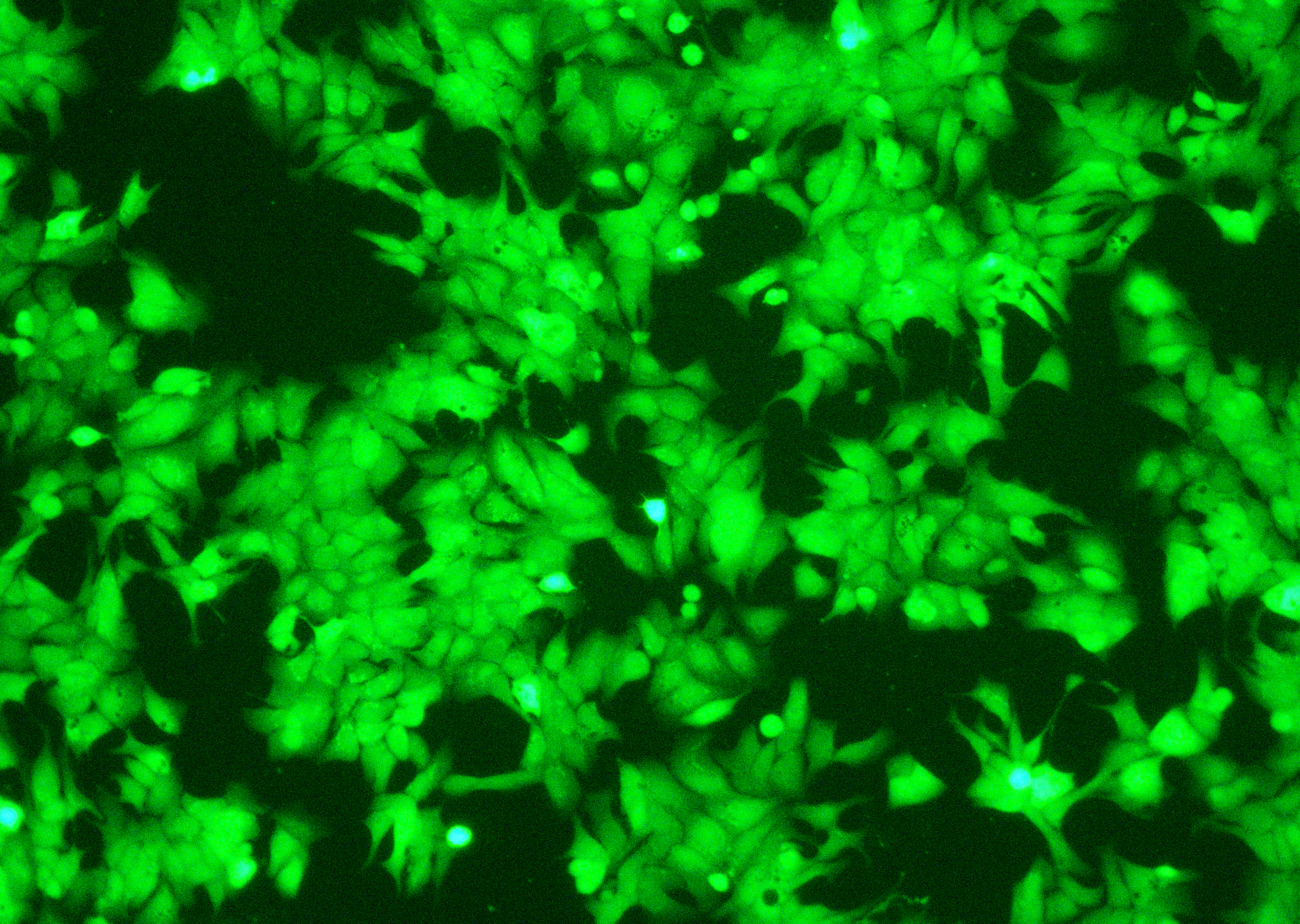

Supplement: Supplementary file 7 — Source data Fig. 5 [file 44321_2025_308_MOESM7_ESM.zip › Figure 5/5a/DLD1 3.png]

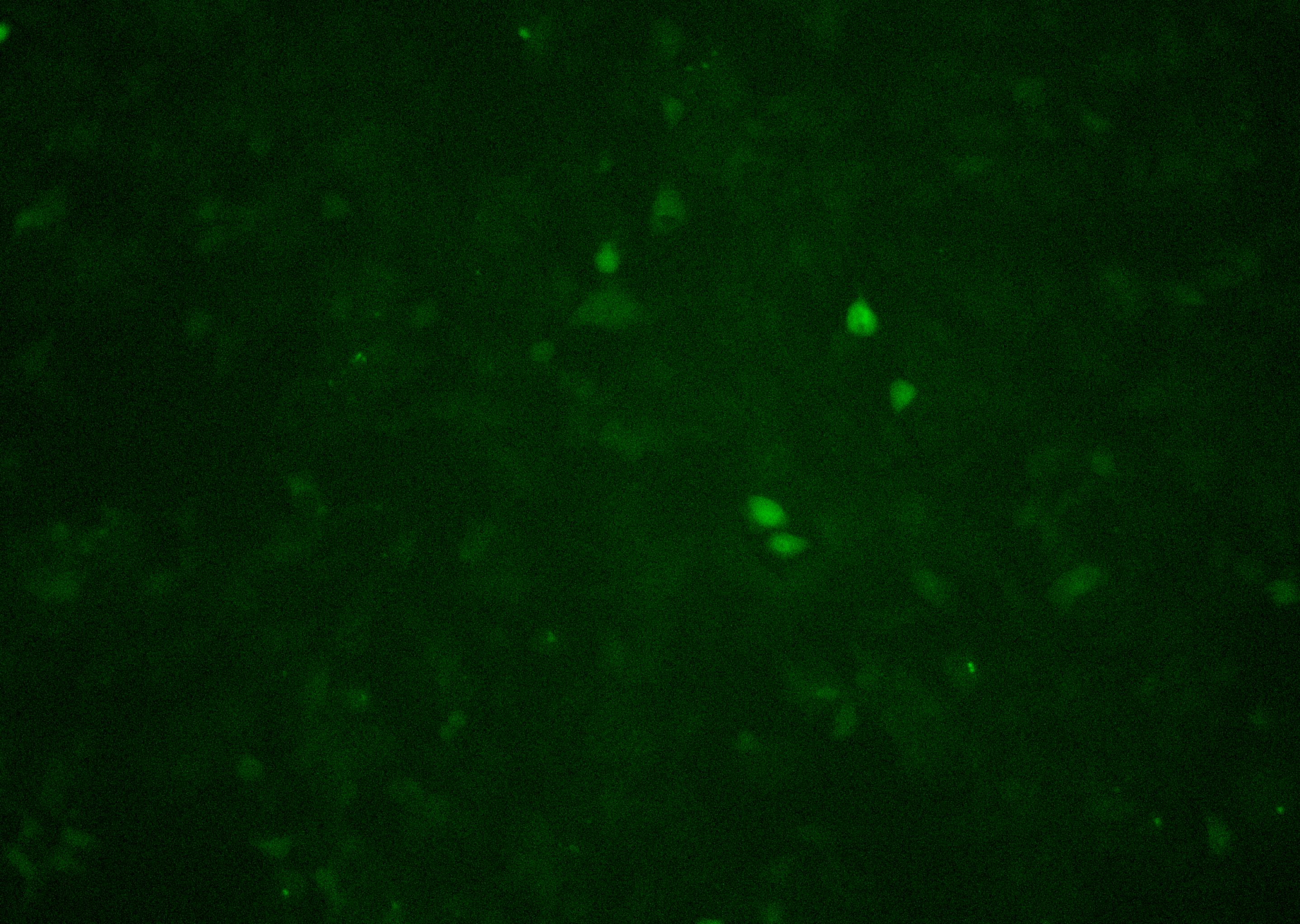

Supplement: Supplementary file 7 — Source data Fig. 5 [file 44321_2025_308_MOESM7_ESM.zip › Figure 5/5a/DLD1 CON.png]

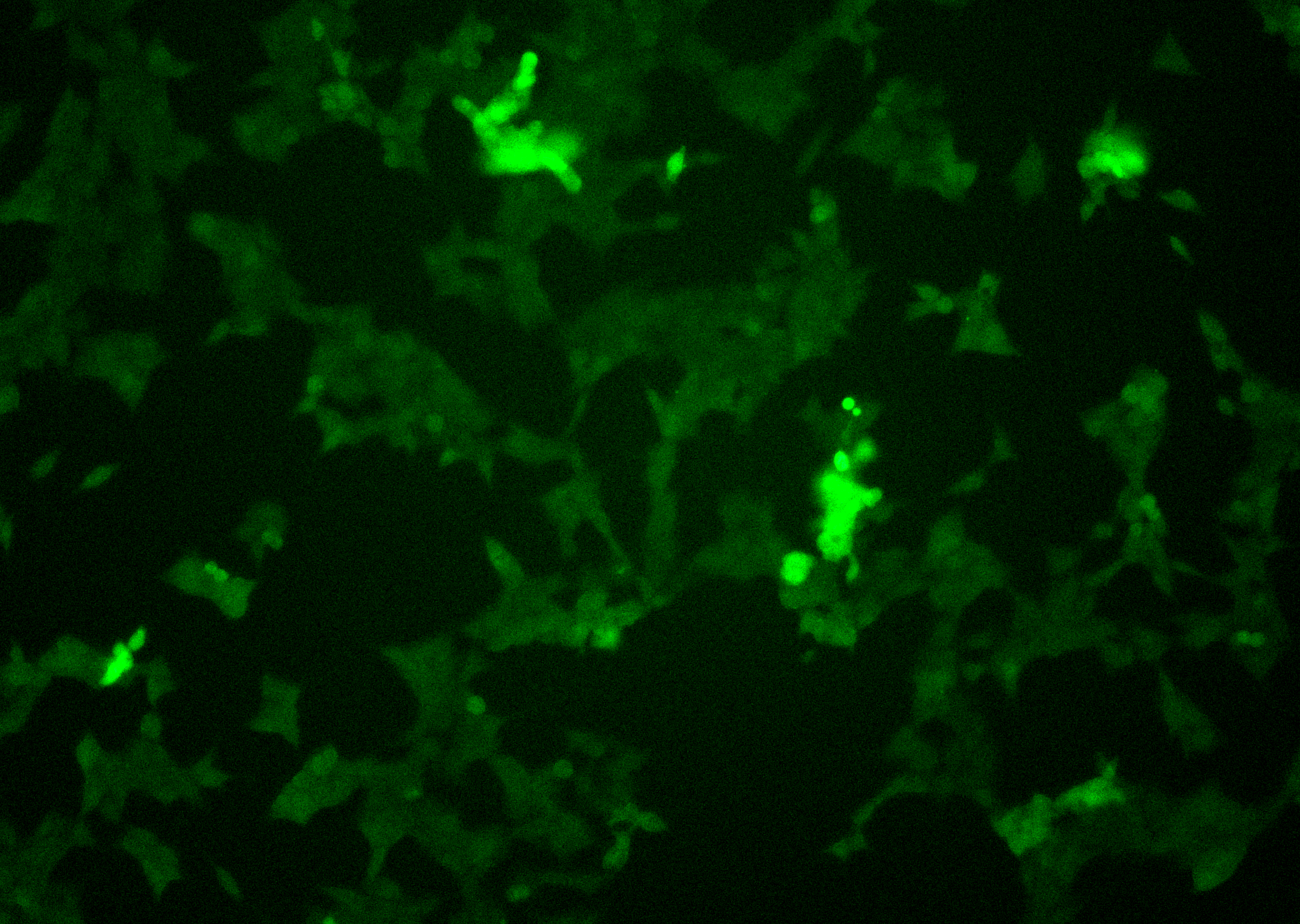

Supplement: Supplementary file 7 — Source data Fig. 5 [file 44321_2025_308_MOESM7_ESM.zip › Figure 5/5a/DLD1 NAC+3.png]

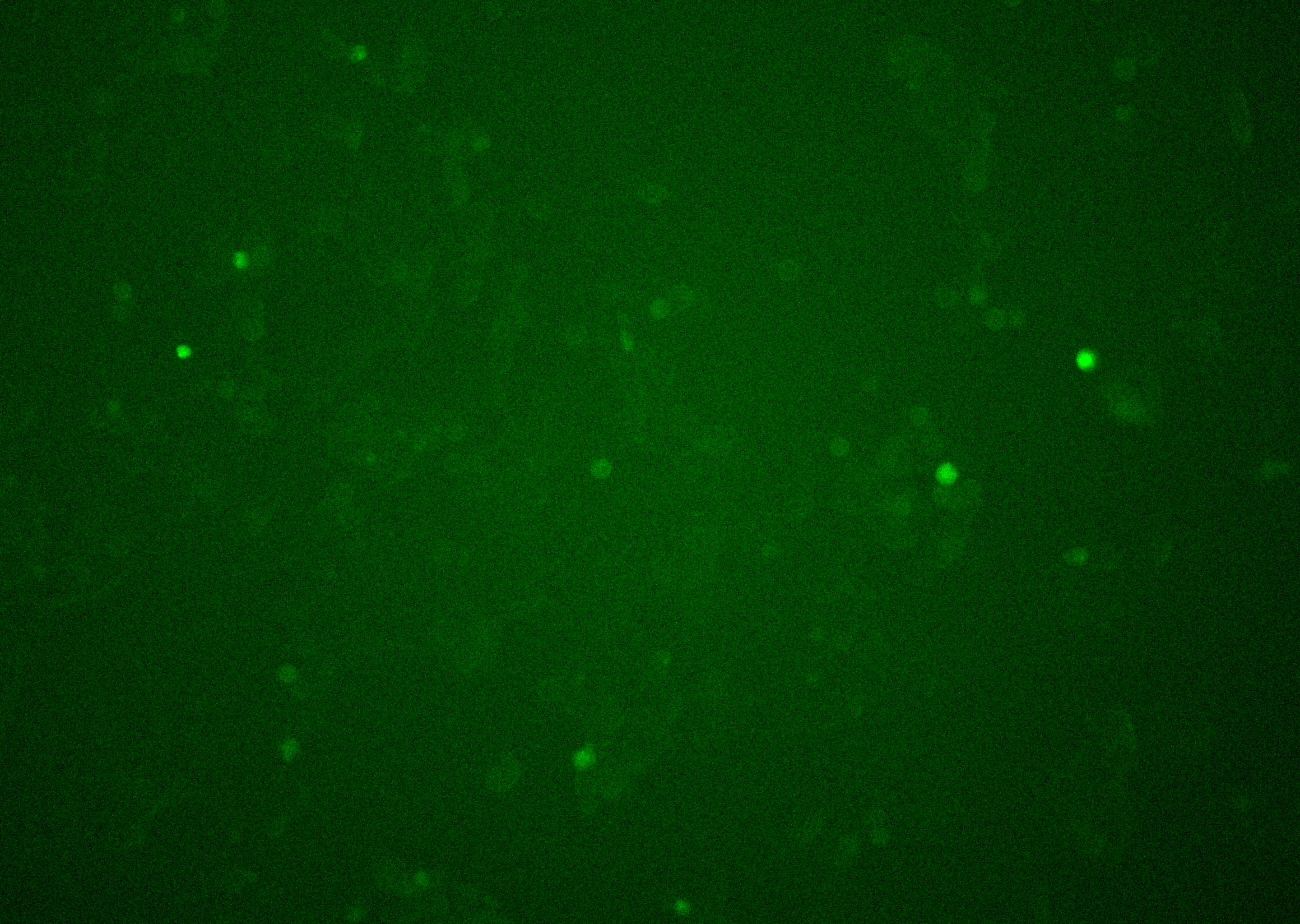

Supplement: Supplementary file 7 — Source data Fig. 5 [file 44321_2025_308_MOESM7_ESM.zip › Figure 5/5a/DLD1 NAC.png]

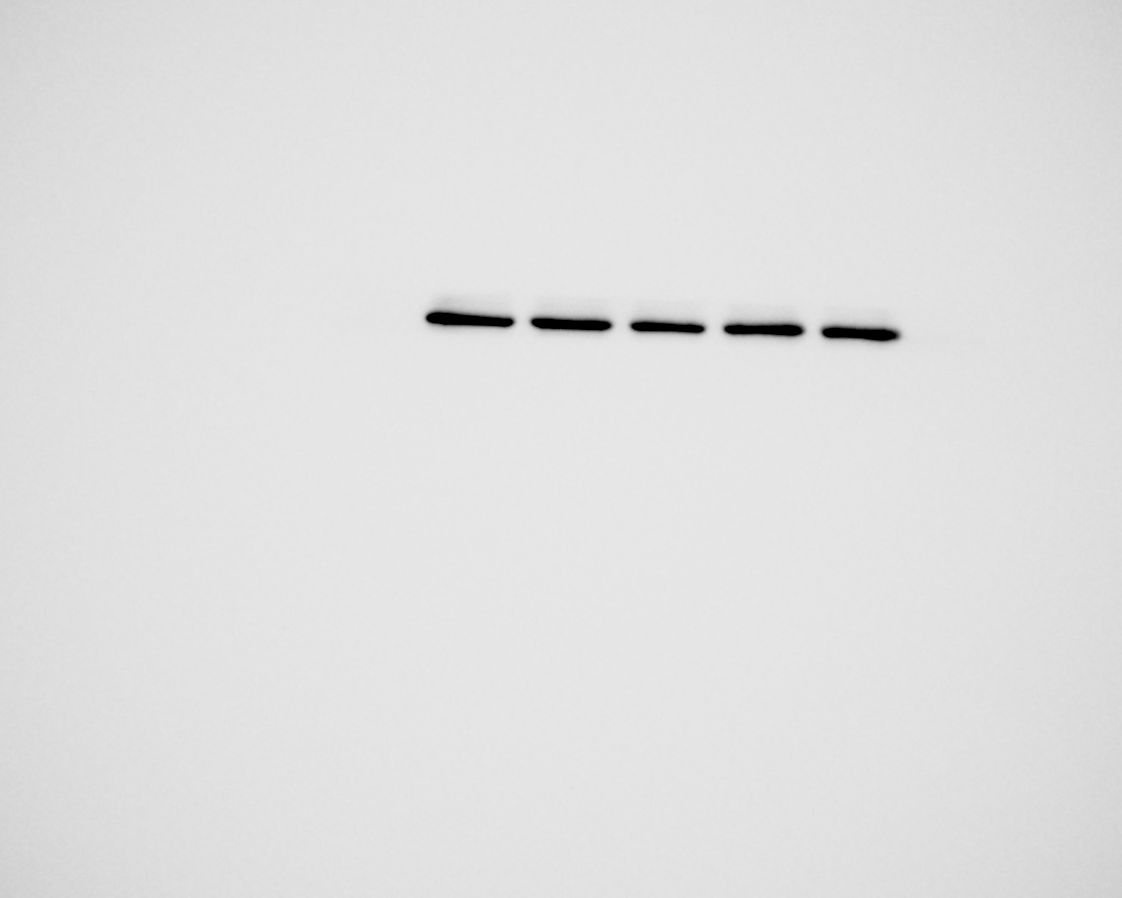

Supplement: Supplementary file 7 — Source data Fig. 5 [file 44321_2025_308_MOESM7_ESM.zip › Figure 5/5c/western actin 1.tif]

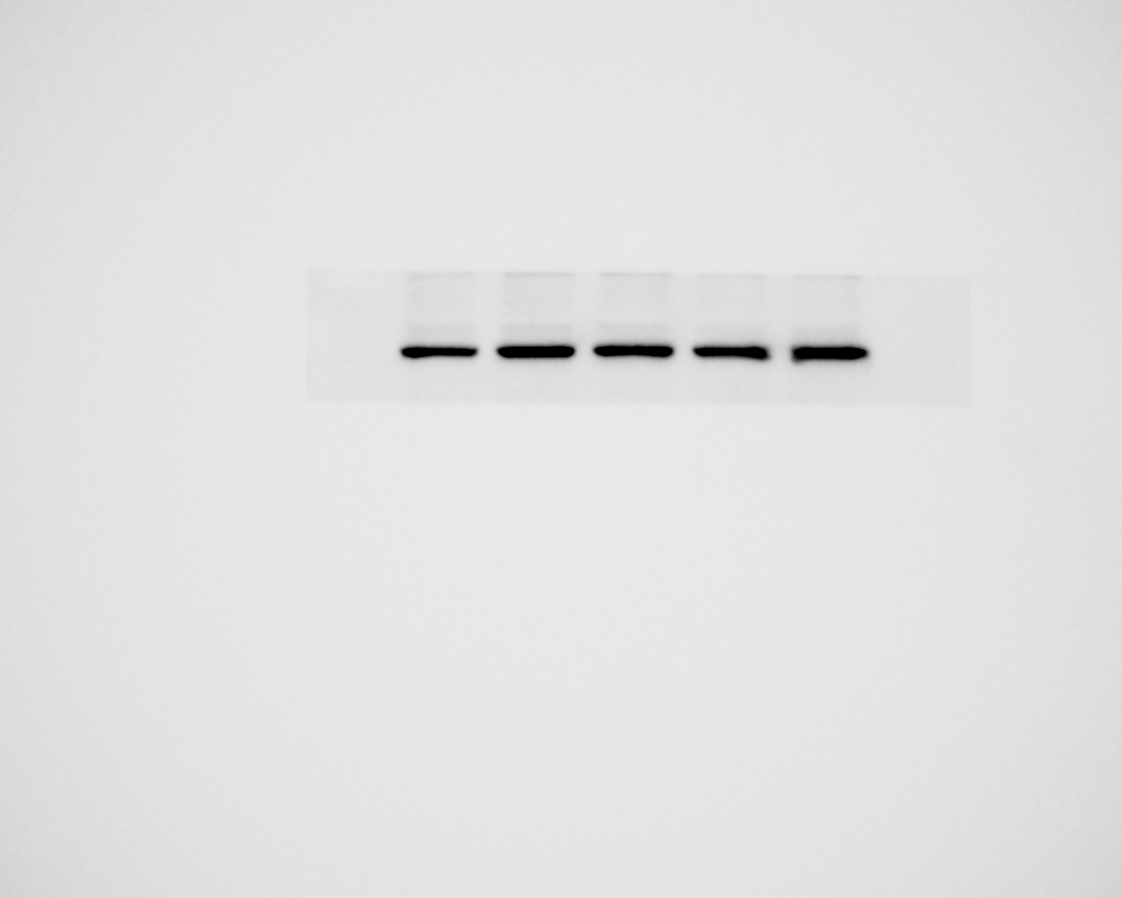

Supplement: Supplementary file 7 — Source data Fig. 5 [file 44321_2025_308_MOESM7_ESM.zip › Figure 5/5c/western actin 2.tif]

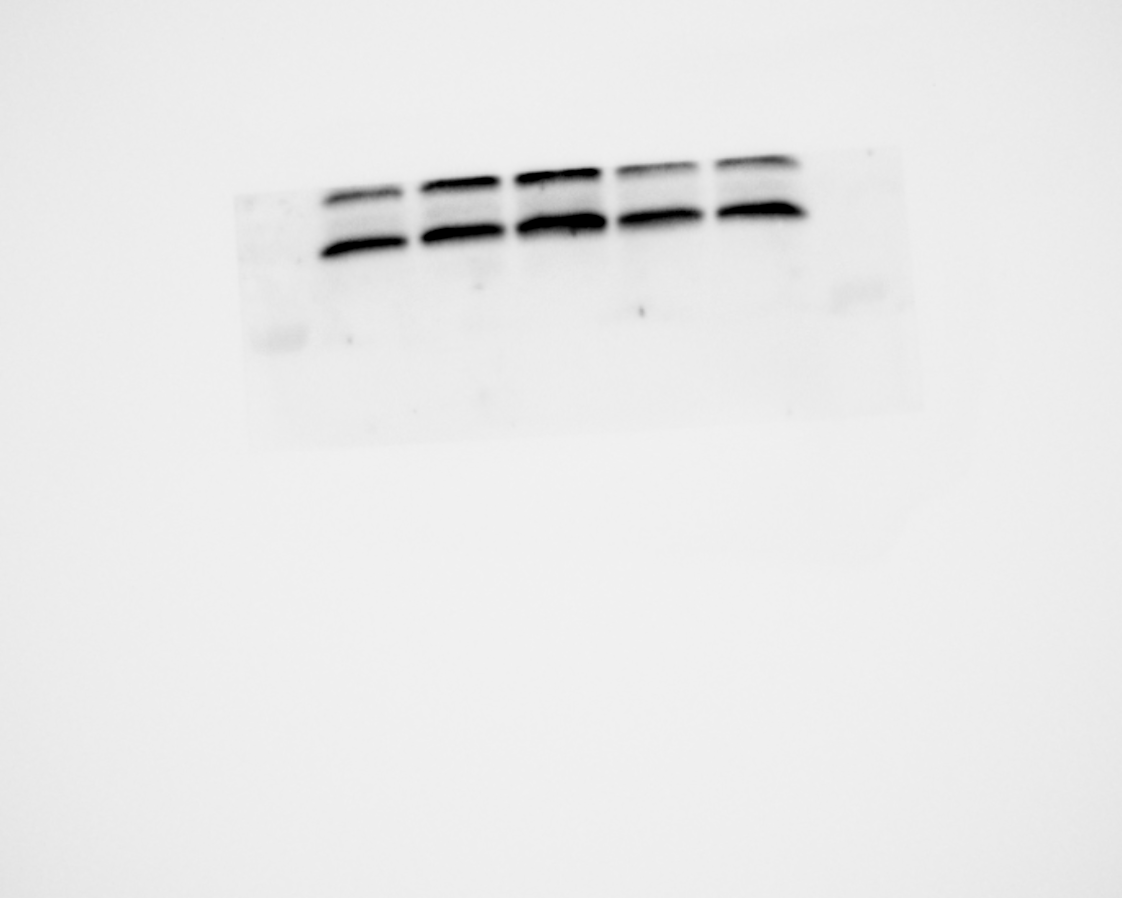

Supplement: Supplementary file 7 — Source data Fig. 5 [file 44321_2025_308_MOESM7_ESM.zip › Figure 5/5c/western bax 1.tif]

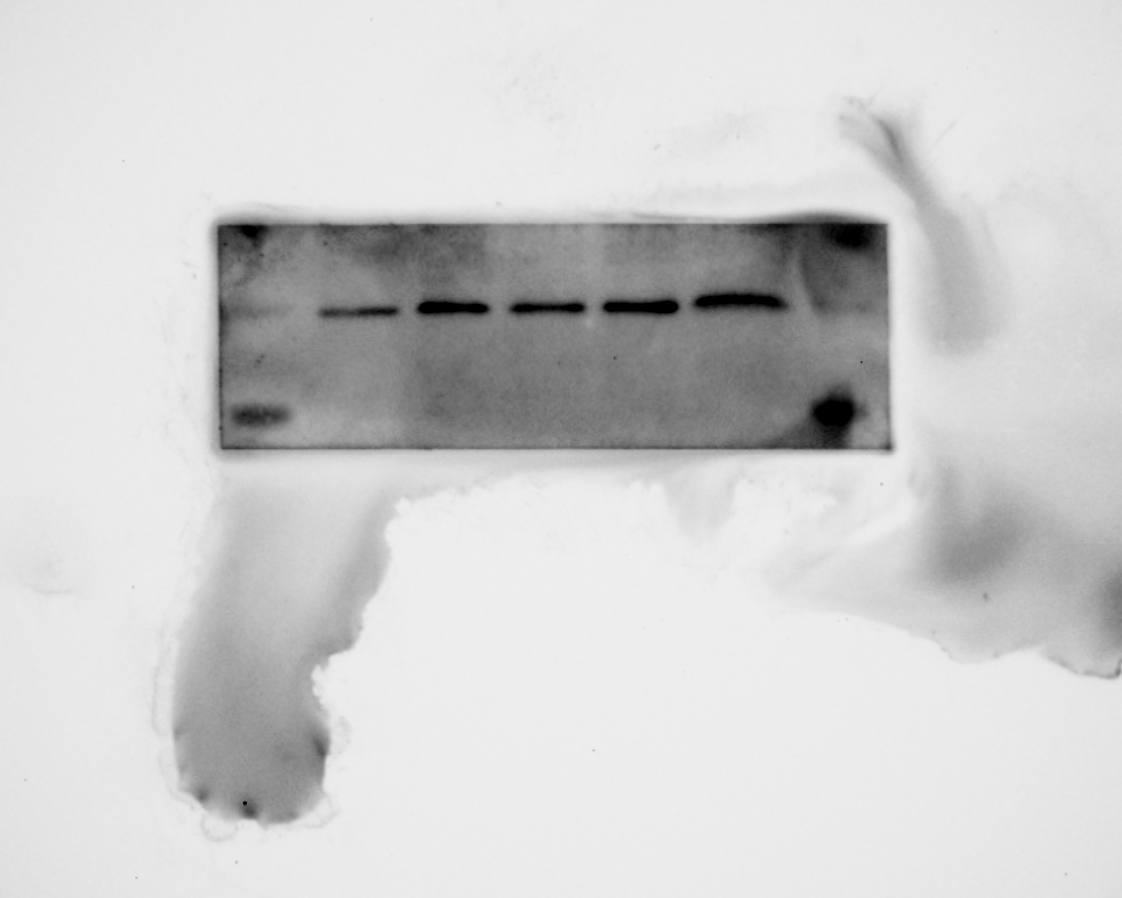

Supplement: Supplementary file 7 — Source data Fig. 5 [file 44321_2025_308_MOESM7_ESM.zip › Figure 5/5c/western bax 2 (2).tif]

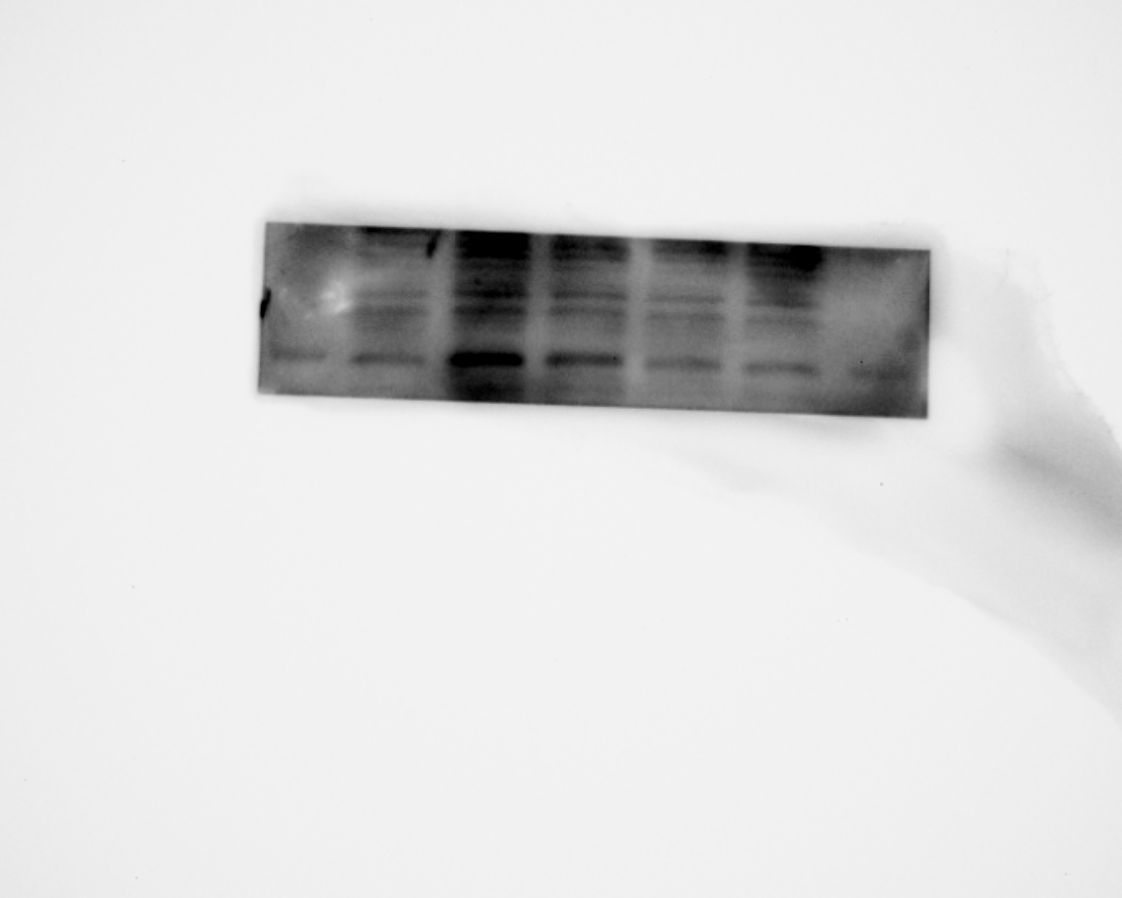

Supplement: Supplementary file 7 — Source data Fig. 5 [file 44321_2025_308_MOESM7_ESM.zip › Figure 5/5c/western bax 2.tif]

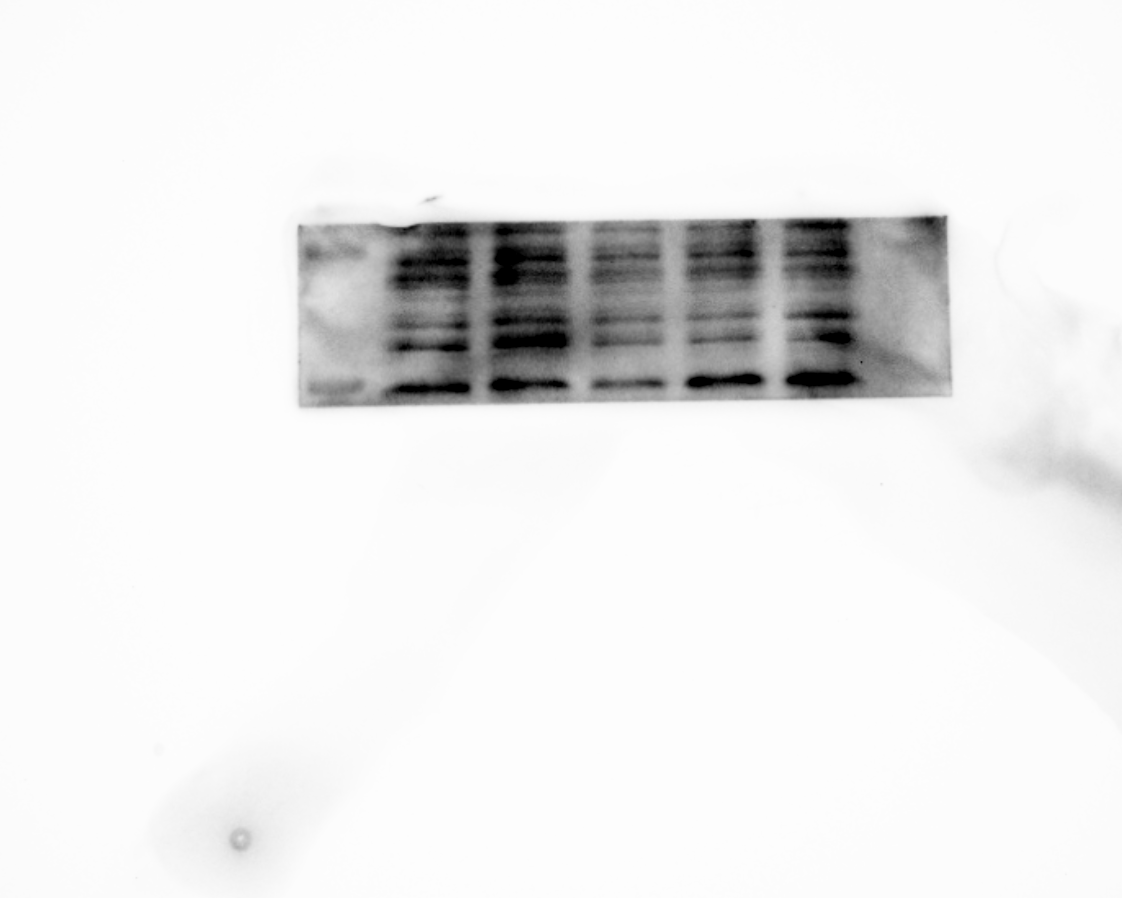

Supplement: Supplementary file 7 — Source data Fig. 5 [file 44321_2025_308_MOESM7_ESM.zip › Figure 5/5c/western bcl 1.tif]

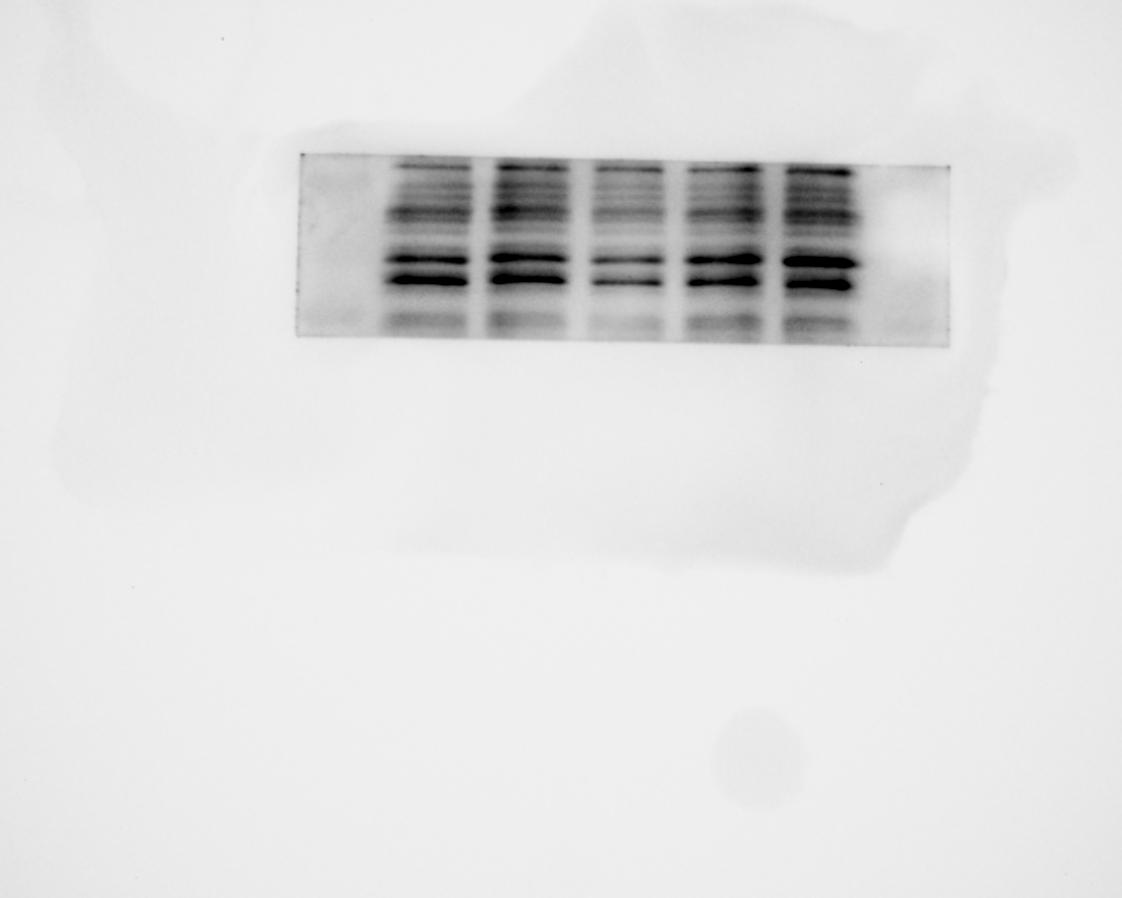

Supplement: Supplementary file 7 — Source data Fig. 5 [file 44321_2025_308_MOESM7_ESM.zip › Figure 5/5c/western bcl 2.tif]

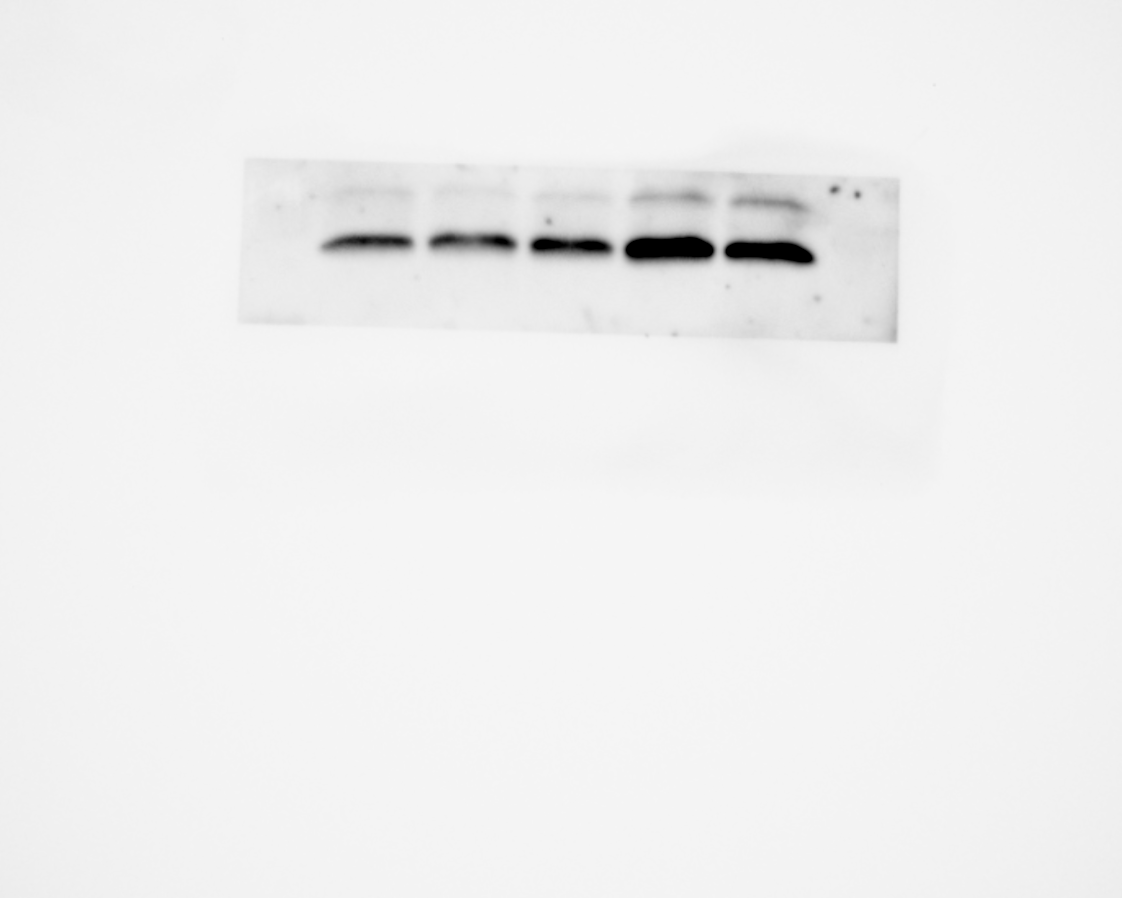

Supplement: Supplementary file 7 — Source data Fig. 5 [file 44321_2025_308_MOESM7_ESM.zip › Figure 5/5c/western lc3b 1.tif]

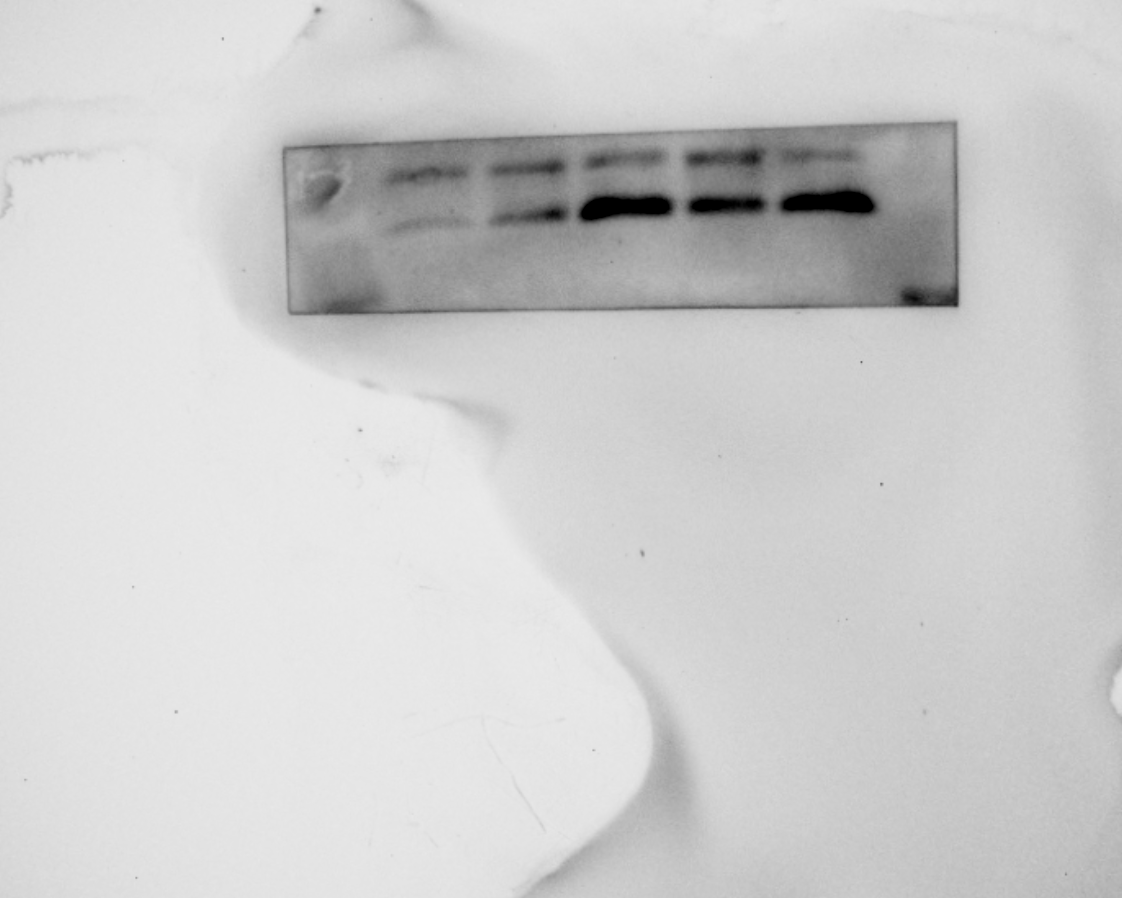

Supplement: Supplementary file 7 — Source data Fig. 5 [file 44321_2025_308_MOESM7_ESM.zip › Figure 5/5c/western lc3b 2.tif]

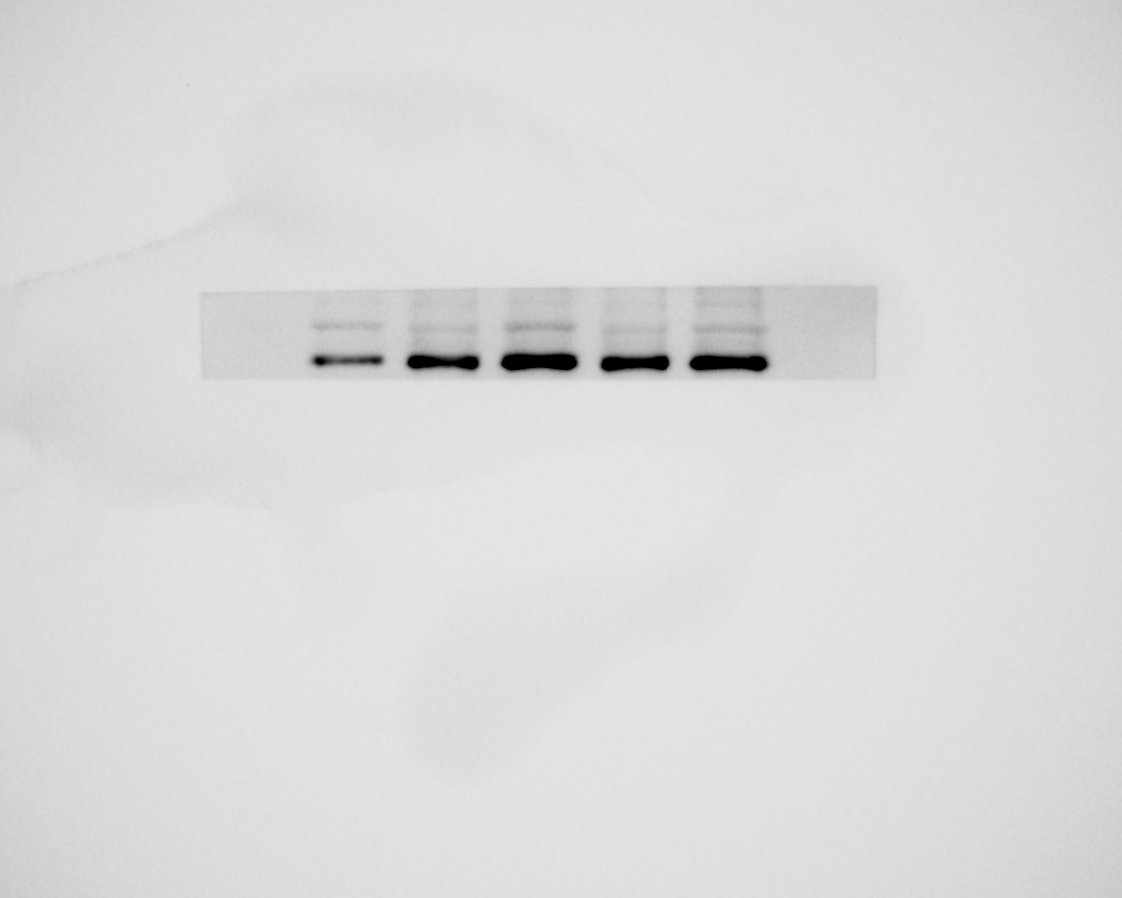

Supplement: Supplementary file 7 — Source data Fig. 5 [file 44321_2025_308_MOESM7_ESM.zip › Figure 5/5c/western p62 1.tif]

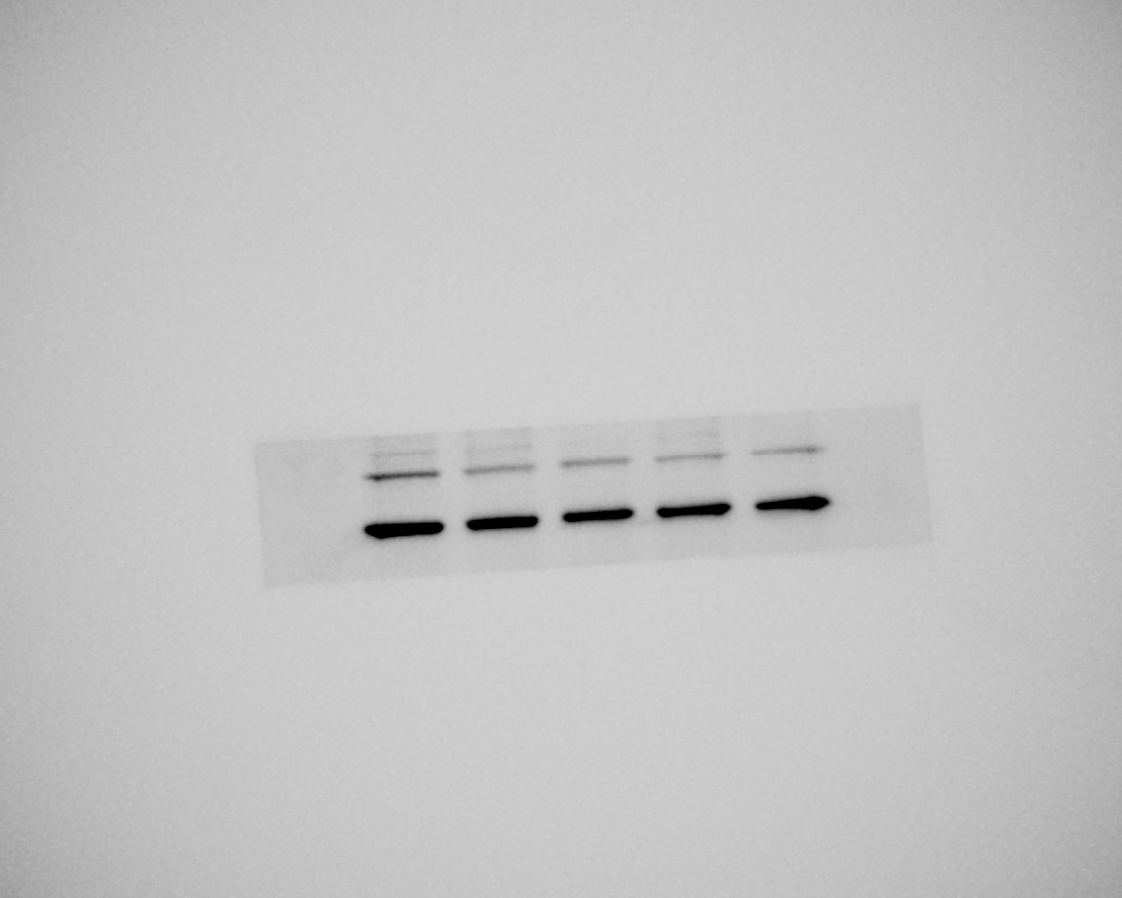

Supplement: Supplementary file 7 — Source data Fig. 5 [file 44321_2025_308_MOESM7_ESM.zip › Figure 5/5d/western actin 1.tif]

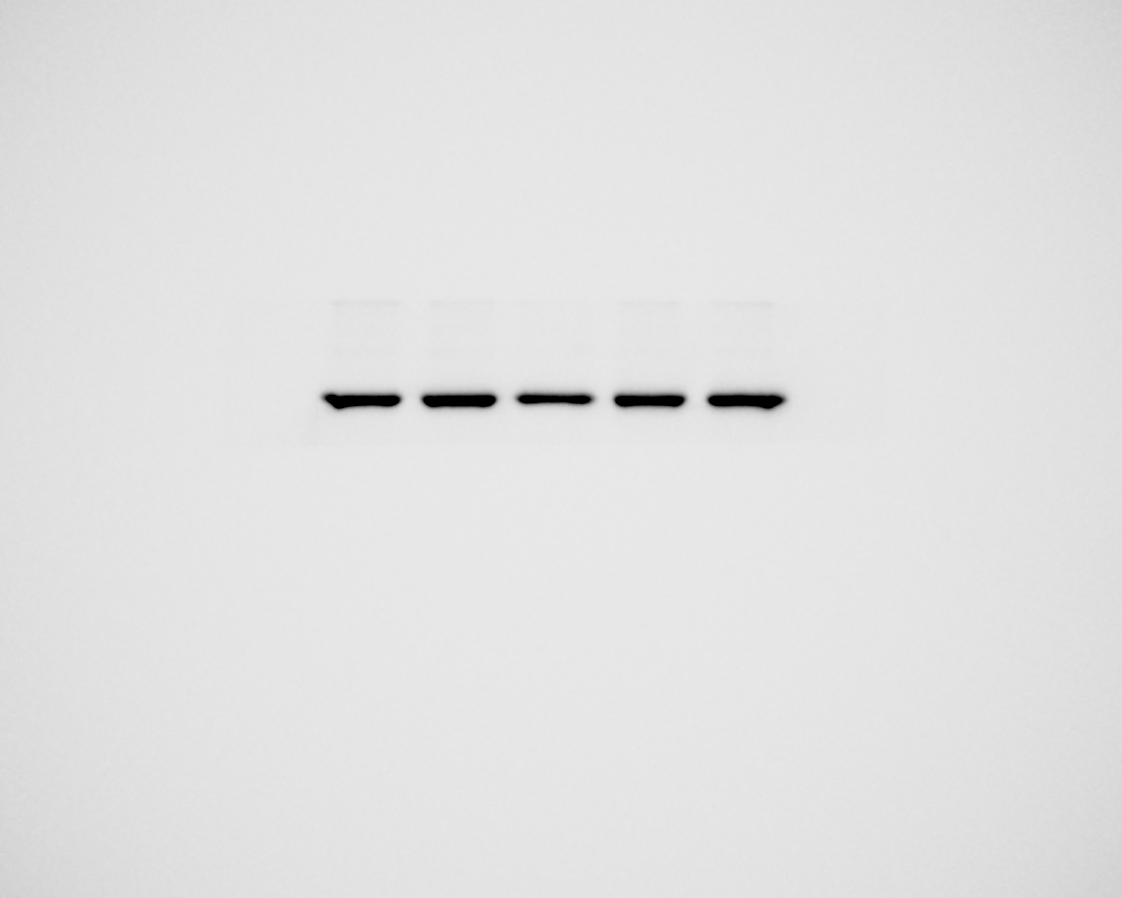

Supplement: Supplementary file 7 — Source data Fig. 5 [file 44321_2025_308_MOESM7_ESM.zip › Figure 5/5d/western actin 2.tif]

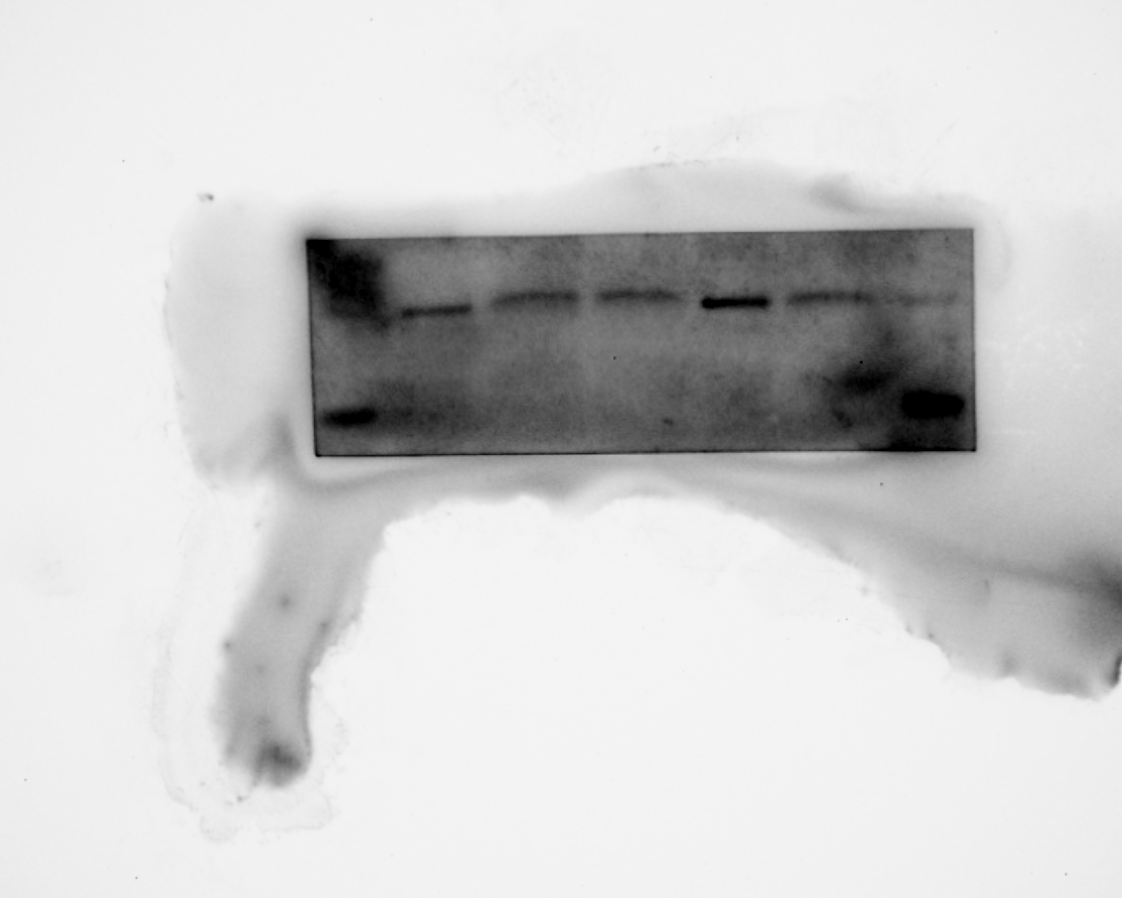

Supplement: Supplementary file 7 — Source data Fig. 5 [file 44321_2025_308_MOESM7_ESM.zip › Figure 5/5d/western bax 2.tif]

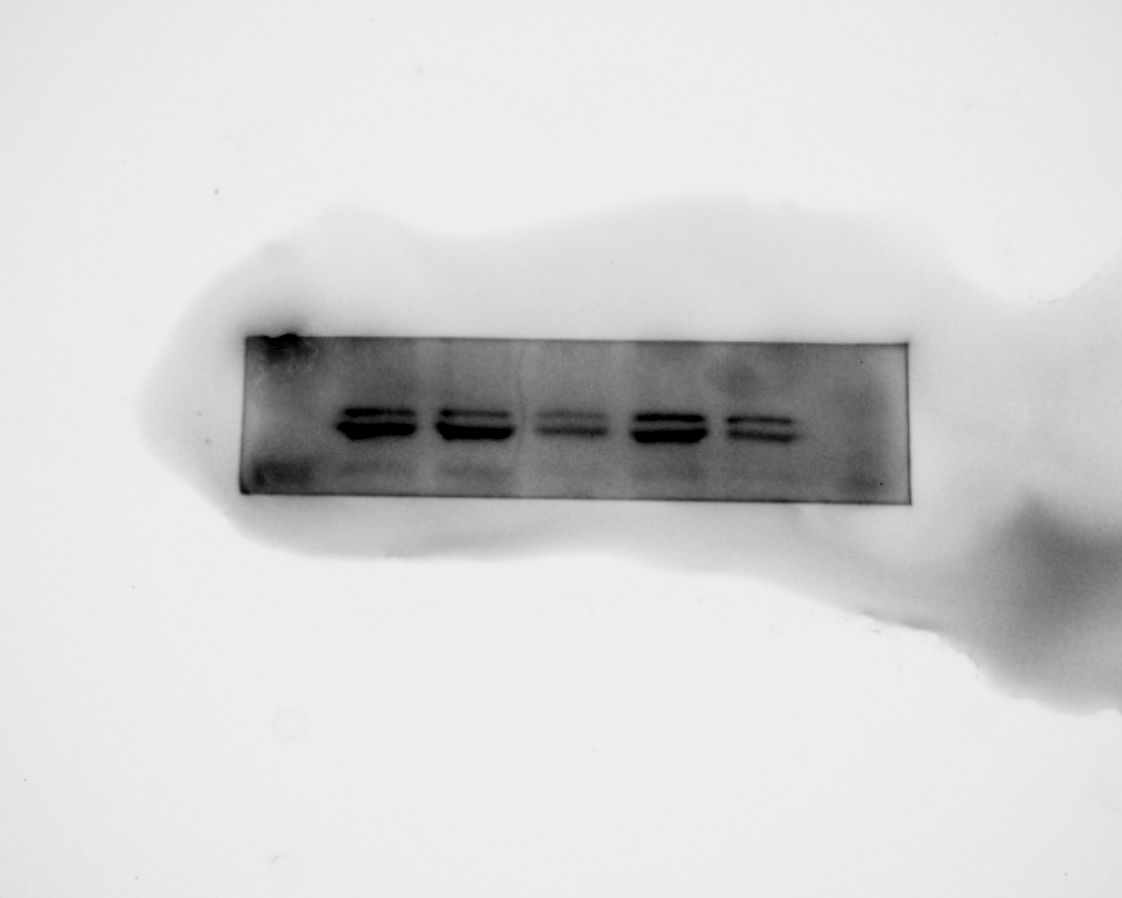

Supplement: Supplementary file 7 — Source data Fig. 5 [file 44321_2025_308_MOESM7_ESM.zip › Figure 5/5d/western bcl2 1.tif]

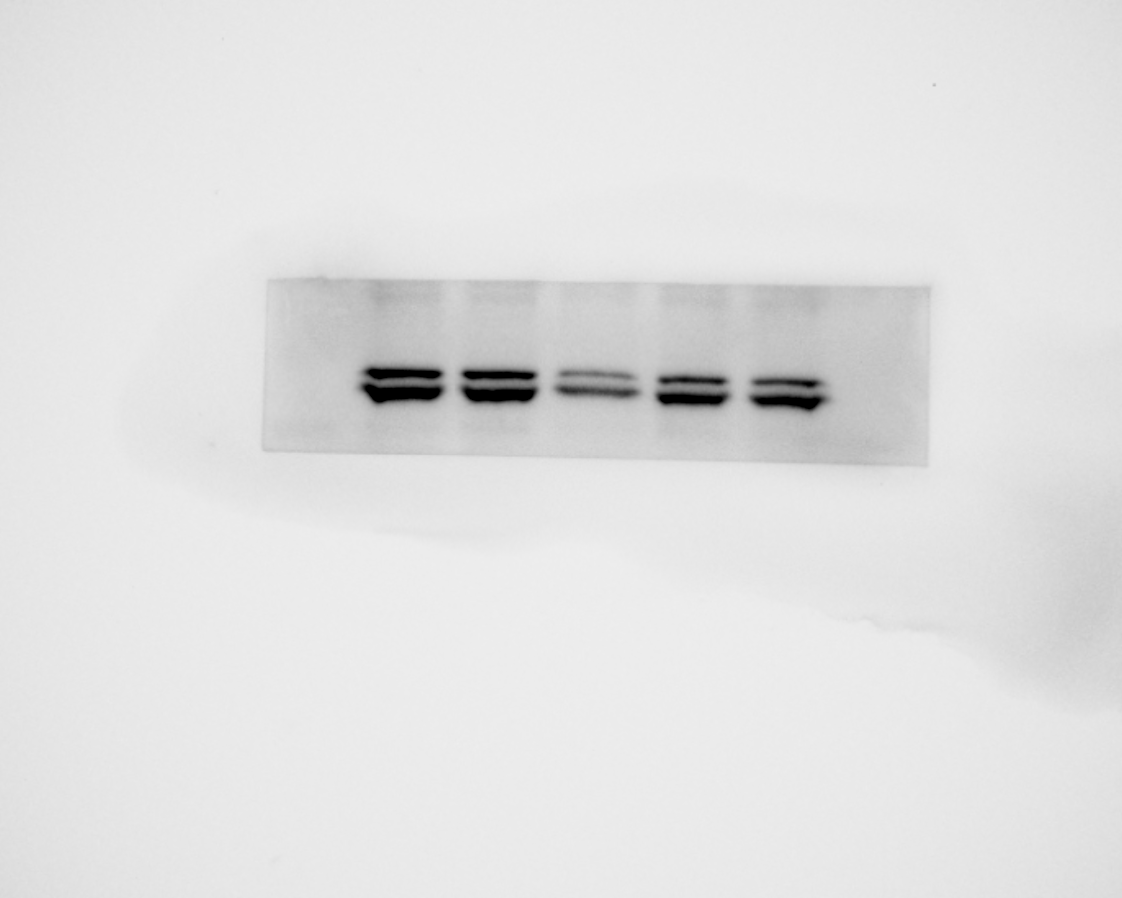

Supplement: Supplementary file 7 — Source data Fig. 5 [file 44321_2025_308_MOESM7_ESM.zip › Figure 5/5d/western bcl2 2.tif]

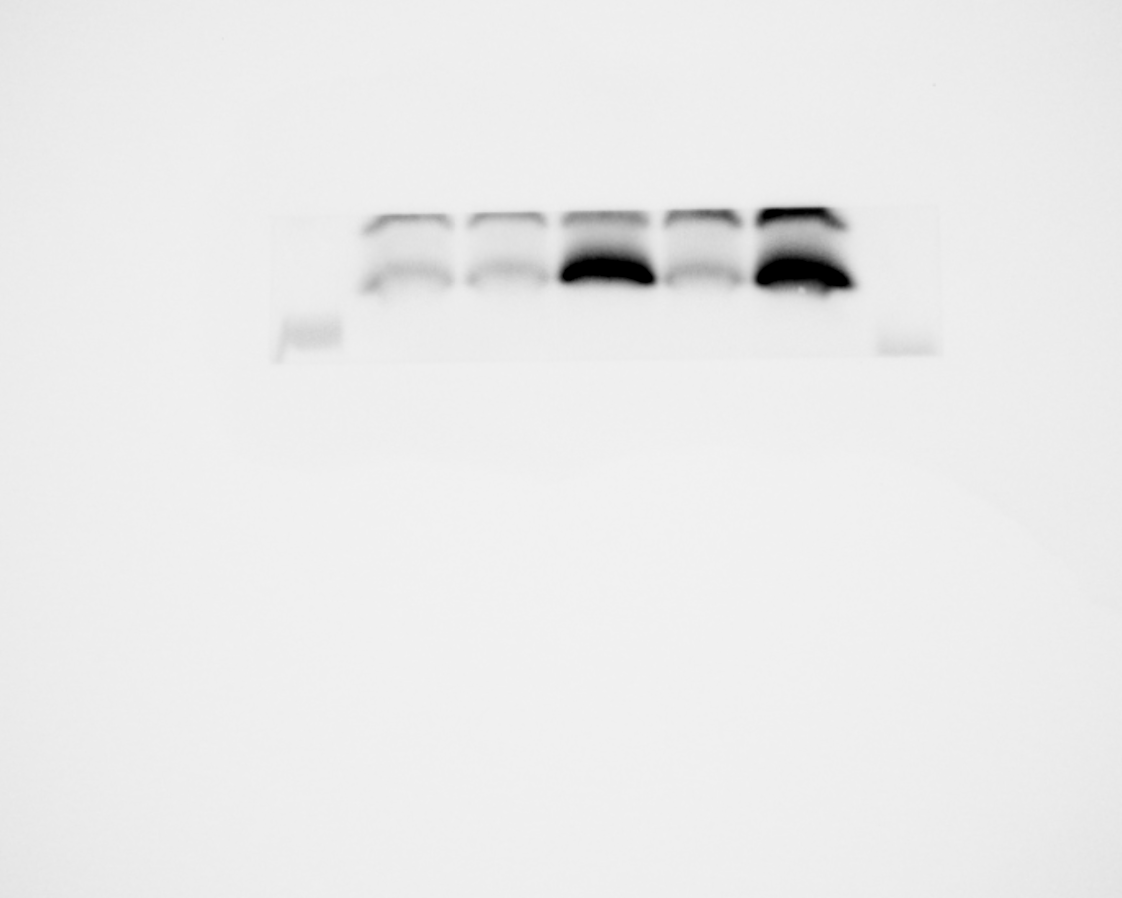

Supplement: Supplementary file 7 — Source data Fig. 5 [file 44321_2025_308_MOESM7_ESM.zip › Figure 5/5d/western lc3b 1.tif]

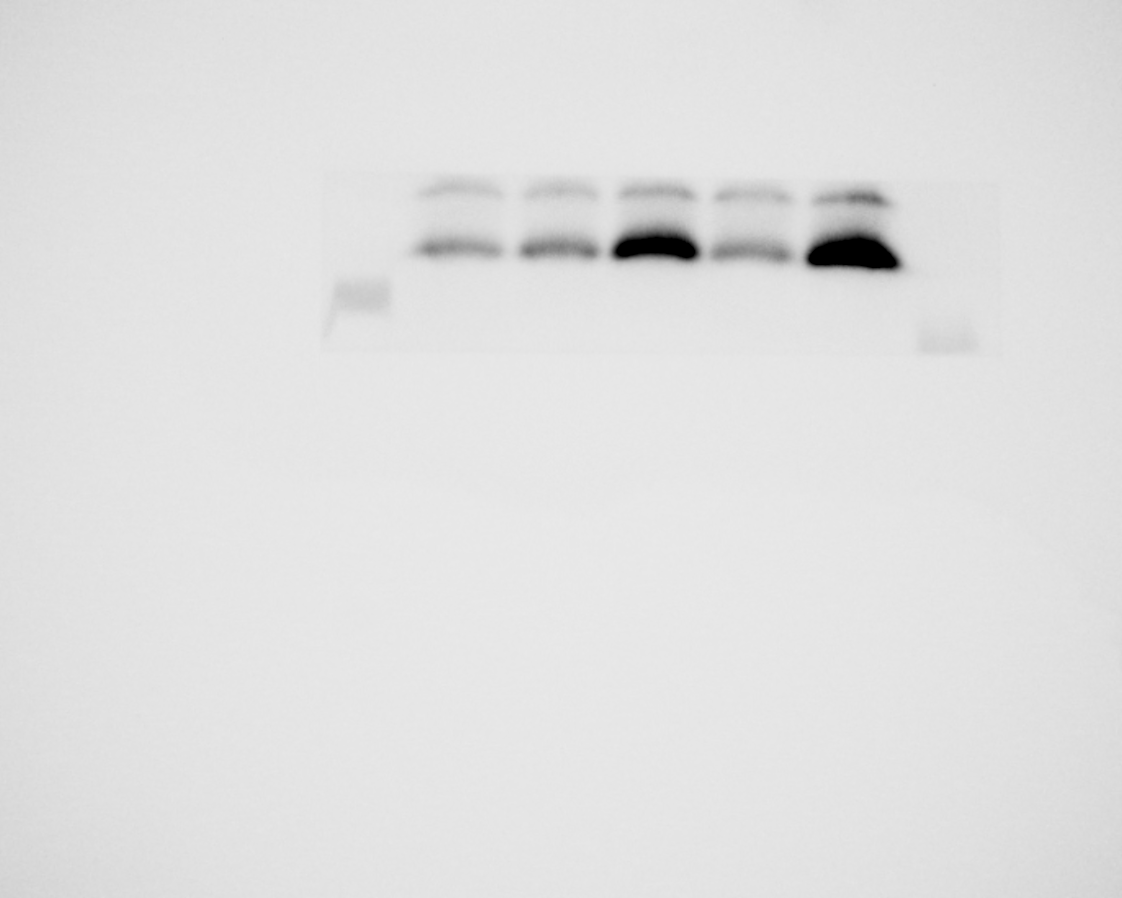

Supplement: Supplementary file 7 — Source data Fig. 5 [file 44321_2025_308_MOESM7_ESM.zip › Figure 5/5d/western lc3b 2.tif]

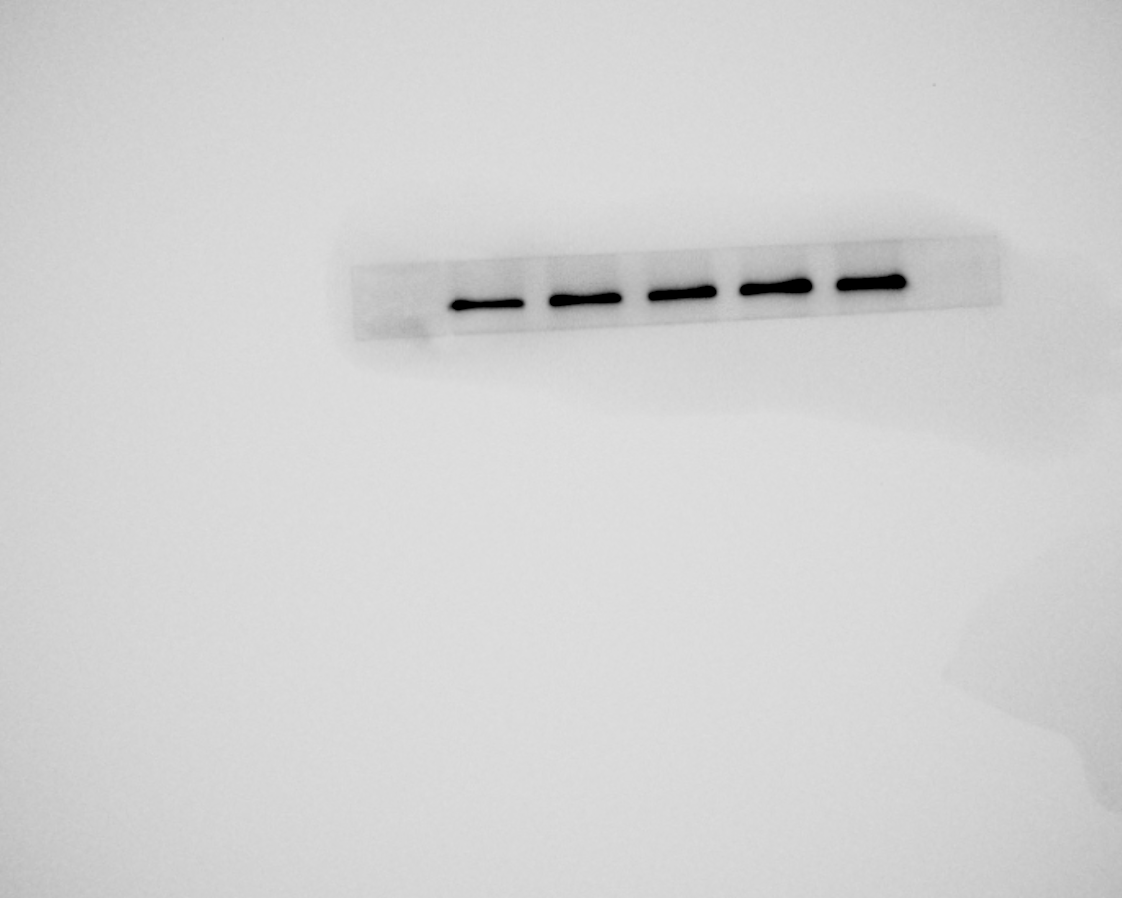

Supplement: Supplementary file 7 — Source data Fig. 5 [file 44321_2025_308_MOESM7_ESM.zip › Figure 5/5d/western p62 1.tif]

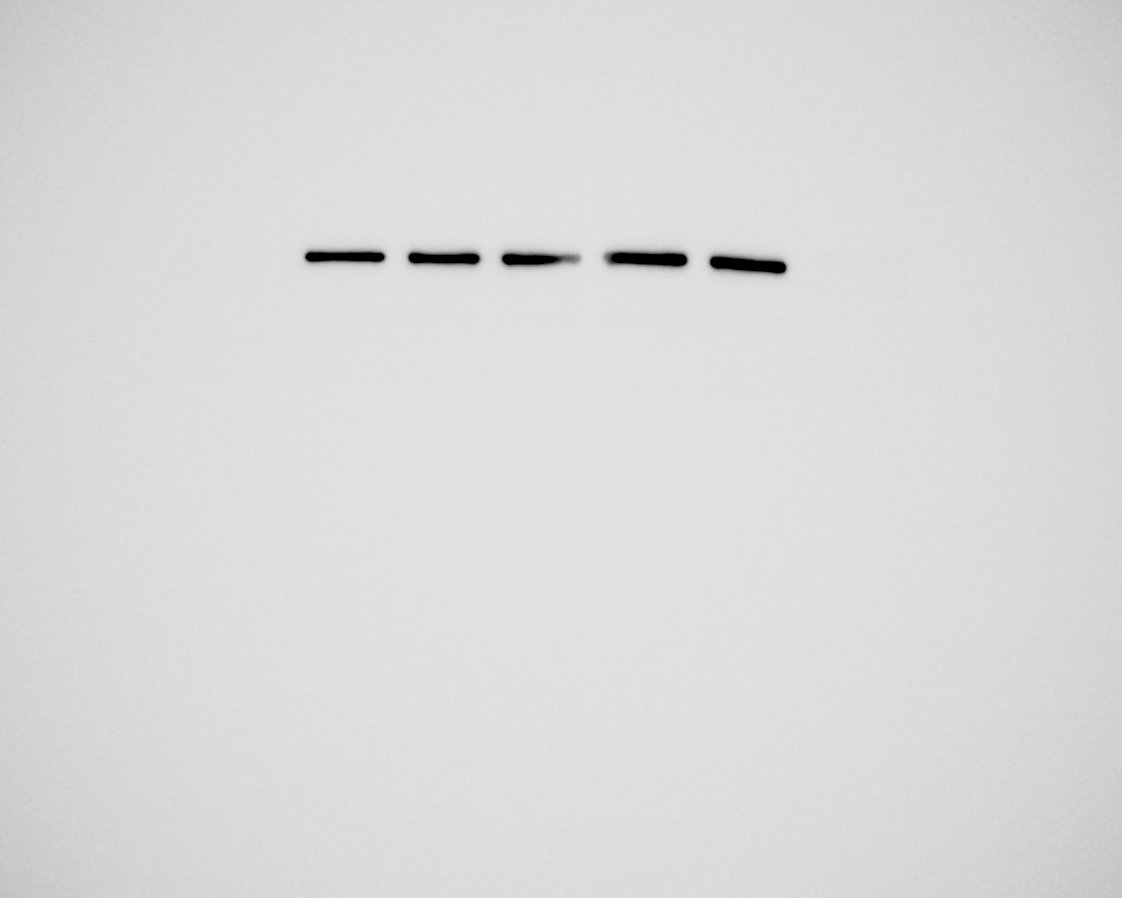

Supplement: Supplementary file 7 — Source data Fig. 5 [file 44321_2025_308_MOESM7_ESM.zip › Figure 5/5d/western p62 2.tif]

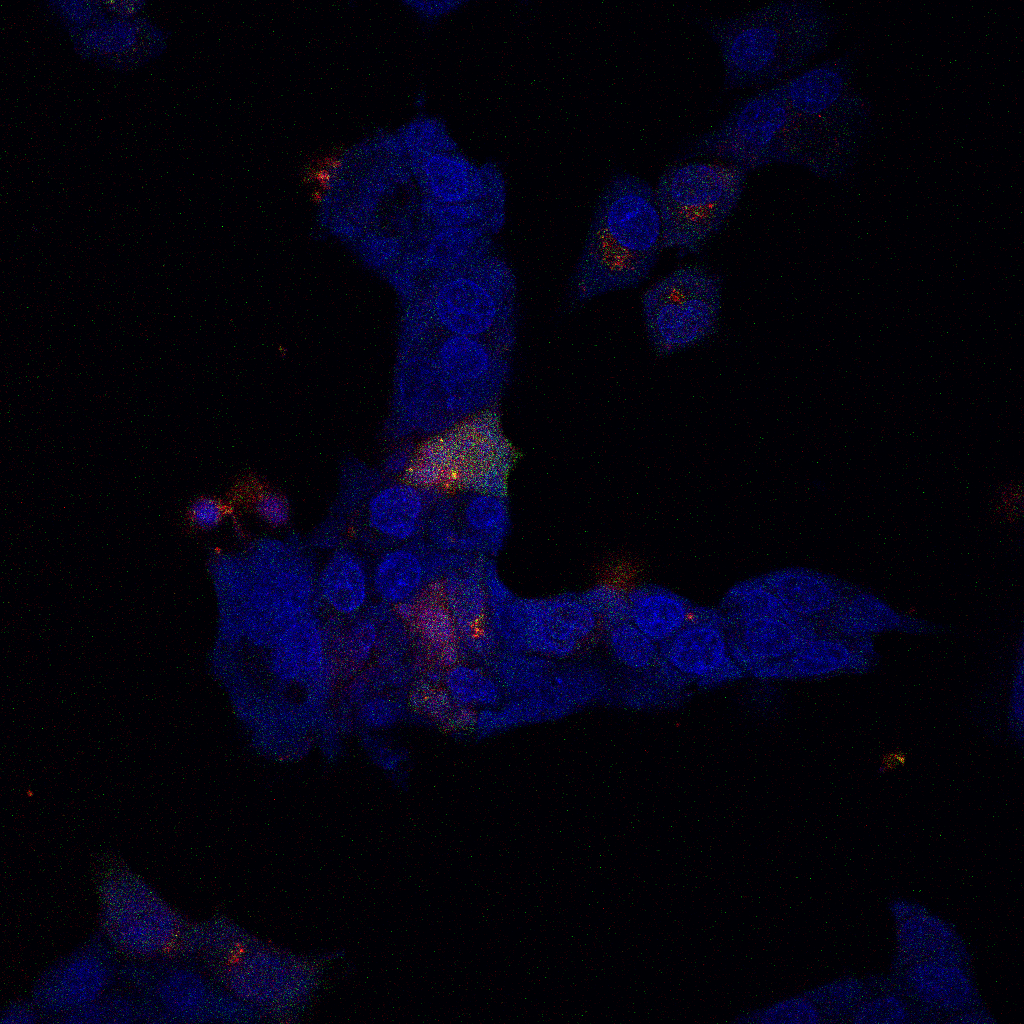

Supplement: Supplementary file 7 — Source data Fig. 5 [file 44321_2025_308_MOESM7_ESM.zip › Figure 5/5e/HCT116 0.5 1/116_0.51_RGB.tif]

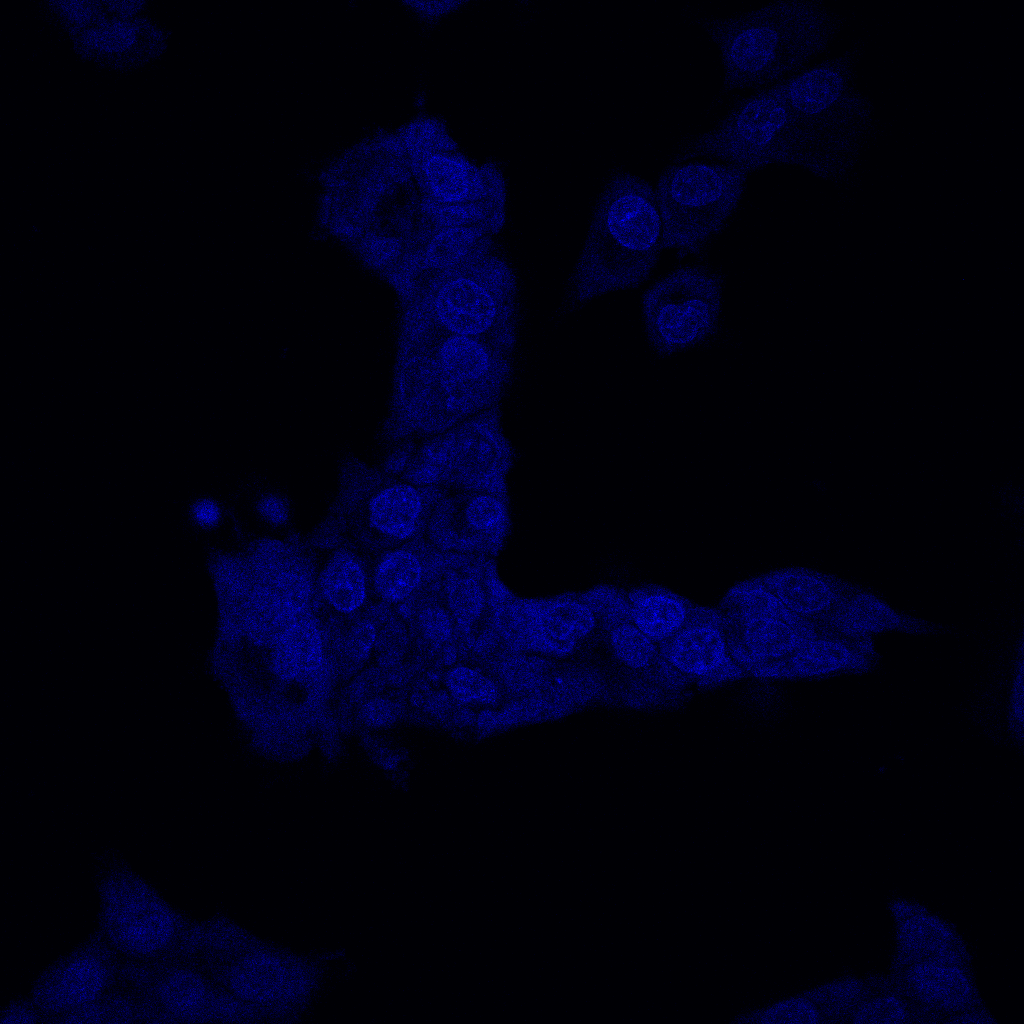

Supplement: Supplementary file 7 — Source data Fig. 5 [file 44321_2025_308_MOESM7_ESM.zip › Figure 5/5e/HCT116 0.5 1/116_0.51_RGB_DAPI.tif]

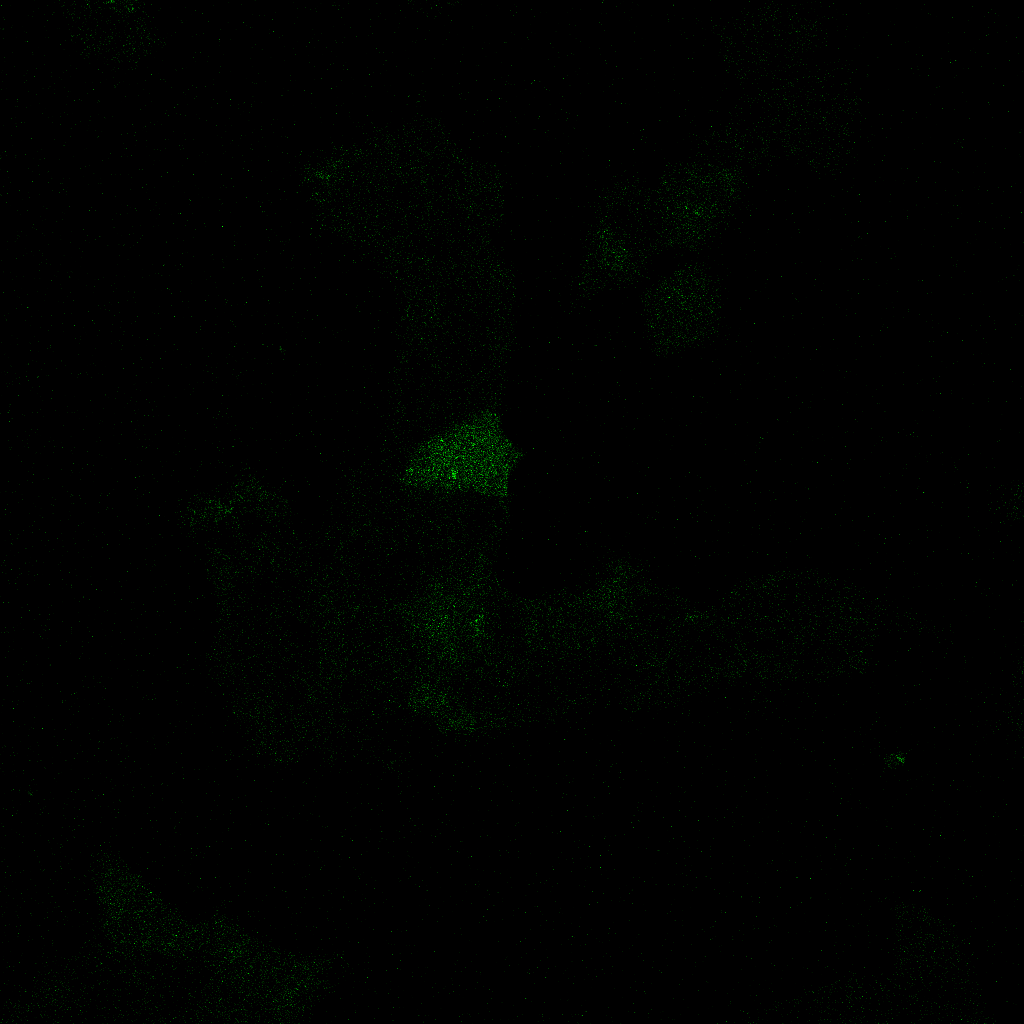

Supplement: Supplementary file 7 — Source data Fig. 5 [file 44321_2025_308_MOESM7_ESM.zip › Figure 5/5e/HCT116 0.5 1/116_0.51_RGB_FITC.tif]

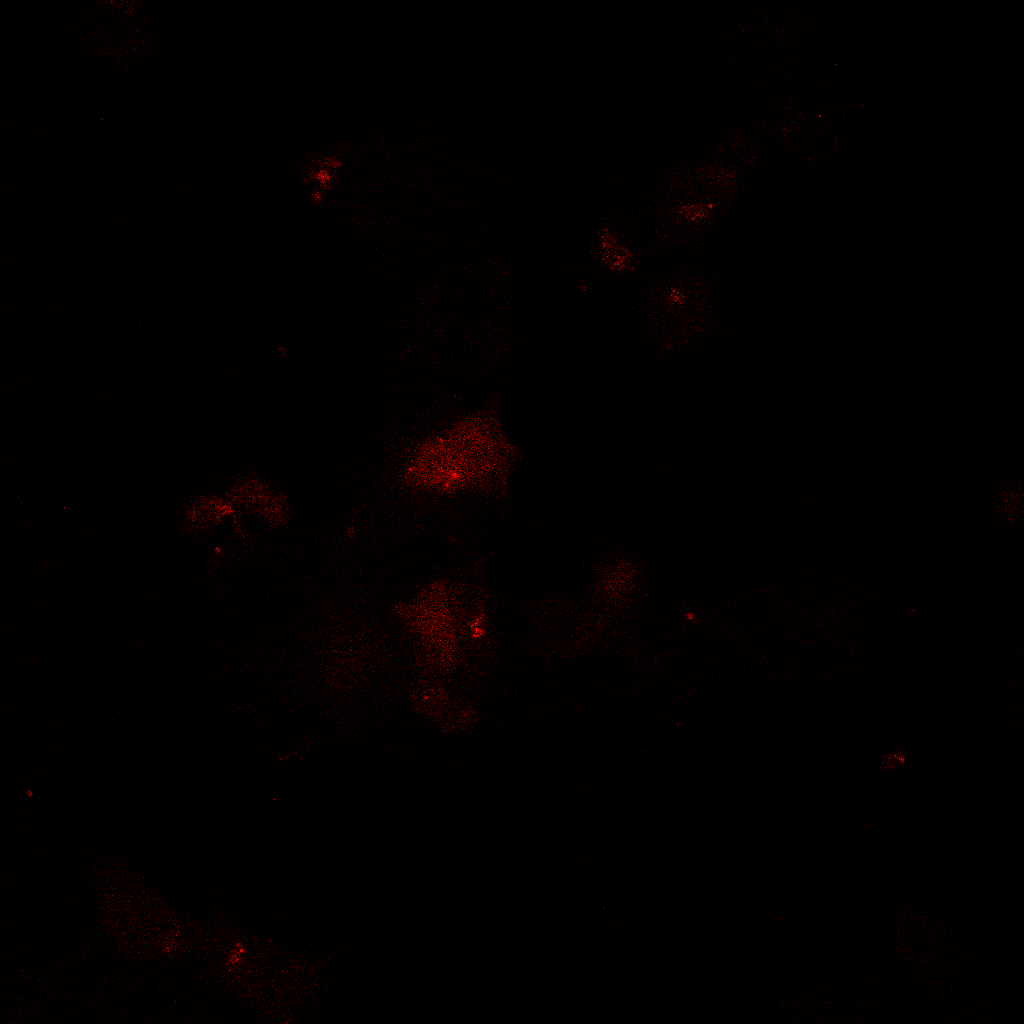

Supplement: Supplementary file 7 — Source data Fig. 5 [file 44321_2025_308_MOESM7_ESM.zip › Figure 5/5e/HCT116 0.5 1/116_0.51_RGB_TRITC.tif]

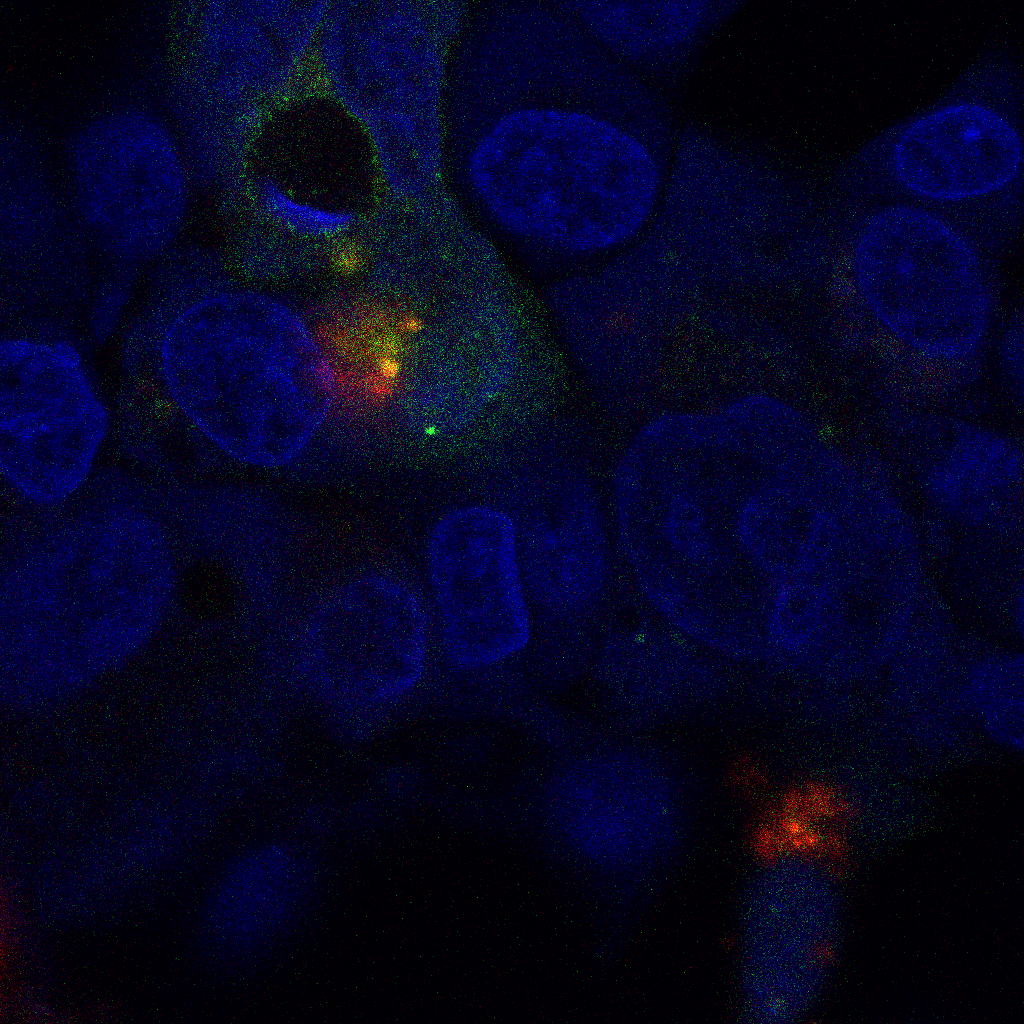

Supplement: Supplementary file 7 — Source data Fig. 5 [file 44321_2025_308_MOESM7_ESM.zip › Figure 5/5e/HCT116 0.5 2/116_0.52_RGB.tif]

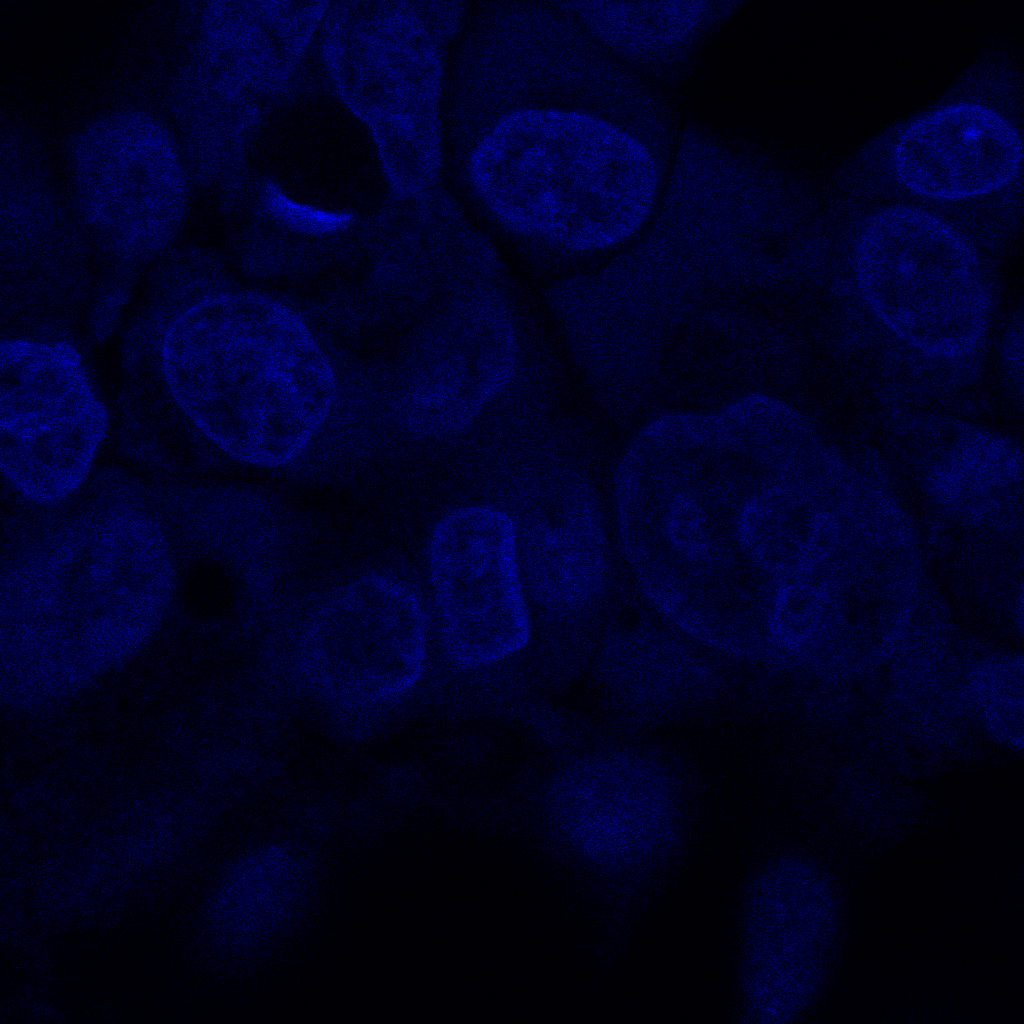

Supplement: Supplementary file 7 — Source data Fig. 5 [file 44321_2025_308_MOESM7_ESM.zip › Figure 5/5e/HCT116 0.5 2/116_0.52_RGB_DAPI.tif]

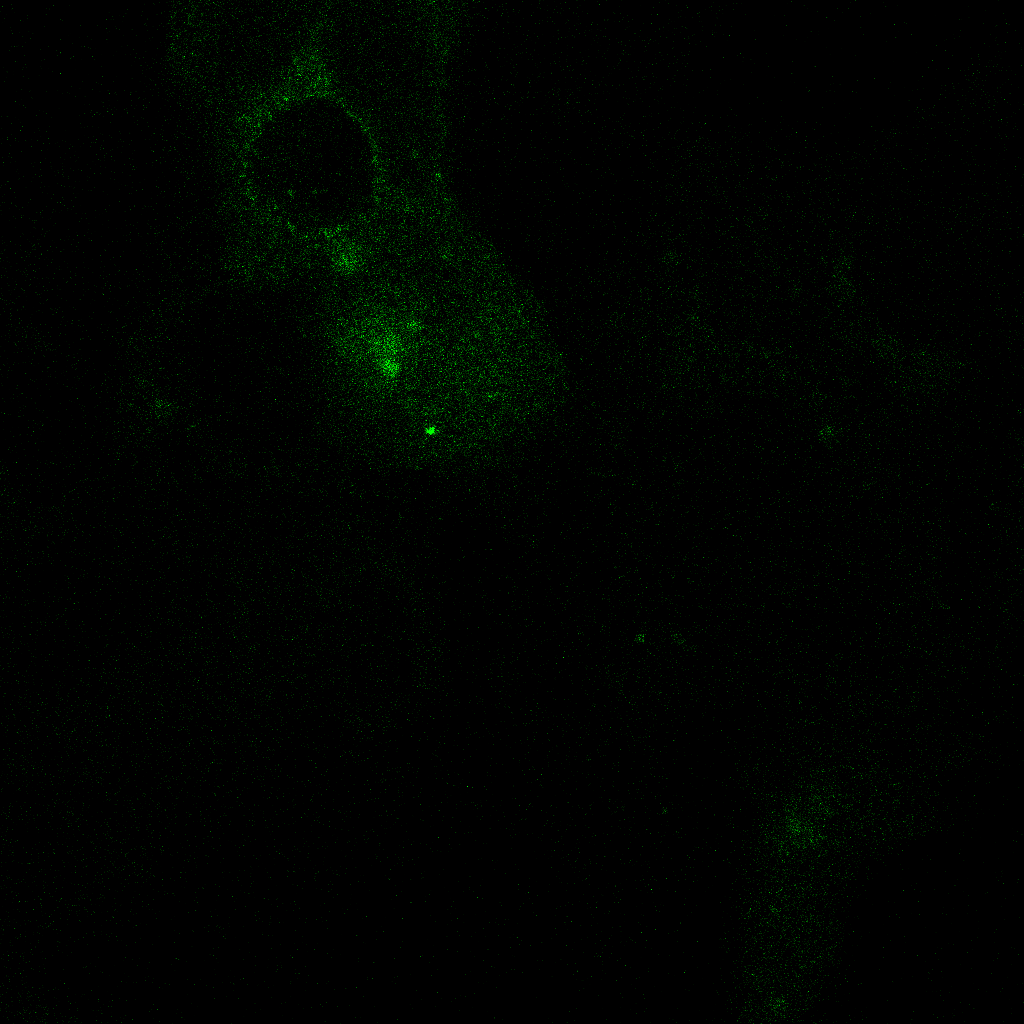

Supplement: Supplementary file 7 — Source data Fig. 5 [file 44321_2025_308_MOESM7_ESM.zip › Figure 5/5e/HCT116 0.5 2/116_0.52_RGB_FITC.tif]

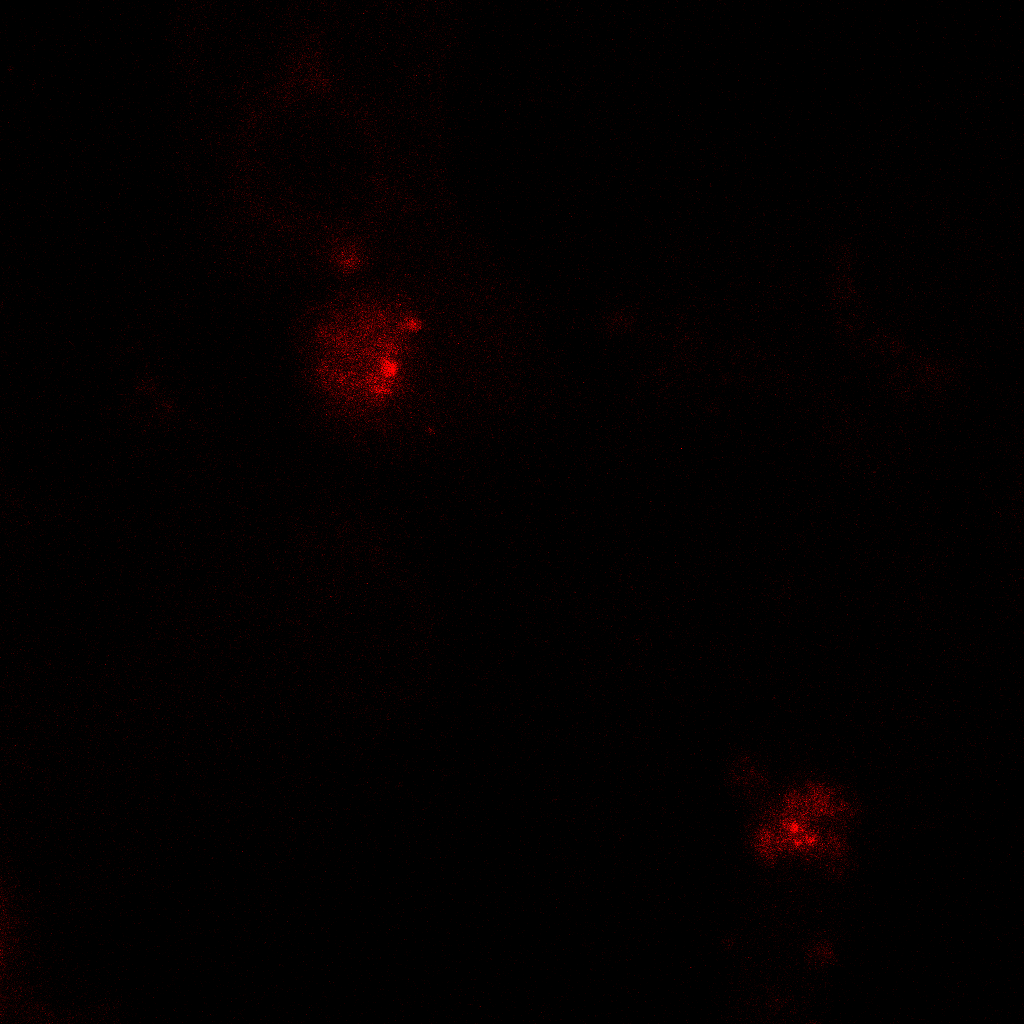

Supplement: Supplementary file 7 — Source data Fig. 5 [file 44321_2025_308_MOESM7_ESM.zip › Figure 5/5e/HCT116 0.5 2/116_0.52_RGB_TRITC.tif]

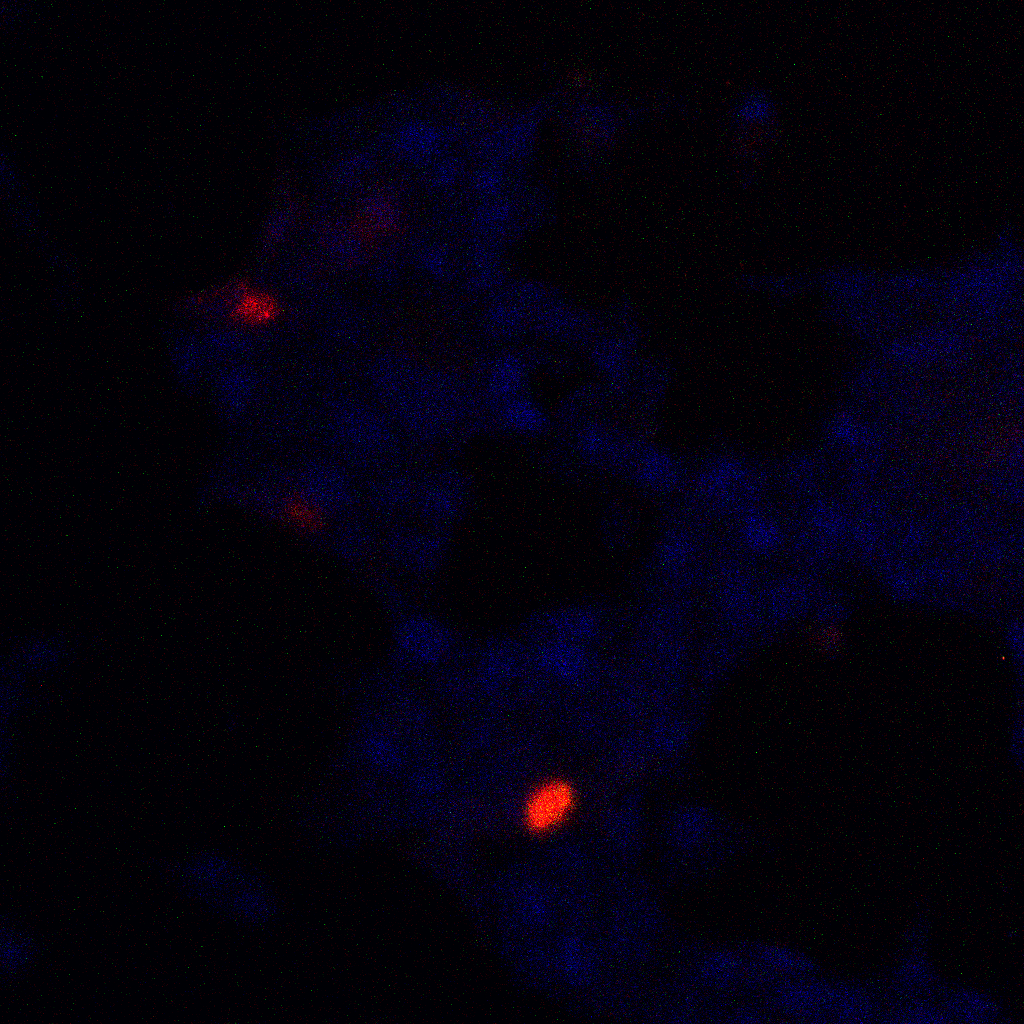

Supplement: Supplementary file 7 — Source data Fig. 5 [file 44321_2025_308_MOESM7_ESM.zip › Figure 5/5e/HCT116 0.5 3/116_0.53_RGB.tif]

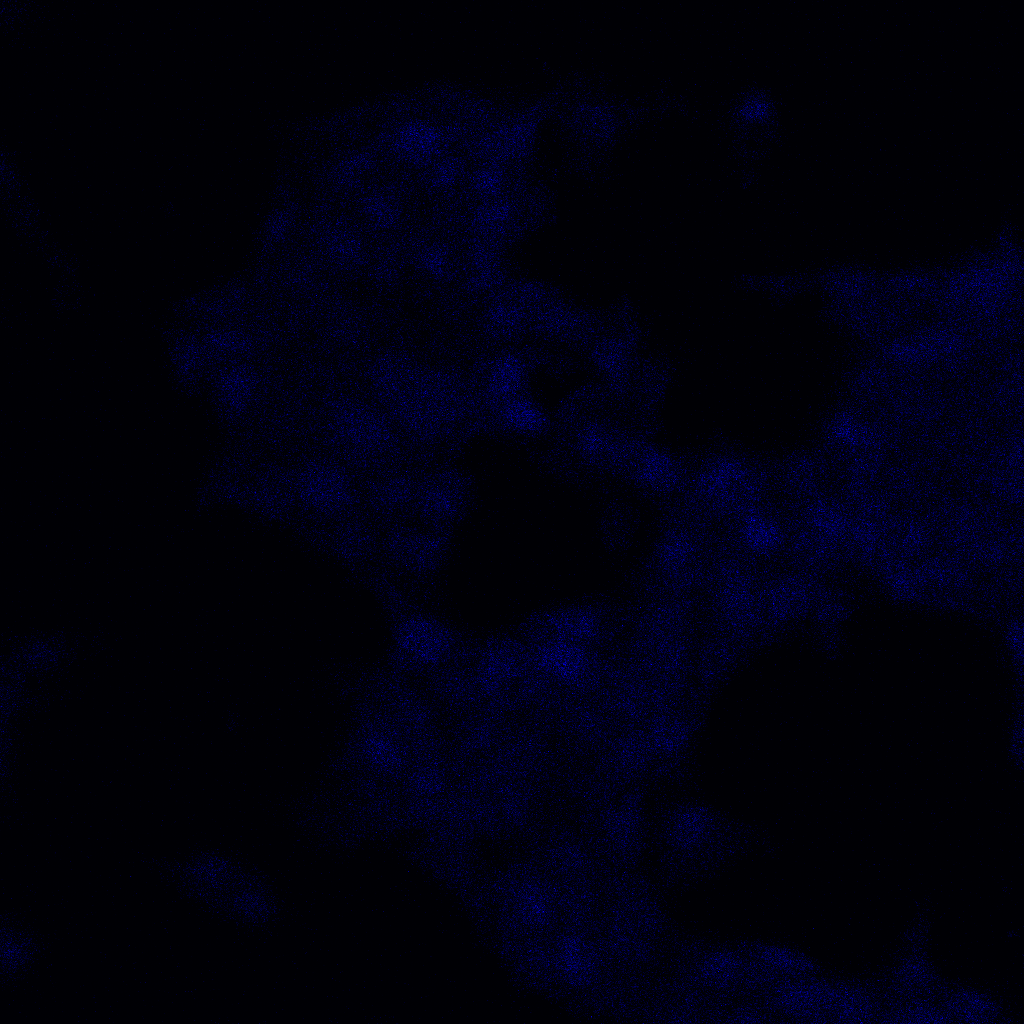

Supplement: Supplementary file 7 — Source data Fig. 5 [file 44321_2025_308_MOESM7_ESM.zip › Figure 5/5e/HCT116 0.5 3/116_0.53_RGB_DAPI.tif]

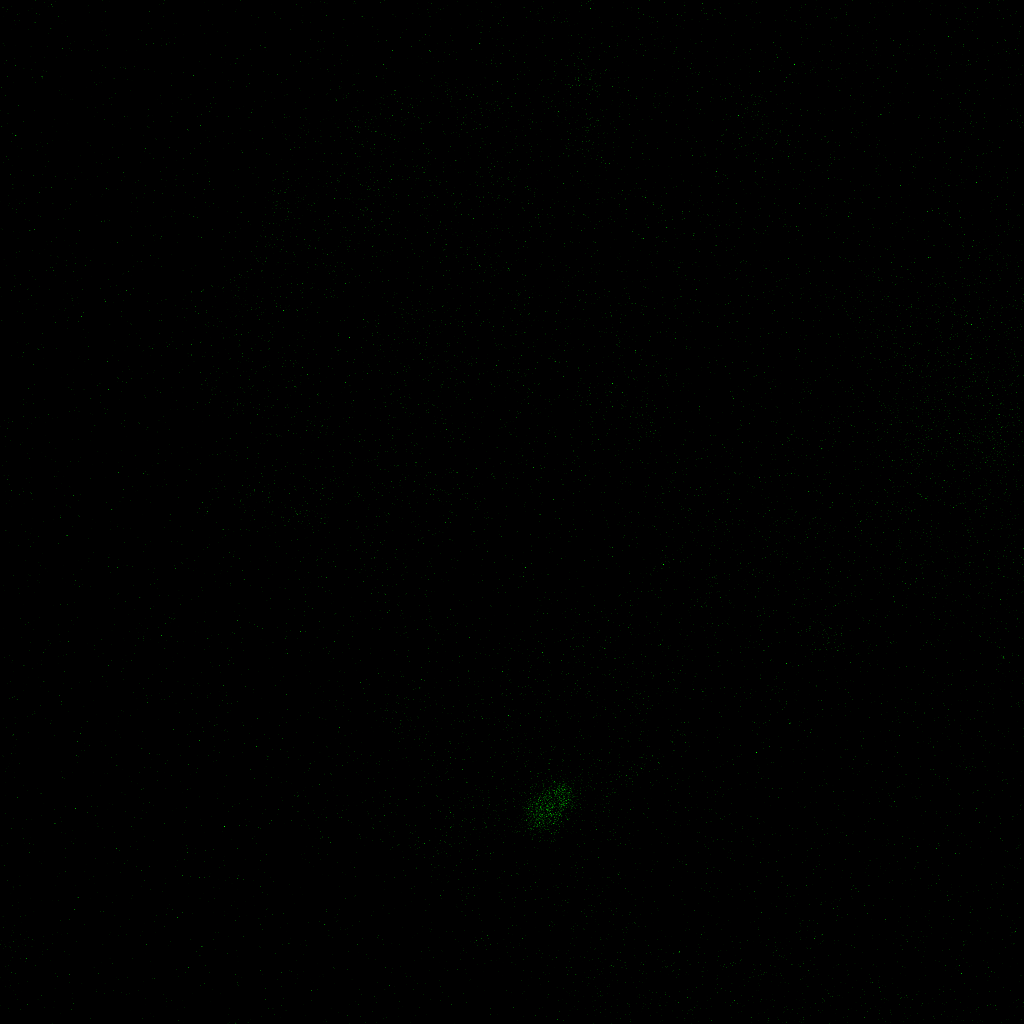

Supplement: Supplementary file 7 — Source data Fig. 5 [file 44321_2025_308_MOESM7_ESM.zip › Figure 5/5e/HCT116 0.5 3/116_0.53_RGB_FITC.tif]

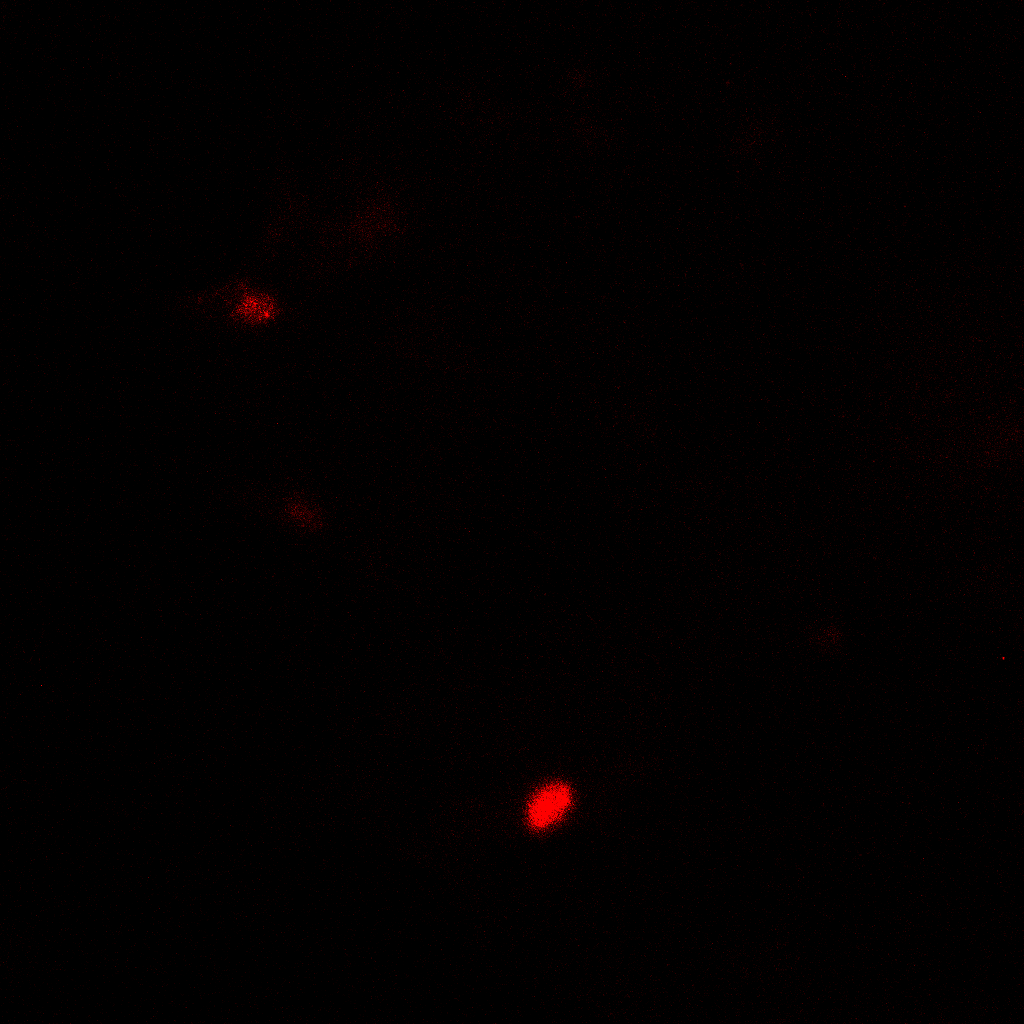

Supplement: Supplementary file 7 — Source data Fig. 5 [file 44321_2025_308_MOESM7_ESM.zip › Figure 5/5e/HCT116 0.5 3/116_0.53_RGB_TRITC.tif]

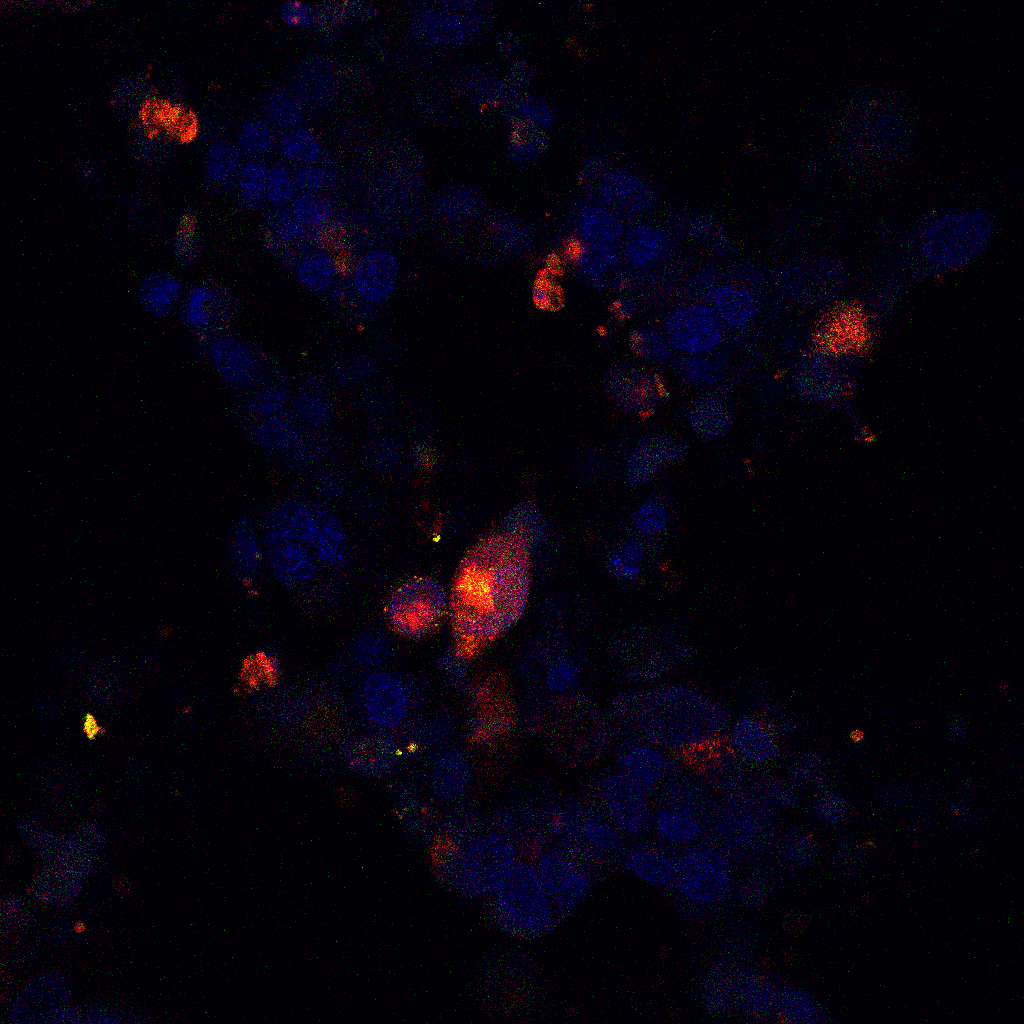

Supplement: Supplementary file 7 — Source data Fig. 5 [file 44321_2025_308_MOESM7_ESM.zip › Figure 5/5e/HCT116 1 1/116_11_RGB.tif]

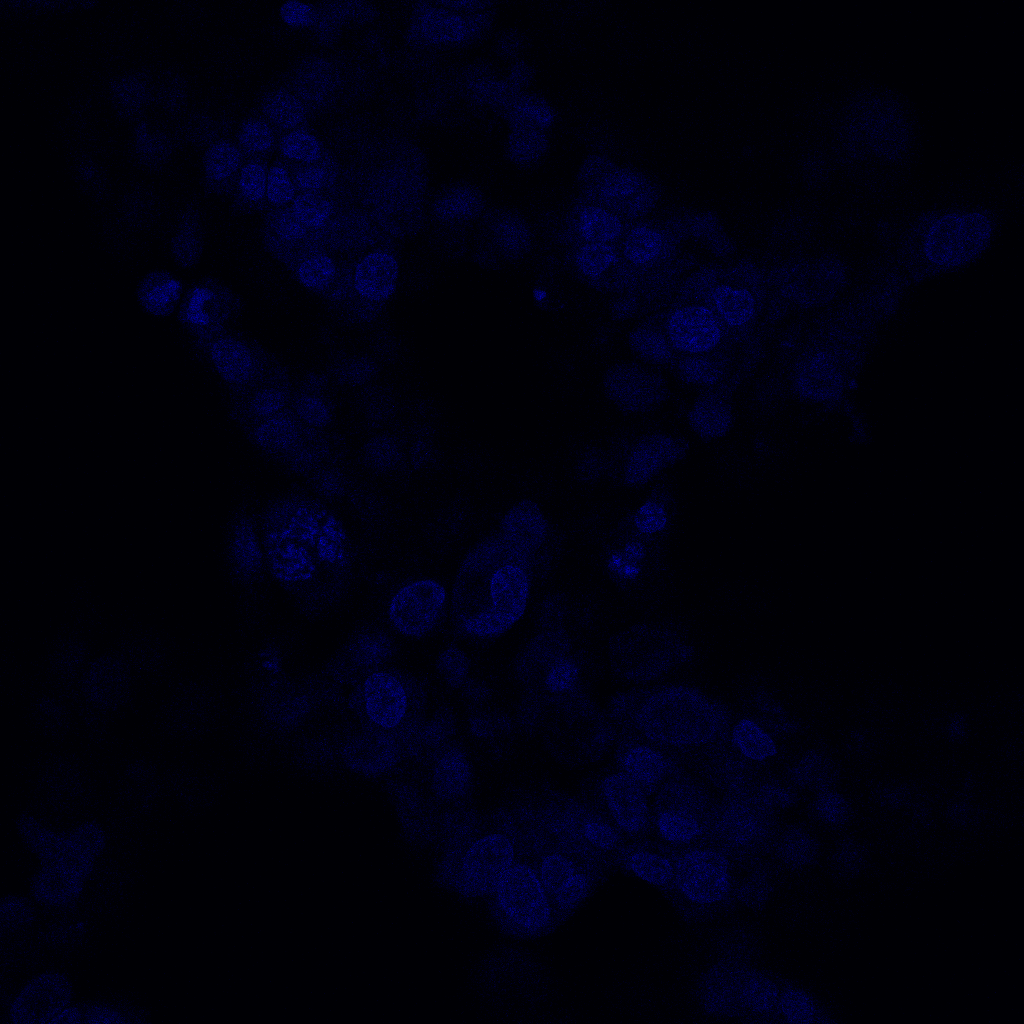

Supplement: Supplementary file 7 — Source data Fig. 5 [file 44321_2025_308_MOESM7_ESM.zip › Figure 5/5e/HCT116 1 1/116_11_RGB_DAPI.tif]

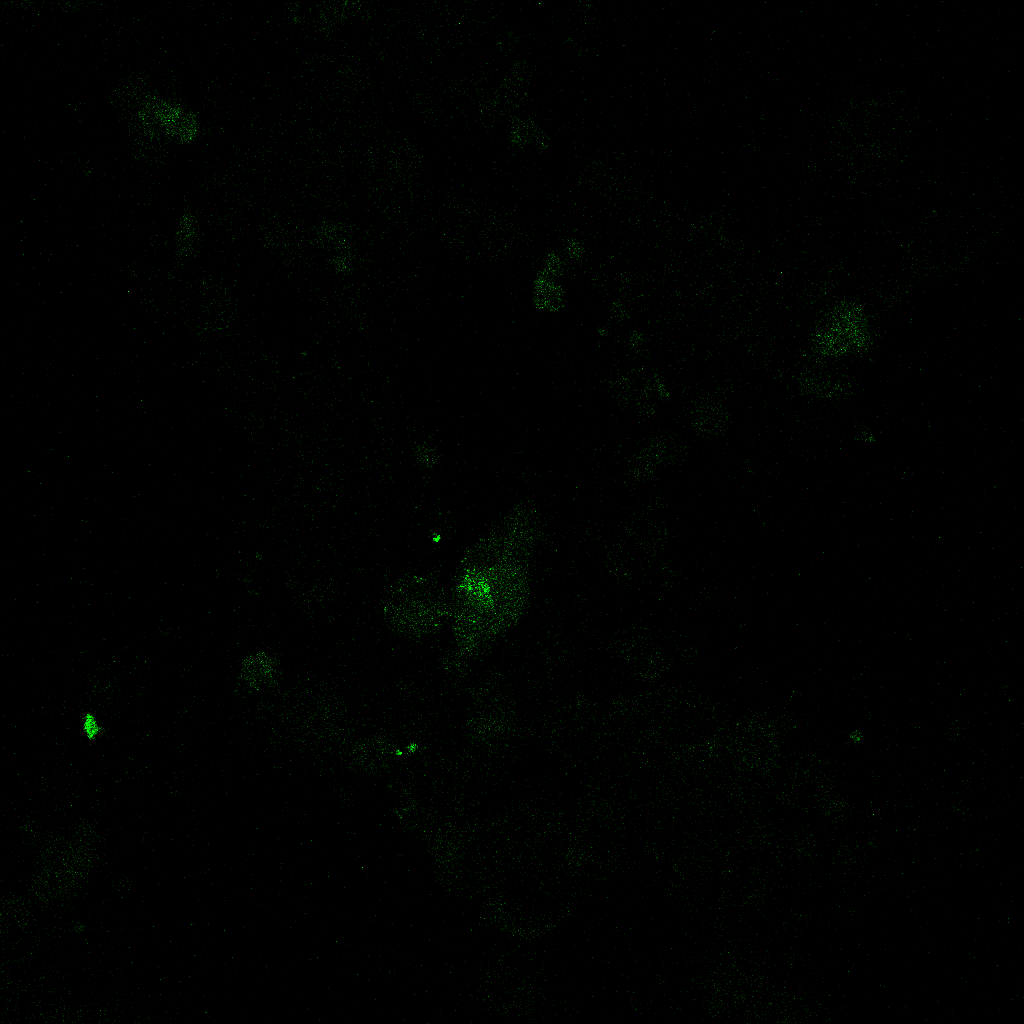

Supplement: Supplementary file 7 — Source data Fig. 5 [file 44321_2025_308_MOESM7_ESM.zip › Figure 5/5e/HCT116 1 1/116_11_RGB_FITC.tif]

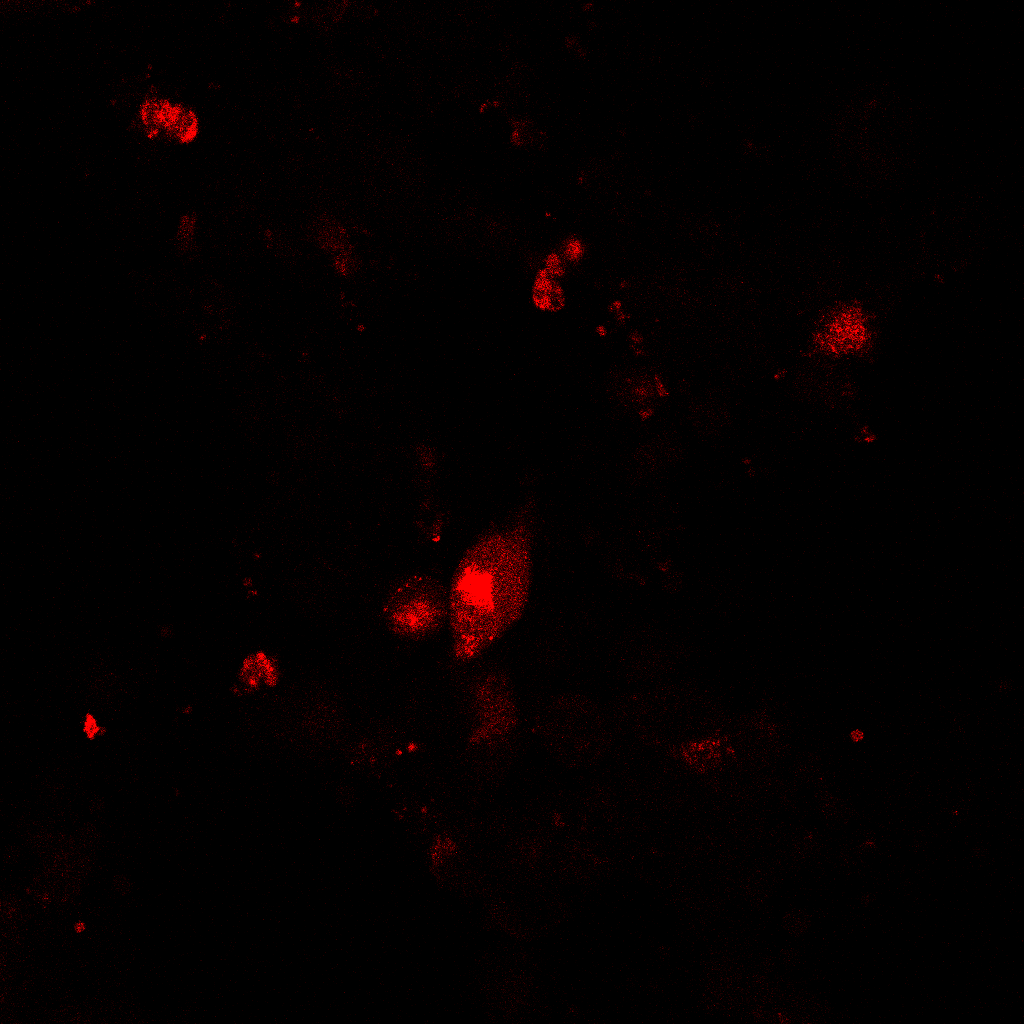

Supplement: Supplementary file 7 — Source data Fig. 5 [file 44321_2025_308_MOESM7_ESM.zip › Figure 5/5e/HCT116 1 1/116_11_RGB_TRITC.tif]

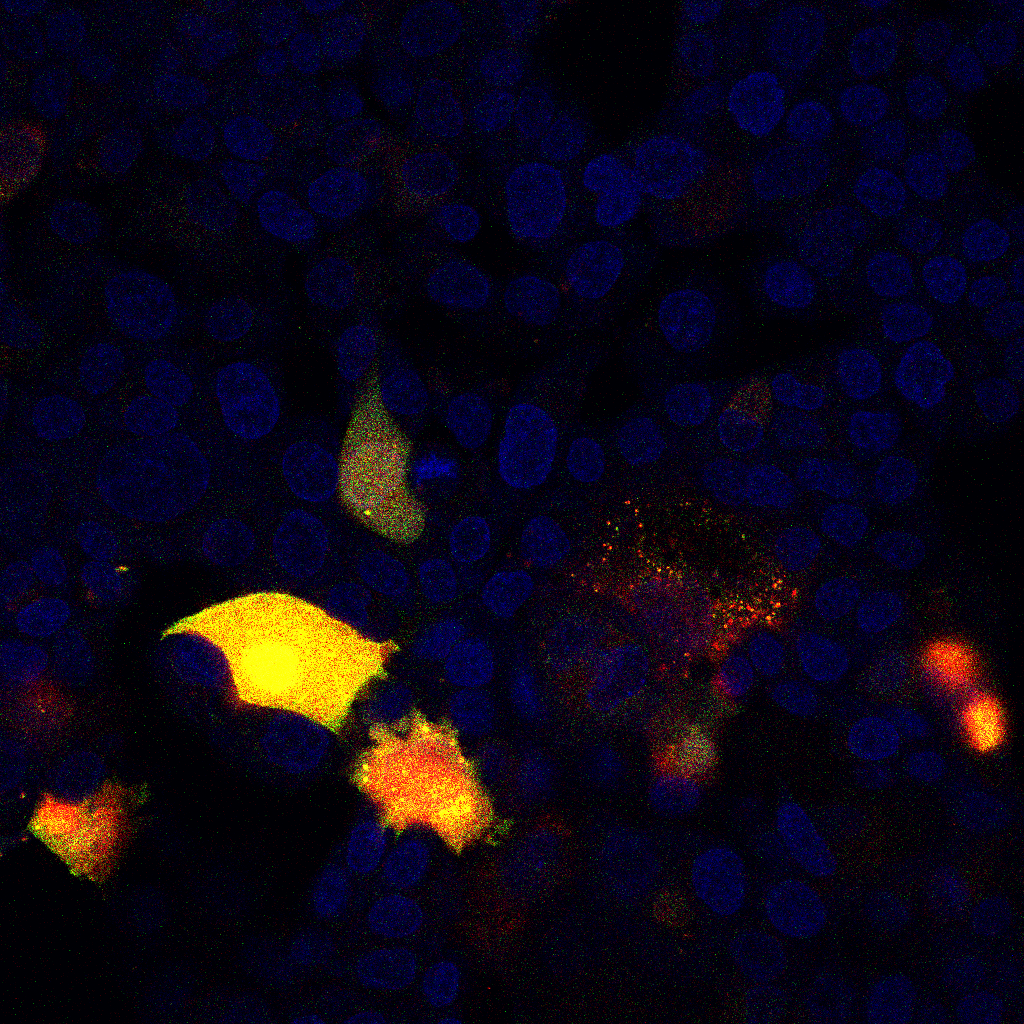

Supplement: Supplementary file 7 — Source data Fig. 5 [file 44321_2025_308_MOESM7_ESM.zip › Figure 5/5e/HCT116 1 2/116_12_RGB.tif]

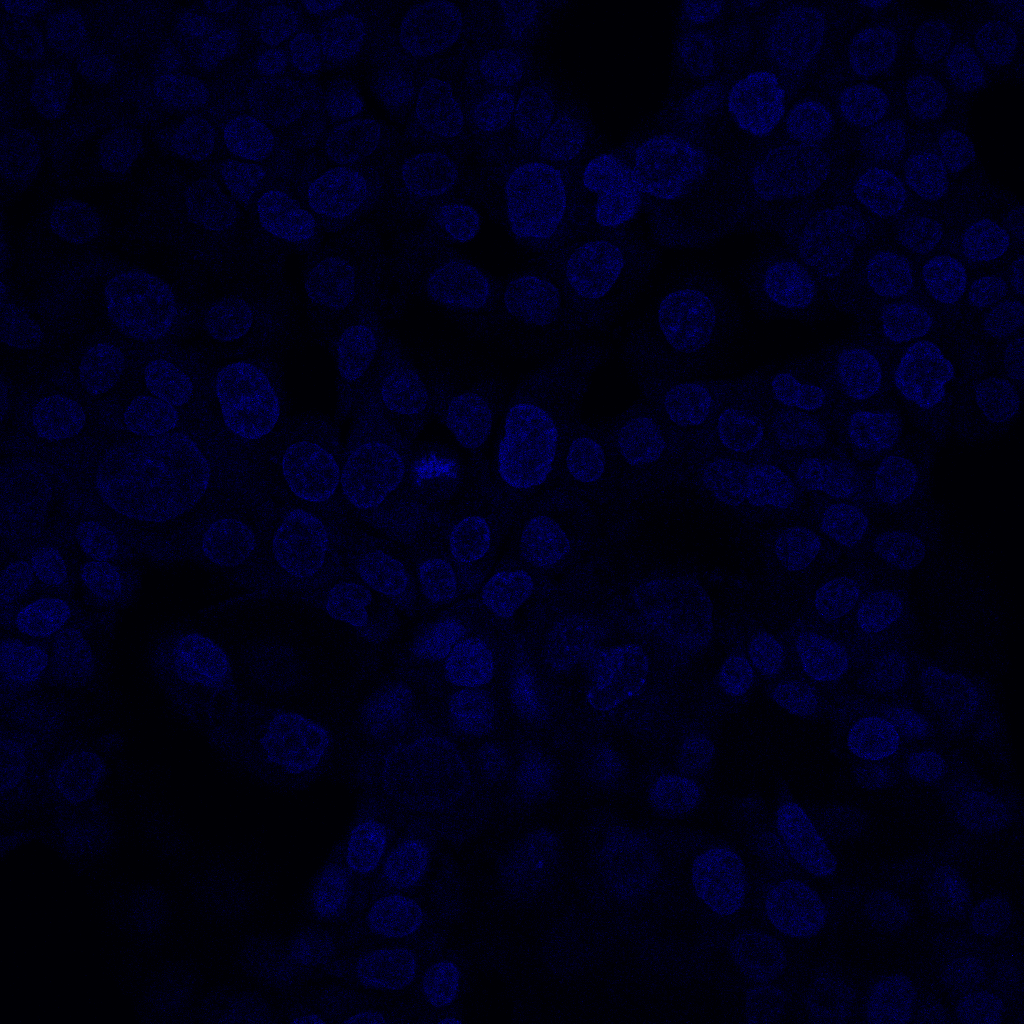

Supplement: Supplementary file 7 — Source data Fig. 5 [file 44321_2025_308_MOESM7_ESM.zip › Figure 5/5e/HCT116 1 2/116_12_RGB_DAPI.tif]
