# Supplementary figures and images for: AI-identified CD133-targeting natural compounds demonstrate differential anti-tumor effects and mechanisms in pan-cancer models (part 3 of 4)
Source: EMBO Mol Med. 2025 Oct 2;17(11):2932–65. doi: 10.1038/s44321-025-00308-1 (PMC12603267; doi:10.1038/s44321-025-00308-1)

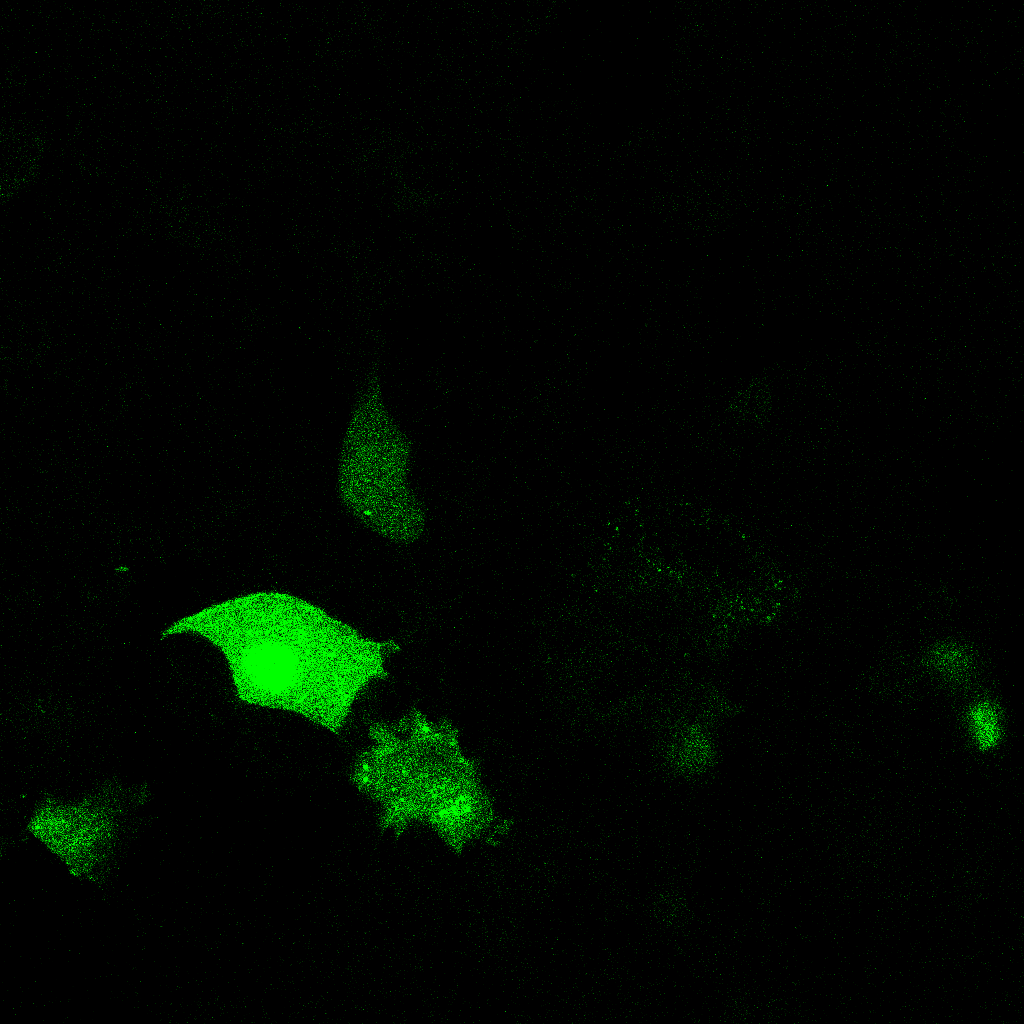

Supplement: Supplementary file 7 — Source data Fig. 5 [file 44321_2025_308_MOESM7_ESM.zip › Figure 5/5e/HCT116 1 2/116_12_RGB_FITC.tif]

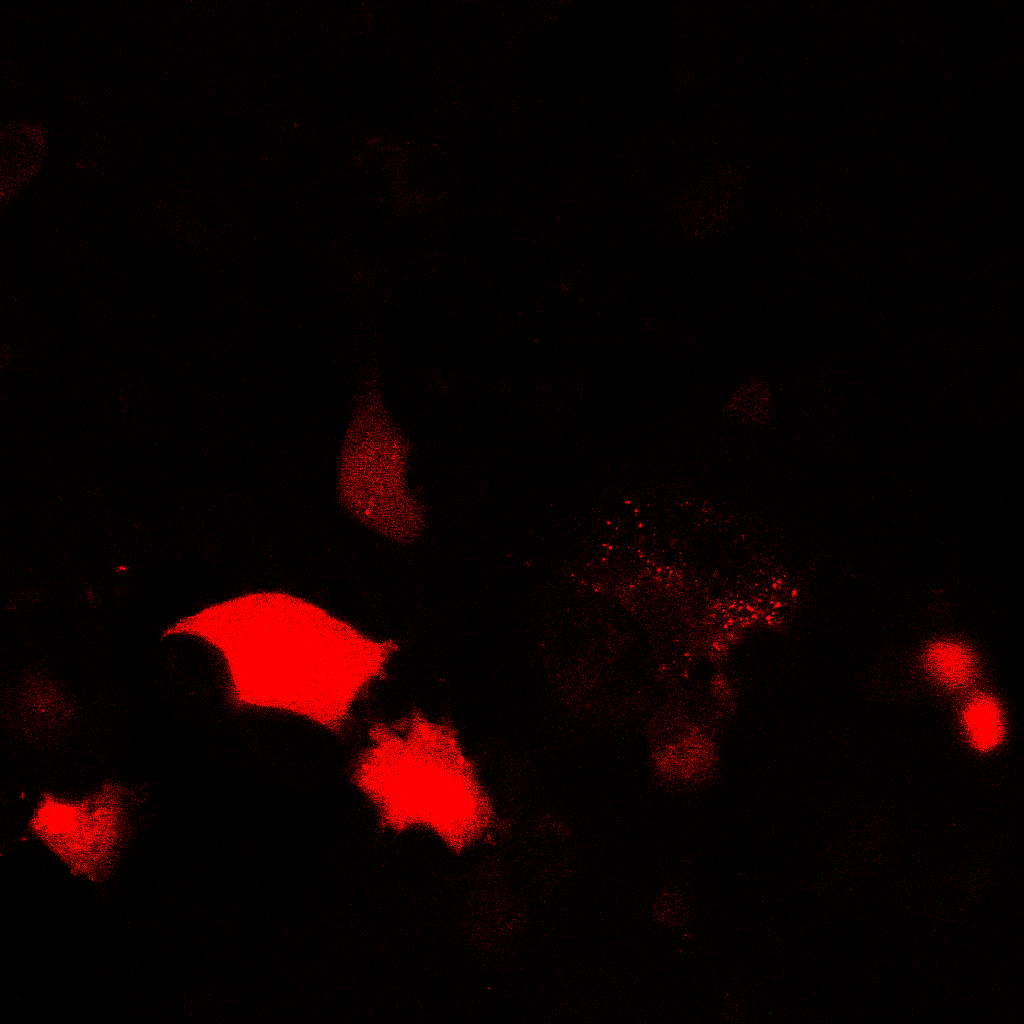

Supplement: Supplementary file 7 — Source data Fig. 5 [file 44321_2025_308_MOESM7_ESM.zip › Figure 5/5e/HCT116 1 2/116_12_RGB_TRITC.tif]

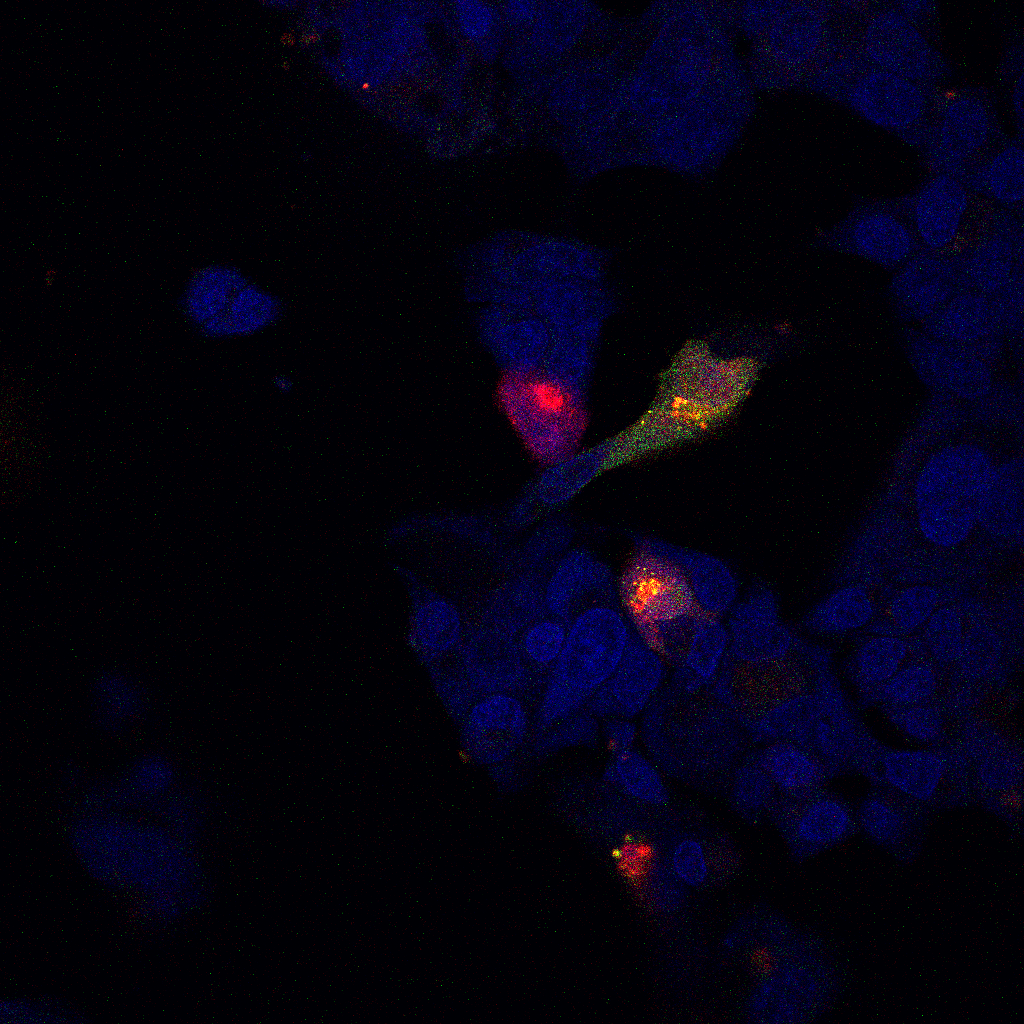

Supplement: Supplementary file 7 — Source data Fig. 5 [file 44321_2025_308_MOESM7_ESM.zip › Figure 5/5e/HCT116 1 3/116_13_RGB.tif]

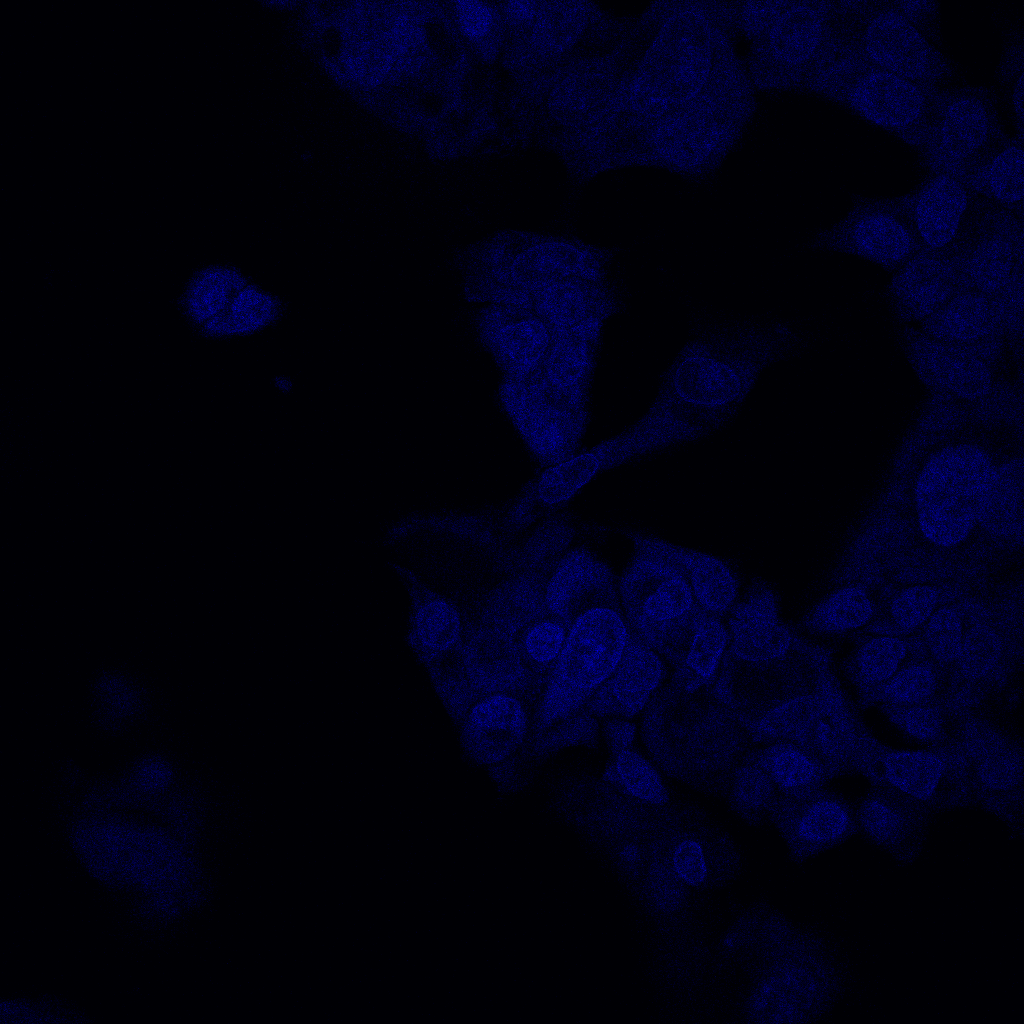

Supplement: Supplementary file 7 — Source data Fig. 5 [file 44321_2025_308_MOESM7_ESM.zip › Figure 5/5e/HCT116 1 3/116_13_RGB_DAPI.tif]

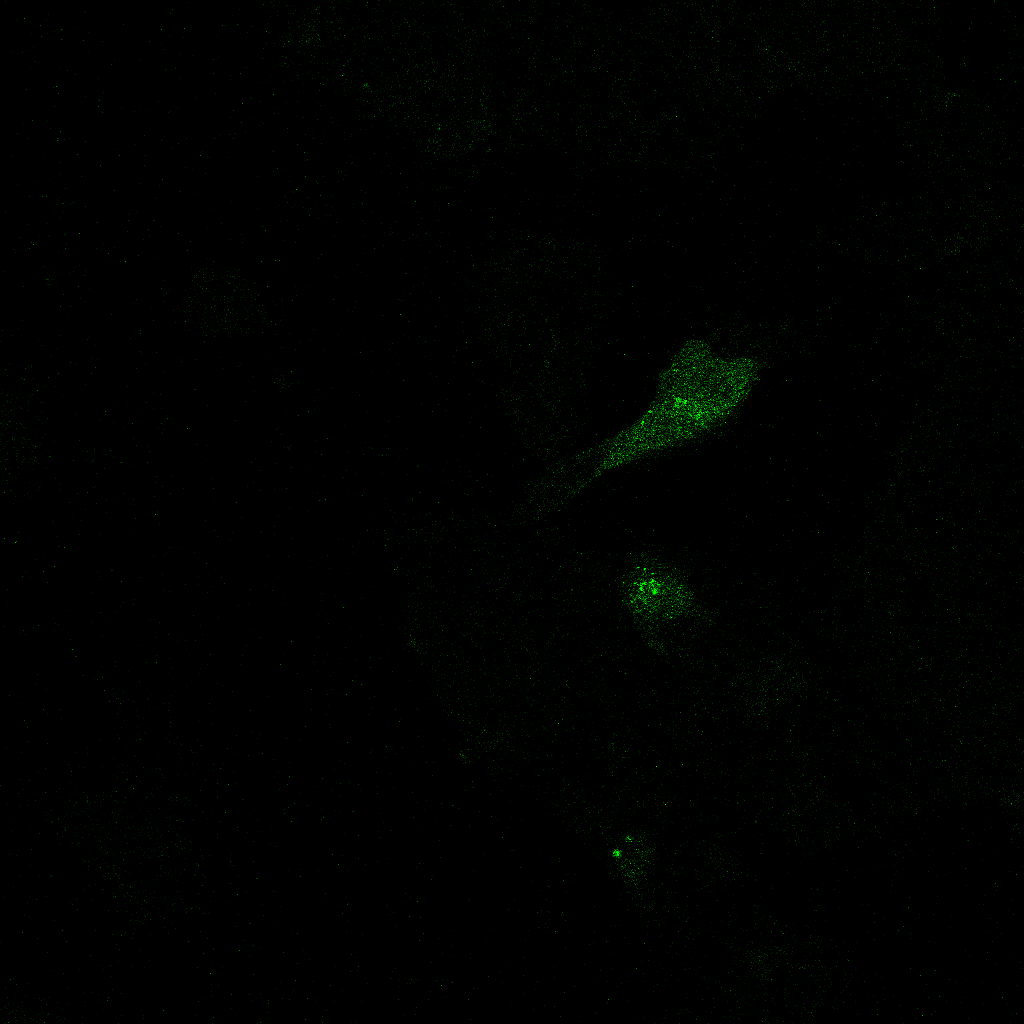

Supplement: Supplementary file 7 — Source data Fig. 5 [file 44321_2025_308_MOESM7_ESM.zip › Figure 5/5e/HCT116 1 3/116_13_RGB_FITC.tif]

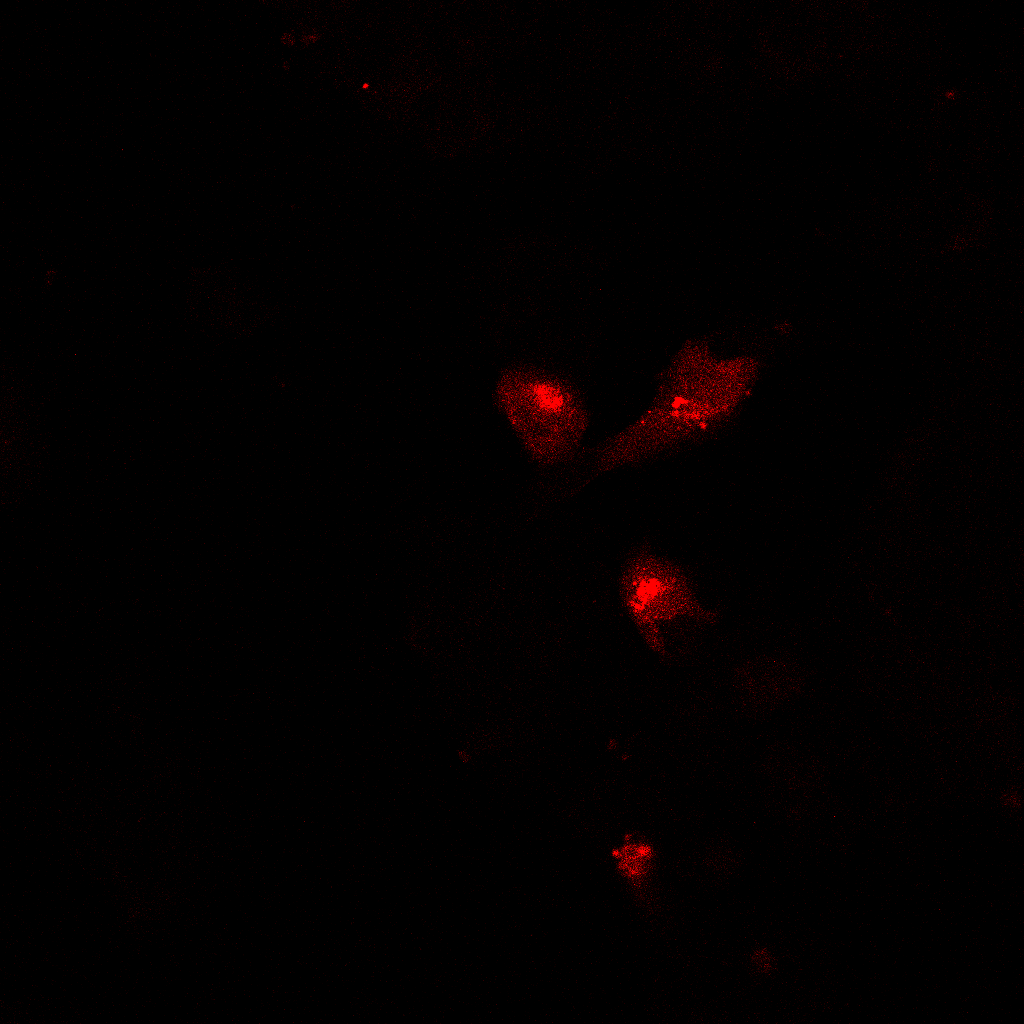

Supplement: Supplementary file 7 — Source data Fig. 5 [file 44321_2025_308_MOESM7_ESM.zip › Figure 5/5e/HCT116 1 3/116_13_RGB_TRITC.tif]

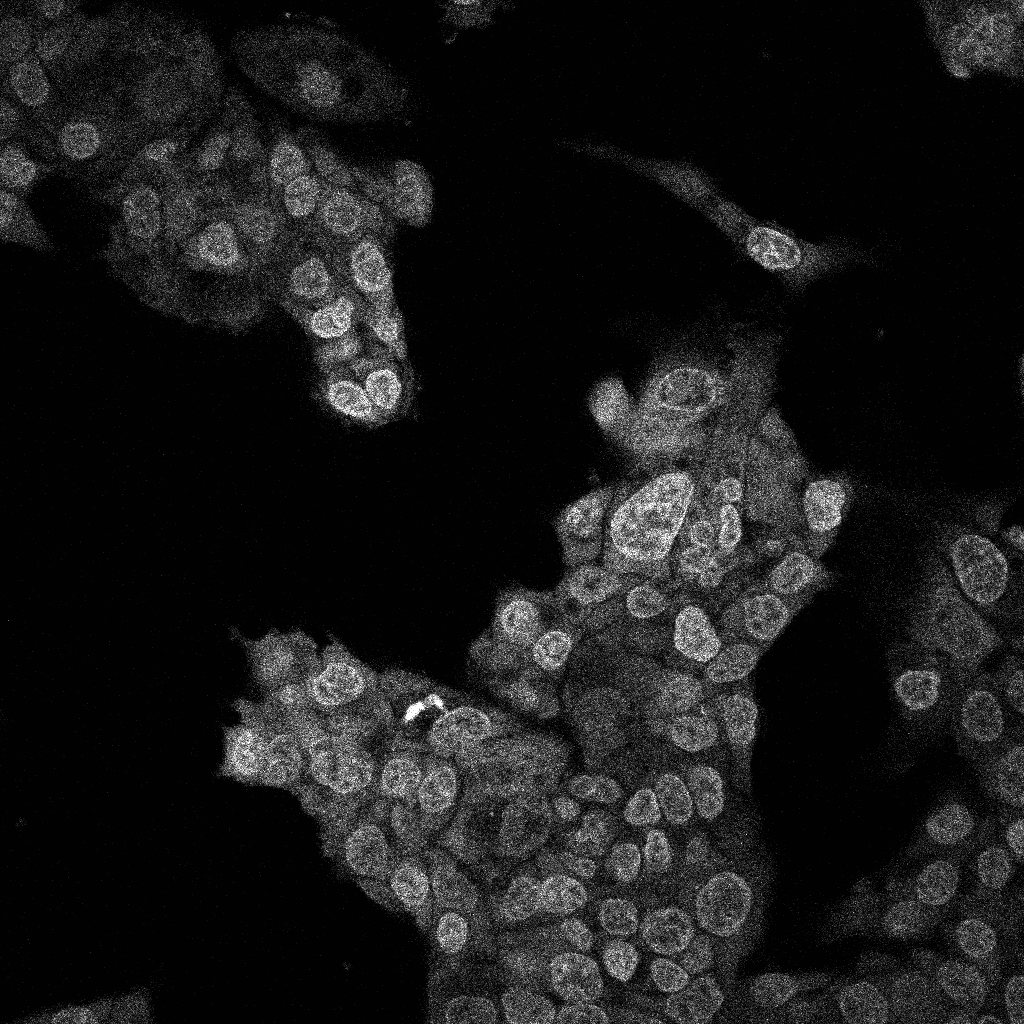

Supplement: Supplementary file 7 — Source data Fig. 5 [file 44321_2025_308_MOESM7_ESM.zip › Figure 5/5e/HCT116 Con 1/116 C1_116 C1.tif]

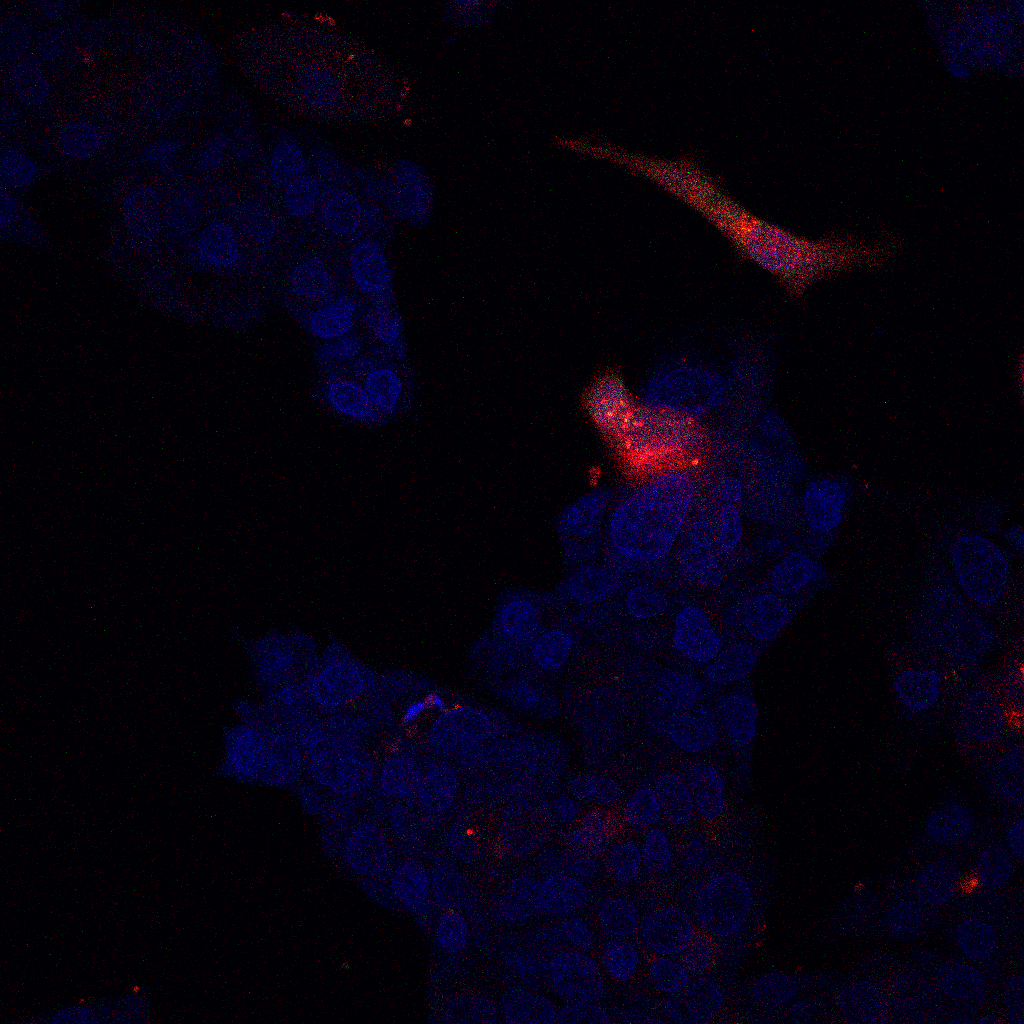

Supplement: Supplementary file 7 — Source data Fig. 5 [file 44321_2025_308_MOESM7_ESM.zip › Figure 5/5e/HCT116 Con 1/116 C1_116 C1_RGB.tif]

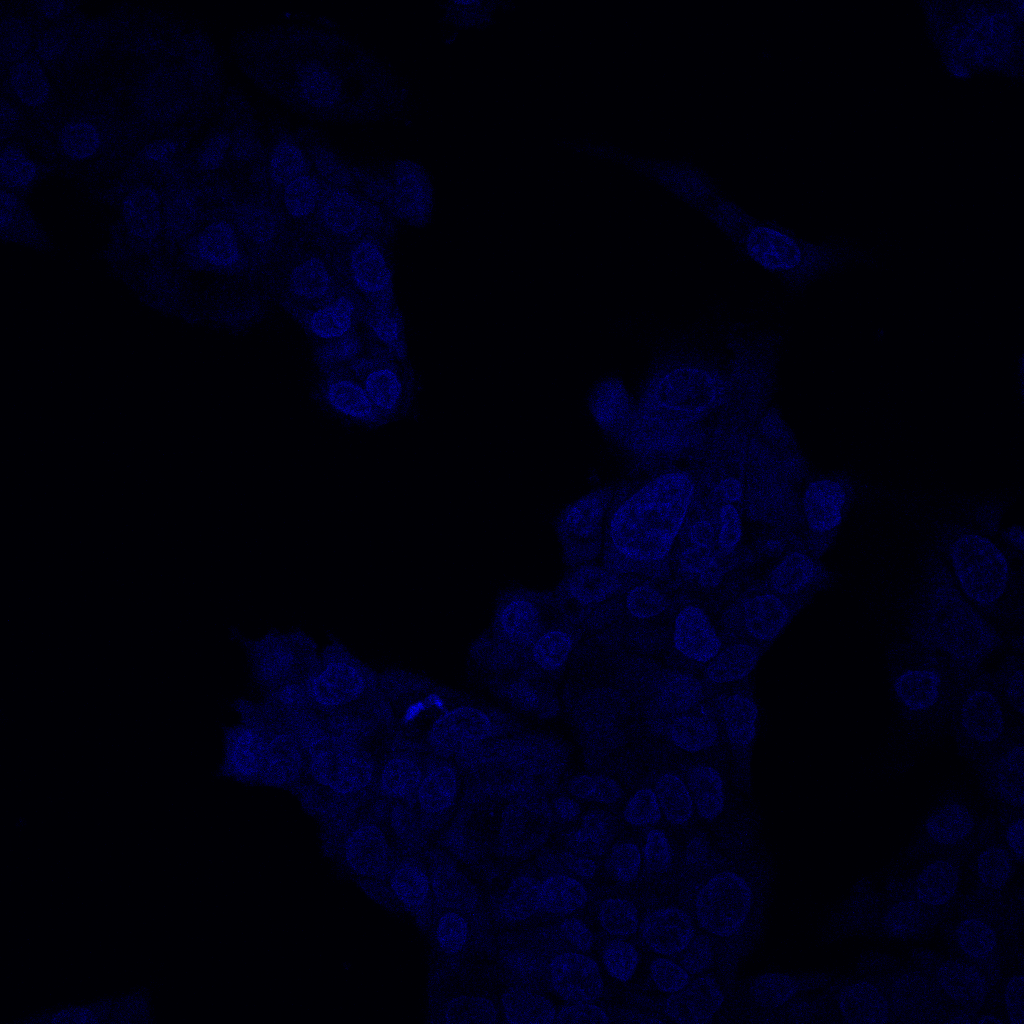

Supplement: Supplementary file 7 — Source data Fig. 5 [file 44321_2025_308_MOESM7_ESM.zip › Figure 5/5e/HCT116 Con 1/116 C1_116 C1_RGB_DAPI.tif]

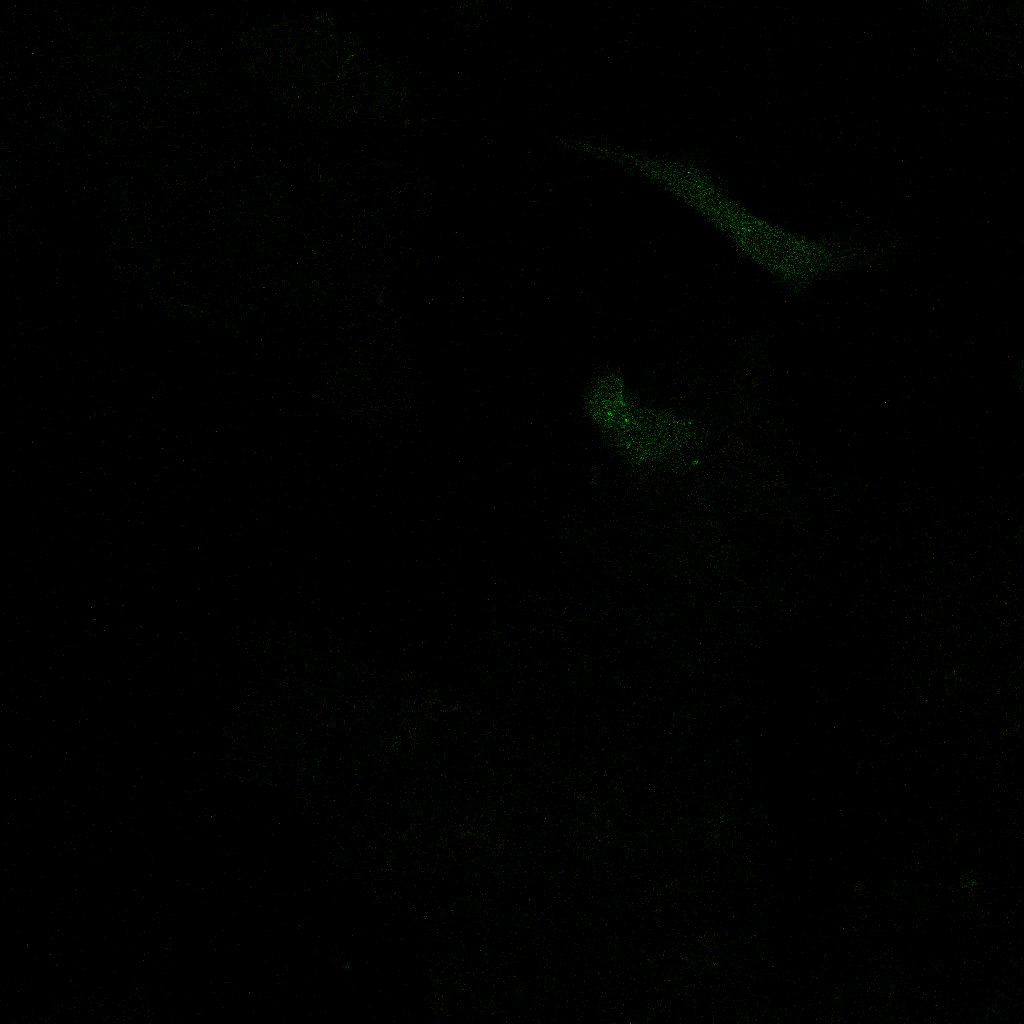

Supplement: Supplementary file 7 — Source data Fig. 5 [file 44321_2025_308_MOESM7_ESM.zip › Figure 5/5e/HCT116 Con 1/116 C1_116 C1_RGB_FITC.tif]

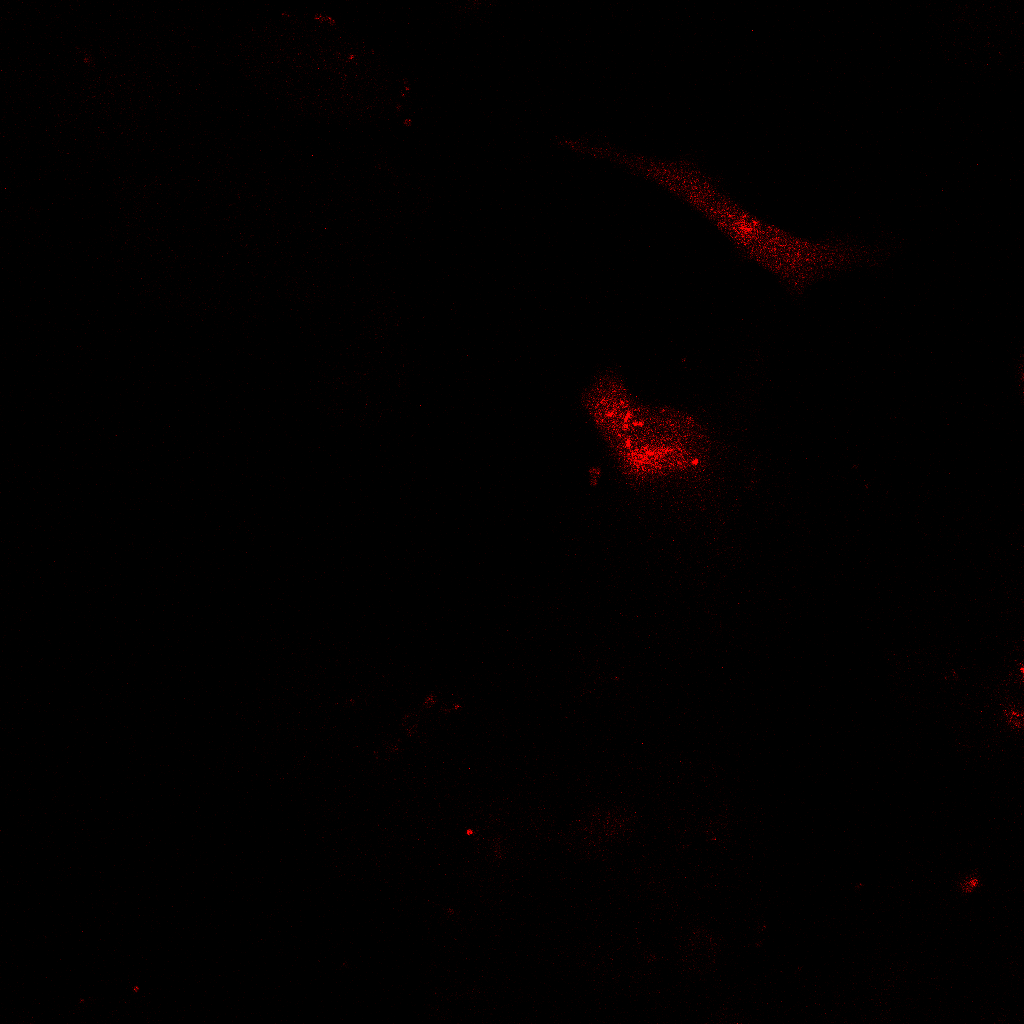

Supplement: Supplementary file 7 — Source data Fig. 5 [file 44321_2025_308_MOESM7_ESM.zip › Figure 5/5e/HCT116 Con 1/116 C1_116 C1_RGB_TRITC.tif]

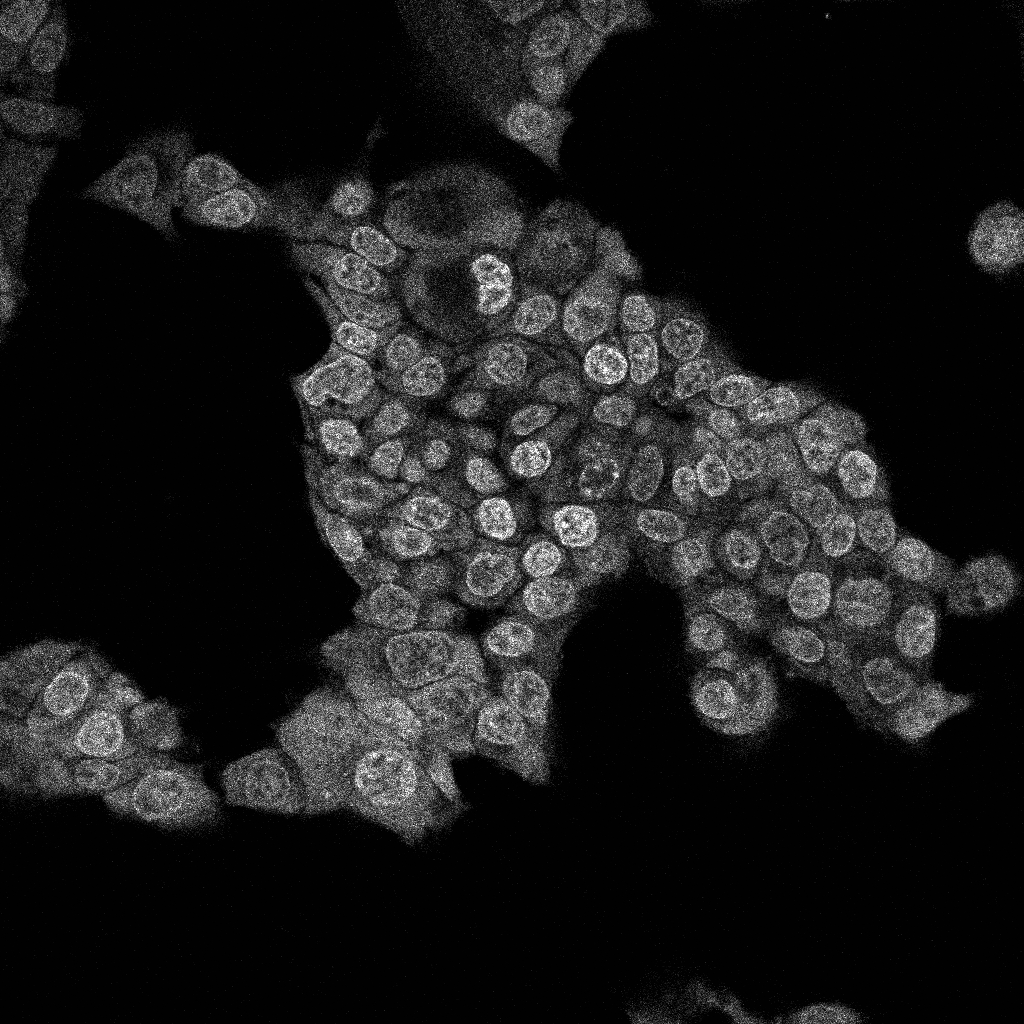

Supplement: Supplementary file 7 — Source data Fig. 5 [file 44321_2025_308_MOESM7_ESM.zip › Figure 5/5e/HCT116 Con 2/116 C2_116 C2.tif]

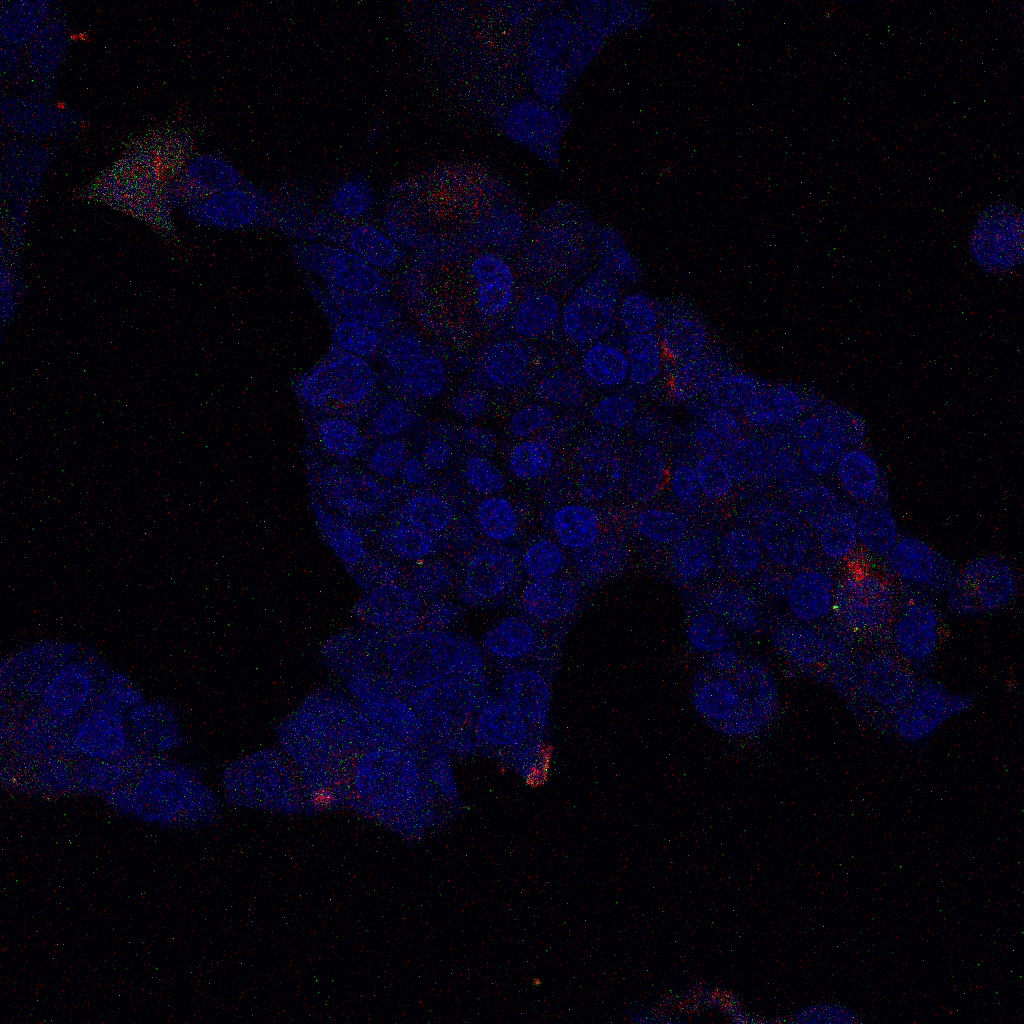

Supplement: Supplementary file 7 — Source data Fig. 5 [file 44321_2025_308_MOESM7_ESM.zip › Figure 5/5e/HCT116 Con 2/116 C2_116 C2_RGB.tif]

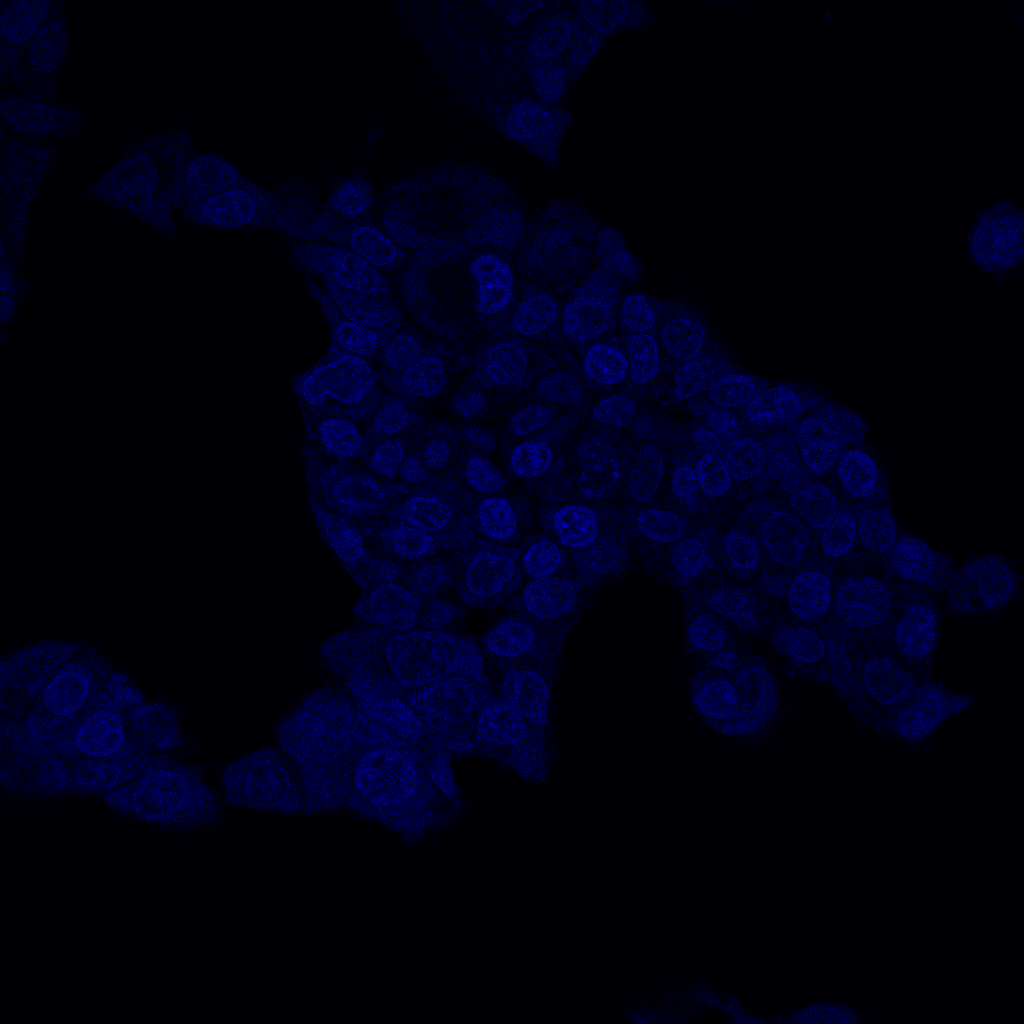

Supplement: Supplementary file 7 — Source data Fig. 5 [file 44321_2025_308_MOESM7_ESM.zip › Figure 5/5e/HCT116 Con 2/116 C2_116 C2_RGB_DAPI.tif]

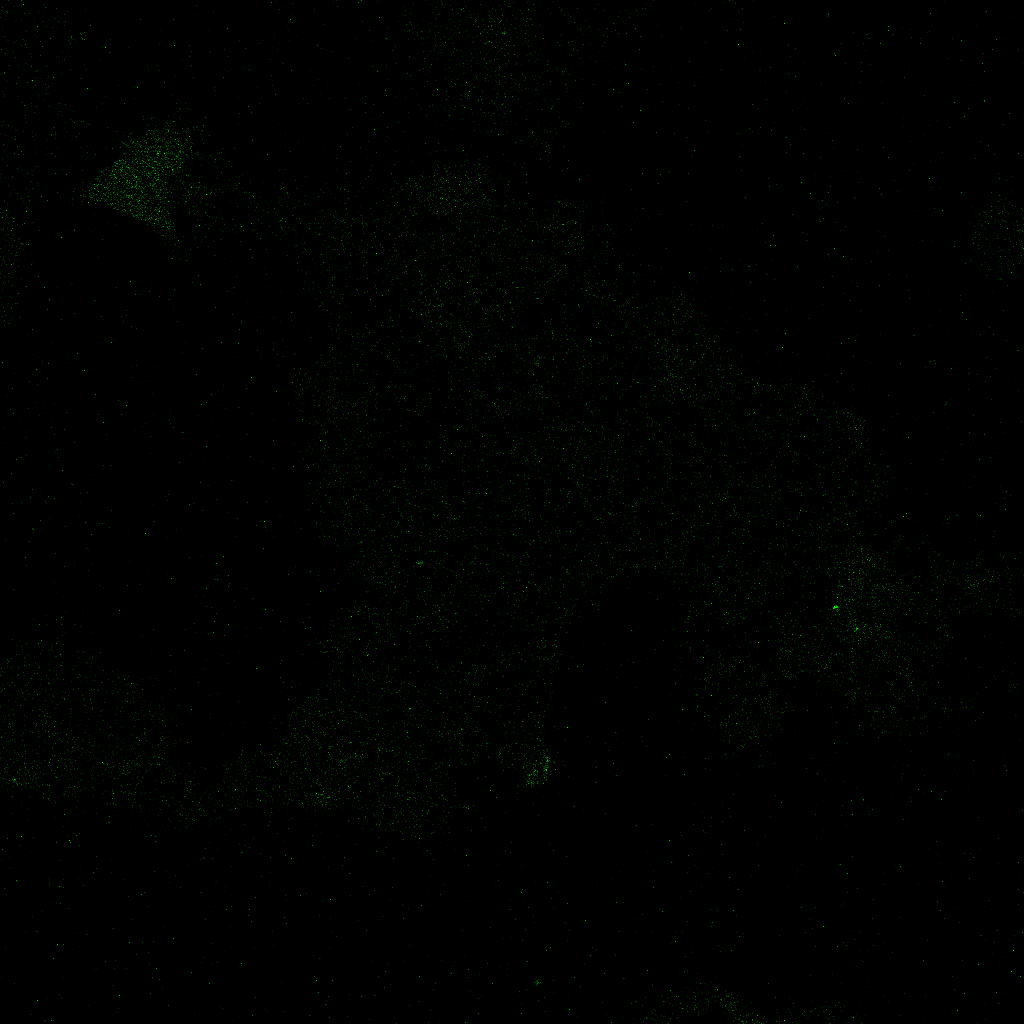

Supplement: Supplementary file 7 — Source data Fig. 5 [file 44321_2025_308_MOESM7_ESM.zip › Figure 5/5e/HCT116 Con 2/116 C2_116 C2_RGB_FITC.tif]

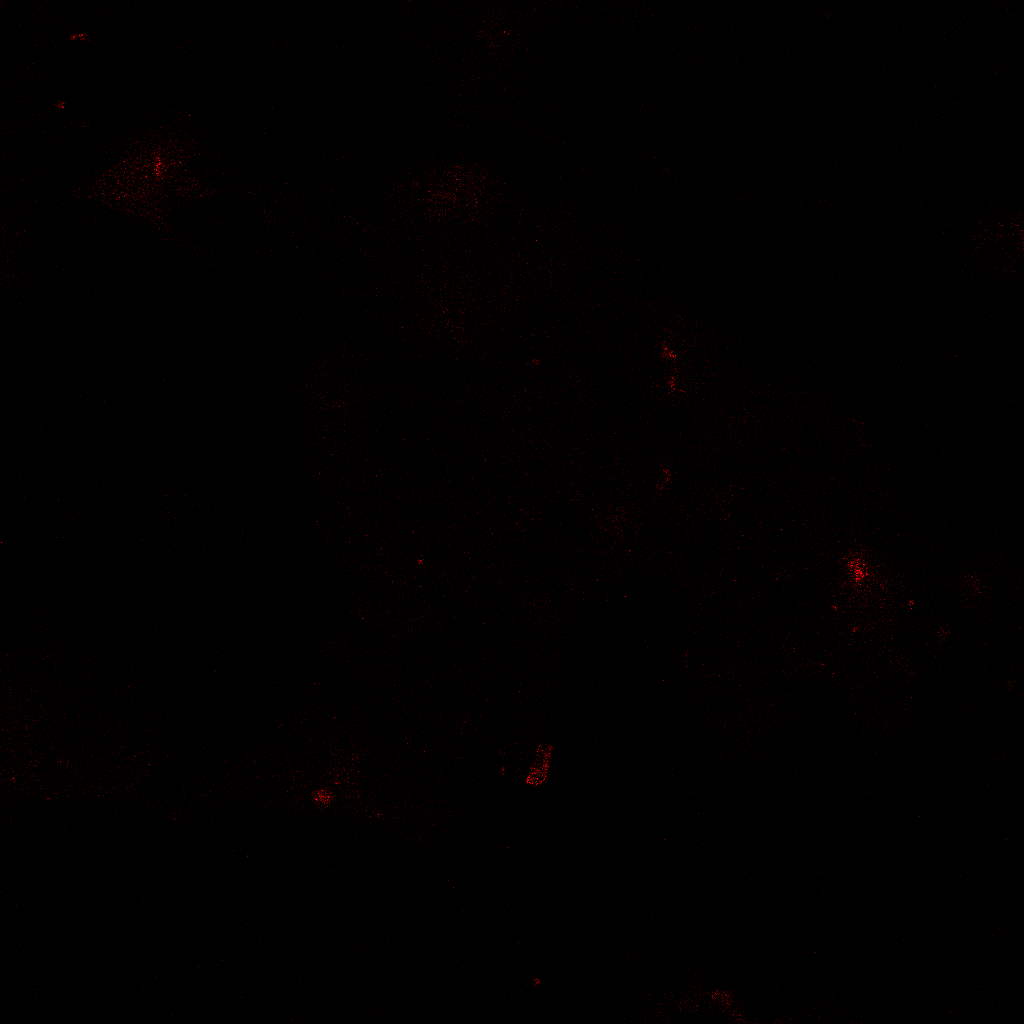

Supplement: Supplementary file 7 — Source data Fig. 5 [file 44321_2025_308_MOESM7_ESM.zip › Figure 5/5e/HCT116 Con 2/116 C2_116 C2_RGB_TRITC.tif]

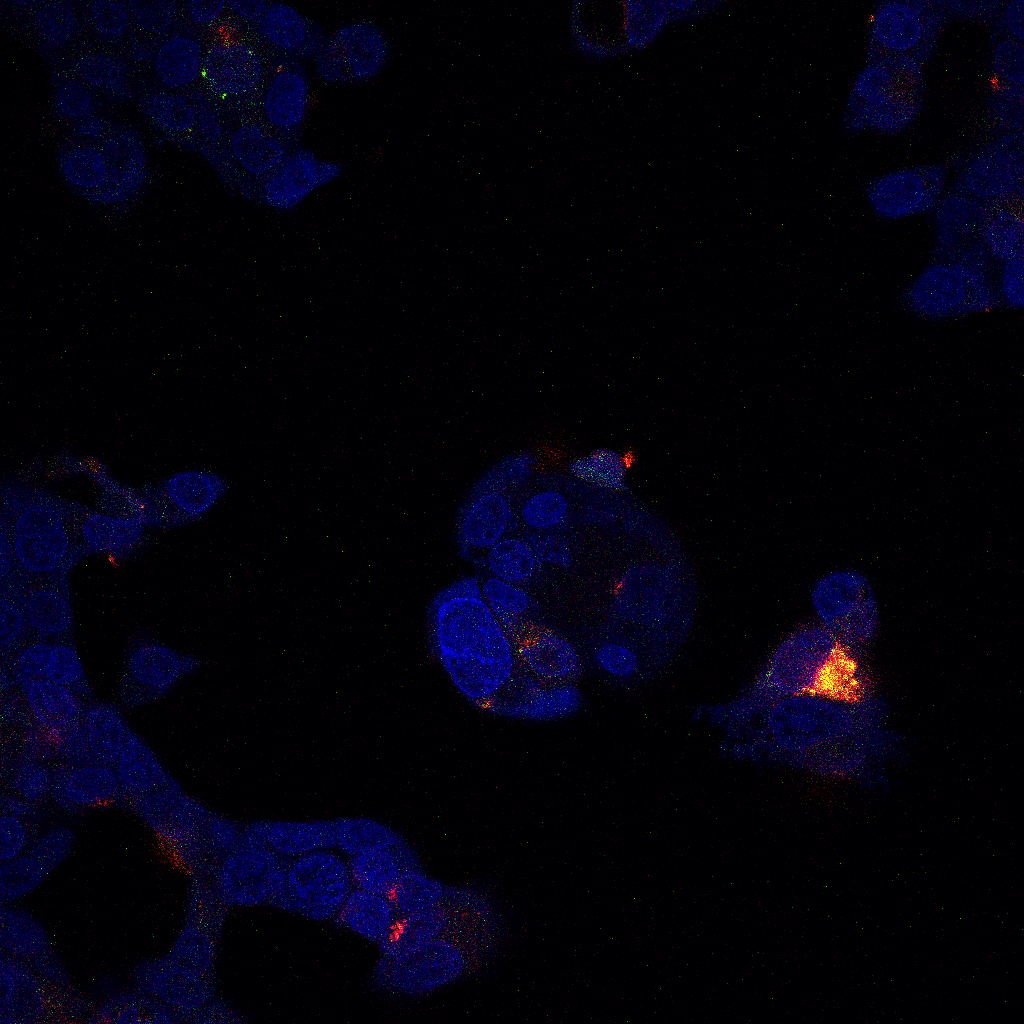

Supplement: Supplementary file 7 — Source data Fig. 5 [file 44321_2025_308_MOESM7_ESM.zip › Figure 5/5e/HCT116 Con 3/116 C3 _RGB.tif]

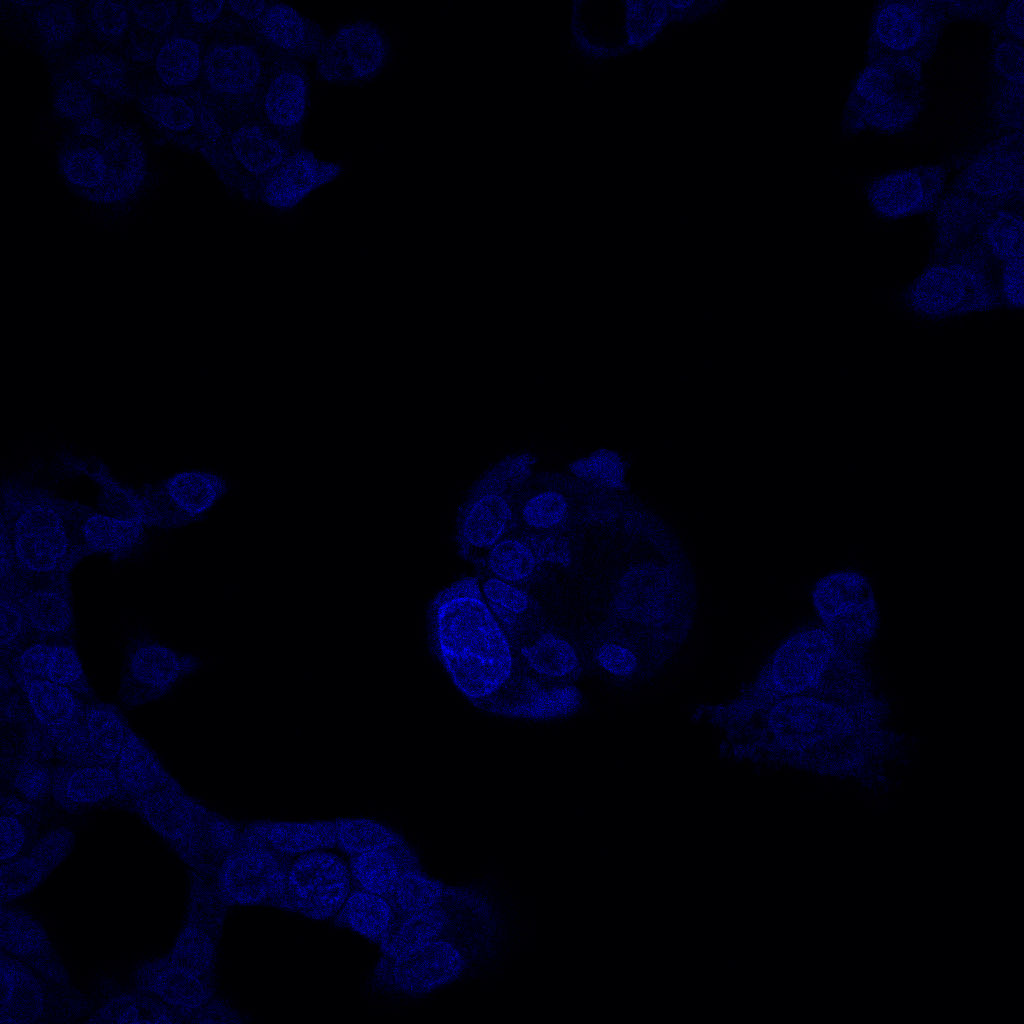

Supplement: Supplementary file 7 — Source data Fig. 5 [file 44321_2025_308_MOESM7_ESM.zip › Figure 5/5e/HCT116 Con 3/116 C3 _RGB_DAPI.tif]

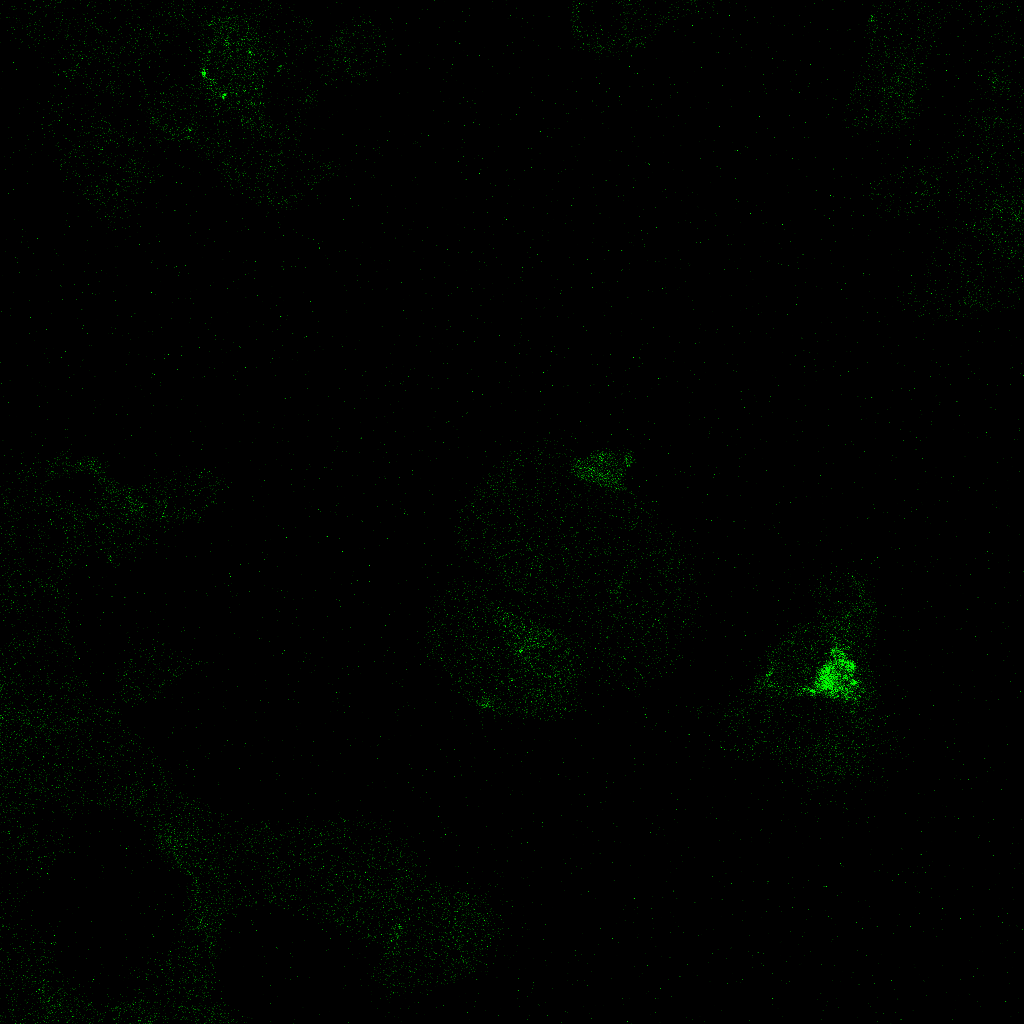

Supplement: Supplementary file 7 — Source data Fig. 5 [file 44321_2025_308_MOESM7_ESM.zip › Figure 5/5e/HCT116 Con 3/116 C3 _RGB_FITC.tif]

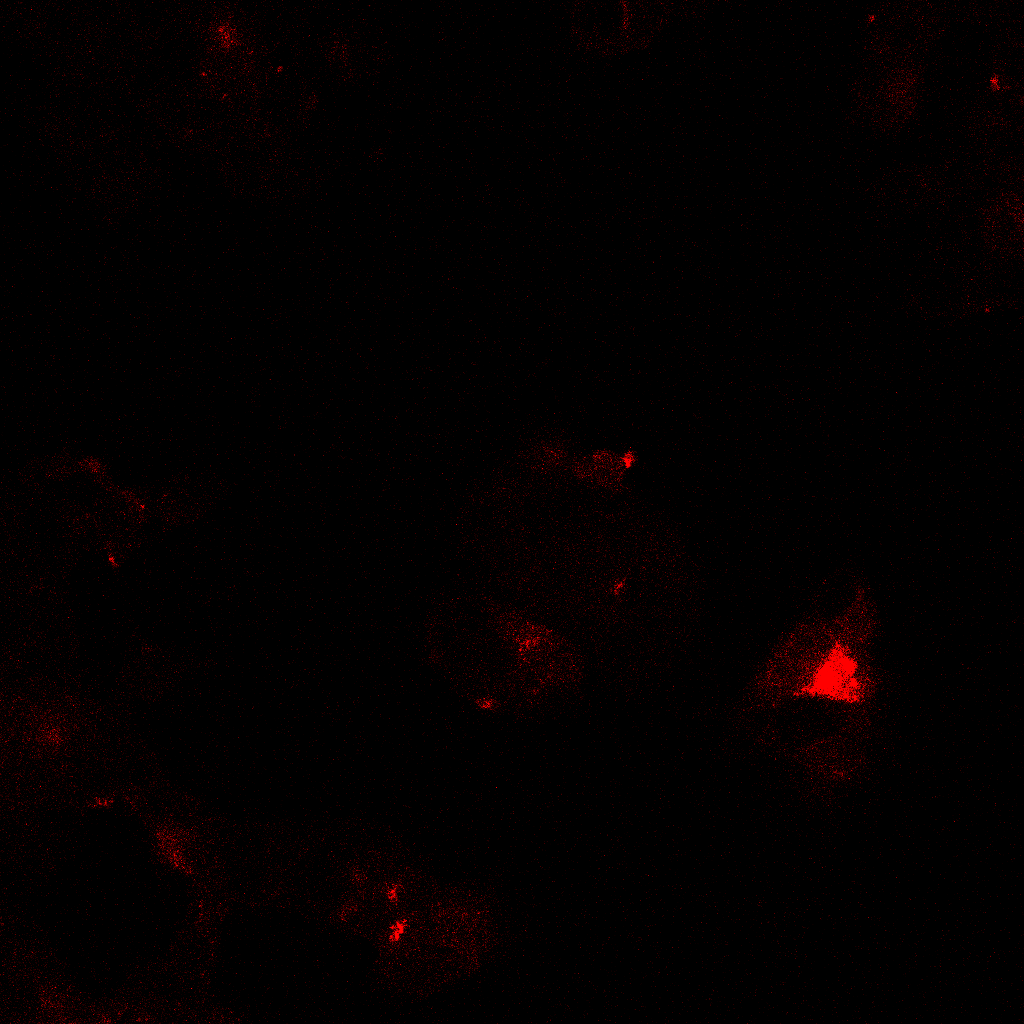

Supplement: Supplementary file 7 — Source data Fig. 5 [file 44321_2025_308_MOESM7_ESM.zip › Figure 5/5e/HCT116 Con 3/116 C3 _RGB_TRITC.tif]

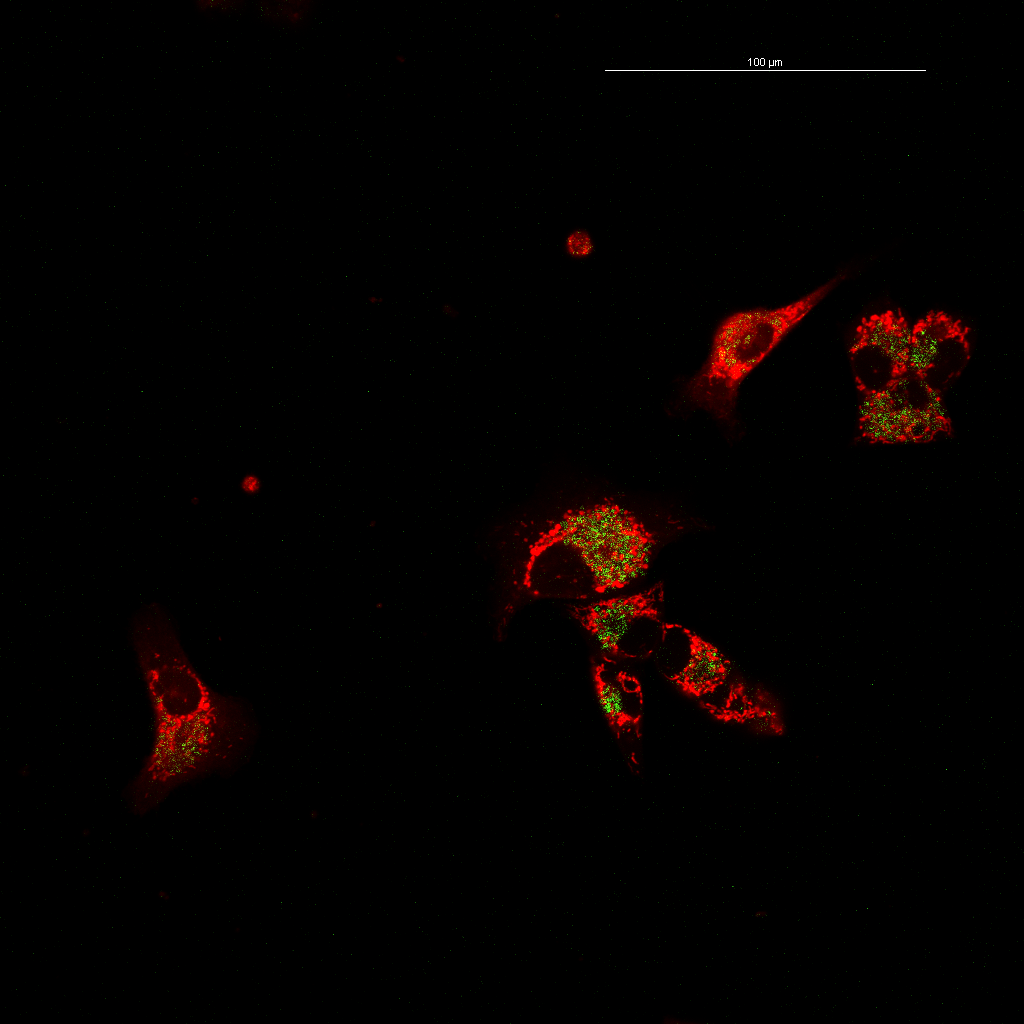

Supplement: Supplementary file 7 — Source data Fig. 5 [file 44321_2025_308_MOESM7_ESM.zip › Figure 5/5f/HCT116 0.5 1/7_7_RGB.tif]

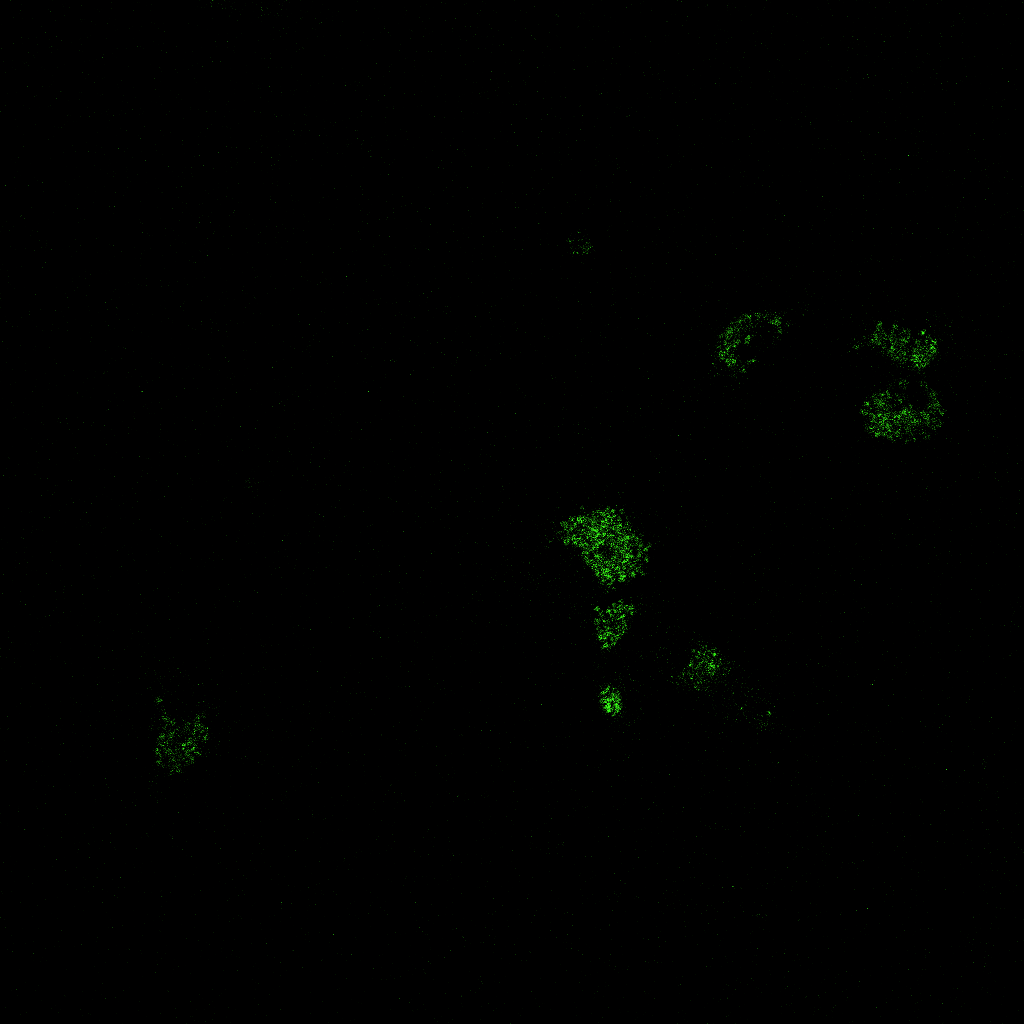

Supplement: Supplementary file 7 — Source data Fig. 5 [file 44321_2025_308_MOESM7_ESM.zip › Figure 5/5f/HCT116 0.5 1/7_7_RGB_LysoTracker Green.tif]

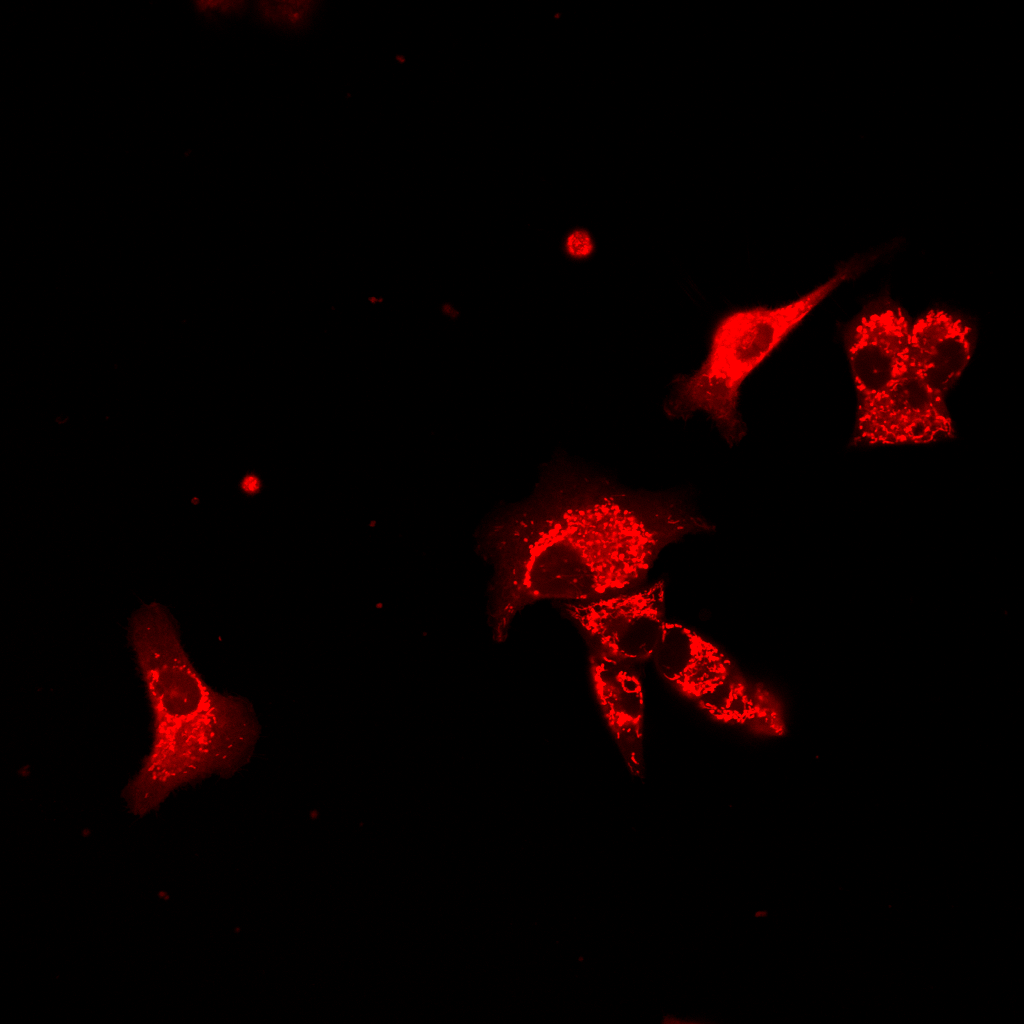

Supplement: Supplementary file 7 — Source data Fig. 5 [file 44321_2025_308_MOESM7_ESM.zip › Figure 5/5f/HCT116 0.5 1/7_7_RGB_TRITC.tif]

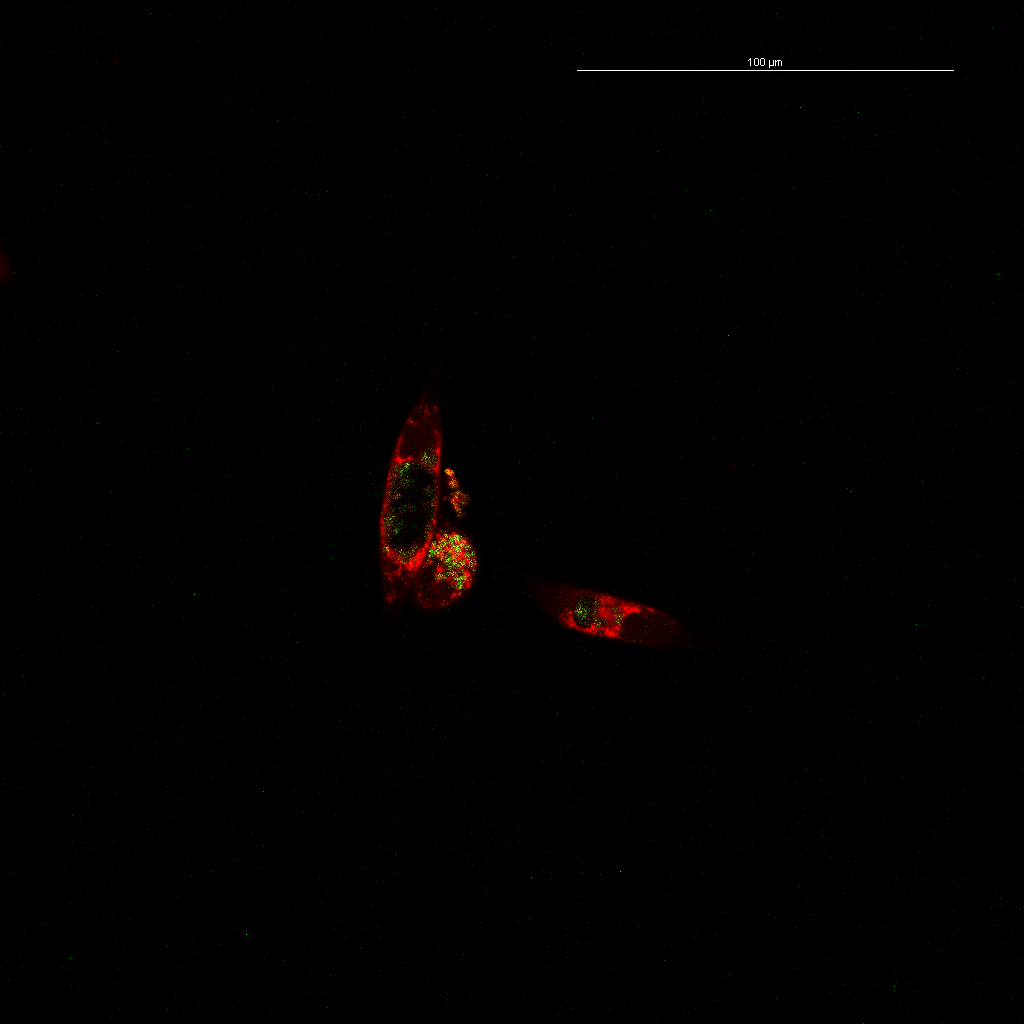

Supplement: Supplementary file 7 — Source data Fig. 5 [file 44321_2025_308_MOESM7_ESM.zip › Figure 5/5f/HCT116 0.5 2/4_4_RGB.tif]

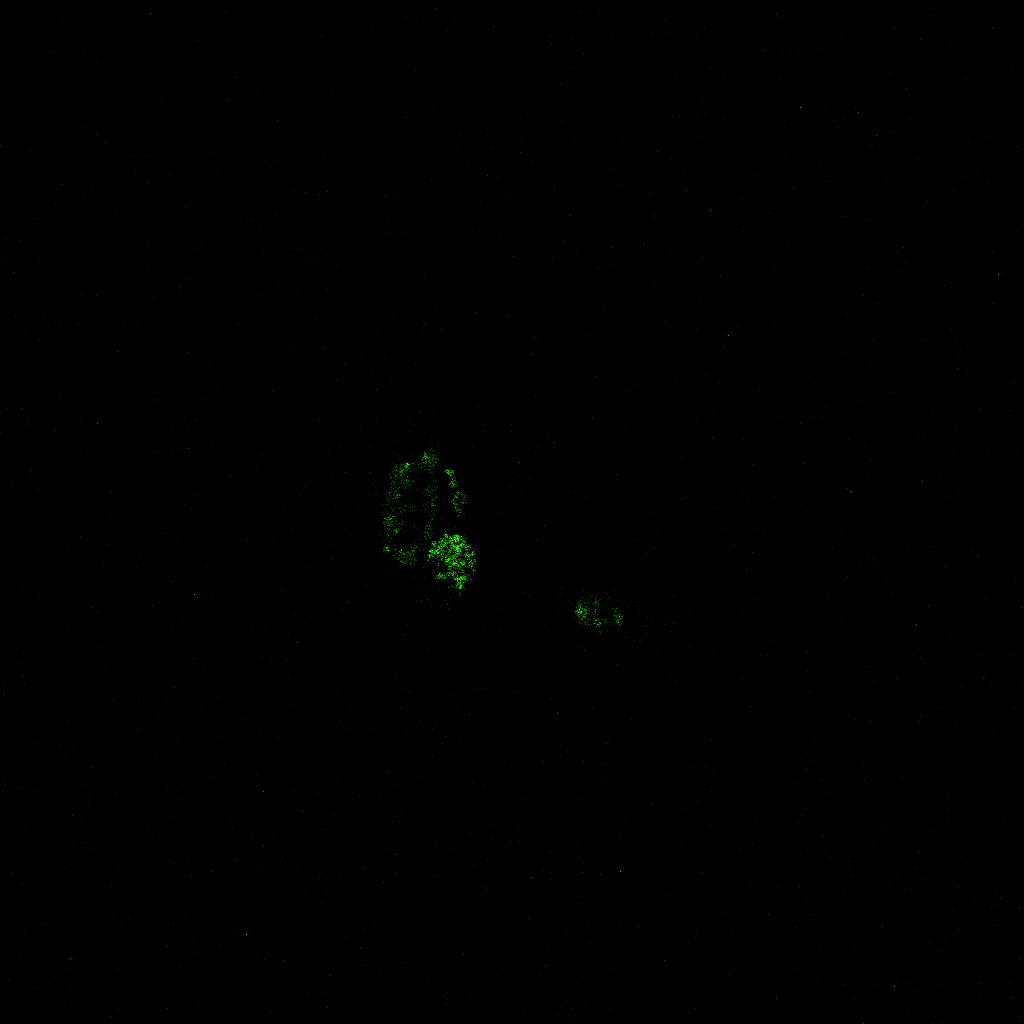

Supplement: Supplementary file 7 — Source data Fig. 5 [file 44321_2025_308_MOESM7_ESM.zip › Figure 5/5f/HCT116 0.5 2/4_4_RGB_LysoTracker Green.tif]

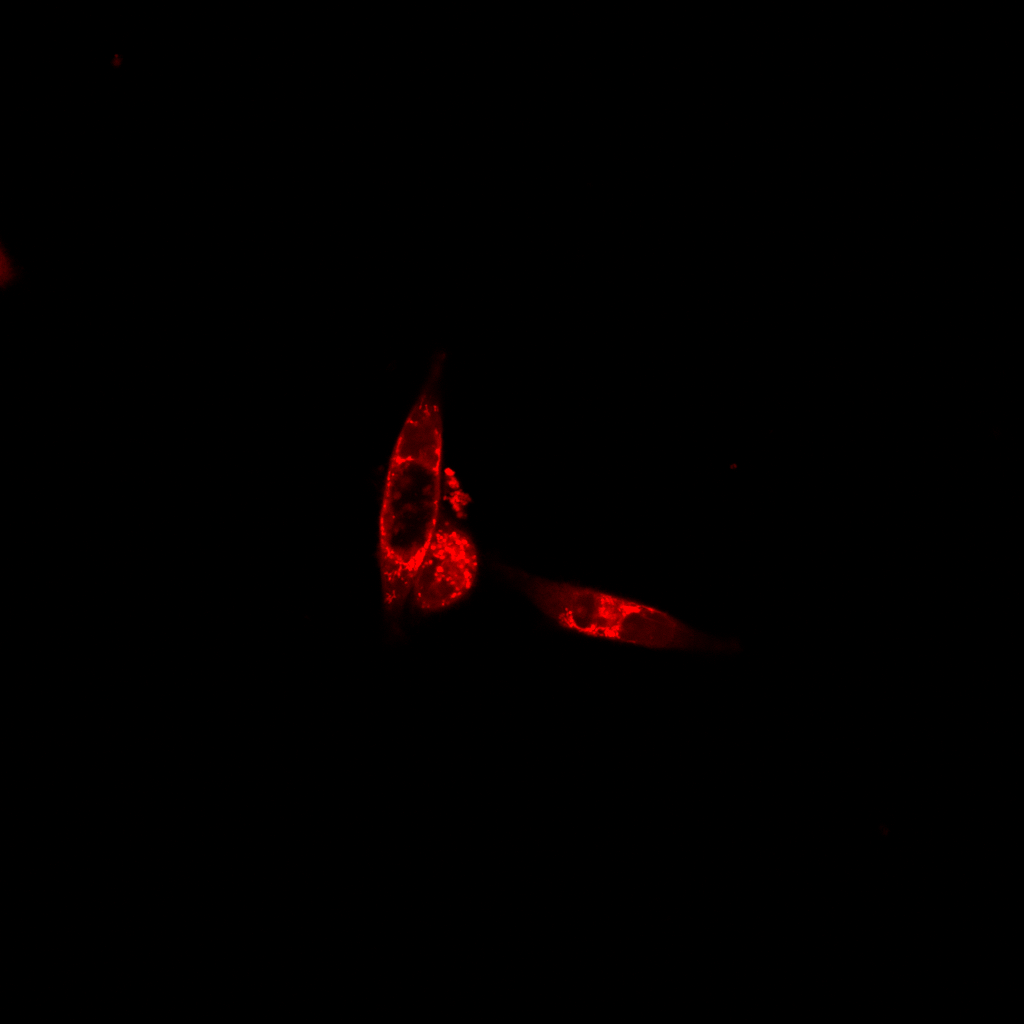

Supplement: Supplementary file 7 — Source data Fig. 5 [file 44321_2025_308_MOESM7_ESM.zip › Figure 5/5f/HCT116 0.5 2/4_4_RGB_TRITC.tif]

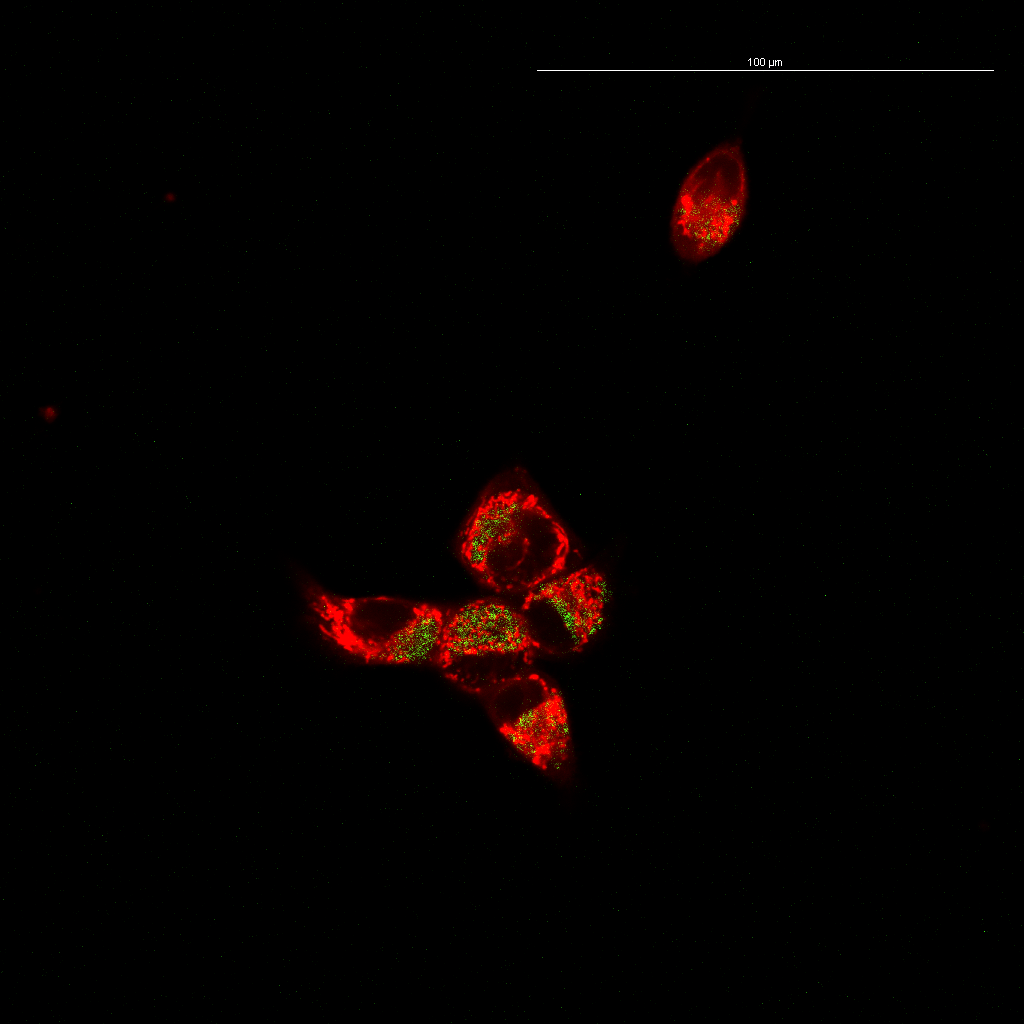

Supplement: Supplementary file 7 — Source data Fig. 5 [file 44321_2025_308_MOESM7_ESM.zip › Figure 5/5f/HCT116 0.5 3/3_3_RGB.tif]

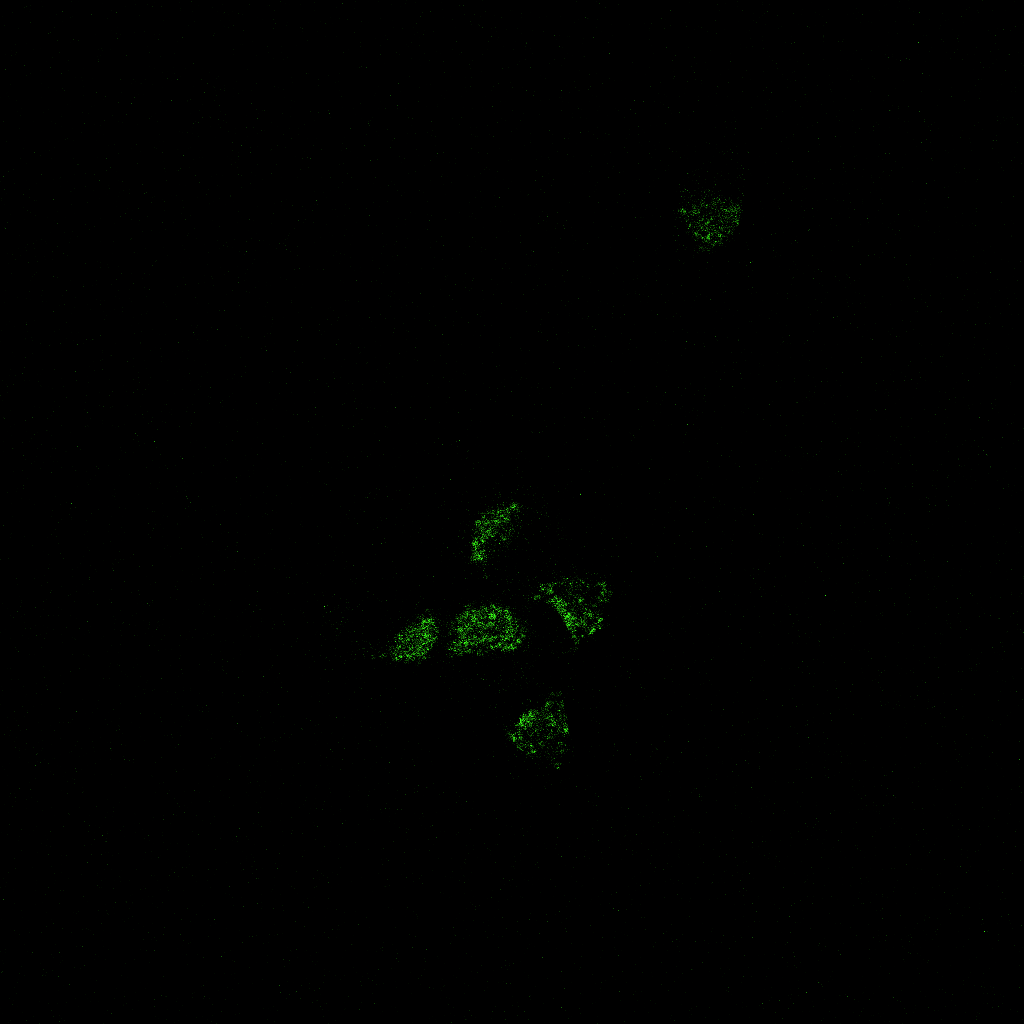

Supplement: Supplementary file 7 — Source data Fig. 5 [file 44321_2025_308_MOESM7_ESM.zip › Figure 5/5f/HCT116 0.5 3/3_3_RGB_LysoTracker Green.tif]

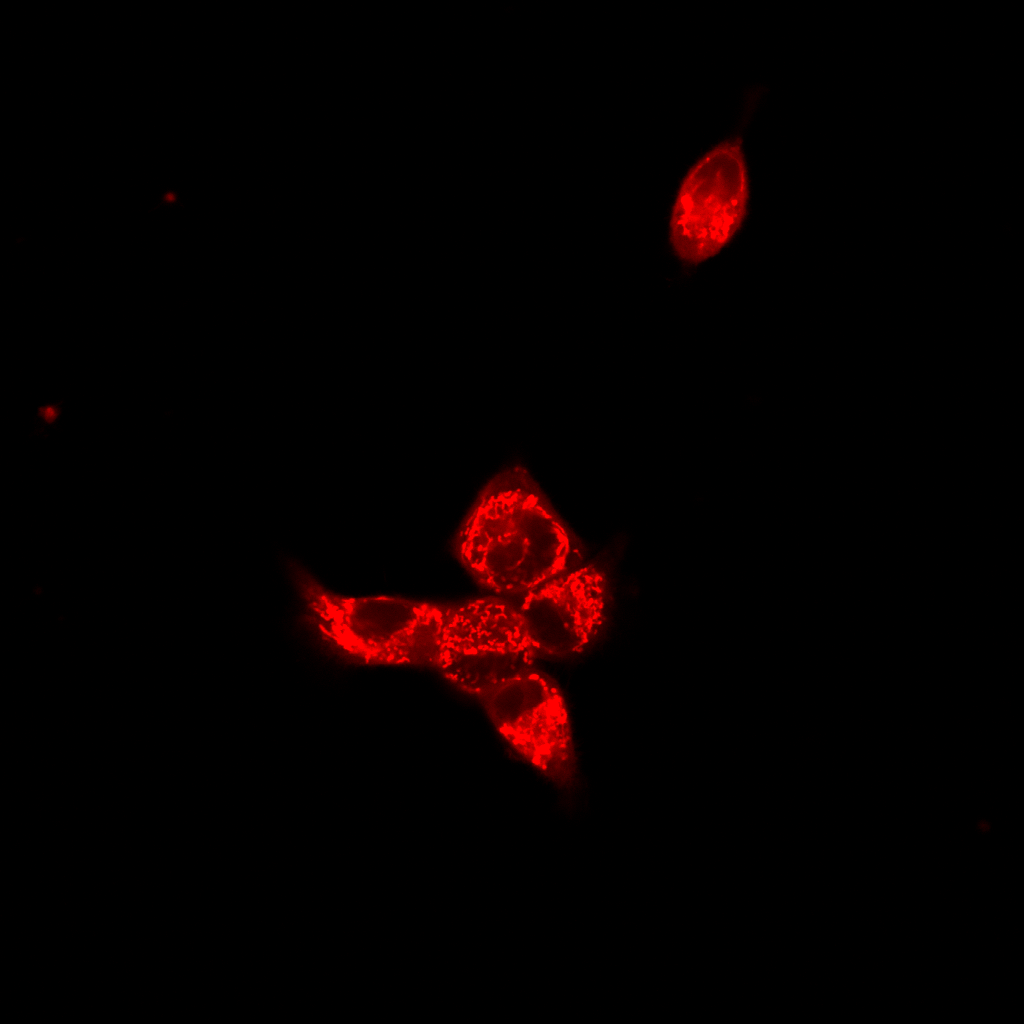

Supplement: Supplementary file 7 — Source data Fig. 5 [file 44321_2025_308_MOESM7_ESM.zip › Figure 5/5f/HCT116 0.5 3/3_3_RGB_TRITC.tif]

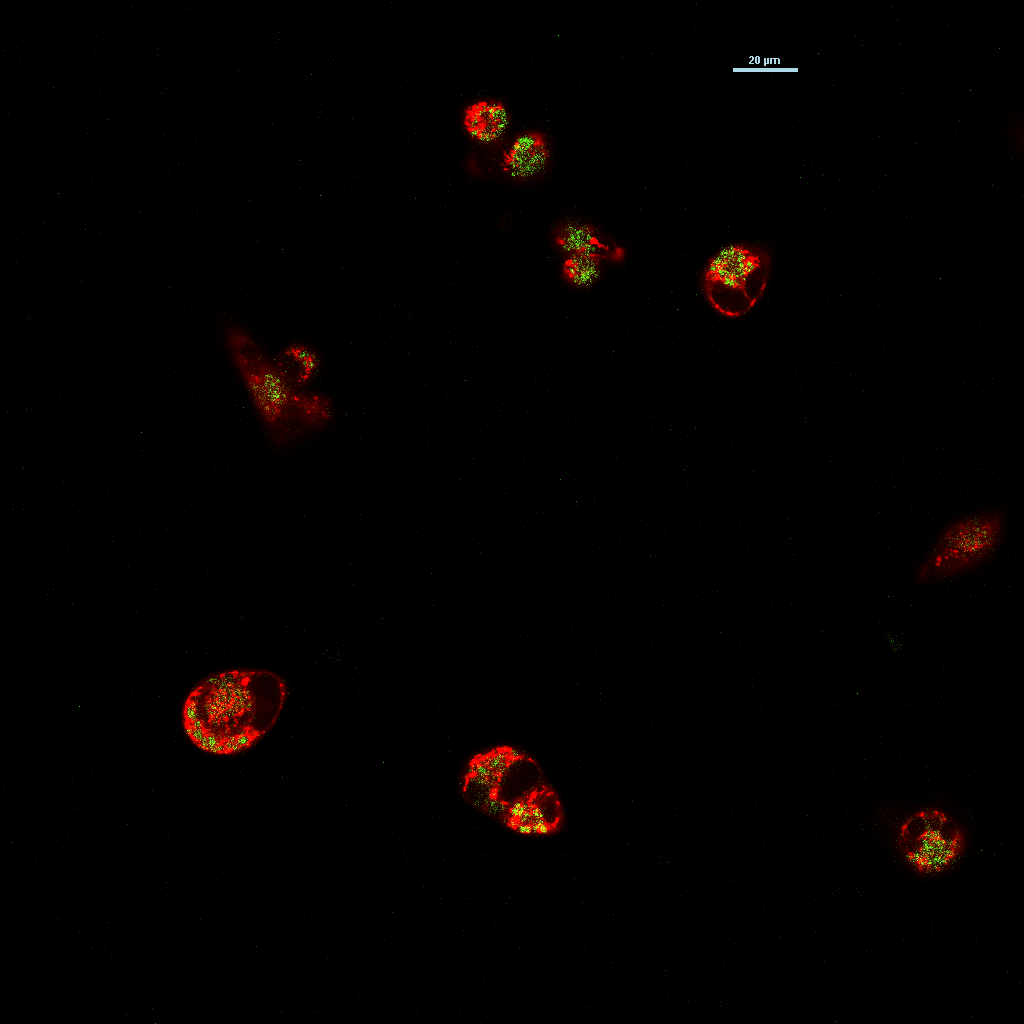

Supplement: Supplementary file 7 — Source data Fig. 5 [file 44321_2025_308_MOESM7_ESM.zip › Figure 5/5f/HCT116 1 1/7_7_RGB.tif]

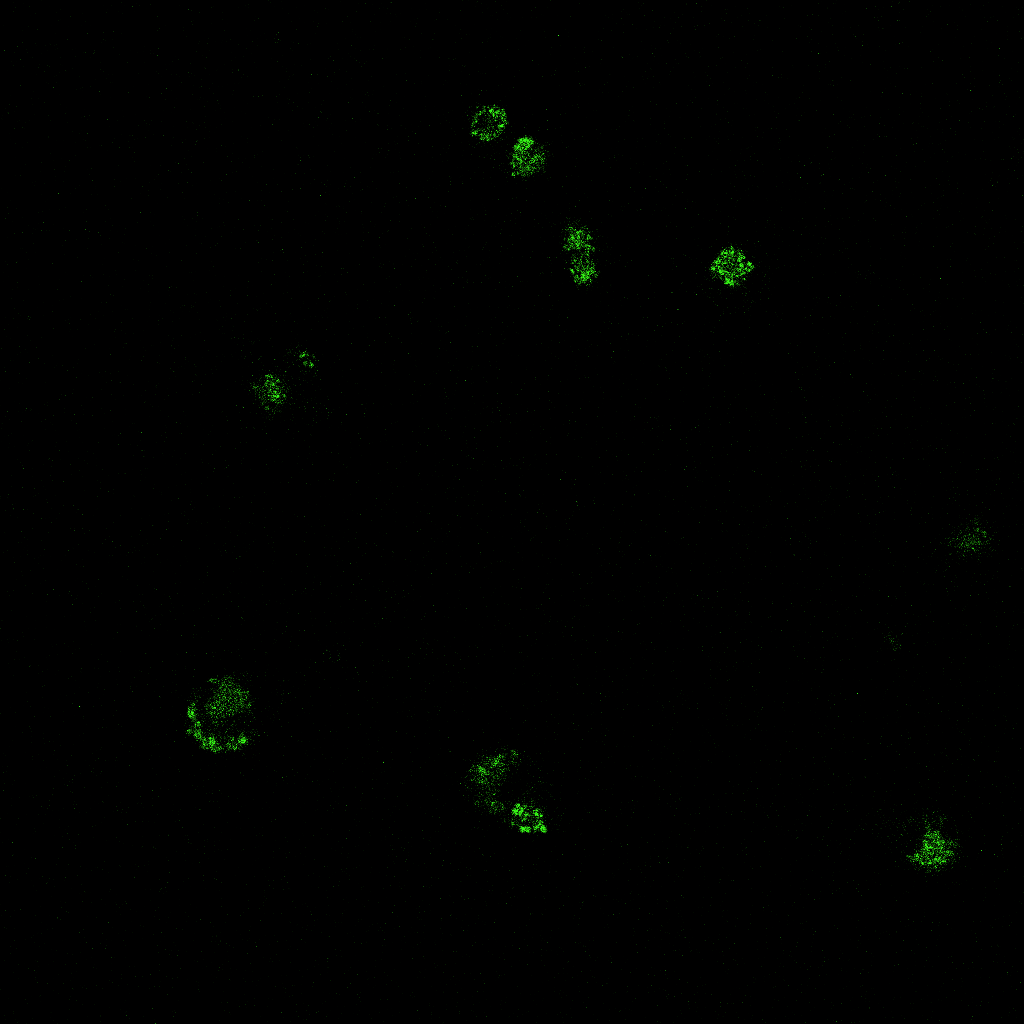

Supplement: Supplementary file 7 — Source data Fig. 5 [file 44321_2025_308_MOESM7_ESM.zip › Figure 5/5f/HCT116 1 1/7_7_RGB_LysoTracker Green.tif]

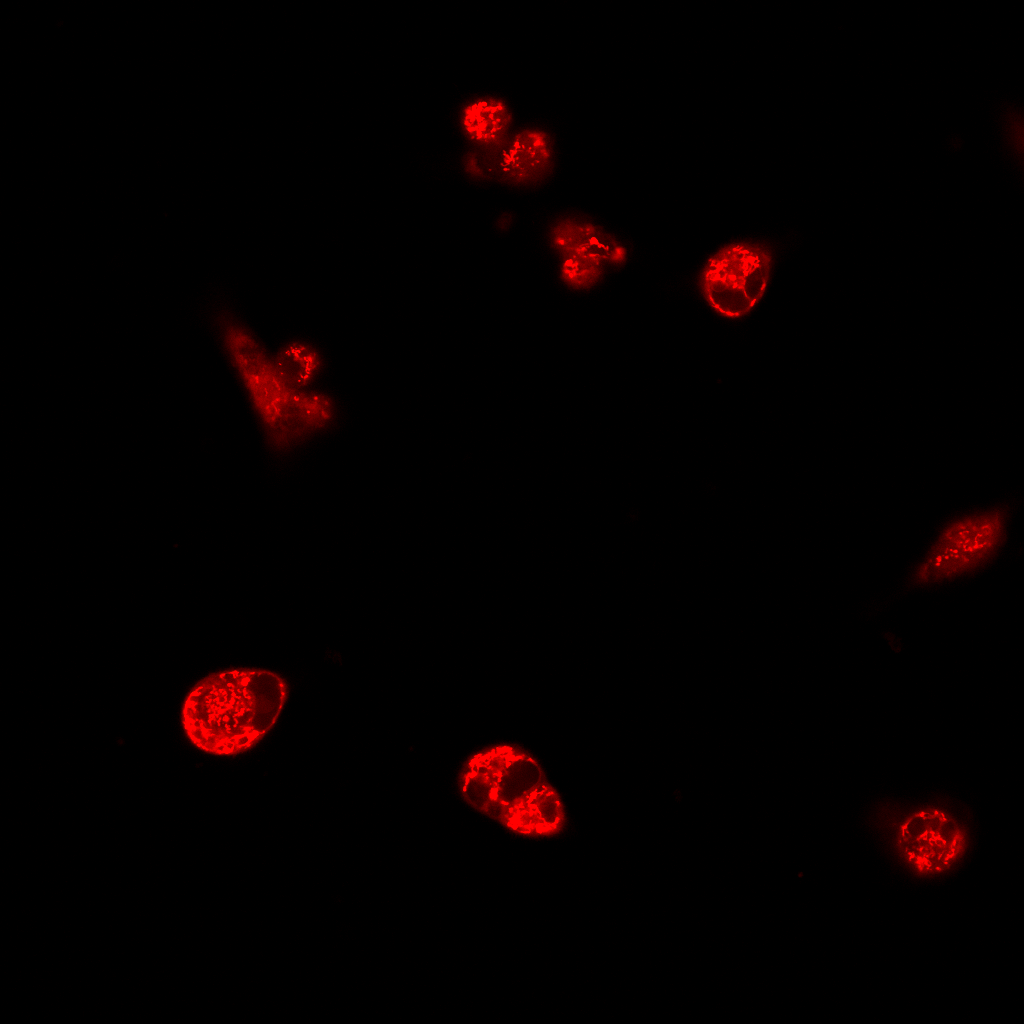

Supplement: Supplementary file 7 — Source data Fig. 5 [file 44321_2025_308_MOESM7_ESM.zip › Figure 5/5f/HCT116 1 1/7_7_RGB_TRITC.tif]

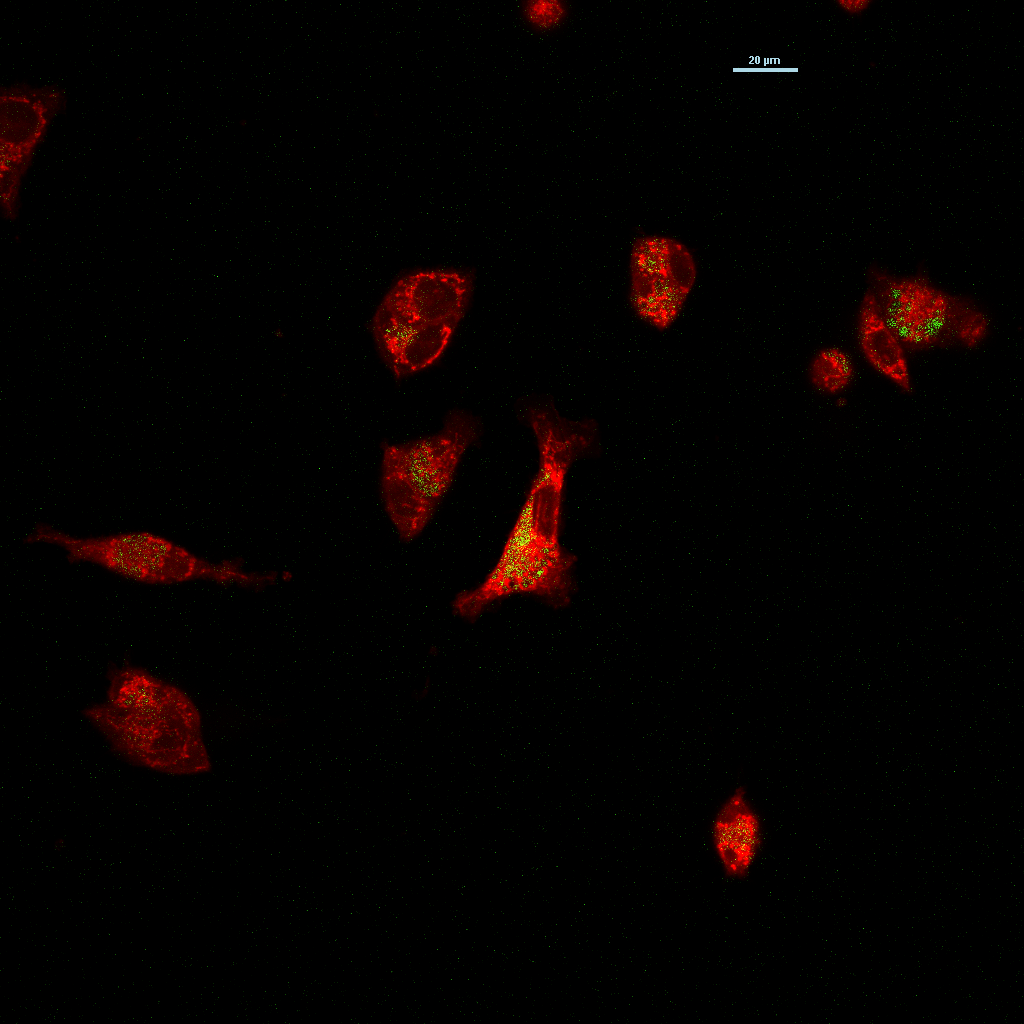

Supplement: Supplementary file 7 — Source data Fig. 5 [file 44321_2025_308_MOESM7_ESM.zip › Figure 5/5f/HCT116 1 2/8_8_RGB.tif]

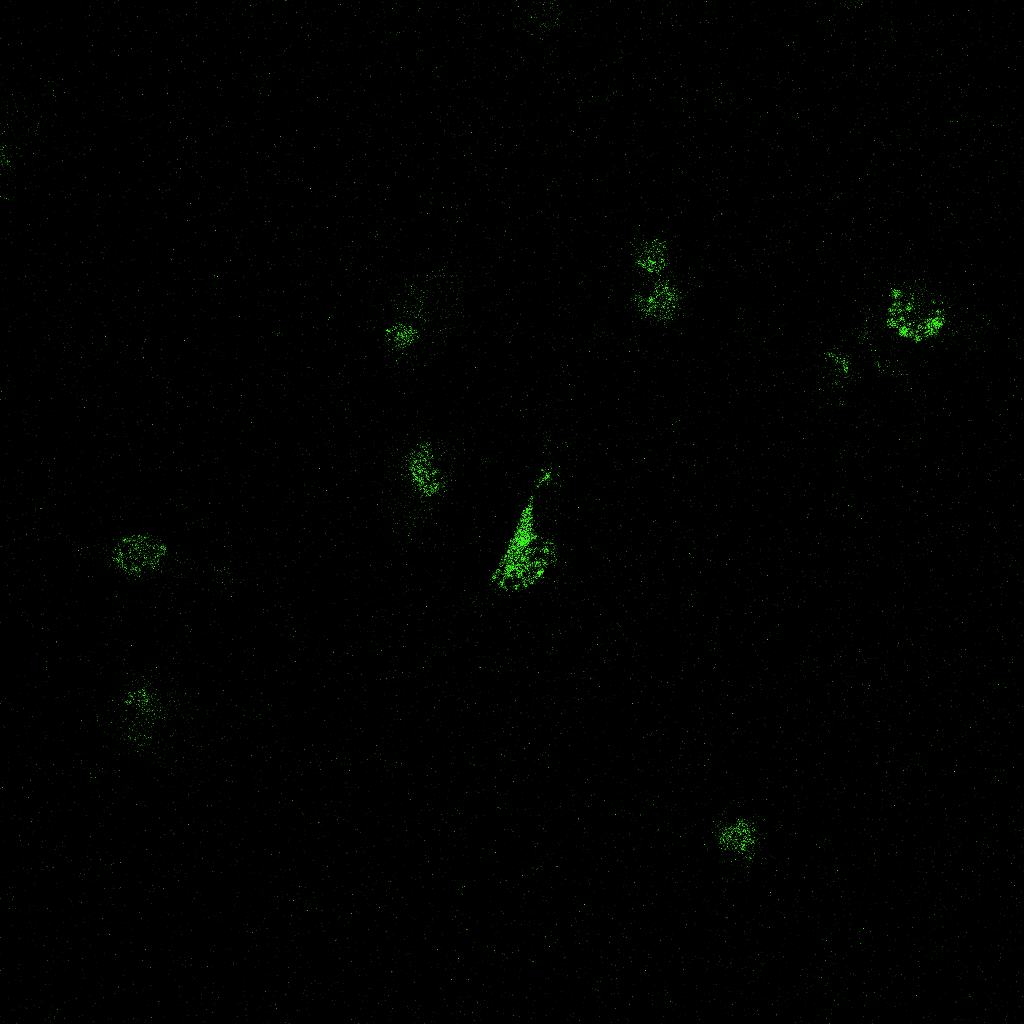

Supplement: Supplementary file 7 — Source data Fig. 5 [file 44321_2025_308_MOESM7_ESM.zip › Figure 5/5f/HCT116 1 2/8_8_RGB_LysoTracker Green.tif]

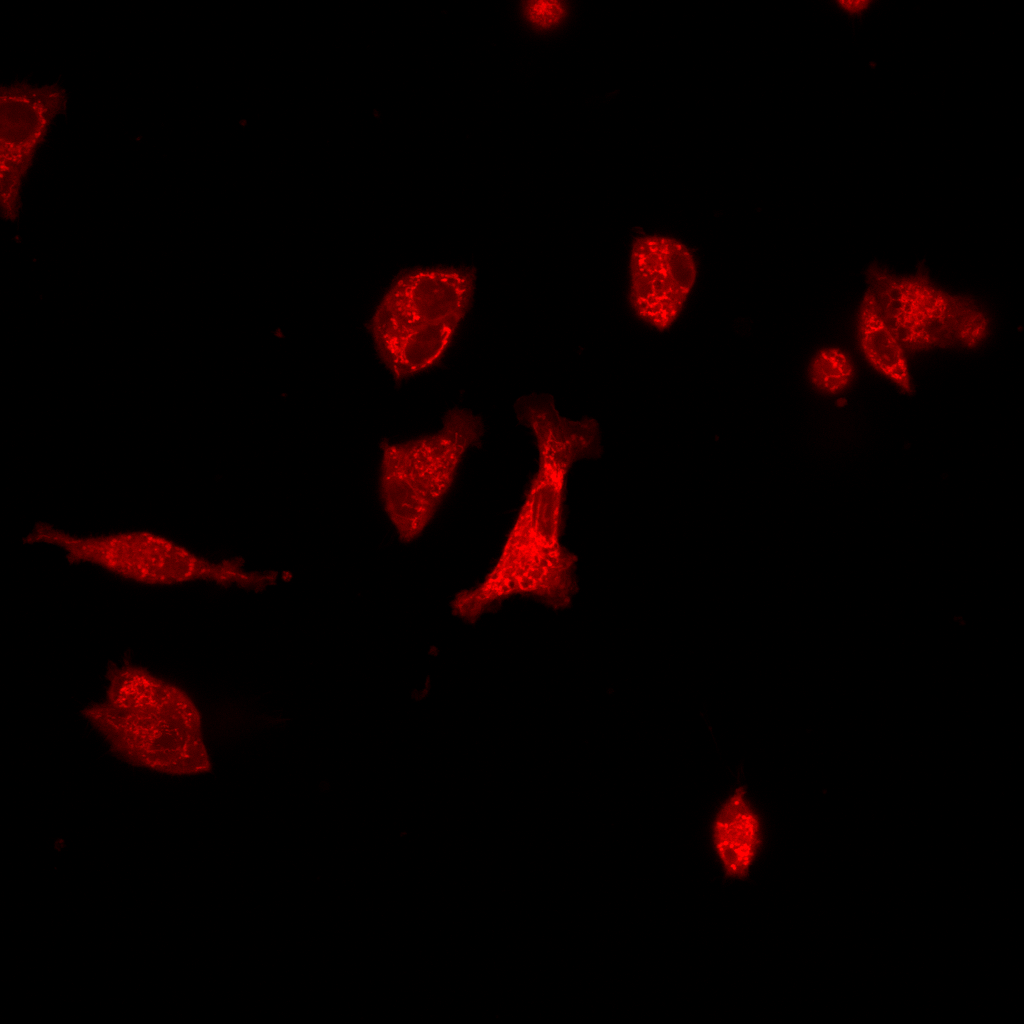

Supplement: Supplementary file 7 — Source data Fig. 5 [file 44321_2025_308_MOESM7_ESM.zip › Figure 5/5f/HCT116 1 2/8_8_RGB_TRITC.tif]

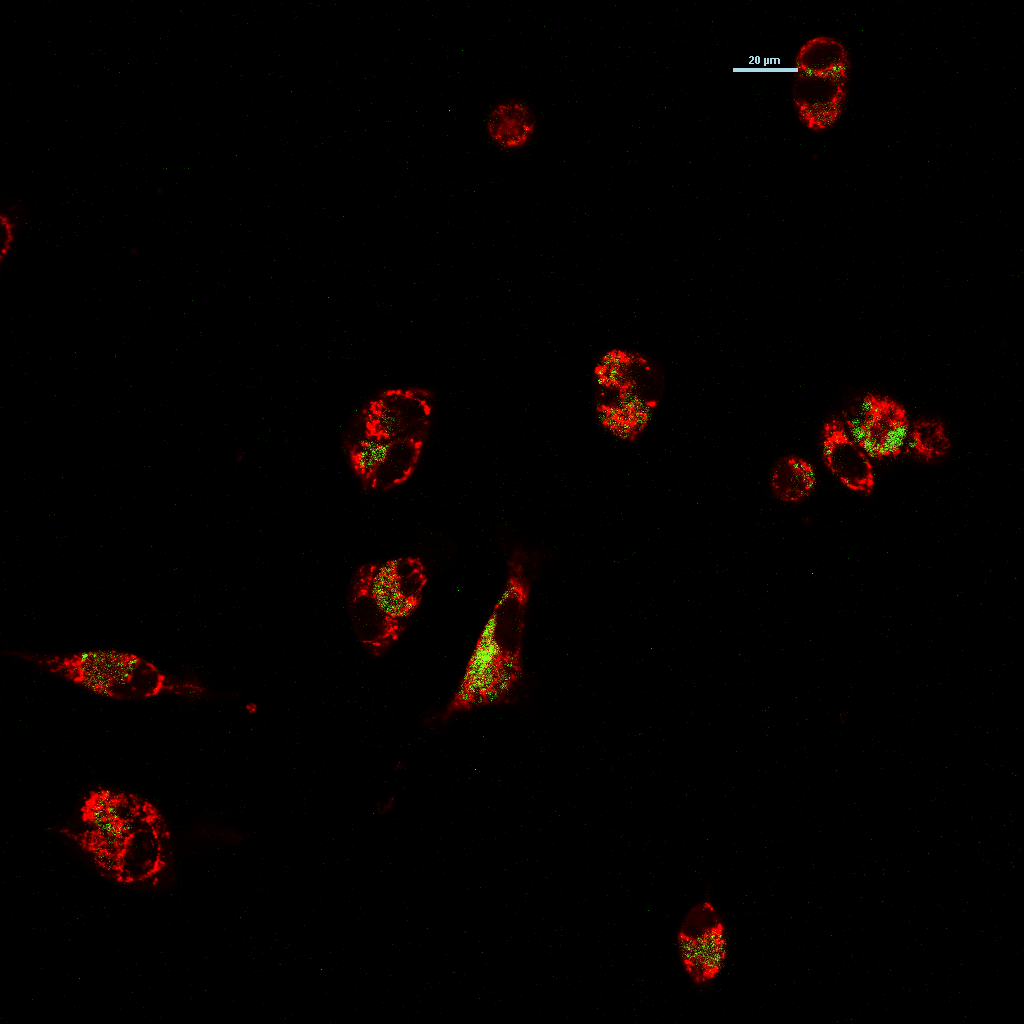

Supplement: Supplementary file 7 — Source data Fig. 5 [file 44321_2025_308_MOESM7_ESM.zip › Figure 5/5f/HCT116 1 3/5_5_RGB.tif]

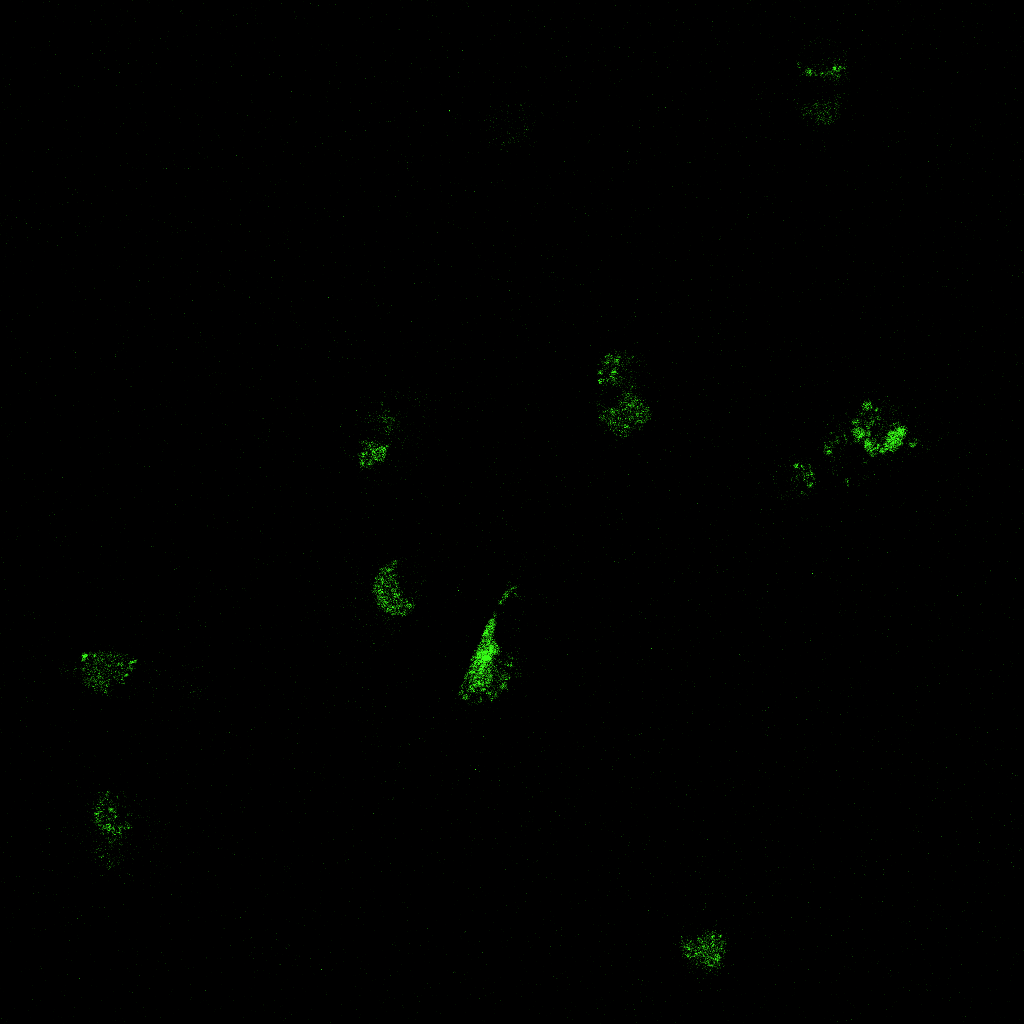

Supplement: Supplementary file 7 — Source data Fig. 5 [file 44321_2025_308_MOESM7_ESM.zip › Figure 5/5f/HCT116 1 3/5_5_RGB_LysoTracker Green.tif]

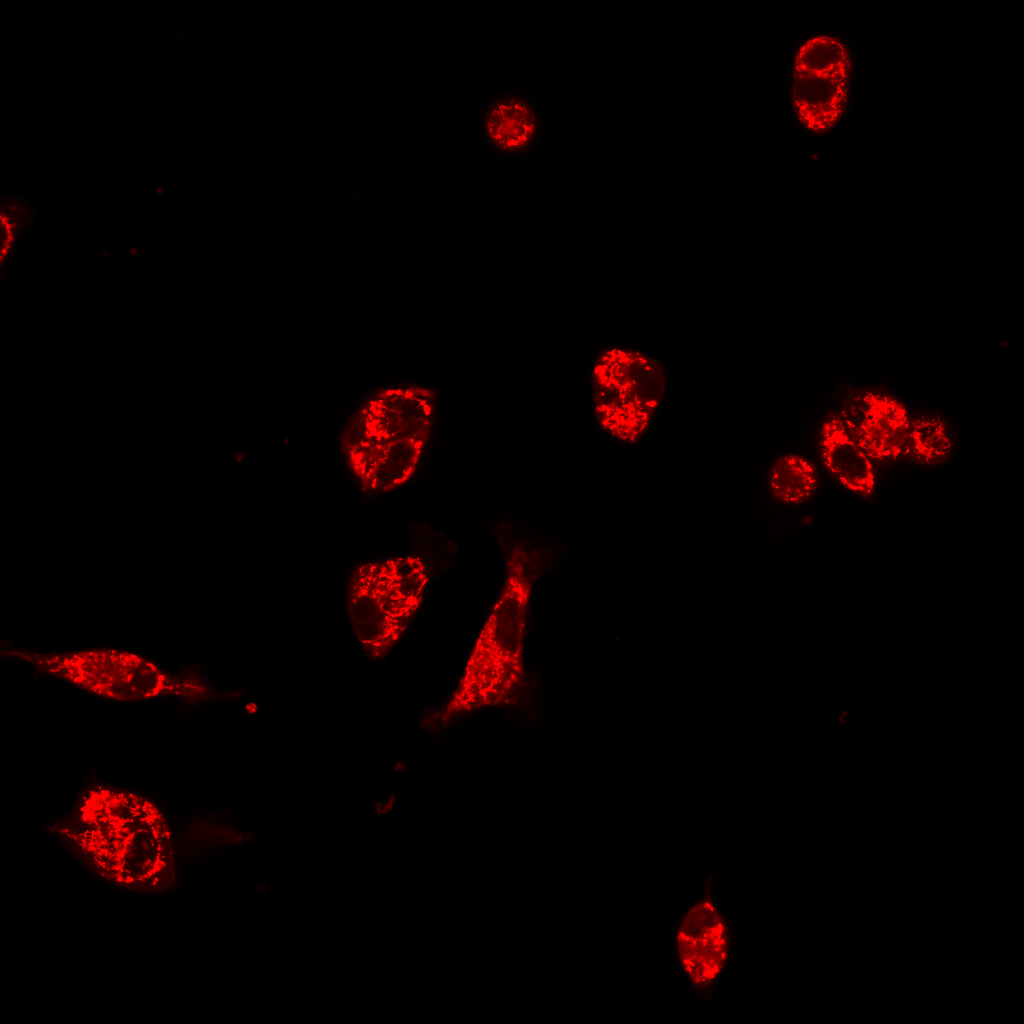

Supplement: Supplementary file 7 — Source data Fig. 5 [file 44321_2025_308_MOESM7_ESM.zip › Figure 5/5f/HCT116 1 3/5_5_RGB_TRITC.tif]

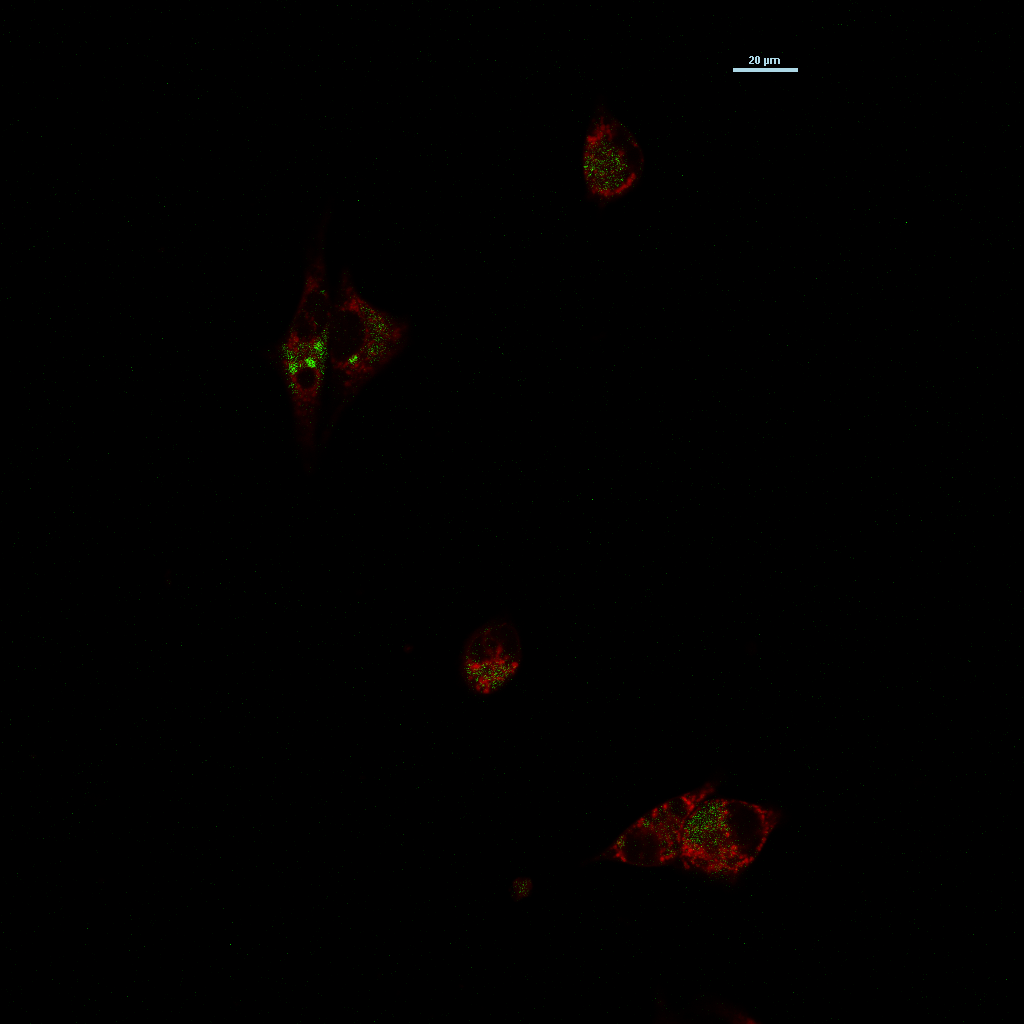

Supplement: Supplementary file 7 — Source data Fig. 5 [file 44321_2025_308_MOESM7_ESM.zip › Figure 5/5f/HCT116 Con 1/1_1_RGB.tif]

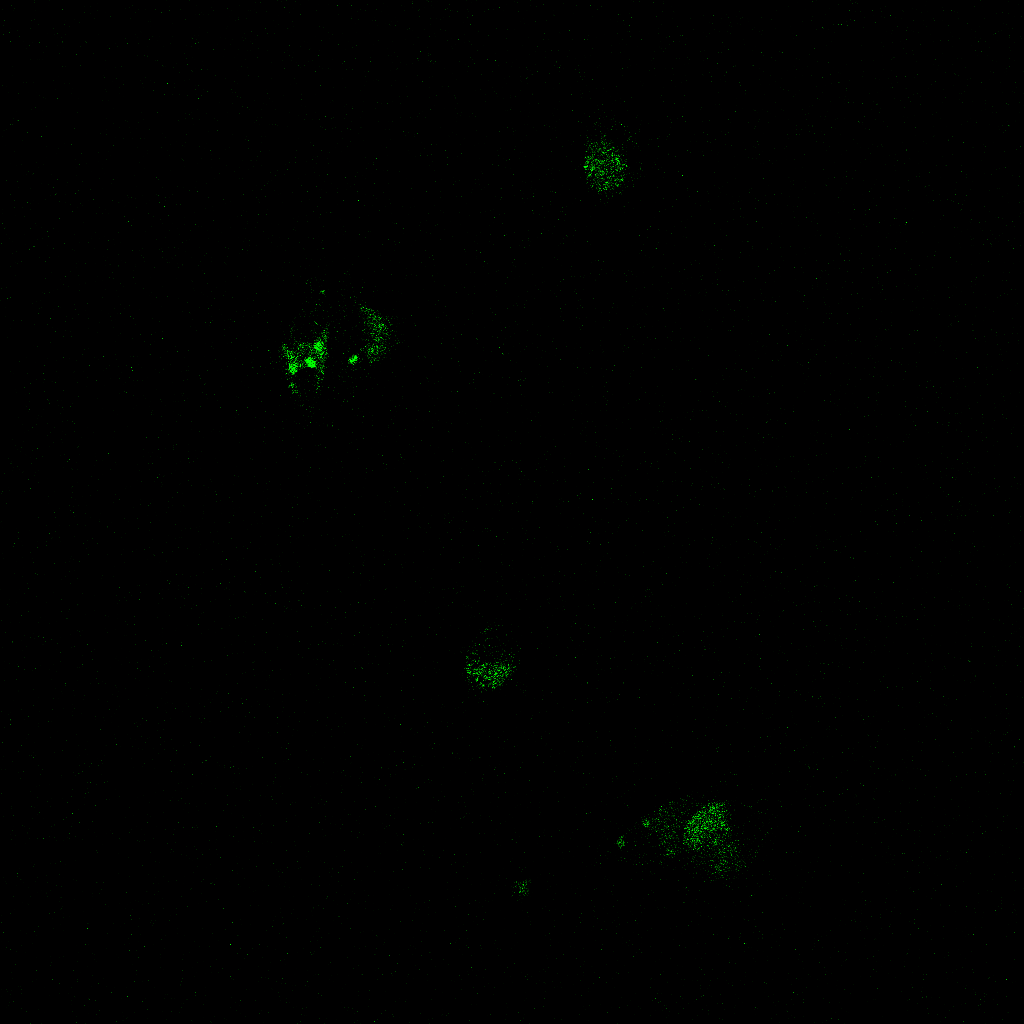

Supplement: Supplementary file 7 — Source data Fig. 5 [file 44321_2025_308_MOESM7_ESM.zip › Figure 5/5f/HCT116 Con 1/1_1_RGB_FITC.tif]

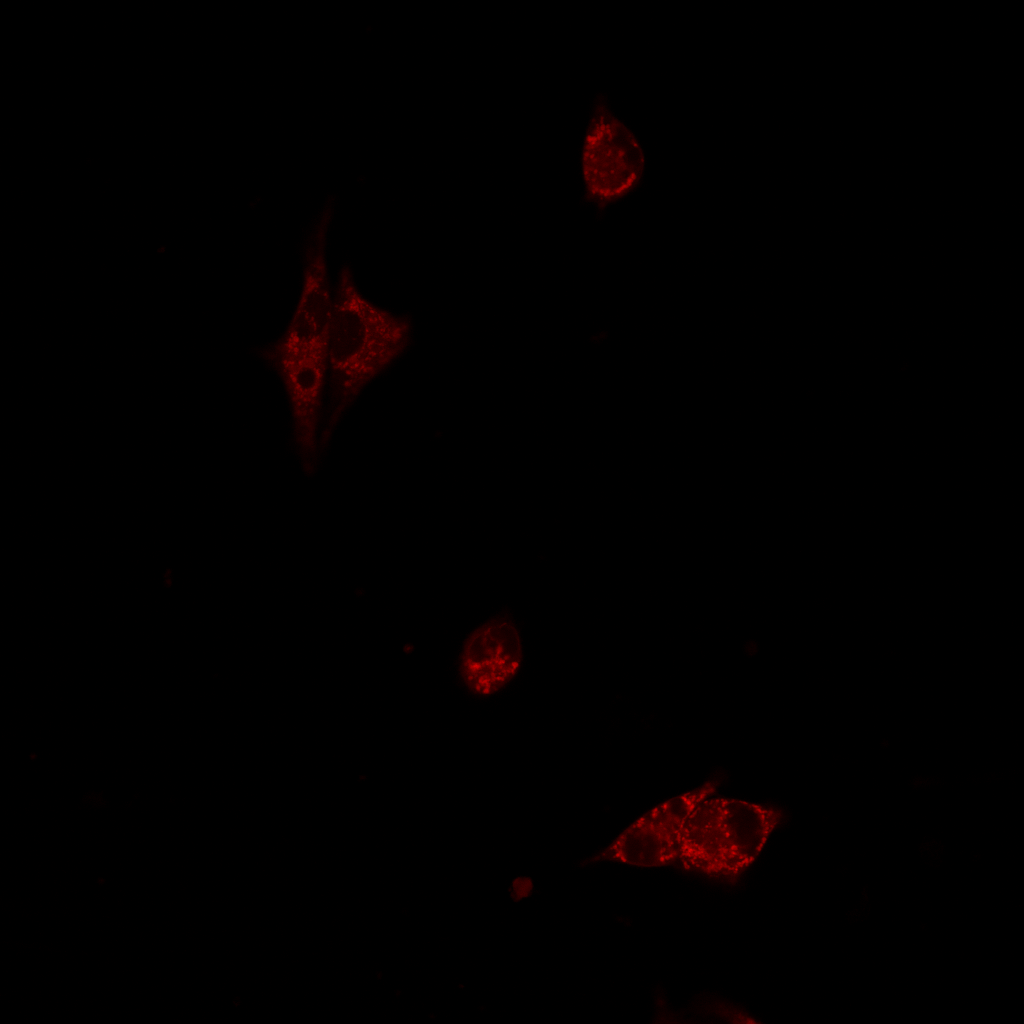

Supplement: Supplementary file 7 — Source data Fig. 5 [file 44321_2025_308_MOESM7_ESM.zip › Figure 5/5f/HCT116 Con 1/1_1_RGB_TRITC.tif]

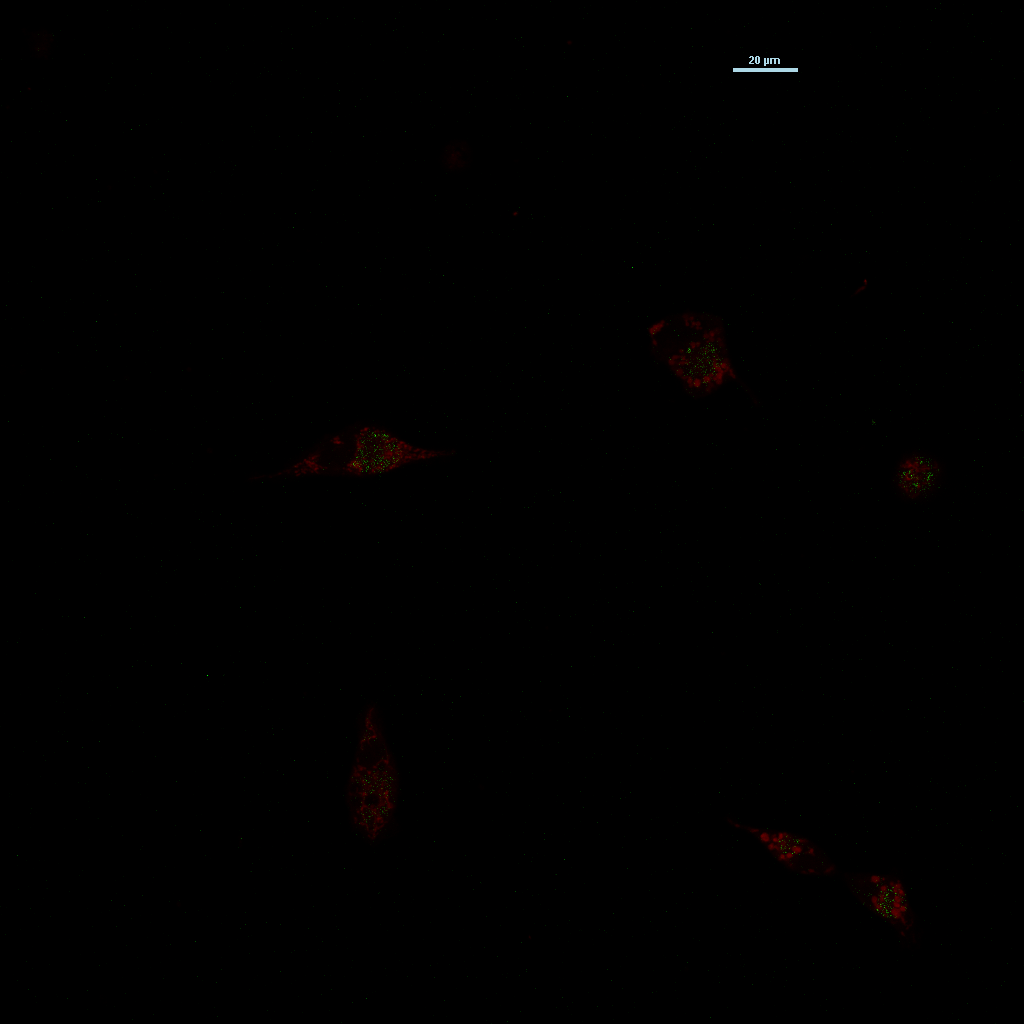

Supplement: Supplementary file 7 — Source data Fig. 5 [file 44321_2025_308_MOESM7_ESM.zip › Figure 5/5f/HCT116 Con 2/2_2_RGB.tif]

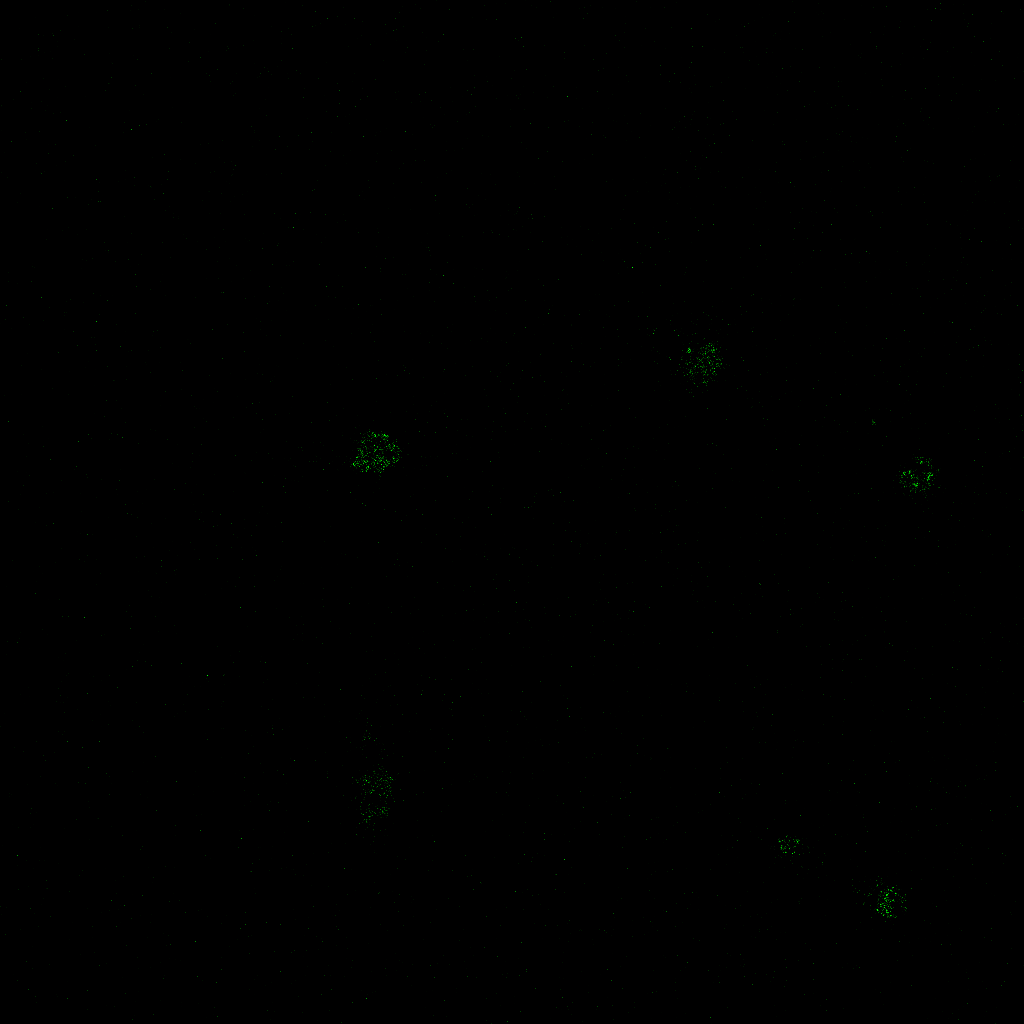

Supplement: Supplementary file 7 — Source data Fig. 5 [file 44321_2025_308_MOESM7_ESM.zip › Figure 5/5f/HCT116 Con 2/2_2_RGB_FITC.tif]

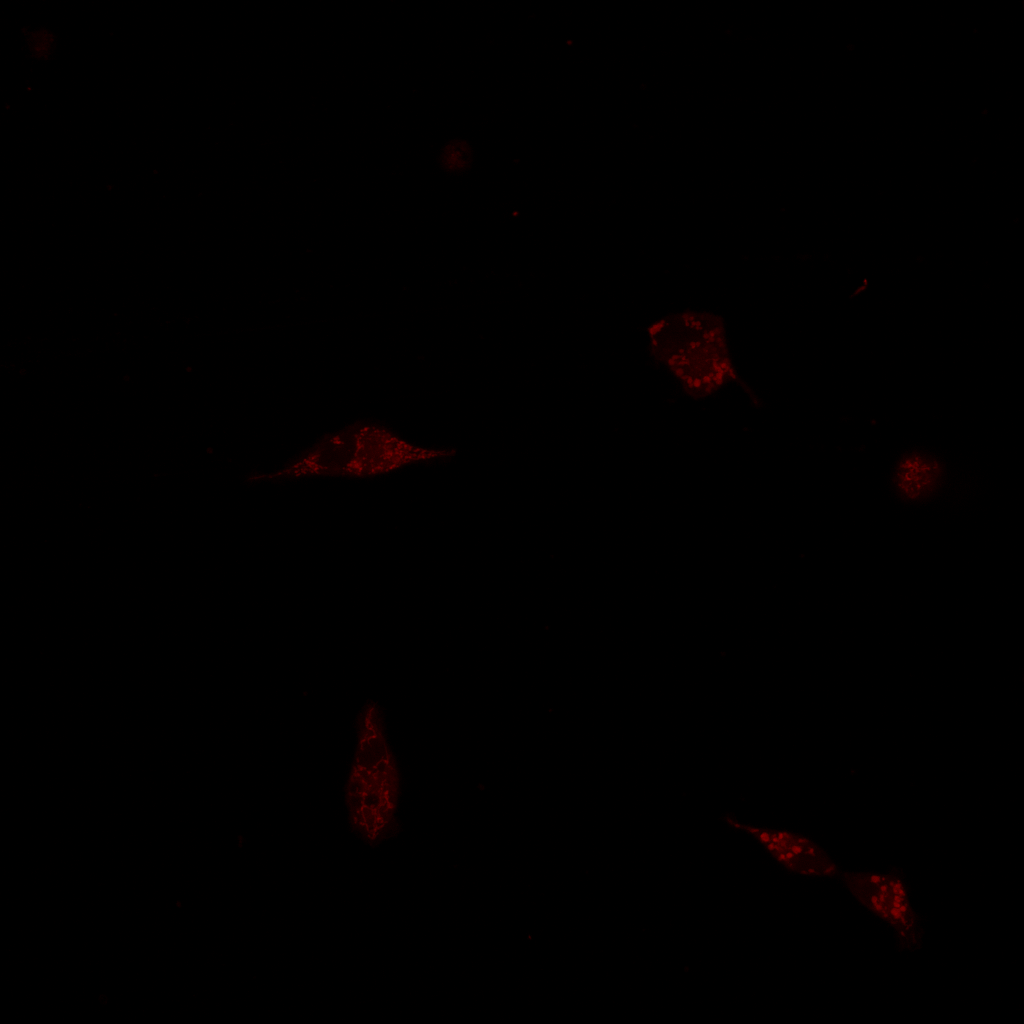

Supplement: Supplementary file 7 — Source data Fig. 5 [file 44321_2025_308_MOESM7_ESM.zip › Figure 5/5f/HCT116 Con 2/2_2_RGB_TRITC.tif]

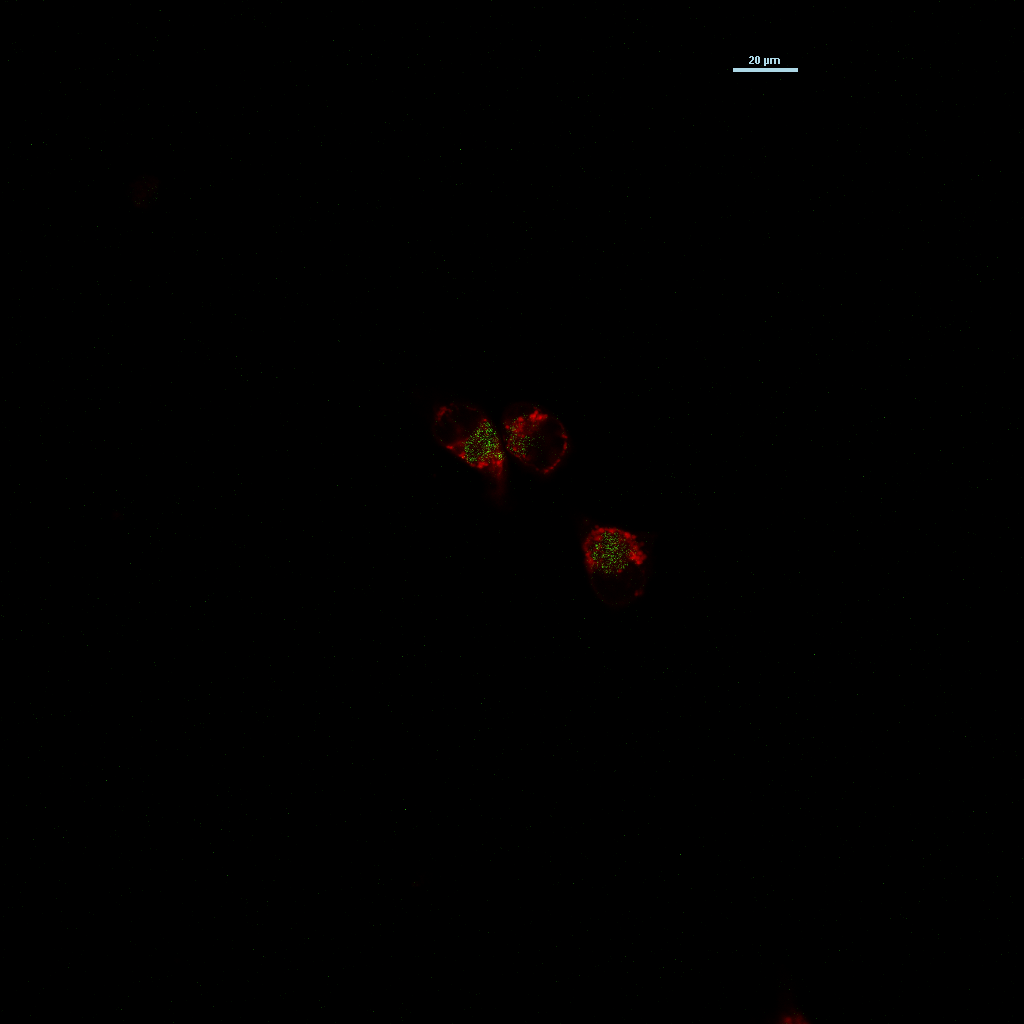

Supplement: Supplementary file 7 — Source data Fig. 5 [file 44321_2025_308_MOESM7_ESM.zip › Figure 5/5f/HCT116 Con 3/4_4_RGB.tif]

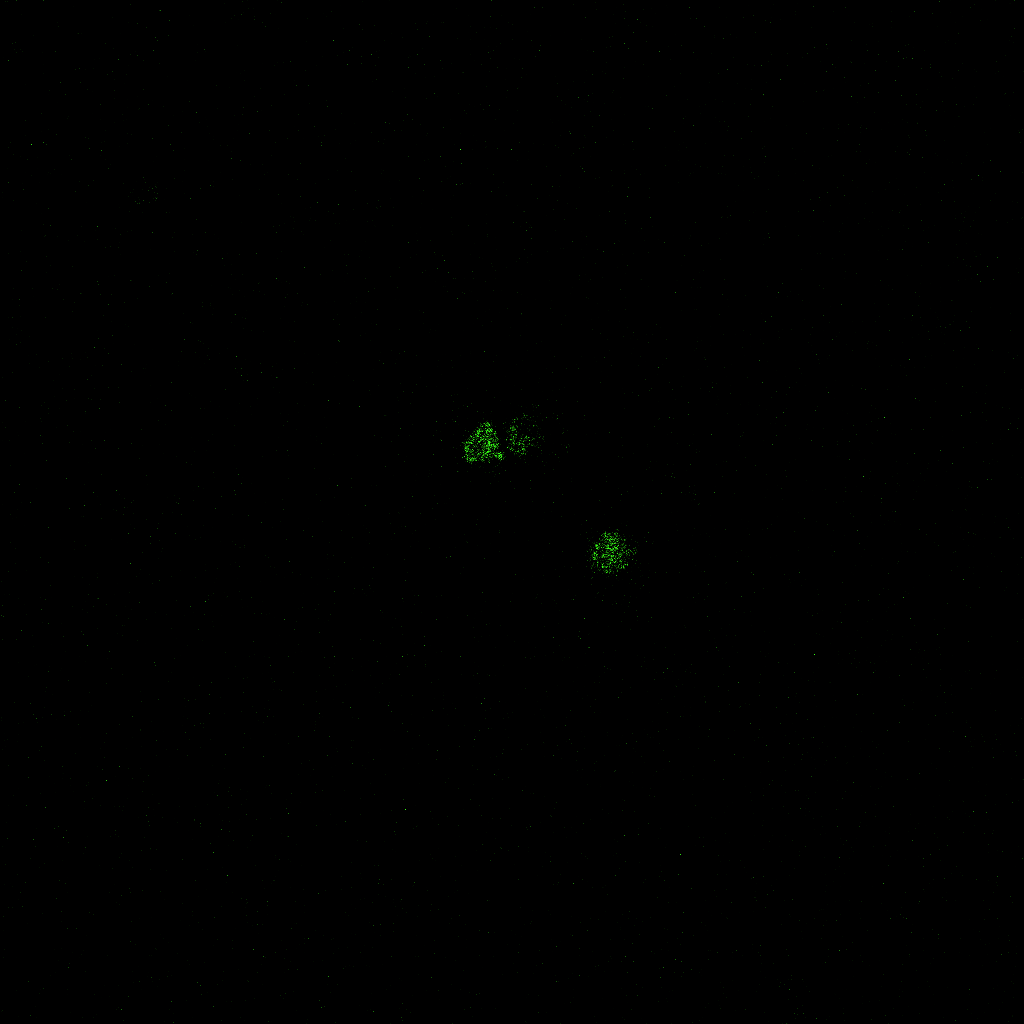

Supplement: Supplementary file 7 — Source data Fig. 5 [file 44321_2025_308_MOESM7_ESM.zip › Figure 5/5f/HCT116 Con 3/4_4_RGB_LysoTracker Green.tif]

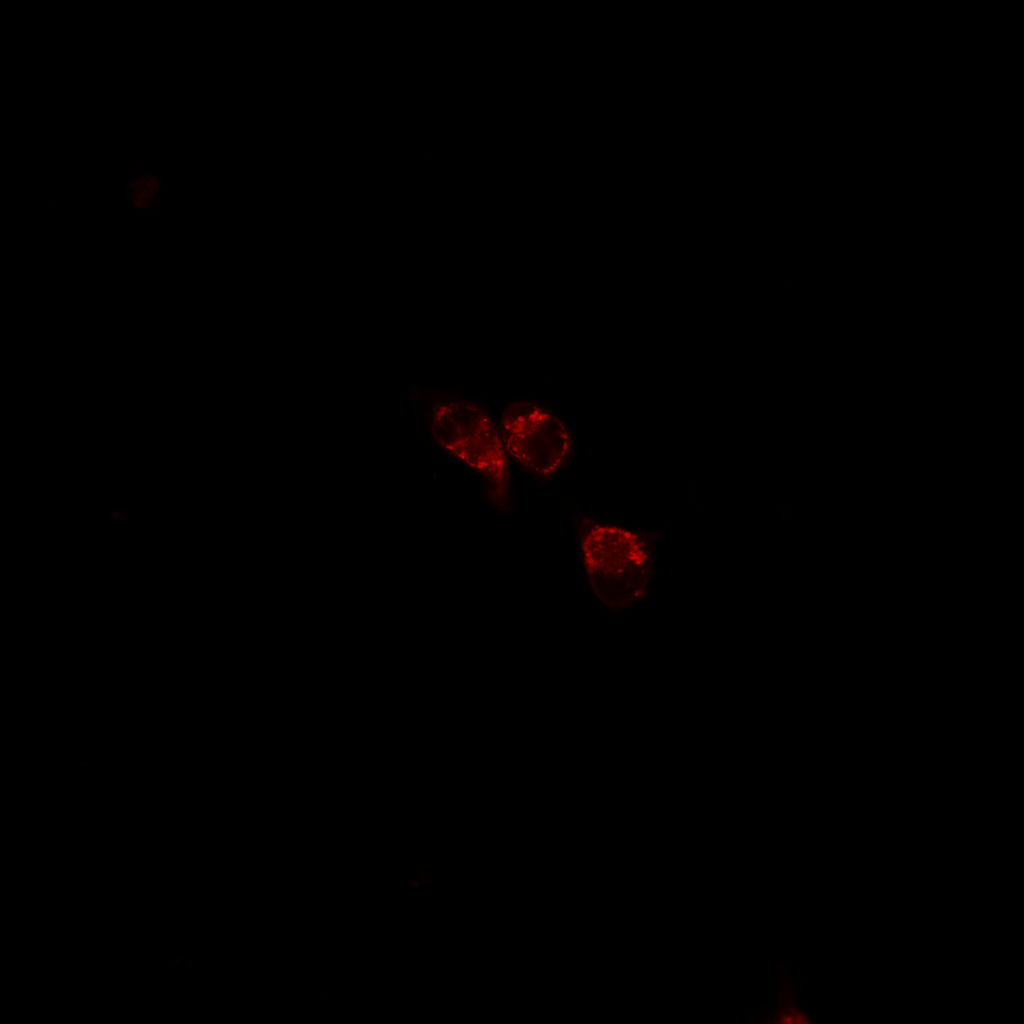

Supplement: Supplementary file 7 — Source data Fig. 5 [file 44321_2025_308_MOESM7_ESM.zip › Figure 5/5f/HCT116 Con 3/4_4_RGB_TRITC.tif]

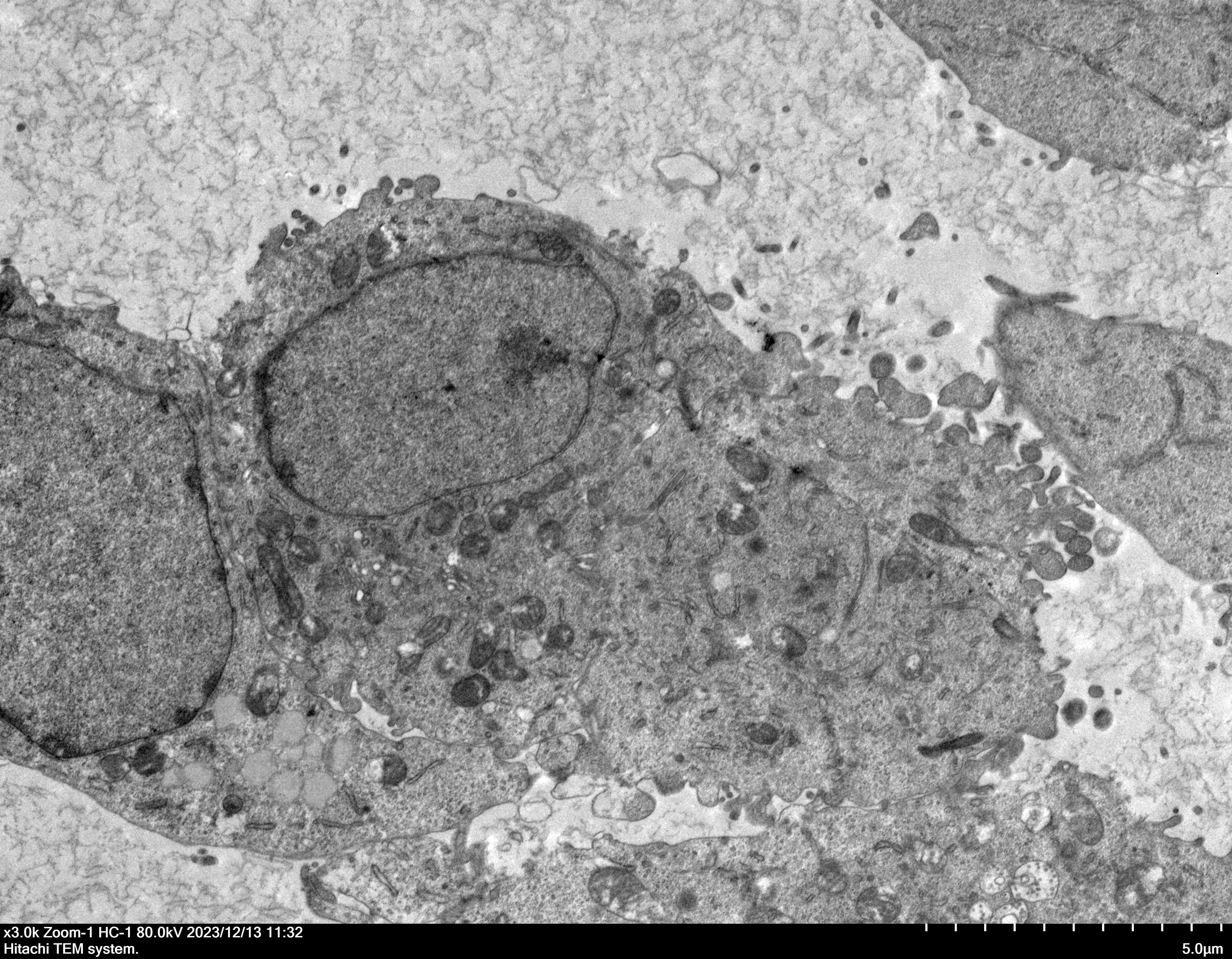

Supplement: Supplementary file 7 — Source data Fig. 5 [file 44321_2025_308_MOESM7_ESM.zip › Figure 5/5g, h/Con 5x (1).tif]

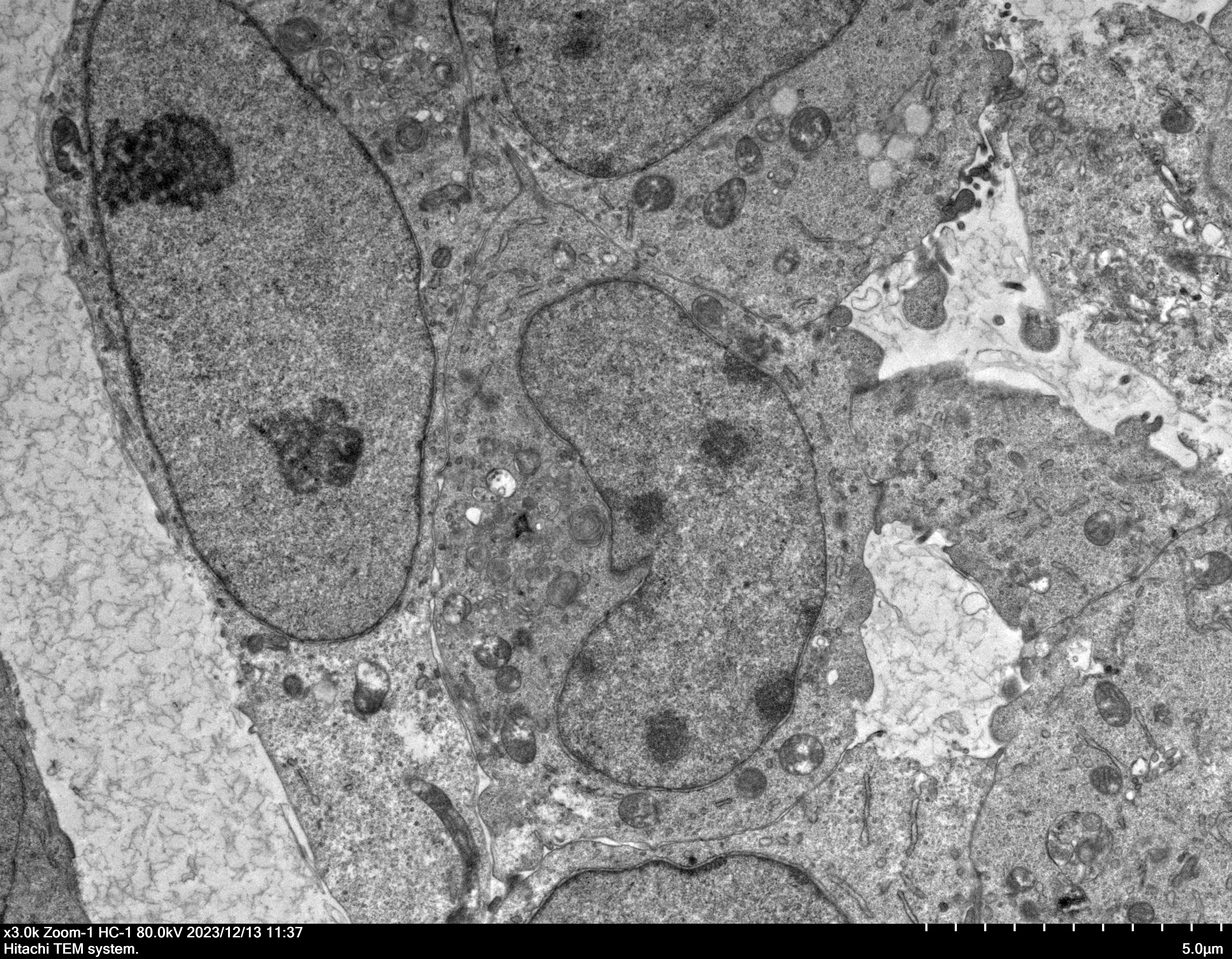

Supplement: Supplementary file 7 — Source data Fig. 5 [file 44321_2025_308_MOESM7_ESM.zip › Figure 5/5g, h/Con 5x (2).tif]

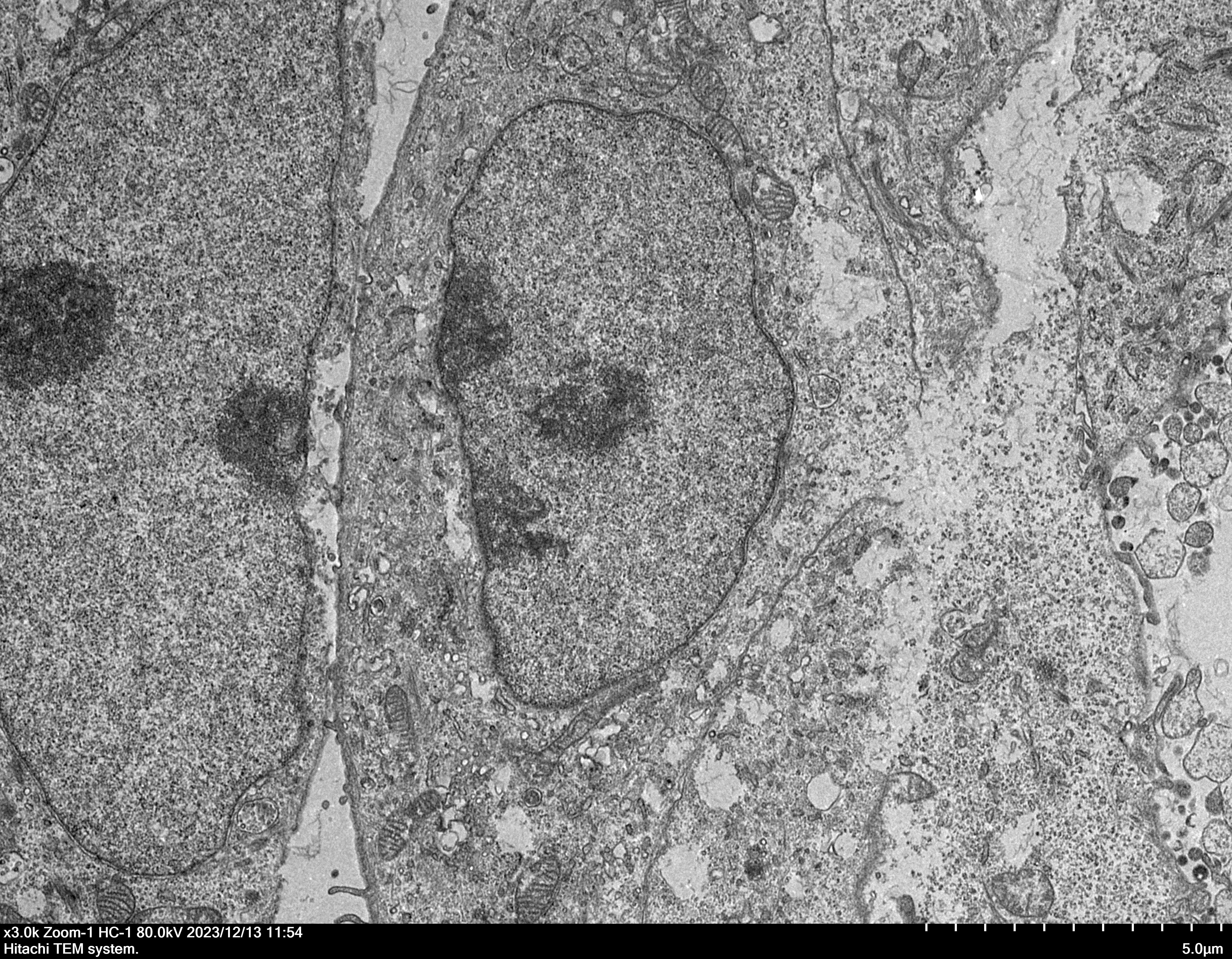

Supplement: Supplementary file 7 — Source data Fig. 5 [file 44321_2025_308_MOESM7_ESM.zip › Figure 5/5g, h/Con 5x (3).tif]

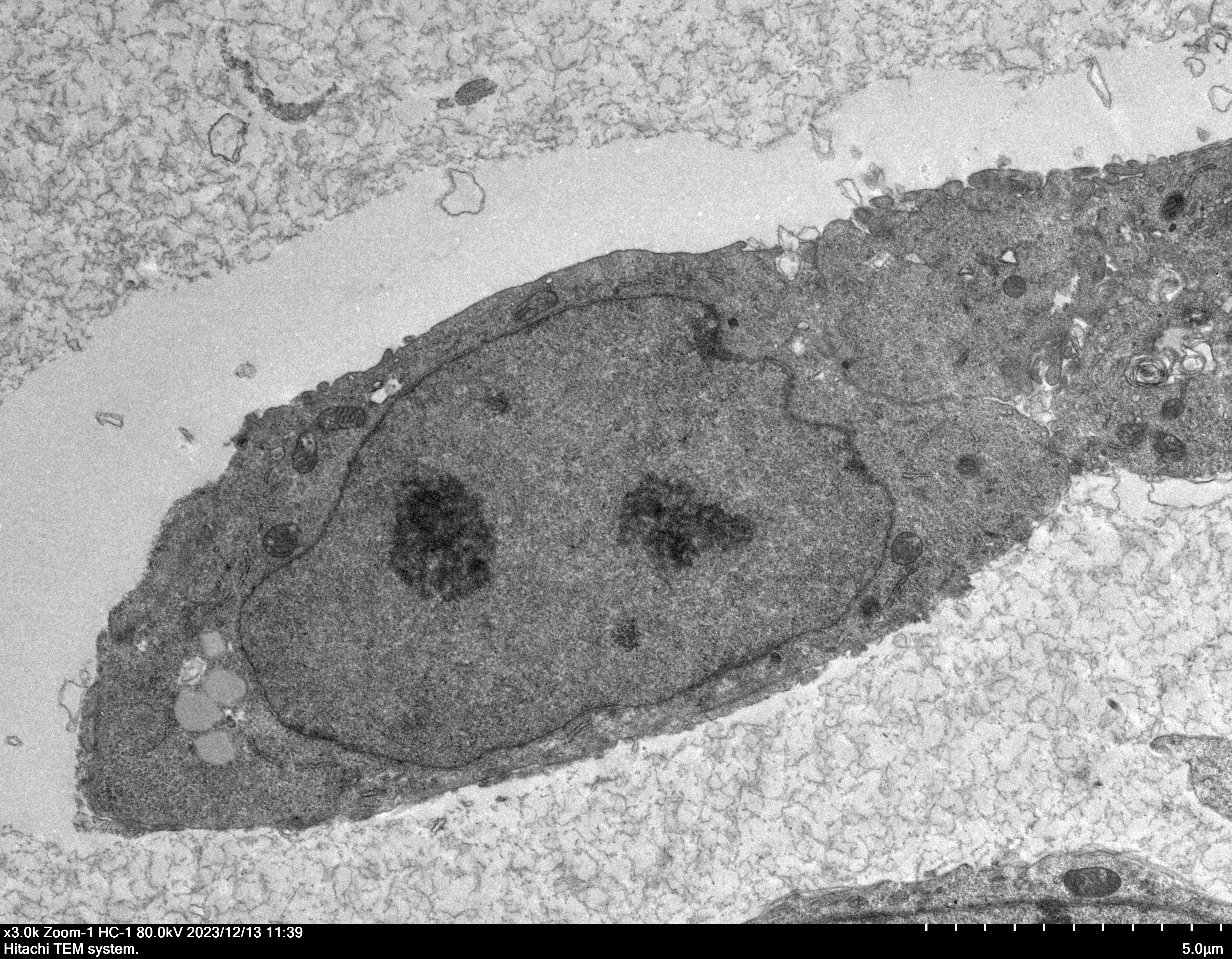

Supplement: Supplementary file 7 — Source data Fig. 5 [file 44321_2025_308_MOESM7_ESM.zip › Figure 5/5g, h/Con 5x (4).tif]

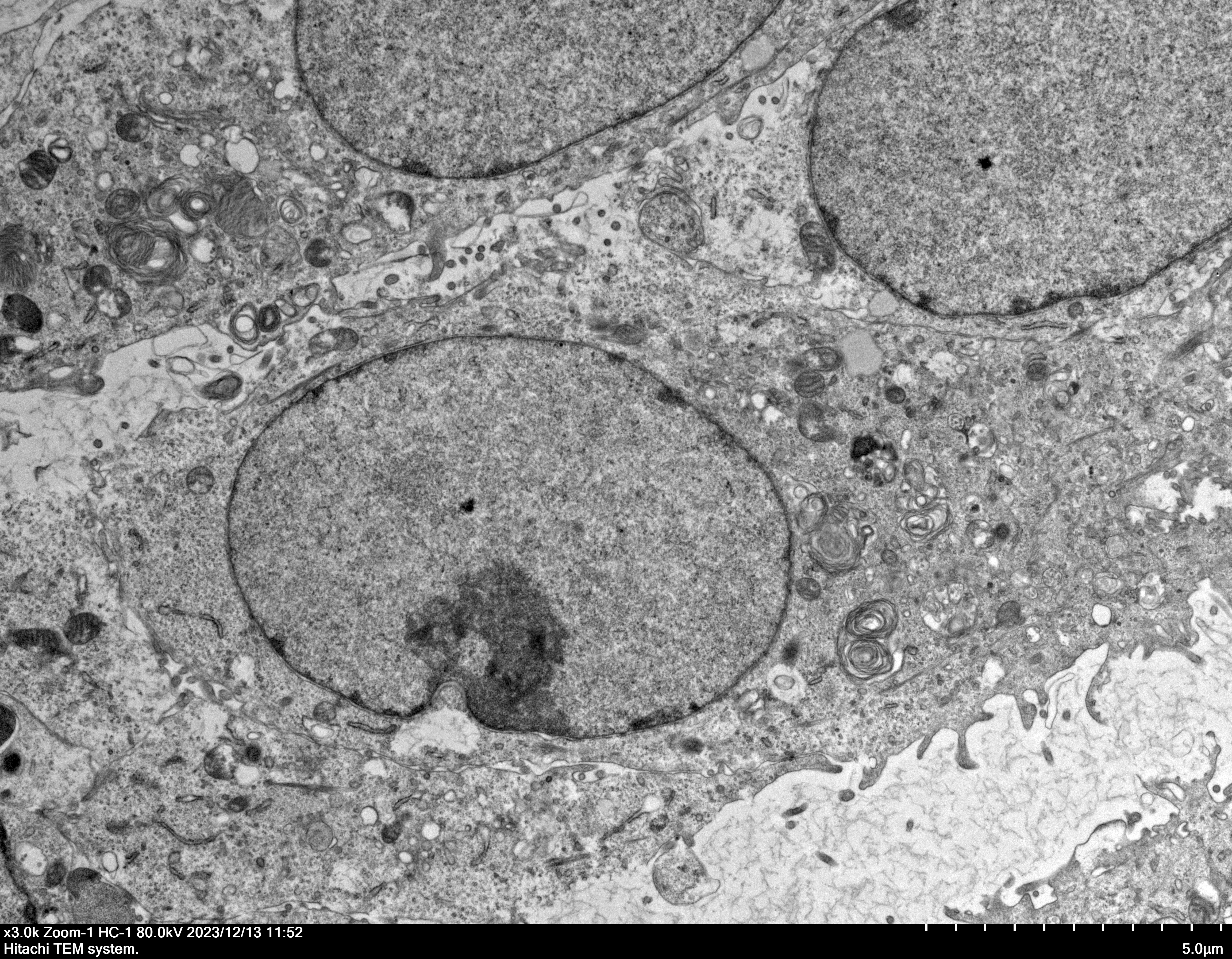

Supplement: Supplementary file 7 — Source data Fig. 5 [file 44321_2025_308_MOESM7_ESM.zip › Figure 5/5g, h/PP10 5x (1).tif]

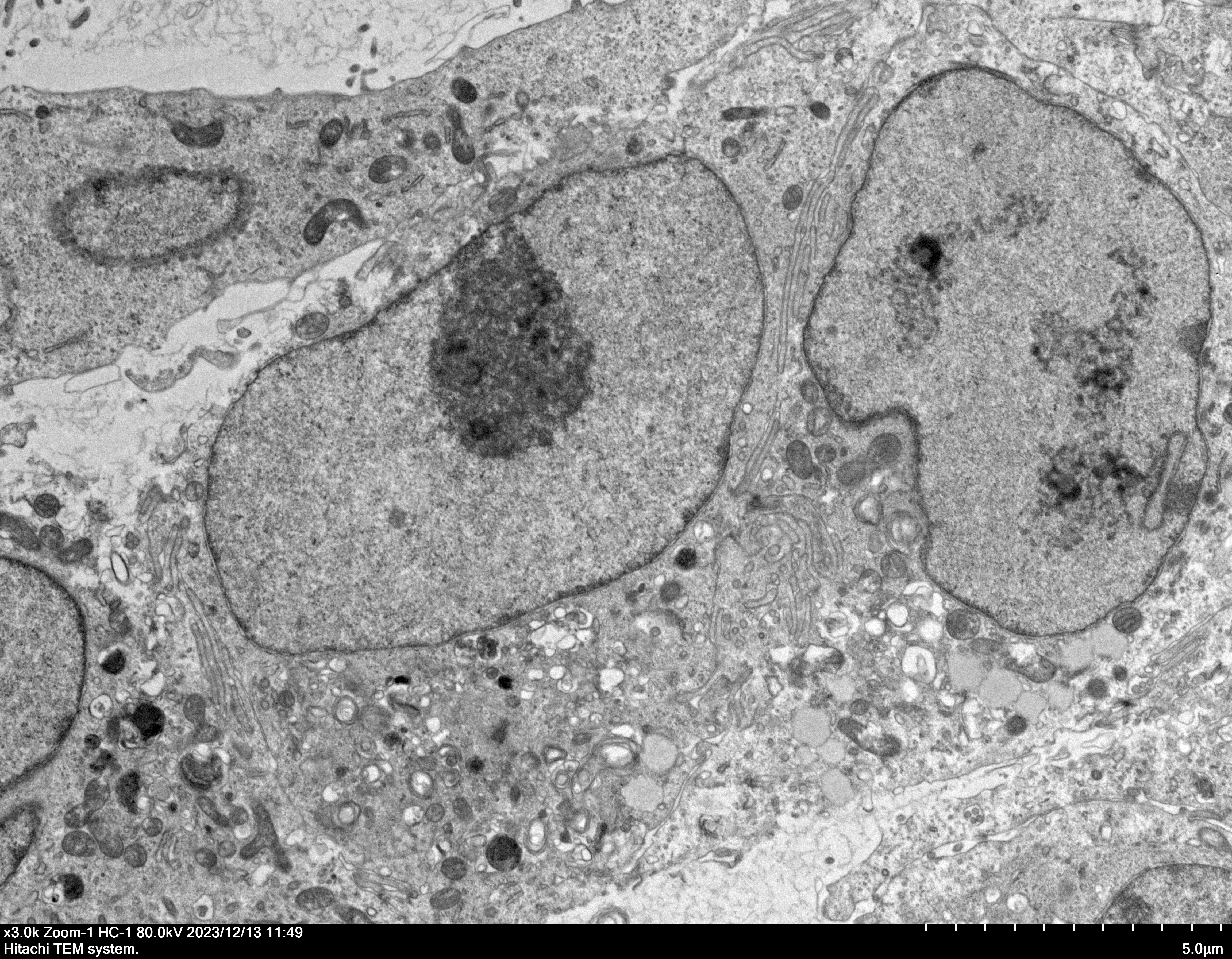

Supplement: Supplementary file 7 — Source data Fig. 5 [file 44321_2025_308_MOESM7_ESM.zip › Figure 5/5g, h/PP10 5x (2).tif]

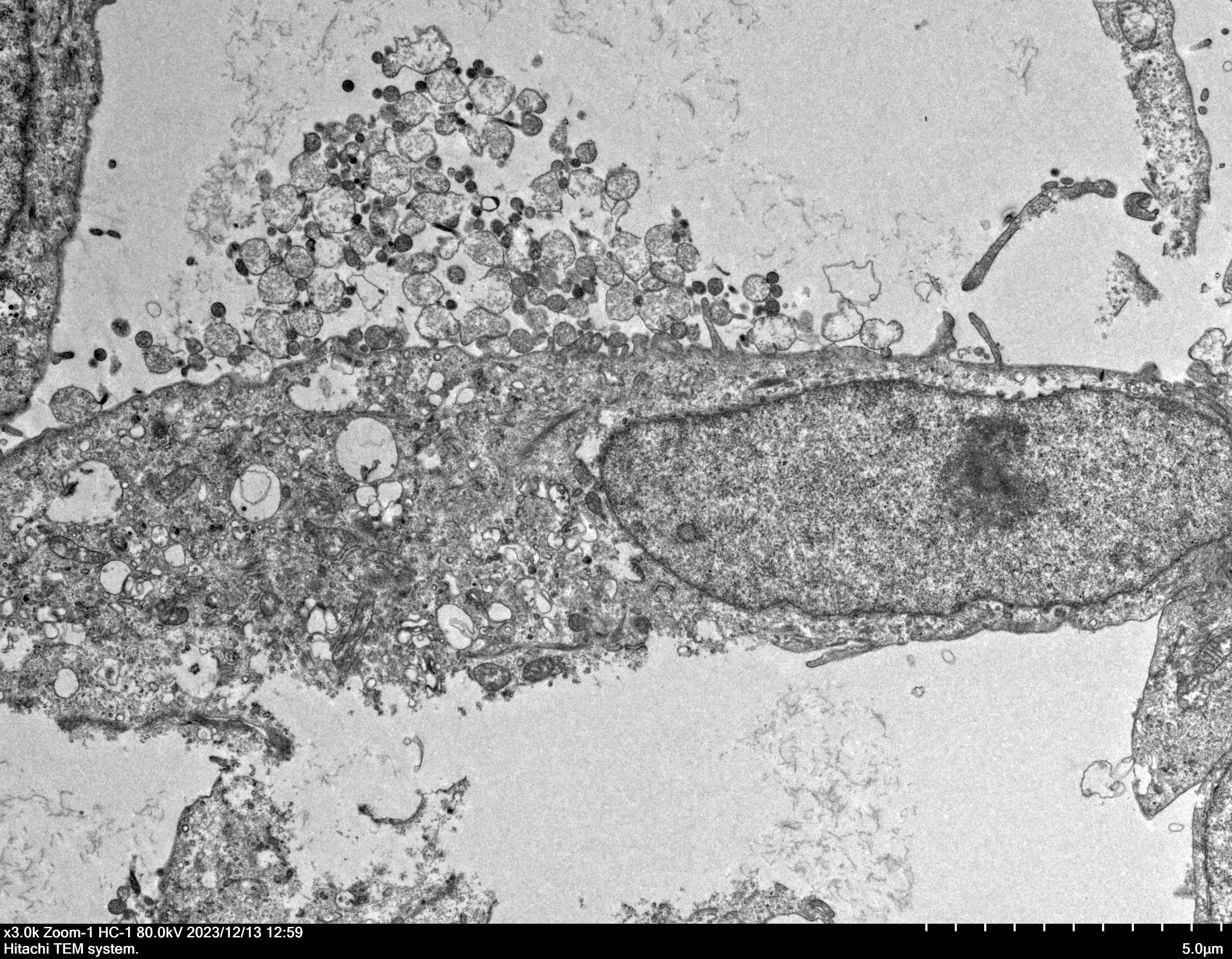

Supplement: Supplementary file 7 — Source data Fig. 5 [file 44321_2025_308_MOESM7_ESM.zip › Figure 5/5g, h/PP10 5x (3).tif]

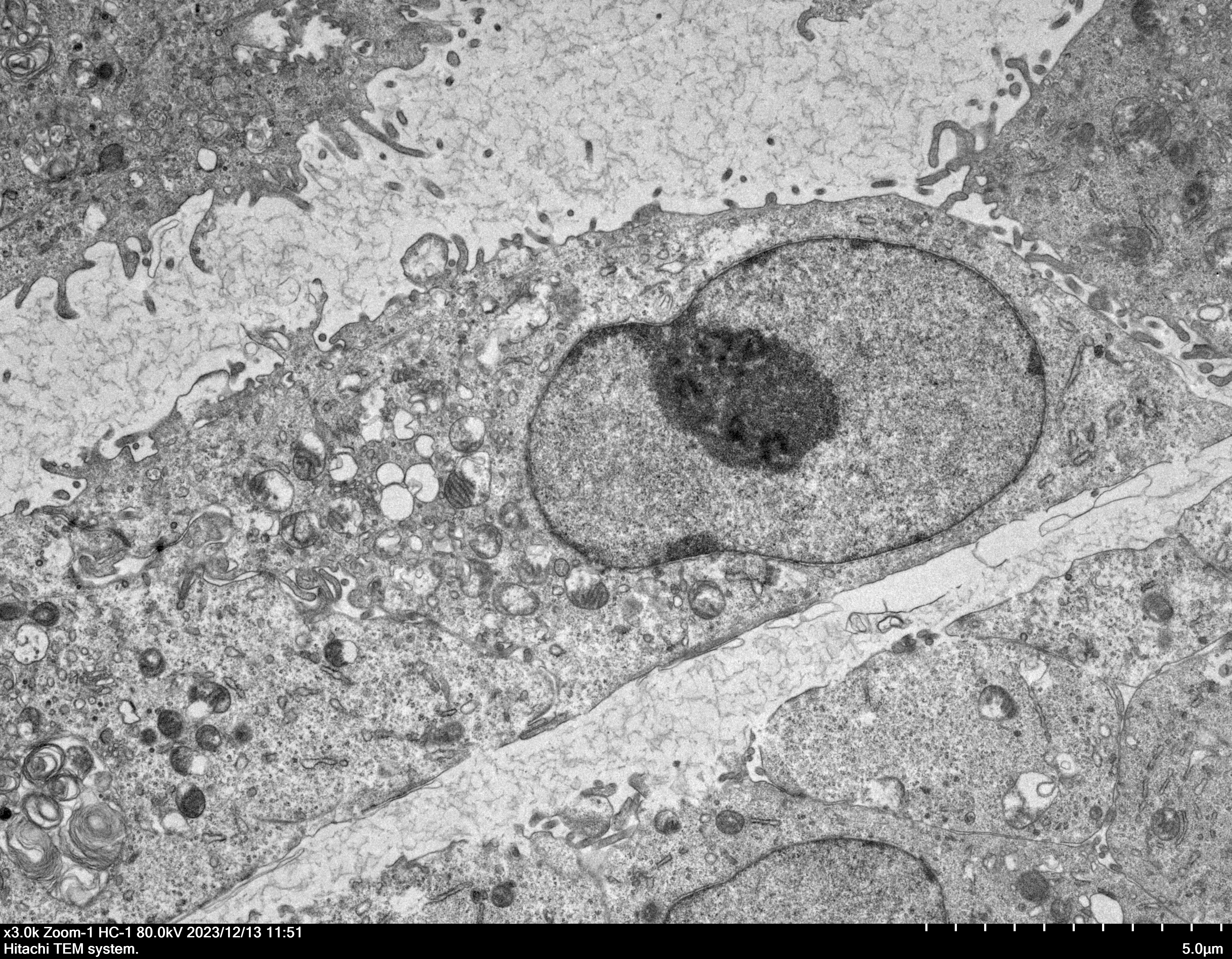

Supplement: Supplementary file 7 — Source data Fig. 5 [file 44321_2025_308_MOESM7_ESM.zip › Figure 5/5g, h/PP10 5x (4).tif]

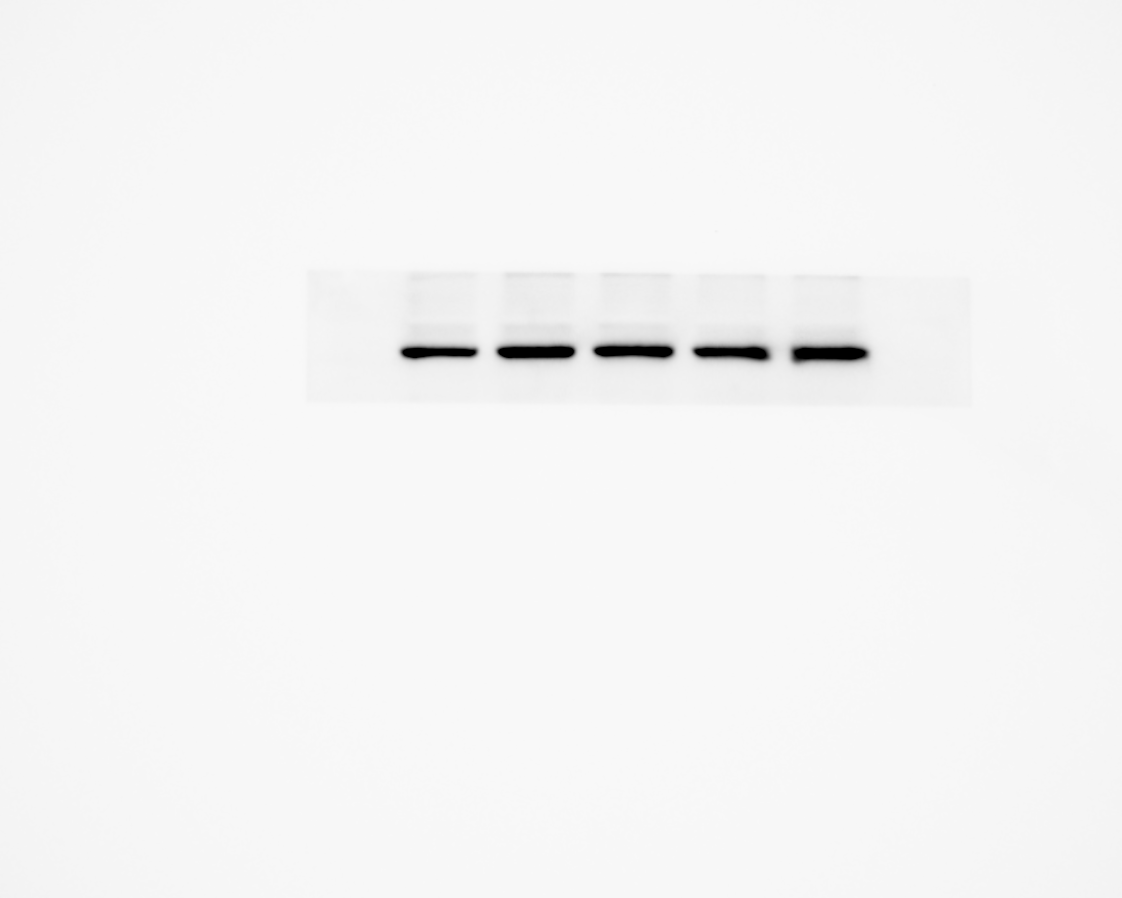

Supplement: Supplementary file 7 — Source data Fig. 5 [file 44321_2025_308_MOESM7_ESM.zip › Figure 5/5i/western actin 1.tif]

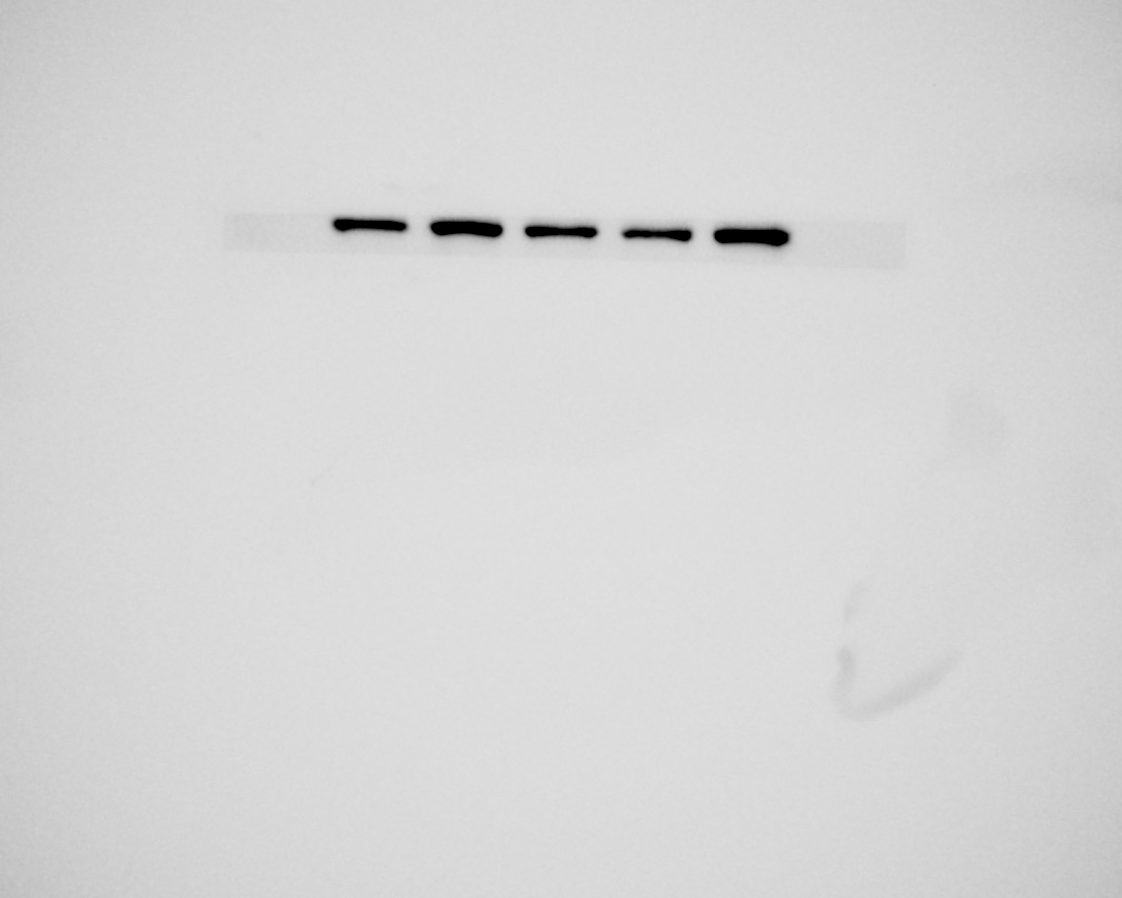

Supplement: Supplementary file 7 — Source data Fig. 5 [file 44321_2025_308_MOESM7_ESM.zip › Figure 5/5i/western actin 2.tif]

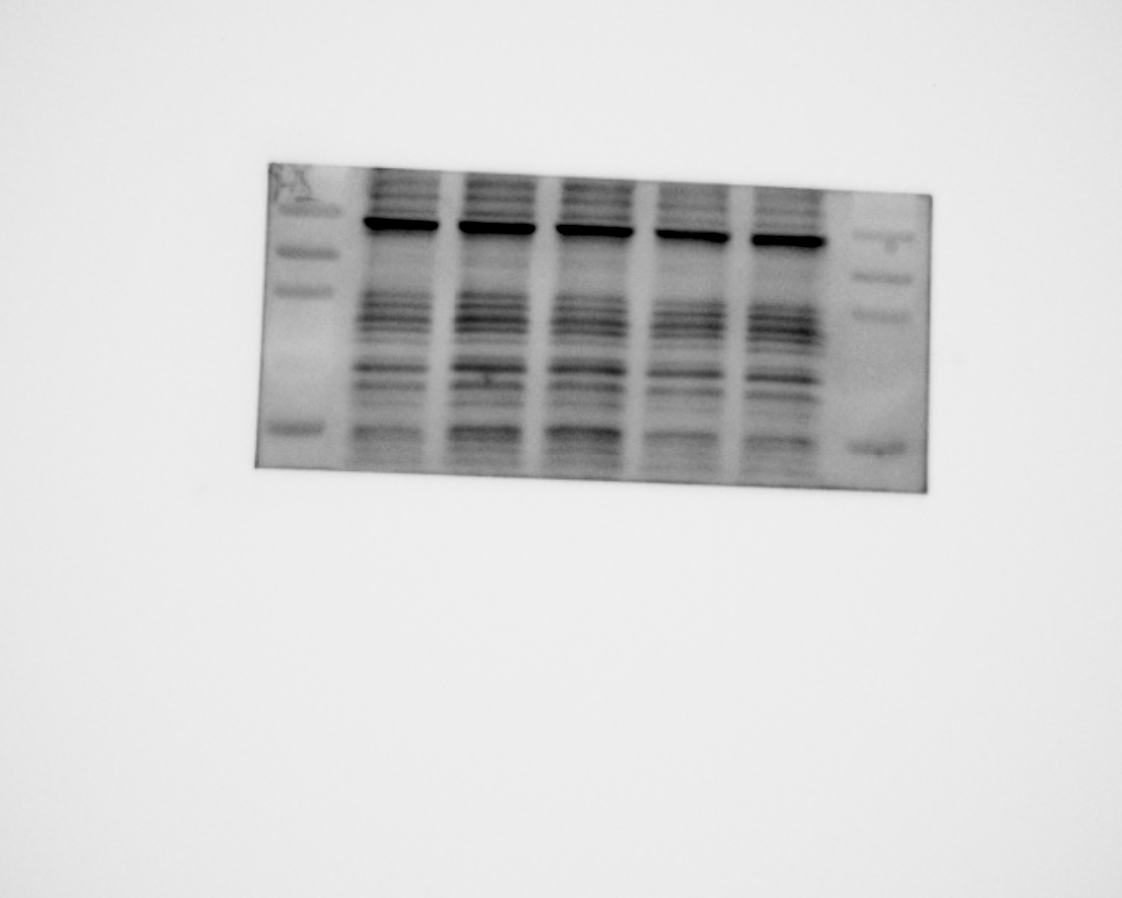

Supplement: Supplementary file 7 — Source data Fig. 5 [file 44321_2025_308_MOESM7_ESM.zip › Figure 5/5i/western caspase1 1.tif]

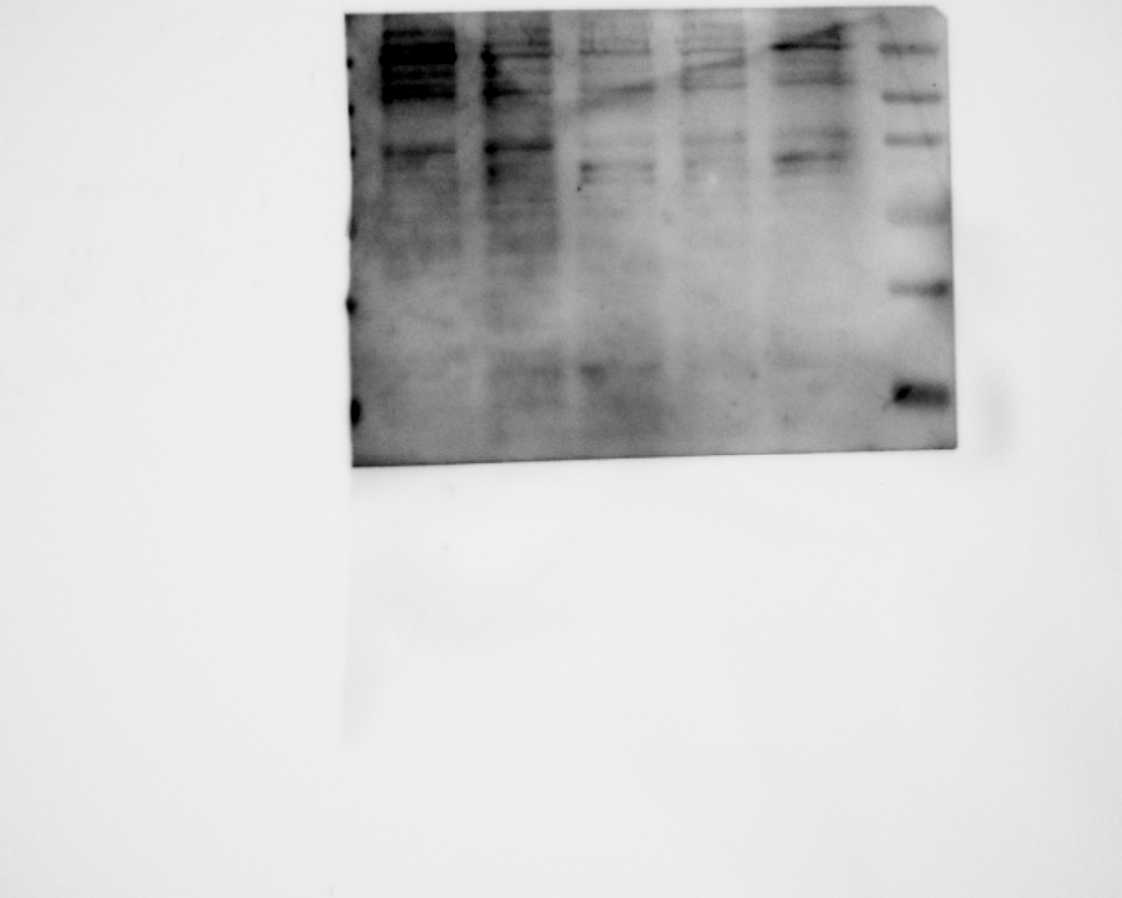

Supplement: Supplementary file 7 — Source data Fig. 5 [file 44321_2025_308_MOESM7_ESM.zip › Figure 5/5i/western caspase1 2.tif]

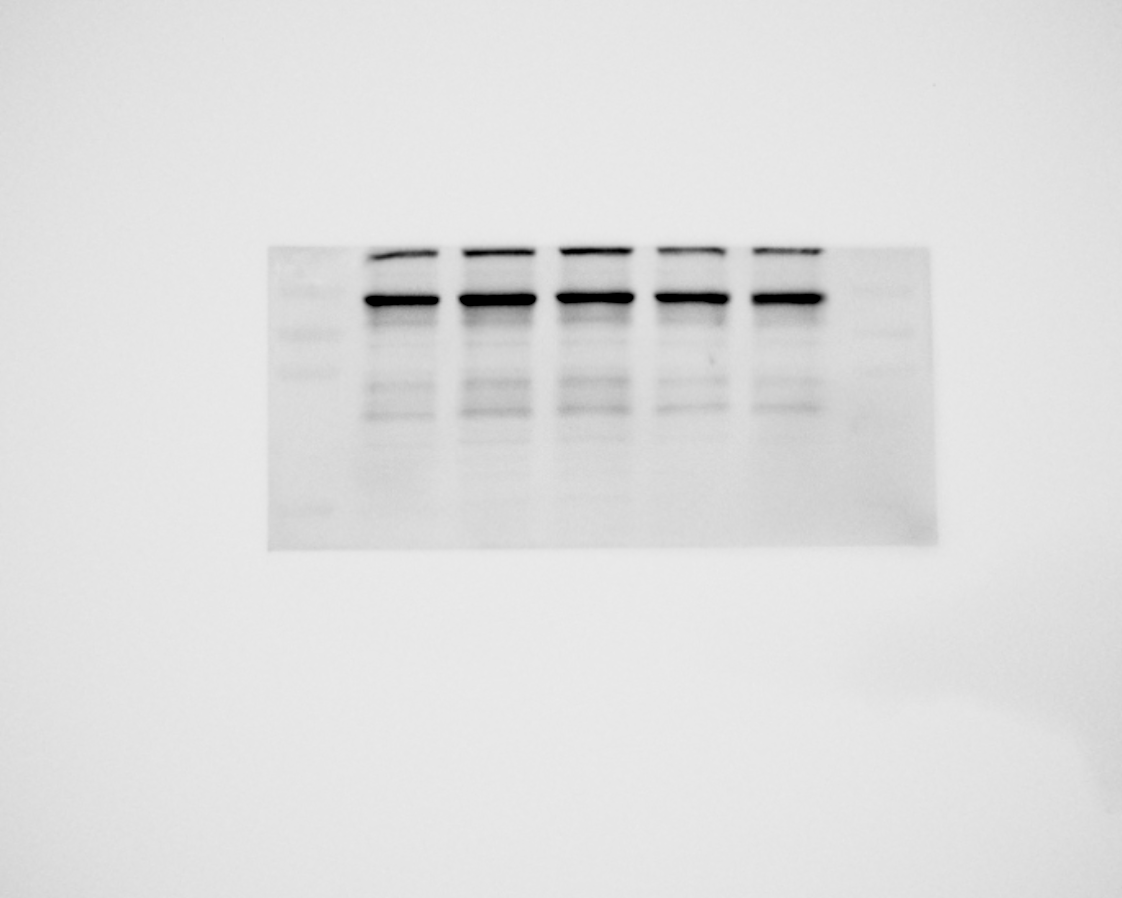

Supplement: Supplementary file 7 — Source data Fig. 5 [file 44321_2025_308_MOESM7_ESM.zip › Figure 5/5i/western GSDMD 1.tif]

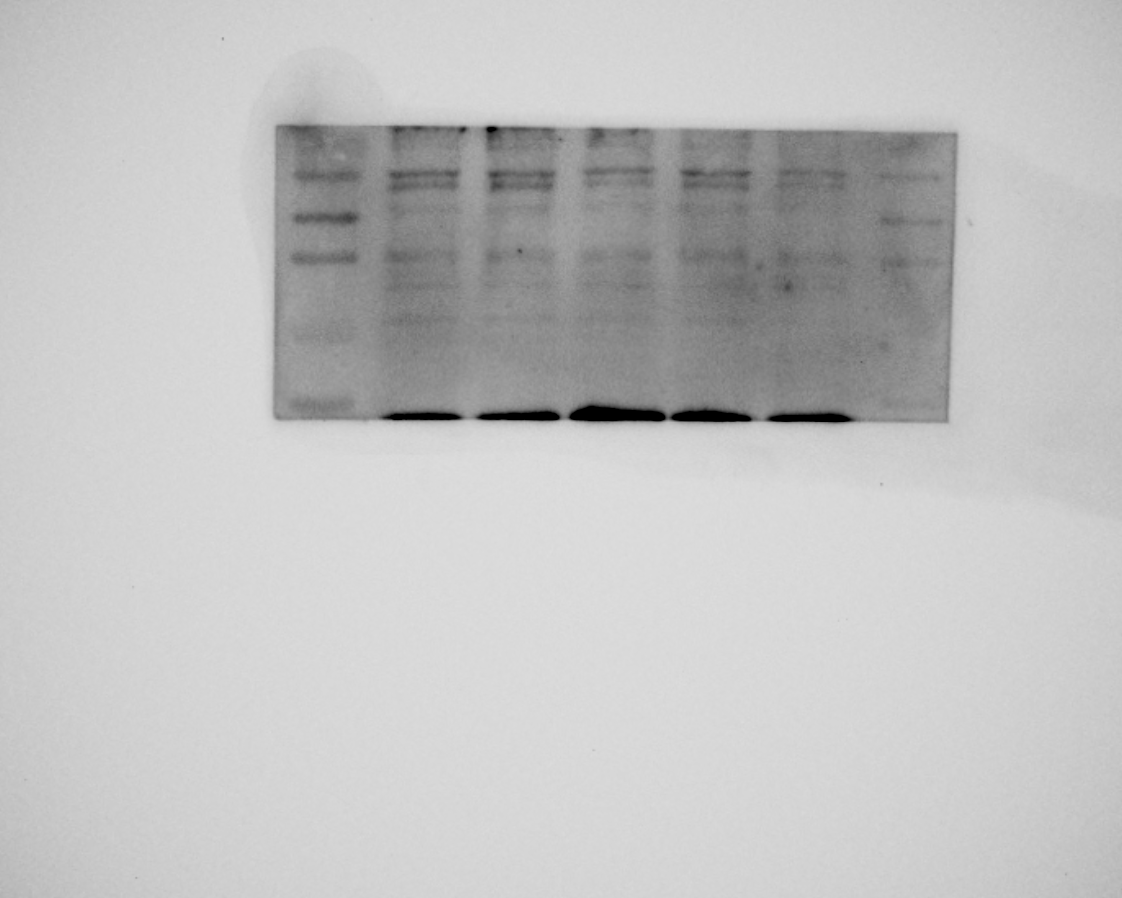

Supplement: Supplementary file 7 — Source data Fig. 5 [file 44321_2025_308_MOESM7_ESM.zip › Figure 5/5i/western GSDMD 2.tif]

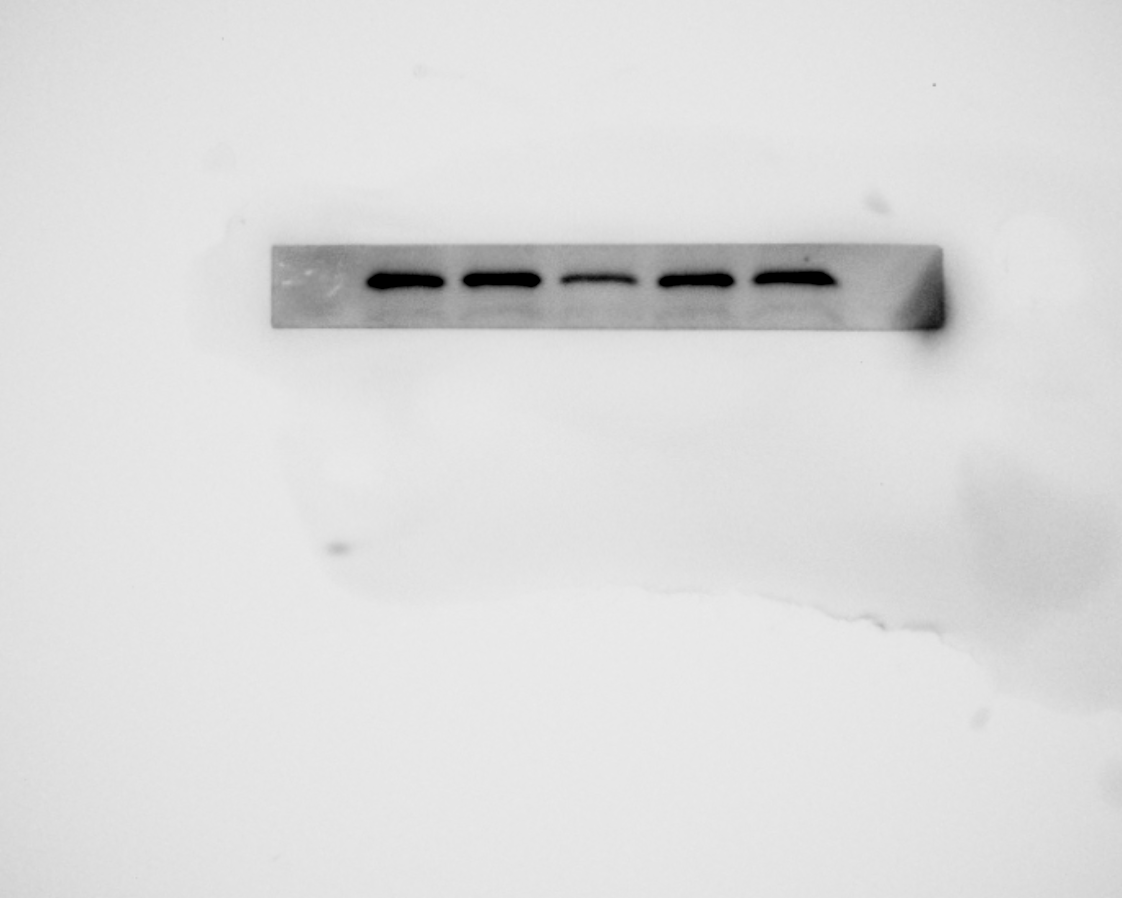

Supplement: Supplementary file 7 — Source data Fig. 5 [file 44321_2025_308_MOESM7_ESM.zip › Figure 5/5i/western il1b 1.tif]

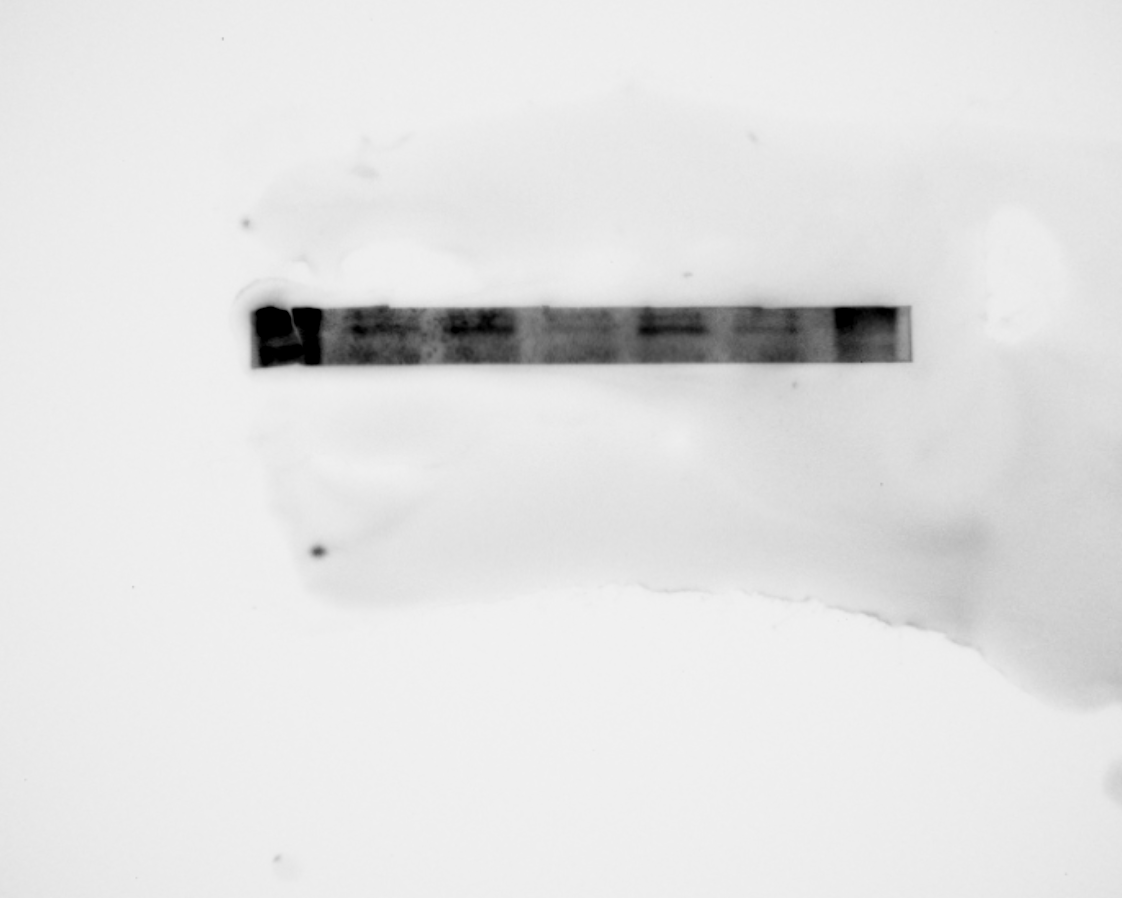

Supplement: Supplementary file 7 — Source data Fig. 5 [file 44321_2025_308_MOESM7_ESM.zip › Figure 5/5i/western il1b 2.tif]

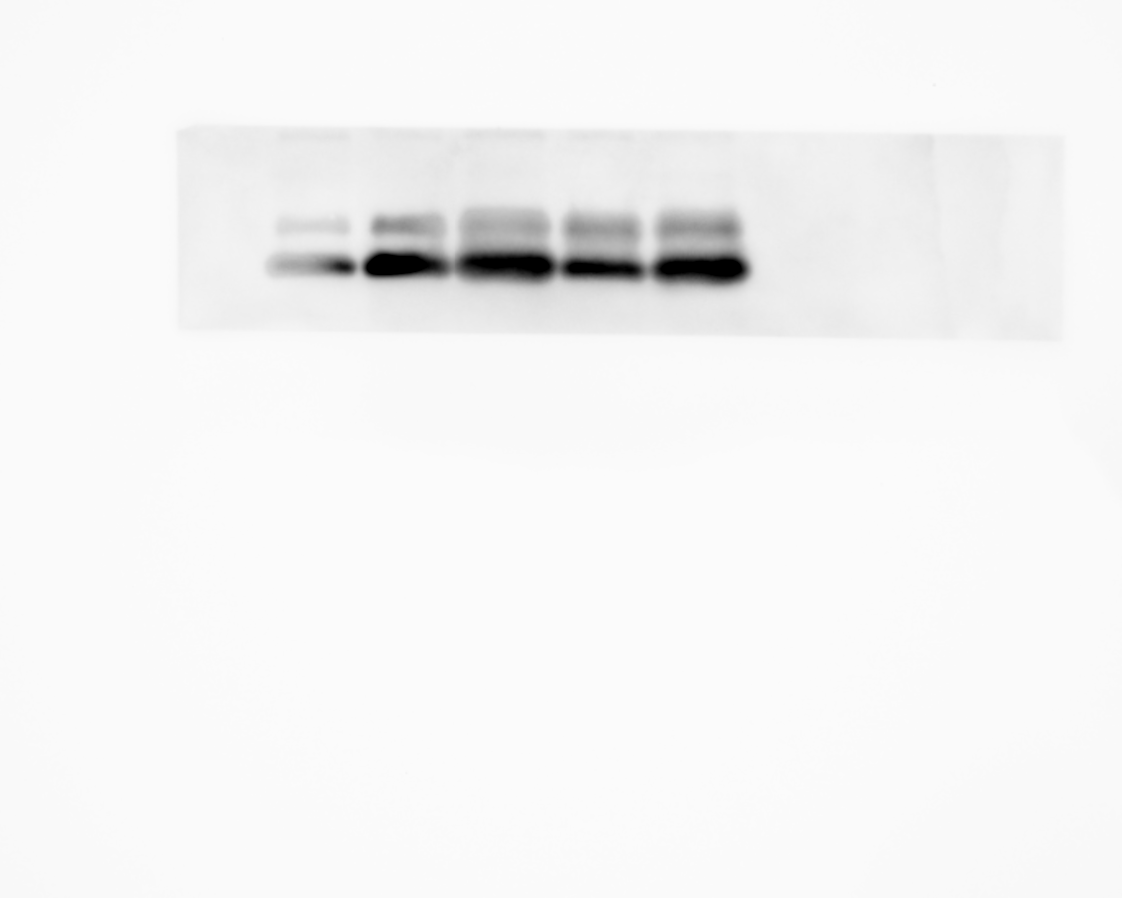

Supplement: Supplementary file 7 — Source data Fig. 5 [file 44321_2025_308_MOESM7_ESM.zip › Figure 5/5i/western il1b-m 1.tif]

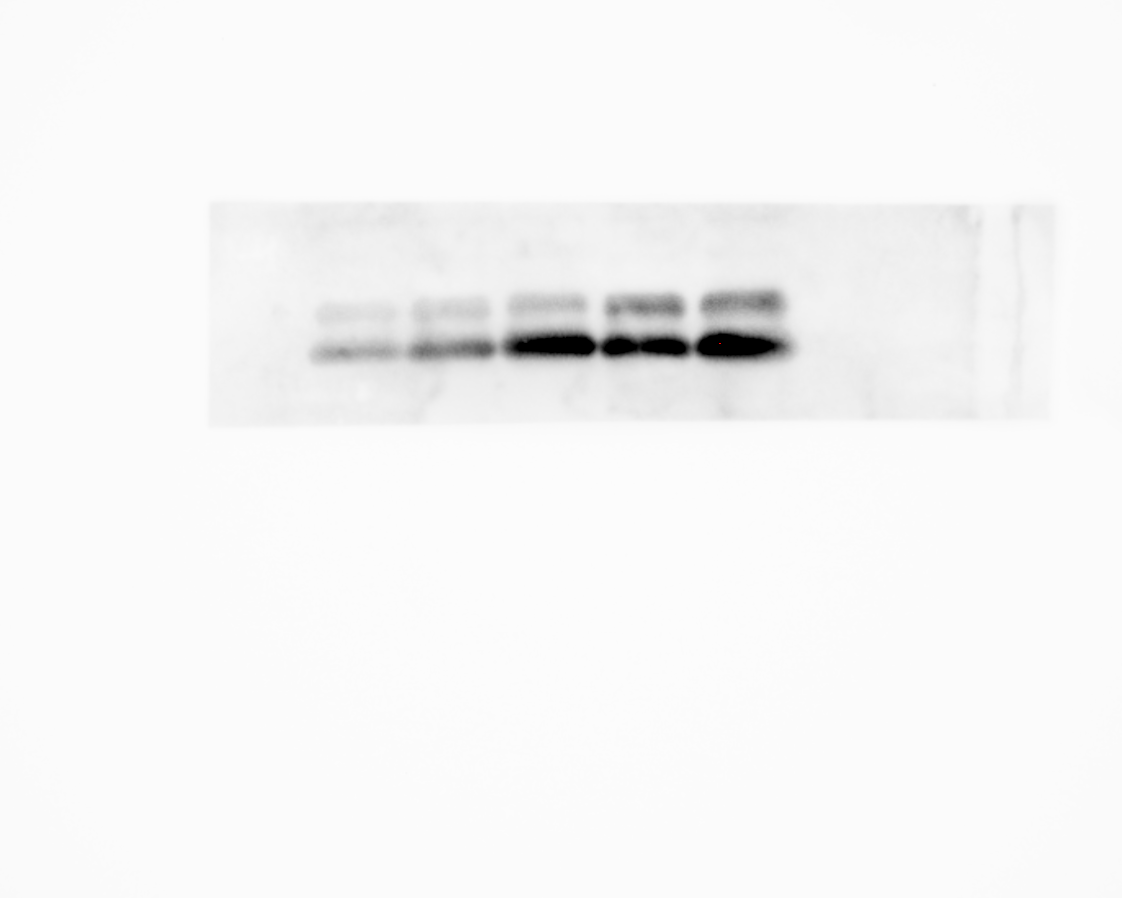

Supplement: Supplementary file 7 — Source data Fig. 5 [file 44321_2025_308_MOESM7_ESM.zip › Figure 5/5i/western il1b-m 2.tif]

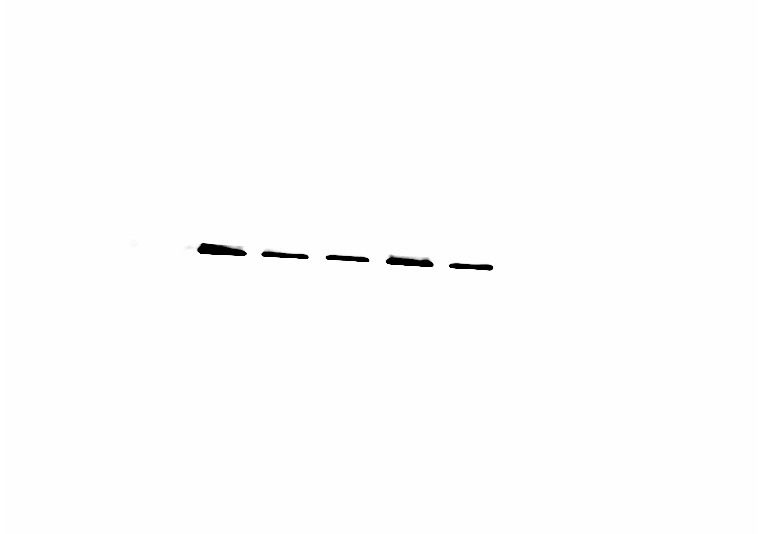

Supplement: Supplementary file 8 — Source data Fig. 6 [file 44321_2025_308_MOESM8_ESM.zip › Figure 6/6c/western AKT.tif]

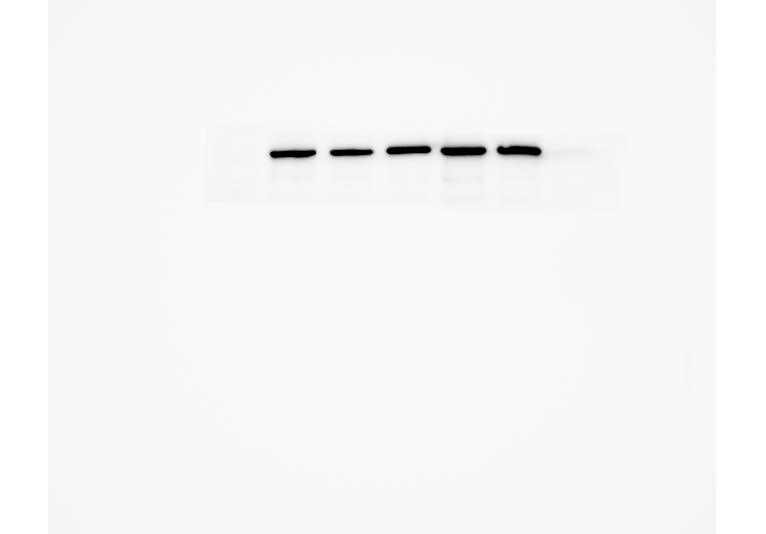

Supplement: Supplementary file 8 — Source data Fig. 6 [file 44321_2025_308_MOESM8_ESM.zip › Figure 6/6c/western bactin.tif]

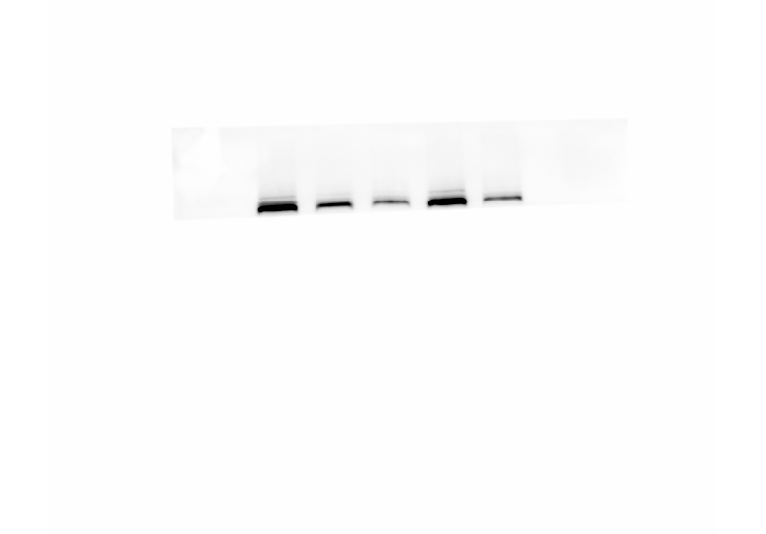

Supplement: Supplementary file 8 — Source data Fig. 6 [file 44321_2025_308_MOESM8_ESM.zip › Figure 6/6c/western bcatnenin.tif]

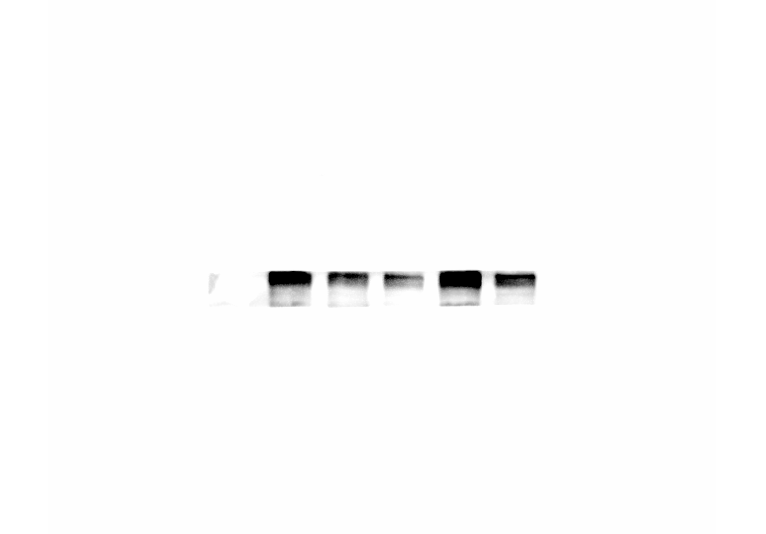

Supplement: Supplementary file 8 — Source data Fig. 6 [file 44321_2025_308_MOESM8_ESM.zip › Figure 6/6c/western Dvl2.tif]

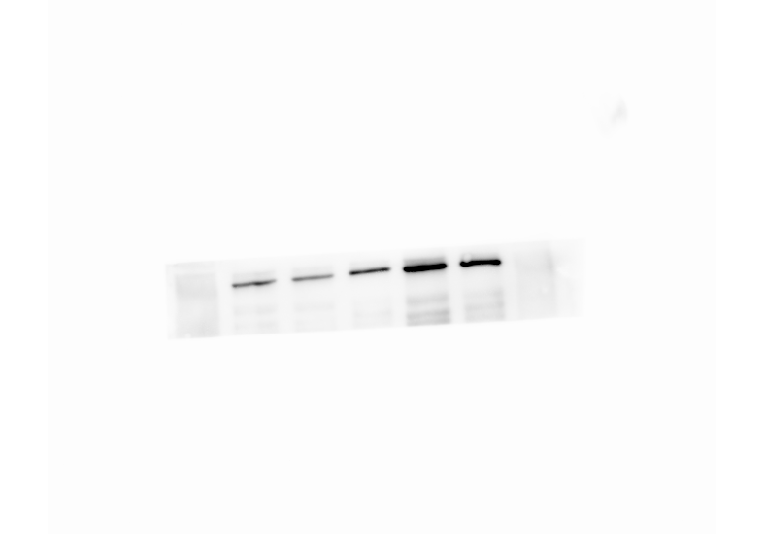

Supplement: Supplementary file 8 — Source data Fig. 6 [file 44321_2025_308_MOESM8_ESM.zip › Figure 6/6c/western gsk3b.tif]

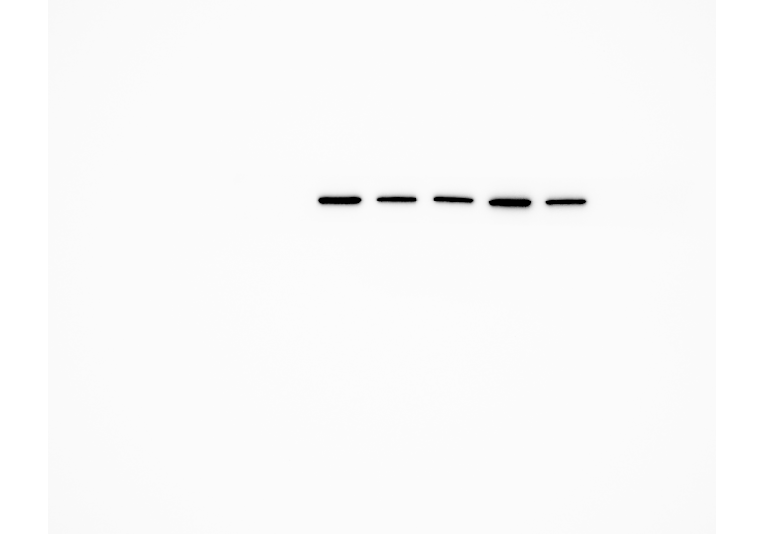

Supplement: Supplementary file 8 — Source data Fig. 6 [file 44321_2025_308_MOESM8_ESM.zip › Figure 6/6c/western Laminb1.tif]

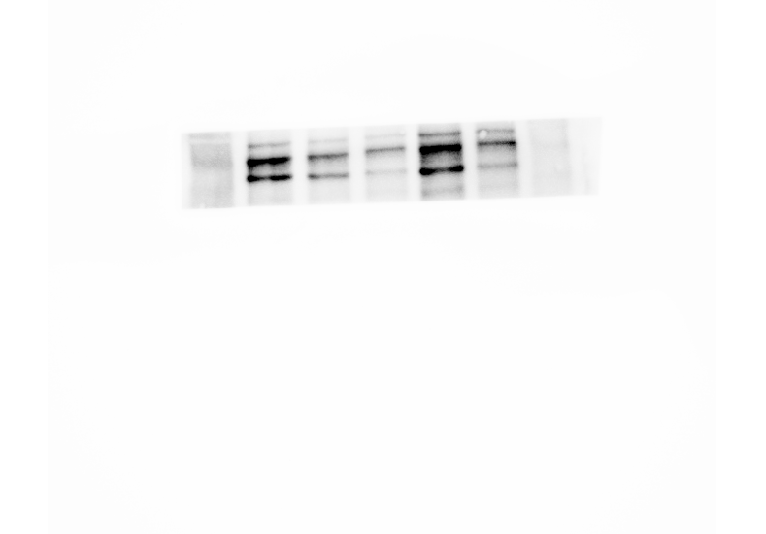

Supplement: Supplementary file 8 — Source data Fig. 6 [file 44321_2025_308_MOESM8_ESM.zip › Figure 6/6c/western LEF1.tif]

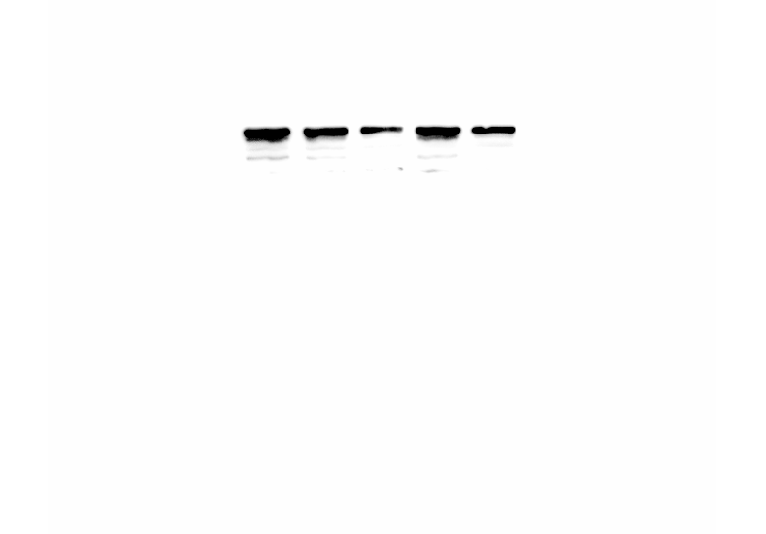

Supplement: Supplementary file 8 — Source data Fig. 6 [file 44321_2025_308_MOESM8_ESM.zip › Figure 6/6c/western Naked1.tif]

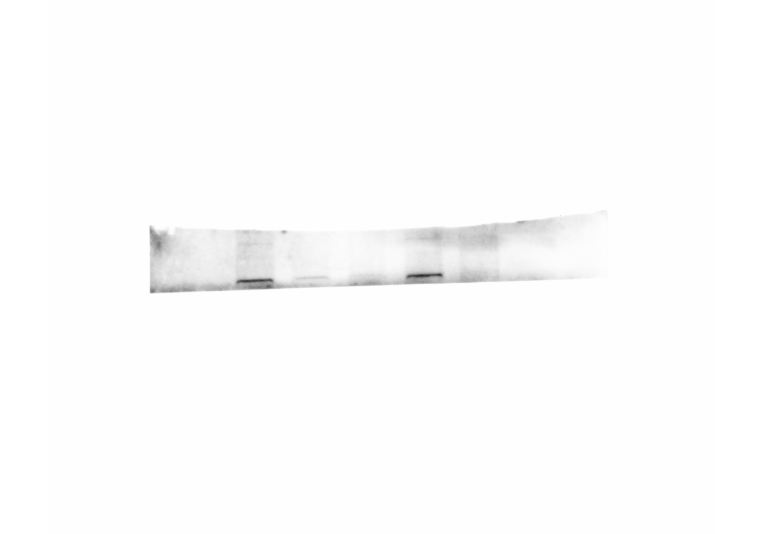

Supplement: Supplementary file 8 — Source data Fig. 6 [file 44321_2025_308_MOESM8_ESM.zip › Figure 6/6c/western p bcat.tif]

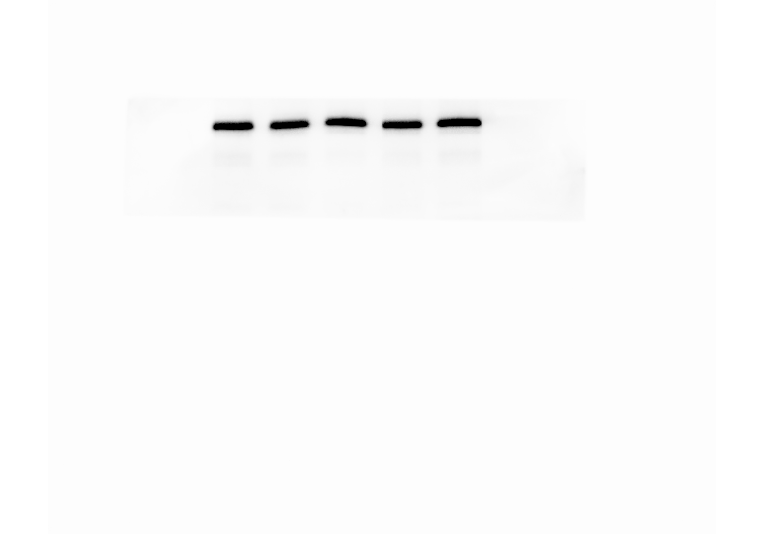

Supplement: Supplementary file 8 — Source data Fig. 6 [file 44321_2025_308_MOESM8_ESM.zip › Figure 6/6c/western p gsk3b.tif]

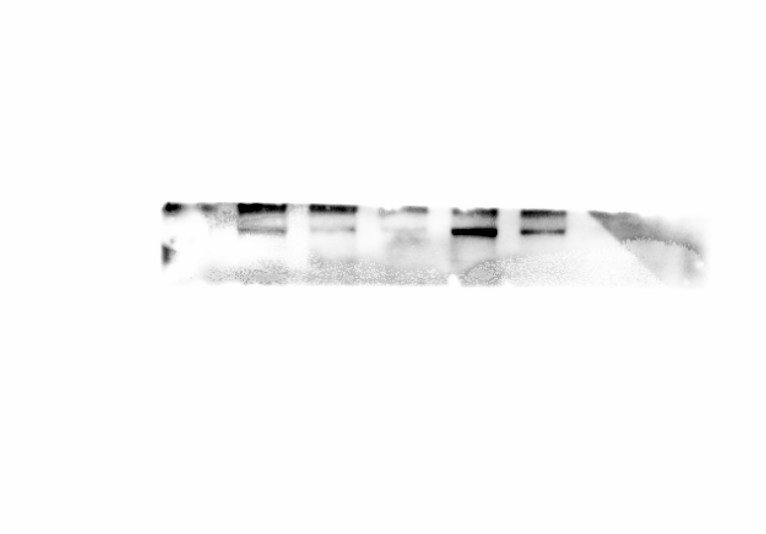

Supplement: Supplementary file 8 — Source data Fig. 6 [file 44321_2025_308_MOESM8_ESM.zip › Figure 6/6c/western pAKT.tif]

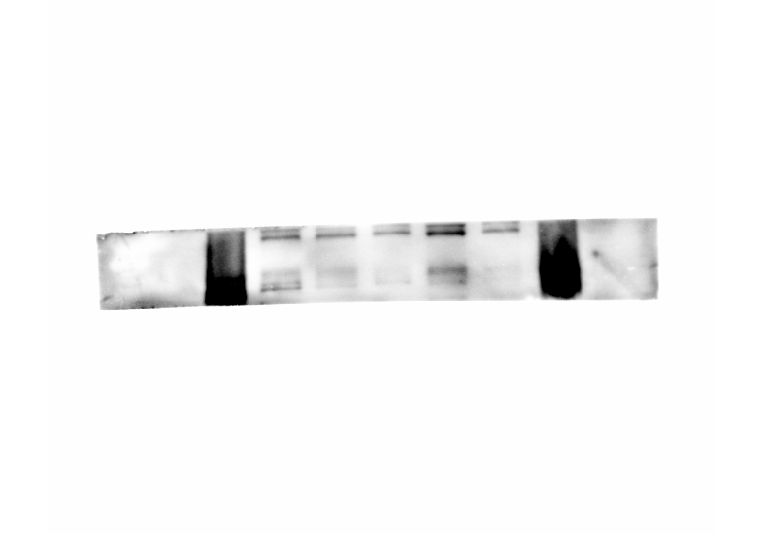

Supplement: Supplementary file 8 — Source data Fig. 6 [file 44321_2025_308_MOESM8_ESM.zip › Figure 6/6c/western pGSK3b.tif]

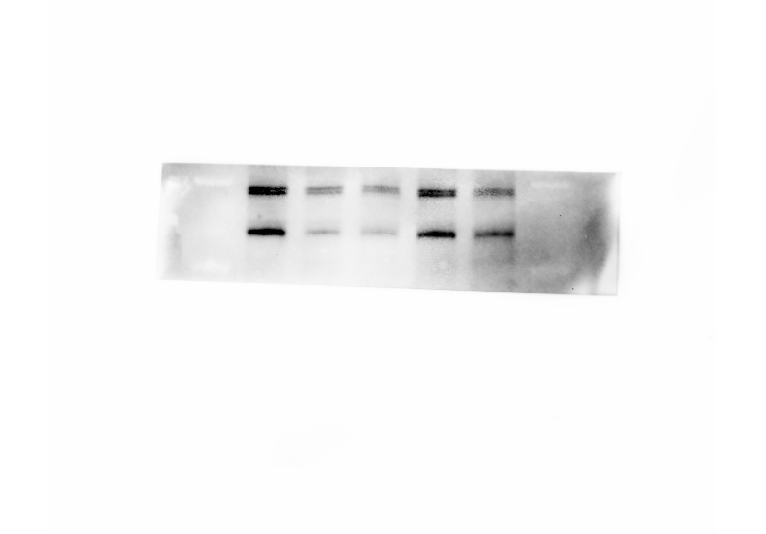

Supplement: Supplementary file 8 — Source data Fig. 6 [file 44321_2025_308_MOESM8_ESM.zip › Figure 6/6c/western TCF17.tif]

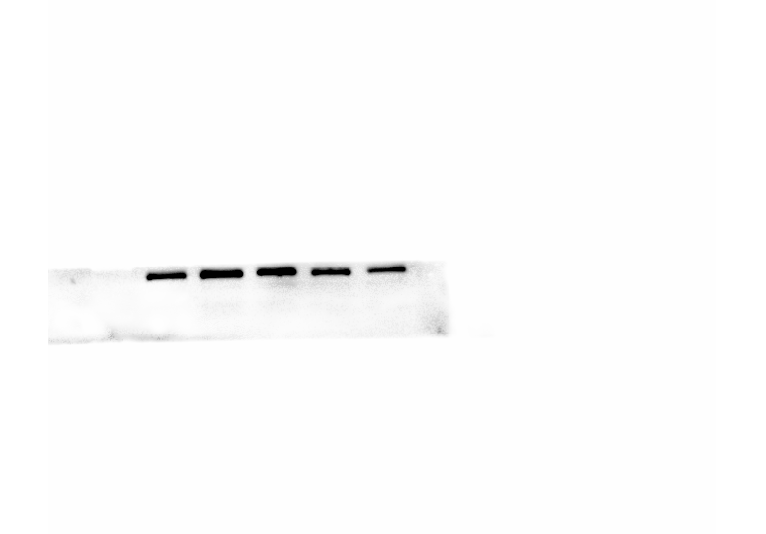

Supplement: Supplementary file 8 — Source data Fig. 6 [file 44321_2025_308_MOESM8_ESM.zip › Figure 6/6c/western-GSK3b.tif]

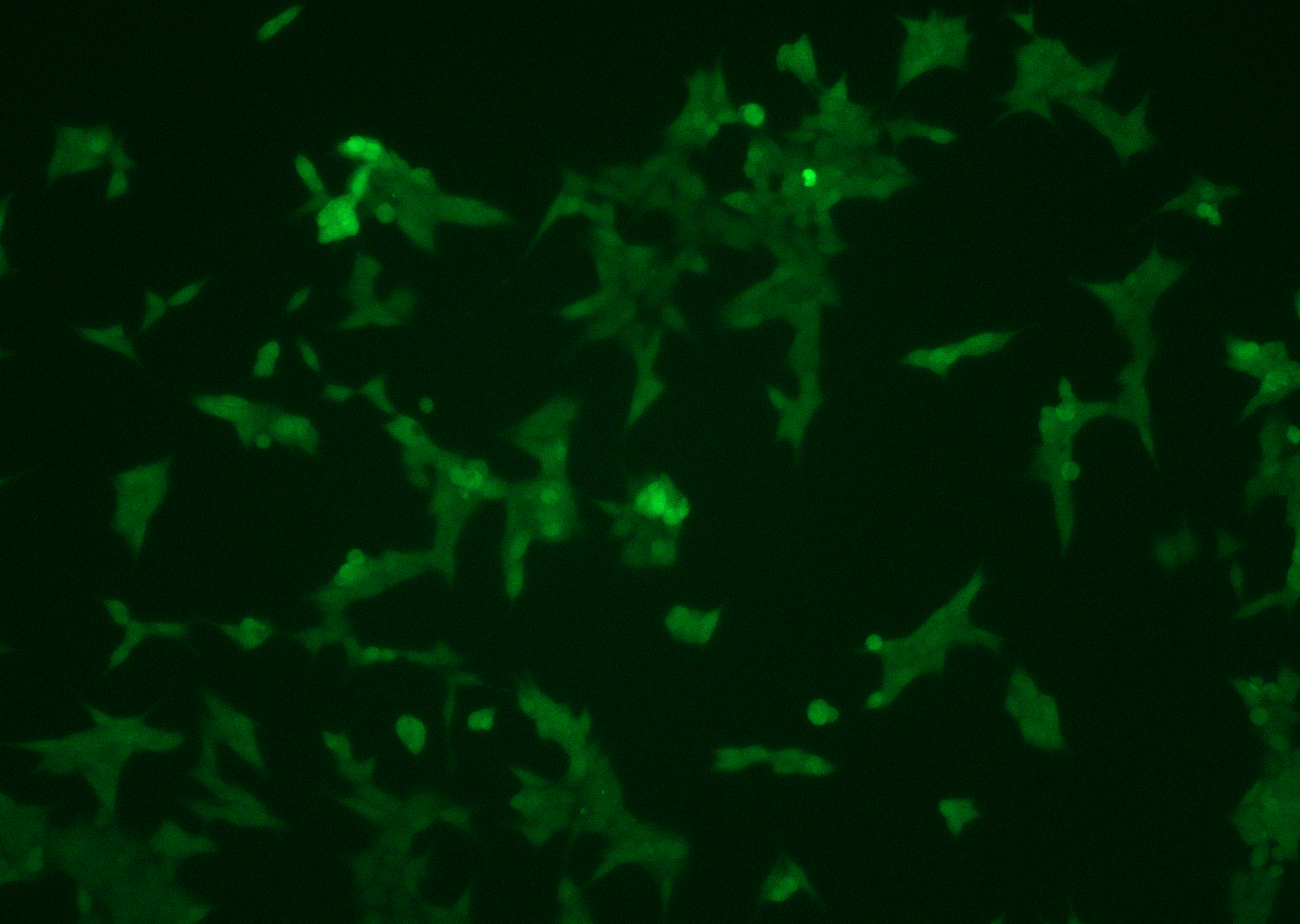

Supplement: Supplementary file 8 — Source data Fig. 6 [file 44321_2025_308_MOESM8_ESM.zip › Figure 6/6d/1.5 (1).tif]

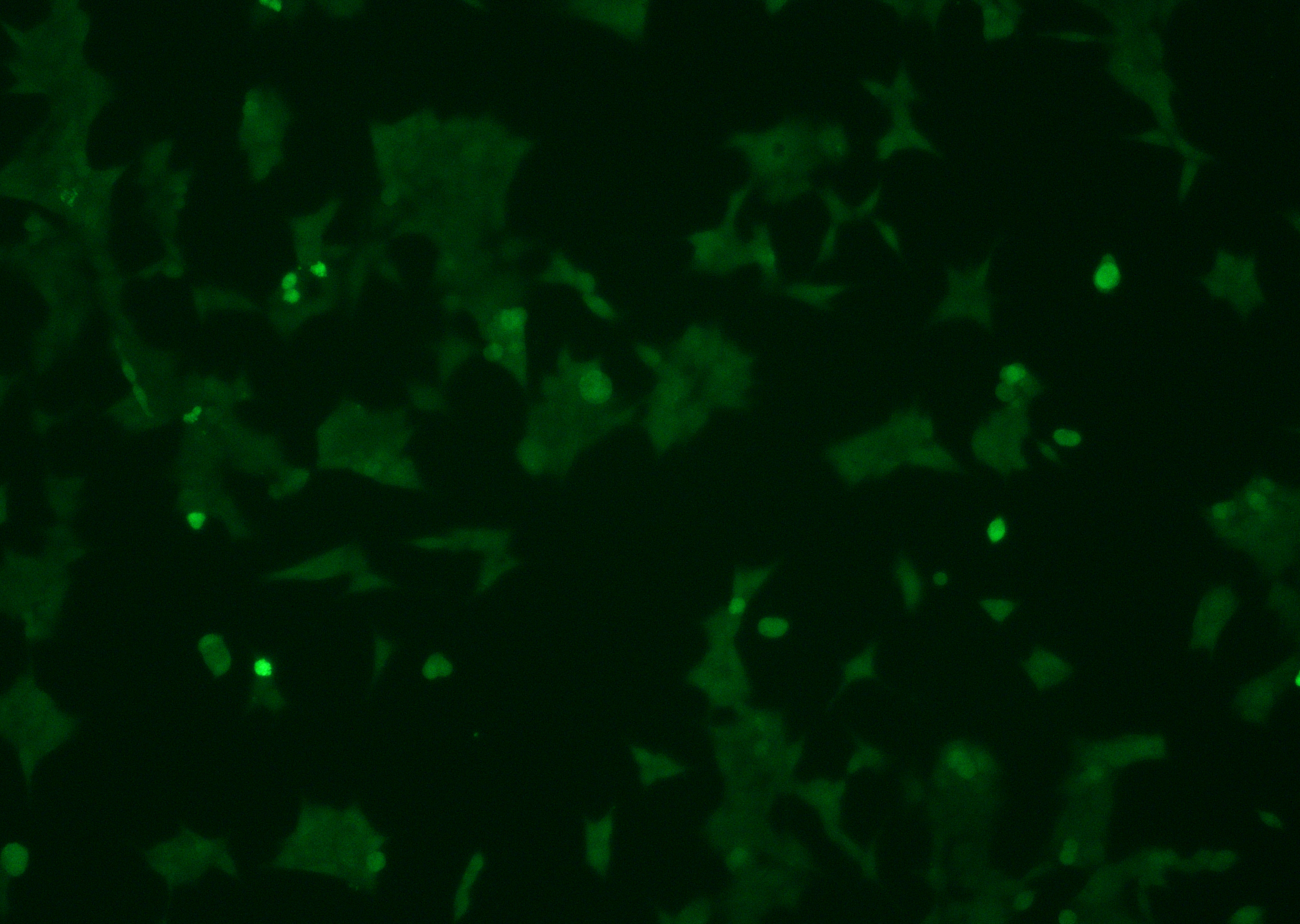

Supplement: Supplementary file 8 — Source data Fig. 6 [file 44321_2025_308_MOESM8_ESM.zip › Figure 6/6d/1.5 (2).tif]

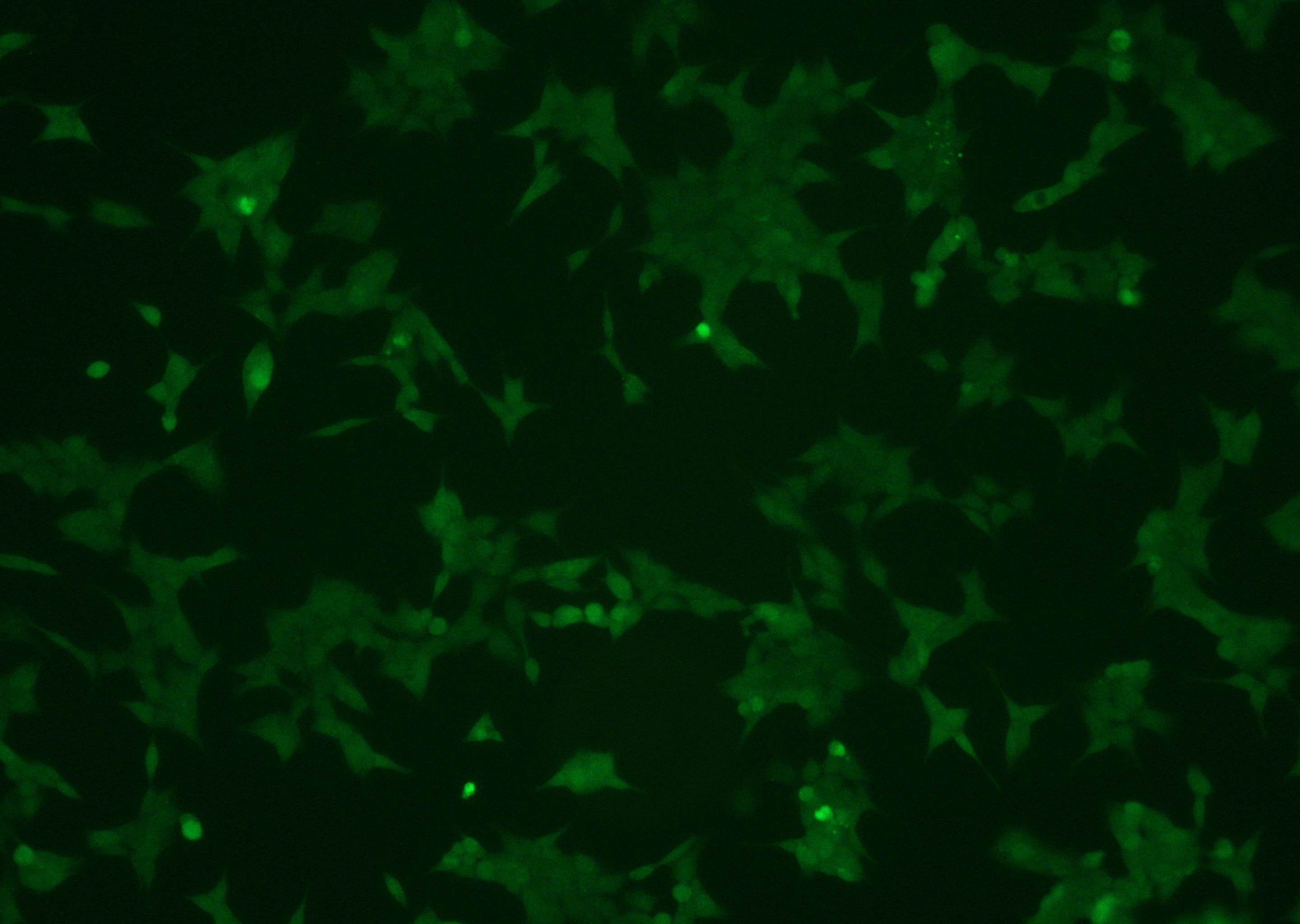

Supplement: Supplementary file 8 — Source data Fig. 6 [file 44321_2025_308_MOESM8_ESM.zip › Figure 6/6d/1.5 (3).tif]

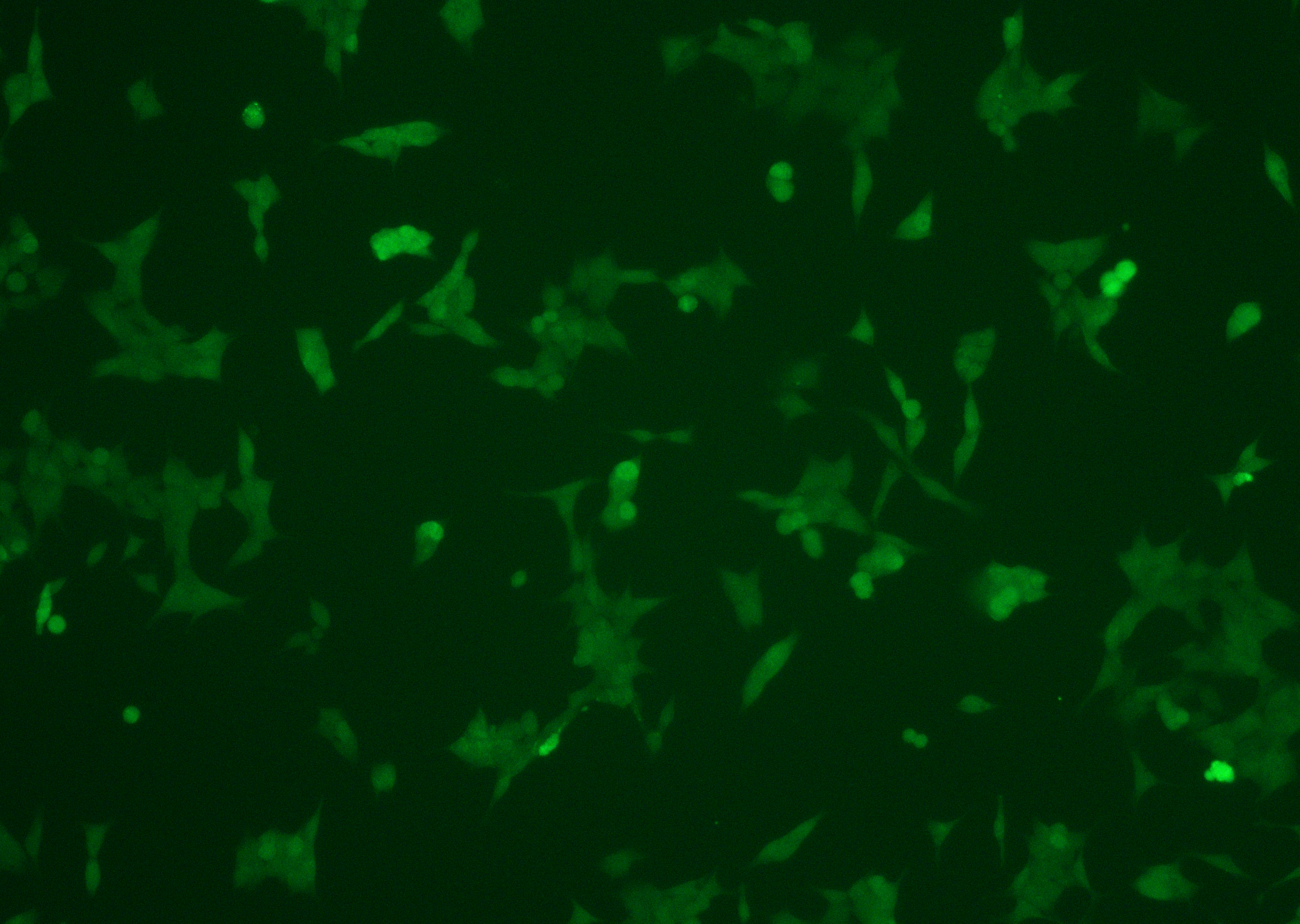

Supplement: Supplementary file 8 — Source data Fig. 6 [file 44321_2025_308_MOESM8_ESM.zip › Figure 6/6d/3 (1).tif]

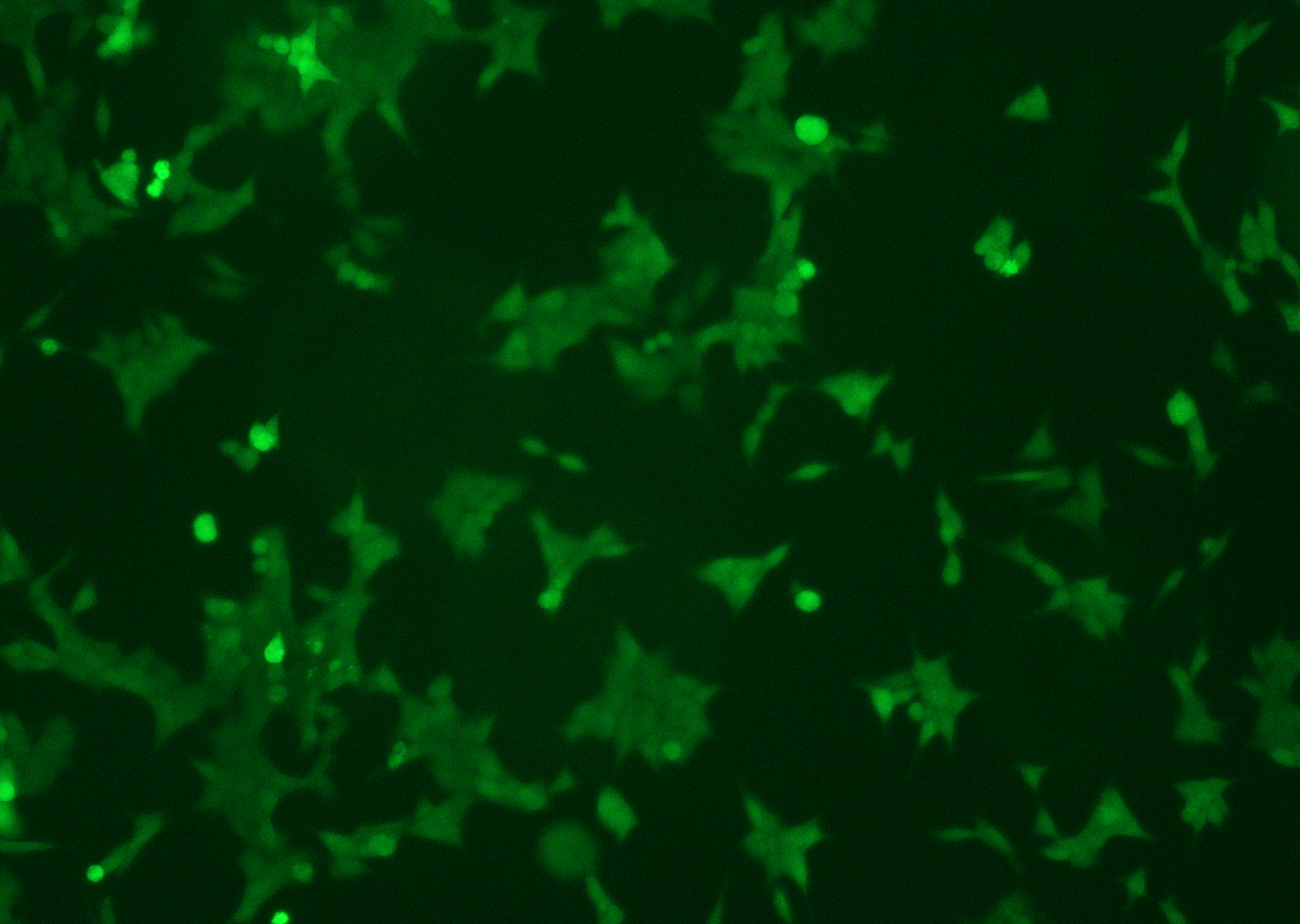

Supplement: Supplementary file 8 — Source data Fig. 6 [file 44321_2025_308_MOESM8_ESM.zip › Figure 6/6d/3 (2).tif]

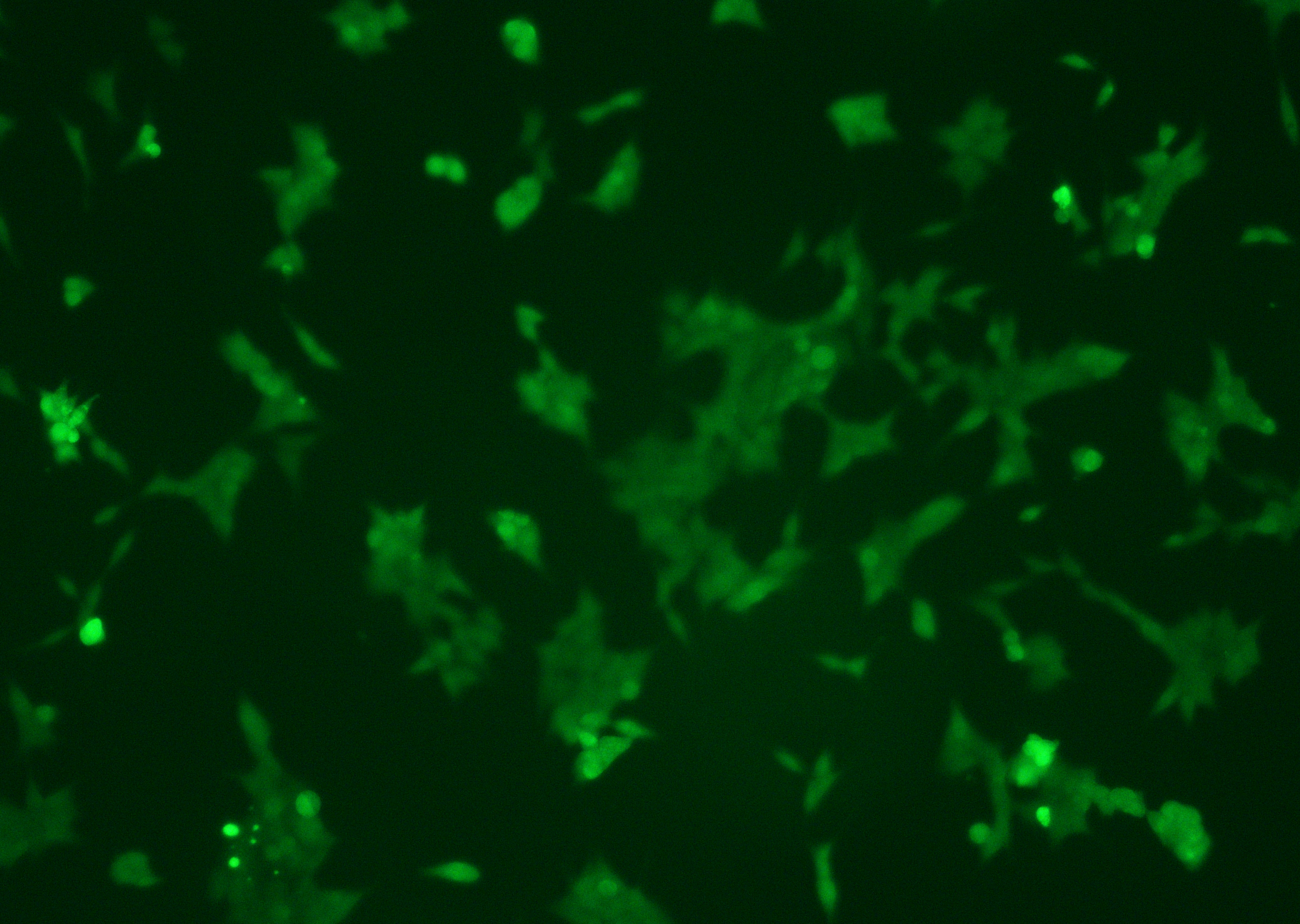

Supplement: Supplementary file 8 — Source data Fig. 6 [file 44321_2025_308_MOESM8_ESM.zip › Figure 6/6d/3 (3).tif]

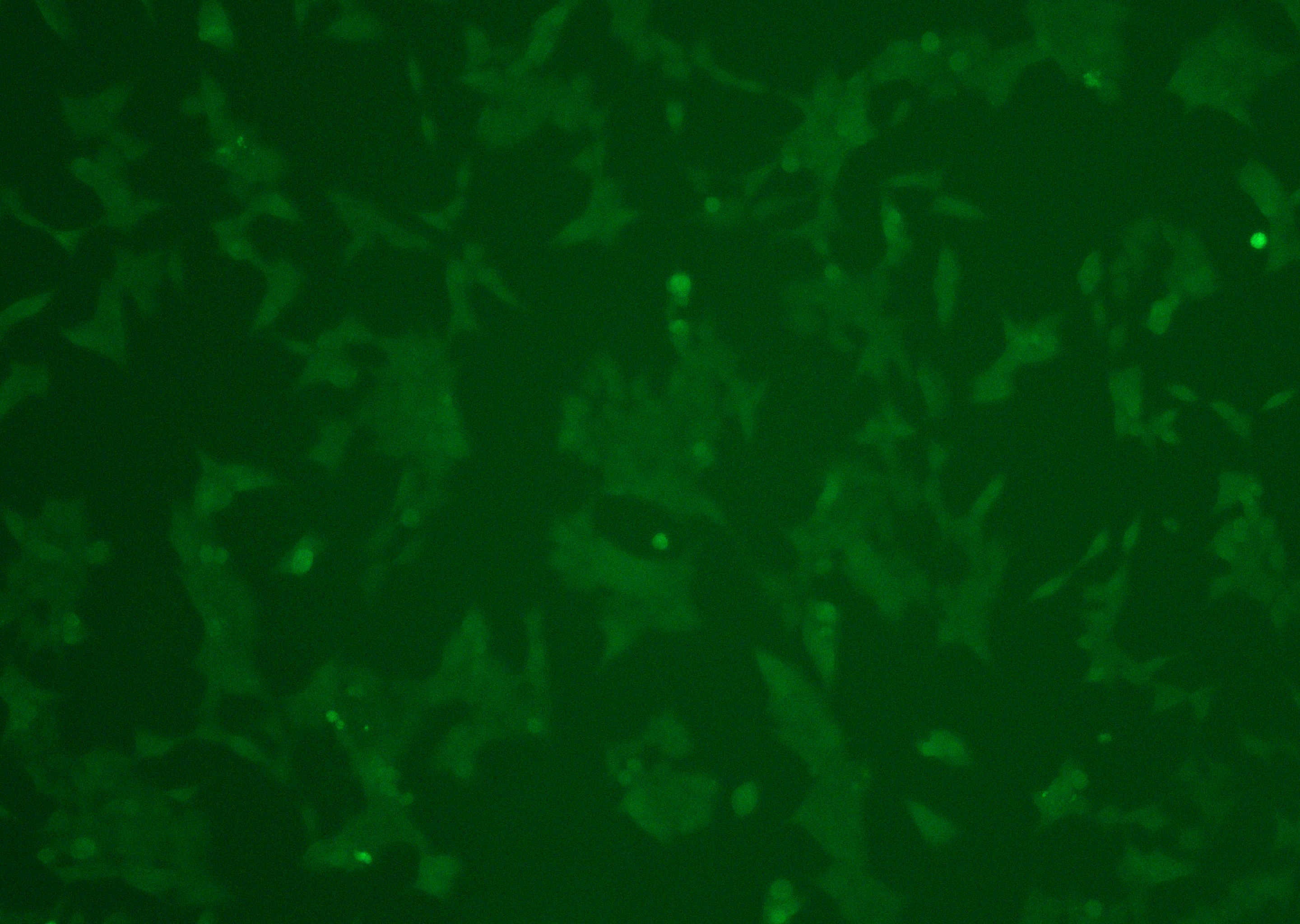

Supplement: Supplementary file 8 — Source data Fig. 6 [file 44321_2025_308_MOESM8_ESM.zip › Figure 6/6d/Con (1).tif]

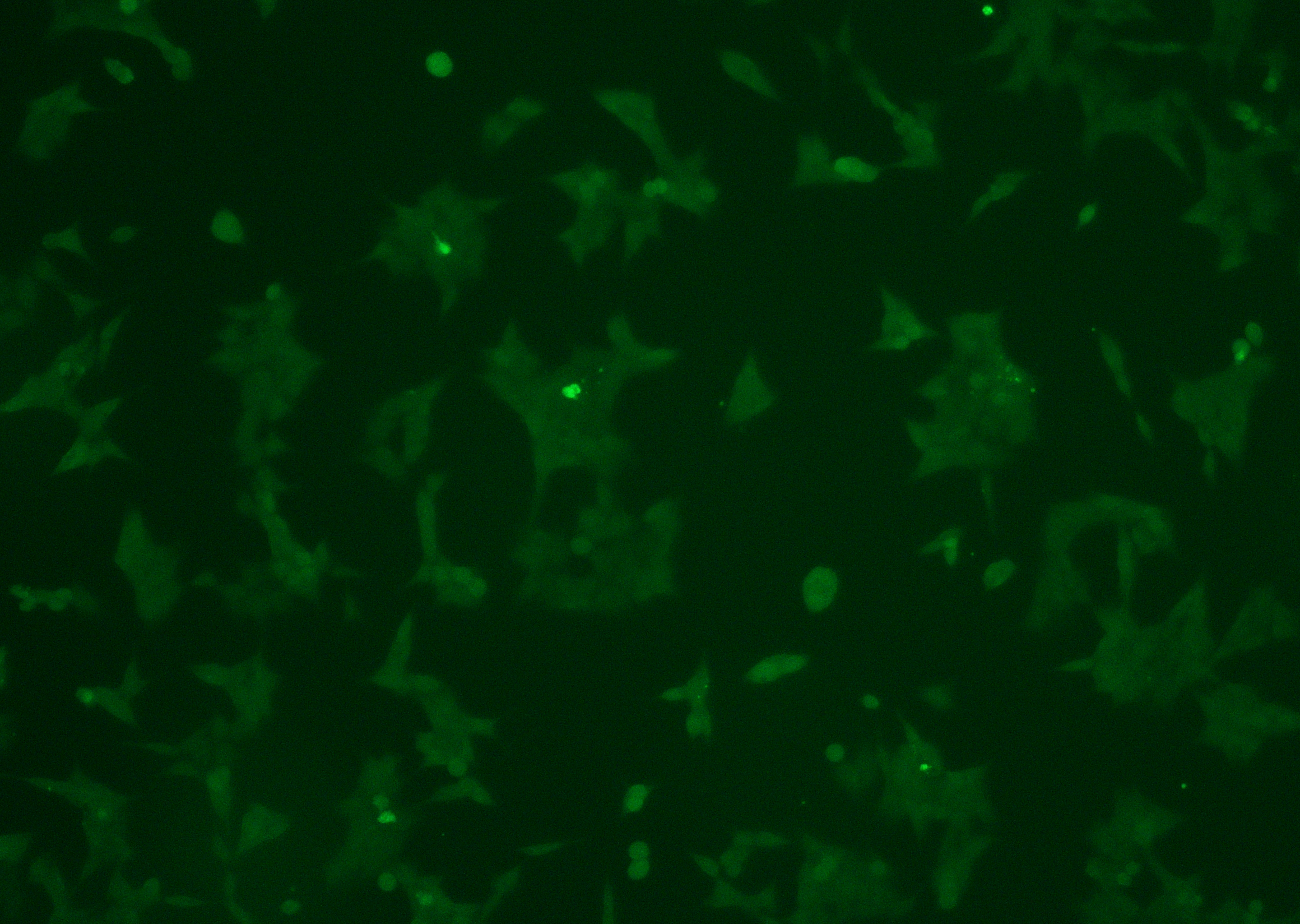

Supplement: Supplementary file 8 — Source data Fig. 6 [file 44321_2025_308_MOESM8_ESM.zip › Figure 6/6d/Con (2).tif]

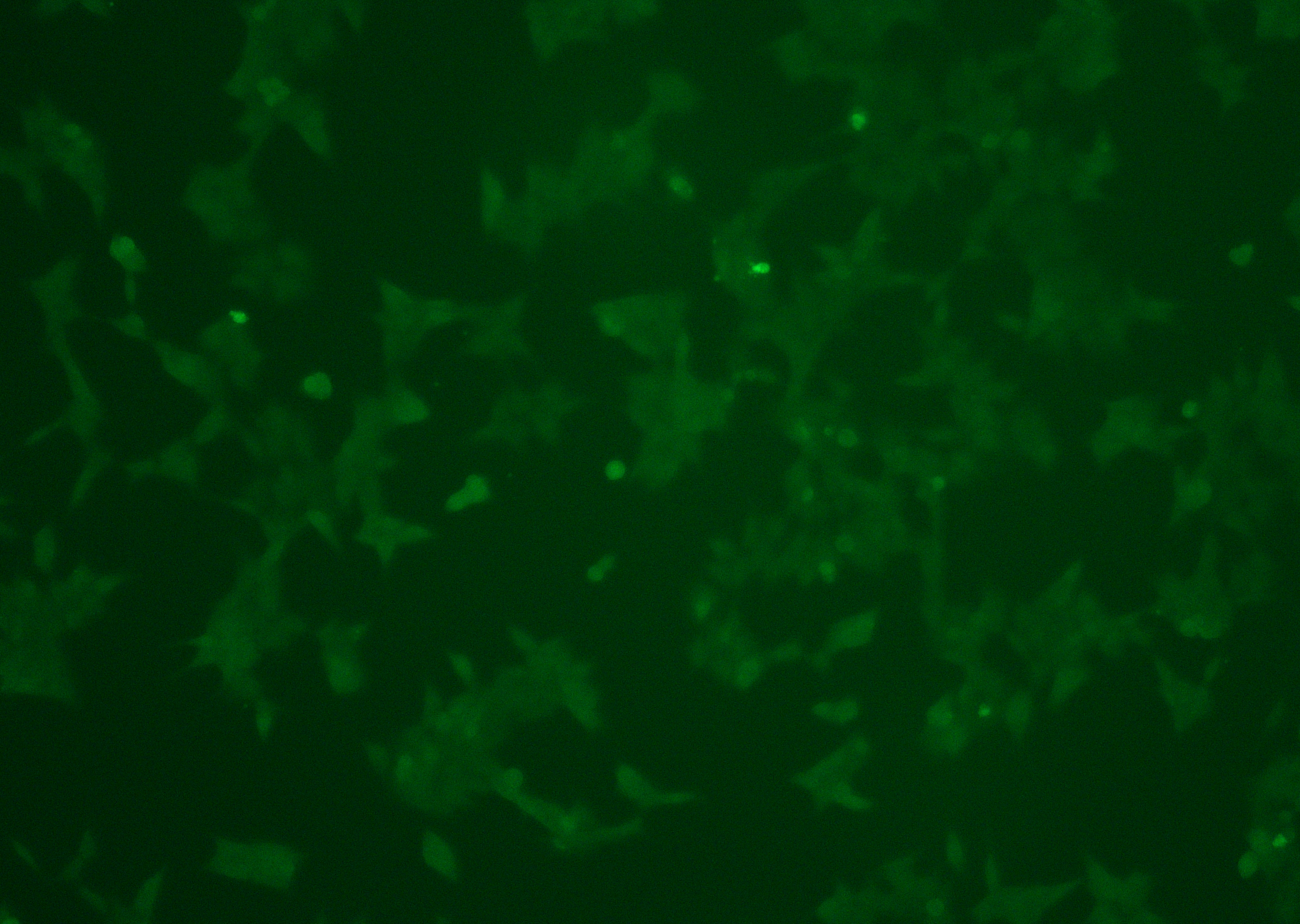

Supplement: Supplementary file 8 — Source data Fig. 6 [file 44321_2025_308_MOESM8_ESM.zip › Figure 6/6d/Con (3).tif]

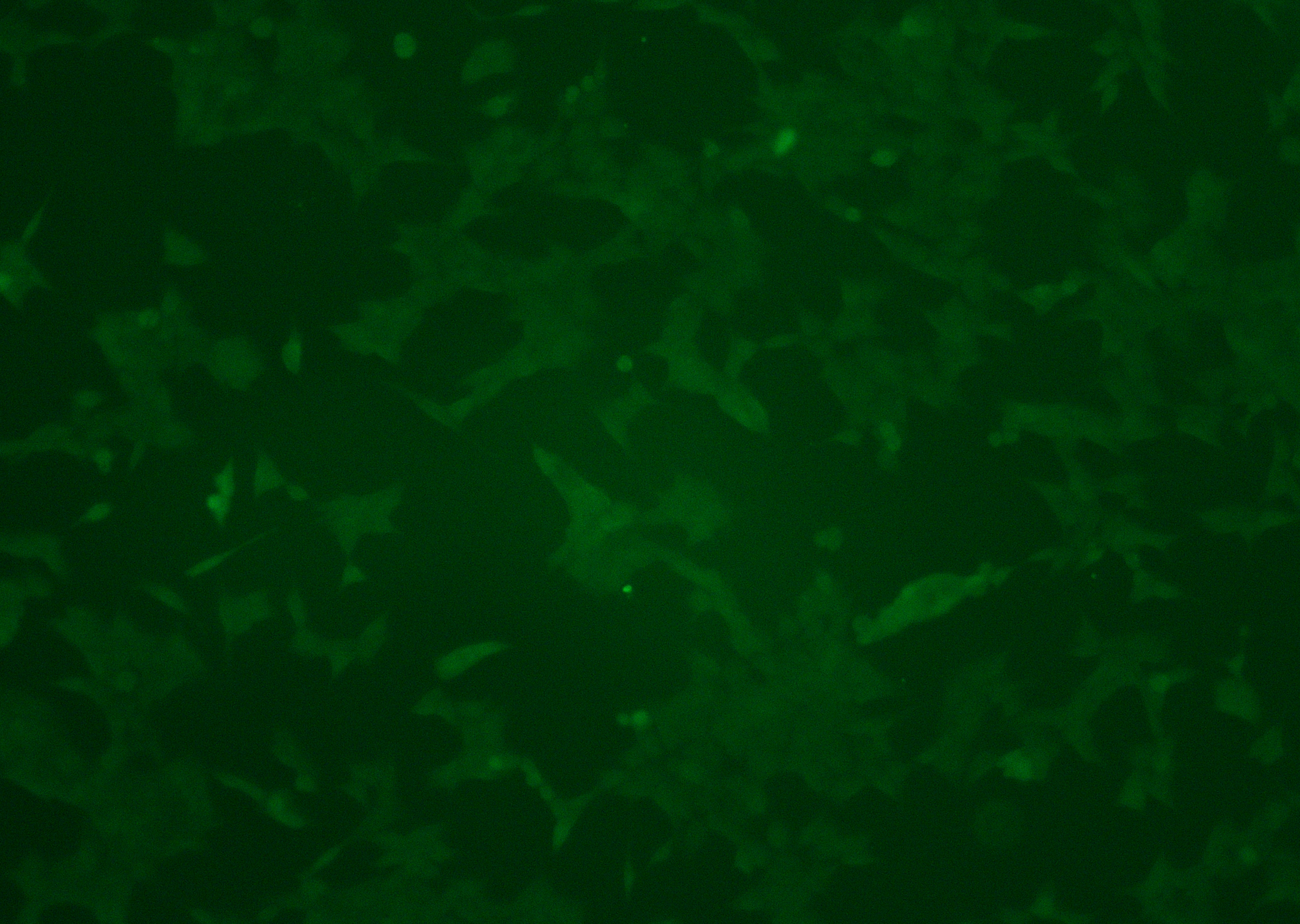

Supplement: Supplementary file 8 — Source data Fig. 6 [file 44321_2025_308_MOESM8_ESM.zip › Figure 6/6d/NAC (1).tif]

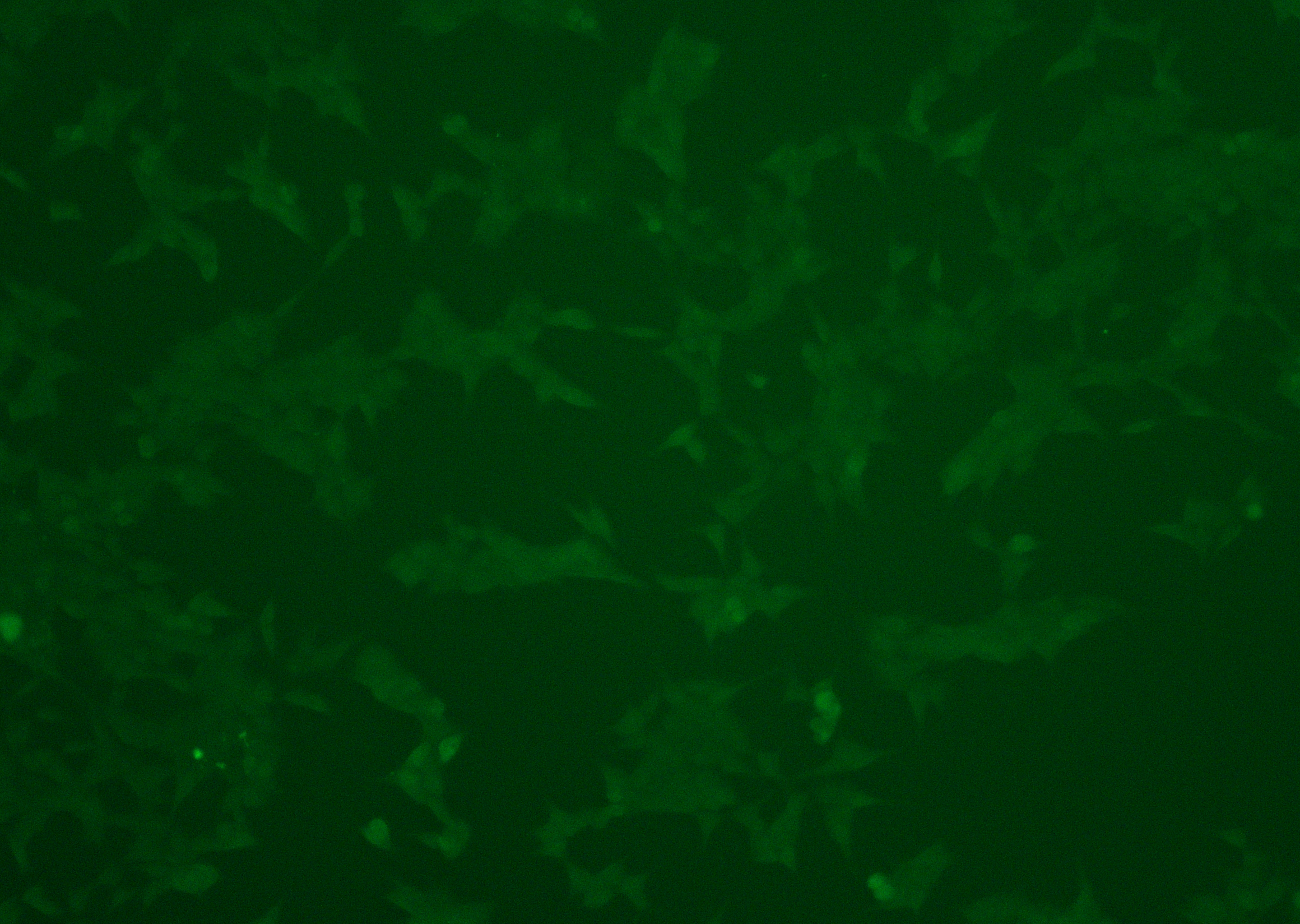

Supplement: Supplementary file 8 — Source data Fig. 6 [file 44321_2025_308_MOESM8_ESM.zip › Figure 6/6d/NAC (2).tif]

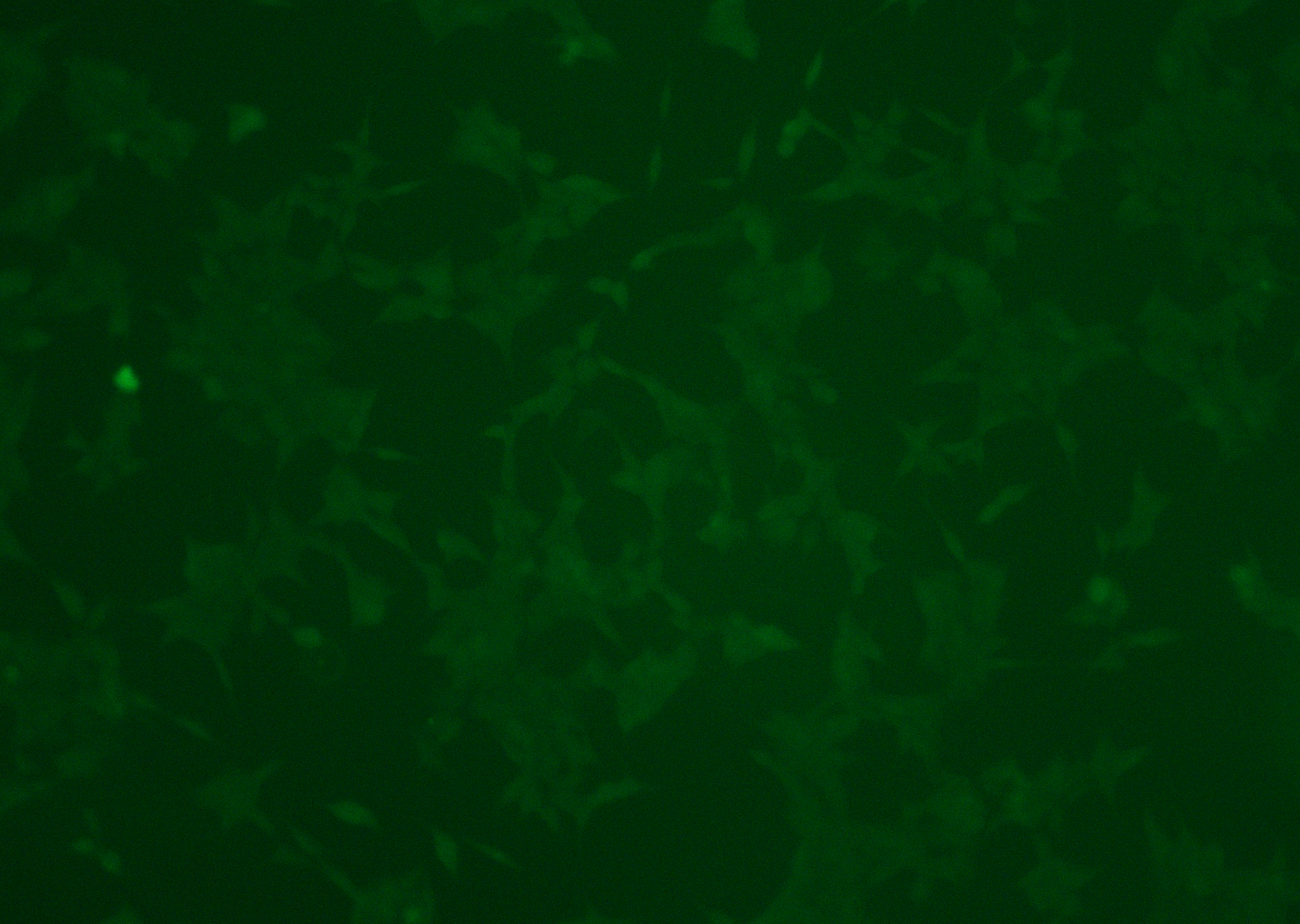

Supplement: Supplementary file 8 — Source data Fig. 6 [file 44321_2025_308_MOESM8_ESM.zip › Figure 6/6d/NAC (3).tif]

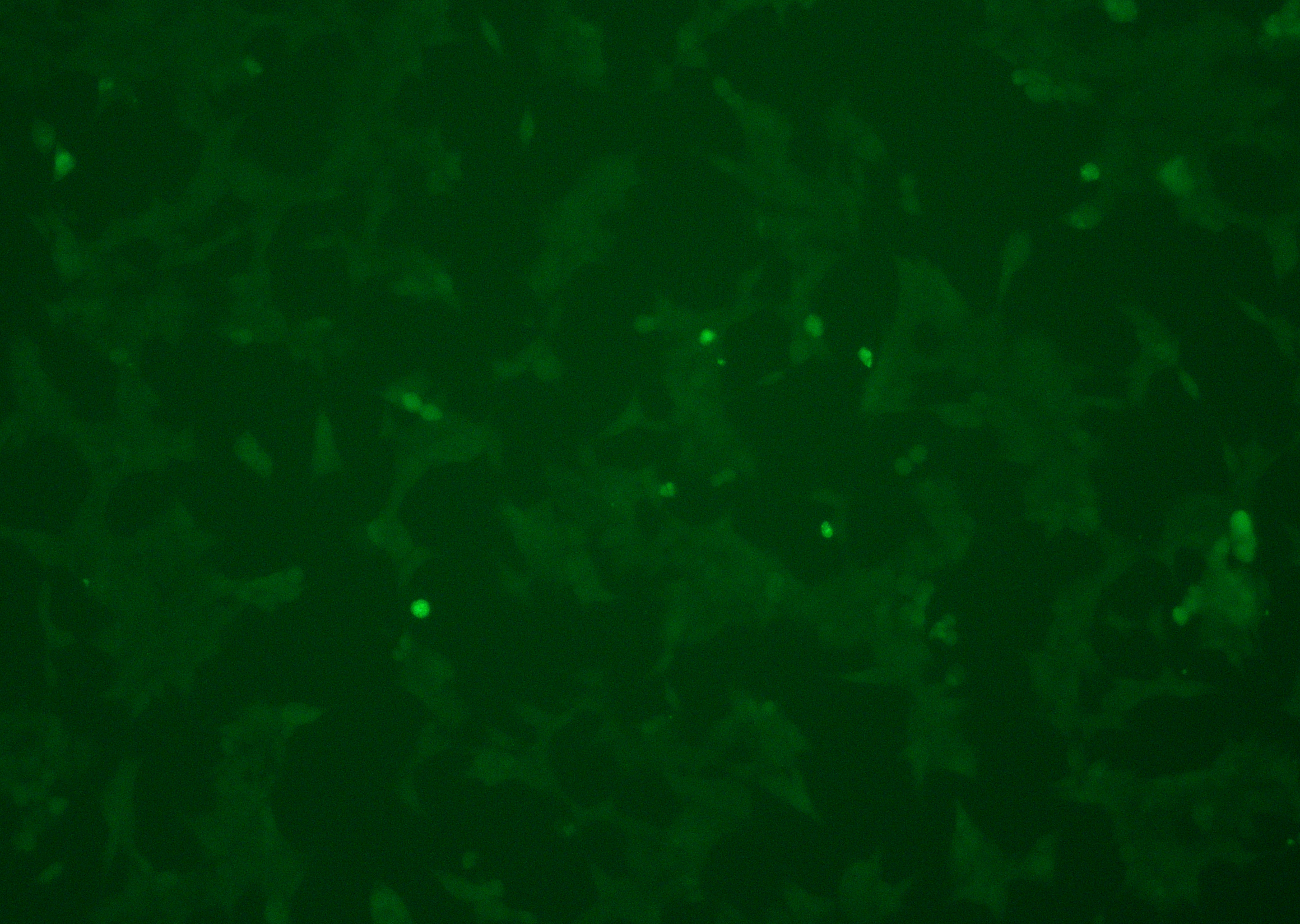

Supplement: Supplementary file 8 — Source data Fig. 6 [file 44321_2025_308_MOESM8_ESM.zip › Figure 6/6d/NAC+3 (1).tif]

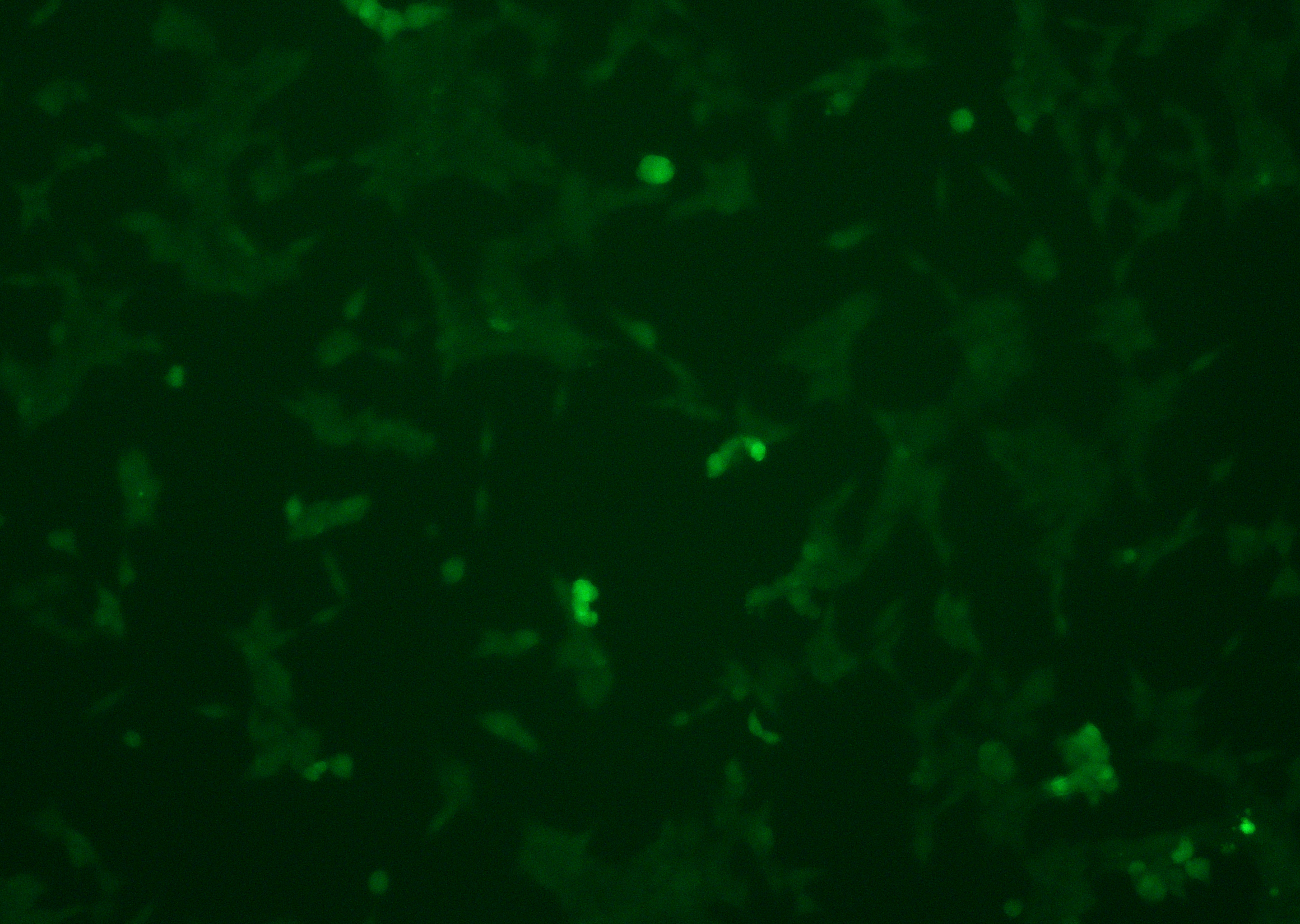

Supplement: Supplementary file 8 — Source data Fig. 6 [file 44321_2025_308_MOESM8_ESM.zip › Figure 6/6d/NAC+3 (2).tif]

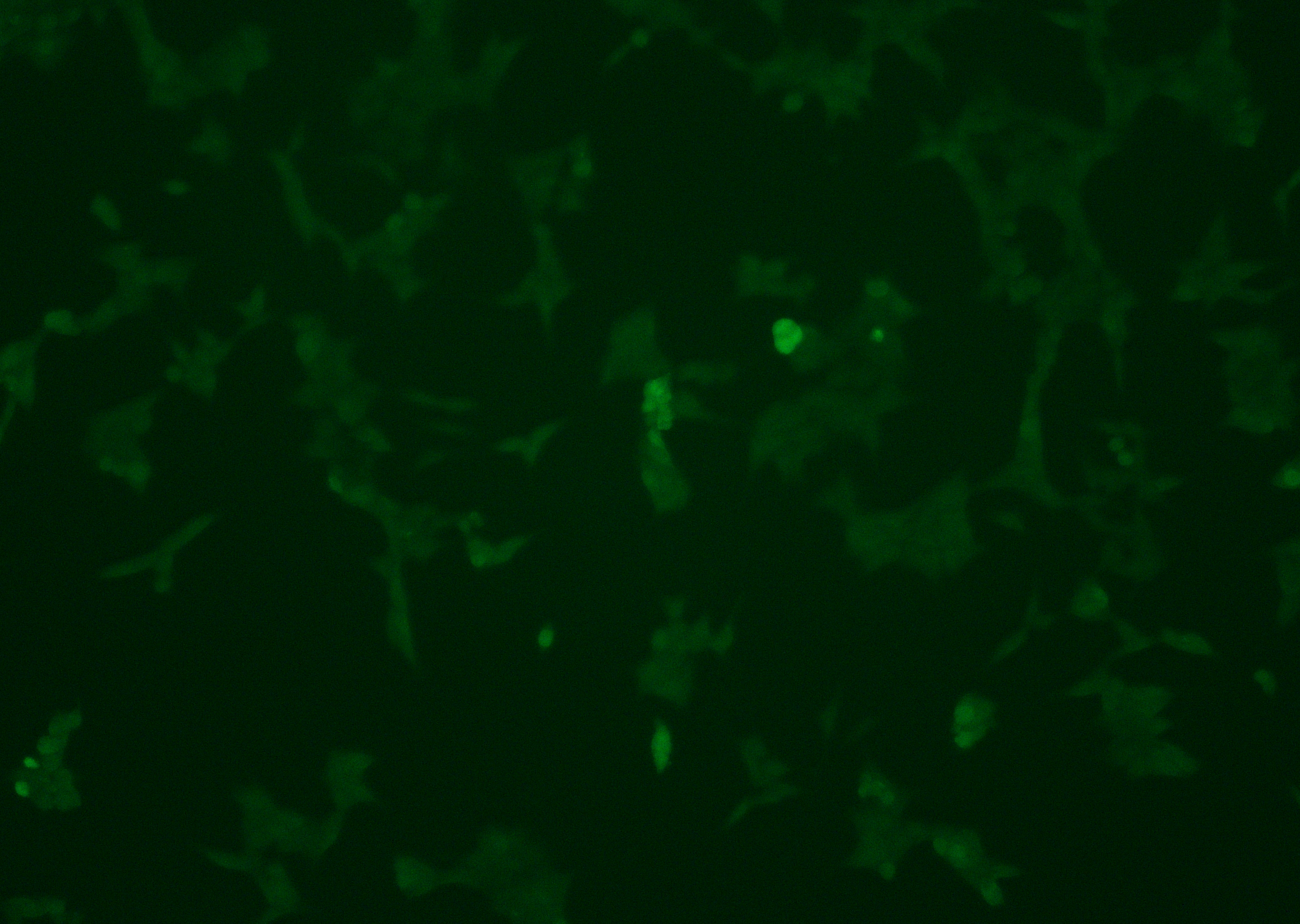

Supplement: Supplementary file 8 — Source data Fig. 6 [file 44321_2025_308_MOESM8_ESM.zip › Figure 6/6d/NAC+3 (3).tif]

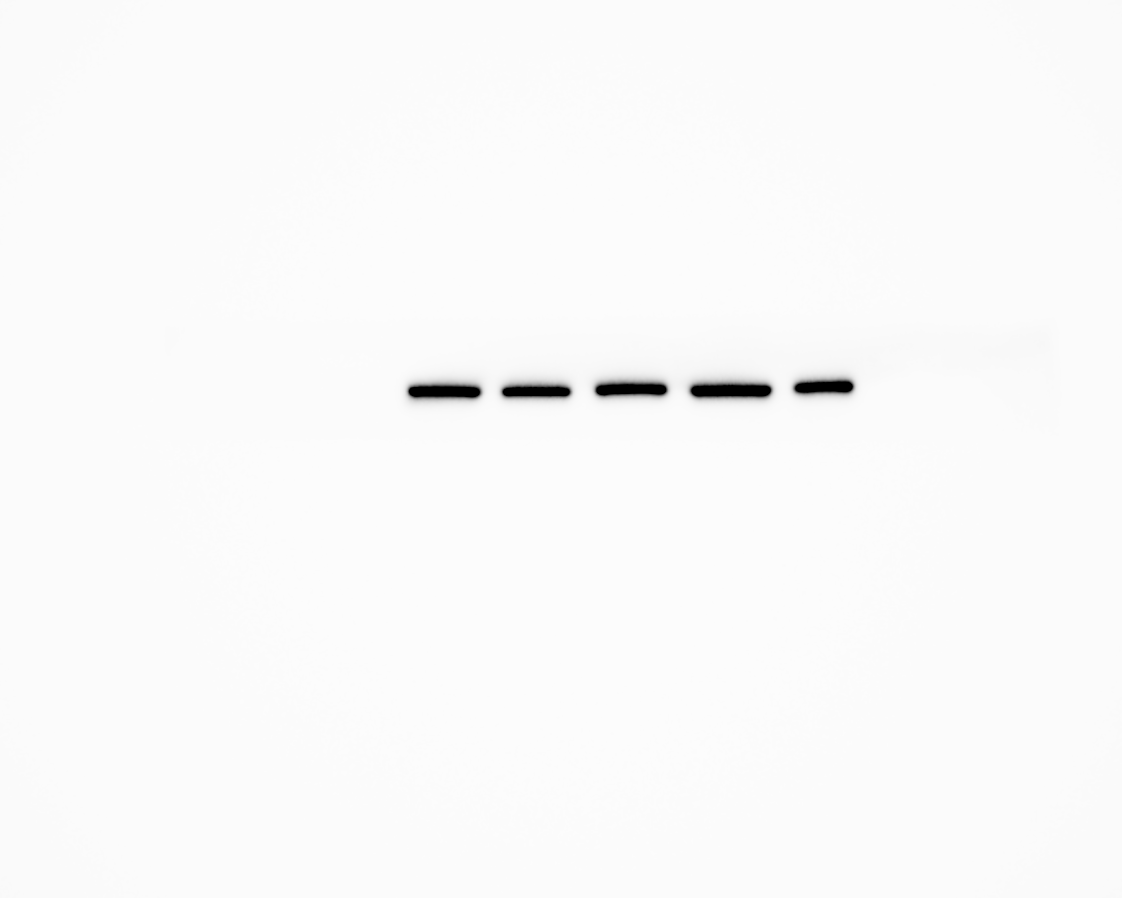

Supplement: Supplementary file 8 — Source data Fig. 6 [file 44321_2025_308_MOESM8_ESM.zip › Figure 6/6h/western actin 1.tif]

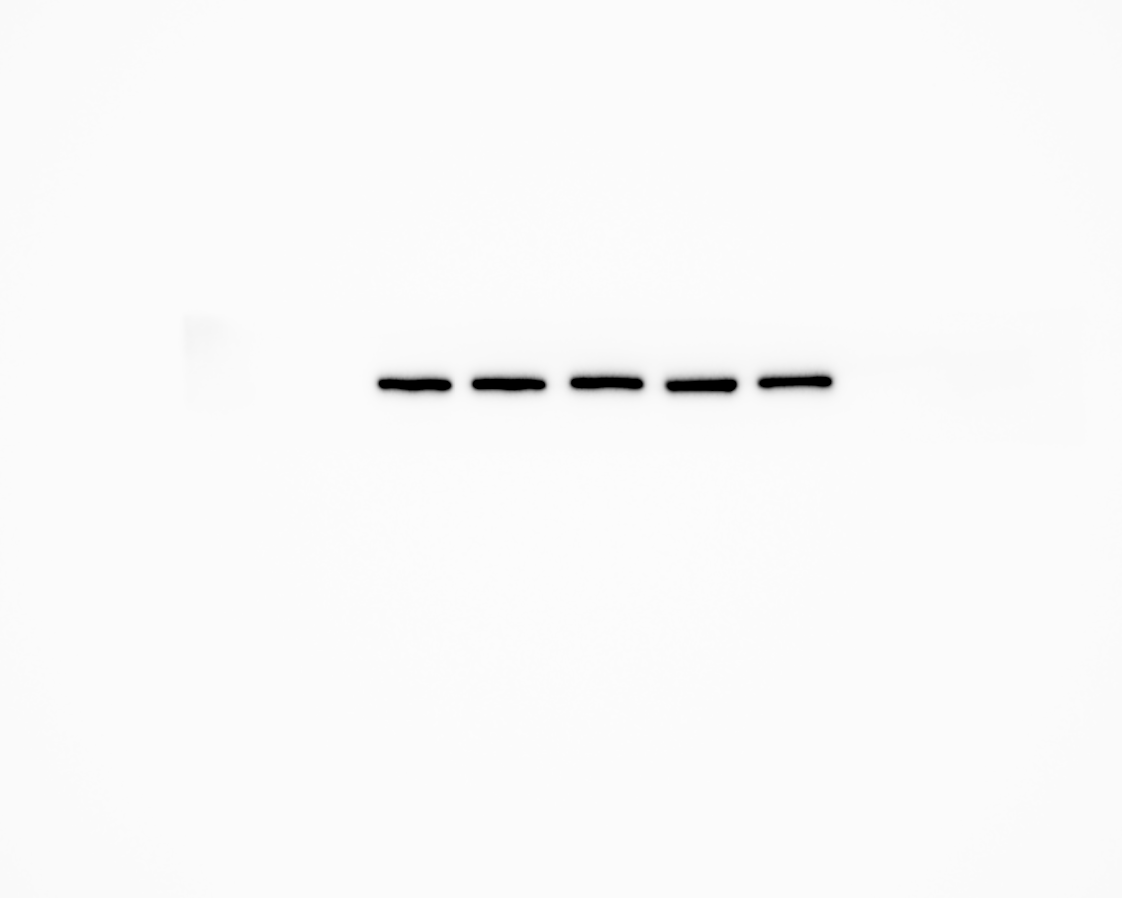

Supplement: Supplementary file 8 — Source data Fig. 6 [file 44321_2025_308_MOESM8_ESM.zip › Figure 6/6h/western actin 2.tif]

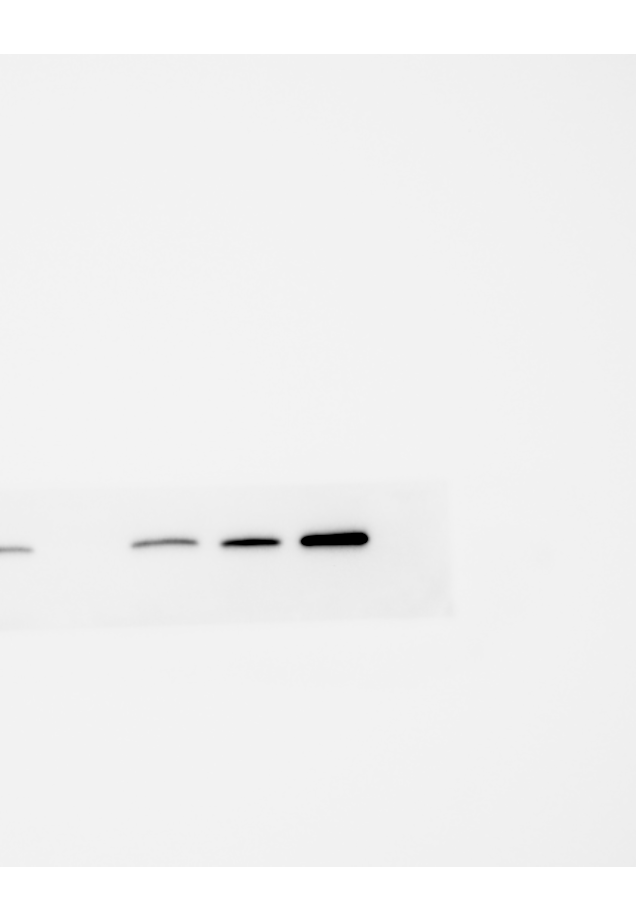

Supplement: Supplementary file 8 — Source data Fig. 6 [file 44321_2025_308_MOESM8_ESM.zip › Figure 6/6h/western bax 1.tif]

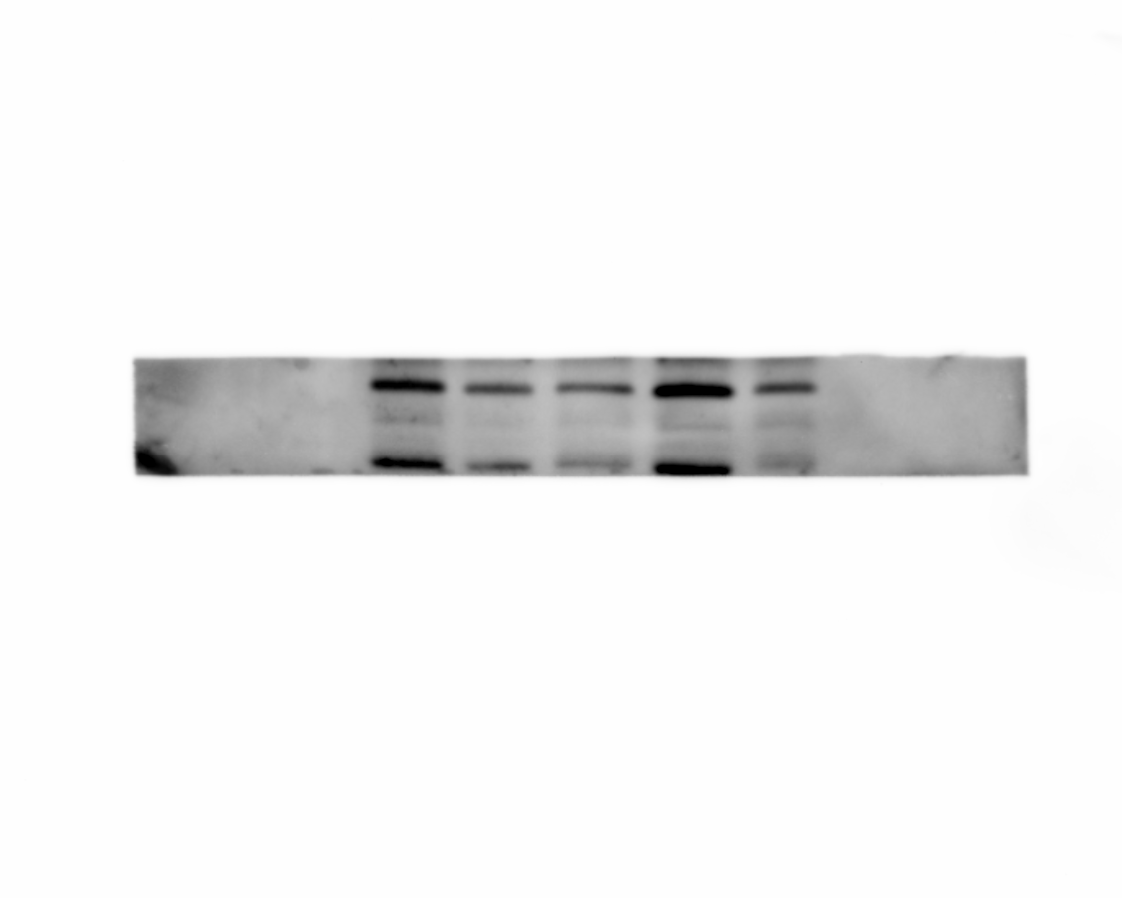

Supplement: Supplementary file 8 — Source data Fig. 6 [file 44321_2025_308_MOESM8_ESM.zip › Figure 6/6h/western bcl2 1.tif]

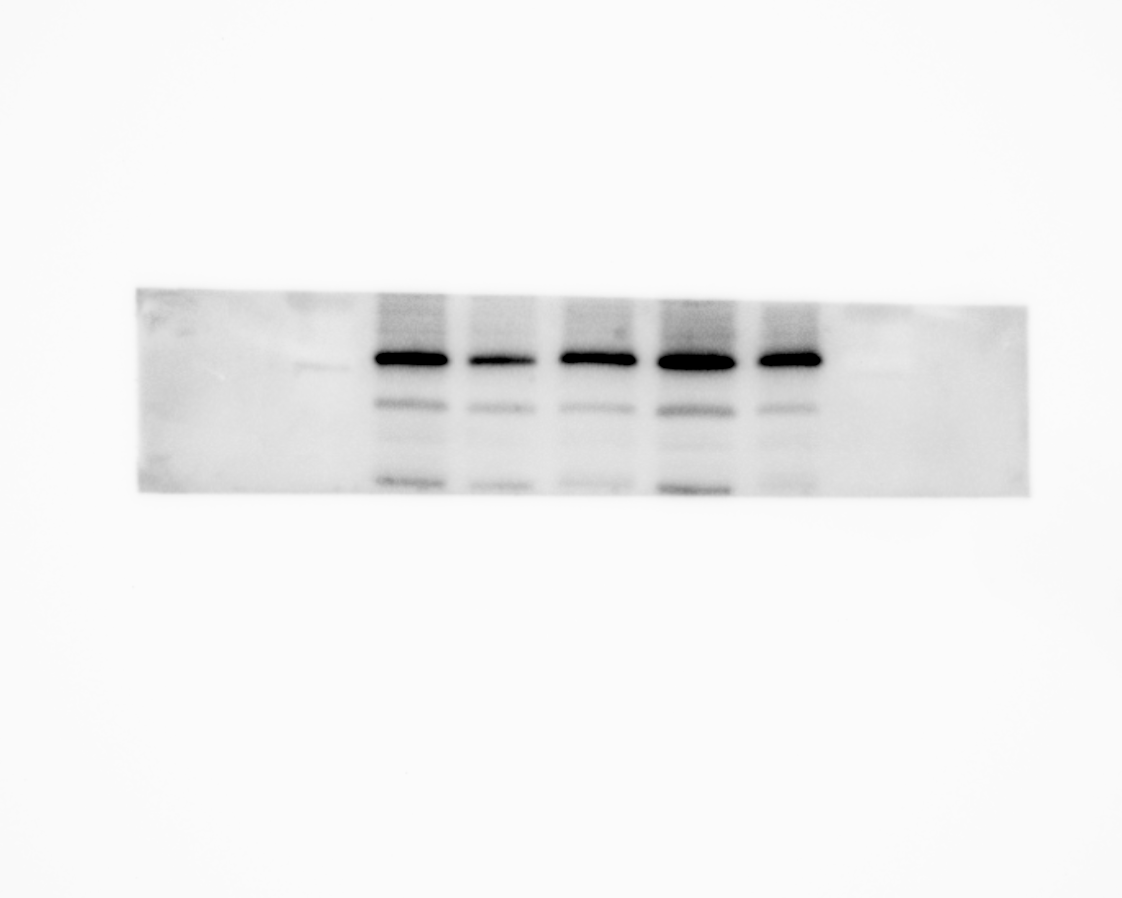

Supplement: Supplementary file 8 — Source data Fig. 6 [file 44321_2025_308_MOESM8_ESM.zip › Figure 6/6h/western bcl2 2.tif]

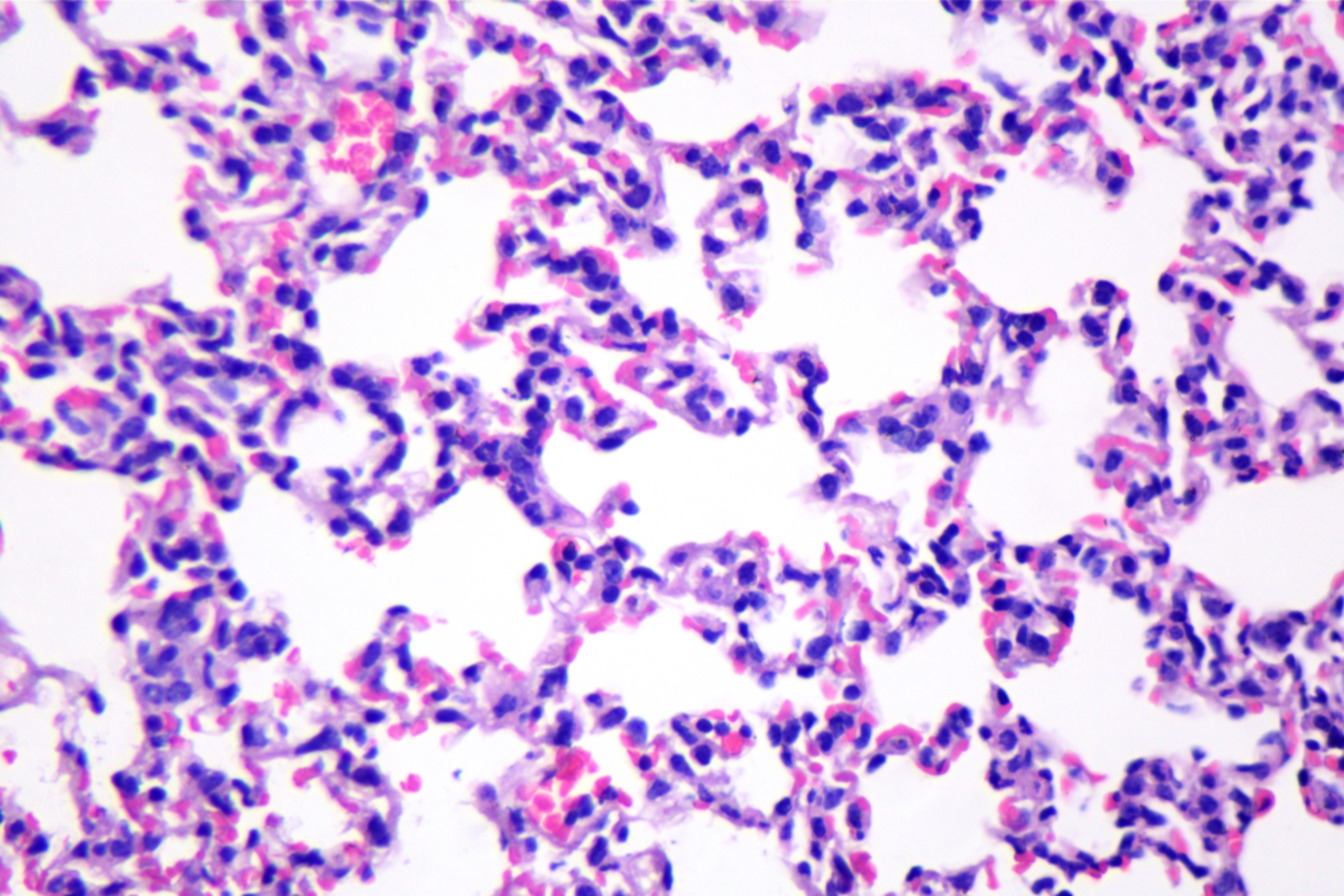

Supplement: Supplementary file 10 — Appendix Figure Source Data [file 44321_2025_308_MOESM10_ESM.zip › AF S5/5 A lung/control-lung 40X (1).jpg]
