# Supplementary figures and images for: AI-identified CD133-targeting natural compounds demonstrate differential anti-tumor effects and mechanisms in pan-cancer models (part 4 of 4)
Source: EMBO Mol Med. 2025 Oct 2;17(11):2932–65. doi: 10.1038/s44321-025-00308-1 (PMC12603267; doi:10.1038/s44321-025-00308-1)

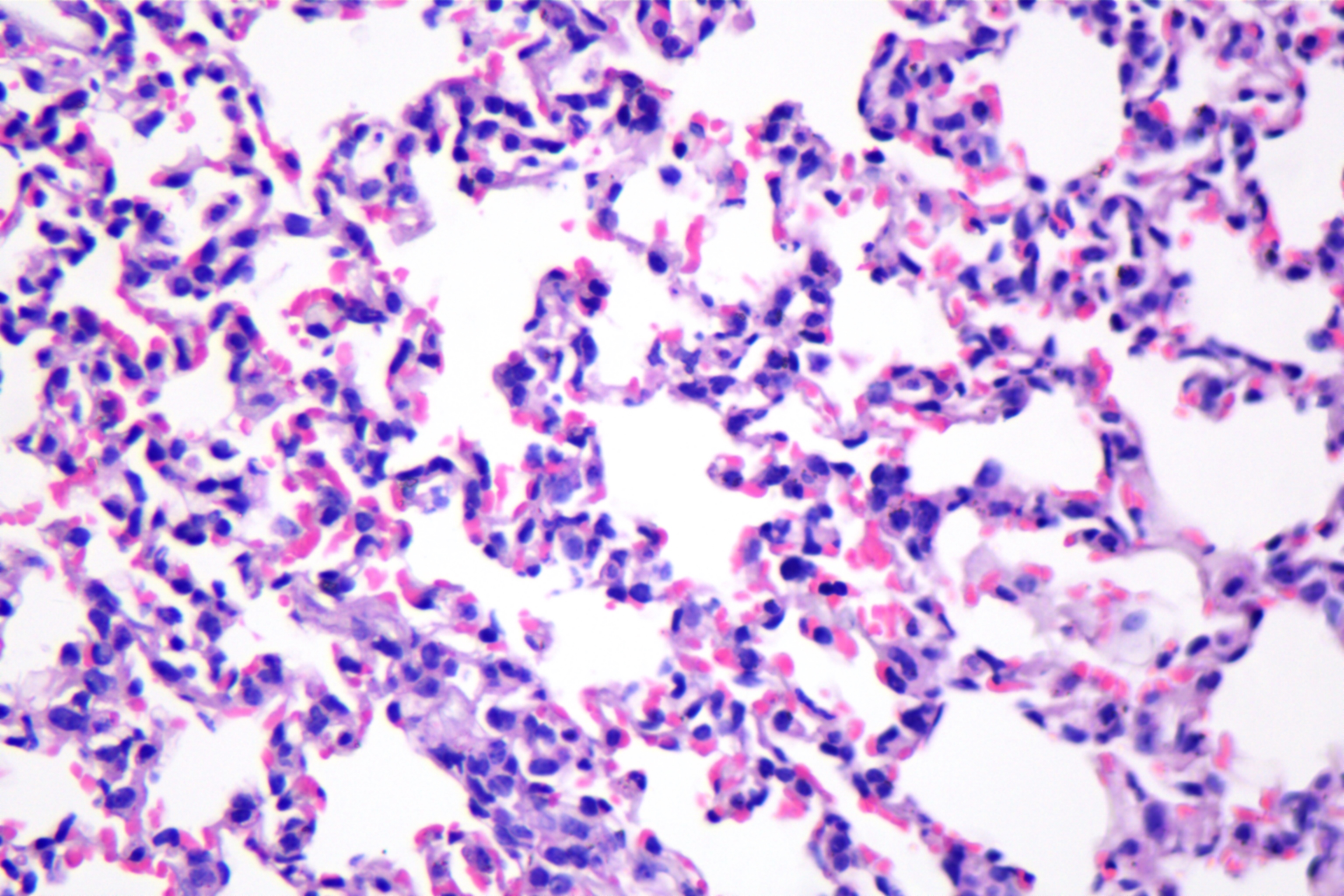

Supplement: Supplementary file 10 — Appendix Figure Source Data [file 44321_2025_308_MOESM10_ESM.zip › AF S5/5 A lung/control-lung 40X (2).jpg]

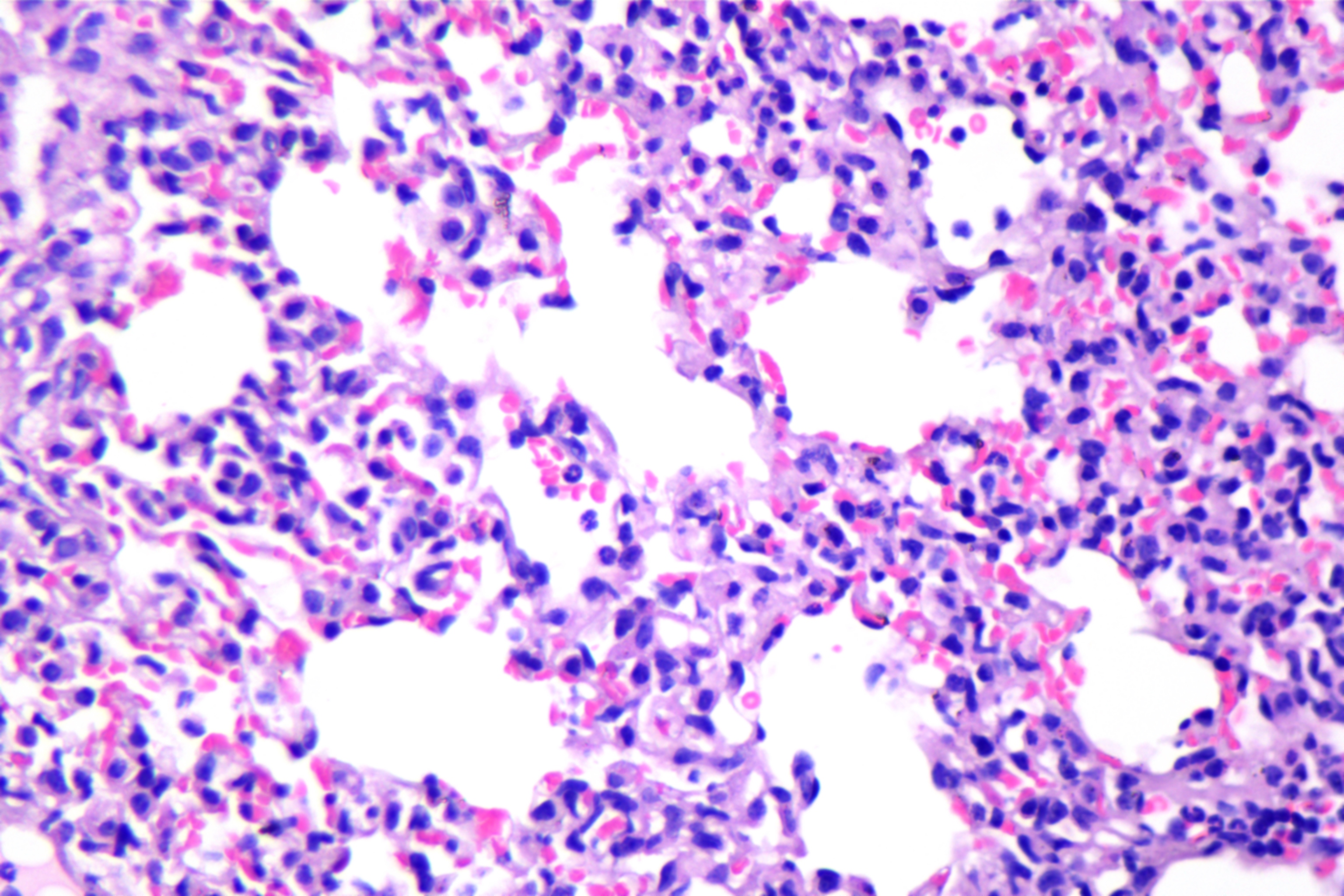

Supplement: Supplementary file 10 — Appendix Figure Source Data [file 44321_2025_308_MOESM10_ESM.zip › AF S5/5 A lung/control-lung 40X (3).jpg]

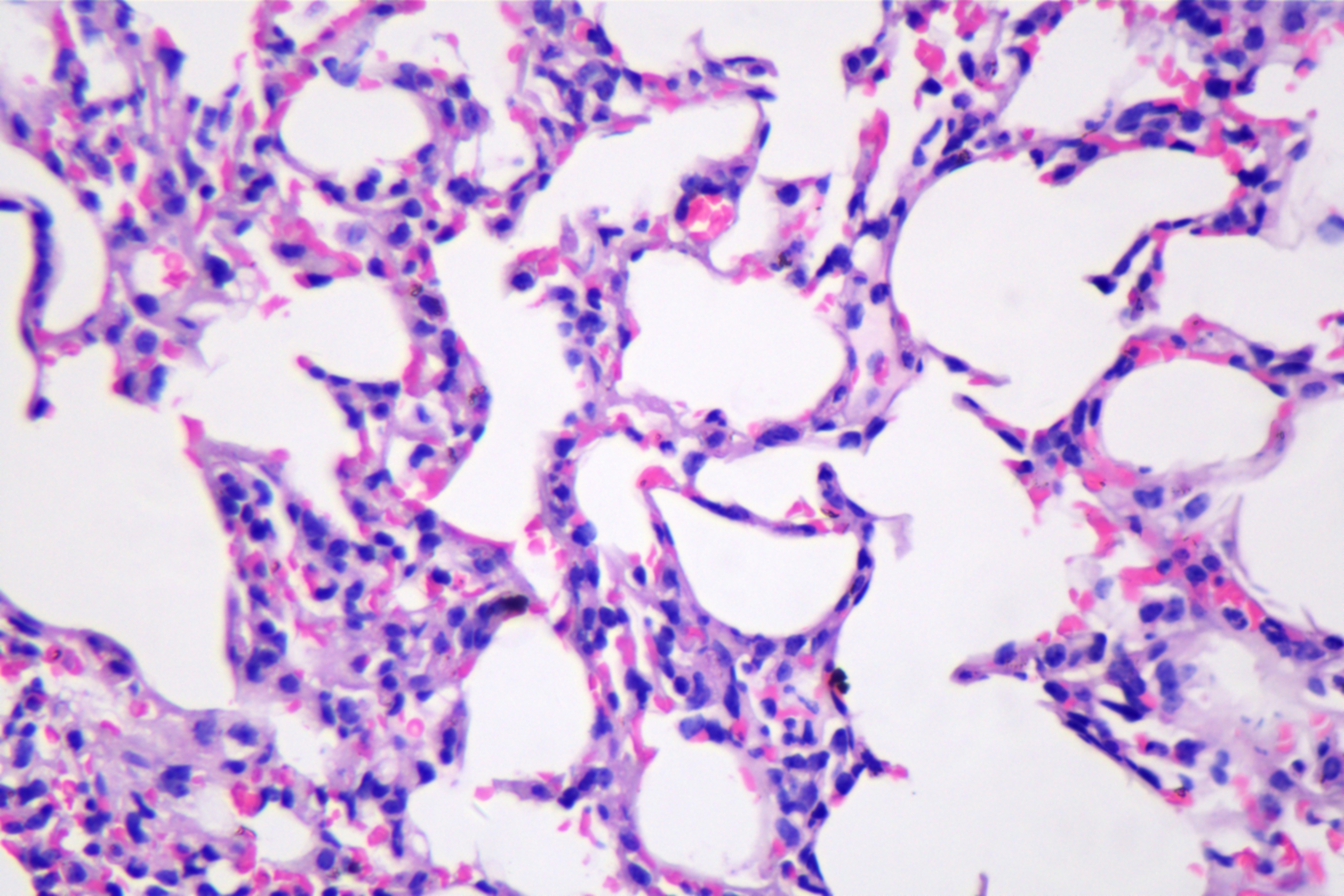

Supplement: Supplementary file 10 — Appendix Figure Source Data [file 44321_2025_308_MOESM10_ESM.zip › AF S5/5 A lung/control-lung 40X (4).jpg]

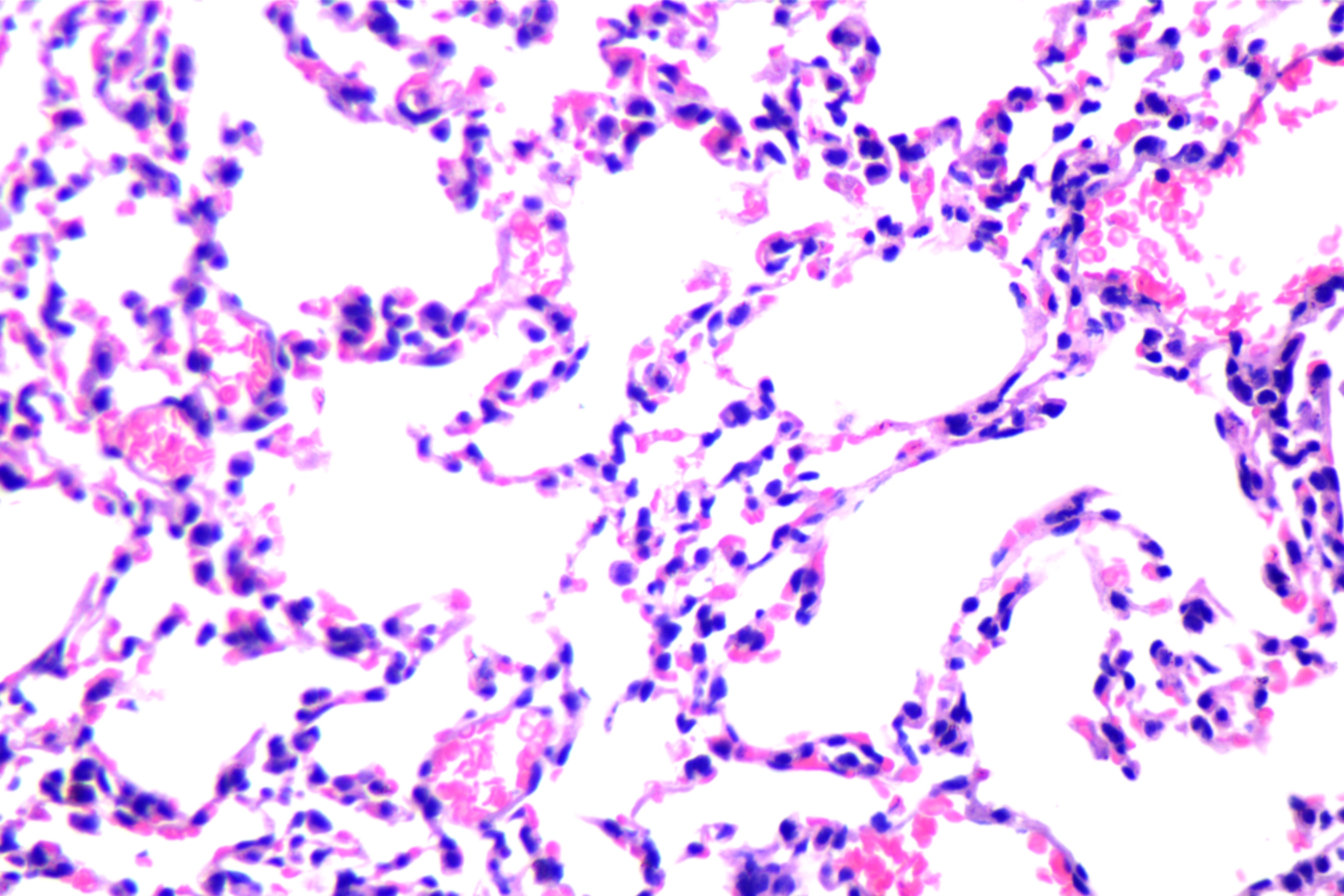

Supplement: Supplementary file 10 — Appendix Figure Source Data [file 44321_2025_308_MOESM10_ESM.zip › AF S5/5 A lung/PP10-2.5-lung 40X (1).jpg]

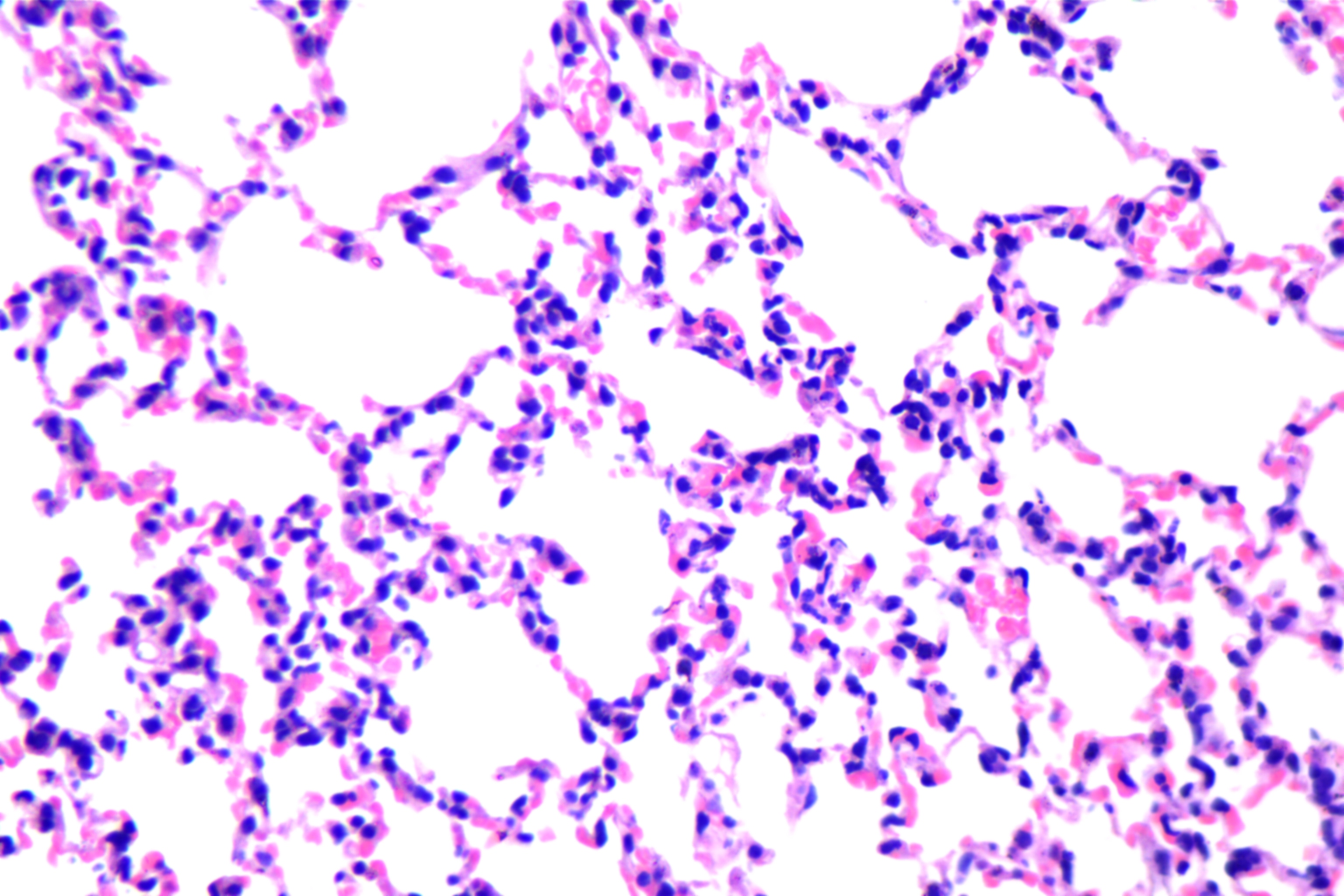

Supplement: Supplementary file 10 — Appendix Figure Source Data [file 44321_2025_308_MOESM10_ESM.zip › AF S5/5 A lung/PP10-2.5-lung 40X (2).jpg]

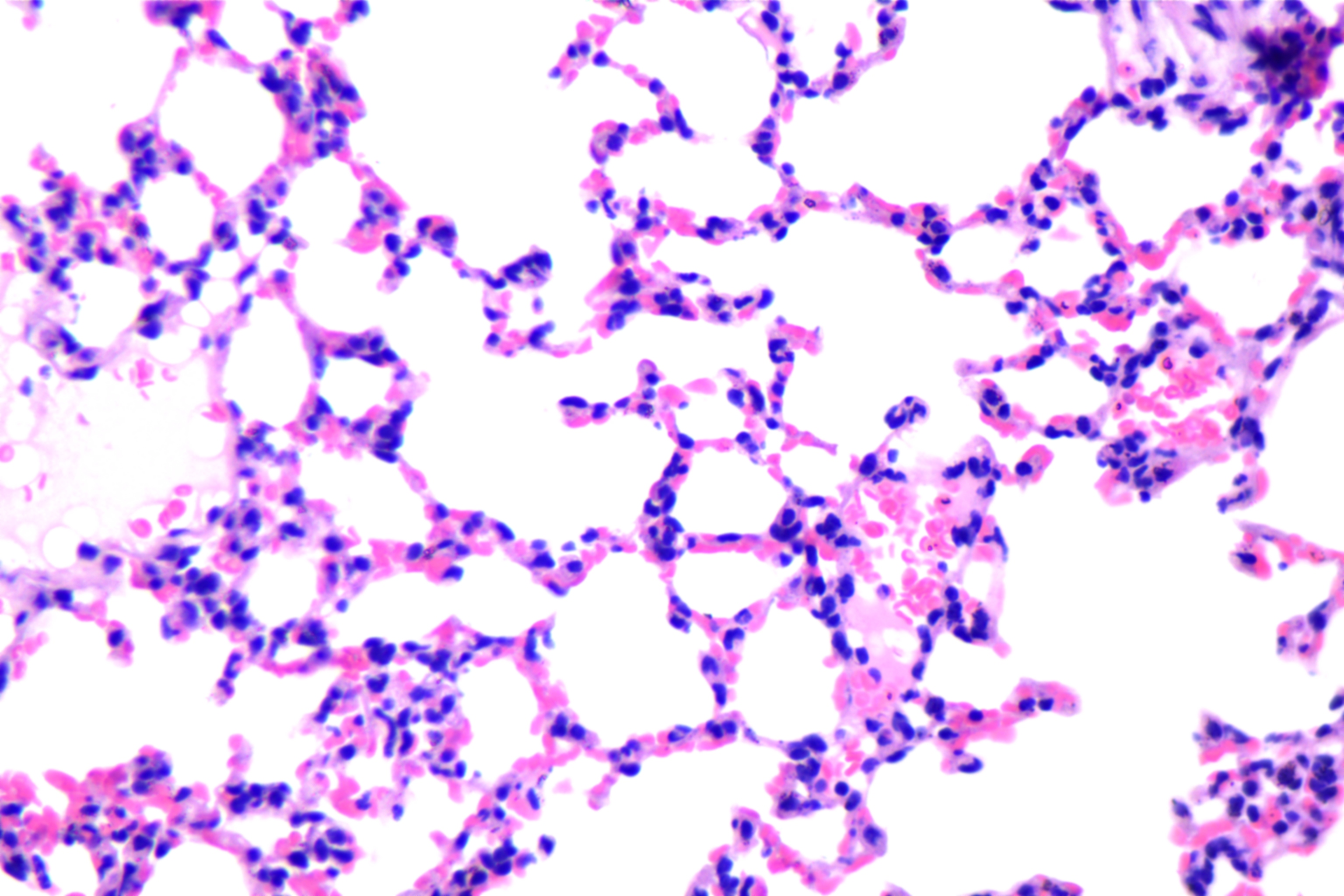

Supplement: Supplementary file 10 — Appendix Figure Source Data [file 44321_2025_308_MOESM10_ESM.zip › AF S5/5 A lung/PP10-2.5-lung 40X (3).jpg]

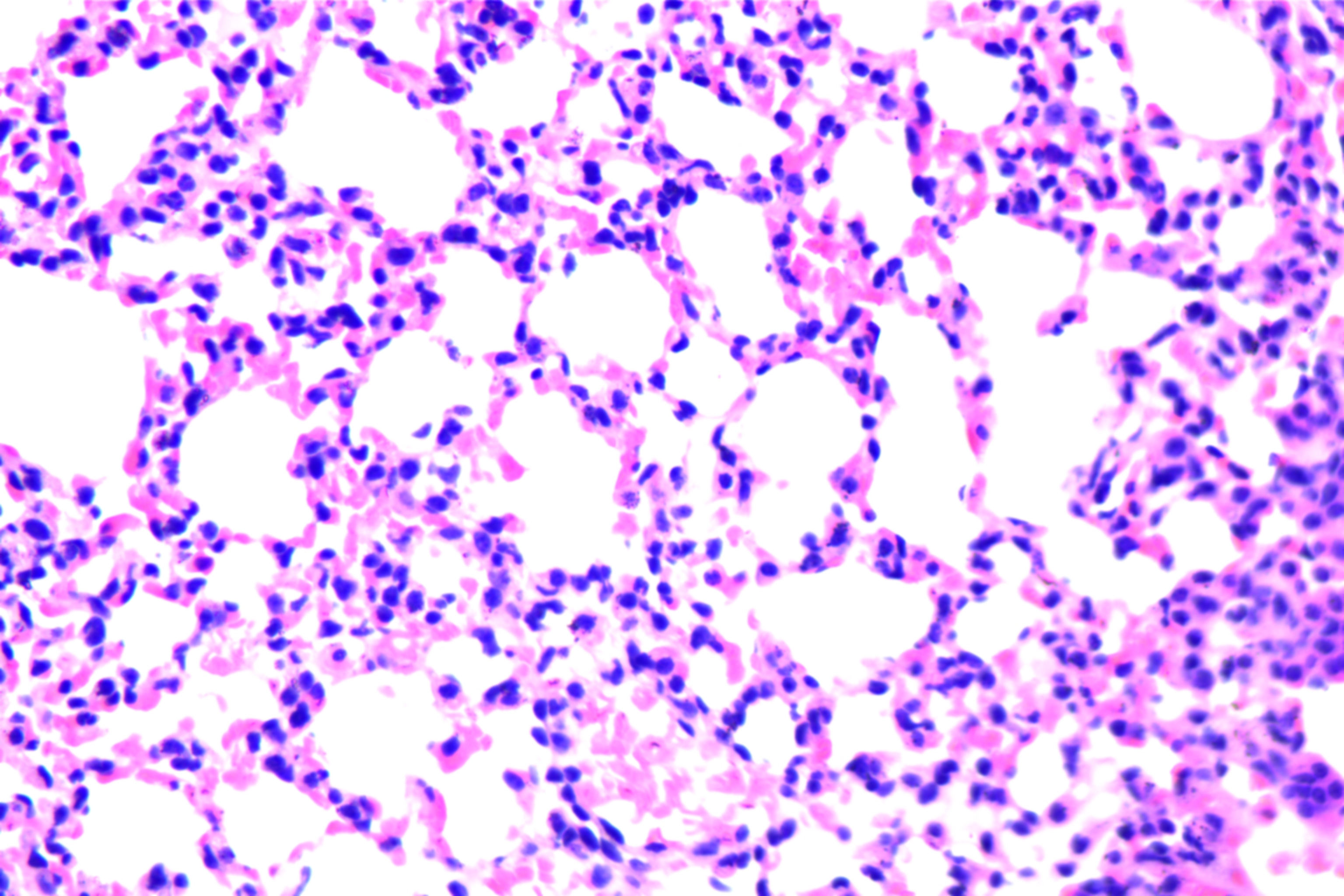

Supplement: Supplementary file 10 — Appendix Figure Source Data [file 44321_2025_308_MOESM10_ESM.zip › AF S5/5 A lung/PP10-2.5-lung 40X (4).jpg]

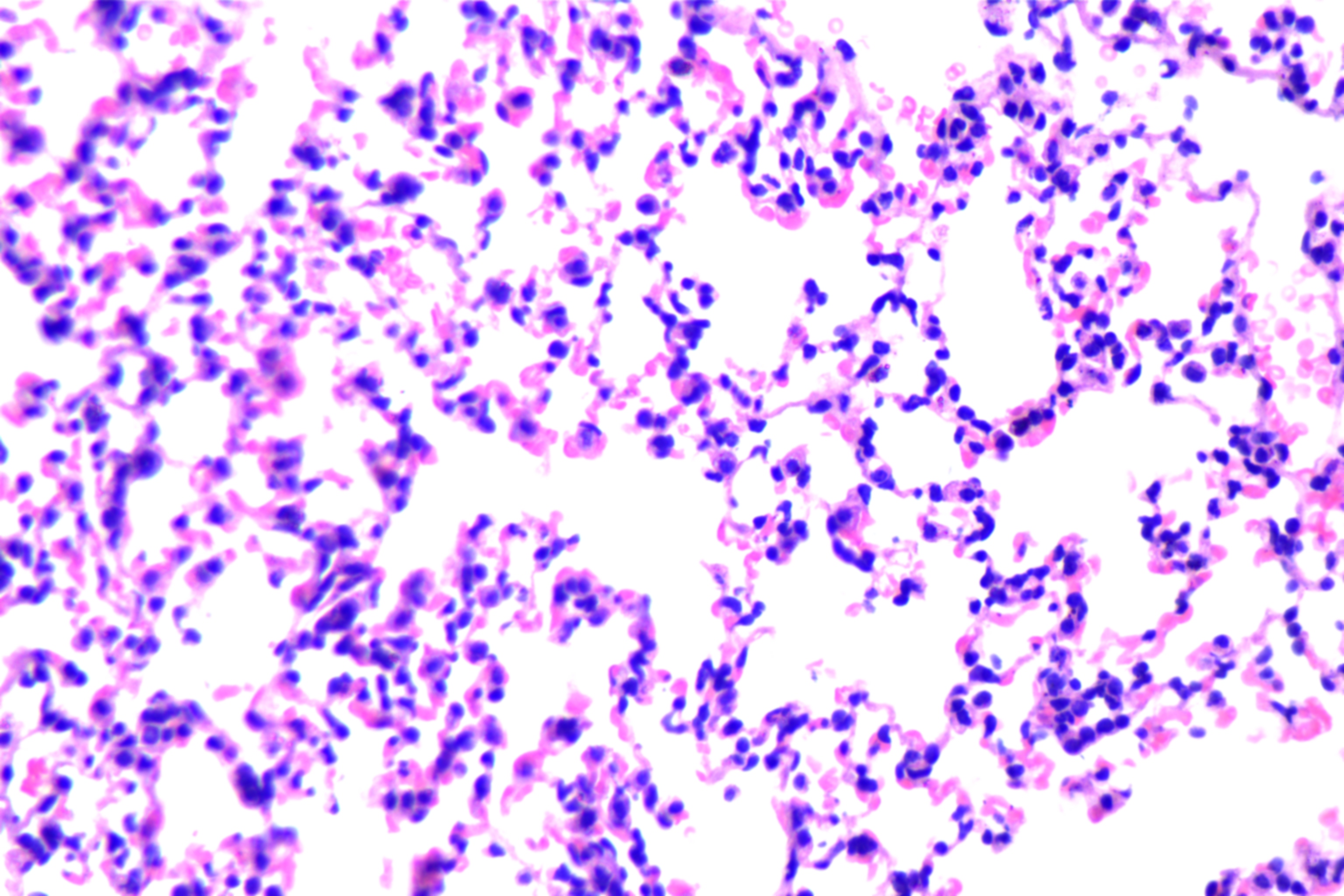

Supplement: Supplementary file 10 — Appendix Figure Source Data [file 44321_2025_308_MOESM10_ESM.zip › AF S5/5 A lung/PP10-5-lung 40X (1).jpg]

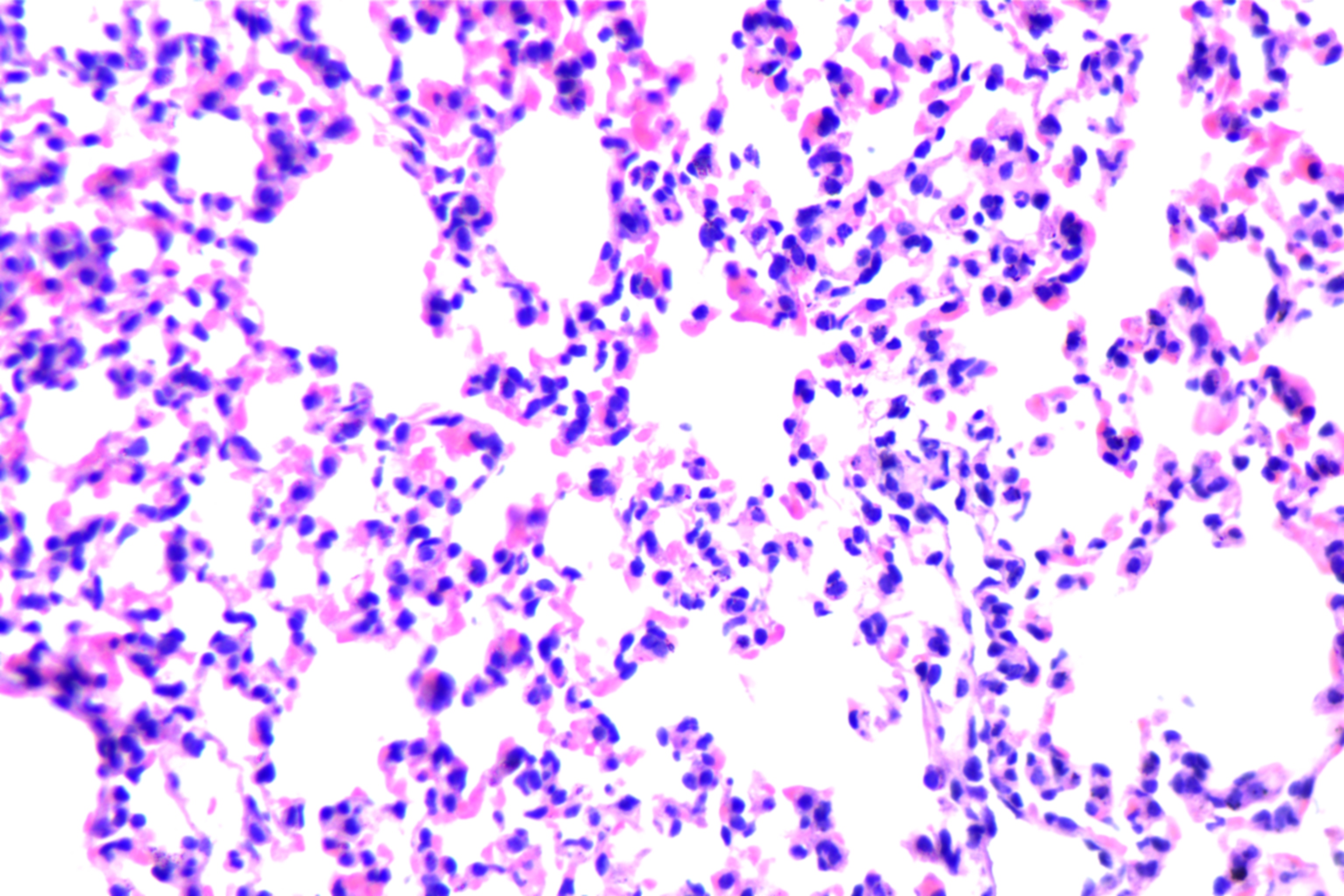

Supplement: Supplementary file 10 — Appendix Figure Source Data [file 44321_2025_308_MOESM10_ESM.zip › AF S5/5 A lung/PP10-5-lung 40X (2).jpg]

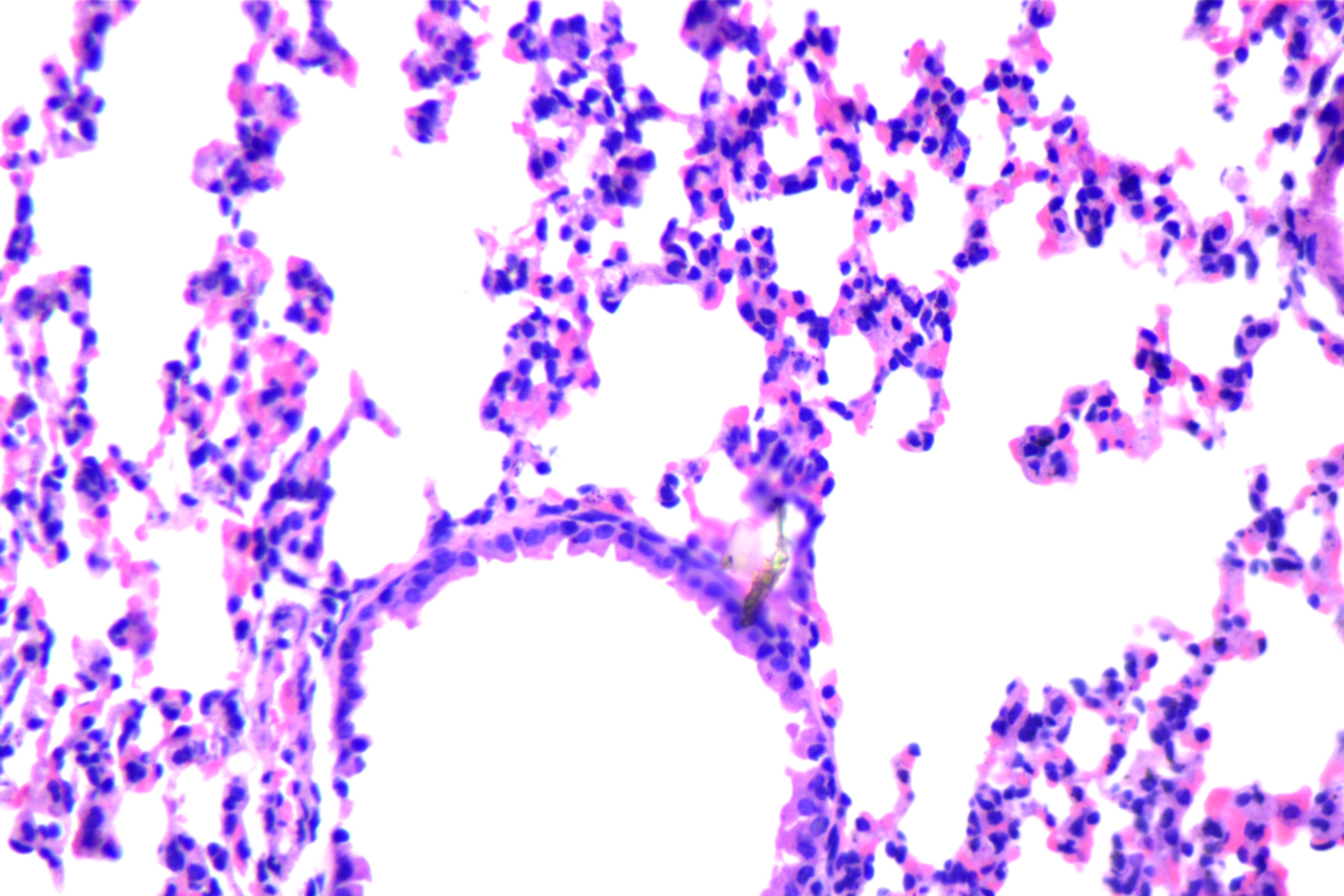

Supplement: Supplementary file 10 — Appendix Figure Source Data [file 44321_2025_308_MOESM10_ESM.zip › AF S5/5 A lung/PP10-5-lung 40X (3).jpg]

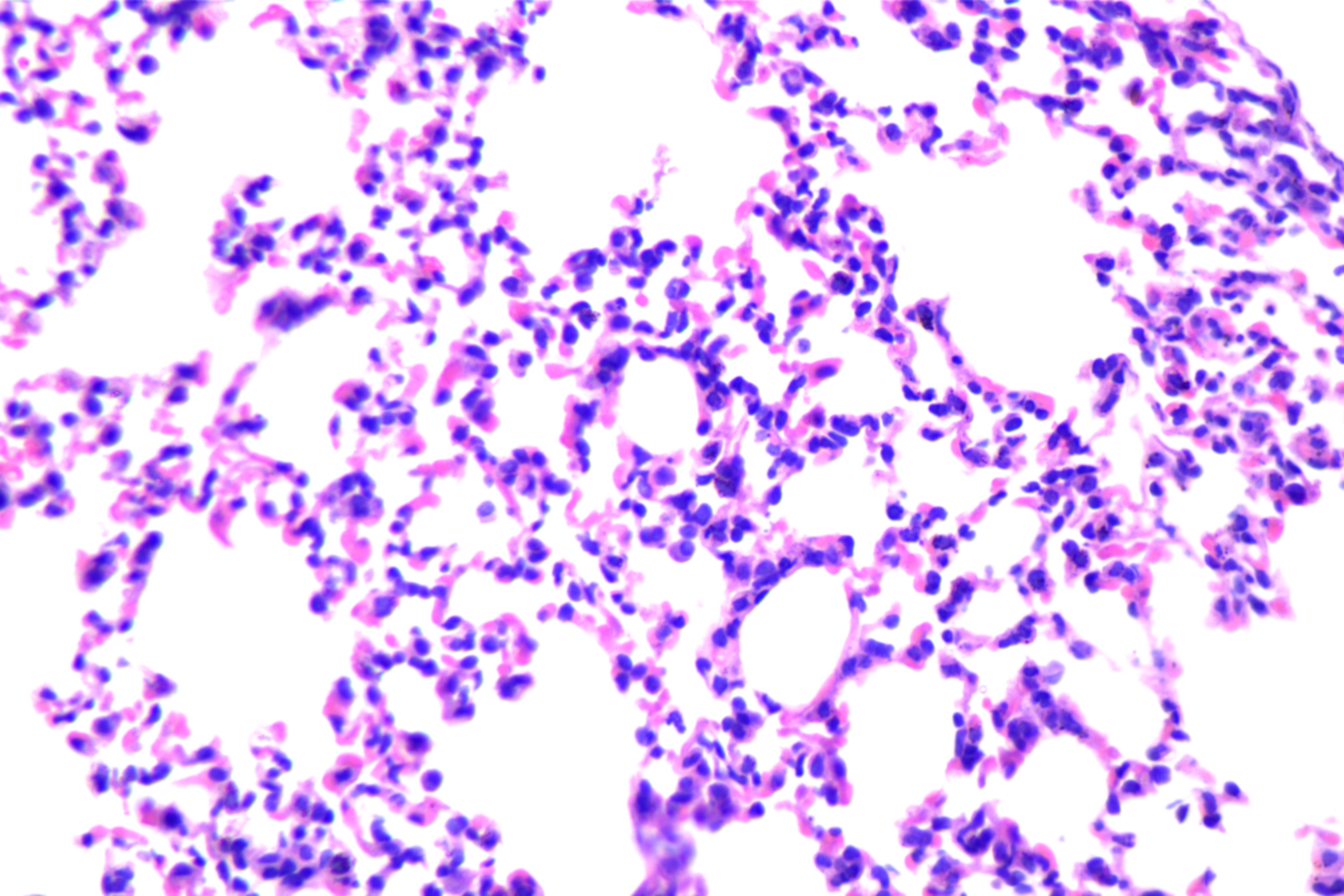

Supplement: Supplementary file 10 — Appendix Figure Source Data [file 44321_2025_308_MOESM10_ESM.zip › AF S5/5 A lung/PP10-5-lung 40X (4).jpg]

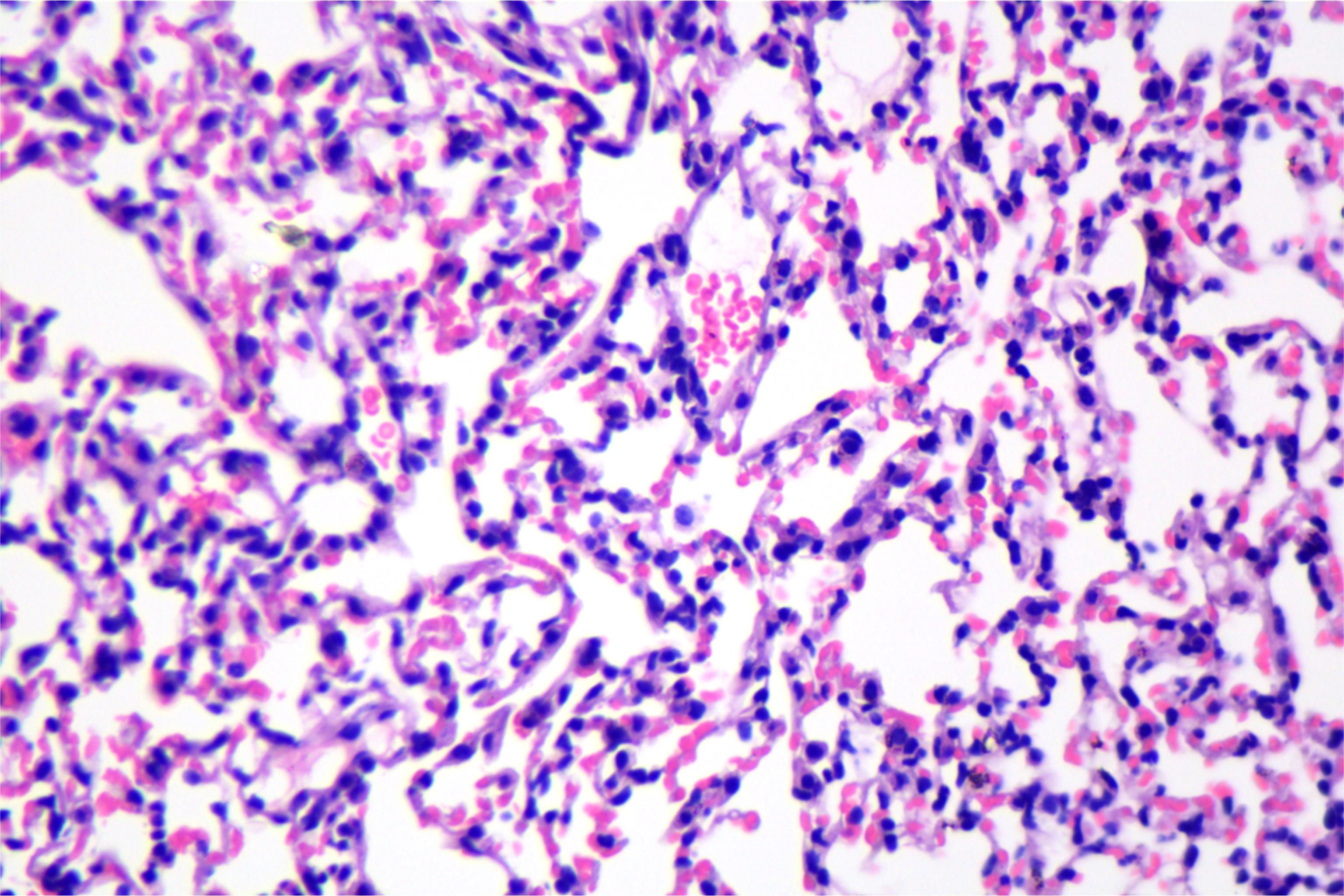

Supplement: Supplementary file 10 — Appendix Figure Source Data [file 44321_2025_308_MOESM10_ESM.zip › AF S5/5 A lung/PP24 2.5H-lung 40X (1).jpg]

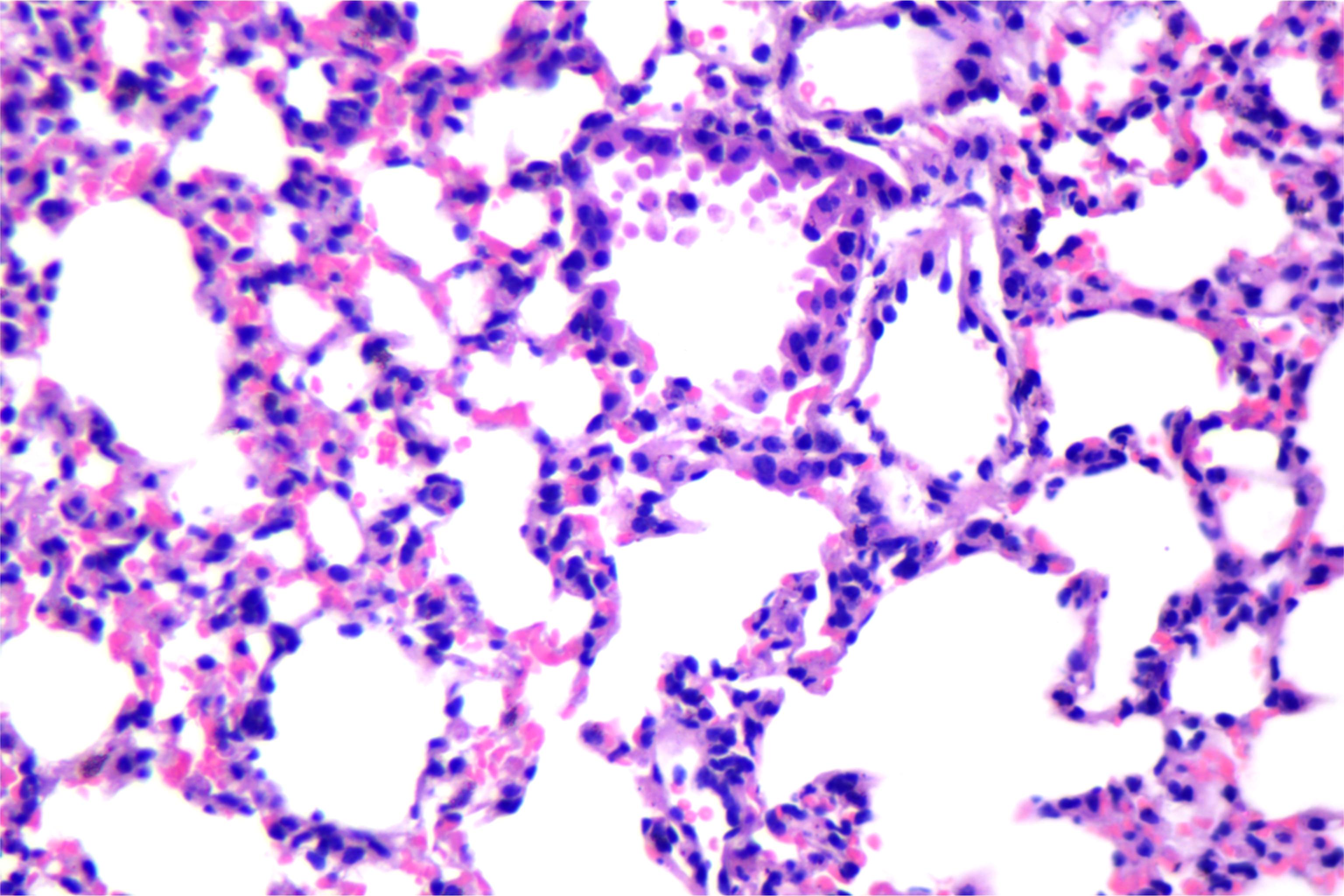

Supplement: Supplementary file 10 — Appendix Figure Source Data [file 44321_2025_308_MOESM10_ESM.zip › AF S5/5 A lung/PP24 2.5H-lung 40X (2).jpg]

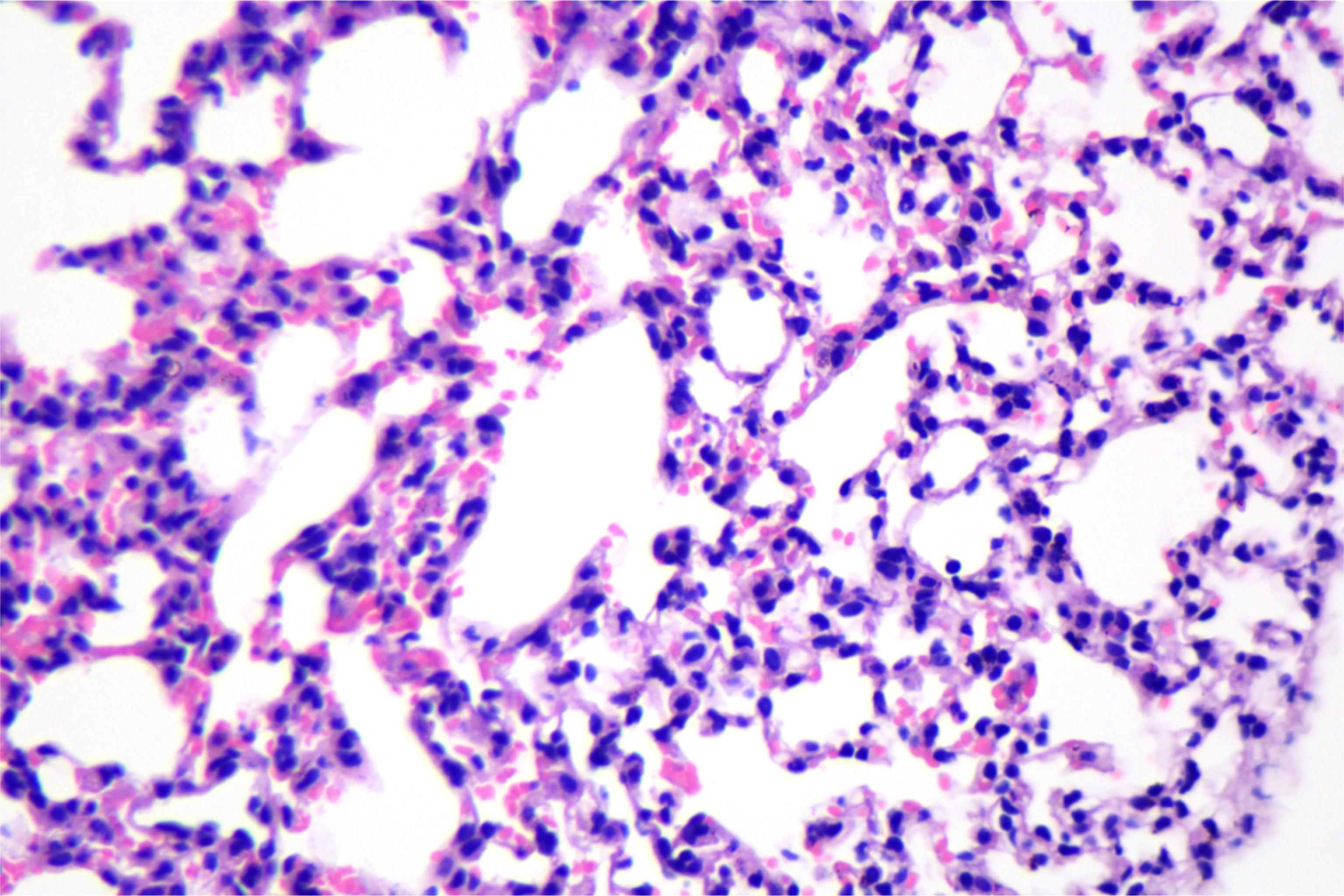

Supplement: Supplementary file 10 — Appendix Figure Source Data [file 44321_2025_308_MOESM10_ESM.zip › AF S5/5 A lung/PP24 2.5H-lung 40X (3).jpg]

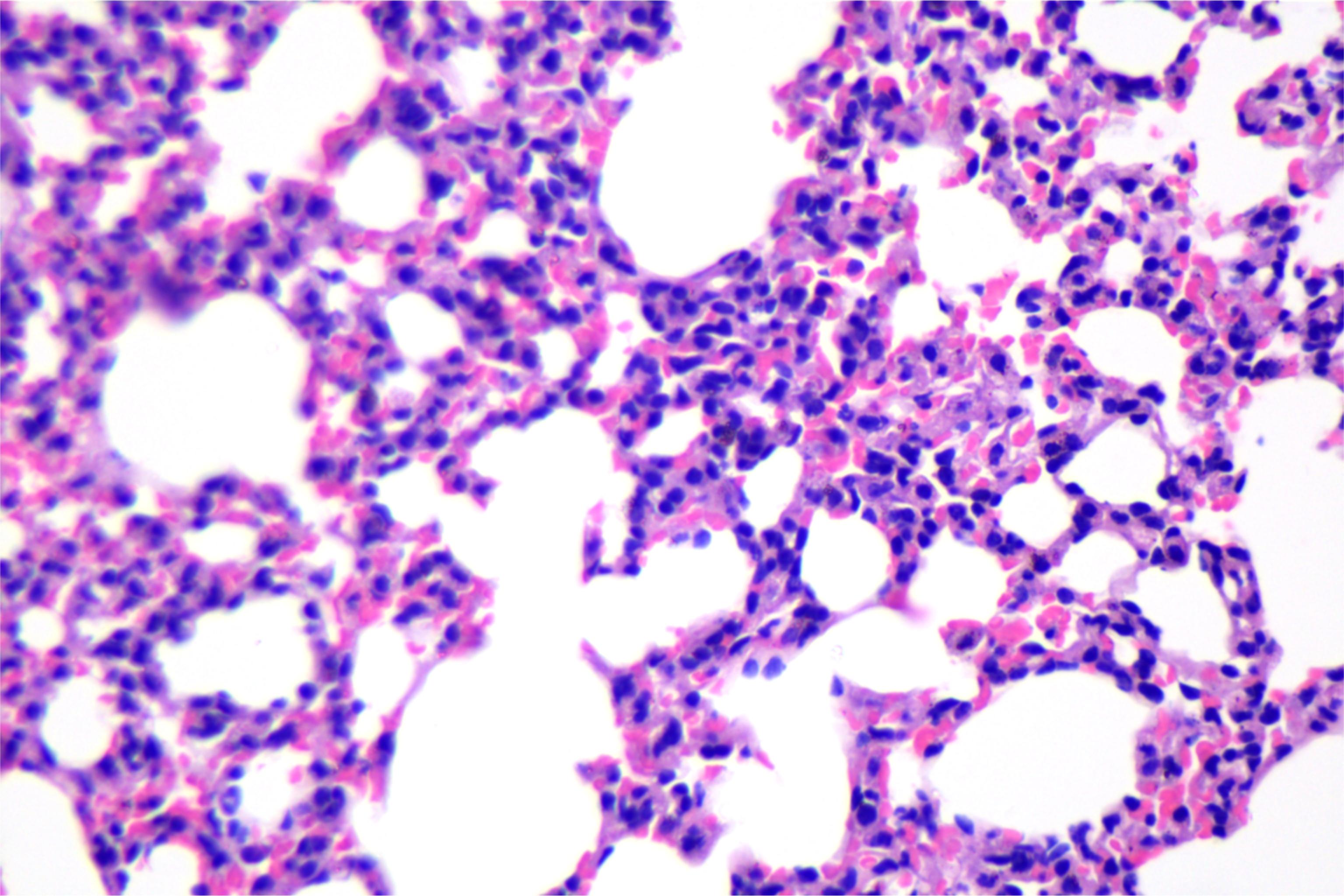

Supplement: Supplementary file 10 — Appendix Figure Source Data [file 44321_2025_308_MOESM10_ESM.zip › AF S5/5 A lung/PP24 2.5H-lung 40X (4).jpg]

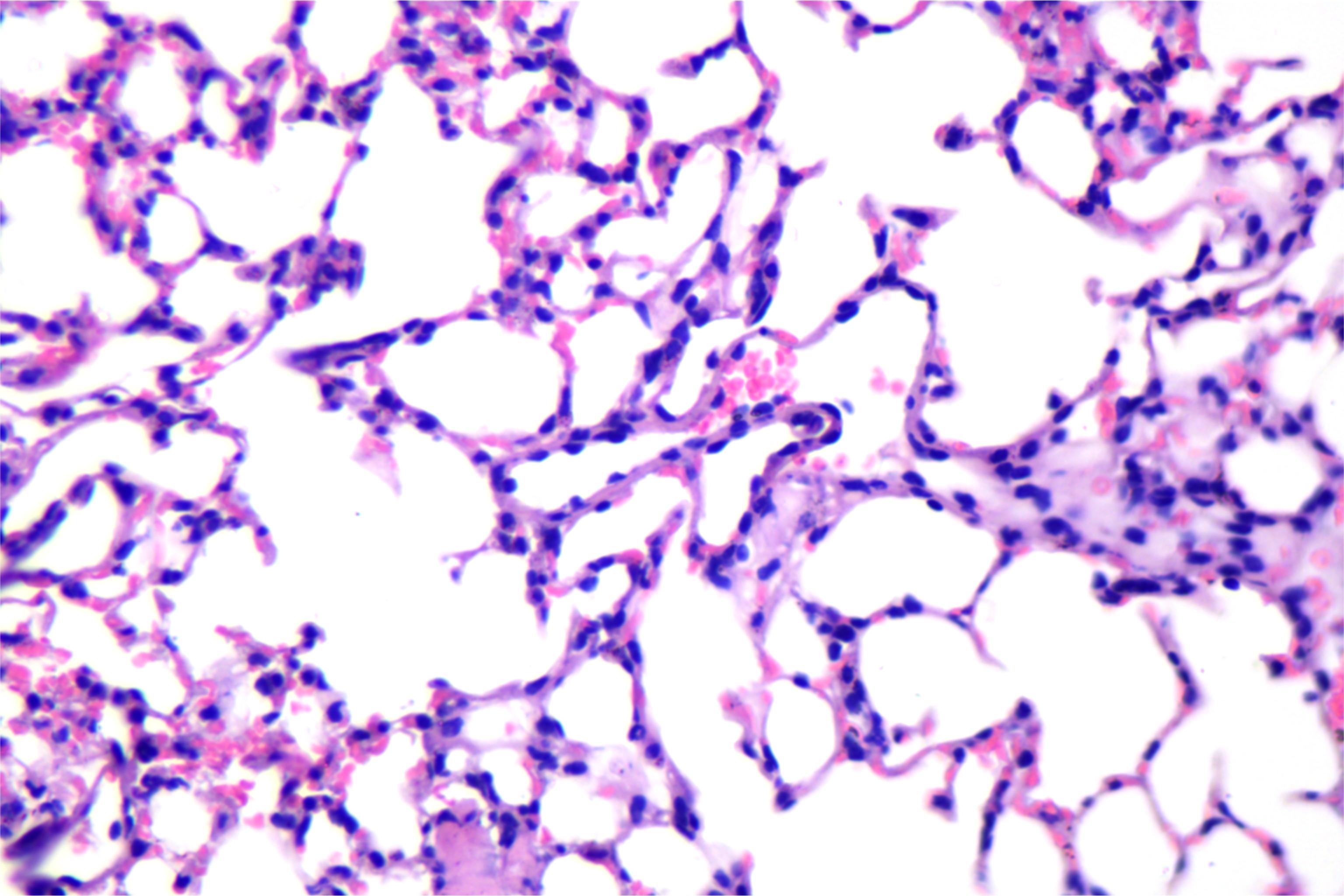

Supplement: Supplementary file 10 — Appendix Figure Source Data [file 44321_2025_308_MOESM10_ESM.zip › AF S5/5 A lung/PP24-5-lung 40X (1).jpg]

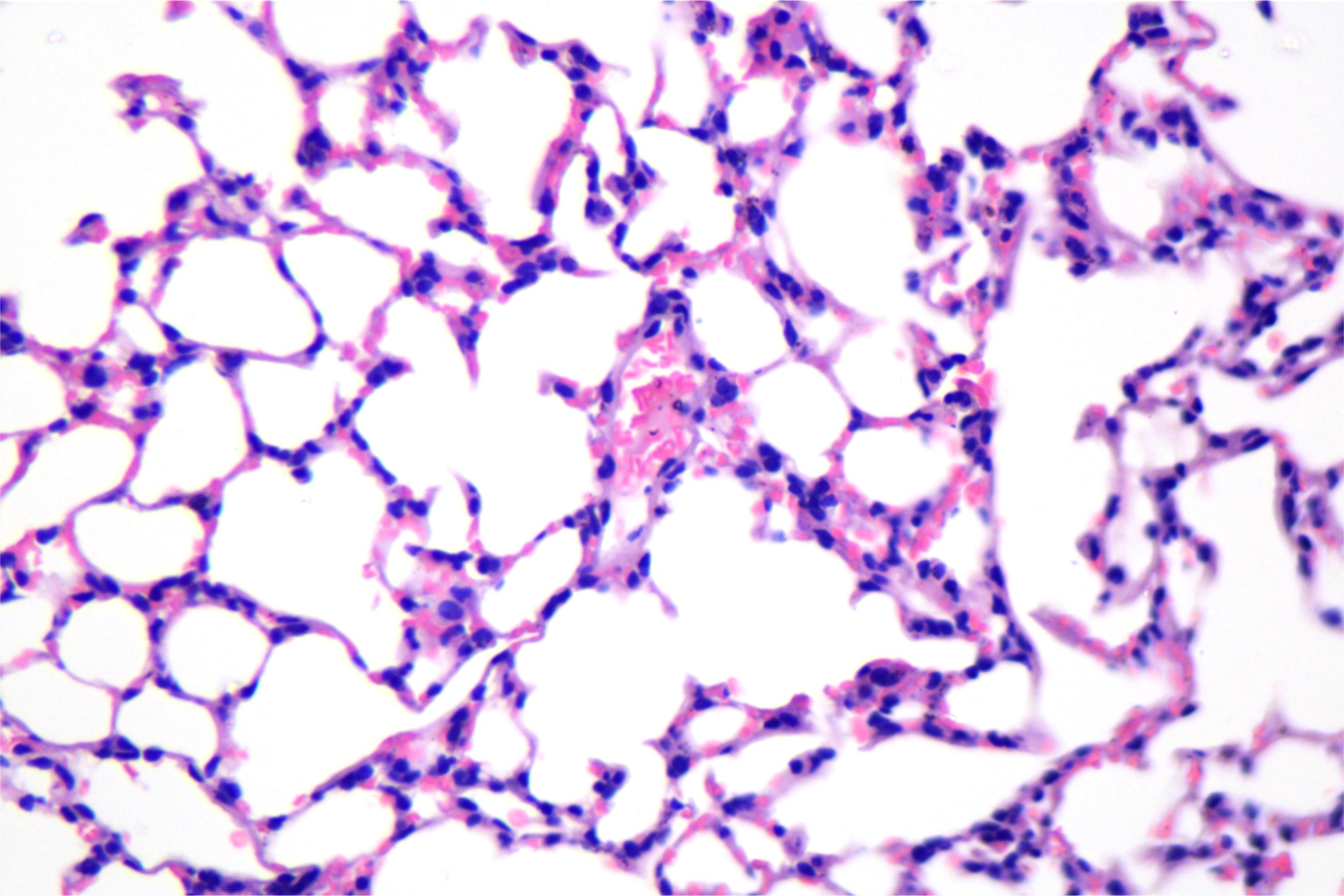

Supplement: Supplementary file 10 — Appendix Figure Source Data [file 44321_2025_308_MOESM10_ESM.zip › AF S5/5 A lung/PP24-5-lung 40X (2).jpg]

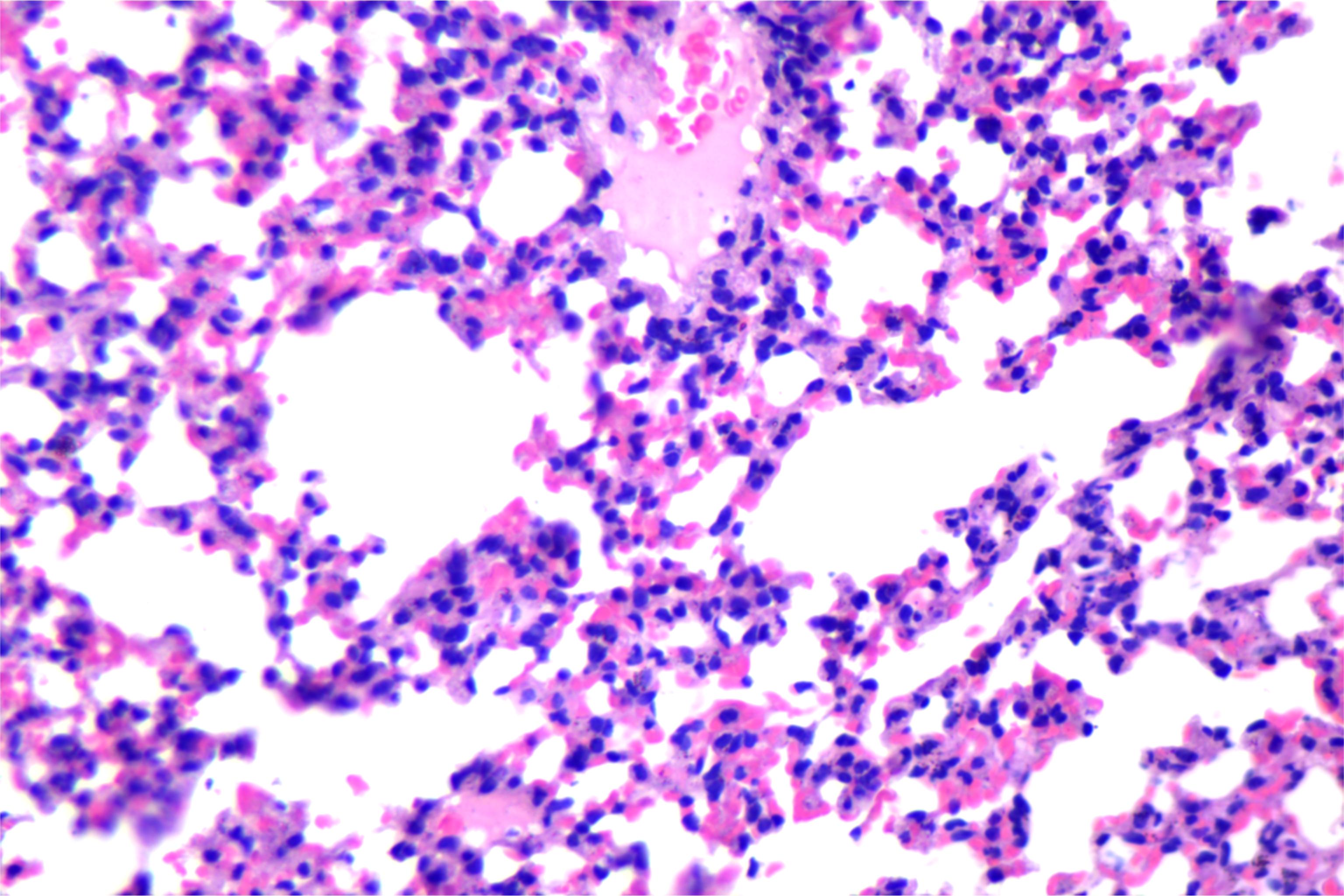

Supplement: Supplementary file 10 — Appendix Figure Source Data [file 44321_2025_308_MOESM10_ESM.zip › AF S5/5 A lung/PP24-5-lung 40X (3).jpg]

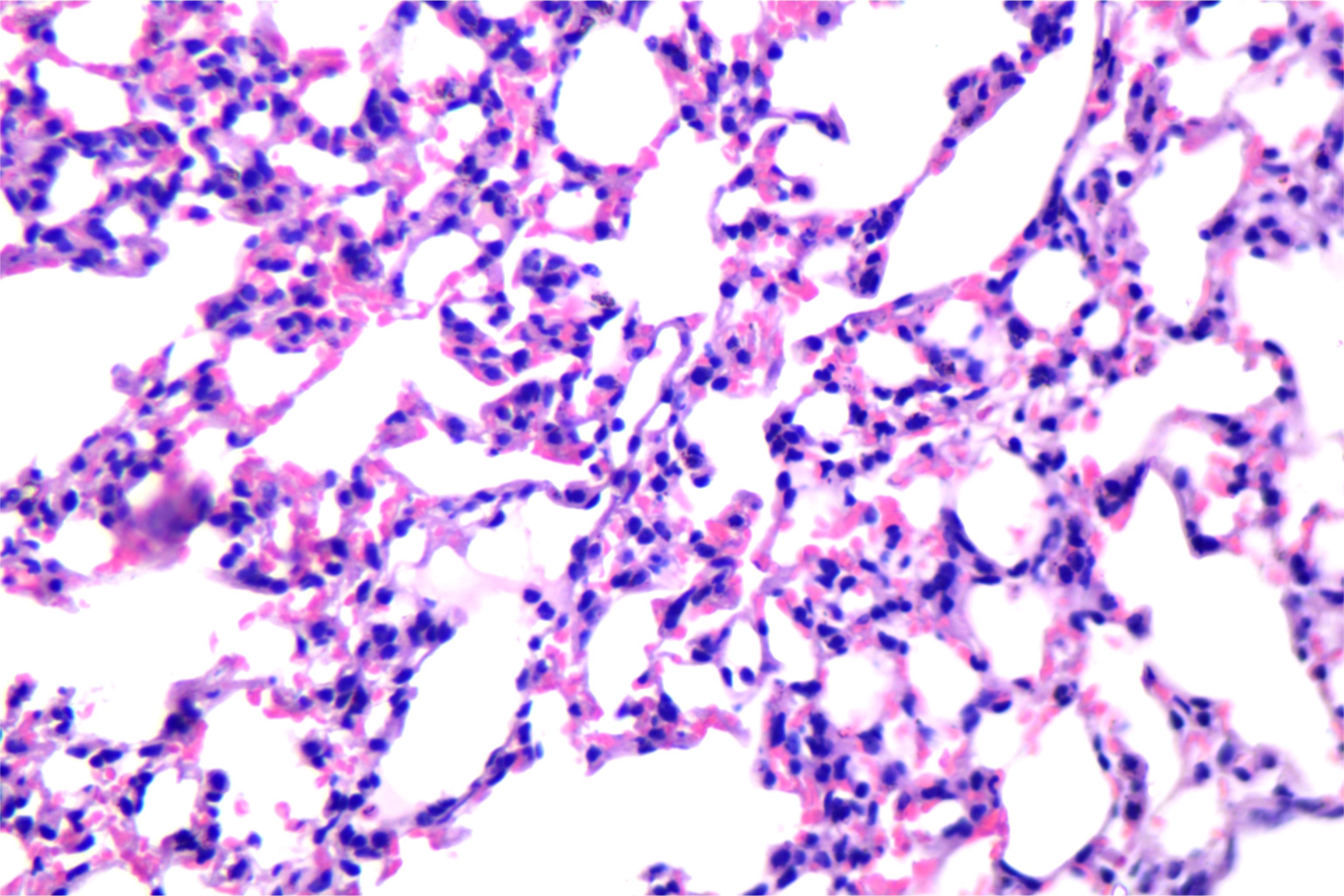

Supplement: Supplementary file 10 — Appendix Figure Source Data [file 44321_2025_308_MOESM10_ESM.zip › AF S5/5 A lung/PP24-5-lung 40X (4).jpg]
