# Supplementary material for: AI-identified CD133-targeting natural compounds demonstrate differential anti-tumor effects and mechanisms in pan-cancer models
Source: EMBO Mol Med. 2025 Oct 2;17(11):2932–65. doi: 10.1038/s44321-025-00308-1 (PMC12603267; doi:10.1038/s44321-025-00308-1)
Supplement: Supplementary file 11 — Expanded View Figures [file 44321_2025_308_MOESM11_ESM.pdf]

Expanded View Figures

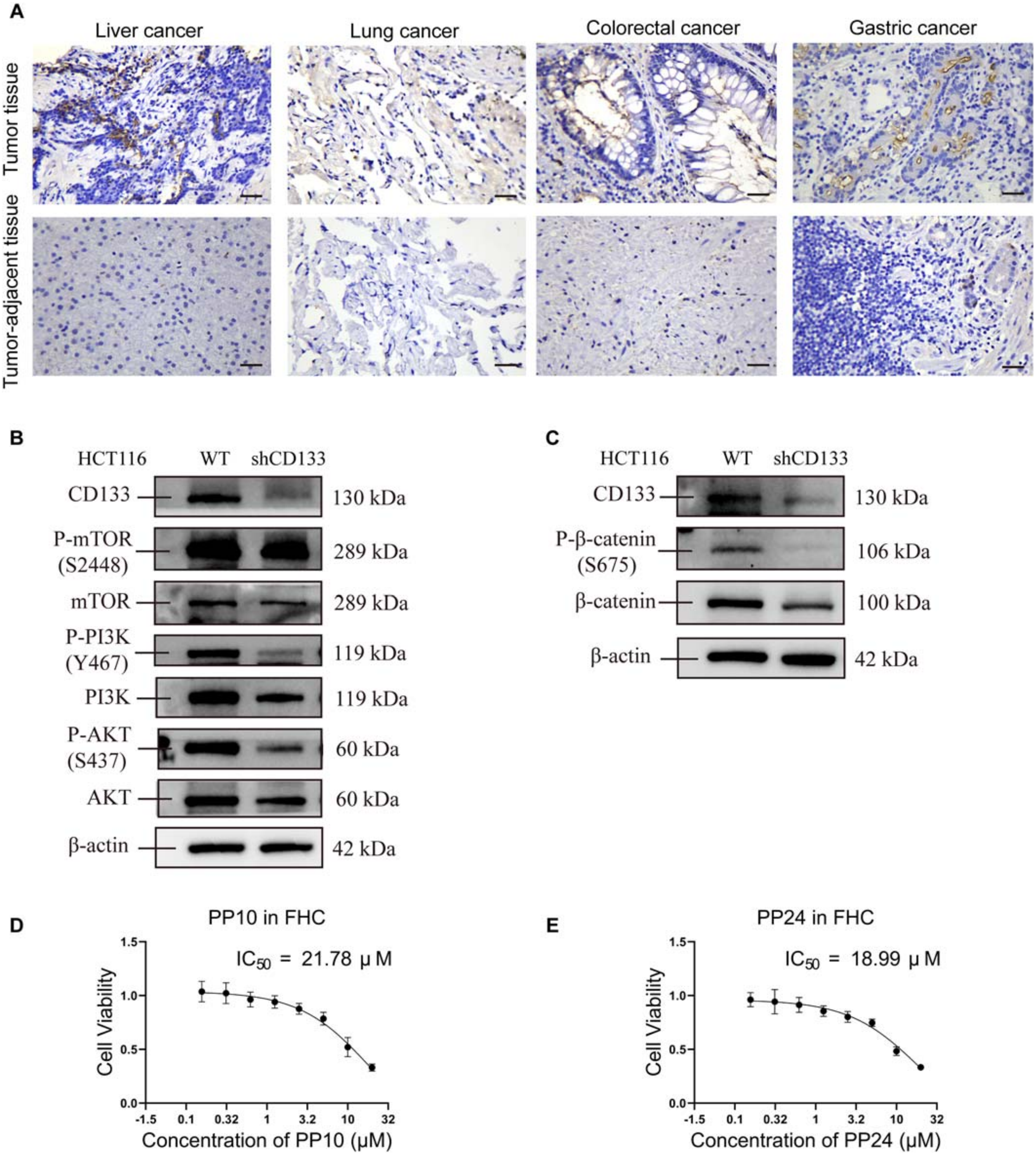

Figure EV1. Expression level of CD133 in various cancer and cytotoxicity of PP10 and PP24 on normal cell lines.

(**A**) The IHC staining of CD133 in various cancer, including liver cancer, lung cancer, colorectal cancer, and gastric cancer. Scale bar, 50 µm. (**B**, **C**) Expression level of signaling pathway in WT and shCD133 HCT116 cell lines. (**B**) PI3K–AKT pathway, (**C**) Wnt-β-catenin pathway (**D**, **E**) Cell viability assay results showing the cytotoxicity of PP10 and PP24 in FHC cells. Data are presented as mean ± SD. *$P < 0.05$, **$P < 0.01$, and ***$P < 0.001$ when compared with control group.

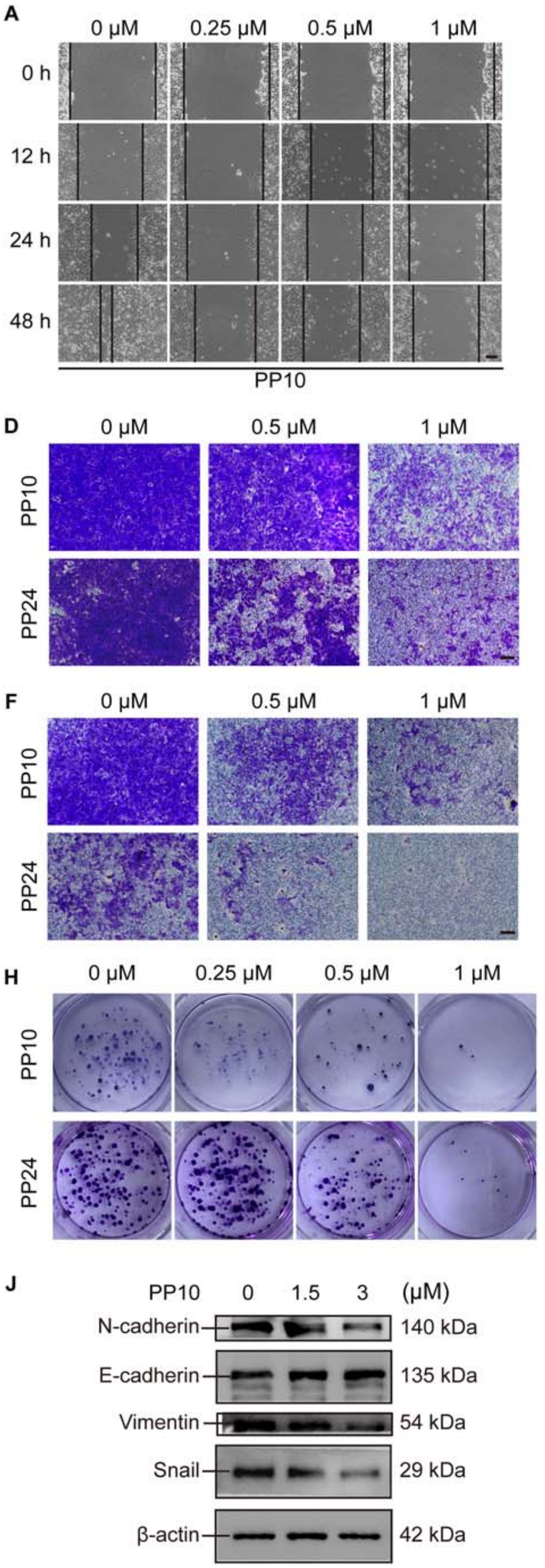
A
0 μM
0.25 μM
0.5 μM
1 μM
0 h
12 h
24 h
48 h
PP10
B
PP24
D
PP10
PP24
C
Confluence (%)
Concentration of PP10 (μM)
Concentration of PP24 (μM)
E
Number of migration cells
F
G
Number of invasion cells
H
I
Count of colony
J
PP10
0
1.5
3
(μM)
N-cadherin
140 kDa
E-cadherin
135 kDa
Vimentin
54 kDa
Snail
29 kDa
β-actin
42 kDa
K
PP24

Figure EV2. PP10 and PP24 inhibit migration, invasion, and colony formation of CRC cells in vitro.

(**A**, **B**) PP10 and PP24 inhibit the wound healing ability of HCT116 cells. (**C**) Statistics of wound healing confluence in (**A**, **B**) ($N = 3$). (**D**) PP10 and PP24 inhibit the migration ability of HCT116 cells assessed by Transwell assay. (**E**) Statistics of migrated cell counts in (**D**) ($N = 3$). (**F**) PP10 and PP24 suppress the metastasis potential of HCT116 cells in Transwell assays. (**G**) Statistics of migrated cell counts in (**F**) ($N = 3$). (**H**) PP10 and PP24 reduce colony formation ability of HCT116 cells. (**I**) Statistics of colony numbers formed in (**H**) ($N = 3$). Western blot analysis of EMT pathway related proteins after treating with PP10 (**J**) and PP24 (**K**). Scale bar, 50 µm. one-way ANOVA test. Exact *P* value are presented in Appendix Table S2. Data are presented as mean ± SD. $^{*}P < 0.05$, $^{**}P < 0.01$, and $^{***}P < 0.001$ when compared with control group.

© The Author(s)

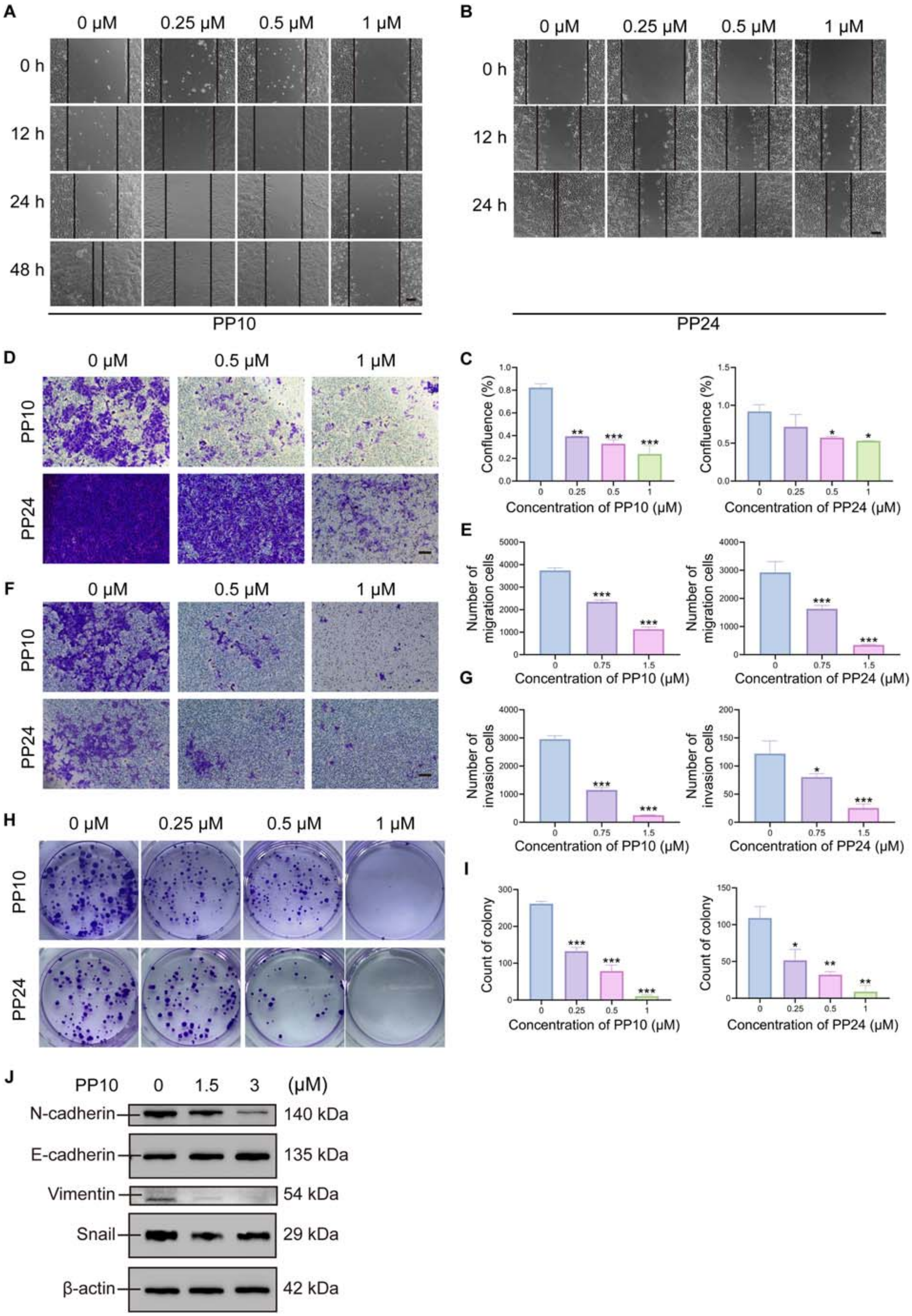
A
0 μM
0.25 μM
0.5 μM
1 μM
0 h
12 h
24 h
48 h
PP10
B
0 μM
0.25 μM
0.5 μM
1 μM
0 h
12 h
24 h
PP24
D
0 μM
0.5 μM
1 μM
PP10
PP24
C
Confluence (%)
Concentration of PP10 (μM)
Concentration of PP24 (μM)
E
Number of migration cells
Concentration of PP10 (μM)
Concentration of PP24 (μM)
F
0 μM
0.5 μM
1 μM
PP10
PP24
G
Number of invasion cells
Concentration of PP10 (μM)
Concentration of PP24 (μM)
H
0 μM
0.25 μM
0.5 μM
1 μM
PP10
PP24
I
Count of colony
Concentration of PP10 (μM)
Concentration of PP24 (μM)
J
PP10 0 1.5 3 (μM)
N-cadherin 140 kDa
E-cadherin 135 kDa
Vimentin 54 kDa
Snail 29 kDa
β-actin 42 kDa

 © The Author(s)

Figure EV3. PP10 and PP24 inhibit migration, invasion, and colony formation of CRC cells in vitro.

(**A**, **B**) PP10 and PP24 inhibit the wound healing ability of DLD1 cells. (**C**) Statistics of wound healing confluence in (**A**, **B**) ($N = 3$). (**D**) PP10 and PP24 inhibit the migration ability of DLD1 cells assessed by Transwell assay. (**E**) Statistics of migrated cell counts in (**D**) ($N = 3$). (**F**) PP10 and PP24 suppress the metastasis potential of DLD1 cells in Transwell assays. (**G**) Statistics of migrated cell counts in (**F**) ($N = 3$). (**H**) PP10 and PP24 reduce colony formation ability of DLD1 cells. (**I**) Statistics of colony numbers formed in (**H**) ($N = 3$). Western blot analysis of EMT pathway related proteins after treating with PP10 (**J**). Scale bar, 50 µm. one-way ANOVA test. Exact *P* value are presented in Appendix Table S2. Data are presented as mean ± SD. $*P < 0.05$, $**P < 0.01$, and $***P < 0.001$, when compared with control group.

© The Author(s)

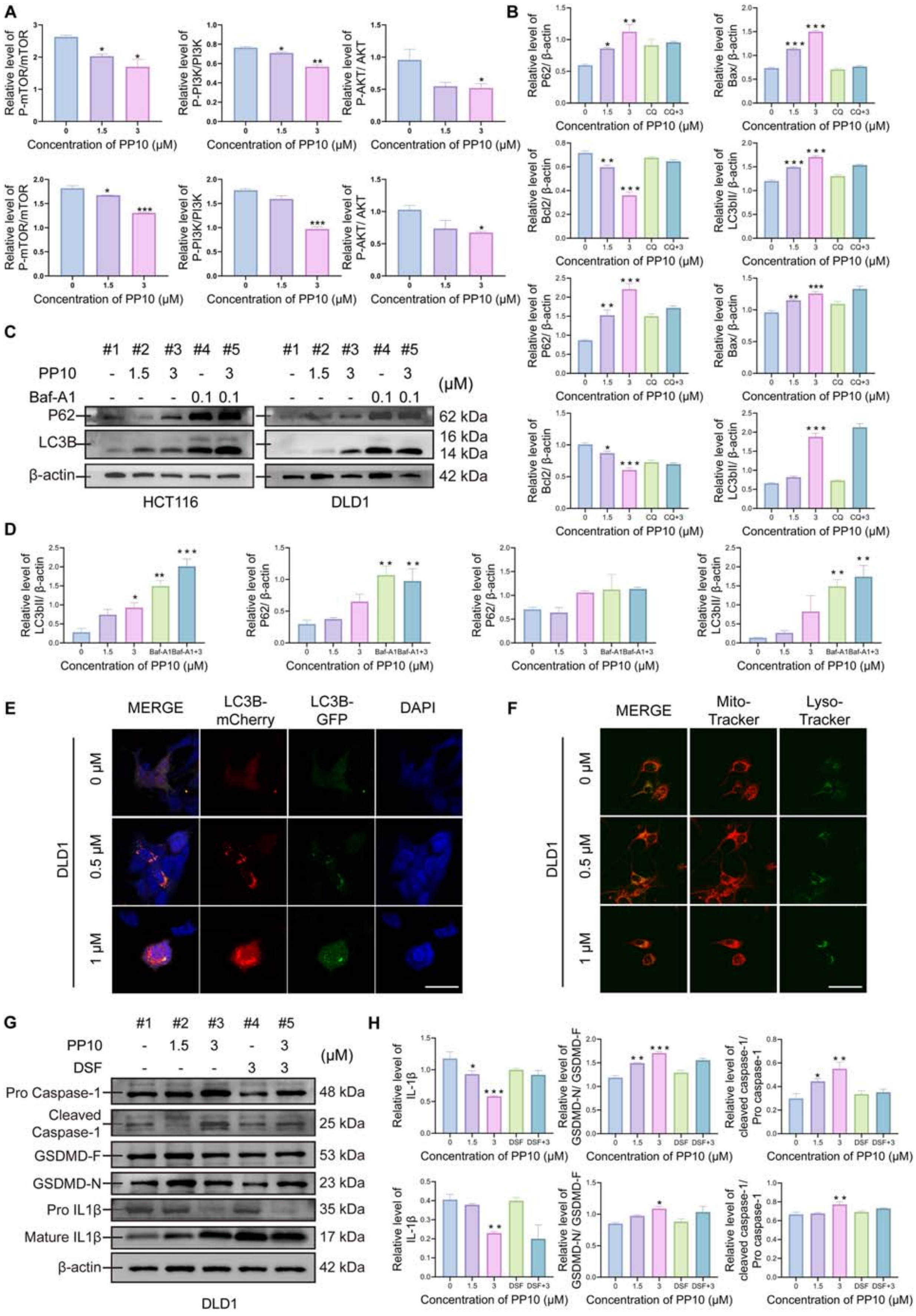
A
Relative level of P-mTOR/mTOR
Relative level of P-PI3K/PI3K
Relative level of P-AKT/ AKT
Concentration of PP10 (μM)
B
Relative level of P62/ β-actin
Relative level of Bax/ β-actin
Relative level of Bcl2/ β-actin
Relative level of LC3bII/ β-actin
C
#1 #2 #3 #4 #5
PP10 - 1.5 3 - 3
Baf-A1 - - - 0.1 0.1
(μM)
P62
LC3B
β-actin
62 kDa
16 kDa
14 kDa
42 kDa
HCT116
DLD1
D
E
MERGE
LC3B-mCherry
LC3B-GFP
DAPI
DLD1
0 μM
0.5 μM
1 μM
F
MERGE
Mito-Tracker
Lyso-Tracker
G
PP10 - 1.5 3 - 3
DSF - - - 3 3
Pro Caspase-1
Cleaved Caspase-1
GSDMD-F
GSDMD-N
Pro IL1β
Mature IL1β
48 kDa
25 kDa
53 kDa
23 kDa
35 kDa
17 kDa
H
Relative level of IL-1β
Relative level of GSDMD-N/ GSDMD-F
Relative level of cleaved caspase-1/ Pro caspase-1

Figure EV4. Statistical analysis of protein expression level.

(**A**) Quantification of expression levels normalized to β-actin in Fig. 4H, I ($N = 3$). (**B**) Quantification of relative expression levels for the mitophagy pathway to β-actin in Fig. 5C,D ($N = 3$). (**C**) Western blot analysis of mitophagy-related proteins in HCT116 and DLD1 cells treated with PP10 and Baf-A1. (**D**) Quantification of relative expression levels for the mitophagy pathway to β-actin in (**D**) ($N = 3$). (**E**) Microscopic observation of mCherry-GFP-LC3B infected DLD1 cells, showing autophagosome-lysosome fusion following PP10 treatment. Scale bar, 50 μm. (**F**) Microscopic observation of mitochondria and lysosome co-localization in DLD1 cells using Mito/Lyso-Tracker probes after PP10 treatment. Scale bar, 50 μm. (**G**) Western blot analysis of pyroptosis-related proteins in DLD1 cells treated with PP10. (**H**) Quantification of relative expression levels for pyroptosis pathway to β-actin in Fig. 5I, Fig. EV4G ($N = 3$). one-way ANOVA test. Exact *P* value are presented in Appendix Table S2. Data are presented as mean ± SD. $^*P < 0.05$, $^{**}P < 0.01$, and $^{***}P < 0.001$, when compared with control group.

© The Author(s)

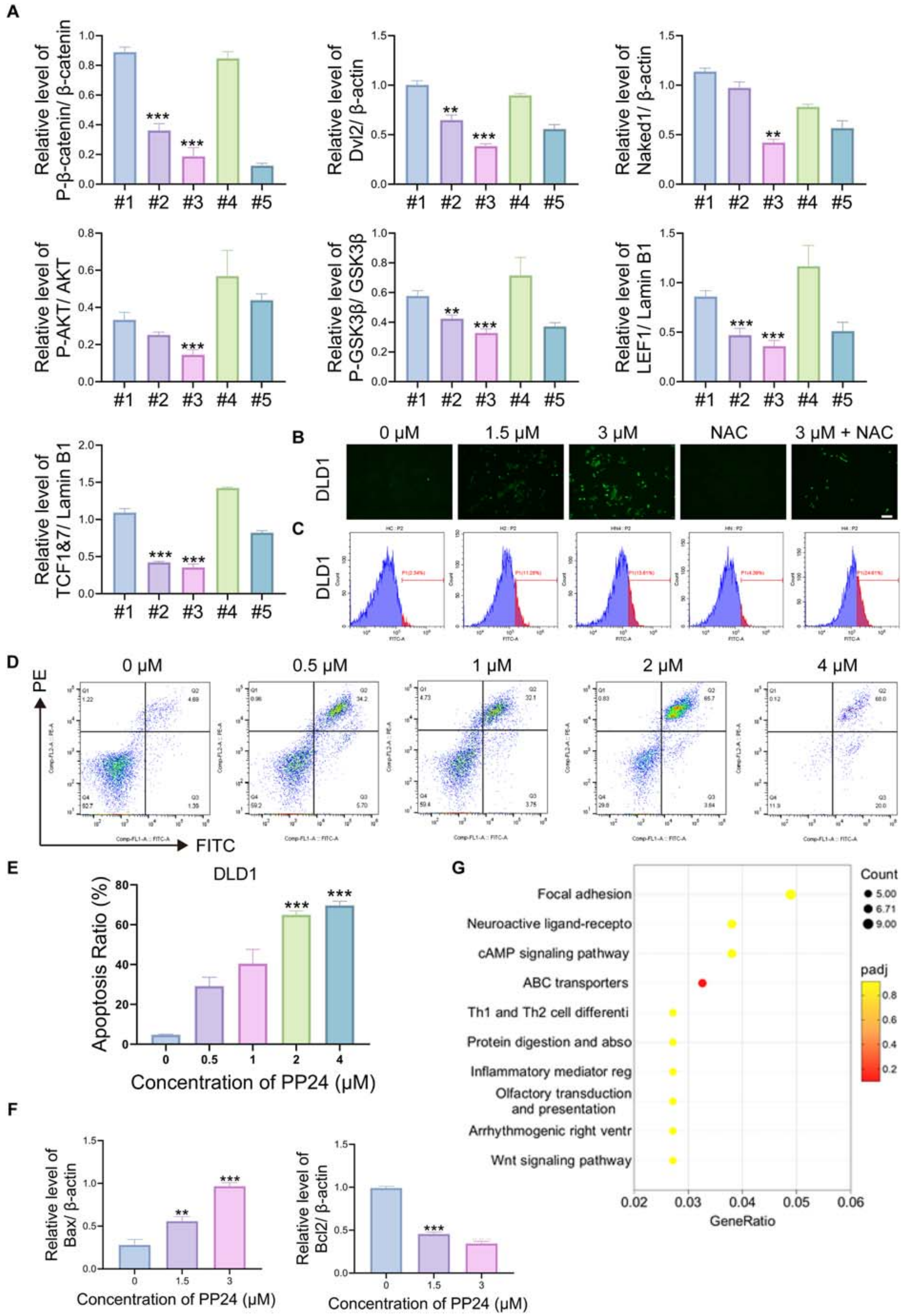
A
Relative level of P-β-catenin/ β-catenin
Relative level of Dvl2/ β-actin
Relative level of Naked1/ β-actin
Relative level of P-AKT/ AKT
Relative level of P-GSK3β/ GSK3β
Relative level of LEF1/ Lamin B1
Relative level of TCF1&7/ Lamin B1
#1 #2 #3 #4 #5
B
DLD1
0 μM
1.5 μM
3 μM
NAC
3 μM + NAC
C
D
PE
FITC
0 μM
0.5 μM
1 μM
2 μM
4 μM
E
DLD1
Apoptosis Ratio (%)
Concentration of PP24 (μM)
F
Relative level of Bax/ β-actin
Relative level of Bcl2/ β-actin
Concentration of PP24 (μM)
G
Focal adhesion
Neuroactive ligand-recepto
cAMP signaling pathway
ABC transporters
Th1 and Th2 cell differenti
Protein digestion and abso
Inflammatory mediator reg
Olfactory transduction and presentation
Arrhythmogenic right ventr
Wnt signaling pathway
GeneRatio
Count
padj

Figure EV5. Statistical analysis of protein expression level and detection of ROS.

(**A**) Quantification of relative expression levels to β-actin and Lamin B1 ($N = 3$) in Fig. 6C. (**B**) Microscopic observation of ROS levels in DLD1 treated with PP24 and/or NAC using DCFH-DA probe. (**C**) Flow cytometry results showing ROS detection after treatment with PP24 and NAC in DLD1 cells. (**D**, **E**) Detection of apoptosis of DLD1 cells after treatment of PP24 by flow cytometry. (**F**) Quantification of relative expression levels to β-actin in Fig. 6F ($N = 3$). (**G**) KEGG pathway enrichment analysis showing the functional pathways affected by DEGs in DLD1 cells following PP24 treatment. One-way ANOVA test. Exact *P* value are presented in Appendix Table S2. Data are presented as mean ± SD. $^{*}P < 0.05$, $^{**}P < 0.01$, and $^{***}P < 0.001$, when compared with control group.

© The Author(s)

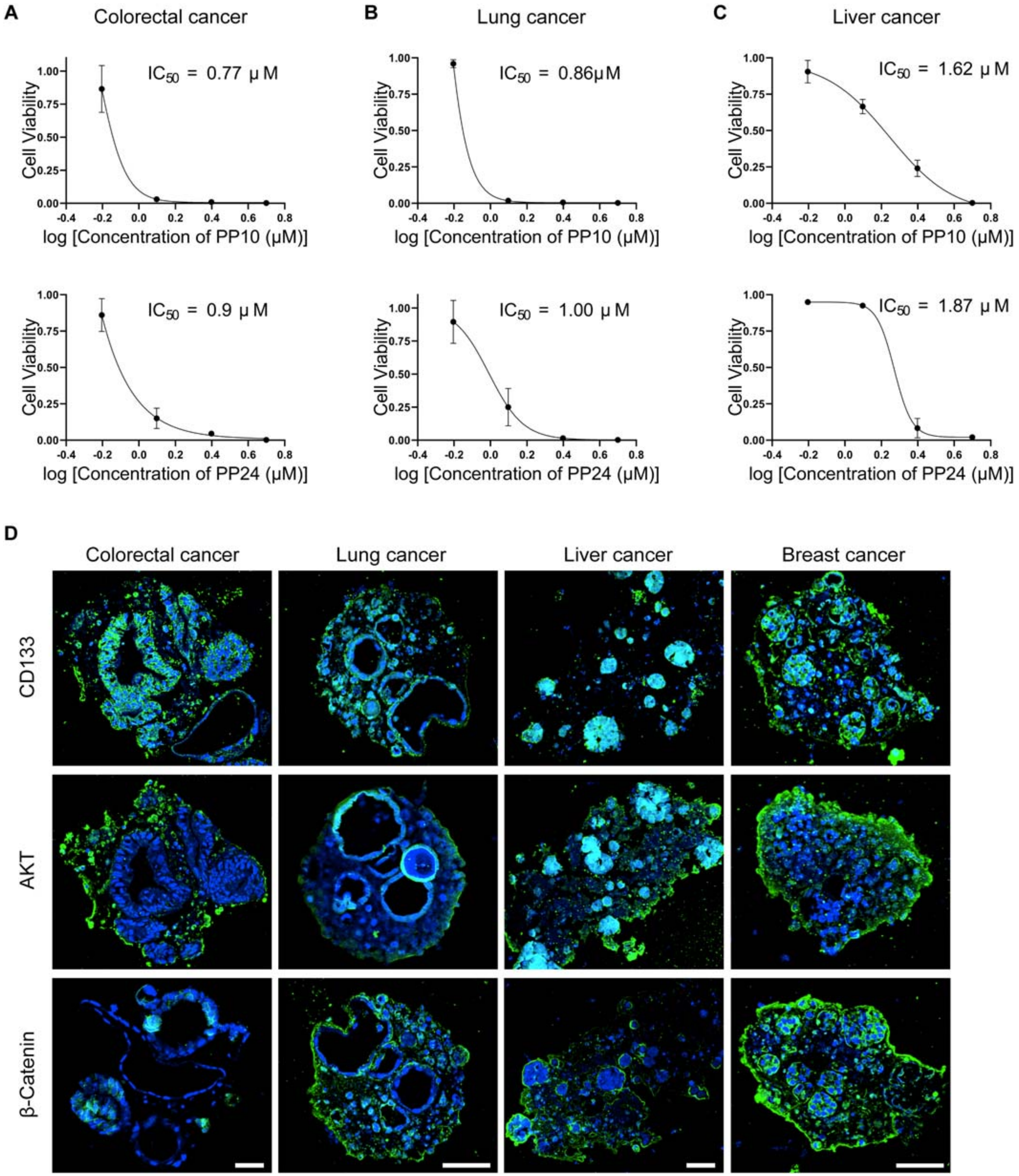

Figure EV6. PP10 and PP24 inhibit viability of pan-cancer organoids.

PP10 and PP24 inhibited the cell viability of colorectal cancer organoids (**A**), lung cancer organoids (**B**) and liver cancer organoids (**C**) ($N = 3$). (**D**) Expression level of CD133 in pan-cancer patient-derived organoids, namely, Colorectal cancer, Lung cancer, Liver cancer, Thyroid cancer, and Breast cancer. Scale bar = 50 µm. one-way ANOVA test. Data are presented as mean ± SD. *$P < 0.05$, **$P < 0.01$, and ***$P < 0.001$, when compared with control group.

 © The Author(s)

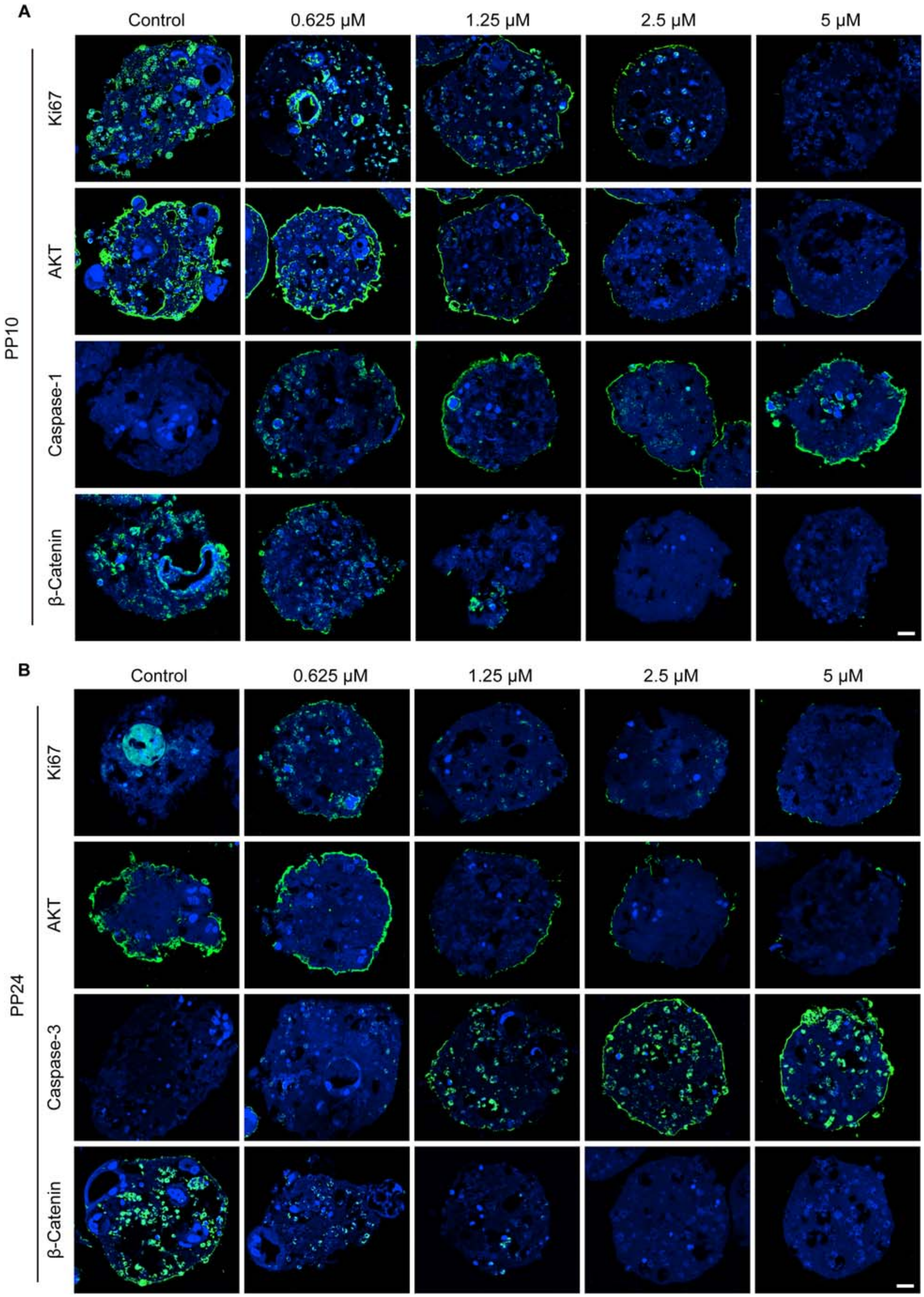
A
Control
0.625 μM
1.25 μM
2.5 μM
5 μM
PP10
Ki67
AKT
Caspase-1
β-Catenin
B
Control
0.625 μM
1.25 μM
2.5 μM
5 μM
PP24
Ki67
AKT
Caspase-3
β-Catenin

Figure EV7. Immunofluorescence staining of organoid.

(**A**) Immunofluorescence staining of Ki67, AKT, Caspase-1, and β-catenin in CRC organoids after treatment with PP10. (**B**) Immunofluorescence staining of frozen sections of CRC organoids after treatment with PP24 on Ki67, AKT, Caspase-3, and β-Catenin. Scale bar, 50 μm.

© The Author(s)
